# Supplementary material for: Diminishing benefits of urban living for children and adolescents’ growth and development
Source: Nature. 2023 Mar 29;615(7954):874–83. doi: 10.1038/s41586-023-05772-8 (PMC10060164; doi:10.1038/s41586-023-05772-8)
Supplement: Supplementary file 1 — Supplementary Tables 1–4, Supplementary Figs. 1–8 and Supplementary References; see contents page for details. [file 41586_2023_5772_MOESM1_ESM.pdf]

nature portfolio

https://doi.org/10.1038/s41586-023-05772-8

Supplementary information

Diminishing benefits of urban living for children and adolescents' growth and development

In the format provided by the authors and unedited

Supplementary Table 1. List of analysis regions and super-regions, and countries in each region.

Super-region	Region
**Central and eastern Europe**	**Central Europe**: Albania, Bosnia and Herzegovina, Bulgaria, Croatia, Czechia, Hungary, Montenegro, North Macedonia, Poland, Romania, Serbia, Slovakia, Slovenia
	**Eastern Europe**: Belarus, Estonia, Latvia, Lithuania, Moldova, Russian Federation, Ukraine
**Central Asia, Middle East and north Africa**	**Central Asia**: Armenia, Azerbaijan, Georgia, Kazakhstan, Kyrgyzstan, Mongolia, Tajikistan, Turkmenistan, Uzbekistan
	**Middle East and north Africa**: Algeria, Bahrain, Egypt, Iran, Iraq, Jordan, Kuwait, Lebanon, Libya, Morocco, Oman, Qatar, Saudi Arabia, State of Palestine, Syrian Arab Republic, Tunisia, Turkey, United Arab Emirates, Yemen
**East and southeast Asia**	**East Asia**: China, North Korea, Taiwan
	**Southeast Asia**: Brunei Darussalam, Cambodia, Indonesia, Lao PDR, Malaysia, Maldives, Myanmar, Philippines, Thailand, Timor-Leste, Viet Nam
**High-income Asia Pacific**	**High-income Asia Pacific**: Japan, Singapore, South Korea
**High-income western**	**High-income English-speaking countries***: Australia, Canada, Ireland, New Zealand, United Kingdom, United States of America
	**Northwestern Europe**: Austria, Belgium, Denmark, Finland, Germany, Greenland, Iceland, Luxembourg, Netherlands, Norway, Sweden, Switzerland
	**Southwestern Europe**: Andorra, Cyprus, France, Greece, Israel, Italy, Malta, Portugal, Spain
**Latin America and Caribbean**	**Andean Latin America**: Bolivia, Ecuador, Peru
	**Caribbean**: Antigua and Barbuda, Bahamas, Barbados, Belize, Bermuda, Cuba, Dominica, Dominican Republic, Grenada, Guyana, Haiti, Jamaica, Puerto Rico, Saint Kitts and Nevis, Saint Lucia, Saint Vincent and the Grenadines, Suriname, Trinidad and Tobago
	**Central Latin America**: Colombia, Costa Rica, El Salvador, Guatemala, Honduras, Mexico, Nicaragua, Panama, Venezuela
	**Southern Latin America**: Argentina, Brazil, Chile, Paraguay, Uruguay
**Oceania**	**Melanesia:** Fiji, Papua New Guinea, Solomon Islands, Vanuatu
	**Polynesia and Micronesia**: American Samoa, Cook Islands, French Polynesia, Kiribati, Marshall Islands, Micronesia (Federated States of), Nauru, Niue, Palau, Samoa, Tokelau, Tonga, Tuvalu

**South Asia**	**South Asia**: Afghanistan, Bangladesh, Bhutan, India, Nepal, Pakistan, Sri Lanka
**Sub-Saharan Africa**	**Central Africa**: Angola, Central African Republic, Congo, DR Congo, Equatorial Guinea, Gabon
	**East Africa**: Burundi, Comoros, Djibouti, Eritrea, Ethiopia, Kenya, Madagascar, Malawi, Mauritius, Mozambique, Rwanda, Seychelles, Somalia, South Sudan, Sudan, Tanzania, Uganda, Zambia
	**Southern Africa**: Botswana, Eswatini, Lesotho, Namibia, South Africa, Zimbabwe
	**West Africa**: Benin, Burkina Faso, Cabo Verde, Cameroon, Chad, Cote d'Ivoire, Gambia, Ghana, Guinea, Guinea Bissau, Liberia, Mali, Mauritania, Niger, Nigeria, Sao Tome and Principe, Senegal, Sierra Leone, Togo

* Although high-income English-speaking countries are geographically separated, they experienced remarkably similar trends in cardiometabolic risk factors and outcomes[1-5]. They were therefore grouped together so that the statistical model shares information amongst them more than it does with other countries that are geographically closer but epidemiologically more distinct.

We did not have data on population by age group for American Samoa, Bermuda, Greenland, and Tokelau. Country-specific estimates were made but were not used in calculation of regional and global prevalence because the latter requires weighting by age-specific population.

Supplementary Table 2. Data sources used in the analysis.

Country	Study years	Survey/study name/citation	Level of representativeness	Rural, urban or both	Age range as in NCD-RisC database *		Sample size (height)		Sample size (BMI)		Note
					Female	Male	Female	Male	Female	Male	
**Central and eastern Europe**											
Central Europe											
Albania	2013	Childhood Obesity Surveillance Initiative 3	National	both	7-9	7-9	2794	2971	2794	2971	
Albania	2013-2015	Balkan Survey of Inactivity in Children (BASIC)	Subnational	both	5-16	5-16	4985	4986	4982	4986	
Albania	2015-2016	Childhood Obesity Surveillance Initiative 4	National	both	7-9	7-9	3062	3359	3062	3358	
Albania	2017-2018	DHS	National	both	15-59	15-59	4557	2044	1649	727	
Bosnia and Herzegovina	2002	Non-communicable disease risk factor survey, Federation of B&H	Subnational	both	25-64	25-64	148	73			
Bosnia and Herzegovina	2012	Non-communicable disease risk factor survey, Federation of B&H	Subnational	both	18+	18+	371	408	55	72	
Bulgaria	1993	Anthropometric characterization of growth and development in children aged 7 to 13 years from Sofia at the beginning of the 21st century	Community	urban	6-7	6-7	119	109	119	109	
Bulgaria	1994	Anthropometric characterization of growth and development in children aged 7 to 13 years from Sofia at the beginning of the 21st century	Community	urban	7-8	7-8	118	108	118	108	
Bulgaria	1995	Anthropometric characterization of growth and development in children aged 7 to 13 years from Sofia at the beginning of the 21st century	Community	urban	8-9	8-9	120	109	120	109	
Bulgaria	1996	Anthropometric characterization of growth and development in children aged 7 to 13 years from Sofia at the beginning of the 21st century	Community	urban	9-10	9-10	119	109	119	109	
Bulgaria	1997	Anthropometric characterization of growth and development in children aged 7 to 13 years from Sofia at the beginning of the 21st century	Community	urban	10-11	10-11	119	109	119	109	
Bulgaria	1998	Anthropometric characterization of growth and development in children aged 7 to 13 years from Sofia at the beginning of the 21st century	Community	urban	11-12	11-12	118	109	118	109	
Bulgaria	1995-2001	Anthropometric characterization of growth and development in children aged 7 to17 years from Sofia at the beginning of the 21st century	Community	urban	7-17	7-17	1132	1128	1132	1127	
Bulgaria	1999	Anthropometric characterization of growth and development in children aged 7 to 13 years from Sofia at the beginning of the 21st century	Community	urban	12-13	12-13	118	109	118	109	
Bulgaria	2004-2005	Anthropometric characterization of growth and development in children aged 3 to 6 years from Sofia at the beginning of the 21st century	Community	urban	5-6	5-6	160	160	160	160	
Bulgaria	2008	Childhood Obesity Surveillance Initiative 1	National	both	7-8	7-8	1661	1657	1661	1657	
Bulgaria	2013	Childhood Obesity Surveillance Initiative 3	National	both	7	7	1677	1671	1677	1671	
Bulgaria	2014	Bulgarian National Monitoring of Dietary Intake	National	both	20+	20+	224	254			
Bulgaria	2015-2016	Childhood Obesity Surveillance Initiative 4	National	both	7	7	1698	1702	1698	1702	
Bulgaria	2016	Feel4Diabetes	Community	urban	6-10	6-10	1522	1447	1522	1447	
Bulgaria	2016-2017	Erasmus plus KA2, Healthyland	Community	urban	5	5	24	26	24	26	
Bulgaria	2017-2018	Erasmus plus KA2, Healthyland	Community	urban	5-6	5-6	51	49	51	49	
Bulgaria	2019	Childhood Obesity Surveillance Initiative 5	National	both	7	7	1531	1537	1531	1536	
Croatia	2003	Croatian Adult Health Survey 2003	National	both	18+	18+	627	241			
Croatia	2003-2004	School Health Survey	National	both	6-19	6-19	1297	1502	1297	1502	
Croatia	2002-2007	Epidemiology of arterial hypertension in Croatia (EH-UH)	National	both	18+	18+	48	45			
Croatia	2005	Endemic Nephropathy and Arterial hypertension (ENAH)	Subnational	rural	18+	18+	45	37	3		
Croatia	2006-2008	The cardiovascular risk factors in school age - intervention model development	National	both	6-20	6-20	5625	6012	5625	6011	
Croatia	2008	Endemic Nephropathy and Arterial hypertension (ENAH)	Subnational	rural	18+	18+	63	45	8	10	
Croatia	2010	Endemic Nephropathy and Arterial hypertension (ENAH)	Subnational	rural	18+	18+	54	30	5	3	
Croatia	2014	Croatian Physical Activity in Adolesence Longitudinal Study (CRO-PALS)	Community	urban	14-17	14-17	413	429	410	428	
Croatia	2015	Endemic Nephropathy and Arterial hypertension (ENAH) Follow-up Study	Subnational	rural	18+	18+	15	10			
Croatia	2015-2016	Childhood Obesity Surveillance Initiative 4	National	both	8	8	1365	1365	1364	1364	
Croatia	2016-2017	Croatian Physical Activity in Adolesence Longitudinal Study (CRO-PALS)	Community	urban	17-20	17-20	384	383	384	383	
Croatia	2015-2019	Motor skills in preschool children	National	both	5-7	5-7	381	407	381	405	
Croatia	2019	Childhood Obesity Surveillance Initiative 5	National	both	8-9	8-9	2678	2779	2677	2778	
Croatia	2018-2021	Epidemiology of arterial hypertension in Croatia (EH-UH)	National	both	18+	18+	35	16			
Czechia	1981	4th Nationwide Anthropometric Survey of Children and Adolescents 1981	National	both	5-17	5-17	22816	21972			
Czechia	1991	5th Nationwide Anthropometric Survey of Children and Adolescents 1991	National	both	5-17	5-17	35659	34641			
Czechia	1997-1998	Czech post-MONICA	National	both	25-64	25-64	159	140			
Czechia	2000-2001	Czech post-MONICA	National	both	25-64	25-64	166	151			
Czechia	2001	6th Nationwide Anthropometric Survey of Children and Adolescents 2001	National	both	5-20	5-19			22523	18960	
Czechia	2008	Childhood Obesity Surveillance Initiative 1	National	both	6-7	6-7	796	800	796	800	
Czechia	2006-2009	Czech post-MONICA	National	both	25-64	25-64	155	135			
Czechia	2010	Childhood Obesity Surveillance Initiative 2	National	both	6-7	6-7	1207	1181	1207	1181	
Czechia	2013	Childhood Obesity Surveillance Initiative 3	National	both	6-7	6-7	1153	1229	1153	1229	
Czechia	2014-2015	European Heath Examination Survey	National	both	25-64	25-64	49	43			
Czechia	2015-2016	Childhood Obesity Surveillance Initiative 4	National	both	6-7	6-7	833	771	832	771	
Czechia	2015-2018	Czech post-MONICA	National	both	25-64	25-64	87	61			
Czechia	2020	Childhood Obesity Surveillance Initiative 5	National	both	6-7	6-7	1097	1136	1097	1136	
Czechia	2019-2020	European Heath Examination Survey	National	both	25-64	25-64	36	25			
Hungary	2006-2007	HELENA	Community	urban	12-17	12-17	197	197	197	197	
Hungary	2007-2010	Identification and prevention of Dietary- and lifestyle-induced health EFfects In Children and infantS (IDEFICS)	Community	urban	5-9	5-9	1169	1128	1169	1128	
Hungary	2010	Childhood Obesity Surveillance Initiative 2	National	both	7	7	682	553	682	553	
Hungary	2010	EuropeaN Energy balance Research to prevent excessive weight Gain among Youth - The ENERGY-project	National	both	11-13	11-13	558	452	557	452	
Hungary	2015-2016	Childhood Obesity Surveillance Initiative 4	National	both	6-8	6-8	2579	2753	2572	2750	
Hungary	2016	Feel4Diabetes	Community	both	6-10	6-10	1522	1447	1522	1447	
Hungary	2016	Hungarian National Student Fitness Test (NETFIT)	National	both	14-19	10-19	132739	134442	132326	134129	
Hungary	2016	Hungarian National Student Fitness Test (NETFIT)	National	both	42278	42278	158353	165185	158087	164897	
Hungary	2017	Hungarian National Student Fitness Test (NETFIT)	National	both	14-19	10-19	134845	136597	134646	136278	
Hungary	2017	Hungarian National Student Fitness Test (NETFIT)	National	both	42278	42278	163787	171217	163599	170978	
Hungary	2018	Hungarian National Student Fitness Test (NETFIT)	National	both	14-19	10-19	128131	129327	127821	128717	
Hungary	2018	Hungarian National Student Fitness Test (NETFIT)	National	both	42278	42278	163083	170447	162664	169915	
Hungary	2019	Childhood Obesity Surveillance Initiative 5	National	both	6-8	6-8	2996	3173	2996	3173	

Country	Study years	Survey/study name/citation	Level of representativeness	Rural, urban or both	Age range as in NCD-RisC database *		Sample size (height)		Sample size (BMI)		Note
					Female	Male	Female	Male	Female	Male	
Hungary	2019	Hungarian National Student Fitness Test (NETFIT)	National	both	14-19	10-19	130226	132673	130182	132647	
Hungary	2019	Hungarian National Student Fitness Test (NETFIT)	National	both	42278	42278	165219	171735	165199	171701	
Montenegro	1987	Anthropometric Characteristics of Montenegrin Recruiters	National	both		17-28				9934	1
Montenegro	1988	Anthropometric Characteristics of Montenegrin Recruiters	National	both		17-28				86	1
Montenegro	2016	Anthropometric parameters as an indicator of obesity at adolescents in Montenegro	National	both	14-18	14-18	771	678	771	678	
Montenegro	2015-2016	Childhood Obesity Surveillance Initiative 4	National	both	6-8	6-8	1623	1802	1623	1802	
Montenegro	2019	Body composition of high school students in Montenegro and its relationship with their eating habits	National	both	18-20	18-20	497	504	495	501	
Montenegro	2019	Childhood Obesity Surveillance Initiative 5	National	both	6-8	6-8	1626	1729	1626	1729	
Montenegro	2019	Initiative for monitoring obesity of children aged 11 to 12 in Montenegro	National	both	11-12	11-12	619	666	619	666	
Montenegro	2019	Initiative for monitoring obesity of children aged 5 to 6 in Montenegro and Slovenia	National	both	5-6	5-6	214	231	214	231	
Montenegro	2018-2019	Initiative for monitoring obesity of children aged 6 to 9 in Montenegro and Slovenia	National	both	6-9	6-9	969	1072	969	1072	
North Macedonia	1999	Multiple Indicator Cluster Survey	National	both	15-45		733		46		
North Macedonia	2009	Annual assessment of nutritional status of school children aged 7 years	National	both	7	7	983	1088	982	1087	
North Macedonia	2010	Childhood Obesity Surveillance Initiative 2	National	both	7	7	1311	1427	1311	1427	
North Macedonia	2013	Childhood Obesity Surveillance Initiative 3	National	both	6-7	6-7	1511	1655	1511	1655	
North Macedonia	2015-2016	Childhood Obesity Surveillance Initiative 4	National	both	7-8	7-8	1727	1809	1727	1809	
North Macedonia	2019	Childhood Obesity Surveillance Initiative 5	National	both	7-8	7-8	1614	1580	1611	1579	
Poland	1988	Fourth National Survey	Subnational	both	7-19	7-19	9808	11724			
Poland	1995	Poland Conscripts 10% Sample Cohort	National	both		18-19		31026		31007	
Poland	1995-1996	Polish Program CINDI (CINDI Lodz 1995)	Community	urban	17-64	17-64	387	300	111	79	
Poland	1997	Wroclaw survey in adolescents	Community	both	12-17	12-17	2136	2196	2136	2196	
Poland	2000	The health status, risk factors of chronic diseases and health behaviors of residents of Torun (CINDI Torun 2000)	Community	urban	16-83	16-83	264	256	85	111	
Poland	2000	Secular trends in BMI changes among the military population between 2000 and 2010 in Poland - a retrospective study	Subnational	both		19-20		3004		3003	
Poland	2001	Secular trends in BMI changes among the military population between 2000 and 2010 in Poland - a retrospective study	Subnational	both		19-20		3425		3420	
Poland	2001	Poland Conscripts 10% Sample Cohort	National	both		18-19		31253		31211	
Poland	2000-2001	Household Food Consumption and Anthropometric Survey	National	both	5+	5+	829	799	572	555	
Poland	2001-2002	The health status, risk factors of chronic diseases and health behaviors of residents of Lodz (CINDI Lodz 2001)	Community	urban	18-64	18-64	258	261	39	61	
Poland	2002	Secular trends in BMI changes among the military population between 2000 and 2010 in Poland - a retrospective study	Subnational	both		19-20		3547		3544	
Poland	2002	NATPOL	National	both	18+	18+	222	201	34	32	
Poland	2003	Secular trends in BMI changes among the military population between 2000 and 2010 in Poland - a retrospective study	Subnational	both		19-20		3637		3633	
Poland	2004	Secular trends in BMI changes among the military population between 2000 and 2010 in Poland - a retrospective study	Subnational	both		19-20		3540		3538	
Poland	2003-2005	National Multicenter Health Survey in Poland. Project WOBASZ	National	both	20-74	20-74	1379	1204			
Poland	2005	Secular trends in BMI changes among the military population between 2000 and 2010 in Poland - a retrospective study	Subnational	both		19-20		3308		3308	
Poland	2003-2006	Mogielica Human Ecology Study Site	Community	rural	18+	18+	60	8			
Poland	2006	The health, risk factors for chronic diseases, attitudes and behaviors of health residents of Torun (CINDI Torun 2006)	Community	urban	15-65	15-65	257	194	74	70	
Poland	2006	Secular trends in BMI changes among the military population between 2000 and 2010 in Poland - a retrospective study	Subnational	both		19-20		3702		3701	
Poland	2007	Secular trends in BMI changes among the military population between 2000 and 2010 in Poland - a retrospective study	Subnational	both		19-20		3612		3612	
Poland	2008	Secular trends in BMI changes among the military population between 2000 and 2010 in Poland - a retrospective study	Subnational	both		19-20		3435		3435	
Poland	2007-2009	Elaboration of the reference range of arterial blood pressure for the population of children and adolescents in Poland - PL0080 OLAF	National	both	6-18	6-18	9172	8386	9162	8383	
Poland	2009	Secular trends in BMI changes among the military population between 2000 and 2010 in Poland - a retrospective study	Subnational	both		19-20		3407		3405	
Poland	2007-2010	Mogielica Human Ecology Study Site	Community	rural	18+	18+	50				
Poland	2010	Secular trends in BMI changes among the military population between 2000 and 2010 in Poland - a retrospective study	Subnational	both		19-20		3317		3317	
Poland	2009-2010	Poland Conscripts 10% Sample Cohort	National	both		18-19		8347		8344	
Poland	2011	NATPOL	National	both	18-79	18-79	266	239	29	39	
Poland	2010-2012	Blood pressure references for Polish preschool children - the OLA study	National	both	5-6	5-6	905	926	904	926	
Poland	2012-2013	Fifth National Survey	Subnational	both	6-19	6-19	2678	3083	2677	3074	
Poland	2011-2014	Mogielica Human Ecology Study Site	Community	rural	18+	18+	8				
Poland	2014	Prevalence of risk factors for obesity and hypertension among Polish children and adolescents	Subnational	both	7-18	7-18	283	285	283	284	
Poland	2013-2014	National Multicenter Health Survey in Poland. Project WOBASZ II	National	both	20+	20+	408	392			
Poland	2016	Body fat in Polish adolescents	Community	urban	11-13	11-13	82	76	82	76	
Poland	2014-2017	The impact of physical activity and selected perinatal risk factors on the occurrence of overweight and obesity and hypertension in children	Subnational	both	5-15	5-15	454	516	454	516	
Poland	2015-2016	Childhood Obesity Surveillance Initiative 4	National	both	8	8	1665	1671	1662	1671	
Poland	2015-2016	LIPIDOGRAM2015 & LIPIDOGEN2015 Study - National epidemiological study of lipid disorders and selected risk factors of cardiovascular disease in primary health care in Poland	National	both	18+	18+	356	214	21	24	
Poland	2016-2017	Erasmus plus KA2, Healthyland	Community	urban	6	6	25	25	25	25	
Poland	2017	Multivariate assessment of the occurrence of noncommunicable diseases and their risk factors among preschool children	Community	urban	5-7	5-7	206	226	206	226	
Poland	2016-2017	The occurrence of overweight and obesity in children from different place of residence	Subnational	both	7-13	7-13	151	164	151	164	
Poland	2017	Preferences for sweet and fatty taste in children and their mothers in association with weight status	Subnational	both	8-15	8-15	150	138	150	138	
Poland	2017	Risk factors for obesity among Polish adolescents	Community	both	14-19	14-19	36	39	36	39	
Poland	2018	Childhood Obesity Surveillance Initiative 5	National	both	8	8	1303	1387	1303	1387	
Poland	2017-2018	Erasmus plus KA2, Healthyland	Community	urban	5-6	5-6	26	24	26	24	
Poland	2018	Multivariate assessment of the occurrence of noncommunicable diseases and their risk factors among preschool children	Community	urban	5-7	5-7	385	393	385	393	
Poland	2016-2020	The prevalence of overweight and obesity and the assessment of body balance among children in a rural area in Poland	Subnational	rural	7-15	7-15	557	580	557	580	
Poland	2018	Mogielica Human Ecology Study Site	Community	rural	18+	18+	8	4			
Poland	2019	Multivariate assessment of the occurrence of noncommunicable diseases and their risk factors among preschool children	Community	urban	5-7	5-7	375	395	375	395	
Poland	2018-2020	Child of Kraków 2020	Community	urban	5-18	5-18	1487	1382	1487	1382	

Country	Study years	Survey/study name/citation	Level of representativeness	Rural, urban or both	Age range as in NCD-RisC database *		Sample size (height)		Sample size (BMI)		Note
					Female	Male	Female	Male	Female	Male	
Poland	2019-2020	Assessment of the olfactory and taste functions of children with type 1 diabetes	Community	rural	10-15	10-15	46	54	46	54	
Poland	2019-2020	Child health status and risk factors	Subnational	both	6-17	6-17	325	370	325	370	
Romania	1999	Romania physical development	National	both	5-18	5-18	43675	41890	43648	41875	
Romania	2006-2008	Hypertension in Romanian Children and Adolescents: A Cross-Sectional Survey	Subnational	both	5-17	5-17	2339	2313	2339	2313	
Romania	2008	Healthy traditions for healthy children	Community	rural	7-11	7-11	69	74	69	74	
Romania	2008-2009	Healthy traditions for healthy children	Community	urban	7-11	7-11	525	562	525	562	
Romania	2009-2011	Study on children in Dolj County, South Romania	Subnational	both	5-21	5-21	672	746	665	734	
Romania	2010-2013	Healthy traditions for healthy children	Community	urban	5-11	5-11	1798	1736	1798	1736	
Romania	2011-2012	Study for the Evaluation of Prevalence of Hypertension and cArdiovascular Risk among the Adult Population of Romania - SEPHAR II	National	both	18-80	18-80	124	134	5	4	
Romania	2013	Childhood Obesity Surveillance Initiative 3	National	both	8	8	2173	2175	2173	2175	
Romania	2013	Healthy traditions for healthy children	Subnational	rural	5-11	5-11	107	123	107	123	
Romania	2013-2014	Auxological evaluation of school children in Mures County	Subnational	both	6-14	6-14	957	936	957	936	
Romania	2014	Timis County Study	Community	urban	6-19	6-19	205	238	205	237	
Romania	2014-2015	Healthy traditions for healthy children	Subnational	rural	5-10	5-10	561	599	561	599	
Romania	2015	Healthy traditions for healthy children	Community	urban	5-10	5-10	211	183	210	183	
Romania	2015-2016	Childhood Obesity Surveillance Initiative 4	National	both	7-9	7-9	3674	3779	3595	3699	
Romania	2015-2016	Study for the Evaluation of Prevalence of Hypertension and cArdiovascular Risk among the Adult Population of Romania - SEPHAR III	National	both	18-80	18-80	184	154	26	26	
Romania	2016-2017	Erasmus plus KA2, Healthyland	Community	urban	5-6	5-6	16	14	16	14	
Romania	2017-2018	Erasmus plus KA2, Healthyland	Community	urban	5-7	5-7	34	31	34	31	
Romania	2018	Healthy traditions for healthy children	Community	both	5-13	5-13	1085	1144	1085	1142	
Romania	2019	Childhood Obesity Surveillance Initiative 5	National	both	7-9	7-9	5142	5234	5142	5234	
Romania	2019	Resita preschool measurements	Community	urban	5-8	5-8	236	232	236	232	
Serbia	2000	Health Status, Health Needs and Utilization of Health Care of the Population of Serbia	National	both	7+	7+	1933	1624	932	884	
Serbia	2006	The 2006 National Health Survey for the Population of Serbia	National	both	7+	7+	2382	2361	1303	1331	
Serbia	2013	The 2013 National Health Survey for the Population of Serbia	National	both	7+	7+	2018	1970	1091	1097	
Serbia	2013-2014	Stay Fit for Lifelong Health; the Prevalence of Lifestyle Health Conditions in Serbian Population	National	urban	18-65	18-65	42	217		4	
Serbia	2015-2016	Childhood Obesity Surveillance Initiative 4	National	both	6-8	6-8	2386	2475	2386	2475	
Serbia	2019	Childhood Obesity Surveillance Initiative 5	National	both	7-9	7-9	1504	1697	1504	1697	
Slovakia	1985	Effects of somatic development and environmental factors on blood pressure in children	Community	urban	5-7	5-7	388	412			
Slovakia	1993	Countrywide Integrated Noncommunicable Diseases Intervention Programme	National	both	15-64	15-64	135	199	135	199	
Slovakia	1998	Countrywide Integrated Noncommunicable Diseases Intervention Programme	National	both	15-64	15-64	289	249	122	112	
Slovakia	2001	National Anthropological Survey	National	both	5-6	5-6	806	882	806	882	
Slovakia	2001	National Anthropological Survey	National	both	6-18	6-18	10737	10883	10667	10881	
Slovakia	2003	Countrywide Integrated Noncommunicable Diseases Intervention Programme	National	both	15-64	15-64	200	188	60	89	
Slovakia	2008	Countrywide Integrated Noncommunicable Diseases Intervention Programme	National	both	15-64	15-64	108	92	36	36	
Slovakia	2011	National Anthropological Survey	National	both	7-18	7-18	9028	9066	9028	9064	
Slovakia	2011-2012	European Health Examination Survey	National	both	18-64	18-64	240	213	68	46	
Slovakia	2015-2016	Childhood Obesity Surveillance Initiative 4	National	both	7	7	1382	1390	1379	1390	
Slovakia	2018	Childhood Obesity Surveillance Initiative 5	National	both	7-8	7-8	3005	2975	3004	2975	
Slovenia	1982	the SLOFIT monitoring system	National	both	6-19	6-19	8583	8817			
Slovenia	1983	Analysis of Children's Development in Slovenia (ACDSi)	National	both	7-14	7-14	1200	1160			
Slovenia	1983	the SLOFIT monitoring system	National	both	6-19	6-19	10177	10718			
Slovenia	1984	the SLOFIT monitoring system	National	both	6-19	6-19	16221	17052			
Slovenia	1985	the SLOFIT monitoring system	National	both	6-19	6-19	19112	20403			
Slovenia	1986	the SLOFIT monitoring system	National	both	6-19	6-19	20061	21082			
Slovenia	1987	the SLOFIT monitoring system	National	both	6-19	6-19	46934	48114	48784	49089	1
Slovenia	1988	the SLOFIT monitoring system	National	both	6-19	6-19	82328	84750	83390	85444	1
Slovenia	1989	the SLOFIT monitoring system	National	both	6-19	6-19	106316	106245	106639	106538	1
Slovenia	1990	the SLOFIT monitoring system	National	both	6-19	6-19	128572	129317	128572	129317	
Slovenia	1991	the SLOFIT monitoring system	National	both	6-19	6-19	129843	130726	129842	130726	
Slovenia	1992	the SLOFIT monitoring system	National	both	6-19	6-19	134855	135243	134853	135239	
Slovenia	1993	Analysis of Children's Development in Slovenia (ACDSi)	National	both	6-14	6-14	1678	1674	1678	1674	
Slovenia	1993	the SLOFIT monitoring system	National	both	6-19	6-19	141967	143183	141966	143182	
Slovenia	1994	Analysis of Children's Development in Slovenia (ACDSi)	National	both	14-18	14-18	696	683	696	683	
Slovenia	1994	the SLOFIT monitoring system	National	both	6-19	6-19	143290	145348	143290	145348	
Slovenia	1995	the SLOFIT monitoring system	National	both	6-19	6-19	140692	142246	140692	142245	
Slovenia	1996	the SLOFIT monitoring system	National	both	6-19	6-19	138122	140718	138120	140714	
Slovenia	1997	the SLOFIT monitoring system	National	both	6-19	6-19	120331	127551	120331	127551	
Slovenia	1998	the SLOFIT monitoring system	National	both	6-19	6-19	121550	126342	121549	126342	
Slovenia	1999	the SLOFIT monitoring system	National	both	6-19	6-19	119408	122655	119408	122655	
Slovenia	2000	the SLOFIT monitoring system	National	both	6-19	6-19	116281	121918	116281	121918	
Slovenia	2001	the SLOFIT monitoring system	National	both	6-19	6-19	114607	118997	114606	118997	
Slovenia	2002	the SLOFIT monitoring system	National	both	6-19	6-19	110691	114716	110691	114716	
Slovenia	2003	Analysis of Children's Development in Slovenia (ACDSi)	National	both	5-14	5-14	1939	2061	1939	2061	
Slovenia	2003	the SLOFIT monitoring system	National	both	6-19	6-19	112944	117278	112944	117278	
Slovenia	2004	Analysis of Children's Development in Slovenia (ACDSi)	National	both	14-19	14-19	712	950	712	950	

Country	Study years	Survey/study name/citation	Level of representativeness	Rural, urban or both	Age range as in NCD-RisC database *		Sample size (height)		Sample size (BMI)		Note
					Female	Male	Female	Male	Female	Male	
Slovenia	2004	the SLOFIT monitoring system	National	both	6-19	6-19	112576	117768	112575	117768	
Slovenia	2005	the SLOFIT monitoring system	National	both	6-19	6-19	109546	114966	109542	114966	
Slovenia	2006	the SLOFIT monitoring system	National	both	6-19	6-19	102840	109878	102838	109877	
Slovenia	2007	the SLOFIT monitoring system	National	both	6-19	6-19	100366	103108	100364	103105	
Slovenia	2008	the SLOFIT monitoring system	National	both	6-19	6-19	98071	103736	98070	103735	
Slovenia	2009	the SLOFIT monitoring system	National	both	6-19	6-19	98894	103673	98891	103669	
Slovenia	2010	the SLOFIT monitoring system	National	both	6-19	6-19	96687	101165	96686	101163	
Slovenia	2011	the SLOFIT monitoring system	National	both	6-19	6-19	96314	100811	96309	100802	
Slovenia	2012	the SLOFIT monitoring system	National	both	6-19	6-19	95917	101178	95917	101172	
Slovenia	2013	the SLOFIT monitoring system	National	both	6-19	6-19	95669	99198	95666	99191	
Slovenia	2013-2014	Analysis of Children's Development in Slovenia (ACDSi)	National	both	6-15	6-15	1627	1665	1627	1665	
Slovenia	2014	Analysis of Children's Development in Slovenia (ACDSi)	National	both	14-19	14-19	724	703	724	702	
Slovenia	2014	the SLOFIT monitoring system	National	both	6-21	6-21	97864	102793	97736	102558	
Slovenia	2015	the SLOFIT monitoring system	National	both	6-19	6-19	99295	103570	99293	103568	
Slovenia	2016	the SLOFIT monitoring system	National	both	6-19	6-19	102180	107427	102174	107421	
Slovenia	2017	the SLOFIT monitoring system	National	both	6-19	6-19	104582	108784	104582	108780	
Slovenia	2018	the SLOFIT monitoring system	National	both	6-19	6-19	105866	110216	105861	110213	
Slovenia	2019	the SLOFIT monitoring system	National	both	6-19	6-19	94793	99448	94791	99445	
Eastern Europe											
Belarus	2016-2017	STEPS	National	both	18-69	18-69	344	331	29	32	
Estonia	1997	Pomerleau et al., Public Health Nutrition 3(1):3-10, 2000	National	both	19-64	19-64	224	183	11	13	
Estonia	2002	Estonian Biobank	National	both	18+	18+	53	21	10	5	
Estonia	2003	Estonian Biobank	National	both	18+	18+	1114	646	186	153	
Estonia	2004	Estonian Biobank	National	both	18+	18+	111	74	22	18	
Estonia	2007	Estonian Biobank	National	both	18+	18+	465	243	95	53	
Estonia	2008	Estonian Biobank	National	both	18+	18+	2766	1592	501	315	
Estonia	2009	Estonian Biobank	National	both	18+	18+	1324	1131	282	204	
Estonia	2007-2010	Identification and prevention of Dietary- and lifestyle-induced health EFfects In Children and infantS (IDEFICS)	Community	urban	5-9	5-9	634	558	634	558	
Estonia	2010	Estonian Biobank	National	both	18+	18+	1609	1320	301	284	
Estonia	2013-2015	National Dietary Survey (RTU) 2014	National	both	5-74	5-74	753	574	475	400	
Estonia	2015-2016	Childhood Obesity Surveillance Initiative 4	National	both	7-8	7-8	6196	6495	6192	6489	
Estonia	2018-2019	Childhood Obesity Surveillance Initiative 5	National	both	7-11	7-11	6036	6061	6036	6060	
Latvia	2008	Childhood Obesity Surveillance Initiative 1	National	both	7-8	7-8	2101	2283	2101	2283	
Latvia	2008-2009	Cardiovascular risk factor study	National	both	25-74	25-74	218	135			
Latvia	2010	Childhood Obesity Surveillance Initiative 2	National	both	7-8	7-8	2053	2093	2053	2093	
Latvia	2012	Childhood Obesity Surveillance Initiative 3	National	both	6-7	6-7	1677	1804	1677	1804	
Latvia	2015-2016	Childhood Obesity Surveillance Initiative 4	National	both	7-9	7-9	2991	2952	2991	2952	
Latvia	2019	Childhood Obesity Surveillance Initiative 5	National	both	6-9	6-9	3441	3420	3441	3420	
Lithuania	1998-1999	Countrywide Integrated Noncommunicable Diseases Intervention Programme survey	Subnational	rural	25-64	25-64	61	48			
Lithuania	2002	Pomerleau et al., Public Health Nutrition 3(1):3-10, 2000	National	both	24-70	24-70	189	69			
Lithuania	2006-2007	Countrywide Integrated Noncommunicable Diseases Intervention Programme survey	Subnational	rural	25-64	25-64	74	45			
Lithuania	2008	Childhood Obesity Surveillance Initiative 1	National	both	7-8	7-8	2344	2532	2344	2532	
Lithuania	2010	Childhood Obesity Surveillance Initiative 2	National	both	7-9	7-9	3414	3306	3414	3306	
Lithuania	2010-2012	Prevalence and risk factors of high blood pressure in 12-15-year-old Lithuanian children and adolescents (Study 1, 2010-2012)	Community	both	12-15	12-15	3963	3494	3963	3494	
Lithuania	2013	Childhood Obesity Surveillance Initiative 3	National	both	7-8	7-8	1885	1878	1884	1878	
Lithuania	2012-2013	Prevalence and risk factors of high blood pressure in 12-15-year-old Lithuanian children and adolescents (Study 2, 2012-2013)	Community	both	12-15	12-15	985	962	985	962	
Lithuania	2015-2016	Childhood Obesity Surveillance Initiative 4	National	both	7-8	7-8	1681	1735	1665	1721	
Lithuania	2019	Childhood Obesity Surveillance Initiative 5	National	both	7-8	7-8	1569	1633	1569	1633	
Moldova	2005	DHS	National	both	15-49		3445		1364		
Moldova	2013	Childhood Obesity Surveillance Initiative 3	National	both	7-8	7-8	1751	1931	1751	1931	
Moldova	2013	STEPS	National	both	18-69	18-69	464	314	67	60	
Russian Federation	1992-1993	Russia Longitudinal Monitoring Survey- Higher School of Economics Round II	National	both	5+	5+	584	547	576	547	
Russian Federation	1993	Russia Longitudinal Monitoring Survey- Higher School of Economics Round III	National	both	5+	5+	1567	1643	1536	1631	
Russian Federation	1993-1994	Russia Longitudinal Monitoring Survey- Higher School of Economics Round IV	National	both	5+	5+	1655	1664	1605	1617	
Russian Federation	1994	Russia Longitudinal Monitoring Survey- Higher School of Economics Round V	National	both	5+	5+	1250	1278	1240	1268	
Russian Federation	1995	Russia Longitudinal Monitoring Survey- Higher School of Economics Round VI	National	both	5+	5+	1201	1212	1201	1210	
Russian Federation	1996	Russia Longitudinal Monitoring Survey- Higher School of Economics Round VII	National	both	5+	5+	1970	1845	1167	1179	
Russian Federation	1998-1999	Russia Longitudinal Monitoring Survey- Higher School of Economics Round VIII	National	both	5+	5+	2019	1918	1195	1202	
Russian Federation	2000	Russia Longitudinal Monitoring Survey- Higher School of Economics Round IX	National	both	5+	5+	2098	2019	1174	1225	
Russian Federation	2001	Russia Longitudinal Monitoring Survey- Higher School of Economics Round X	National	both	5+	5+	2326	2178	1269	1298	
Russian Federation	2002	Russia Longitudinal Monitoring Survey- Higher School of Economics Round XI	National	both	5+	5+	2372	2244	1291	1301	
Russian Federation	2002	Russian Karelia Survey in Pitkaranta	Community	both	25-64	25-64	40	35			
Russian Federation	2003	Russia Longitudinal Monitoring Survey- Higher School of Economics Round XII	National	both	5+	5+	2385	2284	1260	1315	
Russian Federation	2003	School Children Moscow	Community	urban	7-8	7-8	112	133	112	133	
Russian Federation	2004	Russia Longitudinal Monitoring Survey- Higher School of Economics Round XIII	National	both	5+	5+	2347	2225	1231	1267	
Russian Federation	2004	School Children Moscow	Community	urban	8-9	8-9	185	180	185	180	

Country	Study years	Survey/study name/citation	Level of representativeness	Rural, urban or both	Age range as in NCD-RisC database *		Sample size (height)		Sample size (BMI)		Note
					Female	Male	Female	Male	Female	Male	
Russian Federation	2005	Russia Longitudinal Monitoring Survey- Higher School of Economics Round XIV	National	both	5+	5+	2228	2156	1143	1214	
Russian Federation	2005	School Children Moscow	Community	urban	9-10	9-10	191	178	191	178	
Russian Federation	2006	School Children Moscow	Community	urban	10-11	10-11	195	163	195	163	
Russian Federation	2007	School Children Moscow	Community	urban	11-12	11-12	167	143	167	143	
Russian Federation	2007	Russian Karelia Survey in Pitkaranta	Community	both	25-64	25-64	26	17			
Russian Federation	2008	School Children Moscow	Community	urban	12-13	12-13	141	111	141	111	
Russian Federation	2009	School Children Moscow	Community	urban	13-14	13-14	140	124	140	124	
Russian Federation	2010	School Children Moscow	Community	urban	14-15	14-15	137	116	137	116	
Russian Federation	2011	School Children Moscow	Community	urban	15-16	15-16	125	117	125	117	
Russian Federation	2012	School Children Moscow	Community	urban	16-17	16-17	108	87	108	87	
Russian Federation	2012-2014	Epidemiology of Cardiovascular Diseases in Different Regions of Russia (ESSE-RF)	National	both	25-64	25-64	1249	1192			
Russian Federation	2015-2016	Childhood Obesity Surveillance Initiative 4	Community	urban	6-8	6-8	1529	1499	1529	1499	
Russian Federation	2017	Epidemiology of Cardiovascular Diseases in Different Regions of Russia - 2 (ESSE-RF-2)	Subnational	both	25-64	25-64	384	365			
Russian Federation	2020	Childhood Obesity Surveillance Initiative 5	Community	urban	7	7	1376	1310	1376	1310	
Russian Federation	2019-2020	Childhood Obesity Surveillance Initiative 5	Community	urban	6-7	6-7	1203	1201	1202	1201	
Russian Federation	2019-2021	Ural Children Eye Study	Community	urban	6-18	6-18	2053	1951	2052	1950	
Ukraine	2006-2007	Physical Development of School Children Ukraine	Community	rural	6-17	6-17	1336	1226	1336	1226	
Ukraine	2007-2008	Physical Development of School Children Ukraine	Community	urban	6-17	6-17	1126	1224	1126	1224	
Ukraine	2012	Epidemiological aspects of obesity and systemic hypertension among school children of Western Ukraine	Community	urban	10-17	10-17	257	271	257	271	
Ukraine	2013-2014	The prevalence of underweight, overweight and obesity in children and adolescents from Ukraine	National	both	6-18	6-18	7143	6596	7143	6596	
Ukraine	2018	Prevalence of obesity in Ukrainian children and adolescents	National	both	7-17	7-17	4667	4471	4667	4471	
Ukraine	2019	Prevalence of obesity in Ukrainian children and adolescents	National	both	7-17	7-17	4760	4245	4760	4245	
Ukraine	2019	STEPS	National	both	18-69	18-69	299	256	41	28	
**Central Asia, Middle East and north Africa**											
Central Asia											
Armenia	1998	The health and nutritional status of children and women in Armenia	National	both	18-45		1502		72		
Armenia	2000	DHS	National	both	15-49		2794		1090		
Armenia	2005	DHS	National	both	15-49	15-49	2982	577	1064	236	
Armenia	2015-2016	DHS	National	both	15-49		2640		707		
Armenia	2016	STEPS	National	both	18-69	18-69	270	168	26	14	
Armenia	2019	Childhood Obesity Surveillance Initiative 5	National	both	7-8	7-8	1712	1875	1703	1860	
Azerbaijan	1996	Health and Nutrition Survey	National	both	19-59	19-59	137	34	3		
Azerbaijan	2006	DHS	National	both	15-49	15-59	3814	984	1402	368	
Azerbaijan	2013	Azerbaijan Nutrition Survey	National	both	15-49		1339		399		
Azerbaijan	2017	STEPS	National	both	18-69	18-69	252	212	28	21	
Georgia	2010	STEPS	National	both	18-64	18-64	678	392	144	97	
Georgia	2015-2016	Childhood Obesity Surveillance Initiative 4	National	both	7-8	7-8	1585	1687	1585	1685	
Georgia	2016	STEPS	National	both	18-69	18-69	352	204	49	26	
Georgia	2019	Childhood Obesity Surveillance Initiative 5	National	both	7-8	7-8	1646	1777	1646	1777	
Kazakhstan	1995	DHS	National	both	15-49		650		638		
Kazakhstan	1999	DHS	National	both	15-49		1027		341		
Kazakhstan	2015	Almaty STEPS	Subnational	both	18-69	18-69	171	65	8	7	
Kazakhstan	2015	Shymkent STEPS	Subnational	both	18-69	18-69	152	90	22	19	
Kazakhstan	2015-2016	Aktobe STEPS	Subnational	both	18-69	18-69	179	73	12	3	
Kazakhstan	2015-2016	Childhood Obesity Surveillance Initiative 4	National	both	8-10	8-10	2683	2756	2683	2755	
Kazakhstan	2019	A health status assessment of a population of Karaganda urban region	Community	urban	18+	18+	92	67	6		
Kazakhstan	2018-2019	Early diagnosis of metabolic syndrome in children and adolescents of Semey Region	Subnational	both	6-18	6-18	963	1008	963	1008	
Kazakhstan	2020	Childhood Obesity Surveillance Initiative 5	National	both	6-9	6-9	3379	3396	3379	3396	
Kyrgyzstan	1997	DHS	National	both	15-49		1904		699		
Kyrgyzstan	2012	DHS	National	both	15-49		4343		1526		
Kyrgyzstan	2013	STEPS	National	both	25-64	25-64	221	134			
Kyrgyzstan	2015-2016	Childhood Obesity Surveillance Initiative 4	National	both	6-9	6-9	3955	4025	3905	3944	
Mongolia	2005	STEPS	National	both	15-64	15-64	519	512	194	227	
Mongolia	2009	STEPS	National	both	15-64	15-64	932	661	228	180	
Mongolia	2013	Global School-based Student Health Survey	National	both	13-17	13-17	2360	2095	2360	2095	
Mongolia	2013	STEPS	National	both	15-64	15-64	1234	1124	471	492	
Mongolia	2019	STEPS	National	both	15-69	15-69	755	654	176	163	
Tajikistan	2003	Micronutrient Status Survey	National	both	15-49		1179		355		
Tajikistan	2012	DHS	National	both	15-49		5455		1923		
Tajikistan	2015-2016	Childhood Obesity Surveillance Initiative 4	National	both	7	7	1519	1502	1457	1438	
Tajikistan	2016	STEPS	National	both	18-69	18-69	394	310	38	38	
Tajikistan	2017	DHS	National	both	15-49		5714		1815		
Tajikistan	2019	Childhood Obesity Surveillance Initiative 5	National	both	7-8	7-8	1623	1809	1621	1809	
Turkmenistan	2000	DHS	National	both	15-49		1390				
Turkmenistan	2013	STEPS	National	both	18-64	18-64	767	559	121	51	
Turkmenistan	2015-2016	Childhood Obesity Surveillance Initiative 4	National	both	7-8	7-8	1976	1973	1956	1952	

Country	Study years	Survey/study name/citation	Level of representativeness	Rural, urban or both	Age range as in NCD-RisC database *		Sample size (height)		Sample size (BMI)		Note
					Female	Male	Female	Male	Female	Male	
Turkmenistan	2018	STEPS	National	both	18-69	18-69	523	429	65	29	
Uzbekistan	1996	DHS	National	both	15-49		2440		898		
Uzbekistan	2002	DHS	National	both	15-49	15-59	3008	1165	1104	418	
Uzbekistan	2014	STEPS	National	both	18-64	18-64	582	461	75	83	
Uzbekistan	2019	STEPS	National	both	18-69	18-69	258	172	25	10	
Middle East and north Africa											
Algeria	2003	STEPS	Subnational	both	25-64	25-64	478	256			
Algeria	2016-2017	STEPS	National	both	18-69	18-69	788	626	120	99	
Bahrain	1998-1999	National Nutrition Survey	National	both	19+	19+	203	212			
Bahrain	2001-2004	Global database on growth and malnutrition of school children and adolescents, WHO	National	both	6-20	6-19			1326	1268	
Bahrain	2016	Global School-based Student Health Survey	National	both	12-17	12-17	3262	3416	3262	3416	
Egypt	2000	DHS	National	both	20-49		5026				
Egypt	2002	National Survey of Smoking, Obesity, Blood Pressure and Blood Glucose	National	both	5+	5+	2836	2371	1793	1685	
Egypt	2003	DHS	National	both	20-49		3159				
Egypt	2003-2004	Marzouk et al., Gut 56(8):1105-10, 2007	Community	rural	25+	25+	66	39			
Egypt	2005	STEPS	National	both	15-65	15-65	2102	2148	659	812	
Egypt	2005	DHS	National	both	20-49		6725				
Egypt	2008	DHS	National	both	20-49	10-59	6570	11328		9694	
Egypt	2011	Global School-based Student Health Survey	National	both	13-17	13-17	454	325	454	325	
Egypt	2011	STEPS	National	both	15-65	15-65	965	533	222	185	
Egypt	2014	DHS	National	both	20-49		7717				
Egypt	2015	DHS	National	both	15-59	15-59	3988	3035	1330	1226	
Egypt	2017	STEPS	National	both	15-69	15-69	947	638	316	279	
Iran	1990-1991	National Health Survey I	National	both	5-18	5-18	9220	9083	9038	8883	
Iran	1999-2000	National Health Survey II	National	both	5+	5+	16933	14769	11480	11063	
Iran	1999-2001	Tehran Lipid and Glucose Study	Community	urban	5+	5+	3626	2878	2226	2033	
Iran	2001	Isfahan Healthy Heart Programme (IHHP), Arak	Community	both	19+	19+	987	1030	93	128	
Iran	2001	Isfahan Healthy Heart Programme (IHHP), Isfahan	Community	both	19+	19+	666	677	67	75	
Iran	2001	Isfahan Healthy Heart Programme (IHHP), Najaf Abad	Community	both	19+	19+	303	327	22	25	
Iran	2001	Isfahan Healthy Heart Programme (IHHP) Students, Arak	Community	both	11-18	11-18	495	467	495	467	
Iran	2001	Isfahan Healthy Heart Programme (IHHP) Students, Isfahan	Community	both	11-18	11-18	343	331	343	331	
Iran	2001	Isfahan Healthy Heart Programme (IHHP) Students, Najaf Abad	Community	both	11-18	11-18	146	123	146	123	
Iran	2003-2004	Childhood and Adolescence Surveillance and Prevention of Adult Noncommunicable Disease (CASPIAN)	National	both	6-18	6-18	10180	10805	10170	10791	
Iran	2002-2005	Tehran Lipid and Glucose Study	Community	urban	5+	5+	1385	1079	790	698	
Iran	2003-2004	The Persian Gulf Healthy Heart Study	Subnational	urban	25-75	25-75	350	326			
Iran	2005	Dastgiri et al., Public Health Nutr 9(8): 996-1000, 2006	Subnational	urban	15-70	15-70	66	51	21	14	
Iran	2005	STEPS	National	both	15-64	15-64	12022	12409	4069	4546	
Iran	2006	STEPS	National	both	16-65	16-65	4224	4437	1238	1470	
Iran	2007	Isfahan Healthy Heart Programme (IHHP), Arak	Community	both	19+	19+	756	894	79	121	
Iran	2007	Isfahan Healthy Heart Programme (IHHP), Isfahan	Community	both	19+	19+	482	553	22	38	
Iran	2007	Isfahan Healthy Heart Programme (IHHP), Najaf Abad	Community	both	19+	19+	294	259	18	15	
Iran	2007	Isfahan Healthy Heart Programme (IHHP) Students, Arak	Community	both	11-18	11-18	505	504	505	504	
Iran	2007	Isfahan Healthy Heart Programme (IHHP) Students, Isfahan	Community	both	11-18	11-18	354	412	354	412	
Iran	2007	Isfahan Healthy Heart Programme (IHHP) Students, Najaf Abad	Community	both	11-18	11-18	106	82	106	82	
Iran	2007	STEPS	National	both	15-64	15-64	701	743	195	255	
Iran	2007	STEPS	National	both	15-64	15-64	4358	4640	1347	1678	
Iran	2005-2008	Tehran Lipid and Glucose Study	Community	urban	5+	5+	1448	1201	701	660	
Iran	2008	STEPS	National	both	15-64	15-64	4384	4596	1366	1706	
Iran	2009	STEPS	National	both	15-64	15-64	4370	4717	1347	1594	
Iran	2009-2010	Childhood and Adolescence Surveillance and Prevention of Adult Noncommunicable Disease (CASPIAN)	National	both	10-18	10-18	2814	2799	2814	2799	
Iran	2008-2011	Tehran Lipid and Glucose Study	Community	urban	20+	20+	1318	999			
Iran	2011	STEPS	National	both	6-69	6-69	2507	2203	1027	1018	
Iran	2011-2012	Amol county study	Community	both	10+	10+	700	1017	266	434	
Iran	2011-2012	Childhood and Adolescence Surveillance and Prevention of Adult Noncommunicable Disease (CASPIAN)	National	both	6-18	6-18	6448	6658	6443	6648	
Iran	2012	National Integrated Micronutrient Survey (NIMS) 2012	National	both	6-60	6-60	6510	6066	5978	5649	
Iran	2012-2013	Tehran City	Community	urban	10-90	10-90	136	116	41	39	
Iran	2012-2013	Zahedan City	Community	urban	10-90	10-90	471	578	187	306	
Iran	2013-2014	Isfahan Salt Study (ISS)	Community	urban	6-18	6-18	383	400	383	400	
Iran	2014-2015	Childhood and Adolescence Surveillance and Prevention of Adult Noncommunicable Disease (CASPIAN)	National	both	7-18	7-18	6974	7177	6966	7164	
Iran	2015	Iranian School Measurement Database	National	both	6-18	6-18	912041	910541	911344	909863	
Iran	2016	STEPS	National	both	18+	18+	3098	2832	221	204	
Iran	2016-2017	Iranian Children and Adolescents Psychiatric Disorders (IRCAP) Survey	National	both	6-18	6-18	13493	13071	13476	13049	
Iran	2018-2019	Prevalence of risk factors for cardiovascular disease among a rural population in eastern Iran	Subnational	rural	18-69	18-69	20	32	2		
Iran	2016-2019	The Khuzestan comprehensive health study: A platform for NCDs, blood borne and mental diseases research	Subnational	both	20-65	20-65	3786	1910			
Iraq	2006	STEPS	National	both	25-64	25-64	948	1046			
Iraq	2013-2014	Qadir et al., Malays J Med Health Sci, 10(2):27-38, 2014	Community	urban	13-17	13-17	748	832	748	832	

Country	Study years	Survey/study name/citation	Level of representativeness	Rural, urban or both	Age range as in NCD-RisC database *		Sample size (height)		Sample size (BMI)		Note
					Female	Male	Female	Male	Female	Male	
Iraq	2015	STEPS	National	both	18+	18+	556	411	88	78	
Jordan	1997	DHS	National	both	20-49		1674				
Jordan	2002	DHS	National	both	20-49		1862				
Jordan	2004	Behavioural Risk Factor Surveillence Survey	National	rural	18+	18+	116	57	14	12	
Jordan	2007	Behavioural Risk Factor Surveillence Survey	National	both	18+	18+	85	74	17	10	
Jordan	2007	DHS	National	both	20-49		1601				
Jordan	2009	Metablic abnomalities and vitamin D study	National	both	7+	7+	1295	638	618	488	
Jordan	2009	DHS	National	both	20-49		1455				
Jordan	2012	DHS	National	both	20-49		2096				
Jordan	2015-2016	Zayed et al., BMC Public Health 16(1):1040, 2016	Subnational	both	6-17	6-15	1333	1075	1262	972	
Jordan	2016-2017	National Cardiovascular Diseases and Diabetes Study (NCDDS)	National	both	18+	18+	579	154	120	33	
Jordan	2017-2018	DHS	National	both	15-49		2096				
Jordan	2018-2019	Anthropometric Indices of Obesity as Predictors of High Blood Pressure among Schoolchildren	Community	urban	10-14	10-14	504	284	504	284	
Jordan	2019	STEPS	National	both	18-69	18-69	790	574	134	145	
Kuwait	1993-1994	al-Isa, Ann Nutr Metab 41(5):307-14, 1997	Community	both		18+				210	
Kuwait	2001	Kuwait Nutrition Surveillance System	National	urban	5-19	5-19	5391	4523	5389	4520	
Kuwait	2001	Kuwait Nutrition Surveillance System	National	urban	20+	20+	915	406			
Kuwait	2002	Kuwait Nutrition Surveillance System	National	urban	5-19	5-19	5239	5519	5239	5517	
Kuwait	2002	Kuwait Nutrition Surveillance System	National	urban	20+	20+	825	512			
Kuwait	2003	Kuwait Nutrition Surveillance System	National	both	5-19	5-19	5132	5179	5132	5179	
Kuwait	2003	Kuwait Nutrition Surveillance System	National	both	20+	20+	409	490			
Kuwait	2004	Kuwait Nutrition Surveillance System	National	both	5-19	5-19	4880	5021	4880	5021	
Kuwait	2004	Kuwait Nutrition Surveillance System	National	both	20+	20+	532	328			
Kuwait	2005	Kuwait Nutrition Surveillance System	National	both	5-19	5-19	5391	4523	5389	4520	
Kuwait	2005	Kuwait Nutrition Surveillance System	National	both	20+	20+	388	351			
Kuwait	2006	Kuwait Nutrition Surveillance System	National	both	5-19	5-19	5069	5261	5066	5259	
Kuwait	2006	Kuwait Nutrition Surveillance System	National	both	20+	20+	531	302			
Kuwait	2006	STEPS	National	both	20-64	20-64	309	222			
Kuwait	2007	Kuwait Nutrition Surveillance System	National	both	5-19	5-19	5504	5489	5502	5481	
Kuwait	2007	Kuwait Nutrition Surveillance System	National	both	20+	20+	403	405			
Kuwait	2008	Kuwait Nutrition Surveillance System	National	both	5-19	5-19	5596	5759	5595	5757	
Kuwait	2008	Kuwait Nutrition Surveillance System	National	both	20+	20+	492	416			
Kuwait	2009	Kuwait Nutrition Surveillance System	National	both	5-19	5-19	5576	5361	5574	5358	
Kuwait	2008-2009	National Nutrition Program for the State of Kuwait	National	both	5+	5+	402	414	294	314	
Kuwait	2008-2010	Gulf Cooperation Council World Health Survey	National	both	18+	18+	528	360	57	42	
Kuwait	2009	Kuwait Nutrition Surveillance System	National	both	20+	20+	456	341			
Kuwait	2011	Global School-based Student Health Survey	National	both	13-17	13-17	1274	1265	1274	1265	
Kuwait	2011-2014	Kuwait Diabetes Epidemiology Program	National	both	18-82	18-82	190	213	5	5	
Kuwait	2014	STEPS	National	both	18-69	18-69	692	506	47	62	
Kuwait	2015	Global School-based Student Health Survey	National	both	13-17	13-17	1553	1364	1553	1363	
Lebanon	1997	Obesity in Lebanon: National Survey	National	both	5+	5+	624	468	450	370	
Lebanon	2008-2009	STEPS	National	both	5+	5+	885	851	464	485	
Lebanon	2017	STEPS	National	both	18-69	18-69	123	98	11	4	
Libya	2009	STEPS	National	both	25-64	25-64	273	256			
Morocco	2003-2004	DHS	National	both	15-49		8904		3230		
Morocco	2017	STEPS	National	both	18+	18+	651	335	89	63	
Morocco	2019-2020	National Nutrition Survey	National	both	6-12	6-12	591	575	591	574	
Occupied Palestinian Territory	1996-1998	Ramallah study	Community	both	15-64	15-64	487		146		
Occupied Palestinian Territory	1999-2000	The First National Health and Nutrition Survey	National	both	18-64	18-64	451	399	62	47	
Occupied Palestinian Territory	2010	Global School-based Student Health Survey	National	both	13-17	13-17	1865	1823	1865	1822	
Occupied Palestinian Territory	2010	STEPS	National	both	15-64	15-64	1153	961	358	419	
Oman	2008	Gulf Cooperation Council World Health Survey	National	both	18+	18+	850	882	129	134	
Oman	2010	Global School-based Student Health Survey	National	both	13-17	13-17	300	251	300	251	
Oman	2015	Global School-based Student Health Survey	National	both	13-17	13-17	1551	1330	1551	1330	
Oman	2017	STEPS	National	both	15+	15+	813	886	169	165	
Qatar	2006	World Health Survey	National	both	18+	18+	540	399	60	67	
Qatar	2011	Global School-based Student Health Survey	National	both	13	13	126	102	126	102	
Qatar	2012	STEPS	National	both	18-64	18-64	399	296	88	70	
Saudi Arabia	1985-1988	National Nutrition Survey	National	both	5-75	5-75	2928	2717	542	273	1
Saudi Arabia	1989-1994	National Nutrition Survey	National	both	18-40	18-40			1905	1452	
Saudi Arabia	1992-1995	Saudi Health Information Survey	National	both	14-50	14-50			2484	2018	
Saudi Arabia	2005	STEPS	National	both	15-64	15-64	1115	982			
Saudi Arabia	2005	El Mouzan et al., Ann Saudi Med 30(3):203-208, 2010	National	both	5-18	5-18			9519	9853	
Saudi Arabia	2007	Gulf Cooperation Council World Health Survey	National	both	18+	18+	1287	1328	192	187	
Saudi Arabia	2009-2010	Arab Teens Lifestyle Study (ATLS)	Subnational	urban	14-19	14-19	1479	1384	1479	1384	
Saudi Arabia	2011-2013	Jeddah City Study	Community	urban	5+	5+	431	601	226	438	

Country	Study years	Survey/study name/citation	Level of representativeness	Rural, urban or both	Age range as in NCD-RisC database *		Sample size (height)		Sample size (BMI)		Note
					Female	Male	Female	Male	Female	Male	
Saudi Arabia	2011-2012	Jeeluna Study- National Assessment of the Health Needs of Adolescents in Saudi Arabia	National	both	12-19	12-19	5796	6245	5790	6234	
Saudi Arabia	2013	Saudi Health Information Survey	National	both	15+	15+	1937	1721	526	654	
Tunisia	1996-1997	Tunisian National Nutrition Survey 1996-1997	National	both	5+	5+	2227	1614	1606	1411	
Tunisia	2005	Aounallah et al., Public Health 12(1):98, 2012	National	both	15-19	15-19	1566	1290	1566	1290	
Tunisia	2009-2010	ObeMaghreb	Subnational	urban	5-49	5-49	1510	1148	1001	911	
Turkey	1998	DHS	National	both	20-49		1493				
Turkey	2003	Prevalence, awareness, treatment and control of hypertension in Turkey in 2003	National	both	18+	18+	784	526	129	102	
Turkey	2003	DHS	National	both	20-49		1858				
Turkey	2003-2005	Prevalence of prehypertension and associated risk factors among Turkish adults: Trabzon Hypertension Study	Subnational	both	20+	20+	699	607			
Turkey	2008	DHS	National	both	15-49		2095				
Turkey	2011	Chronic Diseases and Risk Factors Survey in Turkey	National	both	15+	15+	2542	2320	843	899	
Turkey	2009-2012	Prevalence of diabetes and associated risk factors among adult population in Trabzon city	Subnational	both	20+	20+	477	314			
Turkey	2013	Childhood Obesity Surveillance Initiative 3	National	both	7-8	7-8	2475	2483	2475	2483	
Turkey	2013	DHS	National	both	15-49		3937		1367		
Turkey	2015-2016	Childhood Obesity Surveillance Initiative 4	National	both	6-7	6-7	5336	5480	5336	5479	
Turkey	2016-2017	Erasmus plus KA2, Healthyland	Community	urban	6	6	22	29	22	29	
Turkey	2017	STEPS	National	both	15+	15+	612	469	151	142	
Turkey	2017-2018	Erasmus plus KA2, Healthyland	Community	urban	6	6	50	50	50	50	
United Arab Emirates	2005	Global School-based Student Health Survey	National	both	12-15	12-15	6268	5595	6268	5595	
United Arab Emirates	2009	Gulf Cooperation Council World Health Survey	National	both	18+	18+	182	148	24	31	
United Arab Emirates	2010	Global School-based Student Health Survey	National	both	13-17	13-17	1257	948	1257	948	
United Arab Emirates	2016	Global School-based Student Health Survey	National	both	12-17	12-17	2675	2323	2674	2323	
United Arab Emirates	2017-2018	STEPS	National	both	18+	18+	514	368	43	36	
Yemen	1997	DHS	National	both	15-49		1569				
Yemen	2005-2006	Yemen Household Budget Survey 2005-2006	National	both	5+	5+	2386	2451	1733	1914	
Yemen	2007-2009	Hypertension and Diabetes in Yemen (HYDY)	National	both	6-70	6-70	2705	2632	1546	1558	
Yemen	2013	DHS	National	both	15-49		15621		5930		
**East and southeast Asia**											
East Asia											
China	1985	Chinese National Surveys on Students Constitution and Health	National	both	7-18	7-18	136880	136878			
China	1989	China Health and Nutrition Study	National	both	5-45	5-45	167	200	166	197	2
China	1991	China Health and Nutrition Study	National	both	5+	5+	1684	1746	1661	1730	2
China	1992	China National Nutrition Survey	National	both	5+	5+	10379	11005	10380	11004	
China	1993	China Health and Nutrition Study	National	both	5+	5+	1523	1699	1500	1677	2
China	1995	Chinese National Surveys on Students Constitution and Health	National	both	7-18	7-18	101772	103009	101772	103009	
China	1997	China Health and Nutrition Study	National	both	5+	5+	2223	2418	1366	1532	2
China	2000	China Health and Nutrition Study	National	both	5+	5+	2041	2192	1277	1452	2
China	2000	Chinese National Surveys on Students Constitution and Health	National	both	7-18	7-18	108097	107997	108097	107997	
China	1996-2003	Wu et al., Osteoporos Int 15:751-59, 2004	Community	urban	18+				114		
China	2002	China National Nutrition and Health Survey	National	both	5+	5+	28161	29026	18713	20739	
China	2004	Beijing Child and Adolescent Metabolic Syndrome study	Community	both	5-18	5-18	10413	10564	10412	10562	
China	2004	China Health and Nutrition Study	National	both	5+	5+	1331	1487	828	968	2
China	2005	Chinese National Surveys on Students Constitution and Health	National	both	7-18	7-18	116705	117598	116705	117598	
China	2004-2005	Xinjiang Children and Adolescent Survey	Community	urban	6-18	6-18	2240	2035	2233	2030	
China	2006	China Health and Nutrition Study	National	both	5+	5+	1096	1189	676	787	2
China	2005-2006	Hong Kong Growth Survey	Community	urban	7-19	7-19	7370	7472	7370	7472	
China	2007	Beijing Child and Adolescent Metabolic Syndrome study	Community	urban	7-18	7-18	664	863	664	863	
China	2009	The 33 Chinese Communities Health Study (33CCHS)	Subnational	urban	18-74	18-74	903	1612	47	88	
China	2009	China Health and Nutrition Study	National	both	5+	5+	1080	1170	602	740	2
China	2009	The nutrition-based comprehensive intervention study on childhood obesity in China	Subnational	urban	6-11	6-11	4269	4495	4269	4495	
China	2010	China Chronic Disease and Risk Factors Surveillance (CCDRFS)	National	both	18+	18+	7443	7204	1254	1229	
China	2009-2010	China National Survey of Chronic Kidney Disease	National	both	18+	18+	2717	2651	92	123	
China	2010	Chinese National Surveys on Students Constitution and Health	National	both	7-18	7-18	107612	107611	107612	107611	
China	2009-2011	The FAMILY Cohort	Community	urban	15+	15+	2010	1984	780	897	
China	2011	Beijing Childhood Eye Study	Community	both	7-18	7-18	7301	6886	6967	6686	
China	2011	Beijing Children Eye Study	Community	both	5-13	5-13	275	304	261	289	
China	2011	China Health and Nutrition Study	National	both	5+	5+	1506	1457	881	943	2
China	2012	Beijing Children Eye Study	Community	both	5-13	5-13	255	283	249	282	
China	2012	Beijing Eye High School Students Study	Community	both	16-18	16-18	2407	2103	2340	2088	
China	2011-2013	International Study of Childhood Obesity, Lifestyle and the Environment (ISCOLE)	Community	urban	9-11	9-11	259	293	258	293	
China	2012	Shandong Children Study	Community	both	5-18	5-18	2766	3086	2766	3086	
China	2010-2014	National Free Preconception Health Examination Project	National	rural		20-64		10576140			
China	2013	Gobi Desert Children Eye Study	Community	urban	6-21	6-21	761	800	761	797	
China	2011-2014	The FAMILY Cohort	Community	urban	15+	15+	1048	1029	325	350	
China	2012-2013	The Kailuan Study	Community	urban	18+	18+	1260	6475		39	
China	2012-2013	The Seven Northeastern Cities (SNEC) Study	Subnational	urban	5-17	5-17	4579	4770	4577	4769	

Country	Study years	Survey/study name/citation	Level of representativeness	Rural, urban or both	Age range as in NCD-RisC database *		Sample size (height)		Sample size (BMI)		Note
					Female	Male	Female	Male	Female	Male	
China	2014	Chinese National Surveys on Students Constitution and Health	National	both	7-18	7-18	107138	107216	107138	107216	
China	2014	Shanghai Municipal Surveys on Students Constitution and Health	Community	both	7-18	7-18	7665	7758	7665	7758	
China	2015	China Health and Nutrition Study	National	both	5+	5+	966	1149	608	665	2
China	2014-2015	The Kailuan Study	Community	urban	18+	18+	880	6170		2	
China	2016	Greater Beijing School Children Myopia Study	Subnational	both	6-18	6-18	16516	17259	16516	17257	
China	2016-2018	Smart device usage, lifestyles behaviors, physical fitness, and eye problems: A prospective study in Hong Kong adolescents	Community	urban	7-15	7-15	741	703	741	703	
Taiwan	1993-1996	Nutrition and Health Survey in Taiwan	National	both	5+	5+	1614	1543	1610	1536	
Taiwan	2001-2002	Nutrition and Health Survey in Taiwan	National	both	6-12	6-12	1139	1334	1139	1334	
Taiwan	2005-2008	Nutrition and Health Survey in Taiwan	National	both	19+	19+	174	151	7	13	
Taiwan	2007	Taiwanese Survey on Hypertension, Hyperglycemia and Hyperlipidemia	National	both	20+	20+	381	368			
Taiwan	2010	Nutrition and Health Survey in Taiwan	National	both	13-15	13-15	927	852	927	852	
Taiwan	2011	Nutrition and Health Survey in Taiwan	National	both	16-18	16-18	591	580	591	580	
Taiwan	2012	Global School-based Student Health Survey	National	both	13-17	13-17	2927	2998	2927	2998	
Taiwan	2012	Nutrition and Health Survey in Taiwan	National	both	7-12	7-12	499	510	499	510	
Taiwan	2013-2016	Nutrition and Health Survey in Taiwan	National	both	5+	5+	1311	1226	1105	1056	
Taiwan	2019	Global School-based Student Health Survey	National	both	15-17	15-17	1854	1701	1854	1701	
Southeast Asia											
Brunei Darussalam	2010-2011	National Health And Nutritional Status Survey (NHANSS)	National	both	5-75	5-75	578	578	349	347	
Brunei Darussalam	2014	Global School-based Student Health Survey	National	both	12-17	12-17	1326	1145	1326	1145	
Brunei Darussalam	2015-2016	National Non-Communicable Diseases Survey (NNCDS)	National	both	18-69	18-69	283	221	48	51	
Brunei Darussalam	2019	Global School-based Student Health Survey	National	both	12-17	12-17	1153	1061	1152	1061	
Cambodia	2000	DHS	National	both	15-49		3676		1620		
Cambodia	2005	DHS	National	both	15-49		4462		1765		
Cambodia	2010	DHS	National	both	15-49		5157		1894		
Cambodia	2010	STEPS	National	both	25-64	25-64	504	303			
Cambodia	2014	DHS	National	both	15-49		5901		1842		
Indonesia	1993-1994	Indonesian Family Life Surveys	National	both	5+	5+	3308	3224	3275	3216	
Indonesia	1997-1998	Indonesian Family Life Surveys	National	both	5+	5+	7312	6734	5006	4960	
Indonesia	2000-2001	Indonesian Family Life Surveys	National	both	5+	5+	9134	8637	5561	5580	
Indonesia	2003	A genetic-ecological study of the risk foctors for lifestyle-related diseases in Oceanian populations, Study A	Community	rural	18-79	18-79	44	23	13	3	
Indonesia	2003	A genetic-ecological study of the risk foctors for lifestyle-related diseases in Oceanian populations, Study B	Community	rural	18-79	18-79	58	45	12	13	
Indonesia	2007-2008	Indonesian Family Life Surveys	National	both	5+	5+	10037	9395	5630	5779	
Indonesia	2011	SEANUTS	National	both	5-12	5-12	1380	1363	1380	1363	
Indonesia	2013	Population Health Basic Health Research 2013 (Riskesdas 2013)	National	both	5-19	5-19	146291	155850	145926	155332	
Indonesia	2013	Population Health Basic Health Research 2013 (Riskesdas 2013)	National	both	20+	20+	66157	60693			
Indonesia	2015	Global School-based Student Health Survey	National	both	13-17	13-17	4688	3845	4688	3845	
Indonesia	2014-2015	Indonesian Family Life Surveys	National	both	5+	5+	10772	10008	6639	6814	
Indonesia	2018	Indonesian Basic Health Survey 2018	National	both	15+	15+	2815	1611	837	641	
Lao PDR	2006	Multiple Indicator Cluster Survey 3	National	both	15-49		409		164		
Lao PDR	2008	STEPS	Community	both	25-64	25-64	330	173			
Lao PDR	2013	STEPS	National	both	18-64	18-64	430	228	53	38	
Malaysia	1996	National Health and Morbidity Survey (NHMS)	National	both	18+	18+	5419	4451	927	730	
Malaysia	2002-2003	Malaysian Adult Nutrition Survey	National	both	18-59	18-59	418	501	62	73	
Malaysia	2004	Rampal et al., Public Health 122(1):11-8, 2008	National	both	15+	15+	2981	2230	1229	976	
Malaysia	2005	STEPS	National	both	25-64	25-64	443	438			
Malaysia	2006	National Health and Morbidity Survey (NHMS)	National	both	5+	5+	12391	12090	8540	8767	
Malaysia	2008	National Iodine Deficiency Disorder (IDD) Survey	National	both	7-10	7-10	8660	9391	8659	9388	
Malaysia	2008	Metabolic Syndrome Study in Malaysia	National	both	18+	18+	317	214	54	31	
Malaysia	2011	National Health and Morbidity Survey (NHMS)	National	both	5+	5+	2253	2342	364	389	
Malaysia	2010-2011	SEANUTS	National	both	5-12	5-12	1352	1306	1352	1306	
Malaysia	2012	Malaysian School-Based Health Survey	National	both	9-17	9-17	19625	20224	19616	20214	
Malaysia	2012-2013	Petaling District	Subnational	urban	12-17	12-17	1364	882	1364	882	
Malaysia	2013-2014	Batang Padang District	Subnational	both	12-17	12-17	3319	2928	3319	2928	
Malaysia	2014	Malaysian Adult Nutrition Survey	National	both	18-59	18-59	377	391	44	79	
Malaysia	2015	National Health and Morbidity Survey (NHMS)	National	both	5+	5+	5483	5556	3660	3628	
Malaysia	2019	National Health and Morbidity Survey	National	both	5+	5+	2639	2680	1744	1765	
Maldives	2001	Multiple Indicator Cluster Survey	National	both	15-50		578				
Maldives	2004	STEPS	Subnational	urban	25-64	25-64	311	292			
Maldives	2009	Global School-based Student Health Survey	National	both		13-17		807		806	
Maldives	2009	DHS	National	both	20-49		2299				
Maldives	2011	STEPS	Subnational	urban	15-64	15-64	395	312	113	131	
Maldives	2014	Global School-based Student Health Survey	National	both		13-17		931		931	
Maldives	2016-2017	DHS	National	both	15-49	15-49	3298	2032	934	851	
Myanmar	2003-2004	STEPS	Subnational	both	25-74	25-74	186	156			
Myanmar	2009	STEPS	National	both	15-64	15-64	1025	720	262	176	
Myanmar	2011	Underweight prevalence among young adults from rural areas, Salin Township, Magwe Region	Community	rural	15-35	15-35	183	140	60	51	

Country	Study years	Survey/study name/citation	Level of representativeness	Rural, urban or both	Age range as in NCD-RisC database *		Sample size (height)		Sample size (BMI)		Note
					Female	Male	Female	Male	Female	Male	
Myanmar	2014	STEPS	National	both	25-64	25-64	492	314			
Myanmar	2013-2014	STEPS, Yangon	Subnational	both	25-74	25-74	67	60			
Myanmar	2015-2016	DHS	National	both	15-49		5510		1777		
Philippines	1991-1992	Cebu Longitudinal Health and Nutrition Survey 1991 Child Follow-up	Community	both	8	8	1076	1202	1076	1202	
Philippines	1994-1995	Cebu Longitudinal Health and Nutrition Survey 1994-1995 Mother Follow-up	Community	both	15-59		250		244		
Philippines	1998-1999	Cebu Longitudinal Health and Nutrition Survey 1998-1999 Child Follow-up	Community	both	14-16	14-16	994	1099	994	1099	
Philippines	1998-1999	Cebu Longitudinal Health and Nutrition Survey 1998-1999 Mother Follow-up	Community	both	15-59		3				
Philippines	2002	Cebu Longitudinal Health and Nutrition Survey 2002 Child Follow-up	Community	both	17-19	17-19	961	1087	907	1087	
Philippines	2003	Global School-based Student Health Survey	National	both	13		291		291		
Philippines	2003	6th National Nutrition Survey	National	both	5+	5+	6578	6323	4350	4494	
Philippines	2005	Cebu Longitudinal Health and Nutrition Survey 2005 Child Follow-up	Community	both	20-22	20-22	903	1006			
Philippines	2007	Global School-based Student Health Survey	National	both	13		254		254		
Philippines	2007	Cebu Longitudinal Health and Nutrition Survey 2007 Child Follow-up	Community	both	23-24	23-24	816	937			
Philippines	2008	7th National Nutrition Survey	National	both	5+	5+	33476	37432	26584	28859	
Philippines	2009	Cebu Longitudinal Health and Nutrition Survey 2009 Child Follow-up	Community	both	24-26	24-26	796	864			
Philippines	2011	Global School-based Student Health Survey	National	both	13		540		540		
Philippines	2011	2011 Updating of Nutritional Status of Filipino Children	National	both	5+	5+	35989	37670	26983	29544	
Philippines	2013-2014	8th National Nutrition Survey	National	both	5+	5+	30662	31628	22865	24303	
Philippines	2015	Global School-based Student Health Survey	National	both	13-17		3730		3730		
Philippines	2015	2015 Updating of Nutritional Status of Filipino Children and Other Population Groups	National	both	5+	5+	37206	38158	27178	28876	
Philippines	2019	Global School-based Student Health Survey	National	both	12-16	12-16	4290	3573	4286	3571	
Thailand	1991	Thailand National Health Examination Survey I	National	both	5+	5+	3236	3202	3228	3197	
Thailand	1997	Thailand National Health Examination Survey II	National	both	5-59	5-59	3451	3276	2944	2987	
Thailand	2004	Thailand National Health Examination Survey III	National	both	15+	15+	1827	2144	502	702	
Thailand	2003-2004	The Fifth National Nutrition Survey of Thailand	National	both	19+	19+	635	265			
Thailand	2009	Thailand National Health Examination Survey IV	National	both	5+	5+	945	925	3842	3874	
Thailand	2011	SEANUTS	National	both	5-12	5-12	939	922	939	922	
Thailand	2014	Thailand National Health Examination Survey V	National	both	10+	10+	2631	2356	1811	1744	
Thailand	2015	Global School-based Student Health Survey	National	both	12-17	12-17	3106	2240	3106	2240	
Thailand	2019-2020	Thailand National Health Examination Survey VI	National	both	10+	10+	3524	3129	2103	2098	
Timor-Leste	2009-2010	DHS	National	both	15-49		7262		3065		
Timor-Leste	2013	Child measurements in Ossu and in Natarbora, Timor Leste	Subnational	rural	5-19	5-19	239	245	239	245	
Timor-Leste	2014	STEPS	National	both	18-69	18-69	404	220	70	29	
Timor-Leste	2016	DHS	National	both	15-49	15-59	7010	2199	3019	1037	
Timor-Leste	2018	Child measurements in Ossu and in Natarbora, Timor Leste	Subnational	rural	5-19	5-19	246	234	246	234	
Timor-Leste	2018	Child measurements in Ossu and in Natarbora, Timor Leste	Subnational	rural	5-19	5-19	133	99	132	97	
Viet Nam	1987-1989	General Nutrition Survey	National	both	15-70	15-70			2303	2236	1
Viet Nam	1992-1993	Living Standard Survey	National	both	5+	5+	4128	4172	4107	4172	
Viet Nam	1997-1998	Living Standard Survey	National	both	5+	5+	6866	6954	4908	5059	
Viet Nam	2000	National Nutrition Survey	National	both	20+	20+	2347	2455			
Viet Nam	2001-2002	Viet Nam National Health Survey 2001-2002	National	both	5+	5+	36661	36636	25748	27137	
Viet Nam	2001-2003	The National Epidemiological Survey on Hypertension and Its Risk Factors (North)	Subnational	both	25-74	25-74	427	265			
Viet Nam	2003-2004	The Survey on Heart Failure and Its Risk Factors	Subnational	both	25-74	25-74	291	195			
Viet Nam	2004	The Hypertension Management Programme in Rural Communes (Hanoi)	Community	rural	25-74	25-74	85	61			
Viet Nam	2005	National Adult Overweight Survey	National	both	25-64	25-64	1039	1038			
Viet Nam	2005	The Survey on Non-Communicable Disease Risk Factors	Subnational	both	25-74	25-74	104	105			
Viet Nam	2005	Non-communicable disease risk factors in Ho Chi Minh City	Community	urban	25-64	25-64	101	110			
Viet Nam	2006	Qualitative and quantitative assessment of nutritional status and lifestyles of Vietnamese adolescents	Subnational	both	15-19	15-19	697	506	697	506	
Viet Nam	2006	The Hypertension Management Programme in Rural Communes (Bavi)	Community	rural	25-74	25-74	45	17			
Viet Nam	2007	Global School-based Student Health Survey	National	both	12-15	12-15	996	697	995	697	
Viet Nam	2007	The Hypertension Management Programme in Rural Communes (Phu Phuong)	Community	rural	25-74	25-74	52	15			
Viet Nam	2006-2008	The National Epidemiological Survey on Hypertension and Its Risk Factors (South)	Subnational	both	25-74	25-74	177	115			
Viet Nam	2008-2009	The Survey on Diabetes and Its Risk Factors	Subnational	both	25+	25+	83	44			
Viet Nam	2009	The Hypertension Management Programme in Rural Communes (Phu Cuong)	Community	rural	25-74	25-74	42	9			
Viet Nam	2009	STEPS	National	both	25-64	25-64	830	634			
Viet Nam	2009-2010	Vietnam National Nutrition Survey 2009-2010	National	both	5+	5+	8444	8067	5160	5210	
Viet Nam	2011	SEANUTS	National	both	5-11	5-11	980	975	980	975	
Viet Nam	2013	Global School-based Student Health Survey	National	both	13-17	13-17	1578	1368	1578	1368	
Viet Nam	2015	STEPS	National	both	18-69	18-69	251	220	19	26	
Viet Nam	2019	Global School-based Student Health Survey	National	both	13-18	13-18	4118	3572	4117	3572	
**High-income Asia Pacific**											
Japan	1976	National Nutrition Survey	National	both	5+	5+	228	204			
Japan	1977	National Nutrition Survey	National	both	5+	5+	326	358			
Japan	1978	National Nutrition Survey	National	both	5+	5+	570	608			
Japan	1979	National Nutrition Survey	National	both	5+	5+	770	784			
Japan	1980	National Cardiovascular Survey	National	both	5+	5+	872	938			

Country	Study years	Survey/study name/citation	Level of representativeness	Rural, urban or both	Age range as in NCD-RisC database *		Sample size (height)		Sample size (BMI)		Note
					Female	Male	Female	Male	Female	Male	
Japan	1981	National Nutrition Survey	National	both	5+	5+	976	956			
Japan	1982	National Nutrition Survey	National	both	5+	5+	1235	1264			
Japan	1983	National Nutrition Survey	National	both	5+	5+	1333	1398			
Japan	1984	National Nutrition Survey	National	both	5+	5+	1314	1346			
Japan	1985	National Nutrition Survey	National	both	5+	5+	1551	1659			
Japan	1986	National Nutrition Survey	National	both	5+	5+	1688	1765			
Japan	1987	National Nutrition Survey	National	both	5+	5+	1573	1602	1845	1863	1
Japan	1988	National Nutrition Survey	National	both	5+	5+	1650	1759	1838	1912	1
Japan	1989	National Nutrition Survey	National	both	5+	5+	1380	1534	1453	1607	1
Japan	1990	National Nutrition Survey and National Cardiovascular Survey	National	both	5+	5+	1527	1657	1526	1655	
Japan	1991	National Nutrition Survey	National	both	5+	5+	1451	1548	1450	1546	
Japan	1992	National Nutrition Survey	National	both	5+	5+	1340	1429	1337	1425	
Japan	1993	Iwata kids health study	Community	urban	10	10	485	513	485	513	
Japan	1993	National Nutrition Survey	National	both	5+	5+	1399	1451	1398	1450	
Japan	1994	Iwata kids health study	Community	urban	10	10	567	569	567	569	
Japan	1994	National Nutrition Survey	National	both	5+	5+	1236	1285	1235	1281	
Japan	1995	Iwata kids health study	Community	urban	10	10	567	524	567	524	
Japan	1995	National Nutrition Survey	National	both	5+	5+	1213	1311	1211	1307	
Japan	1996	Iwata kids health study	Community	urban	10	10	480	552	480	552	
Japan	1996	National Nutrition Survey	National	both	5+	5+	1871	1739	1090	1104	
Japan	1997	Iwata kids health study	Community	urban	10	10	537	506	537	506	
Japan	1997	National Nutrition Survey	National	both	5+	5+	1809	1669	1023	1065	
Japan	1998	Iwata kids health study	Community	urban	10	10	464	527	464	527	
Japan	1998	National Nutrition Survey	National	both	5+	5+	1740	1684	1043	1090	
Japan	1999	Iwata kids health study	Community	urban	10	10	463	468	463	468	
Japan	1999	National Nutrition Survey	National	both	5+	5+	1498	1321	816	835	
Japan	2000	Iwata kids health study	Community	urban	10	10	401	440	401	440	
Japan	2000	National Nutrition Survey and National Cardiovascular Survey	National	both	5+	5+	1480	1422	925	902	
Japan	2001	Iwata kids health study	Community	urban	10	10	414	452	414	452	
Japan	2001	National Nutrition Survey	National	both	5+	5+	1417	1331	862	913	
Japan	2001	The Japan Association of Health Service Database	Subnational	both	20+	20+	186355	247264			
Japan	2002	Iwata kids health study	Community	urban	10	10	398	496	398	496	
Japan	2002	National Nutrition Survey	National	both	5+	5+	1194	1101	736	719	
Japan	2003	Iwata kids health study	Community	urban	10	10	399	415	399	415	
Japan	2003	National Health and Nutrition Survey	National	both	5+	5+	1157	1061	703	674	
Japan	2004	Iwata kids health study	Community	urban	10	10	412	463	412	463	
Japan	2004	National Health and Nutrition Survey	National	both	5+	5+	912	941	545	642	
Japan	2005	Iwata kids health study	Community	urban	10	10	420	476	420	476	
Japan	2005	National Health and Nutrition Survey	National	both	5+	5+	808	754	512	480	
Japan	2006	Iwata kids health study	Community	urban	10	10	391	417	391	417	
Japan	2006	National Health and Nutrition Survey	National	both	5+	5+	918	891	584	615	
Japan	2007	Fukuroi kids health study	Community	urban	13-14	13-14	372	395	372	395	
Japan	2007	Iwata kids health study	Community	urban	10	10	394	439	394	439	
Japan	2007	National Health and Nutrition Survey	National	both	5+	5+	902	831	594	589	
Japan	2008	Fukuroi kids health study	Community	urban	13-14	13-14	346	381	346	381	
Japan	2008	Iwata kids health study	Community	urban	10	10	417	406	417	406	
Japan	2008	MEXT School Health Statistics	National	both	5-17	5-17	326966	326407	326957	326405	
Japan	2008	National Health and Nutrition Survey	National	both	5+	5+	784	767	488	504	
Japan	2009	Fukuroi kids health study	Community	urban	13-14	13-14	357	388	357	388	
Japan	2009	MEXT School Health Statistics	National	both	5-17	5-17	327108	326529	327098	326525	
Japan	2009	National Health and Nutrition Survey	National	both	5+	5+	871	809	574	563	
Japan	2010	Fukuroi kids health study	Community	urban	13-14	13-14	387	360	387	360	
Japan	2010	MEXT School Health Statistics	National	both	5-17	5-17	326414	326517	326401	326509	
Japan	2010	National Health and Nutrition Survey	National	both	5+	5+	719	705	458	486	
Japan	2011	Fukuroi kids health study	Community	urban	13-14	13-14	369	402	369	402	
Japan	2011	MEXT School Health Statistics	National	both	5-17	5-17	306389	305274	306383	305270	
Japan	2011	National Health and Nutrition Survey	National	both	5+	5+	723	642	457	435	
Japan	2011	The Tokyo Health Service Association Database	Community	urban	20+	20+	17931	15068			
Japan	2012	Fukuroi kids health study	Community	urban	13-14	13-14	353	432	353	432	
Japan	2012	MEXT School Health Statistics	National	both	5-17	5-17	326577	326527	326572	326524	
Japan	2012	National Health and Nutrition Survey	National	both	5+	5+	2485	2380	1602	1610	
Japan	2013	Awaji Child Health Study	Community	urban	10-14	10-14	203	198	203	198	
Japan	2013	Fukuroi kids health study	Community	urban	13-14	13-14	404	387	404	387	
Japan	2013	MEXT School Health Statistics	National	both	5-17	5-17	327581	327926	327578	327923	
Japan	2013	National Health and Nutrition Survey	National	both	5+	5+	695	719	438	458	
Japan	2014	Awaji Child Health Study	Community	urban	10-14	10-14	218	229	218	229	

Country	Study years	Survey/study name/citation	Level of representativeness	Rural, urban or both	Age range as in NCD-RisC database *		Sample size (height)		Sample size (BMI)		Note
					Female	Male	Female	Male	Female	Male	
Japan	2014	MEXT School Health Statistics	National	both	5-17	5-17	326885	327062	326884	327062	
Japan	2014	National Health and Nutrition Survey	National	both	5+	5+	677	640	440	460	
Japan	2014-2015	Nagaoka Health Screening	Community	both	20-89	20-89	473	294			
Japan	2015	Awaji Child Health Study	Community	urban	10-14	10-14	228	230	228	230	
Japan	2015	MEXT School Health Statistics	National	both	5-17	5-17	327212	326383	327210	326382	
Japan	2015	National Health and Nutrition Survey	National	both	5+	5+	631	609	417	434	
Japan	2016	MEXT School Health Statistics	National	both	5-17	5-17	334447	334735	334444	334734	
Japan	2016	National Health and Nutrition Survey	National	both	5+	5+	1926	1929	1269	1346	
Japan	2017	MEXT School Health Statistics	National	both	5-17	5-17	333725	333185	333723	333184	
Japan	2017	National Health and Nutrition Survey	National	both	5+	5+	513	494	345	327	
Japan	2017	The Tokyo Health Service Association Database	Community	urban	20+	20+	14319	11117			
Japan	2018	MEXT School Health Statistics	National	both	5-17	5-17	336954	337022	336953	337022	
Japan	2018	National Health and Nutrition Survey	National	both	5+	5+	558	562	369	388	
Japan	2019	MEXT School Health Statistics	National	both	5-17	5-17	333546	332232	333546	332231	
Japan	2019	National Health and Nutrition Survey	National	both	5+	5+	429	453	286	317	
Japan	2020	MEXT School Health Statistics	National	both	5-17	5-17	333858	333405	333857	333405	
Singapore	2004-2007	Singapore Cardiovascular Cohort Study and Singapore Prospective Study Program	National	both	24+	24+	126	90			
Singapore	2012-2013	Singapore Health Study 2012	National	both	18-79	18-79	218	224	44	36	
Singapore	2014-2015	Singapore Health 2	National	both	18-80	18-80	138	115	21	15	
South Korea	1984	Kim et al., Am J Phys Anthropol 136:230-6, 2008	National	both	5-20	5-20	26029	27035			
South Korea	1998	Korea National Health and Nutrition Examination Survey	National	both	10+	10+	1726	1576	891	917	
South Korea	1997-1998	National Anthropometric Survey in Korean Children and Adolescents	National	both	5-19	5-19	26469	29318	26469	29318	
South Korea	1999	The South Korean Consciption Database	National	both		19		401721		401721	
South Korea	2000	The South Korean Consciption Database	National	both		19		402758		402758	
South Korea	2001	The South Korean Consciption Database	National	both		19		398653		398653	
South Korea	2001	Korea National Health and Nutrition Examination Survey	National	both	5+	5+	1796	1751	1163	1286	
South Korea	2002	The South Korean Consciption Database	National	both		19		367024		367024	
South Korea	2003	The South Korean Consciption Database	National	both		19		329626		329626	
South Korea	2004	The South Korean Consciption Database	National	both		19		323001		323001	
South Korea	2005	The South Korean Consciption Database	National	both		19		313378		313378	
South Korea	2005	Korea National Health and Nutrition Examination Survey	National	both	5+	5+	1202	1131	800	858	
South Korea	2005	National Anthropometric Survey in Korean Children and Adolescents	National	both	5-19	5-19	39197	41723	39200	41727	
South Korea	2006	The South Korean Consciption Database	National	both		19		302587		302587	
South Korea	2007	The South Korean Consciption Database	National	both		19		312795		312795	
South Korea	2007	Korea National Health and Nutrition Examination Survey	National	both	5+	5+	642	633	460	496	
South Korea	2008	The South Korean Consciption Database	National	both		19		312919		312919	
South Korea	2008	Korea National Health and Nutrition Examination Survey	National	both	5+	5+	1450	1386	956	1056	
South Korea	2009	The South Korean Consciption Database	National	both		19		324818		324818	
South Korea	2009	Korea National Health and Nutrition Examination Survey	National	both	5+	5+	1531	1524	1008	1083	
South Korea	2009	Korea National School Health Examination Survey (KNSHES)	National	both	6-20	6-20	89897	104042	89881	103997	
South Korea	2010	The South Korean Consciption Database	National	both		19		347249		347249	
South Korea	2007-2012	JS High-School Study	Community	rural	14-17	14-17	508	553	508	553	
South Korea	2010	Korea National Health and Nutrition Examination Survey	National	both	5+	5+	1226	1178	802	897	
South Korea	2010	Korea National School Health Examination Survey (KNSHES)	National	both	6-20	6-20	86244	99965	86226	99944	
South Korea	2011	The South Korean Consciption Database	National	both		19		364982		364982	
South Korea	2011	Korea National Health and Nutrition Examination Survey	National	both	5+	5+	1063	1021	700	751	
South Korea	2011	Korea National School Health Examination Survey (KNSHES)	National	both	6-20	6-20	83059	97377	83046	97363	
South Korea	2012	The South Korean Consciption Database	National	both		19		361009		361009	
South Korea	2012	Korea National Health and Nutrition Examination Survey	National	both	5+	5+	982	960	619	721	
South Korea	2012	Korea National School Health Examination Survey (KNSHES)	National	both	6-20	6-20	42009	45075	42005	45066	
South Korea	2013	The South Korean Consciption Database	National	both		19		363914		363914	
South Korea	2013	Korea National Health and Nutrition Examination Survey	National	both	5+	5+	1071	1051	702	770	
South Korea	2013	Korea National School Health Examination Survey (KNSHES)	National	both	6-20	6-20	40791	43685	40785	43675	
South Korea	2014	The South Korean Consciption Database	National	both		19		363597		363597	
South Korea	2014	Korea National Health and Nutrition Examination Survey	National	both	5+	5+	895	845	554	621	
South Korea	2014	Korea National School Health Examination Survey (KNSHES)	National	both	6-20	6-20	39991	42580	39987	42570	
South Korea	2015	The South Korean Consciption Database	National	both		19		350518		350518	
South Korea	2015	Korea National Health and Nutrition Examination Survey	National	both	5+	5+	853	909	539	612	
South Korea	2015	Korea National School Health Examination Survey (KNSHES)	National	both	6-20	6-20	41654	43152	41645	43141	
South Korea	2016	The South Korean Consciption Database	National	both		19		339410		339410	
South Korea	2016	Korea National Health and Nutrition Examination Survey	National	both	5+	5+	997	951	635	675	
South Korea	2016	Korea National School Health Examination Survey (KNSHES)	National	both	6-20	6-20	40635	42245	40631	42242	
South Korea	2017	The South Korean Consciption Database	National	both		19		323457		323457	
South Korea	2017	Korea National Health and Nutrition Examination Survey	National	both	5+	5+	963	941	608	623	
South Korea	2018	Korea National Health and Nutrition Examination Survey	National	both	5+	5+	938	915	562	590	
South Korea	2019	Korea National Health and Nutrition Examination Survey	National	both	5+	5+	905	984	576	622	

Country	Study years	Survey/study name/citation	Level of representativeness	Rural, urban or both	Age range as in NCD-RisC database *		Sample size (height)		Sample size (BMI)		Note
					Female	Male	Female	Male	Female	Male	
South Korea	2020	Korea National Health and Nutrition Examination Survey	National	both	5+	5+	815	924	449	545	
**High-income western**											
High-income English-speaking countries											
Australia	1985	Austrailian Council for Health, Physical Education and Recreation survey	National	both	7-12	7-12	702	707			
Australia	1995	National Nutrition Survey 1995	National	both	5+	5+	1175	1237	1171	1236	
Australia	1996	The Nepean Longitudinal Cohort Study	Community	urban	7-8	7-8	215	221	215	221	
Australia	1999-2000	The Australian Diabetes, Obesity and Lifestyle Study 1999-2000	National	both	25+	25+	319	263			
Australia	1999-2003	North West Adelaide Health Study	Community	urban	18+	18+	225	236	37	31	
Australia	2004	The Longitudinal Study of Australian Children, K cohort (child)	National	both	5	5	429	424	429	424	
Australia	2004	The Nepean Longitudinal Cohort Study	Community	urban	14-15	14-15	150	143	149	143	
Australia	2004-2006	North West Adelaide Health Study	Community	urban	20+	20+	62	77			
Australia	2006	The Longitudinal Study of Australian Children, K cohort (child)	National	both	6-7	6-7	2160	2246	2152	2242	
Australia	2007	Children's Nutrition and Physical Activity Survey	National	both	5-16	5-16	1652	1626	1649	1623	
Australia	2008	The Longitudinal Study of Australian Children, B cohort (infant)	National	both	5	5	512	508	511	508	
Australia	2008	The Longitudinal Study of Australian Children, K cohort (child)	National	both	8-9	8-9	2020	2123	2018	2120	
Australia	2007-2008	National Health Survey	National	both	18+	18+	951	970	136	148	
Australia	2008-2010	North West Adelaide Health Study	Community	urban	24+	24+	23	27			
Australia	2010	The Longitudinal Study of Australian Children, B cohort (infant)	National	both	6-7	6-7	2019	2142	2011	2137	
Australia	2010	The Longitudinal Study of Australian Children, K cohort (child)	National	both	10-11	10-11	1943	2044	1940	2042	
Australia	2011-2013	Australian Health Survey	National	both	5+	5+	3934	3920	2386	2451	3
Australia	2011-2013	International Study of Childhood Obesity, Lifestyle and the Environment (ISCOLE)	Community	urban	9-11	9-11	285	243	285	243	
Australia	2012	The Longitudinal Study of Australian Children, B cohort (infant)	National	both	8-9	8-9	1952	2053	1946	2041	
Australia	2012	The Longitudinal Study of Australian Children, K cohort (child)	National	both	12-13	12-13	1882	1963	1850	1948	
Australia	2014	The Longitudinal Study of Australian Children, B cohort (infant)	National	both	10-11	10-11	1763	1848	1742	1829	
Australia	2014	The Longitudinal Study of Australian Children, K cohort (child)	National	both	14-15	14-15	1634	1709	1581	1694	
Australia	2014-2015	National Health Survey	National	both	5+	5+	2687	2770	1673	1824	
Australia	2016	The Longitudinal Study of Australian Children, B cohort (infant)	National	both	12-13	12-13	1560	1647	1529	1638	
Australia	2016	The Longitudinal Study of Australian Children, K cohort (child)	National	both	16-17	16-17	1427	1484	1380	1477	
Australia	2018	The Longitudinal Study of Australian Children, B cohort (infant)	National	both	14-15	14-15	1433	1532	1403	1523	
Australia	2018	The Longitudinal Study of Australian Children, K cohort (child)	National	both	18-19	18-19	1269	1310	1233	1304	
Australia	2017-2018	National Health Survey	National	both	18+	18+	1203	1116	166	155	
Canada	1981	Canada Fitness Survey	National	both	7-64	7-64	758	838			
Canada	1986-1992	Canada Heart Health Survey	National	both	18-74	18-74	142	110	309	300	1
Canada	2004-2005	Canadian Multicentre Osteoporosis Study (CaMos) - Youth baseline	Subnational	both	16-24	16-24	520	471	262	242	
Canada	2006-2008	Canadian Multicentre Osteoporosis Study (CaMos) - Youth Year 2 follow-up	Subnational	both	17-27	17-27	383	331	110	93	
Canada	2007-2009	Canadian Health Measures Survey, Cycle 1	National	both	6-79	6-79	1284	1280	1028	1053	
Canada	2009-2011	Canadian Health Measures Survey, Cycle 2	National	both	5-79	5-79	1394	1402	1148	1189	
Canada	2011-2013	International Study of Childhood Obesity, Lifestyle and the Environment (ISCOLE)	Community	urban	9-11	9-11	328	239	327	238	
Canada	2012-2013	Canadian Health Measures Survey, Cycle 3	National	both	5-79	5-79	1277	1289	1095	1106	
Canada	2014-2015	Canadian Health Measures Survey, Cycle 4	National	both	5-79	5-79	1297	1297	1116	1131	
Canada	2016-2017	Canadian Health Measures Survey, Cycle 5	National	both	5-79	5-79	1271	1296	1124	1140	
Canada	2018-2019	Canadian Health Measures Survey, Cycle 6	National	both	5-79	5-79	1282	1245	1124	1090	
Ireland	1998	Survey of Lifestyle, Attitudes and Nutritional in Ireland 1998	National	both	18+	18+	46	25	6		
Ireland	2002	Survey of Lifestyle, Attitudes and Nutritional in Ireland 2002	National	both	18+	18+	32	18	6		
Ireland	2003-2004	National Children's Food Survey	National	both	5-12	5-12	301	293	301	293	
Ireland	2005-2006	National Teens Food Survey	National	both	13-17	13-17	218	223	216	224	
Ireland	2006-2007	Survey of Lifestyle, Attitudes and Nutritional in Ireland 2006-2007	National	both	18+	18+	171	162	28	27	
Ireland	2008	Childhood Obesity Surveillance Initiative 1	National	both	7	7	1285	1098	1285	1098	
Ireland	2007-2008	Growing Up in Ireland - Child Cohort	National	both	9	9	4112	3901	4101	3891	4
Ireland	2008-2010	National Adult Nutrition Survey	National	both	18+	18+	168	191	19	35	
Ireland	2010	Murtagh et al., Pediatr Exerc Sci 25(2):300-7, 2013	Community	rural	7-12	7-12	11	20	11	20	
Ireland	2010	Murtagh et al., Pediatr Exerc Sci 25(2):300-7, 2013	Community	rural	7-12	7-12	19	19	19	19	
Ireland	2010	Murtagh et al., Pediatr Exerc Sci 25(2):300-7, 2013	Community	rural	7-12	7-12	12	14	12	14	
Ireland	2010	Murtagh et al., Pediatr Exerc Sci 25(2):300-7, 2013	Community	rural	7-12	7-12	21	16	21	16	
Ireland	2010	Childhood Obesity Surveillance Initiative 2	National	both	6-9	6-9	1533	1452	1533	1452	
Ireland	2011-2012	Growing Up in Ireland - Child Cohort	National	both	13	13	3706	3550	3661	3537	4
Ireland	2012-2013	Childhood Obesity Surveillance Initiative 3	National	both	6-9	6-9	1054	1087	1054	1087	
Ireland	2013	Growing Up in Ireland - Infant Cohort	National	both	5	5	4379	4493	4372	4482	4
Ireland	2015	Active Classrooms Study	Community	both	8-11	8-11	120	123	120	123	
Ireland	2015-2016	Growing Up in Ireland - Child Cohort	National	both	18	18	595	556	583	551	4
Ireland	2013-2016	Project Spraoi	Community	both	5-11	5-11	427	472	427	472	
Ireland	2015-2016	Childhood Obesity Surveillance Initiative 4	National	both	6-10	6-10	1681	1441	1679	1441	
Ireland	2016	Growing Up in Ireland - Infant Cohort	National	both	7-8	7-8	1921	2016	1921	2016	4
Ireland	2017-2018	Growing Up in Ireland - Infant Cohort	National	both	9	9	3906	3945	3877	3925	4
Ireland	2017-2018	National Children's Food Survey II	National	both	5-12	5-12	298	298	298	298	
Ireland	2018-2019	Childhood Obesity Surveillance Initiative 5	National	both	6-12	6-12	2768	2793	2768	2793	

Country	Study years	Survey/study name/citation	Level of representativeness	Rural, urban or both	Age range as in NCD-RisC database *		Sample size (height)		Sample size (BMI)		Note
					Female	Male	Female	Male	Female	Male	
Ireland	2018-2019	Growing Up in Ireland - Child Cohort	National	both	20	20	2270	2094			4
Ireland	2019-2020	National Teens' Food Survey II	National	both	13-18	13-18	215	211	214	211	
New Zealand	1989	The Life in New Zealand Survey	National	both	15+	15+	98	87	104	97	1
New Zealand	1990-1993	Williams, N Z Med J 113(1114):308-11, 2000	Community	both	18-21	18-21			417	450	
New Zealand	1996-1997	National Nutrition Survey	National	both	15+	15+	587	383	156	128	
New Zealand	2002	National Children's Nutrition Survey	National	both	5-14	5-14	1487	1566	1484	1564	
New Zealand	2002-2003	New Zealand Health Survey	National	both	15+	15+	1381	935	325	293	
New Zealand	2006-2007	New Zealand Health Survey	National	both	5+	5+	2844	2685	1901	2014	
New Zealand	2008-2009	New Zealand Adult Nutrition Survey	National	both	15+	15+	709	573	404	339	
New Zealand	2011-2012	New Zealand Health Survey	National	both	5+	5+	2190	2154	1406	1511	
New Zealand	2012-2013	New Zealand Health Survey	National	both	5+	5+	2426	2301	1452	1573	
New Zealand	2013-2014	New Zealand Health Survey	National	both	5+	5+	2661	2491	1644	1681	
New Zealand	2014-2015	New Zealand Health Survey	National	both	5+	5+	2768	2580	1745	1775	
New Zealand	2015-2016	New Zealand Health Survey	National	both	5+	5+	2603	2649	1603	1816	
New Zealand	2016-2017	New Zealand Health Survey	National	both	5+	5+	2511	2433	1562	1682	
New Zealand	2017-2018	New Zealand Health Survey	National	both	5+	5+	2610	2631	1664	1785	
New Zealand	2018-2019	New Zealand Health Survey	National	both	5+	5+	2527	2466	1611	1671	
New Zealand	2019-2020	New Zealand Health Survey	National	both	5+	5+	1749	1672	1134	1229	
United Kingdom	1986-1987	Dietary and Nutritional Survey of British Adults 1986-1987	National	both	16-64	16-64	22	35	82	111	1
United Kingdom	1991-1992	Health Survey for England	National	both	16+	16+	127	113	122	113	
United Kingdom	1993	Health Survey for England	National	both	16+	16+	462	442	442	441	
United Kingdom	1994	Health Survey for England	National	both	16+	16+	399	380	384	374	
United Kingdom	1995	Health Survey for England	National	both	5+	5+	1772	1725	1711	1689	
United Kingdom	1995	Scottish Health Survey (SHeS)	Subnational	both	16-64	16-64	182	179	170	176	
United Kingdom	1996	Health Survey for England	National	both	5+	5+	3297	3031	1840	1904	
United Kingdom	1997	Health Survey for England	National	both	5+	5+	3587	3459	2810	2815	
United Kingdom	1997	National Diet and Nutrition Survey (NDNS)	National	both	5-18	5-18	899	934	896	933	
United Kingdom	1993-2000	EPIC Oxford	Subnational	both	20+	20+	7220	1393			
United Kingdom	1998	Health Survey for England	National	both	5+	5+	3080	2849	1737	1774	
United Kingdom	1998	Scottish Health Survey (SHeS)	Subnational	both	5-74	5-74	2328	2191	1550	1613	
United Kingdom	1999	Health Survey for England	National	both	5+	5+	1403	1353	849	872	
United Kingdom	1998-1999	SportsLinx	Community	urban	9-10	9-10	1369	1433	1364	1429	
United Kingdom	2000	Health Survey for England	National	both	5+	5+	1426	1400	851	903	
United Kingdom	1999-2000	SportsLinx	Community	urban	9-10	9-10	1468	1504	1439	1469	
United Kingdom	2001	Health Survey for England	National	both	5+	5+	2830	2460	1624	1538	
United Kingdom	2000-2001	National Diet and Nutrition Survey (NDNS)	National	both	19-64	19-64	165	140	9	9	
United Kingdom	2000-2001	SportsLinx	Community	urban	9-10	9-10	1159	1175	1154	1166	
United Kingdom	2002	Health Survey for England	National	both	5+	5+	4797	4532	3375	3468	
United Kingdom	2001-2002	SportsLinx	Community	urban	9-10	9-10	743	867	743	866	
United Kingdom	2003	Health Survey for England	National	both	5+	5+	2576	2300	1558	1488	
United Kingdom	2003	Scottish Health Survey (SHeS)	Subnational	both	5+	5+	1631	1495	1156	1135	
United Kingdom	2002-2003	SportsLinx	Community	urban	9-10	9-10	754	728	749	725	
United Kingdom	2004	Health Survey for England	National	both	5+	5+	1028	967	613	656	
United Kingdom	2003-2004	SportsLinx	Community	urban	9-10	9-10	1931	1907	1931	1906	
United Kingdom	2005	Health Survey for England	National	both	5+	5+	1691	1547	1138	1140	
United Kingdom	2004-2005	SportsLinx	Community	urban	9-10	9-10	1739	1736	1712	1724	
United Kingdom	2006	Health Survey for England	National	both	5+	5+	3666	3438	2739	2777	
United Kingdom	2006	Millennium Cohort Study	National	both	5-6	5-6	5945	6194	5938	6187	
United Kingdom	2005-2006	SportsLinx	Community	urban	9-10	9-10	1421	1460	1409	1455	
United Kingdom	2007	Health Survey for England	National	both	5+	5+	3153	3184	2639	2770	
United Kingdom	2006-2007	National Child Measurement Programme	National	both	5-11	5-11	358284	380590	358273	380581	
United Kingdom	2006-2007	SportsLinx	Community	urban	9-10	9-10	1688	1761	1683	1759	
United Kingdom	2007	Welsh Health Survey (WHS)	Subnational	both	5-15	5-15	762	788	751	782	
United Kingdom	2008	Health Survey for England	National	both	5+	5+	3933	3616	2849	2800	
United Kingdom	2008	Millennium Cohort Study	National	both	6-8	6-8	6783	6928	6762	6884	
United Kingdom	2007-2008	National Child Measurement Programme	National	both	5-11	5-11	374787	396801	374775	396795	
United Kingdom	2008	Scottish Health Survey (SHeS)	Subnational	both	5+	5+	1024	883	632	620	
United Kingdom	2007-2008	SportsLinx	Community	urban	9-10	9-10	1821	1855	1815	1852	
United Kingdom	2008	Welsh Health Survey (WHS)	Subnational	both	5-15	5-15	635	741	624	737	
United Kingdom	2009	Health Survey for England	National	both	5+	5+	1669	1733	1374	1505	
United Kingdom	2008-2009	National Child Measurement Programme	National	both	5-11	5-11	377149	398497	377146	398496	
United Kingdom	2009	Scottish Health Survey (SHeS)	Subnational	both	5+	5+	1307	1237	843	918	
United Kingdom	2008-2009	SportsLinx	Community	urban	9-10	9-10	1864	1837	1854	1824	
United Kingdom	2009	Welsh Health Survey (WHS)	Subnational	both	5-15	5-15	865	912	857	907	
United Kingdom	2010	Health Survey for England	National	both	5+	5+	2469	2396	1875	1980	
United Kingdom	2009-2010	National Child Measurement Programme	National	both	5-11	5-11	377962	398272	377960	398272	

Country	Study years	Survey/study name/citation	Level of representativeness	Rural, urban or both	Age range as in NCD-RisC database *		Sample size (height)		Sample size (BMI)		Note
					Female	Male	Female	Male	Female	Male	
United Kingdom	2008-2012	National Diet and Nutrition Survey (NDNS)	National	both	5+	5+	1534	1451	1259	1272	
United Kingdom	2010	Scottish Health Survey (SHeS)	Subnational	both	5+	5+	1051	976	575	639	
United Kingdom	2009-2010	SportsLinx	Community	urban	9-10	9-10	1449	1497	1429	1493	
United Kingdom	2010	Welsh Health Survey (WHS)	Subnational	both	5-15	5-15	870	901	859	892	
United Kingdom	2011	Health Survey for England	National	both	5+	5+	1283	1044	713	639	
United Kingdom	2010-2011	National Child Measurement Programme	National	both	5-11	5-11	373473	393149	373472	393146	
United Kingdom	2011	Scottish Health Survey (SHeS)	Subnational	both	5+	5+	1043	955	601	618	
United Kingdom	2010-2011	SportsLinx	Community	urban	9-10	9-10	1261	1338	1252	1332	
United Kingdom	2011	Welsh Health Survey (WHS)	Subnational	both	5-15	5-15	847	953	836	949	
United Kingdom	2012	Health Survey for England	National	both	5+	5+	1237	1069	672	695	
United Kingdom	2011-2013	International Study of Childhood Obesity, Lifestyle and the Environment (ISCOLE)	Community	urban	9-11	9-11	288	237	287	237	
United Kingdom	2012	Millennium Cohort Study	National	both	10-12	10-12	4980	5169	4965	5151	
United Kingdom	2011-2012	National Child Measurement Programme	National	both	5-11	5-11	373068	390385	373067	390383	
United Kingdom	2012	Scottish Health Survey (SHeS)	Subnational	both	5+	5+	858	778	590	569	
United Kingdom	2011-2012	SportsLinx	Community	urban	9-10	9-10	1378	1334	1330	1286	
United Kingdom	2012	Welsh Health Survey (WHS)	Subnational	both	5-15	5-15	721	808	712	804	
United Kingdom	2013	Health Survey for England	National	both	5+	5+	1307	1118	758	737	
United Kingdom	2012-2013	National Child Measurement Programme	National	both	5-11	5-11	376152	395648	376152	395648	
United Kingdom	2013	Scottish Health Survey (SHeS)	Subnational	both	5+	5+	836	830	543	594	
United Kingdom	2014	Health Survey for England	National	both	5+	5+	1186	1084	711	717	
United Kingdom	2013-2014	National Child Measurement Programme	National	both	5-11	5-11	386932	404989	386932	404990	
United Kingdom	2013-2014	National Diet and Nutrition Survey (NDNS)	National	both	5+	5+	574	525	464	469	
United Kingdom	2014	Scottish Health Survey (SHeS)	Subnational	both	5+	5+	822	761	563	558	
United Kingdom	2013-2014	Swan-Linx Project	Community	both	9-11	9-11	348	335	333	329	
United Kingdom	2015	Health Survey for England	National	both	18+	18+	558	388	66	63	
United Kingdom	2015	Millennium Cohort Study	National	both	13-15	13-15	5696	5695	5468	5625	
United Kingdom	2014-2015	National Child Measurement Programme	National	both	5-11	5-11	394748	412886	394750	412887	
United Kingdom	2015	Scottish Health Survey (SHeS)	Subnational	both	5+	5+	670	679	396	459	
United Kingdom	2015	Swan-Linx Project	Community	both	9-11	9-11	403	421	395	420	
United Kingdom	2016	Health Survey for England	National	both	18+	18+	555	413	59	61	
United Kingdom	2015-2016	National Child Measurement Programme	National	both	5-11	5-11	409834	427241	409834	427243	
United Kingdom	2015-2016	National Diet and Nutrition Survey (NDNS)	National	both	5+	5+	580	549	456	490	
United Kingdom	2016	Scottish Health Survey (SHeS)	Subnational	both	5+	5+	714	683	476	494	
United Kingdom	2016	Swan-Linx Project	Community	both	9-11	9-11	661	606	653	604	
United Kingdom	2017	Health Survey for England	National	both	18+	18+	470	360	56	49	
United Kingdom	2016-2017	National Child Measurement Programme	National	both	5-11	5-11	412586	430677	412586	430677	
United Kingdom	2017	Scottish Health Survey	Subnational	both	5+	5+	640	672	432	499	
United Kingdom	2018	Health Survey for England	National	both	18+	18+	516	381	53	63	
United Kingdom	2017-2018	National Child Measurement Programme	National	both	5-11	5-11	426632	444135	426632	444135	
United Kingdom	2016-2019	National Diet and Nutrition Survey (NDNS)	National	both	5+	5+	746	702	599	618	
United Kingdom	2018	Scottish Health Survey	Subnational	both	5+	5+	779	783	534	602	
United Kingdom	2019	Health Survey for England	National	both	18+	18+	510	370	79	72	
United Kingdom	2019	Scottish Health Survey	Subnational	both	5+	5+	869	820	602	634	
United States of America	1976-1977	The Bogalusa Heart Study	Community	rural	5-17	5-17	274	298			
United States of America	1978-1979	The Bogalusa Heart Study	Community	rural	5-17	5-17	597	582			
United States of America	1981-1982	The Bogalusa Heart Study	Community	rural	5-17	5-17	995	978			
United States of America	1983-1985	The Bogalusa Heart Study	Community	rural	5-17	5-17	833	775			
United States of America	1987-1988	The Bogalusa Heart Study	Community	rural	5-17	5-17	1595	1648			
United States of America	1988-1994	US NHANES III	National	both	5+	5+	3862	3661	3775	3634	
United States of America	1992-1994	The Bogalusa Heart Study	Community	rural	5-17	5-17	1593	1528	1593	1525	
United States of America	1996	National Longitudinal Study of Adolescent Health Wave II	National	both	11-21	11-21	2437	2256	2377	2217	5
United States of America	1998-1999	Coronary Artery Risk Detection in Appalachian Communities (CARDIAC), 5th Grade	Subnational	both	10-12	10-12	451	534	450	534	
United States of America	1999	Early Childhood Longitudinal Study	National	both	5-7	5-7	2493	2553	2476	2523	
United States of America	2000-2001	Coronary Artery Risk Detection in Appalachian Communities (CARDIAC), 5th Grade	Subnational	both	10-12	10-12	566	639	566	639	
United States of America	2000	Early Childhood Longitudinal Study	National	both	6-8	6-8	7761	8104	7548	7887	
United States of America	1999-2000	US NHANES 1999-2000	National	both	5+	5+	2210	2090	1714	1766	
United States of America	2001	Coronary Artery Risk Detection in Appalachian Communities (CARDIAC), 5th Grade	Subnational	both	10-12	10-12	1629	1724	1625	1724	
United States of America	2001-2002	National Longitudinal Study of Adolescent Health Wave III	National	both	18-28	18-28	2521	2178	318	227	5
United States of America	2002-2003	Coronary Artery Risk Detection in Appalachian Communities (CARDIAC), 5th Grade	Subnational	both	10-12	10-12	2588	2638	2585	2630	
United States of America	2002	Early Childhood Longitudinal Study	National	both	8-9	8-9	6689	6888	6674	6873	
United States of America	2001-2002	US NHANES 2001-2002	National	both	5+	5+	2470	2281	1812	1826	
United States of America	2003-2004	Coronary Artery Risk Detection in Appalachian Communities (CARDIAC), 5th Grade	Subnational	both	10-12	10-12	4063	4581	4055	4573	
United States of America	2004-2005	Coronary Artery Risk Detection in Appalachian Communities (CARDIAC), 5th Grade	Subnational	both	10-12	10-12	4276	4342	4272	4332	
United States of America	2004	Early Childhood Longitudinal Study	National	both	10-11	10-11	5122	5116	5115	5111	
United States of America	2003-2004	US NHANES 2003-2004	National	both	5+	5+	2157	2108	1643	1700	
United States of America	2005-2006	Coronary Artery Risk Detection in Appalachian Communities (CARDIAC), 2nd Grade	Subnational	urban	7-9	7-9	262	318	261	318	

Country	Study years	Survey/study name/citation	Level of representativeness	Rural, urban or both	Age range as in NCD-RisC database *		Sample size (height)		Sample size (BMI)		Note
					Female	Male	Female	Male	Female	Male	
United States of America	2005-2006	Coronary Artery Risk Detection in Appalachian Communities (CARDIAC), 5th Grade	Subnational	both	10-12	10-12	3712	4379	3705	4371	
United States of America	2006-2007	Coronary Artery Risk Detection in Appalachian Communities (CARDIAC), 2nd Grade	Subnational	both	7-9	7-9	265	346	264	344	
United States of America	2006-2007	Coronary Artery Risk Detection in Appalachian Communities (CARDIAC), 5th Grade	Subnational	both	10-12	10-12	2911	3532	2905	3526	
United States of America	2005-2006	US NHANES 2005-2006	National	both	5+	5+	2368	2154	1714	1746	
United States of America	2007-2008	Coronary Artery Risk Detection in Appalachian Communities (CARDIAC), 2nd Grade	Subnational	both	7-9	7-9	3356	3279	3354	3279	
United States of America	2007-2008	Coronary Artery Risk Detection in Appalachian Communities (CARDIAC), 5th Grade	Subnational	both	10-12	10-12	3074	3506	3070	3502	
United States of America	2007	Early Childhood Longitudinal Study	National	both	13-14	13-14	3890	3856	3829	3804	
United States of America	2008-2009	Coronary Artery Risk Detection in Appalachian Communities (CARDIAC), 2nd Grade	Subnational	both	7-9	7-9	4407	4231	4398	4220	
United States of America	2008-2009	Coronary Artery Risk Detection in Appalachian Communities (CARDIAC), 5th Grade	Subnational	both	10-12	10-12	3050	3582	3048	3581	
United States of America	2007-2008	US NHANES 2007-2008	National	both	5+	5+	1666	1765	1234	1340	
United States of America	2009-2010	Coronary Artery Risk Detection in Appalachian Communities (CARDIAC), 2nd Grade	Subnational	both	7-9	7-9	5260	4970	5255	4963	
United States of America	2009-2010	Coronary Artery Risk Detection in Appalachian Communities (CARDIAC), 5th Grade	Subnational	both	10-12	10-12	2929	3388	2927	3386	
United States of America	2008-2009	National Longitudinal Study of Adolescent Health Wave IV	National	both	24-34	24-34	1639	1291			5
United States of America	2010-2011	Coronary Artery Risk Detection in Appalachian Communities (CARDIAC), 2nd Grade	Subnational	both	7-9	7-9	5038	4786	5036	4785	
United States of America	2010-2011	Coronary Artery Risk Detection in Appalachian Communities (CARDIAC), 5th Grade	Subnational	both	10-12	10-12	2769	3246	2769	3243	
United States of America	2010	Early Childhood Longitudinal Study	National	both	5-6	5-6	7281	7687	7277	7683	
United States of America	2009-2010	US NHANES 2009-2010	National	both	5+	5+	1845	1872	1297	1402	
United States of America	2011-2012	Coronary Artery Risk Detection in Appalachian Communities (CARDIAC), 2nd Grade	Subnational	both	7-9	7-9	4130	3844	4127	3842	
United States of America	2011-2012	Coronary Artery Risk Detection in Appalachian Communities (CARDIAC), 5th Grade	Subnational	both	10-12	10-12	2089	2593	2086	2591	
United States of America	2011	Early Childhood Longitudinal Study	National	both	5-7	5-7	2460	2691	2460	2689	
United States of America	2011	Early Childhood Longitudinal Study	National	both	5-7	5-7	8245	8599	8237	8592	
United States of America	2012-2013	Coronary Artery Risk Detection in Appalachian Communities (CARDIAC), 2nd Grade	Subnational	both	7-9	7-9	4876	4330	4868	4326	
United States of America	2012-2013	Coronary Artery Risk Detection in Appalachian Communities (CARDIAC), 5th Grade	Subnational	both	10-12	10-12	2279	2726	2279	2726	
United States of America	2012	Early Childhood Longitudinal Study	National	both	6-8	6-8	2213	2388	2213	2386	
United States of America	2012	Early Childhood Longitudinal Study	National	both	6-8	6-8	7217	7513	7205	7498	
United States of America	2011-2013	International Study of Childhood Obesity, Lifestyle and the Environment (ISCOLE)	Community	urban	9-11	9-11	370	281	368	281	
United States of America	2011-2012	US NHANES 2011-2012	National	both	5+	5+	1757	1854	1292	1366	
United States of America	2013-2014	Coronary Artery Risk Detection in Appalachian Communities (CARDIAC), 2nd Grade	Subnational	both	7-9	7-9	5107	4712	5101	4708	
United States of America	2013-2014	Coronary Artery Risk Detection in Appalachian Communities (CARDIAC), 5th Grade	Subnational	both	10-12	10-12	1961	2365	1961	2364	
United States of America	2013	Early Childhood Longitudinal Study	National	both	7-9	7-9	6550	6794	6526	6778	
United States of America	2014-2015	Coronary Artery Risk Detection in Appalachian Communities (CARDIAC), 2nd Grade	Subnational	both	7-9	7-9	5202	4893	5200	4891	
United States of America	2014-2015	Coronary Artery Risk Detection in Appalachian Communities (CARDIAC), 5th Grade	Subnational	both	10-12	10-12	1867	2269	1867	2269	
United States of America	2014	Early Childhood Longitudinal Study	National	both	8-10	8-10	6021	6290	5981	6251	
United States of America	2013-2014	US NHANES 2013-2014	National	both	5+	5+	1875	1917	1410	1467	
United States of America	2015	Early Childhood Longitudinal Study	National	both	9-11	9-11	5578	5805	5512	5755	
United States of America	2016	Early Childhood Longitudinal Study	National	both	10-12	10-12	5231	5487	5170	5429	
United States of America	2015-2016	US NHANES 2015-2016	National	both	5+	5+	1831	1806	1337	1376	
United States of America	2017-2018	US NHANES 2017-2018	National	both	5+	5+	1556	1523	1158	1150	
United States of America	2019-2020	US NHANES 2019-2020	Subnational	both	5+	5+	1006	1066	740	838	6
Northwestern Europe											
Austria	1987	The Austrian Conscription Database	National	both		17-18				55292	1
Austria	1988	The Austrian Conscription Database	National	both		17-18		25865		51050	1
Austria	1989	The Austrian Conscription Database	National	both		17-18		49381		49380	1
Austria	1990	The Austrian Conscription Database	National	both		17-18		47103		47102	
Austria	1991	The Austrian Conscription Database	National	both		17-18		44288		44288	
Austria	1992	The Austrian Conscription Database	National	both		17-18		43555		43553	
Austria	1992	Vorarlberg Health Monitoring and Promotion Programme (VHM&PP)	Subnational	both	18+	18+	73	54	73	54	
Austria	1993	The Austrian Conscription Database	National	both		17-18		42357		42356	
Austria	1994	The Austrian Conscription Database	National	both		17-18		40402		40401	
Austria	1995	The Austrian Conscription Database	National	both		17-18		39813		39809	
Austria	1996	The Austrian Conscription Database	National	both		17-18		39587		39587	
Austria	1997	The Austrian Conscription Database	National	both		17-18		40408		40408	
Austria	1998	The Austrian Conscription Database	National	both		17-18		43131		43131	
Austria	1998	Vorarlberg Health Monitoring and Promotion Programme (VHM&PP)	Subnational	both	18+	18+	2339	1403	30	18	
Austria	1999	The Austrian Conscription Database	National	both		17-18		44163		44163	
Austria	1998-1999	CINDI survey Vorarlberg/Austria	Subnational	both	25-64	25-64	48	37			
Austria	2000	The Austrian Conscription Database	National	both		17-18		44278		44275	
Austria	2001	The Austrian Conscription Database	National	both		17-18		43318		43315	
Austria	2002	The Austrian Conscription Database	National	both		17-18		42899		42899	
Austria	2003	The Austrian Conscription Database	National	both		17-18		42389		42389	
Austria	2004	The Austrian Conscription Database	National	both		17-18		42774		42774	
Austria	2004	Vorarlberg Health Monitoring and Promotion Programme (VHM&PP)	Subnational	both	18+	18+	2052	1346	68	30	
Austria	2005	The Austrian Conscription Database	National	both		17-18		42907		42906	
Austria	2004-2005	Vorarlberg Health Monitoring and Promotion Programme (VHM&PP)	Subnational	both	6-17	6-17	15822	17503	15822	17503	
Austria	2006	The Austrian Conscription Database	National	both		17-18		44572		44571	
Austria	2007	The Austrian Conscription Database	National	both		17-18		44756		44755	

Country	Study years	Survey/study name/citation	Level of representativeness	Rural, urban or both	Age range as in NCD-RisC database *		Sample size (height)		Sample size (BMI)		Note
					Female	Male	Female	Male	Female	Male	
Austria	2006-2007	HELENA	Community	urban	12-17	12-17	211	191	211	191	
Austria	2008	The Austrian Conscription Database	National	both		17-18		44880		44880	
Austria	2009	The Austrian Conscription Database	National	both		17-18		45594		45594	
Austria	2008-2009	Vorarlberg Health Monitoring and Promotion Programme (VHM&PP)	Subnational	both	6-17	6-17	15294	16846	15294	16846	
Austria	2009-2011	Mayer et al., Ann Hum Biol 42(1):45-55, 2015	National	both	5-17	5-17	6339	6638	6340	6634	
Austria	2010	The Austrian Conscription Database	National	both		17-18		44968		44968	
Austria	2010-2012	Austrian Study on Nutritional Status 2012	National	both	6-80	6-80	250	213	183	181	
Austria	2011	The Austrian Conscription Database	National	both		17-18		44076		44076	
Austria	2012	The Austrian Conscription Database	National	both		17-18		42768		42768	
Austria	2011-2012	BMI in Upper Austrian children and adolescents	Subnational	both	6-17	6-17			6853	7808	
Austria	2013	The Austrian Conscription Database	National	both		17-18		41574		41574	
Austria	2012-2013	Vorarlberg Health Monitoring and Promotion Programme (VHM&PP)	Subnational	both	6-17	6-17	13648	14759	13648	14759	
Austria	2014	The Austrian Conscription Database	National	both		17-18		41740		41740	
Austria	2013-2014	Prevalence of obesity and motor performance in Tyrolean preschool children	Subnational	both	5	5	513	550	513	550	
Austria	2013-2017	Austrian Study on Nutrition (ASN)	National	both	19-64	19-64	242	130	5	4	
Austria	2015	The Austrian Conscription Database	National	both		17-18		39154		39154	
Austria	2014-2015	Influence of selected risk factors on the motor performance of 10 to 11-year-old schoolchildren	Subnational	both	10-11	10-11	129	197	129	197	
Austria	2016	The Austrian Conscription Database	National	both		17-19		35944		35944	
Austria	2015-2016	Childhood Obesity Surveillance Initiative 4	National	both	8-9	8-9	1175	1220	1175	1220	
Austria	2015-2017	EVA Tyrol Study Austria North East Tyrol	Subnational	both	14-17	14-17	636	545	636	545	
Austria	2015-2016	Vorarlberg Health Monitoring and Promotion Programme (VHM&PP)	Subnational	both	6-17	6-17	12047	13037	12047	13037	
Austria	2017	The Austrian Conscription Database	National	both		17-19		34400		34399	
Austria	2018	The Austrian Conscription Database	National	both		17-19		34188		34188	
Austria	2019	The Austrian Conscription Database	National	both		17-19		32185		32185	
Austria	2019	Childhood Obesity Surveillance Initiative 5	National	both	8-9	8-9	1152	1242	1137	1223	
Belgium	1996-1998	Flemish Study on Environment, Genes and Health Outcomes	Community	rural	10-84	10-84	129	132	76	72	
Belgium	1998-2000	Flemish Study on Environment, Genes and Health Outcomes	Community	rural	10-80	10-80	64	64	32	25	
Belgium	1999-2001	Flemish Study on Environment, Genes and Health Outcomes	Community	rural	10-81	10-81	60	56	34	29	
Belgium	2001	Flemish Study on Environment, Genes and Health Outcomes	Community	rural	10-78	10-78	62	67	22	19	
Belgium	2002-2003	Flemish Study on Environment, Genes and Health Outcomes	Community	rural	10-81	10-81	46	30	21	9	
Belgium	2002-2005	Flemish Study on Environment, Genes and Health Outcomes	Community	rural	10-88	10-88	125	123	68	70	
Belgium	2005-2008	Flemish Study on Environment, Genes and Health Outcomes	Community	rural	10-89	10-89	65	82	23	24	
Belgium	2006-2007	HELENA	Community	urban	12-17	12-17	180	156	180	156	
Belgium	2007-2008	Childhood Obesity Surveillance Initiative 1	Subnational	both	6-9	6-9	61755	64322	61754	64322	
Belgium	2007-2010	Identification and prevention of Dietary- and lifestyle-induced health EFfects In Children and infantS (IDEFICS)	Community	urban	5-9	5-9	834	822	834	822	
Belgium	2009-2010	Childhood Obesity Surveillance Initiative 2	Subnational	both	6-9	6-9	65373	67783	65365	67775	
Belgium	2010	EuropeaN Energy balance Research to prevent excessive weight Gain among Youth - The ENERGY-project	Subnational	urban	10-12	10-12	497	460	497	460	
Belgium	2009-2013	Flemish Study on Environment, Genes and Health Outcomes	Community	rural	20-88	20-88	24	16			
Belgium	2012-2013	Childhood Obesity Surveillance Initiative 3	Subnational	both	6-9	6-9	67876	70442	67874	70441	
Belgium	2010-2015	Flemish Study on Environment, Genes and Health Outcomes	Community	rural	15-87	15-87	48	38	2	2	
Belgium	2014-2015	Food Consumption Survey	National	urban	5-64	5-64	1036	1050	892	908	
Belgium	2018-2019	Belgian Health Examination Survey	National	both	18+	18+	76	65	6	7	
Denmark	1977	Copenhagen School Health Records Register	Community	urban	6-13	6-13	1004	1025			
Denmark	1978	Copenhagen School Health Records Register	Community	urban	6-13	6-13	2587	2594			
Denmark	1979	Copenhagen School Health Records Register	Community	urban	6-13	6-13	3855	3837			
Denmark	1980	Copenhagen School Health Records Register	Community	urban	6-13	6-13	5143	5211			
Denmark	1981	Copenhagen School Health Records Register	Community	urban	6-13	6-13	6859	6845			
Denmark	1982	Copenhagen School Health Records Register	Community	urban	6-13	6-13	7964	8087			
Denmark	1983	Copenhagen School Health Records Register	Community	urban	6-13	6-13	6941	6929			
Denmark	1984	Copenhagen School Health Records Register	Community	urban	6-13	6-13	4947	4886			
Denmark	1985	Copenhagen School Health Records Register	Community	urban	6-13	6-13	4275	4266			
Denmark	1986	Copenhagen School Health Records Register	Community	urban	6-13	6-13	4462	4533			
Denmark	1987	Copenhagen School Health Records Register	Community	urban	6-13	6-13	4544	4686			
Denmark	1988	Copenhagen School Health Records Register	Community	urban	6-13	6-13	4501	4738			
Denmark	1989	Copenhagen School Health Records Register	Community	urban	6-13	6-13	4598	4845			
Denmark	1990	Copenhagen School Health Records Register	Community	urban	6-13	6-13	4449	4669	4449	4669	
Denmark	1991	Copenhagen School Health Records Register	Community	urban	6-13	6-13	4811	4844	4811	4844	
Denmark	1992	Copenhagen School Health Records Register	Community	urban	6-13	6-13	5104	5243	5104	5243	
Denmark	1993	Copenhagen School Health Records Register	Community	urban	6-13	6-13	4890	4968	4890	4968	
Denmark	1994	Copenhagen School Health Records Register	Community	urban	6-13	6-13	4005	4065	4005	4065	
Denmark	1995	Copenhagen School Health Records Register	Community	urban	6-13	6-13	5379	5437	5379	5437	
Denmark	1996	Copenhagen School Health Records Register	Community	urban	6-13	6-13	4670	4674	4670	4674	
Denmark	1997	Copenhagen School Health Records Register	Community	urban	7-13	7-13	4025	4105	4025	4105	
Denmark	1998	Copenhagen School Health Records Register	Community	urban	8-13	8-13	3253	3203	3253	3203	
Denmark	1997-1998	The European Youth Heart Study	Community	urban	8-18	8-18	532	485	532	485	
Denmark	1999	Copenhagen School Health Records Register	Community	urban	9-13	9-13	2777	2860	2777	2860	

Country	Study years	Survey/study name/citation	Level of representativeness	Rural, urban or both	Age range as in NCD-RisC database *		Sample size (height)		Sample size (BMI)		Note
					Female	Male	Female	Male	Female	Male	
Denmark	2000	Copenhagen School Health Records Register	Community	urban	10-13	10-13	1923	1911	1923	1911	
Denmark	2001	Copenhagen School Health Records Register	Community	urban	11-13	11-13	1595	1594	1595	1594	
Denmark	2001-2002	The Copenhagen School Child Intervention Study	Community	urban	5-8	5-8	329	362	329	362	
Denmark	2002	Copenhagen School Health Records Register	Community	urban	12-13	12-13	895	860	895	860	
Denmark	2001-2003	Copenhagen City Heart Study	Subnational	urban	20+	20+	281	171			
Denmark	2003	Copenhagen School Health Records Register	Community	urban	13	13	369	322	369	322	
Denmark	2003-2004	The European Youth Heart Study	Community	urban	8-17	8-17	509	392	509	392	
Denmark	2003-2004	Copenhagen General Population Study 1	Subnational	urban	20+	20+	136	92			
Denmark	2004-2005	The Copenhagen School Child Intervention Study	Community	urban	8-11	8-11	130	121	130	121	
Denmark	2005	Copenhagen General Population Study 1	Subnational	urban	20+	20+	123	85			
Denmark	2006	The Danish Conscription Database	National	both		17-26		12860		9823	
Denmark	2006	Copenhagen General Population Study 1	Subnational	urban	20+	20+	81	59			
Denmark	2007	The Danish Conscription Database	National	both		17-26		27007		20753	
Denmark	2006-2008	The Health2006 Cohort	Subnational	urban	18-71	18-71	196	109	13	14	
Denmark	2007	Copenhagen General Population Study 1	Subnational	urban	20+	20+	243	211			
Denmark	2008	The Childhood Health Activity and Motor Performance School Study	Community	both	5-10	5-10	627	545	626	544	
Denmark	2008	The Danish Conscription Database	National	both		17-26		24278		19590	
Denmark	2008	The Copenhagen School Child Intervention Study	Community	urban	12-14	12-14	112	99	111	99	
Denmark	2007-2008	The Danish Health Examination Survey 2007-2008	National	both	18+	18+	703	365	100	63	
Denmark	2008	Copenhagen General Population Study 1	Subnational	urban	20+	20+	129	100			
Denmark	2009	The Childhood Health Activity and Motor Performance School Study	Community	both	6-12	6-12	246	238	246	238	
Denmark	2009	The Childhood Health Activity and Motor Performance School Study	Community	both	6-11	6-11	243	231	243	231	
Denmark	2009	The Danish Conscription Database	National	both		17-26		26604		19876	
Denmark	2009	Copenhagen General Population Study 1	Subnational	urban	20+	20+	43	43			
Denmark	2010	The Childhood Health Activity and Motor Performance School Study	Community	both	7-13	7-13	248	254	248	253	
Denmark	2010	The Childhood Health Activity and Motor Performance School Study	Community	both	7-12	7-12	235	234	235	234	
Denmark	2010	The Danish Conscription Database	National	both		17-26		30656		23027	
Denmark	2009-2010	The European Youth Heart Study	Community	both	14-28	14-28	563	482	219	176	
Denmark	2010	Copenhagen General Population Study 1	Subnational	urban	20+	20+	84	64			
Denmark	2011	The Childhood Health Activity and Motor Performance School Study	Community	both	8-13	8-13	241	245	241	245	
Denmark	2011	The Danish Conscription Database	National	both		17-26		30636		23679	
Denmark	2011	Copenhagen General Population Study 1	Subnational	urban	20+	20+	74	46			
Denmark	2012	The Childhood Health Activity and Motor Performance School Study	Community	both	9-14	9-14	251	274	249	273	
Denmark	2012	The Danish Conscription Database	National	both		17-26		29573		23366	
Denmark	2011-2012	The OPUS School Meal Study	Subnational	both	8-11	8-11	388	427	388	427	
Denmark	2012	Copenhagen General Population Study 1	Subnational	urban	20+	20+	47	52			
Denmark	2011-2012	The Health2006 cohort - 5-year follow-up	Subnational	urban	24-76	24-76	58	39			
Denmark	2013	The Childhood Health Activity and Motor Performance School Study	Community	both	10-15	10-15	210	225	208	225	
Denmark	2013	The Danish Conscription Database	National	both		17-26		30523		24466	
Denmark	2013	Learning, Cognition and Motion (LCoMotion)	Subnational	both	11-14	11-14	365	353	365	353	
Denmark	2013	Copenhagen General Population Study 1	Subnational	urban	20+	20+	129	97			
Denmark	2014	The Danish Conscription Database	National	both		17-26		32367		26595	
Denmark	2012-2015	The Danish study of Functional Disorders (DanFunD)	Subnational	urban	18-72	18-72	357	289	29	17	
Denmark	2014	Copenhagen General Population Study 2	Subnational	urban	20+	20+	32	22			
Denmark	2015	The Childhood Health Activity and Motor Performance School Study	Community	both	12-17	12-17	122	122	120	122	
Denmark	2015	The Danish Conscription Database	National	both		17-26		28873		24458	
Denmark	2014-2015	Copenhagen General Population Study 1	Subnational	urban	20+	20+	65	50			
Denmark	2015	Copenhagen General Population Study 2	Subnational	urban	20+	20+	93	87			
Denmark	2016	The Danish Conscription Database	National	both		17-29		29057		24145	
Denmark	2015-2016	Childhood Obesity Surveillance Initiative 4	National	both	6-7	6-7	1273	1327	1271	1326	
Denmark	2016	Copenhagen General Population Study 2	Subnational	urban	20+	20+	103	89			
Denmark	2017	The Danish Conscription Database	National	both		17-29		31057		26415	
Denmark	2017	Copenhagen General Population Study 2	Subnational	urban	20+	20+	46	31			
Denmark	2018	The Danish Conscription Database	National	both		17-29		27597		24085	
Denmark	2019	The Danish Conscription Database	National	both		17-29		27412		22663	
Denmark	2019	Childhood Obesity Surveillance Initiative 5	National	both	6-7	6-7	1288	1201	1288	1201	
Finland	1980	Young Finns Study 1980	National	both	5-18	5-18	621	600			
Finland	1983	Young Finns Study 1983	National	both	6-21	6-21	773	786			
Finland	1986	Young Finns Study 1986	National	both	9-24	9-24	730	715			
Finland	2001	Young Finns Study 2001	National	both	24-39	24-39	581	487			
Finland	2001-2002	Northern Finland Birth Cohort 1986	Community	both	15-17	15-17	3202	3133	3194	3125	
Finland	2002	The National FINRISK Study	National	both	25-74	25-74	426	278			
Finland	2007	The National FINRISK Study	National	both	25-74	25-74	295	216			
Finland	2007	Young Finns Study 2007	National	both	30-45	30-45	161	160			
Finland	2011-2013	International Study of Childhood Obesity, Lifestyle and the Environment (ISCOLE)	Community	urban	9-11	9-11	283	253	282	253	
Finland	2012	The National FINRISK Study	National	both	25-74	25-74	262	203			

Country	Study years	Survey/study name/citation	Level of representativeness	Rural, urban or both	Age range as in NCD-RisC database *		Sample size (height)		Sample size (BMI)		Note
					Female	Male	Female	Male	Female	Male	
Finland	2015-2016	Childhood Obesity Surveillance Initiative 4	National	both	7-9	7-9	6388	6598	6388	6598	
Finland	2016	Register of Primary Health Care visits	Subnational	both	5-17	5-17	122959	129618	122959	129618	
Finland	2017	The FinHealth Survey	National	both	18+	18+	280	241	19	11	
Finland	2017	Register of Primary Health Care visits	Subnational	both	5-17	5-17	139888	148712	139888	148712	
Finland	2018	Register of Primary Health Care visits	Subnational	both	5-17	5-17	148201	157581	148201	157581	
Finland	2019	Childhood Obesity Surveillance Initiative 5	National	both	7-9	7-9	5096	5750	5096	5749	
Germany	1990	The German Conscription Database	Subnational	both		19		206615		206599	
Germany	1991	The German Conscription Database	National	both		19		138205		138195	
Germany	1992	The German Conscription Database	National	both		19		220992		220956	
Germany	1993	The German Conscription Database	National	both		19		188700		188655	
Germany	1994	The German Conscription Database	National	both		19		155442		155426	
Germany	1995	The German Conscription Database	National	both		19		185793		185762	
Germany	1996	The German Conscription Database	National	both		19		191265		191260	
Germany	1997	The German Conscription Database	National	both		19		148774		148738	
Germany	1997-1999	German National Health Interview and Examination Survey (GNHIES98)	National	both	18-79	18-79	634	645	124	142	
Germany	1998	The German Conscription Database	National	both		19		146563		146528	
Germany	1999	The German Conscription Database	National	both		19		292829		292732	
Germany	1997-2001	Study of Health in Pomerania (SHIP-0) baseline study	Subnational	both	20-80	20-80	318	274			
Germany	1999-2001	KORA-S4 (Cooperative Health Research in the Augsburg Region)	Community	both	24-75	24-75	171	149			
Germany	2002	Echinoccoccus Multilocularis and Internal Diseases in Leutkirch	Community	urban	12-65	12-65	316	313	177	162	
Germany	2002-2006	Study of Health in Pomerania (SHIP-1) 5-year follow-up	Subnational	both	25-85	25-85	86	60			
Germany	2003-2006	German Health Interview and Examination Survey for Children and Adolescents (KiGGS)	National	both	5-17	5-17	6286	6622	6260	6602	
Germany	2006-2007	HELENA	Community	urban	12-17	12-17	194	282	194	282	
Germany	2008	The German Conscription Database	National	both		19		98942		98926	
Germany	2009	The German Conscription Database	National	both		19		111461		111455	
Germany	2007-2010	Identification and prevention of Dietary- and lifestyle-induced health EFfects In Children and infantS (IDEFICS)	Community	urban	5-9	5-9	762	772	762	772	
Germany	2010	The German Conscription Database	National	both		19		101928		101911	
Germany	2008-2011	German Health Interview and Examination Survey for adults 2008-11 (DEGS1)	National	both	18-79	18-79	545	520	92	123	
Germany	2008-2012	Study of Health in Pomerania, second cohort (SHIP-TREND)	Subnational	both	20-79	20-79	194	190			
Germany	2014-2017	German Health Interview and Examination Survey for Children and Adolescents (KiGGS) - Wave 2	National	both	5-17	5-17	1584	1541	1584	1541	
Germany	2018-2019	Childhood Obesity Surveillance Initiative 5	Community	urban	7-9	7-9	1111	1110	1111	1110	
Greenland	2005-2010	Population Health Survey in Greenland	National	both	18+	18+	333	242	50	40	
Iceland	2005-2011	Risk Evaluation For INfarct Estimates (REFINE)	Subnational	urban	20-73	20-73	249	232			
Iceland	2010-2012	Risk Evaluation For INfarct Estimates (REFINE) follow-up visit (REFINELO)	Subnational	urban	26-74	26-74	13	6			
Luxembourg	2007-2009	Observation of cardiovascular risk factors in Luxembourg (ORISCAV-LUX)	National	both	18-69	18-69	115	106	13	16	
Luxembourg	2013-2015	European Health Examination Survey	National	both	25-64	25-64	65	42			
Luxembourg	2016-2018	Observation of cardiovascular risk factors in Luxembourg (ORISCAV-LUX2)	National	both	25-79	25-79	23	29			
Netherlands	1998-2001	Regenboog Project	National	both	12-89	12-89	483	409	205	179	
Netherlands	2004-2006	Prevention and Incidence of Asthma and Mite Allergy (PIAMA)	National	both	7-9	7-9	1103	1106	1103	1106	
Netherlands	2008-2011	Prevention and Incidence of Asthma and Mite Allergy (PIAMA)	National	both	12-13	12-13	771	738	769	738	
Netherlands	2008-2011	Amsterdam Born Children and their Development Study (ABCD)	Community	both	5-7	5-7	1435	1453	1434	1450	
Netherlands	2010	EuropeaN Energy balance Research to prevent excessive weight Gain among Youth - The ENERGY-project	National	both	10-12	10-12	400	404	399	404	
Netherlands	2011-2013	GECKO Drenthe Onderzoek	Subnational	rural	5-7	5-7	1133	1139	1133	1139	
Netherlands	2011-2015	Healthy Life in an Urban Setting (HELIUS)	Community	urban	18-71	18-71	471	289	50	30	
Netherlands	2012-2014	Prevention and Incidence of Asthma and Mite Allergy (PIAMA)	National	both	15-17	15-17	415	386	413	386	
Netherlands	2012-2016	Amsterdam Born Children and their Development Study (ABCD)	Community	both	9-12	9-12	1082	1074	1080	1073	
Netherlands	2016-2018	GECKO Drenthe Onderzoek	Subnational	rural	8-12	8-12	1117	1085	1114	1083	
Norway	1995-1997	Young-HUNT1 Study	Subnational	rural	12-21	12-21	4251	4196	4241	4194	
Norway	1995-1997	HUNT2 study	Subnational	rural	20+	20+	4764	3963			
Norway	1999-2000	European Youth Heart Study	Community	urban	9-15	9-15	369	365	369	364	
Norway	2000-2001	Young-HUNT2 Study	Subnational	rural	16-21	16-21	901	764	884	755	
Norway	2000-2003	The Oslo cohort (HUBRO), the Oppland and Hedmark cohort (OPPHED), and the Troms and Finnmark cohort (TROFINN) of Cohort of Norway (CONOR)	Subnational	both	30-76	30-76	3768	3024			
Norway	2005-2006	Physical Activity among Norwegian Children and Adolescents	National	both	8-16	8-16	1058	1186	1055	1186	
Norway	2006-2008	Young-HUNT3 Study	Subnational	rural	12-21	12-21	3797	3809	3777	3788	
Norway	2006-2008	HUNT3 Study	Subnational	rural	20+	20+	2638	1859			
Norway	2008	Childhood Obesity Surveillance Initiative 1	National	both	8	8	1399	1435	1399	1435	
Norway	2010	Childhood Obesity Surveillance Initiative 2	National	both	8	8	1286	1335	1286	1335	
Norway	2010	EuropeaN Energy balance Research to prevent excessive weight Gain among Youth - The ENERGY-project	Subnational	urban	10-13	10-13	492	458	491	458	
Norway	2012	Childhood Obesity Surveillance Initiative 3	National	both	8	8	1381	1492	1381	1492	
Norway	2015-2016	Childhood Obesity Surveillance Initiative 4	National	both	7-8	7-8	1645	1690	1645	1690	
Sweden	1978	BMI Epidemiology Study	Community	urban		7		212			
Sweden	1979	BMI Epidemiology Study	Community	urban		8		409			
Sweden	1979-1980	1973 Birth Cohort	National	both	6	6	441	451			
Sweden	1980	BMI Epidemiology Study	Community	urban		9		324			
Sweden	1980-1981	1973 Birth Cohort	National	both	7	7	1334	1428			

Country	Study years	Survey/study name/citation	Level of representativeness	Rural, urban or both	Age range as in NCD-RisC database *		Sample size (height)		Sample size (BMI)		Note
					Female	Male	Female	Male	Female	Male	
Sweden	1981-1982	1973 Birth Cohort	National	both	8	8	902	935			
Sweden	1982-1983	1973 Birth Cohort	National	both	9	9	669	717			
Sweden	1983	BMI Epidemiology Study	Community	urban		7		103			
Sweden	1983-1984	1973 Birth Cohort	National	both	10	10	1367	1488			
Sweden	1984	BMI Epidemiology Study	Community	urban		8		407			
Sweden	1984-1985	1973 Birth Cohort	National	both	11	11	542	523			
Sweden	1985	BMI Epidemiology Study	Community	urban		9		357			
Sweden	1985-1986	1973 Birth Cohort	National	both	12	12	1226	1323			
Sweden	1986-1987	1973 Birth Cohort	National	both	13	13	823	799	729	709	1
Sweden	1986-1987	1981 Birth Cohort	National	both	5	5	961	940	958	935	1
Sweden	1987	The Swedish Conscription Database	National	both		17-18				49300	1
Sweden	1987-1988	1973 Birth Cohort	National	both	14	14	1342	1455	1294	1405	1
Sweden	1987-1988	1981 Birth Cohort	National	both	6	6	781	811	780	808	1
Sweden	1988	The Swedish Conscription Database	National	both		17-18		17462		47918	1
Sweden	1988	BMI Epidemiology Study	Community	urban		7		65			
Sweden	1988-1989	1973 Birth Cohort	National	both	15	15	1008	1069	929	964	1
Sweden	1988-1989	1981 Birth Cohort	National	both	7	7	1499	1521	1489	1508	1
Sweden	1989	The Swedish Conscription Database	National	both		17-18		48106		48106	1
Sweden	1989	BMI Epidemiology Study	Community	urban		8		407			
Sweden	1989-1990	1973 Birth Cohort	National	both	16	16	856	971	785	912	
Sweden	1989-1990	1981 Birth Cohort	National	both	8	8	1535	1515	1511	1497	
Sweden	1990	BMI Epidemiology Study	Community	urban		9		379		379	
Sweden	1990	The Swedish Conscription Database	National	both		17-18		48876		48876	
Sweden	1990-1991	1973 Birth Cohort	National	both	17	17	610	910	588	881	
Sweden	1990-1991	1981 Birth Cohort	National	both	9	9	1118	1102	1108	1091	
Sweden	1991	The Swedish Conscription Database	National	both		17-18		49147		49147	
Sweden	1992	1973 Birth Cohort	National	both	18	18	402	1296	390	1290	
Sweden	1991-1992	1981 Birth Cohort	National	both	10	10	1434	1411	1415	1405	
Sweden	1992	The Swedish Conscription Database	National	both		17-18		47272		47272	
Sweden	1992-1993	1981 Birth Cohort	National	both	11	11	812	807	785	785	
Sweden	1993	BMI Epidemiology Study	Community	urban		7		125		125	
Sweden	1993	The Swedish Conscription Database	National	both		17-18		46241		46241	
Sweden	1993-1994	1981 Birth Cohort	National	both	12	12	1405	1343	1378	1318	
Sweden	1994	BMI Epidemiology Study	Community	urban		8		376		376	
Sweden	1994	The Swedish Conscription Database	National	both		17-18		43644		43644	
Sweden	1994-1995	1981 Birth Cohort	National	both	13	13	1015	964	954	924	
Sweden	1995	BMI Epidemiology Study	Community	urban		9		293		293	
Sweden	1995	The Swedish Conscription Database	National	both		17-18		43654		43654	
Sweden	1995-1996	1981 Birth Cohort	National	both	14	14	1316	1238	1266	1202	
Sweden	1996	The Swedish Conscription Database	National	both		17-18		43773		43773	
Sweden	1996-1997	1981 Birth Cohort	National	both	15	15	1190	1187	1134	1143	
Sweden	1997	The Swedish Conscription Database	National	both		17-18		37068		37068	
Sweden	1997-1998	1981 Birth Cohort	National	both	16	16	897	850	862	819	
Sweden	1998	BMI Epidemiology Study	Community	urban		7		707		707	
Sweden	1998	The Swedish Conscription Database	National	both		17-18		42544		42544	
Sweden	1998-1999	1981 Birth Cohort	National	both	17	17	178	416	171	406	
Sweden	1999	1981 Birth Cohort	National	both	18	18	23	831	23	831	
Sweden	1999	BMI Epidemiology Study	Community	urban		8		980		980	
Sweden	1998-1999	European Youth Heart Study (EYHS) I	Subnational	urban	8-16	8-16	603	526	602	525	
Sweden	1999	The Swedish Conscription Database	National	both		17-18		37844		37844	
Sweden	1999	MONICA Northern Sweden	Subnational	both	25-74	25-74	55	62			
Sweden	2000	BMI Epidemiology Study	Community	urban		9		860		860	
Sweden	2000	The Swedish Conscription Database	National	both		17-18		31319		31319	
Sweden	2000-2002	The COMPASS study	Community	urban	14-16	14-16	1597	1718	1597	1718	
Sweden	2001	The Swedish Conscription Database	National	both		17-18		29764		29764	
Sweden	2002	The Swedish Conscription Database	National	both		17-18		24575		24575	
Sweden	2003	BMI Epidemiology Study	Community	urban		7		15		15	
Sweden	2003	The Swedish Conscription Database	National	both		17-18		29192		29192	
Sweden	2001-2004	Swedish INTERGENE Cohort Study	Subnational	both	24-76	24-76	107	82			
Sweden	2004	BMI Epidemiology Study	Community	urban		8		285		285	
Sweden	2004	The Swedish Conscription Database	National	both		17-18		27851		27851	
Sweden	2004	MONICA Northern Sweden	Subnational	both	26-75	26-75	50	62			
Sweden	2005	BMI Epidemiology Study	Community	urban		9		301		301	
Sweden	2004-2005	European Youth Heart Study (EYHS) II	Subnational	urban	15-21	15-21	262	196	151	126	
Sweden	2005	The Swedish Conscription Database	National	both		17-18		25831		25831	
Sweden	2007	BMI Epidemiology Study	Community	urban		7		1327		1327	

Country	Study years	Survey/study name/citation	Level of representativeness	Rural, urban or both	Age range as in NCD-RisC database *		Sample size (height)		Sample size (BMI)		Note
					Female	Male	Female	Male	Female	Male	
Sweden	2006-2007	HELENA	Community	urban	12-17	12-17	208	132	208	132	
Sweden	2008	BMI Epidemiology Study	Community	urban		7-8		2501		2501	
Sweden	2008	Childhood Obesity Surveillance Initiative 1	National	both	7-9	7-9	2189	2374	2189	2374	
Sweden	2009	BMI Epidemiology Study	Community	urban		7-9		3706		3706	
Sweden	2007-2010	Identification and prevention of Dietary- and lifestyle-induced health EFfects In Children and infantS (IDEFICS)	Community	urban	5-9	5-9	557	557	557	557	
Sweden	2009	MONICA Northern Sweden	Subnational	both	25-74	25-74	80	61			
Sweden	2010	BMI Epidemiology Study	Community	urban		8-9		2425		2425	
Sweden	2011	BMI Epidemiology Study	Community	urban		9		1412		1412	
Sweden	2012	BMI Epidemiology Study	Community	urban		7		1551		1551	
Sweden	2013	BMI Epidemiology Study	Community	urban		7-8		2875		2875	
Sweden	2014	BMI Epidemiology Study	Community	urban		7-9		4251		4251	
Sweden	2014	MONICA Northern Sweden	Subnational	both	25-74	25-74	44	45			
Sweden	2015	BMI Epidemiology Study	Community	urban		8-9		1917		1917	
Sweden	2016	BMI Epidemiology Study	Community	urban		9		35		35	
Sweden	2015-2016	Childhood Obesity Surveillance Initiative 4	National	both	6-9	6-9	3798	4071	3798	4070	
Sweden	2019	Childhood Obesity Surveillance Initiative 5	National	both	6-9	6-9	30002	31644	30002	31642	
Switzerland	1993-1994	The Swiss Conscription Database	National	both		19		23499			
Switzerland	2002	Prevalence of overweight and obesity in 6-12-year old children in Switzerland	National	both	6-12	6-12	1235	1196	1235	1196	
Switzerland	2004	The Swiss Conscription Database	National	both		18-20		20491		16987	
Switzerland	2005	The Swiss Conscription Database	National	both		18-20		32131		24432	
Switzerland	2005	Kinder- und Jugendsportstudie (KISS)	Subnational	both	6-13	6-13	256	238	256	238	
Switzerland	2005-2006	BMI Monitoring for Switzerland - Study 1	Community	urban	5-11	5-11	4103	4477	4103	4477	7
Switzerland	2005-2006	Chiolero et al., J Hypertens 25(11):2209-17, 2007	Subnational	both	10-14	10-14	2586	2621	2586	2621	
Switzerland	2006	The Swiss Conscription Database	National	both		18-20		34530		25845	
Switzerland	2006	Kinder- und Jugendsportstudie (KISS)	Subnational	both	7-14	7-14	102	98	102	98	
Switzerland	2006-2007	BMI Monitoring for Switzerland - Study 1	Community	urban	5-11	5-11	3896	4061	3896	4061	7
Switzerland	2007	The Swiss Conscription Database	National	both		18-20		36194		27229	
Switzerland	2007	Prevalence of overweight and obesity in 6-12-year old children in Switzerland	National	both	6-12	6-12	1136	1082	1136	1082	
Switzerland	2007-2008	BMI Monitoring for Switzerland - Study 1	Community	urban	5-11	5-11	4009	4067	4009	4067	7
Switzerland	2008	The Swiss Conscription Database	National	both		18-20		34497		25977	
Switzerland	2008-2009	BMI Monitoring for Switzerland - Study 1	Community	urban	5-11	5-11	3847	3998	3847	3998	7
Switzerland	2008-2010	BMI Monitoring for Switzerland - Study 2	Subnational	both	6	6	1110	1048	1110	1048	7
Switzerland	2009	The Swiss Conscription Database	National	both		18-20		34896		25811	
Switzerland	2009	Kinder- und Jugendsportstudie (KISS)	Subnational	both	10-17	10-17	63	43	63	43	
Switzerland	2009-2010	BMI Monitoring for Switzerland - Study 1	Community	urban	5-11	5-11	3913	4051	3913	4051	7
Switzerland	2010	The Swiss Conscription Database	National	both		18-20		33815		25308	
Switzerland	2007-2012	Bus Santé Study	Subnational	urban	20-80	20-80	57	60			
Switzerland	2010-2011	BMI Monitoring for Switzerland - Study 1	Community	urban	5-11	5-11	3734	4092	3734	4092	7
Switzerland	2011	The Swiss Conscription Database	National	both		18-20		34495		26133	
Switzerland	2011-2012	BMI Monitoring for Switzerland - Study 1	Community	urban	5-11	5-11	3940	4085	3940	4085	7
Switzerland	2010-2013	BMI Monitoring for Switzerland - Study 2	Subnational	both	5-11	5-11	9346	9735	9346	9735	7
Switzerland	2012	The Swiss Conscription Database	National	both		18-20		33590		25709	
Switzerland	2012	Prevalence of overweight and obesity in 6-12-year old children in Switzerland	National	both	6-12	6-12	1464	1499	1464	1499	
Switzerland	2012-2013	BMI Monitoring for Switzerland - Study 1	Community	urban	5-11	5-11	4042	4224	4042	4224	7
Switzerland	2013	The Swiss Conscription Database	National	both		18-20		32882		25309	
Switzerland	2013-2014	BMI Monitoring for Switzerland - Study 1	Community	urban	5-11	5-11	4073	4333	4073	4333	7
Switzerland	2014	The Swiss Conscription Database	National	both		18-20		32687		26517	
Switzerland	2014-2015	National Nutrition Survey menuCH	National	both	18-75	18-75	238	166	21	18	
Switzerland	2014-2015	BMI Monitoring for Switzerland - Study 1	Community	urban	5-11	5-11	4625	4657	4625	4657	7
Switzerland	2014-2016	BMI Monitoring for Switzerland - Study 2	Subnational	both	5-11	5-11	6130	6408	6130	6408	7
Switzerland	2015	The Swiss Conscription Database	National	both		18-20		32604		26547	
Switzerland	2013-2016	Bus Santé Study	Subnational	urban	20-74	20-74	331	306			
Switzerland	2015-2016	BMI Monitoring for Switzerland - Study 1	Community	urban	5-11	5-11	3058	3326	3058	3326	7
Switzerland	2016-2017	BMI Monitoring for Switzerland - Study 1	Community	urban	5-11	5-11	3121	3457	3121	3457	7
Switzerland	2017-2018	National Studie Gesundheit und Ernährung von Primarschülern (CHildHNS)	National	both	6-12	6-12	1144	1135	1144	1135	
Southwestern Europe											
Cyprus	2007-2008	Asthma Study Cyprus	National	both	15-18	15-18	490	368	490	368	
Cyprus	2007-2008	Childhood asthma and atopy in Cyprus	Subnational	both	7-9, 13-15	7-9, 13-15	590	561	587	559	
Cyprus	2007-2010	Identification and prevention of Dietary- and lifestyle-induced health EFfects In Children and infantS (IDEFICS)	Community	urban	5-9	5-9	1106	1129	1106	1129	
Cyprus	2015-2016	Childhood Obesity Surveillance Initiative 4	National	urban	6-9	6-9	623	687	623	685	
Cyprus	2019	Childhood Obesity Surveillance Initiative 5	National	both	6-10	6-10	994	1001	994	1001	
France	2000	Corpulence 7-9 ans	Subnational	both	7-9	7-9	796	786	796	786	
France	2005-2007	Etude individuelle nationale des consommations alimentaires 2 (INCA2), adults	National	both	18-79	18-79	270	179	37	28	
France	2005-2007	Etude individuelle nationale des consommations alimentaires 2 (INCA2), children and adolescents	National	both	5-17	5-17	715	640	684	607	
France	2007	Corpulence 7-9 ans	National	both	7-9	7-9	1244	1281	1244	1281	
France	2006-2007	Etude Nationale Nutrition Santé	National	both	5-74	5-74	876	819	730	726	

Country	Study years	Survey/study name/citation	Level of representativeness	Rural, urban or both	Age range as in NCD-RisC database *		Sample size (height)		Sample size (BMI)		Note
					Female	Male	Female	Male	Female	Male	
France	2006-2007	HELENA	Community	urban	12-17	12-17	165	122	165	122	
France	2012-2014	Cohorte des consultants des Centres d'examens de santé (CONSTANCES)	National	both	18-69	18-69	3079	2158	210	91	
France	2014-2016	Esteban	National	both	6-74	6-74	624	639	551	567	
France	2015-2017	Cohorte des consultants des Centres d'examens de santé (CONSTANCES)	National	both	18-69	18-69	7842	6005	683	454	
France	2015-2016	Childhood Obesity Surveillance Initiative 4	National	both	7-9	7-9	2561	2510	2561	2510	
France	2018-2019	Cohorte des consultants des Centres d'examens de santé (CONSTANCES)	National	both	18-69	18-69	2937	2274	161	133	
Greece	1997	The Didima Study	Community	rural	18+	18+	49	30	5		
Greece	1997	Greece Physical Fitness Study	National	both	8	8	29994	31586	29966	31567	
Greece	1998	Greece Physical Fitness Study	National	both	8-10	8-10	32049	33354	32026	33339	
Greece	1997-2000	Dietary criteria of adolescents: The role of physical activity, anthropometrics, dietetic, psychological and other factors	Community	urban	12-18	12-18	505	494	505	494	
Greece	1999	Greece Physical Fitness Study	National	both	8	8	28577	30083	28569	30067	
Greece	2000	Greece Physical Fitness Study	National	both	8	8	30384	32748	30109	32466	
Greece	2001	Greece Physical Fitness Study	National	both	8	8	30118	31667	29956	31504	
Greece	2003	National Epidemiological Survey	National	both	13-19	13-19	7778	6677	7777	6675	
Greece	2003	Greece Physical Fitness Study	National	both	8	8	31517	33599	31489	33570	
Greece	2004	Greece Physical Fitness Study	National	both	8	8	32004	33247	31989	33239	
Greece	2004-2005	Arsakeion School Study	Community	urban	6-18	6-18	420	358	420	358	
Greece	2005	Daphne	Community	rural	17-18	17-18	57	41	57	41	
Greece	2005	Greece Physical Fitness Study	National	both	9	9	32017	33245	31940	33197	
Greece	2006	Greece Physical Fitness Study	National	both	9	9	31940	33540	31930	33527	
Greece	2005-2006	Prevalence of hypertension and association of dietary mineral intake with blood pressure in healthy school children from Northern Greece aged 7-15 years	Community	urban	7-15	7-15	297	308	297	308	
Greece	2006	Samos	Community	both	5-13	5-13	67	53	65	55	
Greece	2007	Greece Physical Fitness Study	National	both	9	9	31809	33406	31780	33380	
Greece	2006-2007	HELENA, Athens	Community	urban	12-17	12-17	162	158	162	158	
Greece	2006-2007	HELENA, Heraklion	Community	urban	12-17	12-17	149	135	149	135	
Greece	2008	Greece Physical Fitness Study	National	rural	8	8	8080	8426	8077	8411	
Greece	2008	Greece Physical Fitness Study	National	urban	8	8	27294	28238	27247	28189	
Greece	2007-2009	Healthy Growth Study	Subnational	both	9-13	9-13	1291	1307	1291	1306	
Greece	2008-2009	Greek Childhood Obesity Study (GRECO)	National	both	10-12	10-12	2164	2046	2160	2033	
Greece	2010	EuropeaN Energy balance Research to prevent excessive weight Gain among Youth - The ENERGY-project	National	both	10-12	10-12	577	493	577	493	
Greece	2010-2012	ADONUT	National	both	12-19	12-19	18675	18669	18675	18668	
Greece	2010-2011	Childhood Obesity Surveillance Initiative 2	National	both	7-9	7-9	2688	2581	2688	2581	
Greece	2013	Childhood Obesity Surveillance Initiative 3	National	both	7-10	7-10	3908	3965	3908	3965	
Greece	2013-2015	Hellenic National Nutrition and Health Survey (HNNHS)	National	both	5+	5+	951	740	318	303	
Greece	2013-2016	National Survey of Morbidity and Risk Factors (EMENO)	National	both	18+	18+	238	214	36	30	
Greece	2014-2015	Evaluation of a web-based dietary intervention among primary school children (NUTRI-WEB Children project)	Community	both	7-12	7-12	434	387	433	387	
Greece	2015-2016	Childhood Obesity Surveillance Initiative 4	National	both	7-9	7-9	1867	1896	1866	1895	
Greece	2016-2017	Erasmus plus KA2, Healthyland	Community	rural	5	5	9	13	9	13	
Greece	2017-2018	Erasmus plus KA2, Healthyland	Community	rural	5-6	5-6	12	33	12	33	
Greece	2019	Childhood Obesity Surveillance Initiative 5	National	both	7-10	7-10	2014	2013	2014	2013	
Israel	1985-1989	Israeli Conscripts	National	both	16-19	16-19	383	1336	103608	147044	1
Israel	1990-1994	Israeli Conscripts	National	both	16-19	16-19	136928	188334	136928	188334	
Israel	1995-1999	Israeli Conscripts	National	both	16-19	16-19	144909	200216	144909	200216	
Israel	1999-2001	Mabat First Israeli National Health and Nutrition Survey	National	both	25-64	25-64	190	190			
Israel	2000-2004	Israeli Conscripts	National	both	16-19	16-19	151017	200485	151017	200485	
Israel	2003-2004	Mabat Youth First Israeli National Health and Nutrition Survey in 7th-12th grade students	National	both	12-18	12-18	3151	2569	3072	2553	
Israel	2002-2007	Hadera District Study	Subnational	urban	25-78	25-78	43	35			
Israel	2005-2009	Israeli Conscripts	National	both	16-19	16-19	145643	191850	145638	191846	
Israel	2010-2014	Israeli Conscripts	National	both	16-19	16-19	148489	212434	148487	212426	
Israel	2014-2016	Mabat Second Israeli National Health and Nutrition Survey	National	both	18-64	18-64	282	284	29	31	
Israel	2015-2016	Mabat Youth Second Israeli National Health and Nutrition Survey in 7th-12th grade students	National	both	12-18	12-18	2430	2256	2415	2221	
Israel	2015-2019	Israeli Conscripts	National	both	16-19	16-19	152147	198520	152147	198516	
Israel	2018-2019	Childhood Obesity Surveillance Initiative 5	National	both	6-7	6-7	73561	73724	73561	73724	
Italy	1980-1982	Po river delta epidemiological study - first survey	Community	rural	8-64	8-64	86	77			
Italy	1983-1985	Gubbio Study	Community	both	5+	5+	226	274			
Italy	1985-1988	Pisa epidemiological study - first survey	Community	urban	5-90	5-90	242	296			
Italy	1989	Ventimiglia Heart Study	Community	rural	6+	6+	124	119			
Italy	1988-1991	Po river delta epidemiological study - second survey	Community	rural	8-73	8-73	253	251	253	251	
Italy	1989-1992	Gubbio Study	Community	both	10+	10+	166	212	166	212	
Italy	1991-1993	Pisa epidemiological study - second survey	Community	urban	8-97	8-97	146	166	146	166	
Italy	1998-1999	Progetto VIP	Community	both	25-74	25-74	60	60			
Italy	2000	Sorveglianza Nutrizionale Infanzia e Adolescenza (SoNIA)	Subnational	both	13-15	13-15	244	236	244	236	
Italy	2000-2001	Sorveglianza Nutrizionale Infanzia e Adolescenza (SoNIA)	Subnational	both	8-9	8-9	413	444	413	444	
Italy	2003	Sorveglianza Nutrizionale Infanzia e Adolescenza (SoNIA)	Subnational	both	5-6	5-6	1327	1355	1327	1355	
Italy	2001-2007	Gubbio Study	Community	both	26+	26+	27	31			

Country	Study years	Survey/study name/citation	Level of representativeness	Rural, urban or both	Age range as in NCD-RisC database *		Sample size (height)		Sample size (BMI)		Note
					Female	Male	Female	Male	Female	Male	
Italy	2005-2007	Moli-family Study	Subnational	both	14+	14+	127	97	54	41	
Italy	2006-2007	HELENA	Community	urban	12-17	12-17	185	119	185	119	
Italy	2008	Childhood Obesity Surveillance Initiative 1	National	both	8-9	8-9	3788	4006	3788	4005	
Italy	2007-2010	Identification and prevention of Dietary- and lifestyle-induced health EFfects In Children and infantS (IDEFICS)	Community	urban	5-9	5-9	847	893	847	893	
Italy	2009	The ZOOM8 study: nutrition and physical activity of primary school children	National	both	6-11	6-11	1018	1059	1018	1059	
Italy	2008-2009	Progetto VIP	Community	both	25-74	25-74	49	46			
Italy	2009-2010	Grosso et al., J Epidemiol 24(4):327-33, 2014	Community	both	19+	19+	152	105	10	8	
Italy	2010	Childhood Obesity Surveillance Initiative 2	National	both	8-9	8-9	20011	21282	20007	21280	
Italy	2009-2011	Pisa epidemiological study - third survey	Community	urban	6+	6+	55	54	22	19	
Italy	2010-2012	CArdiovascular risk MEtabolic syndrome LIver and Autoimmunity diseases (CA.ME.LI.A)	Community	both	18-75	18-75	78	56	10	10	
Italy	2011	Grosso et al., Nutrients 5(12):4908-23, 2013	Community	both	13-16	13-16	508	627	508	627	
Italy	2011	CONVERGI Study	Community	urban	13-19	13-19	269	159	269	159	
Italy	2011-2012	ALImentazione e stile di vita negli ADOlescenti (Nutrition and lifestyle in adolescents)	Subnational	both	15-16	15-16	194	149	194	149	
Italy	2012	Childhood Obesity Surveillance Initiative 3	National	both	8-9	8-9	22188	22919	22186	22917	
Italy	2012-2014	Mistretta et al., Obes Res Clin Pract 11(2):215-226, 2017	Community	urban	11-16	11-16	758	885	753	878	
Italy	2014	OKkio alla SALUTE	National	both	8-9	8-9	22855	24462	22855	24457	
Italy	2014-2016	Mediterranean healthy Eating, Aging and Lifestyles (MEAL) study	Subnational	urban	20+	20+	188	114			
Italy	2015-2016	Childhood Obesity Surveillance Initiative 4	National	both	8-9	8-9	21457	22737	21454	22732	
Italy	2016	EVA Tyrol Study South Italy	Subnational	both	14-18	14-18	189	97	189	97	
Italy	2018-2019	Progetto VIP	Community	both	25-74	25-74	60	60			
Italy	2019	Childhood Obesity Surveillance Initiative 5	National	both	8-9	8-9	22469	23439	22462	23432	
Malta	2008	Childhood Obesity Surveillance Initiative 1	National	both	6	6	1031	1084	1031	1084	
Malta	2010	Childhood Obesity Surveillance Initiative 2	National	both	6	6	1170	1151	1170	1151	
Malta	2013	Childhood Obesity Surveillance Initiative 3	National	both	7-8	7-8	1692	1754	1692	1753	
Malta	2014-2016	SAHHTEK - The University of Malta Health and Wellbeing Study	National	both	18-70	18-70	120	76	5	5	
Malta	2015-2016	Childhood Obesity Surveillance Initiative 4	National	both	7-8	7-8	1906	2056	1905	2056	
Malta	2019	Childhood Obesity Surveillance Initiative 5	National	both	7-8	7-8	1998	2087	1998	2085	
Portugal	1980-1982	Growth of children from the countryside of Portugal	Community	both	6-15	6-15	424	414			
Portugal	1987	Body-Mass Index of Portuguese Conscripts	National	both		18-20				22582	1
Portugal	1988	Body-Mass Index of Portuguese Conscripts	National	both		18-20				22750	1
Portugal	1989	Body-Mass Index of Portuguese Conscripts	National	both		18-20		124		24259	1
Portugal	1990	Body-Mass Index of Portuguese Conscripts	National	both		18-20		207		207	
Portugal	1991	Body-Mass Index of Portuguese Conscripts	National	both		18-20		17673		16284	
Portugal	1992	Body-Mass Index of Portuguese Conscripts	National	both		18-20		57237		34264	
Portugal	1993	Body-Mass Index of Portuguese Conscripts	National	both		18-20		69414		56888	
Portugal	1994	Body-Mass Index of Portuguese Conscripts	National	both		18-20		63224		49229	
Portugal	1995	Body-Mass Index of Portuguese Conscripts	National	both		18-20		81168		62025	
Portugal	1996	Body-Mass Index of Portuguese Conscripts	National	both		18-21		121229		86907	
Portugal	1997	Body-Mass Index of Portuguese Conscripts	National	both		18-21		59790		49567	
Portugal	1998	Body-Mass Index of Portuguese Conscripts	National	both		18-21		40092		29805	
Portugal	1999	Body-Mass Index of Portuguese Conscripts	National	both		18-21		52572		41300	
Portugal	1998-2000	European Youth Heart Study	Community	both	9-16	9-16	535	554	535	554	
Portugal	2000	Body-Mass Index of Portuguese Conscripts	National	both		18-21		51440		40114	
Portugal	1999-2003	EPIPorto Study	Community	urban	18+	18+	126	86	10	5	
Portugal	2004	Growth of adolescents in Coimbra	Community	both	9-16	9-16	409	265	408	265	
Portugal	2003-2004	EPITeen - Epidemiological Health Investigation of Teenagers in Porto	Community	urban	13-14	13-14	1049	983	1048	981	
Portugal	2004	Growth of adolescents in Gouveia	Community	rural	10-19	10-19	246	238	246	238	
Portugal	2003-2005	Estudo de Prevalência da Obesidade e Consumos Alimentares em Portugal	National	both	18-64	18-64	1329	863	293	171	
Portugal	2007	Growth of adolescents in Tondela	Community	rural	6-19	6-19	312	315	312	314	
Portugal	2008	Azorean Physical Activity and Health Study II	Subnational	urban	15-18	15-18	893	608	893	608	
Portugal	2008	Childhood Obesity Surveillance Initiative 1	National	both	6-8	6-8	1792	1799	1792	1799	
Portugal	2007-2008	EPITeen - Epidemiological Health Investigation of Teenagers in Porto	Community	urban	16-17	16-17	1261	1192	1251	1186	
Portugal	2007-2008	European Youth Heart Study	Community	both	8-17	8-17	311	315	311	315	
Portugal	2007-2010	Promoção do Exercício e Saúde no Sedentarismo e Obesidade da Adolescência (PESSOA Program)	Community	urban	9-16	9-16	1813	1931	1813	1931	
Portugal	2007-2009	Portuguese National Survey of Physical Activity and Physical Fitness	National	both	9+	9+	9536	8853	8747	8123	
Portugal	2007-2008	Primary schools health promotion	Community	urban	6-12	6-12	238	224	238	224	
Portugal	2009	Bracara Study	Community	urban	8-14	8-14	336	398	336	398	
Portugal	2007-2010	The Midland Adolescent Lifestyle Study	Subnational	both	12-16	12-16	235	196	235	196	
Portugal	2009-2010	Portuguese Prevelance Study of Obesity in Childhood	National	both	5-10	5-10	6864	6543	6864	6541	
Portugal	2010	Childhood Obesity Surveillance Initiative 2	National	both	6-8	6-8	1688	1700	1688	1700	
Portugal	2010-2012	Promoção do Exercício e Saúde no Sedentarismo e Obesidade da Adolescência (PESSOA Program)	Community	urban	9-14	9-14	275	287	275	287	
Portugal	2008-2013	Preschool Physical Activity, Body Composition and Lifestyle Study (PRESTYLE)	Community	urban	5-6	5-6	635	703	607	651	
Portugal	2011-2012	The association of childhood obesity with asthma and rhinitis symptoms in 6-8years old children living in the Coimbra district, Portugal: the role of environmental, family and socioeconomic factors	Community	both	5-9	5-9	465	439	465	439	
Portugal	2011-2013	International Study of Childhood Obesity, Lifestyle and the Environment (ISCOLE)	Community	urban	9-11	9-11	419	358	419	358	
Portugal	2011-2013	Environmental Support for Leisure and Active Transport	Subnational	urban	10-15	10-15	340	294	340	294	

Country	Study years	Survey/study name/citation	Level of representativeness	Rural, urban or both	Age range as in NCD-RisC database *		Sample size (height)		Sample size (BMI)		Note
					Female	Male	Female	Male	Female	Male	
Portugal	2011-2013	EPITeen - Epidemiological Health Investigation of Teenagers in Porto	Community	urban	20-23	20-23	905	855			
Portugal	2013	Childhood Obesity Surveillance Initiative 3	National	both	6-8	6-8	2973	2946	2973	2946	
Portugal	2011-2014	Longitudinal Analysis of Biomarkers and Environmental Determinants of Physical activity (LABMED Study)	Subnational	urban	12-18	12-18	460	531	460	531	
Portugal	2013	Childhood obesity in Lousao	Community	rural	5-14	5-14	418	444	418	444	
Portugal	2013-2014	Cultural, social, economic, and environmental factors that can influence children's sport participation and obesity levels	Community	urban	6-10	6-10	408	385	408	385	
Portugal	2015	Inquérito Nacional de Saúde com Exame Físico (INSEF)	National	both	25-74	25-74	179	145			
Portugal	2015-2016	Childhood Obesity Surveillance Initiative 4	National	both	6-8	6-8	3358	3314	3358	3314	
Portugal	2016-2017	Overweight and obesity and their associated factors among early adolescence school children in urban and rural Portugal (rural only)	National	both	10-12	10-12	33	38	33	38	
Portugal	2016-2017	Portuguese Prevalence Study of Obesity in Childhood	Subnational	both	5-10	5-10	3223	3205	3223	3205	
Portugal	2018-2019	Childhood Obesity Surveillance Initiative 5	National	both	6-8	6-8	3452	3644	3429	3626	
Spain	1989	Cardiovascular Risk Factors Study in Catalonia	Subnational	both	15+	15+	22	17			
Spain	1991-1993	Encuesta de Factores de Riesgo Cardiovascular en la Región de Murcia (Cardiovascular Risk Factors Survey)	Subnational	both	18-69	18-69	11	9	11	9	
Spain	1994-1995	Encuesta de Nutrición y Salud Comunidad Valenciana 1994-95 (ENCV)	Subnational	urban	15+	15+	124	109	124	109	
Spain	1998-2000	EnKID study	National	both	5-24	5-24	1730	1452	1120	1030	
Spain	2001-2002	Catalan Health Interview Survey	Subnational	both	18-74	18-74	157	102	22	15	
Spain	2001-2003	DIabetes, Nutrición y Obesidad en la población adulta de la Región de Murcia (DINO)	Subnational	both	20+	20+	157	135			
Spain	2000-2005	CDC of the Canary Islands	Subnational	both	18-75	18-75	647	484	71	36	
Spain	2004	Cardiovascular Risk Study in Castilla y León (RECCyL)	Subnational	both	15+	15+	340	311	88	96	
Spain	2004	Vioque J et al., Obesity 16(3):664-70, 2008	Community	urban	24+	24+	13	12			
Spain	2006-2007	HELENA	Community	urban	12-17	12-17	193	188	193	188	
Spain	2006-2008	Biblioni Mdel et al., Br J Nutr 103(1):99-106, 2010	Community	both	43070	43070			652	571	
Spain	2007-2009	Harmonizing Equation of Risk in Mediterraneon countries EXtremadura (HERMEX)	Subnational	both	25-79	25-79	101	72			
Spain	2008-2010	Study on Nutrition and Cardiovascular Risk in Spain	National	both	18+	18+	1147	1001	164	147	
Spain	2007-2010	Identification and prevention of Dietary- and lifestyle-induced health EFfects In Children and infantS (IDEFICS)	Community	urban	5-9	5-9	468	474	468	474	
Spain	2009	Cardiovascular Risk Study in Castilla y León (RECCyL)	Subnational	both	20+	20+	128	105			
Spain	2010	EuropeaN Energy balance Research to prevent excessive weight Gain among Youth - The ENERGY-project	Subnational	urban	10-12	10-12	520	479	520	479	
Spain	2010-2011	ALimentación, Actividad Física, Desarrollo INfantil y Obesidad (ALADINO); Childhood Obesity Surveillance Initiative 2	National	both	6-9	6-9	3311	3343	3311	3341	
Spain	2012	Effects of a lifestyle intervention on the prevention of childhood obesity: a community-based model	Subnational	urban	8-12	8-12	1081	1171	1081	1171	
Spain	2013	ANIBES Study	National	both	9-75	9-75	343	443	188	301	
Spain	2012-2013	Brain Development and Air Pollution Ultrafine Particles in School Children-BREATHE Project	Subnational	urban	7-12	7-12	1325	1338	1325	1338	
Spain	2013	ALimentación, Actividad Física, Desarrollo INfantil y Obesidad (ALADINO); Childhood Obesity Surveillance Initiative 3	National	both	7-8	7-8	1673	1613	1673	1613	
Spain	2012-2013	INfancia y Medio Ambiente (Childhood and Environment) Project - Menorca	Subnational	both	14-15	14-15	165	162	165	162	
Spain	2012-2013	Infancia y Medio Ambiente (Childhood and Environment) Birth Cohort study - Sabadell	Subnational	urban	5-7	5-7	260	280	260	280	8
Spain	2012-2013	INfancia y Medio Ambiente (Childhood and Environment) Project - Valencia	Subnational	both	7-8	7-8	232	229	232	229	
Spain	2013-2015	INfancia y Medio Ambiente (Childhood and Environment) Project - Valencia	Subnational	both	8-9	8-9	199	210	199	210	
Spain	2013-2014	Effects of a lifestyle intervention on the prevention of childhood obesity: a community-based model	Subnational	urban	9-13	9-13	1004	1086	1004	1086	
Spain	2014	Cardiovascular Risk Study in Castilla y León (RECCyL)	Subnational	both	20+	20+	52	52			
Spain	2013-2016	Infancia y Medio Ambiente (Childhood and Environment) Birth Cohort study - Sabadell	Subnational	urban	7-10	7-10	236	253	236	253	
Spain	2015-2016	ALimentación, Actividad Física, Desarrollo INfantil y Obesidad (ALADINO); Childhood Obesity Surveillance Initiative 4	National	both	6-9	6-9	5367	5532	5367	5532	
Spain	2015-2016	INfancia y Medio Ambiente (Childhood and Environment) Project - Gipuzkoa	Subnational	both	7-8	7-8	195	195	195	195	
Spain	2015-2017	INfancia y Medio Ambiente (Childhood and Environment) Project - Valencia	Subnational	both	9-12	9-12	198	181	198	181	
Spain	2016-2018	Infancia y Medio Ambiente (Childhood and Environment) Birth Cohort study - Sabadell	Subnational	urban	9-12	9-12	239	256	239	256	
Spain	2018-2019	INfancia y Medio Ambiente (Childhood and Environment) Project - Gipuzkoa	Subnational	both	10-11	10-11	204	174	204	174	
Spain	2018	Childhood obesity cohort study of Sant Boi de Llobregat - SantBoiSà	Community	urban	5-10	5-10	266	299	266	299	
Spain	2019	ALimentación, Actividad Física, Desarrollo INfantil y Obesidad (ALADINO); Childhood Obesity Surveillance Initiative 5	National	both	6-9	6-9	8152	8513	8151	8512	
Spain	2019	Physical Activity, Sedentarism and Obesity of Spanish youth	National	both	5-19	5-19	1936	1836	1936	1836	
**Latin America and Caribbean**											
Andean Latin America											
Bolivia	1998	DHS	National	both	20-49		2094				
Bolivia	2003	DHS	National	both	15-49		9505		3625		
Bolivia	2005-2007	Cardiovascular and metabolic syndrome risk assessment of Bolivian school children and adolescents - Relationships to obesity, diabetes, income, food intake and physical activity	National	both	12-18	12-18	1849	1504	1841	1499	
Bolivia	2008	DHS	National	both	15-49		8826		3239		
Bolivia	2019	STEPS	National	both	18-69	18-69	636	405	105	65	
Ecuador	2004	Encuesta Demografica y de Salud Materno e Infantil/ Reproductive Health Survey	National	both	15-49		2443		308		
Ecuador	2004-2005	CArdiovascular Risk factors Multiple Evaluation in Latin America (CARMELA)	Community	urban	25-64	25-64	190	195			
Ecuador	2008-2009	Food Nutrition and Health	Community	both	10-16	10-16	375	382	374	379	
Ecuador	2011-2013	Encuesta Nacional de Salud y Nutrición (ENSANUT)	National	both	5-59	5-59	15100	14822	9292	10711	
Ecuador	2014-2015	Latin American Study of Nutrition and Health (ELANS)	National	urban	15-65	15-65	243	244	75	87	
Ecuador	2018	Encuesta Nacional de Salud y Nutrición (ENSANUT)	National	both	5+	5+	37486	34423	22970	23716	
Ecuador	2018	STEPS	National	both	18-69	18-69	634	509	109	115	
Peru	1996	DHS	National	both	20-49		5656				
Peru	2000	DHS	National	both	15-49		14126		5340		
Peru	2003	Factores de Riesgo de Enfermedades No Transmisibles	Community	urban	16+	16+	105	102	12	23	
Peru	2004	Factores de Riesgo de Enfermedades No Transmisibles	Community	urban	15+	15+	132	71	28	26	
Peru	2004-2006	DHS	National	both	15-49		3034		1175		

Country	Study years	Survey/study name/citation	Level of representativeness	Rural, urban or both	Age range as in NCD-RisC database *		Sample size (height)		Sample size (BMI)		Note
					Female	Male	Female	Male	Female	Male	
Peru	2005	Factores de Riesgo de Enfermedades No Transmisibles	Community	urban	15+	15+	160	78	42	24	
Peru	2004-2005	CArdiovascular Risk factors Multiple Evaluation in Latin America (CARMELA)	Community	urban	25-64	25-64	239	219			
Peru	2004-2005	Encuesta Nacional de Indicadores Nutricionales, Bioquímicos, Socioeconómicos y Culturales Relacionados con las Enfermedades Crónicas Degenerativas	National	both	20+	20+	549	474			
Peru	2006	Factores de Riesgo de Enfermedades No Transmisibles	Community	urban	15+	15+	320	256	98	87	
Peru	2007-2008	Monitoreo de Indicadores Nutricionales en la ENAHO 2007-2008	National	both	5+	5+	8670	8344	5807	5965	
Peru	2007-2008	DHS	National	both	15-49		10850		3940		
Peru	2007-2010	Monitoreo Nacional de Indicadores Nutricionales	National	both	12-49		1759		504		
Peru	2009	DHS	National	both	15-49		11951		4409		
Peru	2009-2011	Monitoreo de Indicadores Nutricionales en la ENAHO 2009-2010	National	both	5+	5+	15477	14729	10943	11184	
Peru	2010	DHS	National	both	15-49		11416		4358		
Peru	2010	Global School-based Student Health Survey	National	both	13-17	13-17	1306	1267	1306	1267	
Peru	2011	DHS	National	both	15-49		11180		4171		
Peru	2011-2012	Monitoreo de Indicadores Nutricionales en la ENAHO 2011	National	both	5+	5+	4006	3730	2820	2816	
Peru	2012	DHS	National	both	15-49		12002		4551		
Peru	2013	DHS	National	both	15+	15+	11280	850	4093	309	
Peru	2014	DHS	National	both	15+	15+	11558	3685	4176	1386	
Peru	2014	Launching a salt substitute to reduce blood pressure at the population level: a cluster randomized stepped wedge trial in Peru	Community	both	18+	18+	334	286	54	51	
Peru	2015	DHS	National	both	15+	15+	17724	4806	5190	1496	
Peru	2014-2015	Latin American Study of Nutrition and Health (ELANS)	National	urban	15-65	15-65	232	251	67	81	
Peru	2016	DHS	National	both	15+	15+	16141	4400	4674	1418	
Peru	2017	DHS	National	both	15+	15+	16855	4469	4743	1354	
Peru	2018	DHS	National	both	15+	15+	17282	4241	4807	1355	
Peru	2017-2018	Vigilancia Alimentario Nutricional por Etapas de Vida (VIANEV) 2017-2018	National	both	18-59	18-59	163	136	13	16	
Peru	2019	DHS	National	both	15+	15+	16403	4132	4732	1306	
Peru	2020	DHS	National	both	15+	15+	10982	2861	3442	1025	
Caribbean											
Antigua and Barbuda	2009	Global School-based Student Health Survey	National	both	13-17	13-17	122	70	122	70	
Bahamas	2011-2012	STEPS	National	both	25-64	25-64	132	111			
Bahamas	2013	Global School-based Student Health Survey	National	both	13-17	13-17	533	460	533	460	
Barbados	2011	Global School-based Student Health Survey	National	both	13-17	13-17	708	628	708	627	
Barbados	2011-2013	Health of the Nation (HotN)	National	both	25+	25+	42	24			
Belize	2004-2005	CAMDI	National	both	20+	20+	200	95			
Belize	2011	Global School-based Student Health Survey	National	both	13	13	188	163	188	163	
Belize	2017	Survey of Risk Factors for Chronic Kidney Disease (SRFCKD)	National	both	20-55	20-55	1436	910			
Cuba	1995	National Survey on Risk Factors and Chronic Diseases (NSRFCD)	National	urban	15+	15+	561	488	561	488	
Cuba	2001	National Survey on Risk Factors and Chronic Diseases (NSRFCD)	National	urban	15+	15+	2933	2777	881	903	
Cuba	2010	National Survey on Risk Factors and Chronic Diseases (NSRFCD)	National	both	15+	15+	782	786	255	244	
Cuba	2011	Non communicable disease risk factor in Cienfuegos	Community	urban	15-80	15-80	176	129	56	41	
Dominica	2007	STEPS	National	both	15-64	15-64	161	131	76	67	
Dominica	2009	Global School-based Student Health Survey	National	both	13-17	13-17	542	509	542	508	
Dominican Republic	1996	DHS	National	both	15-49		4494		1613		
Dominican Republic	1996-1998	Estudio factores de riesgo cardiovascular y sindrome metabolico en la Republica Dominicana I (EFRICARD I)	National	both	18-75	18-75	594	228	49	18	
Dominican Republic	2010-2012	Estudio factores de riesgo cardiovascular y sindrome metabolico en la Republica Dominicana II (EFRICARD II)	National	both	18-75	18-75	703	286	162	63	
Dominican Republic	2013	DHS	National	both	15-49	15-59	4967	4872	1767	1896	
Dominican Republic	2017	Prevalencia de hta y factores de riesgo en La Republica Dominicana al 2017 (ENPREFAR HAS 17)	National	both	18+	18+	327	335	46	72	
Grenada	2011	STEPS	National	both	25-64	25-64	103	77			
Guyana	2009	DHS	National	both	15-49	15-49	2392	1641	913	702	
Guyana	2010	Global School-based Student Health Survey	National	both	13-17	13-17	1261	988	1261	987	
Guyana	2016	Interaction between sex and rurality on the prevalence of diabetes in Guyana: a nationally representative study	National	both	18-69	18-69	137	69	21	16	
Guyana	2016	STEPS	National	both	18-69	18-69	448	273	72	54	
Haiti	2000	DHS	National	both	15-49		5696		2277		
Haiti	2005-2006	DHS	National	both	15-49		3227		1320		
Haiti	2012	DHS	National	both	15-49		5761		2266		
Haiti	2015-2016	Haiti Health Study (Carrefour)	Community	urban	25-65	25-65	196	149			
Haiti	2015-2016	Haiti Health Study (Thomonde)	Community	rural	25-65	25-65	67	45			
Haiti	2016-2017	DHS	National	both	15-49		5526		2169		
Jamaica	2000-2001	Jamaica Health and Lifestyle Survey	National	both	15-74	15-74	406	203	93	72	
Jamaica	2005	Jamaican Youth Risk and Resiliency Behaviour Survey 2005	National	both	10-15	10-15	1519	1379	1386	1328	
Jamaica	2006-2007	Jamaica Youth Risk and Resiliency Behaviour Survey 2006	National	both	15-19	15-19	701	585	701	585	
Jamaica	2007-2008	Jamaica Health and Lifestyle Survey	National	both	15-74	15-74	525	261	148	86	
Jamaica	2010	Global School-based Student Health Survey	National	both	13-17		788		787		
Jamaica	2016-2017	Jamaica Health and Lifestyle Survey	National	both	15+	15+	348	241	95	69	
Puerto Rico	2010-2013	HPV Infection in a Population-Based Sample of Puerto Rican Women	Subnational	both	16-64		118		22		
Saint Kitts and Nevis	2007	STEPS	Subnational	both	25-64	25-64	130	63			
Saint Kitts and Nevis	2011	Global School-based Student Health Survey	National	both	13-17	13-17	814	651	814	650	

Country	Study years	Survey/study name/citation	Level of representativeness	Rural, urban or both	Age range as in NCD-RisC database *		Sample size (height)		Sample size (BMI)		Note
					Female	Male	Female	Male	Female	Male	
Saint Lucia	2012	STEPS	National	both	25-64	25-64	127	82			
Saint Vincent and the Grenadines	2013-2014	STEPS	National	both	18-69	18-69	462	295	68	57	
Saint Vincent and the Grenadines	2018	Global School-based Student Health Survey	National	both	13-17	13-17	841	738	839	737	
Suriname	2013-2015	The Healthy Life in Suriname Study (HELISUR)	Subnational	urban	18-70	18-70	151	93	6	5	
Trinidad and Tobago	1999	Child Health Survey	National	both	5-9	5-9	3273	3064	3272	3060	
Trinidad and Tobago	2001	Adult Survey	National	rural	25+	25+	40	18			
Trinidad and Tobago	2003	Child Health Survey	National	both	5-9	5-9	1978	1833	1974	1832	
Trinidad and Tobago	2003	National Survey of Senior School Health	National	both	15-16	15-16	1113	830	1112	828	
Trinidad and Tobago	2011	STEPS	National	both	15-64	15-64	444	356	121	112	
Central Latin America											
Colombia	2000	DHS	National	both	20-49		1944				
Colombia	2002	The Santa Fe Study (Santa Fe)	Community	urban	15-69	15-69	269	168	86	60	
Colombia	2002	The Santa Fe Study (Tunjuelito)	Community	urban	15-29	15-29	312	208	127	93	
Colombia	2005	DHS	National	both	5-64	5-64	33673	27551	21521	20154	
Colombia	2005	Encuesta Nacional de Situacion Nutricional	National	both	5-49	5-12	5005	2646	3534	2644	
Colombia	2004-2005	CArdiovascular Risk factors Multiple Evaluation in Latin America (CARMELA)	Community	urban	25-64	25-64	182	169			
Colombia	2007	Encuesta Nacional de Salud	National	both	18-69	18-69	2489	1860	394	350	
Colombia	2010	DHS	National	both	5-64	5-64	43675	39428	28434	28359	
Colombia	2010	STEPS	Subnational	both	15-64	15-64	527	403	195	167	
Colombia	2011-2013	International Study of Childhood Obesity, Lifestyle and the Environment (ISCOLE)	Community	urban	9-11	9-11	462	454	462	454	
Colombia	2014-2015	Latin American Study of Nutrition and Health (ELANS)	National	urban	15-65	15-65	152	178	55	66	
Colombia	2015	Encuesta Nacional de Situacion Nutricional	National	both	5-64	5-64	2957	2769	1798	1848	
Colombia	2015	STEPS	Subnational	both	15-64	15-64	324	394	75	173	
Colombia	2018	COPEN: Estudio Colombiano de Perfiles Nutricionales	Subnational	urban	5-75	5-75	324	424	161	248	
Costa Rica	2004	CAMDI	Community	urban	20+	20+	162	75			
Costa Rica	2009	Global School-based Student Health Survey	National	both	13-17	13	1308	356	1308	356	
Costa Rica	2010	Costa Rican National Cardiovascular Risk Factors Survey, 2010	National	both	20+	20+	323	136			
Costa Rica	2014	Costa Rican National Cardiovascular Risk Factors Survey, 2014	National	both	20+	20+	391	159			
Costa Rica	2014-2015	Latin American Study of Nutrition and Health (ELANS)	National	urban	15-65	15-65	150	194	56	72	
El Salvador	2002-2003	Ecuesta Nacional de Salud Familiar	National	both	15-49		2677		381		
El Salvador	2004	CAMDI	Community	urban	20+	20+	173	107			
El Salvador	2008	Ecuesta Nacional de Salud Familiar	National	both	15-49		3798		942		
El Salvador	2013	Global School-based Student Health Survey	National	both	13-15		675		675		
El Salvador	2014-2015	Encuesta Nacional de Enfermedades Crónicas (ENECA-ELS)	National	both	20+	20+	749	399			
Guatemala	1998-1999	DHS	National	both	20-49		1368				
Guatemala	2002	Reproductive Health Survey	National	both	15-49	15-59	4466	1012	1305	387	
Guatemala	2001-2002	CAMDI	Community	urban	20+	20+	176	76			
Guatemala	2003-2005	The Institute of Nutrition of Central America and Panama Nutrition Supplementation Trial Cohort	Community	both	25-41	25-41	88	83			
Guatemala	2008-2009	Encuesta Nacional de Salud Materno Infantil	National	both	15-49	15-59	8722	3032	2632	1244	
Guatemala	2013	Sistema de vigilancia Epidemiológica de Salud y Nutrición (SIVESNU)	National	both	15-49		923		277		
Guatemala	2014-2015	DHS	National	both	15-49		13387		5398		
Guatemala	2015	Sistema de vigilancia Epidemiológica de Salud y Nutrición (SIVESNU)	National	both	15-49		781		239		
Guatemala	2015	STEPS	Subnational	urban	18+	18+	277	111	42	26	
Guatemala	2016	Sistema de vigilancia Epidemiológica de Salud y Nutrición (SIVESNU)	National	both	15-49		809		270		
Guatemala	2018	Assessing the food environment inside and around rural public schools in three villages of El Progreso, Guatemala. A pilot study.	Community	rural	6-15	6-15	192	202	192	202	
Guatemala	2017-2018	Sistema de vigilancia Epidemiológica de Salud y Nutrición (SIVESNU)	National	both	15-49		763		249		
Guatemala	2018-2019	Population-Based Survey of Chronic Kidney Disease in Guatemala	Community	rural	18+	18+	188	93	26	15	
Guatemala	2019	Evaluación del estado nutricional de escolares de nivel preprimario y primario en el marco de la ley de alimentación escolar	National	both	5-14	5-14	8981	9649	8979	9645	
Guatemala	2018-2019	Sistema de vigilancia Epidemiológica de Salud y Nutrición (SIVESNU)	National	both	15-49		777		243		
Honduras	1996	Honduras National Micronutrient Survey	National	both	20-40		240				
Honduras	2003-2004	CAMDI	Community	urban	20+	20+	216	117			
Honduras	2005-2006	DHS	National	both	15-49		11181		4132		
Honduras	2011-2012	DHS	National	both	15-49		12697		4802		
Mexico	1988-1989	Encuesta Nacional de Nutricion	National	both	12-49		5396		5828		1
Mexico	1998-1999	Encuesta Nacional de Nutricion	National	both	12-49		10194		5096		
Mexico	1999	National Survey on School Children	National	both	5-10	5-10	5040	4910	5031	4898	
Mexico	2000	Encuesta Nacional de Salud	National	both	10+	10+	19512	12635	10897	9122	
Mexico	2002	Encuesta Nacional Sobre Niveles de vida de los Hogares	National	both	5+	5+	7638	6738	5052	4771	
Mexico	2005	Encuesta Nacional Sobre Niveles de vida de los Hogares	National	both	5+	5+	7016	6480	4664	4634	
Mexico	2004-2005	CArdiovascular Risk factors Multiple Evaluation in Latin America (CARMELA)	Community	urban	25-64	25-64	213	207			
Mexico	2006	Encuesta Nacional de Salud y Nutrición	National	both	5+	5+	19604	17464	15038	14622	
Mexico	2006	PREVENIMSS National Coverage Surveys	National	urban	20+	20+	2265	1944			
Mexico	2010	PREVENIMSS National Coverage Surveys	National	urban	20+	20+	990	1381			
Mexico	2009-2012	Encuesta Nacional Sobre Niveles de vida de los Hogares	National	both	5+	5+	2371	2046	864	763	
Mexico	2011-2012	Encuesta Nacional de Salud y Nutrición	National	both	5+	5+	19978	18678	15145	15319	
Mexico	2016	Encuesta Nacional de Salud y Nutrición	National	both	5+	5+	4108	3331	2976	2815	

Country	Study years	Survey/study name/citation	Level of representativeness	Rural, urban or both	Age range as in NCD-RisC database *		Sample size (height)		Sample size (BMI)		Note
					Female	Male	Female	Male	Female	Male	
Mexico	2018-2019	Encuesta Nacional de Salud y Nutrición	National	both	5+	5+	7941	7422	5983	5989	
Nicaragua	1997-1998	DHS	National	both	15-49		7617		2966		
Nicaragua	2001	DHS	National	both	15-49		7292		2838		
Nicaragua	2003-2004	CAMDI	Community	urban	20+	20+	204	235			
Nicaragua	2006-2007	Encuesta Nicaraguense de Demografia y Salud	National	both	15-49		7317		2383		
Nicaragua	2011-2012	Encuesta Nicaraguense de Demografia y Salud	National	both	15-49		7451		2562		
Panama	2003	Encuesta de Niveles de Vida	National	both	5+	5+	5942	5983	3980	4154	
Panama	2010-2011	Prevalencia de factores de riesgo asociados a enfermedad cardiovascular 2010-2011	Subnational	both	18+	18+	549	183	98	29	
Panama	2018	Global School-based Student Health Survey	National	both	13-17	13-17	1385	1121	1384	1121	
Panama	2019	Encuesta Nacional de Salud de Panama (ENSPA)	National	both	15+	15+	3372	1027	714	413	
Venezuela	2004-2005	CArdiovascular Risk factors Multiple Evaluation in Latin America (CARMELA)	Community	urban	25-64	25-64	224	178			
Venezuela	2005-2006	Brajkovich et al., Rev Ven Endoc Metab 4(3):31-32, 2006	Community	urban	20-65	20-65	73	22			
Venezuela	2007-2008	Venezuelan Study of Metabolic Syndrome, Obesity and Lifestyle (VEMSOLS)	Community	urban	20+	20+	27	20			
Venezuela	2008-2009	Venezuelan Study of Metabolic Syndrome, Obesity and Lifestyle (VEMSOLS)	Community	rural	20+	20+	29	16			
Venezuela	2010-2011	Cardiometabolic risk factors in schoolchildren and adolescents of Mérida, Venezuela (CREDEFAR)	Community	urban	9-18	9-18	475	443	475	443	
Venezuela	2010-2011	Venezuelan Study of Metabolic Syndrome, Obesity and Lifestyle (VEMSOLS)	Community	urban	20+	20+	35	15			
Venezuela	2014-2015	Latin American Study of Nutrition and Health (ELANS)	National	urban	15-65	15-65	239	241	74	82	
Venezuela	2015-2017	Cardio-Metabolic Health Venezuelan Study (EVESCAM)	National	both	20+	20+	302	126			
Venezuela	2018-2020	Cardio-metabolic Health Venezuelan Study (EVESCAM) follow-up	National	both	22+	22+	45	21			
Southern Latin America											
Argentina	2005	Encuesta Nacional de Nutrición y Salud 2005	National	both	10-49		3939		2171		
Argentina	2004-2005	CArdiovascular Risk factors Multiple Evaluation in Latin America (CARMELA)	Community	urban	25-64	25-64	184	195			
Argentina	2008-2011	The VELA Project	Community	rural	5+	5+	361	281	291	246	
Argentina	2011	Primera Encuesta Alimentaria y Nutricional de la Ciudad Autónoma de Buenos Aires - EAN CABA	Community	urban	5-49; 60+	5-18; 60+	1332	982	1012	982	
Argentina	2012	Global School-based Student Health Survey	National	both	13-17	13-17	8906	8337	8904	8333	
Argentina	2012-2013	Primer estudio sobre el estado nutricional y los hábitos alimentarios de la población adulta de Rosario	Community	urban	18-70	18-70	312	129	54	17	
Argentina	2014-2015	Latin American Study of Nutrition and Health (ELANS)	National	urban	15-65	15-65	223	232	61	91	
Argentina	2018	Encuesta Nacional de Factores de Riesgo 2018	National	both	18+	18+	1931	1605	250	291	
Argentina	2018	Global School-based Student Health Survey	National	both	12-17	12-17	18979	16801	18935	16773	
Brazil	1989	Pesquisa Nacional sobre Saude e Nutricao	National	both	5+	5+	10258	10503	10832	11049	1
Brazil	1995	The 1982 Pelotas (Brazil) Birth Cohort: 13 years follow-up	Community	urban	13	13	363	352	363	352	
Brazil	1995-1996	Cohort study from Porto Alegre	Community	urban	18+	18+	148	151	26	22	
Brazil	1996-1997	Pesquisa sobre Padrões de Vida (PPV)	Subnational	both	5+	5+	4405	4207	2843	2901	
Brazil	1996	DHS	National	both	20-49		1817				
Brazil	1996-1997	The Bambui Cohort Study of Ageing	Community	urban	18+	18+	131	129	20	25	
Brazil	1997	The 1982 Pelotas (Brazil) Birth Cohort: 15 years follow-up	Community	urban	15	15	513	559	513	559	
Brazil	1998	Belo Horizonte Heart Study	Community	urban	6-18	6-18	738	658	738	658	
Brazil	2000	The 1982 Pelotas (Brazil) Birth Cohort: 18 years follow-up	Community	urban		18		2229		2228	
Brazil	1999-2000	Projeto Esporte Brasil	National	urban	6-11	6-11	102	107	102	107	
Brazil	1999-2000	Pelotas cross-sectional survey	Community	urban	20-69	20-69	220	229			
Brazil	1999-2000	Prevalence of Risk Factors for Coronary Artery Disease in the State of Rio Grande do Sul	Subnational	urban	20+	20+	118	125			
Brazil	2001	The 1982 Pelotas (Brazil) Birth Cohort: 19 years follow-up	Community	urban	19		920		919		
Brazil	2001	Projeto Esporte Brasil	National	urban	6-17	6-17	224	251	224	249	
Brazil	2002	Study of the prevalence of obesity in children and adolescents (Estudo da prevalência da obesidade em crianças e adolescentes (EPOCA))	Community	urban	7-10	7-10	1430	1504	1430	1504	
Brazil	2002-2003	Pesquisa de Orcamentos Familiares	National	both	5+	5+	41332	44862	27119	29276	
Brazil	2003	Projeto Esporte Brasil	National	both	6-17	6-17	2078	2022	2073	2015	
Brazil	2003	Women health in Southern Brazil	Community	urban	20-60		262				
Brazil	2004	Caju & Virgen das Gracas	Community	rural	18+	18+	77	73	14	15	
Brazil	2004	Projeto Esporte Brasil	National	both	6-17	6-17	12304	14509	12254	14447	
Brazil	2002-2004	1978-1979 Ribeira Preto Birth Cohort	Community	urban	22-25	22-25	1083	1013			
Brazil	2005	Prevalência e Fatores de Risco Cardiovascular em Crianças	Community	urban	7-12	7-12	720	777	719	776	
Brazil	2004-2006	Hearts of Brazil	National	urban	18+	18+	129	113	20	11	
Brazil	2004-2005	The 1993 Pelotas (Brazil) Birth Cohort: 11 years follow-up	Community	urban	10-12	10-12	2258	2184	2257	2184	
Brazil	2005	Projeto Esporte Brasil	National	both	6-17	6-17	3387	3743	3286	3615	
Brazil	2004-2005	1994 Ribeira Preto Birth Cohort	Community	urban	10-11	10-11	388	400	388	400	
Brazil	2004-2005	Schoolchildren's Health	Community	both	6-17	6-17	1245	1240	1245	1240	
Brazil	2005	Syndrome of Obesity and Risk Factors for Cardiovascular Disease Study	Community	urban	18-90	18-90	211	209	69	74	
Brazil	2004-2005	The 1982 Pelotas (Brazil) Birth Cohort: 23 years follow-up	Community	urban	23	23	2082	2207			
Brazil	2006	ATITUDE	Subnational	both	14-21	14-21	3454	2392	2459	1660	
Brazil	2006	The Ouro Preto Study	Community	urban	7-14	7-14	399	364	399	364	
Brazil	2006	Pesquisa Nacional de Demografia e Saude 2006	National	both	15-49		6916		2329		
Brazil	2006	Projeto Esporte Brasil	National	both	6-17	6-17	6220	7521	6211	7505	
Brazil	2005-2006	Sao Luis Birth Cohort	Community	urban	7-8	7-8	325	347	325	347	
Brazil	2007	Cardiovascular Disease Risk Factors in Caxias do Sul-RS, Brazil Adolescents	Community	urban	11-17	11-17	886	776	886	774	
Brazil	2007	Study of the prevalence of obesity in children and adolescents (Estudo da prevalência da obesidade em crianças e adolescentes (EPOCA))	Community	urban	7-10	7-10	624	603	624	603	

Country	Study years	Survey/study name/citation	Level of representativeness	Rural, urban or both	Age range as in NCD-RisC database *		Sample size (height)		Sample size (BMI)		Note
					Female	Male	Female	Male	Female	Male	
Brazil	2007	Prevalence of overweight and obesity in children from Medianeira, Paraná, Brazil	Community	urban	5-12	5-12	515	529	515	529	
Brazil	2007	Projeto Esporte Brasil	National	both	6-17	6-17	4218	4771	4214	4767	
Brazil	2006-2007	Syndrome of Obesity and Risk Factors for Cardiovascular Disease Study among Teenagers	Community	urban	11-18	11-18	238	231	236	230	
Brazil	2008	Caju & Virgen das Gracas	Community	rural	18+	18+	76	62	24	14	
Brazil	2007-2008	Christofaro et al., Scand J Med Sci Sports 23(3):317-22, 2013	Community	urban	10-16	10-16	528	493	528	493	
Brazil	2007-2008	Nutritional status of children in daycare center	Community	urban	5-7	5-7	81	83	81	83	
Brazil	2008	The 1993 Pelotas (Brazil) Birth Cohort: 15 years follow-up	Community	urban	14-15	14-15	2095	2001	2095	2001	
Brazil	2008	Projeto Esporte Brasil	National	both	6-17	6-17	2395	3511	2384	3474	
Brazil	2007-2009	Schoolchildren's Health	Community	both	6-17	6-17	742	837	742	837	
Brazil	2008-2010	Machado-Rodrigues et al., Ann Hum Biol 41(3): 271-6, 2013	Community	urban	10-17	10-17	507	376	507	376	
Brazil	2008-2009	Pesquisa de Orcamentos Familiares	National	both	5+	5+	42858	43488	25710	27098	
Brazil	2009	Projeto Esporte Brasil	National	both	6-17	6-17	781	969	765	942	
Brazil	2010	Longitudinal Study of Health and Wellbeing in Preschool Age (Project ELOS-Pré)	Community	urban	5	5	247	255	247	255	
Brazil	2010	Projeto Esporte Brasil	National	both	6-17	6-17	1049	1095	1045	1093	
Brazil	2010	San Pedro	Community	rural	18+	18+	48	37	10	8	
Brazil	2009-2010	EpiFloripa Adults Cohort Study	Community	urban	20-59	20-59	266	262			
Brazil	2011	ATITUDE	Subnational	both	14-19	14-19	3636	2416	3636	2415	
Brazil	2010-2011	The 2004 Pelotas (Brazil) Birth Cohort: 6 years follow-up	Community	urban	6-7	6-7	1740	1864	1631	1721	
Brazil	2011	Pregnancy in adolescence in municipalities of small size in the Northeast of Brazil	Community	both	13-19	13-19	563	512	563	512	
Brazil	2011	Projeto Esporte Brasil	National	both	6-17	6-17	933	1256	928	1239	
Brazil	2012	Anthropometric indices in Brazilian children: Colombo, Parana, Brazil	Community	both	6-11	6-11	1012	1021	1012	1021	
Brazil	2012	Longitudinal Study of Health and Wellbeing in Preschool Age (Project ELOS-Pré)	Community	urban	5-7	5-7	349	388	348	388	
Brazil	2011-2013	International Study of Childhood Obesity, Lifestyle and the Environment (ISCOLE)	Community	urban	9-11	9-11	297	287	287	277	
Brazil	2011-2012	The 1993 Pelotas (Brazil) Birth Cohort: 18 years follow-up	Community	urban	17-19	17-19	2004	1970	2004	1970	
Brazil	2012	Projeto Esporte Brasil	National	both	6-17	6-17	1989	3130	1973	3114	
Brazil	2011-2012	Schoolchildren's Health	Community	both	6-17	6-17	1052	891	1052	891	
Brazil	2012	EpiFloripa Adults Cohort Study	Community	urban	22-62	22-62	121	103			
Brazil	2010-2015	Baependi Heart Study	Community	rural	18+	18+	293	240	48	47	
Brazil	2012-2013	Study of the prevalence of obesity in children and adolescents (Estudo da prevalência da obesidade em crianças e adolescentes (EPOCA))	Community	urban	7-10	7-10	799	723	799	723	
Brazil	2012-2013	Evaluation of the realization of the human right to adequate food among public and private elementary school students in Maceió	Subnational	urban	9-11	9-11	702	640	701	639	
Brazil	2013	Pesquisas Nacional de Saude	National	both	18+	18+	7421	6060	1101	1005	
Brazil	2012-2013	Prevalence of Leptin Polymorphism Gln223Arg	Community	urban	18+	18+	137	71	24	15	
Brazil	2013	Projeto Esporte Brasil	National	urban	6-17	6-17	965	1270	960	1269	
Brazil	2012-2013	The 1982 Pelotas (Brazil) Birth Cohort: 30 years follow-up	Community	urban	30	30	1853	1754			
Brazil	2011-2014	Profile of Risk Factors for Coronary Arterial Disease in Rio Grade do Sul - Revaluation After 10 Years	Subnational	urban	20+	20+	63	60			
Brazil	2014	Brazilian Guide to the Physical Fitnees related to Health Assessment and Lifestyle Habits	Community	urban	14-19	14-19	538	473	535	473	
Brazil	2014	Longitudinal Study of Health and Wellbeing in Preschool Age (Project ELOS-Pré)	Community	urban	7-9	7-9	213	246	200	223	
Brazil	2013-2014	Estudo de Riscos Cardiovasculares em Adolescentes (ERICA)	National	both	12-17	12-17	40675	32724	40675	32723	
Brazil	2014	Projeto Esporte Brasil	National	both	6-17	6-17	210	265	210	265	
Brazil	2014-2015	Latin American Study of Nutrition and Health (ELANS)	National	urban	15-65	15-65	323	388	95	140	
Brazil	2015-2016	The Ouro Preto Study	Community	rural	18+	18+	46	27	7	10	
Brazil	2015	The 2004 Pelotas (Brazil) Birth Cohort: 11 years follow-up	Community	urban	10-11	10-11	1632	1736	1632	1736	
Brazil	2015	Projeto Esporte Brasil	National	urban	6-17	6-17	497	639	496	639	
Brazil	2014-2015	Schoolchildren's Health	Community	both	6-17	6-17	1398	1098	1397	1098	
Brazil	2014-2015	II Diagnóstico de Saúde da População Materno-Infantil do Estado de Alagoas	Subnational	both	19-49		947				
Brazil	2014-2015	EpiFloripa Adults Cohort Study	Community	urban	25-65	25-65	55	41			
Brazil	2016	ATITUDE	Subnational	both	14-21	14-21	3206	2570	3182	2553	
Brazil	2016	Projeto Esporte Brasil	National	urban	6-17	6-17	110	175	110	175	
Brazil	2015-2016	The 1993 Pelotas (Brazil) Birth Cohort: 22 years follow-up	Community	urban	21-23	21-23	1886	1703			
Brazil	2017	Projeto Esporte Brasil	National	both	6-17	6-17	1357	2735	1349	2733	
Brazil	2017	Intervention in physical education classes to reduce sedentary behavior and improve cognitive function: SACODE	Community	both	14-19	14-19	624	498	623	498	
Brazil	2016-2017	Schoolchildren's Health	Community	both	6-17	6-17	1424	1073	1424	1073	
Brazil	2016-2017	Study in Presidente Prudente	Community	urban	18+	18+	50	30	7	7	
Brazil	2018	Healthy Living study in Lagoa do Carro	Community	both	5-15	5-15	657	704	657	704	
Brazil	2018	Projeto Esporte Brasil	National	both	6-17	6-17	1160	1330	1151	1314	
Brazil	2018-2019	Study of the prevalence of obesity in children and adolescents (Estudo da prevalência da obesidade em crianças e adolescentes (EPOCA))	Community	urban	7-10	7-10	518	410	518	410	
Brazil	2019	Pesquisas Nacional de Saude	National	both	15+	15+	554	629	127	174	
Brazil	2019	Projeto Esporte Brasil	National	both	6-17	6-17	1396	1623	1355	1587	
Brazil	2018-2019	BP-SAMPA Project	Community	urban	10-17	10-17	1853	1329	1853	1328	
Brazil	2018-2019	Epidemiology in the health (Santo Anastácio Edition)	Community	urban	18+	18+	38	34	8	4	
Brazil	2020	Projeto Esporte Brasil	National	urban	6-17	6-17	153	334	153	332	
Chile	1988	Chilean Health Study	Subnational	urban	15+	15+	54	57			
Chile	1992-1993	Miquel et al., Gastroenterology 115(4):937-46, 1998	Community	urban	18+	18+	14	8	14	8	
Chile	2000	Nervi et al., J Hepatol 45(2):299-305, 2006	Community	urban	18+	18+	15	2			
Chile	2003	Encuesta Nacional de Salud	National	both	17+	17+	306	306	76	63	

Country	Study years	Survey/study name/citation	Level of representativeness	Rural, urban or both	Age range as in NCD-RisC database *		Sample size (height)		Sample size (BMI)		Note
					Female	Male	Female	Male	Female	Male	
Chile	2004-2005	CArdiovascular Risk factors Multiple Evaluation in Latin America (CARMELA)	Community	urban	25-64	25-64	202	182			
Chile	2009-2010	Encuesta Nacional de Salud	National	both	15+	15+	623	441	191	169	
Chile	2010-2011	Encuesta Nacional de Consumo Alimentario	National	both	5+	5+	978	837	631	591	
Chile	2013	Global School-based Student Health Survey	National	both	13-17	13-17	793	799	793	799	
Chile	2014-2015	Latin American Study of Nutrition and Health (ELANS)	National	urban	15-65	15-65	161	176	56	62	
Chile	2016-2017	Encuesta Nacional de Salud	National	both	15+	15+	631	453	183	159	
Paraguay	2011	Primera Encuesta Nacional de Factores de Riesgo de Enfermedades No Transmisibles en la Poblacion General	National	both	15-75	15-75	529	350	125	118	
Paraguay	2017	Global School-based Student Health Survey	National	both	12-16	12-16	1234	1119	1231	1119	
Uruguay	2004	CUiiDARTE Project	National	urban	5-8	5-8	149	129	149	129	
Uruguay	2005	CUiiDARTE Project	National	urban	6-10	6-10	105	105	105	105	
Uruguay	2006	STEPS	National	both	25-64	25-64	73	33			
Uruguay	2009-2010	CUiiDARTE Project	National	urban	10-14	10-14	130	117	129	117	
Uruguay	2012	Global School-based Student Health Survey	National	both	13-15		1377		1377		
Uruguay	2013	STEPS	National	urban	15-64	15-64	317	212	92	75	
Uruguay	2012-2016	Genotype, Phenotype and Environment of Hypertension in Uruguay (GEFA-HT-UY)	Community	urban	19+	19+	13	12			
Uruguay	2015	CUiiDARTE Project	National	urban	17-20	17-20	142	135	142	135	
Uruguay	2016-2017	CUiiDARTE Project	Community	urban	5-6	5-6	375	395	375	395	
Uruguay	2019	Global School-based Student Health Survey	National	both	13-16	13-16	1026	903	1022	901	
**Oceania**											
Melanesia											
Fiji	2002	STEPS	National	both	15-64	15-64	1366	954	412	321	
Fiji	2005-2007	Pacific Obesity Prevention in Communities - Healthy Youth Health Communities Study	Subnational	urban	11-19	11-19	4109	3730	4109	3730	
Fiji	2007-2008	Pacific Obesity Prevention in Communities - Healthy Youth Health Communities Study	Subnational	urban	13-22	13-22	1832	1492	1813	1479	
Fiji	2010	Global School-based Student Health Survey	National	both	13-17	13-17	892	630	892	629	
Fiji	2011	STEPS	National	both	25-64	25-64	162	123			
Fiji	2016	Global School-based Student Health Survey	National	both	13-17	13-17	1469	1394	1469	1394	
Papua New Guinea	2007	STEPS	National	both	15-64	15-64	638	555	211	194	
Solomon Islands	2004	A genetic-ecological study of the risk factors for lifestyle-related diseases in Oceanian populations	Community	both	18-79	18-79	58	50	5	7	
Solomon Islands	2006	STEPS	Subnational	both	15-64	15-64	637	480	146	116	
Solomon Islands	2009-2010	Furusawa et al., N Z Med J 124(1333):17-28, 2011	Subnational	both	5+	5+	222	172	155	148	
Solomon Islands	2015	STEPS	National	both	18-69	18-69	215	169	45	29	
Vanuatu	2005	STEPS	Subnational	both	15-60	15-60	382	316	122	104	
Vanuatu	2011	STEPS	National	both	25-64	25-64	431	384			
Polynesia and Micronesia											
American Samoa	1976-1978	McGarvey, Am J Clin Nutr 53(6 Suppl):1586S-1594S, 1991	National	both	5+	5+	15	14			
American Samoa	2004	STEPS	National	both	25-64	25-64	158	126			
Cook Islands	2003	STEPS	National	both	25-64	25-64	113	80			
Cook Islands	2011	Global School-based Student Health Survey	National	both	13-17	13-17	543	530	543	530	
Cook Islands	2013-2015	STEPS	National	both	18-64	18-64	119	77	23	10	
Cook Islands	2015	Global School-based Student Health Survey	National	both	13-17	13-17	313	305	313	304	
French Polynesia	2010	STEPS	National	both	18-64	18-64	524	396	92	69	
French Polynesia	2015	Global School-based Student Health Survey	National	both	12-17	12-17	1438	1310	1438	1310	
Kiribati	2004	STEPS	National	both	15-64	15-64	301	262	85	89	
Kiribati	2011	Global School-based Student Health Survey	National	both	13		156		156		
Kiribati	2015-2016	STEPS	National	both	18-69	18-69	211	177	11	15	
Marshall Islands	2002	STEPS	National	both	15-64	15-64	623	367	212	155	
Marshall Islands	2017-2018	STEPS	National	both	18+	18+	424	366	78	78	
Micronesia (Federated States of)	2002	STEPS	Subnational	both	25-64	25-64	149	86			
Micronesia (Federated States of)	2006	STEPS	Subnational	both	15-64	15-64	577	380	211	154	
Micronesia (Federated States of)	2008	STEPS	Subnational	both	25-64	25-64	205	119			
Micronesia (Federated States of)	2009	STEPS, Yap	Subnational	both	15-64	15-64	127	117	11	31	
Micronesia (Federated States of)	2009	STEPS, Kosrae	Subnational	both	15-64	15-64	64	31			
Micronesia (Federated States of)	2016	STEPS	Subnational	both	18-69	18-69	124	62	15	7	
Nauru	2004	STEPS	National	both	15-64	15-64	430	404	119	122	
Nauru	2006	STEPS	National	both	16-65	16-65	87	87	18	24	
Nauru	2011	Global School-based Student Health Survey	National	both	13-17		259		259		
Nauru	2015	STEPS	National	both	18-69	18-69	188	200	22	30	
Niue	2010	Global School-based Student Health Survey	National	both	13-17	13-17	38	63	38	63	
Niue	2011	STEPS	National	both	15+	15+	130	117	36	55	
Niue	2019	Global School-based Student Health Survey	National	both	12-17	12-17	65	51	64	51	
Palau	2011-2013	STEPS	National	both	25-64	25-64	90	88			
Palau	2016	STEPS	National	both	18+	18+	89	108	13	13	
Samoa	1979-1982	McGarvey, Am J Clin Nutr 53(6 Suppl):1586S-1594S, 1991	National	both	5+	5+	91	123			
Samoa	2002	STEPS	National	both	25-64	25-64	242	223			
Samoa	2010	Samoan Genome-Wide Association Study	National	both	24-65	24-65	250	182			9
Samoa	2013	STEPS	National	both	18-64	18-64	297	202	41	29	

Country	Study years	Survey/study name/citation	Level of representativeness	Rural, urban or both	Age range as in NCD-RisC database *		Sample size (height)		Sample size (BMI)		Note
					Female	Male	Female	Male	Female	Male	
Tokelau	2005	STEPS	National	both	15-64	15-64	105	101	43	44	
Tokelau	2014	STEPS	National	both	18-64	18-64	85	88	16	19	
Tonga	2004	STEPS	National	both	15-64	15-64	107	95	24	27	
Tonga	2005-2007	Pacific Obesity Prevention in Communities - Ma'alahi Youth Project	Subnational	rural	11-19	11-19	1445	1206	1445	1206	
Tonga	2007-2008	Pacific Obesity Prevention in Communities - Ma'alahi Youth Project	Subnational	rural	13-22	13-22	584	437	529	407	
Tonga	2010	Global School-based Student Health Survey	National	both	13-17	13-17	1069	927	1069	926	
Tonga	2011	STEPS	National	both	15-64	15-64	175	78			
Tonga	2017	Global School-based Student Health Survey	National	both	12-17	12-17	1452	1254	1452	1254	
Tonga	2017	STEPS	National	both	18-69	18-69	508	290	59	45	
Tuvalu	2013	Global School-based Student Health Survey	National	both	13-17	13-17	215	211	215	210	
Tuvalu	2015	STEPS	National	both	18-69	18-69	132	134	17	22	
**South Asia**											
Afghanistan	2013	National Nutrition Survey	National	both	10-49		13361		6443		
Afghanistan	2018	STEPS	National	both	18-69	18-69	638	692	145	139	
Bangladesh	1996-1997	DHS	National	both	20-49		2536				
Bangladesh	1999-2000	DHS	National	both	20-49		2852				
Bangladesh	2002	STEPS	National	both	25-64	25-64	982	912			
Bangladesh	2004	DHS	National	both	20-49		4187				
Bangladesh	2006	Urban Health Survey	Subnational	urban	20-59	20-59	2562	2349			
Bangladesh	2007	DHS	National	both	20-49		4060				
Bangladesh	2009-2010	STEPS	National	both	25+	25+	1139	588			
Bangladesh	2011	DHS	National	both	20+	15+	6837	858		15	
Bangladesh	2011-2012	Chronic Disease Risk Factor Study	Community	rural	14+	14+	187	116	60	47	
Bangladesh	2013	STEPS	National	both	25+	25+	495	279			
Bangladesh	2014	Global School-based Student Health Survey	National	both	11-13		447		447		
Bangladesh	2014	DHS	National	both	20-49		6441				
Bangladesh	2015-2016	An Assessment of BRAC Health Nutrition and Population Programme and Benchmark Survey of Sustainable Development Goal - 2015	National	rural	11+	35+	10262		4025		
Bangladesh	2018	STEPS	National	both	18-69	18-69	1079	765	162	118	
Bangladesh	2017-2018	DHS	National	both	15-49		6973				
Bangladesh	2018-2019	National Nutrition Surveillence	National	both	10+	10+	6521	6136	4849	4894	
Bhutan	2007	STEPS	Community	urban	25-74	25-74	152	119			
Bhutan	2014	STEPS	National	both	18-69	18-69	417	196	28	11	
Bhutan	2019	STEPS	National	both	15-69	15-69	844	482	125	103	
India	1975-1979	National Nutrition Monitoring Bureau rural survey	National	rural	5+	5+	3095	3831			
India	1980	National Nutrition Monitoring Bureau rural survey (Andhra Pradesh)	Subnational	rural	5-59	5-59	496	613			
India	1980	National Nutrition Monitoring Bureau rural survey (Gujarat)	Subnational	rural	5-59	5-59	129	145			
India	1980	National Nutrition Monitoring Bureau rural survey (Karnataka)	Subnational	rural	5-59	5-59	381	348			
India	1980	National Nutrition Monitoring Bureau rural survey (Kerala)	Subnational	rural	5-59	5-59	79	80			
India	1980	National Nutrition Monitoring Bureau rural survey (Orissa)	Subnational	rural	5-59	5-59	178	124			
India	1980	National Nutrition Monitoring Bureau rural survey (Tamil Nadu)	Subnational	rural	5-59	5-59	273	320			
India	1980	National Nutrition Monitoring Bureau rural survey (Uttar Pradesh)	Subnational	rural	5-59	5-59	176	233			
India	1980	National Nutrition Monitoring Bureau rural survey (West Bengal)	Subnational	rural	5-59	5-59	347	365			
India	1981	National Nutrition Monitoring Bureau rural survey (Andhra Pradesh)	Subnational	rural	5-59	5-59	346	436			
India	1981	National Nutrition Monitoring Bureau rural survey (Karnataka)	Subnational	rural	5-59	5-59	268	274			
India	1981	National Nutrition Monitoring Bureau rural survey (Maharashtra)	Subnational	rural	5-59	5-59	201	203			
India	1981	National Nutrition Monitoring Bureau rural survey (Orissa)	Subnational	rural	5-59	5-59	96	112			
India	1981	National Nutrition Monitoring Bureau rural survey (Tamil Nadu)	Subnational	rural	5-59	5-59	319	404			
India	1981	National Nutrition Monitoring Bureau rural survey (Uttar Pradesh)	Subnational	rural	5-59	5-59	129	187			
India	1981	National Nutrition Monitoring Bureau rural survey (West Bengal)	Subnational	rural	5-59	5-59	213	227			
India	1982	National Nutrition Monitoring Bureau rural survey (Andhra Pradesh)	Subnational	rural	5-59	5-59	230	297			
India	1982	National Nutrition Monitoring Bureau rural survey (Gujarat)	Subnational	rural	5-59	5-59	132	143			
India	1982	National Nutrition Monitoring Bureau rural survey (Karnataka)	Subnational	rural	5-59	5-59	262	273			
India	1982	National Nutrition Monitoring Bureau rural survey (Kerala)	Subnational	rural	5-59	5-59	140	137			
India	1982	National Nutrition Monitoring Bureau rural survey (Maharashtra)	Subnational	rural	5-59	5-59	293	387			
India	1982	National Nutrition Monitoring Bureau rural survey (Orissa)	Subnational	rural	5-59	5-59	108	102			
India	1982	National Nutrition Monitoring Bureau rural survey (Tamil Nadu)	Subnational	rural	5-59	5-59	337	380			
India	1982	National Nutrition Monitoring Bureau rural survey (West Bengal)	Subnational	rural	5-59	5-59	43	53			
India	1982-1983	Bengali School Children	Community	urban		7-21		514			
India	1983-1984	National Nutrition Monitoring Bureau rural survey (Andhra Pradesh)	Subnational	rural	5-59	5-59	554	486			
India	1983-1984	National Nutrition Monitoring Bureau rural survey (Gujarat)	Subnational	rural	5-59	5-59	618	641			
India	1983-1984	National Nutrition Monitoring Bureau rural survey (Orissa)	Subnational	rural	5-59	5-59	516	515			
India	1983-1984	National Nutrition Monitoring Bureau rural survey (Tamil Nadu)	Subnational	rural	5-59	5-59	565	586			
India	1990	National Nutrition Monitoring Bureau rural survey	National	rural	5+	5+	3464	3141	3464	3139	
India	1995-1996	Epidemiology of blood pressure across cross-cultural populations of Visakhapatnam district, Andhra Pradesh, India	Community	rural	19-76	19-76	54	47	2		
India	1996	Nutrition Profile of Indians (Amritsar District, Punjab)	Subnational	rural	5-59	5-59	407	342			
India	1996	Nutrition Profile of Indians (Bhatinda District, Punjab)	Subnational	rural	5-59	5-59	347	292			

Country	Study years	Survey/study name/citation	Level of representativeness	Rural, urban or both	Age range as in NCD-RisC database *		Sample size (height)		Sample size (BMI)		Note
					Female	Male	Female	Male	Female	Male	
India	1996	Nutrition Profile of Indians (Faridkot District, Punjab)	Subnational	rural	5-59	5-59	343	279			
India	1996	Nutrition Profile of Indians (Ferozpur District, Punjab)	Subnational	rural	5-59	5-59	417	291			
India	1996	Nutrition Profile of Indians (Gurdaspur District, Punjab)	Subnational	rural	5-59	5-59	395	360			
India	1996	Nutrition Profile of Indians (Hoshiarpur District, Punjab)	Subnational	rural	5-59	5-59	248	226			
India	1996	Nutrition Profile of Indians (Jind District, Haryana)	Subnational	rural	5-59	5-59	311	292			
India	1996	Nutrition Profile of Indians (Kaithal District, Haryana)	Subnational	rural	5-59	5-59	289	308			
India	1996	Nutrition Profile of Indians (Karnal District, Haryana)	Subnational	rural	5-59	5-59	220	134			
India	1996	Nutrition Profile of Indians (Kurkeshetra District, Haryana)	Subnational	rural	5-59	5-59	269	218			
India	1996	Nutrition Profile of Indians (Mahendragarh District, Haryana)	Subnational	rural	5-59	5-59	334	299			
India	1996-1997	National Nutrition Monitoring Bureau rural survey	National	rural	5+	5+	17589	13591	10790	10093	
India	1997	Ramachandran et al., Diabetes Res Clin Pract 44(3):207-13, 1999	Community	rural	20-74	20-74	385	264			
India	1996-1999	Chennai Urban Population Study	Community	urban	20+	20+	136	138			
India	1998-1999	DHS	National	both	20-49		31749				
India	2000	Ramachandran et al., Diabet Med 20(3):220-24, 2003	Subnational	urban	20-75	20-75	1299	1257			
India	1999-2001	Jaipur Heart Watch 2	Community	urban	20-75	20-75	88	91			
India	1998-2002	Vellore Birth Cohort	Subnational	both	25-31	25-31	963	1094			
India	1999-2002	Bengali School Children	Community	urban		7-21		1152		1152	
India	2000-2001	National Nutrition Monitoring Bureau rural survey	National	rural	5+	5+	14500	10281	8479	7615	
India	1999-2002	New Delhi Birth Cohort	Community	urban	26-33	26-33	457	633			
India	2001-2002	Nutrition Profile of Indians (Chamoli District, Uttar Pradesh)	Subnational	rural	5-59	5-59	432	350			
India	2001-2002	Nutrition Profile of Indians (Dehradun District, Uttar Pradesh)	Subnational	rural	5-59	5-59	421	321			
India	2001-2002	Nutrition Profile of Indians (Orissa)	Subnational	rural	5-59	5-59	13886	12019			
India	2001-2002	Nutrition Profile of Indians (Pauri District, Uttar Pradesh)	Subnational	rural	5-59	5-59	286	221			
India	2001-2002	Nutrition Profile of Indians (Tehrigarhwal District, Uttar Pradesh)	Subnational	rural	5-59	5-59	354	291			
India	2001-2002	Nutrition Profile of Indians (Uttarkashi District, Uttar Pradesh)	Subnational	rural	5-59	5-59	471	367			
India	2001-2002	Nutrition Profile of Indians (West Bengal)	Subnational	rural	5-59	5-59	6856	4460			
India	2002-2003	Blood pressure epidemiology in tribal, rural and urban communities of Orissa with special reference to physical and social parameters	Community	rural	18-80	18-80	61	56	9	7	
India	2001-2004	Chennai Urban Rural Epidemiology Study	Community	urban	20+	20+	327	237			
India	2003-2004	ICMR RF RHD Registry, Jai Vigyan Mission Mode, Kochi	Subnational	both	5-16	5-16	13515	11327	13515	11327	
India	2003-2005	STEPS, Ballabgarh	Subnational	both	15-69	15-69	883	841	187	283	
India	2003-2005	STEPS, Chennai	Subnational	both	15-69	15-69	787	788	212	252	
India	2003-2005	STEPS, Delhi	Subnational	urban	15-69	15-69	344	397	145	137	
India	2003-2005	STEPS, Dibrugarh	Subnational	both	15-69	15-69	901	769	201	218	
India	2003-2005	STEPS, Nagpur	Subnational	both	15-69	15-69	768	772	158	198	
India	2003-2005	STEPS, Trivandrum	Subnational	both	15-69	15-69	762	691	200	262	
India	2003-2004	Jaipur Heart Watch 3	Community	urban	20-75	20-75	39	32			
India	2004-2005	India Human Development Survey	National	both	8-11	8-11	6435	6999	6400	6948	10
India	2005-2006	DHS	National	both	15-49	15-54	66021	35133	22318	12515	
India	2005-2006	Risk factor profile for chronic non-communicable diseases: Results of a community-based study in Kerala, India	Community	both	15-64	15-64	870	796	209	273	
India	2005-2006	ICMR RF RHD Registry, Jai Vigyan Mission Mode, Kochi	Subnational	both	5-16	5-16	10509	9754	10509	9754	
India	2005-2006	National Nutrition Monitoring Bureau rural survey	National	rural	5+	5+	13320	10564	7648	7133	
India	2006	Ramachandran et al., Diabetes Care 31(5):893-98, 2008	Community	both	20+	20+	979	1085			
India	2005-2007	Prevalence of cardiovascular risk factors in rural Tamil Nadu	Community	rural	25-64	25-64	1090	1001			
India	2006-2007	Jaipur Heart Watch 4	Community	urban	20-75	20-75	135	79			
India	2006-2008	Kashmiri Young Adults	Subnational	both	20-40	20-40	445	916			
India	2005-2011	Bengali School Children	Community	urban	7-21	7-21	2183	847	1884	580	
India	2007-2008	Integrated Disease Surveillance Project Non-communicable Disease Risk Factors Survey, Andhra	Subnational	both	15-64	15-64	1173	798	239	221	
India	2007-2008	Integrated Disease Surveillance Project Non-communicable Disease Risk Factors Survey, Kerala	Subnational	both	15-64	15-64	747	459	159	194	
India	2007-2008	Integrated Disease Surveillance Project Non-communicable Disease Risk Factors Survey, Madhya	Subnational	both	15-64	15-64	992	1020	250	365	
India	2007-2008	Integrated Disease Surveillance Project Non-communicable Disease Risk Factors Survey, Maharashtra	Subnational	both	15-64	15-64	957	937	273	309	
India	2007-2008	Integrated Disease Surveillance Project Non-communicable Disease Risk Factors Survey, Mizoram	Subnational	both	15-64	15-64	971	846	264	257	
India	2007-2008	Integrated Disease Surveillance Project Non-communicable Disease Risk Factors Survey, Tamil	Subnational	both	15-64	15-64	978	572	191	177	
India	2007-2008	Integrated Disease Surveillance Project Non-communicable Disease Risk Factors Survey, Uttrakhand	Subnational	both	15-64	15-64	1199	769	336	319	
India	2007-2009	Prevalence of NCD risk factor in people above 15 year in rural area Nagpur using WHO STEP approach	Community	rural	15+	15+	609	694	256	262	
India	2008-2010	ICMR-India Diabetes (INDIAB) Study, Phase I	National	both	20+	20+	2033	1800			
India	2009-2010	Baseline Survey for the assessment of prevalence of risk factors of NCDs in Gandhinagar District	Community	both	15-64	15-64	524	598	146	224	
India	2009-2010	Jaipur Heart Watch 5	Community	urban	20-75	20-75	20	67			
India	2010	Kerala 2010 follow-up	Community	rural	21-70	21-70	32	41			
India	2011-2012	Body Mass Index, Social Conditions and Environmental Effect on High Blood Pressure among the Adolescent School Children	National	both	12-16	12-16	1161	1097	1161	1096	
India	2011-2012	India Human Development Survey	National	both	8-11	8-11	5783	6309	5758	6285	11
India	2011-2013	International Study of Childhood Obesity, Lifestyle and the Environment (ISCOLE)	Community	urban	9-11	9-11	328	292	328	292	
India	2011-2012	National Nutrition Monitoring Bureau rural survey	National	rural	5+	5+	21149	17159	12403	11621	
India	2012-2013	Health Survey in Anand School Children	Community	both	5-13	5-13	961	1630	959	1628	
India	2012-2013	District Level Household and Facility Survey (DLHS) 4	National	both	5+	5+	271681	253224	152769	157993	
India	2012-2013	ICMR-India Diabetes (INDIAB) Study, Phase II	Subnational	both	20+	20+	2461	1676			
India	2012-2014	Jaipur Heart Watch 6	Community	urban	20-75	20-75	56	91			

Country	Study years	Survey/study name/citation	Level of representativeness	Rural, urban or both	Age range as in NCD-RisC database *		Sample size (height)		Sample size (BMI)		Note
					Female	Male	Female	Male	Female	Male	
India	2014	Annual Health Survey: Clinical, Anthropometric and Bio-chemical	National	both	5+	5+	366551	361281	230014	245191	
India	2012-2015	ICMR-India Diabetes (INDIAB) Study, North East Phase	Subnational	both	20+	20+	3273	2209			
India	2015-2016	DHS	National	both	15-49	15-54	356491	50210	118744	18490	
India	2015-2016	Diet and nutritional status of urban population and prevalence of hypertension	National	urban	5+	5+	34512	30065	18822	19180	
India	2016-2017	Secular TRends in DiabEtes in India (STRiDE-I) -Change in Prevalence in Ten Years among Urban and Rural Populations in Tamil Nadu	Community	both	20+	20+	969	767			
India	2019-2021	DHS	National	both	15-49	15-54	346224	42508	114078	15572	
Nepal	1996	DHS	National	both	20-49		2216				
Nepal	2001	DHS	National	both	20-49		3290				
Nepal	2005	STEPS	Subnational	both	15-64	15-64	1311	1066	346	358	
Nepal	2006	DHS	National	both	15-49		6224		2283		
Nepal	2007-2008	STEPS	National	both	15-64	15-64	827	687	245	259	
Nepal	2006-2011	Early detection and management of Kidney disease, Hypertension, Diabetes and Cardiovascular disease (KHDC Nepal), Tarahara	Community	rural	18+	18+	661	280	97	48	
Nepal	2006-2011	Early detection and management of Kidney disease, Hypertension, Diabetes and Cardiovascular disease (KHDC Nepal), Damak	Community	urban	18+	18+	387	256	43	37	
Nepal	2006-2011	Early detection and management of Kidney disease, Hypertension, Diabetes and Cardiovascular disease (KHDC Nepal), Dharan	Community	urban	18+	18+	2083	1418	352	351	
Nepal	2011	DHS	National	both	15-49		3510		1288		
Nepal	2012-2013	STEPS	National	both	15-69	15-69	682	284	117	79	
Nepal	2015	Community based intervention for prevention and control of non-communicable diseases risk factors (CIPCON) baseline survey, Dhankuta	Subnational	rural	15-69	15-69	159	78	24	20	
Nepal	2015	Community based intervention for prevention and control of non-communicable diseases risk factors (CIPCON) baseline survey, Ilam	Subnational	rural	15-69	15-69	165	96	26	18	
Nepal	2016	DHS	National	both	15-49	15-49	3540	2109	1251	961	
Nepal	2016-2018	The Population Based Prevalence of Selected Non-Communicable Diseases In Nepal	National	both	20+	20+	1264	561			
Nepal	2019	STEPS	National	both	15-69	15-69	960	449	205	138	
Pakistan	1990-1994	National Health Survey of Pakistan 1990-1994	National	both	5+	5+	3480	3745	3391	3636	
Pakistan	1990-1994	MHS	Community	urban	18+	18+			31	39	
Pakistan	2005	STEPS	National	both	25-65	25-65	252	170			
Pakistan	2011	National Nutrition Survey	National	both	5-49	5-49	34697	17373	20335	14906	
Pakistan	2012-2013	DHS	National	both	20-49		1645				
Pakistan	2014	STEPS	Subnational	both	18-69	18-69	1402	957	264	191	
Pakistan	2016-2017	National Diabetes Survey of Pakistan	National	both	20+	20+	753	654			
Pakistan	2018-2019	National Nutrition Survey	National	both	5-49	5-19	79287	11905	38605	11888	
Pakistan	2017-2018	DHS	National	both	15-49		1978				
Sri Lanka	2006	STEPS	National	both	15-64	15-64	1857	1822	519	688	
Sri Lanka	2014	STEPS	National	both	18-69	18-69	447	260	70	57	
Sri Lanka	2016	Global School-based Student Health Survey	National	both	13-17	13-17	928	671	923	671	
**Sub-Saharan Africa**											
Central Africa											
Angola	2013-2014	CardioBengo - Population based cardiovascular longitudinal study in Bengo Province, Angola	Community	both	14-65	14-65	663	479	200	178	
Central African Republic	2010	STEPS	Subnational	both	25-64	25-64	540	390			
Central African Republic	2017	STEPS	Subnational	both	25-64	25-64	467	292			
Congo	1986	Enquête Brazzaville 1986	Community	urban	5-50	5-50	122	129			
Congo	1987	Enquête Nationale Congo 1987	National	rural	13-49		35		252		1
Congo	1991	Enquête Brazzaville 1991	Community	urban	5-90	5-90	1531	1311	1516	1309	
Congo	1996	Enquête Brazzaville 1996	Community	urban	5-90	5-90	2372	1716	1455	1283	
Congo	2004	STEPS	Community	urban	25-64	25-64	303	276			
Congo	2005	DHS	National	both	15-49		4177		1388		
Congo	2011-2012	DHS	National	both	15-49		3121		1031		
DR Congo	2001	Multiple Indicator Cluster Survey 2	National	both	15-49		2977		464		
DR Congo	2005	STEPS	Subnational	urban	15+	15+	557	362	204	150	
DR Congo	2007	DHS	National	both	15-49		2901		859		
DR Congo	2007	Diabetes and intermediate hyperglycaemia in Kisantu, DR Congo: a cross-sectional prevalence study	Community	urban	20+	20+	149	70			
DR Congo	2008	Visite de la Tension Artérielle et des Facteurs de Risque Associés en Afrique subsaharienne (VITARAA) - Kinshasa, RD Congo	Community	urban	10+	10+	542	510	259	257	
DR Congo	2008	Visite de la Tension Artérielle et des Facteurs de Risque Associés en Afrique subsaharienne (VITARAA) - Sud-Kivu, RD Congo	Community	both	20+	20+	146	81			
DR Congo	2013-2014	DHS	National	both	15-49		5544		1791		
DR Congo	2016-2017	Prevalence and Risk Factors of CKD in South Kivu, Democratic Republic of Congo: A Large-Scale Population Study	Subnational	both	18+	18+	272	165	56	22	
Gabon	2000	DHS	National	both	20-49		1385				
Gabon	2009	STEPS	Subnational	urban	15-64	15-64	695	321	157	77	
Gabon	2012	DHS	National	both	15-49		3131		1123		
East Africa											
Burundi	2010	DHS	National	both	15-49		2906		1143		
Burundi	2016-2017	DHS	National	both	15-49		5079		1922		
Comoros	1996	DHS	National	both	20-49		431				
Comoros	2011	STEPS	National	both	25-64	25-64	837	299			
Comoros	2012	DHS	National	both	15-49		3113		1207		
Eritrea	1995	DHS	National	both	15-49		453				
Eritrea	2002	DHS	National	both	15-49		1784				
Eritrea	2004	STEPS	National	both	15-64	15-64	375	356	123	139	

Country	Study years	Survey/study name/citation	Level of representativeness	Rural, urban or both	Age range as in NCD-RisC database *		Sample size (height)		Sample size (BMI)		Note
					Female	Male	Female	Male	Female	Male	
Eritrea	2010	STEPS	National	both	25-74	25-74	911	147			
Ethiopia	2000	DHS	National	both	15-49		9051		3384		
Ethiopia	2005	DHS	National	both	15-49		4018		1486		
Ethiopia	2006	STEPS	Subnational	urban	25-64	25-64	372	369			
Ethiopia	2011	DHS	National	both	15-49	15-59	9981	7484	3676	2821	
Ethiopia	2015	STEPS	National	both	15-69	15-69	2239	1422	580	416	
Ethiopia	2016	DHS	National	both	15-49	15-59	8919	6283	3226	2446	
Ethiopia	2018	Sustainable Urban Diets (SUDS) Addis Ababa	Community	urban	18-49		549		13		
Ethiopia	2018	Sustainable Urban Diets (SUDS) Kersa	Community	rural	18-49		369		7		
Kenya	1998	DHS	National	both	20-49		1995				
Kenya	2003	DHS	National	both	15-49		4641		1614		
Kenya	2008-2009	DHS	National	both	15-49		4949		1674		
Kenya	2011-2013	International Study of Childhood Obesity, Lifestyle and the Environment (ISCOLE)	Community	urban	9-11	9-11	301	262	301	262	
Kenya	2014	DHS	National	both	15-49		8090		2660		
Kenya	2015	STEPS	National	both	18-69	18-69	839	544	115	75	
Kenya	2018	Assessing the gaps in healthcare and determining the feasibility for the setup of a social enterprise - Viwandani Lown Community Health Center, Kenya	Community	urban	19-73	19-73	109	97	58	54	
Madagascar	1997	DHS	National	both	20-49		1512				
Madagascar	2003-2004	DHS	National	both	15-49		4211		1369		
Madagascar	2005	STEPS	Subnational	both	25-64	25-64	584	581			
Madagascar	2008-2009	DHS	National	both	15-49		4828		1833		
Malawi	2000	DHS	National	both	15-49		8139		2557		
Malawi	2004	DHS	National	both	15-49		6999		2000		
Malawi	2009	STEPS	National	both	25-64	25-64	955	426			
Malawi	2010	DHS	National	both	15-49		4711		1599		
Malawi	2013-2017	NCD Survey Malawi Epidemiology and Intervention Research Unit	Community	both	18+	18+	8582	5207	1545	1149	
Malawi	2015-2016	DHS	National	both	15-49		4767		1569		
Malawi	2017	STEPS	National	both	18-69	18-69	815	449	114	71	
Mauritius	1998	Mauritius Noncommunicable Disease Survey	National	both	25-74	25-74	174	99			
Mauritius	2009	Mauritius Noncommunicable Disease Survey	National	both	19-74	19-74	467	390			
Mauritius	2011	Global School-based Student Health Survey	National	both	13-17	13-17	1043	859	1043	859	
Mauritius	2011	Global School-based Student Health Survey-Rodrigues	Subnational	both	13-17	13-17	546	425	546	425	
Mauritius	2015	Mauritius Noncommunicable Disease Survey	National	both	20-74	20-74	255	217			
Mauritius	2017	Global School-based Student Health Survey	National	both	13-17	13-17	1526	1358	1525	1358	
Mauritius	2019	Global School-based Student Health Survey	Subnational	rural	12-17	12-17	1289	1116	1289	1116	
Mozambique	1997	DHS	National	both	20-49		1915				
Mozambique	2000	Growth of adolescents in Mozambique	Community	urban	9-17	9-17	727	690	727	690	
Mozambique	2003	DHS	National	both	15-49		6878		2207		
Mozambique	2005	STEPS	National	both	25-64	25-64	417	267			
Mozambique	2011	DHS	National	both	15-49		7796		2708		
Mozambique	2014-2015	STEPS	National	both	15-64	15-64	711	487	226	173	
Mozambique	2017-2018	Examining lifestyle behaviours and weight status of primary schoolchildren in Mozambique	Community	both	9-11	9-11	328	286	328	286	
Rwanda	2000	DHS	National	both	15-49		6107		2543		
Rwanda	2005	DHS	National	both	15-49		3286		1300		
Rwanda	2010	DHS	National	both	15-49	15-59	4288	3722	1564	1459	
Rwanda	2012	STEPS	National	both	15-64	15-64	1646	1001	414	264	
Rwanda	2014-2015	DHS	National	both	15-49	15-59	3868	3319	1388	1306	
Rwanda	2019-2020	DHS	National	both	15-49		3830		1647		
Seychelles	1998	School Screening Program	National	both	5-16	5-16	1423	1564	1423	1564	
Seychelles	1999	School Screening Program	National	both	5-16	5-16	2780	2657	2780	2657	
Seychelles	2000	School Screening Program	National	both	5-16	5-16	1829	1778	1829	1778	
Seychelles	2001	School Screening Program	National	both	5-16	5-16	2594	2622	2594	2622	
Seychelles	2002	School Screening Program	National	both	5-16	5-16	2533	2495	2533	2495	
Seychelles	2003	School Screening Program	National	both	5-16	5-16	2832	2814	2832	2814	
Seychelles	2004	School Screening Program	National	both	5-16	5-16	2316	2359	2316	2359	
Seychelles	2004	Seychelles Heart Survey III	National	both	25-64	25-64	70	55			
Seychelles	2005	School Screening Program	National	both	5-16	5-16	2772	2737	2771	2737	
Seychelles	2006	School Screening Program	National	both	5-16	5-16	2621	2692	2621	2691	
Seychelles	2007	Global School-based Student Health Survey	National	both	13-17	13-17	467	387	467	385	
Seychelles	2011	School Screening Program	National	both	5-16	5-16	2272	2300	2271	2298	
Seychelles	2012	School Screening Program	National	both	5-16	5-16	2338	2300	2337	2300	
Seychelles	2013	School Screening Program	National	both	5-16	5-16	2091	1942	2090	1942	
Seychelles	2014	School Screening Program	National	both	5-16	5-16	2219	2145	2219	2144	
Seychelles	2013-2014	Seychelles Heart Survey IV	National	both	25-64	25-64	70	45			
Seychelles	2015	Global School-based Student Health Survey	National	both	13-17	13-17	888	770	888	770	
Seychelles	2015	School Screening Program	National	both	5-16	5-16	2001	1994	2001	1994	

Country	Study years	Survey/study name/citation	Level of representativeness	Rural, urban or both	Age range as in NCD-RisC database *		Sample size (height)		Sample size (BMI)		Note
					Female	Male	Female	Male	Female	Male	
Seychelles	2016	School Screening Program	National	both	5-16	5-16	1893	1780	1892	1778	
Seychelles	2017	School Screening Program	National	both	5-16	5-16	1583	1521	1583	1521	
Seychelles	2018	School Screening Program	National	both	8-16	8-16	1715	1752	1715	1752	
Seychelles	2019	School Screening Program	National	both	8-16	8-16	1672	1624	1671	1623	
Seychelles	2020	School Screening Program	National	both	8-16	8-16	1506	1398	1506	1396	
Somalia	2016	The prevalence of selected risk factors for non-communicable diseases in Hargeisa, Somaliland: a cross-sectional study	Community	urban	20-69	20-69	301	55			
South Sudan	2017	Prevalence of hypertension and associated cardiovascular risk factors among adults aged 18-69 years in Juba City, South Sudan	Community	urban	18-69	18-69	113	88	9	4	
Sudan	2005	STEPS	Subnational	both	25-64	25-64	265	166			
Sudan	2016	STEPS	National	both	18-69	18-69	1448	704	236	155	
Sudan	2018	Prevalence and associated factors of hypertension among adults in Gadarif in eastern Sudan: a community-based study	Community	urban	18+	18+	72	35			
Tanzania	1996	DHS	National	both	20-49		2191				
Tanzania	1998-1999	Bovet et al., Int J Epidemiol 31(1):240-7, 2002	Community	urban	25-64	25-64	2459	1176			
Tanzania	2004-2005	DHS	National	both	15-49		6028		2096		
Tanzania	2009	Ilembula School Study	Community	rural	8-18	8-18	450	449	450	449	
Tanzania	2010	DHS	National	both	15-49		5642		2054		
Tanzania	2011	STEPS	Subnational	both	25-64	25-64	322	193			
Tanzania	2012	STEPS	National	both	25-64	25-64	569	412			
Tanzania	2015-2016	DHS	National	both	15-49		7463		2694		
Uganda	2000-2001	DHS	National	both	15-49		4152		1370		
Uganda	2006	DHS	National	both	15-49	15-54	1727	1300	589	565	
Uganda	2011	DHS	National	both	15-49	15-54	1693	1292	600	565	
Uganda	2012	Prevalence, awareness and control of hypertension in Uganda	Subnational	both	15+	15+	1394	722	385	255	
Uganda	2011-2013	Gulu Health and Demographic Surveillance Site (HDSS)	Community	rural	5+	5+	3295	2847	2392	2328	
Uganda	2011-2012	The Prevalence and Distribution of Non-communicable Diseases and their Risk Factors in Kasese District, Uganda	Subnational	both	25-79	25-79	34	40			
Uganda	2014	STEPS	National	both	18-69	18-69	793	641	160	124	
Uganda	2014-2015	Gulu Health and Demographic Surveillance Site (HDSS)	Community	rural	15-24	15-24	559	671	331	448	
Uganda	2016	DHS	National	both	15-54	15-54	3625	2880	1285	1241	
Uganda	2018-2019	Scaling up Packages of Intervention for Cardiovascular disease prevention in selected sites in Europe and Sub-Saharan Africa (SPICES)	Subnational	both	25-70	25-70	596	353			
Zambia	1996	DHS	National	both	20-49		2394				
Zambia	2001-2002	DHS	National	both	15-49		4739		1616		
Zambia	2007	DHS	National	both	15-49		4407		1447		
Zambia	2008	STEPS	Subnational	urban	25+	25+	412	207			
Zambia	2013-2014	DHS	National	both	15-49		9429		3401		
Zambia	2017	STEPS	National	both	18-69	18-69	883	584	207	109	
Southern Africa											
Botswana	2007	STEPS	National	both	25-64	25-64	666	363			
Botswana	2014	STEPS	National	both	15-69	15-69	932	554	206	126	
Eswatini	2006-2007	DHS	National	both	15-49	15-49	3025	2726	1197	1249	
Eswatini	2014	STEPS	National	both	15-69	15-69	738	509	231	204	
Lesotho	2004-2005	DHS	National	both	15-49		2022		803		
Lesotho	2009-2010	DHS	National	both	15-49	15-59	2368	1878	926	821	
Lesotho	2012	STEPS	National	both	25-64	25-64	275	122			
Lesotho	2014	DHS	National	both	15-49	15-59	1996	1583	760	678	
Namibia	2005	STEPS	National	both	25-64	25-64	310	242			
Namibia	2006-2007	DHS	National	both	15-49		5497		2047		
Namibia	2009	Okambilimbili Survey	Community	urban	5+	5+	550	490	295	265	
Namibia	2013	DHS	National	both	15-64		2501		841		
South Africa	1996	Ellisras Longitudinal Study	Community	rural	5-10	5-10	535	584	535	583	
South Africa	1997	Ellisras Longitudinal Study	Community	rural	5-11	5-11	968	1046	968	1046	
South Africa	1998	DHS	National	both	15+	15+	2900	2424	1048	1066	
South Africa	1998	Ellisras Longitudinal Study	Community	rural	5-12	5-12	856	958	856	958	
South Africa	1999	Ellisras Longitudinal Study	Community	rural	5-13	5-13	917	991	917	991	
South Africa	2000	Ellisras Longitudinal Study	Community	rural	5-14	5-14	877	936	877	936	
South Africa	2001	Ellisras Longitudinal Study	Community	rural	6-15	6-15	904	962	904	962	
South Africa	2000-2001	Transition and Health during Urbanisation of South Africans: Children	Subnational	both	9-15	9-15	640	606	639	606	
South Africa	2002	Ellisras Longitudinal Study	Community	rural	7-16	7-16	823	890	823	890	
South Africa	2002	The 1st South African National Youth Risk Behaviour Survey	National	both	14-18	14-18			4139	3609	
South Africa	2003	DHS	National	both	15+	15+	1711	1433	648	634	
South Africa	2003	Ellisras Longitudinal Study	Community	rural	8-17	8-17	858	911	858	911	
South Africa	2003-2004	Africa Centre Biomeasure Survey	Community	rural	25-49	25-49	355	176			
South Africa	2008	National Income Dynamics Study Wave I	National	both	5+	5+	5873	5251	3748	3804	
South Africa	2007-2008	Cardiometabolic risk profile of South African Learners	Subnational	both	10-16	10-16	776	496	776	496	
South Africa	2008	The 2nd South African National Youth Risk Behaviour Survey	National	both	14-18	14-18			4201	3910	
South Africa	2010	Africa Centre Biomeasure Survey	Community	rural	15+	15+	2782	1836	1045	1022	
South Africa	2010-2011	National Income Dynamics Study Wave II	National	both	5+	5+	6051	5440	3681	3575	
South Africa	2011-2013	International Study of Childhood Obesity, Lifestyle and the Environment (ISCOLE)	Community	urban	9-11	9-11	327	222	327	222	

Country	Study years	Survey/study name/citation	Level of representativeness	Rural, urban or both	Age range as in NCD-RisC database *		Sample size (height)		Sample size (BMI)		Note
					Female	Male	Female	Male	Female	Male	
South Africa	2012	National Income Dynamics Study Wave III	National	both	5+	5+	7919	7219	5085	5087	
South Africa	2012	South African National Health and Nutrition Examination Survey	National	both	5+	5+	837	414			
South Africa	2014-2015	National Income Dynamics Study Wave IV	National	both	5+	5+	9539	8926	6071	6080	
South Africa	2016	DHS	National	both	15-49	15-59	1736	1420	563	565	
South Africa	2017	National Income Dynamics Study Wave V	National	both	5+	5+	9820	9006	6323	6166	
South Africa	2017-2019	The Exercise, Arterial Modulation and Nutrition in Youth South Africa (ExAMIN Youth SA) Study	Subnational	urban	5-9	5-9	575	483	575	483	
South Africa	2018-2020	Vukuzazi Study	Community	both	15+	15+	3901	2725	1485	1436	
Zimbabwe	1991	Zinyowera et al., Cent Afr J Med 40(2):33-8, 1994	Community	both	18+	18+			113	119	
Zimbabwe	1999	DHS	National	both	15-49		3533		1315		
Zimbabwe	2005-2006	DHS	National	both	15-49		5413		1962		
Zimbabwe	2010-2011	DHS	National	both	15-49	15-54	5408	4312	1818	1812	
Zimbabwe	2015	DHS	National	both	15-49	15-54	5633	4597	2049	2051	
West Africa											
Benin	1996	DHS	National	both	20-49		1310				
Benin	2001	DHS	National	both	15-49		3576		1118		
Benin	2006	DHS	National	both	15-49		9174		2602		
Benin	2007	STEPS	Community	urban	25-64	25-64	406	243			
Benin	2008	STEPS	National	both	25-64	25-64	563	631			
Benin	2011-2012	DHS	National	both	15-49		8635		2699		
Benin	2015	STEPS	National	both	18-69	18-69	833	659	101	70	
Benin	2017-2018	DHS	National	both	15-49		4589		1538		
Burkina Faso	1998-1999	DHS	National	both	20-49		1818				
Burkina Faso	2002	Vulnérabilité Alimentaire et Sécurité Nutritionnelle dans la Gnagna (VASN-Gnagna)	Subnational	rural	5+	5+	2219	1025	882	787	
Burkina Faso	2003	DHS	National	both	15-49		6887		2520		
Burkina Faso	2010	DHS	National	both	15-49		4800		1587		
Burkina Faso	2013	STEPS	National	both	25-64	25-64	634	571			
Cabo Verde	2007	STEPS	National	both	25-64	25-64	130	119			
Cabo Verde	2019	STEPS	National	both	18-69	18-69	609	464	68	59	
Cameroon	1998	DHS	National	both	20-49		988				
Cameroon	1998-1999	Essential Non-communicable disease Health Intervention Project (ENHIP)	Community	both	15+	15+	554	424	211	186	
Cameroon	2003	STEPS	Subnational	urban	15+	15+	2648	1744	898	631	
Cameroon	2004	DHS	National	both	15-49		3223		1179		
Cameroon	2007	Cameroon Burden of Diabetes - Second Survey	Subnational	urban	18+	18+	1238	766		2	
Cameroon	2009	National Survey of Micronutrient Status and Consumption of Fortifiable Foods	National	both	15-49		617		75		
Cameroon	2009-2012	Anthropologie nutritionnelle des migrants d'Afrique centrale à la ville et en France	Subnational	both	18-76	18-76	203	157	43	26	
Cameroon	2011	DHS	National	both	15-49		4945		1741		
Cameroon	2013	Prevalence and risk factors of chronic kidney disease in urban adult Cameroonians according to three common estimators of the glomerular filtration rate: a cross-sectional study	Community	urban	19-83	19-83	31	45	2		
Cameroon	2014	Prevalence and determinants of chronic kidney disease in rural and urban Cameroonians: A cross-sectional study	Community	both	20-90	20-90	45	35			
Cameroon	2014-2015	Cardiovascular risk factors screening in urban and rural areas in the Far-North Region Cameroon	Subnational	both	20+	20+	108	208			
Cameroon	2018	Prevalence and determinants of chronic kidney disease in urban adults' populations of northern Cameroon	Community	urban	20-85	20-85	45	43			
Cameroon	2018-2019	DHS	National	both	15-64		4141		1558		
Chad	1996-1997	DHS	National	both	20-49		2254				
Chad	2004	DHS	National	both	20-49		1746				
Chad	2008	STEPS	Community	urban	25-64	25-64	220	240			
Chad	2014-2015	DHS	National	both	15-49		6515		2172		
Côte d'Ivoire	1998-1999	DHS	National	both	15-49		1913		735		
Côte d'Ivoire	2005	STEPS	Subnational	both	15-64	15-64	1356	837	373	240	
Côte d'Ivoire	2011-2012	DHS	National	both	15-49		3004		958		
Gambia	2010	STEPS	National	both	25-64	25-64	775	293			
Gambia	2013	DHS	National	both	15-49		2794		1011		
Gambia	2018	The Gambia Micronutrient Survey (GMNS)	National	both	15-49		963		404		
Gambia	2019-2020	DHS	National	both	15-49		3486		1272		
Ghana	1998	DHS	National	both	20-49		1098				
Ghana	2003	Women's Health Study of Accra (WHSA-I)	Community	urban	18+		265		57		
Ghana	2003	DHS	National	both	15-49		2940		989		
Ghana	2006	STEPS	Community	urban	25+	25+	321	162			
Ghana	2008	DHS	National	both	15-49		2665		959		
Ghana	2008-2010	Women's Health Study of Accra (WHSA-II)	Community	urban	18+		621				
Ghana	2012-2014	Research on Obesity and Diabetes among African Migrants (RODAM), control group	Subnational	both	25+	25+	174	94			
Ghana	2014	DHS	National	both	15-49	15-59	2465	2051	857	871	
Ghana	2016-2017	Ghana Living Standards Survey	National	both	5+	5+	11677	11734	8267	8731	
Ghana	2017	Ghana Micronutrient Survey	National	both	15-49		568		217		
Ghana	2018	Intervention to reduce cardiovascular disease risk factors among secondary school students: Randomised controlled trial - Baseline	Subnational	both	14-19	14-19	437	411	437	411	
Ghana	2018	Intervention to reduce cardiovascular disease risk factors among secondary school students: Randomised controlled trial - Post-intervention study	Subnational	both	14-19	14-19	218	199	218	199	
Guinea	1999	DHS	National	both	20-49		1715				

Country	Study years	Survey/study name/citation	Level of representativeness	Rural, urban or both	Age range as in NCD-RisC database *		Sample size (height)		Sample size (BMI)		Note
					Female	Male	Female	Male	Female	Male	
Guinea	2005	DHS	National	both	15-49		2033		740		
Guinea	2009	STEPS	Subnational	both	15-64	15-64	657	432	203	149	
Guinea	2012	DHS	National	both	15-49		2708		997		
Guinea	2018	DHS	National	both	15-49		3069		1176		
Guinea Bissau	2010	Multiple Indicator Cluster Survey	National	both	15-49		5116		1734		
Liberia	2006-2007	DHS	National	both	15-49		3925		1233		
Liberia	2011	STEPS	National	both	25-64	25-64	353	227			
Liberia	2013	DHS	National	both	15-49	15-49	2588	2183	939	865	
Liberia	2019	DHS	National	both	15-49		2220		841		
Mali	1995-1996	DHS	National	both	20-49		2331				
Mali	1997	Programme Intégré de Développement de Bafoulabé	Community	rural	15-45	15-45	422	242	159	94	
Mali	1999-2000	Bafoulabe Iodine Study	Community	rural	15-45		271		104		
Mali	2001	DHS	National	both	15-49		6786		2102		
Mali	2006	DHS	National	both	15-49		8211		2734		
Mali	2007	STEPS	Subnational	both	15-64	15-64	785	538	299	191	
Mali	2012-2013	DHS	National	both	15-49		2948		839		
Mali	2013	STEPS	Subnational	both	15-65	15-65	412	204	144	69	
Mali	2013	Santé Nutritionnelle à Assise Comunautaire dans la région de Kayes (SNACK)	Subnational	rural	20-68		2484				
Mali	2018	DHS	National	both	15-49		2908		924		
Mauritania	2000-2001	DHS	National	both	15-49		1432				
Mauritania	2006	STEPS	Community	urban	15-64	15-64	505	413	154	128	
Mauritania	2019-2021	DHS	National	both	15-49		4223		1656		
Niger	1998	DHS	National	both	20-49		1856				
Niger	2006	DHS	National	both	15-49		2636		850		
Niger	2007	STEPS	National	both	15-64	15-64	539	390	135	116	
Niger	2012	DHS	National	both	15-49		2794		775		
Nigeria	1999	DHS	National	both	20-49		1295				
Nigeria	2003	DHS	National	both	15-49		4456		1576		
Nigeria	2006	Senbanjo et al., West Afr J Med 30(6):425-31, 2011	Community	urban	5-19	5-19	274	296	274	296	
Nigeria	2008	DHS	National	both	15-49		18426		5850		
Nigeria	2009	Community Health Plan - Kwara Central Survey	Community	rural	5+	5+	1301	1334	1004	1096	
Nigeria	2011	Community Health Plan - Kwara Central Survey	Community	rural	5+	5+	491	504	386	436	
Nigeria	2013	DHS	National	both	15-49		21338		7206		
Nigeria	2013	Community Health Plan - Kwara Central Survey	Community	rural	5+	5+	416	453	355	394	
Nigeria	2018	Hypertension Prevalence, Awareness, Treatment and Control in Rural Area, Nigeria	Community	rural	18+	18+	64	72	20	11	
Nigeria	2017-2019	Removing the Mask on Hypertension (REMAH)	National	both	18+	18+	492	427	67	59	
Sao Tome and Principe	2008-2009	DHS	National	both	15-49	15-59	1448	1150	465	455	
Sao Tome and Principe	2009	STEPS	National	both	25-64	25-64	339	284			
Sao Tome and Principe	2019	STEPS	National	both	18-69	18-69	409	320	72	47	
Senegal	2003	Perceptions of healthy and desirable body size in urban Senegalese women	Community	urban	20-50		117				
Senegal	2005	DHS	National	both	15-49		2810		1107		
Senegal	2010-2011	DHS	National	both	15-49	15-59	3594	2671	1351	1217	
Senegal	2010-2012	Biocultural determinants of overweight and obesity in the context of nutrition transition in Senegal: a holistic anthropological approach	Subnational	both	18+	18+	124	109	34	15	
Senegal	2015	Les maladies chroniques au Sénégal: Une écologie de la santé comparative entre Dakar et Widou Thiengoly	Community	both	20+	20+	320	306			
Senegal	2015	STEPS	National	both	18-70	18-70	1135	613			
Sierra Leone	2008	DHS	National	both	15-49		2007		543		
Sierra Leone	2009	STEPS	National	both	25-64	25-64	814	430			
Sierra Leone	2013	DHS	National	both	15-49	15-59	4529	3409	1744	1449	
Sierra Leone	2019	DHS	National	both	15-49	15-59	4144	3074	1544	1406	
Togo	1998	DHS	National	both	20-49		1779				
Togo	2010	STEPS	National	both	15-64	15-64	1001	841	248	245	
Togo	2013-2014	DHS	National	both	15-49		2566		869		
Togo	2014	Impact evaluation of a cash transfer program in North Togo	Subnational	rural	20-65		1959				

*As described in Methods section, participants aged 5-19 years were used in the BMI analysis and participants aged 5-29 years were used in the height analysis.

1. National studies for the 3 years prior to 1990 were assigned to 1990 in the BMI analysis so that they can inform the estimates in countries with slightly earlier national data.

2. This research uses data from China Health and Nutrition Survey (CHNS). We thank the National Institute of Nutrition and Food Safety, China Center for Disease Control and Prevention, Carolina Population Center (5 R24 HD050924), the University of North Carolina at Chapel Hill, the NIH (R01-HD30880, DK056350, R24-HD050924, and R01-HD38700) and the Fogarty International Center, NIH for financial support for the CHNS data collection and analysis files from 1989 to 2011 and future surveys, and the China-Japan Friendship Hospital, Ministry of Health for support for CHNS 2009.

3. This research uses data from Australia Health Survey (AHS). We thank the Health Section, Australian Bureau of Statistics, Belconnen, ACT, Australia for support for AHS 2011-2012.

4. Accessed via the Irish Social Science Data Archive - www.ucd.ie/issda

5. This research uses data from Add Health, a program project designed by J. Richard Udry, Peter S. Bearman, and Kathleen Mullan Harris, and funded by a grant P01-HD31921 from the Eunice Kennedy Shriver National Institute of Child Health and Human Development, with cooperative funding from 17 other agencies. Special acknowledgment is due Ronald R. Rindfuss and Barbara Entwisle for assistance in the original design. Persons interested in obtaining data files from Add Health should contact Add Health, Carolina Population Center, 123 W. Franklin Street, Chapel Hill, NC 27516-2524 (addhealth@unc.edu). No direct support was received from grant P01-HD31921 for this analysis.

6. Due the COVID-19 pandemic the NHANES 2019-2020 cycle was not completed. As a result the data are not nationally representative and considered subnational.

7. The Swiss BMI Monitoring Study was supported by the Health Service of the town of Berne, Basel Health Service for Children and Adolescents, and the School-Medical Service of the town of Zurich.

8. Data used for this research was provided by the INMA – INfancia y Medio Ambiente [Environment and Childhood] Project (www.proyectoinma.org), which is supported in part by funds. This study was funded by grants from Instituto de Salud Carlos III (Red INMA G03/176 and CB06/02/0041), Spanish Ministry of Health (FIS-97/1102, FIS-07/0252, FIS-PS09/00362, 97/0588, 00/0021-2, PI061756, PS0901958, PI14/00677 incl.FEDER funds, FIS-PS09/00090, PI041436, FIS-PI042018, FIS-PI06/0867, PI081151 incl. FEDER funds, FIS-PI09/02311 and FIS-PI13/02187, and FIS-FEDER 03/1615, 04/1509, 04/1112, 04/1931, 05/1079, 05/1052, 06/1213, 07/0314, 09/02647, 11/01007, 11/0178, 11/02591, 11/02038, PI12/01890 incl. FEDER funds, 13/1944, 13/2032, FIS-PI13/02429, 14/0891, 14/1687 and CP13/00054 incl. FEDER funds), Spanish Ministry of Economy and Competitiveness (SAF2012-32991 incl. FEDER funds), CIBERESP, Generalitat de Catalunya-CIRIT 1999SGR 00241, Generalitat de Catalunya-AGAUR (2009 SGR 501, 2014 SGR 822), the Conselleria de Sanitat Generalitat Valenciana, Department of Health of the Basque Government (2005111093, 2009111069 and 2013111089), the Provincial Government of Gipuzkoa (DFG06/004 and DFG08/001), Fundació La Caixa (97/009-00 and 00/077-00), Beca de la IV convocatoria de Ayudas a la Investigación en Enfermedades Neurodegenerativas de La Caixa, Fundació La marató de TV3 (090430), Obra Social Cajastur/Fundación Liberbank, Universidad de Oviedo, the EU Commission (QLK4-1999-01422, QLK4-CT-2000-00263, QLK4-2002-00603, CONTAMED FP7-ENV-212502, FP7-ENV-2011 cod 282957, HEALTH.2010.2.4.5-1, 261357, 308333, 603794), Agence Nationale de Securite Sanitaire de l'Alimentation de l'Environnement et du Travail (1262C0010), Consejería de Salud de la Junta de Andalucía (grant number 183/07) and Annual agreements with municipalities in the study area (Zumarraga, Urretxu, Legazpi, Azkoitia y Azpeitia y Beasain).

9.Dr Take Naseri (Ministry of Health, Samoa), and Muagututia Sefuiva Reupena (Lutia I Puava Ae Mapu I Fagalele) contributed to the GWAS studies in Samoa.

10.The bibliographic citation for this data source is: Desai, Sonalde, Vanneman, Reeve, and National Council of Applied Economic Research, New Delhi. India Human Development Survey (IHDS), 2005. Inter-university Consortium for Political and Social Research [distributor], 2018-08-08. https://doi.org/10.3886/ICPSR22626.v12

11.The bibliographic citation for this data source is: Desai, Sonalde, Reeve Vanneman and National Council of Applied Economic Research. India Human Development Survey-II (IHDS-II), 2011-12. Inter-university Consortium for Political and Social Research [distributor], 2018-08-08. https://doi.org/10.3886/ICPSR36151.v6

Supplementary Table 3. Urban and rural mean height and urban-rural difference in mean height in 1990 and 2020 by sex, age-standardised and at ages 5, 10, 15 and 19 years.

Girls	Mean height in 1990 (cm)					Mean height in 2020 (cm)				
	Age-standardised	5 years	10 years	15 years	19 years	Age-standardised	5 years	10 years	15 years	19 years
**Central and eastern Europe**										
Central Europe										
Albania										
rural	143.8 (141.5, 146.2)	113.2 (110.4, 115.9)	138.5 (136.1, 140.9)	158.9 (156.1, 161.8)	160.7 (157.2, 164.2)	145.8 (144.5, 147.1)	116.4 (114.1, 118.6)	140.9 (139.4, 142.4)	160.3 (159.1, 161.6)	161.4 (160.0, 163.0)
urban	146.1 (143.7, 148.5)	115.6 (112.8, 118.3)	140.9 (138.5, 143.2)	161.2 (158.3, 164.0)	162.9 (159.3, 166.5)	147.1 (145.8, 148.3)	117.8 (115.6, 120.0)	142.2 (140.7, 143.7)	161.5 (160.3, 162.7)	162.6 (161.1, 164.1)
urban-rural difference	2.3 (1.4, 3.3)	2.4 (1.3, 3.6)	2.4 (1.4, 3.3)	2.3 (1.3, 3.2)	2.2 (1.1, 3.3)	1.3 (0.6, 1.9)	1.4 (0.4, 2.4)	1.3 (0.6, 2.0)	1.2 (0.5, 1.9)	1.1 (0.3, 2.0)
Bosnia and Herzegovina										
rural	148.8 (145.4, 152.2)	116.0 (110.8, 121.2)	143.5 (139.5, 147.6)	164.5 (161.2, 167.8)	167.0 (163.7, 170.2)	150.0 (146.4, 153.7)	118.0 (111.8, 124.1)	145.0 (140.6, 149.3)	165.3 (162.0, 168.7)	167.3 (164.3, 170.3)
urban	150.2 (146.9, 153.7)	117.5 (112.3, 122.7)	144.9 (140.9, 149.0)	165.9 (162.6, 169.2)	168.3 (165.0, 171.6)	150.4 (146.7, 154.1)	118.4 (112.3, 124.5)	145.3 (141.0, 149.7)	165.6 (162.3, 169.0)	167.6 (164.7, 170.5)
urban-rural difference	1.4 (0.1, 2.7)	1.5 (-0.1, 3.1)	1.5 (0.1, 2.8)	1.4 (0.0, 2.7)	1.3 (-0.2, 2.8)	0.3 (-0.9, 1.6)	0.4 (-1.2, 2.0)	0.4 (-1.0, 1.7)	0.3 (-1.0, 1.5)	0.3 (-1.1, 1.6)
Bulgaria										
rural	145.5 (143.5, 147.6)	115.0 (112.9, 117.2)	140.6 (138.6, 142.5)	160.3 (157.8, 162.8)	161.8 (158.3, 165.4)	146.3 (144.9, 147.6)	116.4 (114.4, 118.4)	141.5 (140.0, 143.0)	160.8 (159.0, 162.6)	162.2 (159.9, 164.5)
urban	148.1 (146.3, 149.9)	117.8 (115.9, 119.8)	143.2 (141.5, 145.0)	162.8 (160.5, 165.1)	164.1 (160.8, 167.5)	147.8 (146.6, 149.1)	118.2 (116.3, 120.1)	143.1 (141.7, 144.5)	162.2 (160.6, 163.9)	163.5 (161.3, 165.6)
urban-rural difference	2.5 (1.4, 3.7)	2.8 (1.6, 4.0)	2.6 (1.5, 3.7)	2.4 (1.2, 3.7)	2.3 (0.7, 3.8)	1.5 (0.6, 2.5)	1.8 (0.7, 2.9)	1.6 (0.6, 2.5)	1.4 (0.3, 2.5)	1.3 (-0.1, 2.7)
Croatia										
rural	150.0 (148.2, 151.8)	117.5 (115.3, 119.7)	145.1 (143.3, 147.1)	165.5 (163.4, 167.7)	166.6 (164.0, 169.2)	150.3 (149.2, 151.4)	118.3 (116.4, 120.1)	145.7 (144.4, 147.0)	165.7 (164.3, 167.1)	166.4 (164.8, 168.2)
urban	151.4 (149.6, 153.2)	118.6 (116.4, 120.8)	146.5 (144.7, 148.4)	167.1 (165.0, 169.3)	168.4 (165.8, 171.0)	150.7 (149.7, 151.8)	118.3 (116.5, 120.2)	146.0 (144.8, 147.2)	166.2 (164.9, 167.6)	167.2 (165.6, 168.9)
urban-rural difference	1.5 (0.6, 2.3)	1.1 (0.1, 2.1)	1.4 (0.5, 2.2)	1.6 (0.7, 2.5)	1.8 (0.8, 2.9)	0.4 (-0.3, 1.1)	0.0 (-0.8, 0.9)	0.3 (-0.4, 1.0)	0.5 (-0.2, 1.3)	0.7 (-0.2, 1.7)
Czechia										
rural	147.8 (146.7, 148.9)	114.3 (112.9, 115.8)	142.2 (140.8, 143.6)	163.8 (162.5, 165.2)	166.7 (165.3, 168.2)	149.6 (148.5, 150.7)	117.1 (115.4, 118.8)	144.3 (143.0, 145.5)	165.2 (163.7, 166.7)	167.6 (166.1, 169.2)
urban	148.7 (147.8, 149.7)	115.2 (113.9, 116.5)	143.1 (141.9, 144.3)	164.8 (163.6, 166.0)	167.7 (166.3, 169.1)	149.5 (148.4, 150.5)	116.9 (115.2, 118.6)	144.1 (142.9, 145.3)	165.1 (163.6, 166.5)	167.5 (166.0, 169.0)
urban-rural difference	0.9 (0.0, 1.8)	0.9 (-0.2, 1.9)	0.9 (0.0, 1.8)	0.9 (-0.1, 1.9)	1.0 (-0.2, 2.2)	-0.1 (-0.9, 0.7)	-0.2 (-1.1, 0.7)	-0.2 (-0.9, 0.6)	-0.1 (-1.0, 0.8)	-0.1 (-1.2, 1.1)
Hungary										
rural	145.9 (143.1, 148.6)	113.5 (110.7, 116.2)	142.0 (139.4, 144.6)	160.5 (157.1, 163.9)	161.6 (157.3, 166.0)	148.8 (148.0, 149.6)	118.6 (116.8, 120.4)	145.8 (144.9, 146.7)	162.4 (161.4, 163.3)	162.3 (161.0, 163.6)
urban	148.5 (145.7, 151.2)	115.4 (112.8, 118.1)	144.5 (141.9, 147.0)	163.4 (160.0, 166.7)	164.9 (160.5, 169.1)	150.3 (149.6, 151.1)	119.4 (117.7, 121.2)	147.1 (146.2, 147.9)	164.2 (163.4, 165.0)	164.4 (163.3, 165.5)
urban-rural difference	2.6 (1.6, 3.6)	2.0 (0.8, 3.1)	2.4 (1.5, 3.4)	2.9 (1.8, 3.9)	3.2 (1.9, 4.5)	1.5 (1.0, 2.0)	0.9 (0.0, 1.7)	1.3 (0.8, 1.8)	1.8 (1.1, 2.5)	2.1 (1.2, 3.1)
North Macedonia										
rural	143.6 (141.5, 145.7)	113.3 (110.6, 116.2)	138.7 (136.3, 141.2)	158.3 (155.8, 160.7)	159.6 (157.0, 162.3)	145.6 (144.1, 147.1)	117.0 (114.6, 119.2)	141.3 (139.7, 142.9)	159.5 (157.2, 161.7)	159.8 (157.0, 162.9)
urban	145.6 (143.5, 147.7)	115.4 (112.6, 118.2)	140.8 (138.3, 143.3)	160.3 (157.8, 162.7)	161.6 (159.0, 164.2)	146.5 (145.0, 148.0)	117.9 (115.6, 120.2)	142.2 (140.6, 143.8)	160.4 (158.1, 162.6)	160.7 (157.9, 163.8)
urban-rural difference	2.0 (0.8, 3.2)	2.0 (0.9, 3.2)	2.0 (0.9, 3.1)	2.0 (0.6, 3.4)	2.0 (0.3, 3.7)	0.9 (0.0, 1.8)	1.0 (0.0, 1.9)	0.9 (0.1, 1.8)	0.9 (-0.3, 2.1)	0.9 (-0.5, 2.3)
Montenegro										
rural	148.9 (145.9, 151.9)	116.3 (112.9, 119.8)	143.1 (140.1, 146.0)	164.7 (161.2, 168.3)	167.9 (163.4, 172.6)	150.9 (149.9, 151.8)	118.6 (117.0, 120.2)	145.1 (144.1, 146.2)	166.5 (165.3, 167.8)	169.5 (167.7, 171.4)
urban	150.8 (147.9, 153.8)	118.2 (114.9, 121.7)	145.0 (142.1, 147.9)	166.7 (163.2, 170.1)	169.9 (165.4, 174.5)	151.7 (150.8, 152.6)	119.4 (117.9, 120.9)	146.0 (144.9, 147.0)	167.4 (166.1, 168.6)	170.4 (168.6, 172.2)
urban-rural difference	1.9 (0.8, 3.0)	1.9 (0.8, 3.1)	1.9 (0.9, 3.0)	1.9 (0.7, 3.1)	1.9 (0.4, 3.4)	0.8 (0.1, 1.6)	0.9 (0.0, 1.7)	0.8 (0.1, 1.6)	0.8 (-0.1, 1.7)	0.8 (-0.4, 2.0)
Poland										
rural	145.9 (144.7, 147.1)	112.6 (111.1, 114.1)	141.0 (139.7, 142.2)	161.5 (160.2, 162.8)	163.7 (162.4, 164.9)	147.3 (146.4, 148.2)	112.8 (111.5, 114.2)	142.2 (141.2, 143.2)	163.4 (162.5, 164.3)	166.0 (164.9, 167.1)
urban	146.9 (145.7, 148.1)	113.6 (112.1, 115.1)	142.0 (140.8, 143.2)	162.5 (161.2, 163.7)	164.6 (163.3, 165.8)	147.1 (146.3, 148.0)	112.7 (111.5, 114.0)	142.0 (141.1, 142.9)	163.1 (162.3, 164.0)	165.7 (164.6, 166.9)
urban-rural difference	1.0 (0.3, 1.7)	1.1 (0.2, 1.9)	1.0 (0.3, 1.7)	0.9 (0.3, 1.6)	0.9 (0.1, 1.7)	-0.2 (-0.8, 0.4)	-0.1 (-0.9, 0.7)	-0.2 (-0.8, 0.4)	-0.2 (-0.8, 0.4)	-0.3 (-1.0, 0.4)
Romania										
rural	144.8 (142.9, 146.7)	110.3 (108.4, 112.1)	139.5 (137.6, 141.3)	160.9 (158.6, 163.1)	164.2 (161.2, 167.1)	146.0 (145.1, 147.0)	112.7 (111.1, 114.2)	141.2 (140.2, 142.2)	161.5 (160.4, 162.6)	163.8 (162.2, 165.3)
urban	147.7 (145.7, 149.6)	113.1 (111.3, 115.0)	142.3 (140.5, 144.1)	163.7 (161.4, 166.0)	167.0 (164.0, 170.0)	147.7 (146.8, 148.6)	114.3 (112.9, 115.8)	142.9 (141.9, 143.8)	163.2 (162.1, 164.2)	165.5 (164.0, 166.9)
urban-rural difference	2.8 (2.1, 3.6)	2.8 (1.9, 3.7)	2.8 (2.1, 3.6)	2.8 (2.0, 3.7)	2.8 (1.9, 3.8)	1.7 (1.0, 2.3)	1.7 (0.9, 2.4)	1.7 (1.0, 2.3)	1.7 (1.0, 2.4)	1.7 (0.8, 2.6)
Serbia										
rural	148.2 (146.6, 149.9)	115.5 (113.1, 117.9)	143.1 (141.3, 144.9)	164.0 (162.1, 165.9)	165.4 (163.4, 167.5)	150.3 (149.2, 151.4)	117.5 (115.4, 119.7)	145.2 (144.0, 146.5)	166.1 (164.7, 167.4)	167.6 (165.9, 169.2)
urban	149.9 (148.3, 151.6)	117.1 (114.8, 119.4)	144.8 (143.1, 146.5)	165.8 (163.8, 167.7)	167.3 (165.2, 169.3)	150.9 (149.8, 152.0)	118.0 (115.9, 120.1)	145.8 (144.5, 147.0)	166.7 (165.4, 168.0)	168.3 (166.7, 169.9)
urban-rural difference	1.7 (0.9, 2.5)	1.6 (0.6, 2.6)	1.7 (0.8, 2.5)	1.7 (0.9, 2.5)	1.8 (0.9, 2.8)	0.6 (-0.1, 1.3)	0.5 (-0.4, 1.4)	0.5 (-0.2, 1.3)	0.6 (-0.1, 1.3)	0.7 (-0.2, 1.6)
Slovakia										
rural	147.5 (146.3, 148.8)	114.6 (113.0, 116.2)	142.3 (140.9, 143.7)	163.4 (161.8, 164.9)	165.3 (163.4, 167.1)	149.7 (148.5, 151.0)	117.2 (115.0, 119.3)	144.7 (143.3, 146.0)	165.3 (164.0, 166.7)	166.9 (165.2, 168.6)
urban	149.1 (147.9, 150.4)	116.0 (114.4, 117.6)	143.8 (142.5, 145.2)	165.0 (163.5, 166.6)	167.0 (165.2, 168.9)	150.3 (149.1, 151.4)	117.6 (115.3, 119.8)	145.2 (143.9, 146.5)	165.9 (164.6, 167.3)	167.6 (166.0, 169.2)
urban-rural difference	1.6 (0.7, 2.4)	1.4 (0.5, 2.4)	1.5 (0.7, 2.4)	1.7 (0.7, 2.6)	1.8 (0.6, 2.9)	0.5 (-0.3, 1.4)	0.4 (-0.6, 1.3)	0.5 (-0.3, 1.3)	0.6 (-0.3, 1.6)	0.7 (-0.5, 1.9)
Slovenia										

Girls	Mean height in 1990 (cm)					Mean height in 2020 (cm)				
	Age-standardised	5 years	10 years	15 years	19 years	Age-standardised	5 years	10 years	15 years	19 years
rural	148.3 (147.8, 148.8)	116.6 (115.8, 117.4)	143.2 (142.6, 143.8)	163.9 (163.3, 164.5)	164.8 (164.0, 165.6)	150.6 (149.9, 151.4)	118.1 (116.8, 119.5)	145.5 (144.7, 146.3)	166.5 (165.8, 167.2)	167.5 (166.6, 168.3)
urban	149.0 (148.5, 149.5)	117.2 (116.4, 117.9)	143.8 (143.3, 144.4)	164.6 (164.0, 165.2)	165.5 (164.8, 166.3)	150.2 (149.6, 150.9)	117.6 (116.3, 118.9)	145.1 (144.3, 145.8)	166.1 (165.4, 166.8)	167.1 (166.3, 167.9)
urban-rural difference	0.7 (-0.1, 1.4)	0.6 (-0.3, 1.5)	0.6 (-0.1, 1.3)	0.7 (-0.2, 1.6)	0.7 (-0.4, 1.9)	-0.4 (-1.2, 0.4)	-0.5 (-1.5, 0.5)	-0.4 (-1.2, 0.3)	-0.4 (-1.2, 0.5)	-0.4 (-1.4, 0.8)
Eastern Europe										
Belarus										
rural	145.7 (141.6, 149.8)	111.3 (105.7, 117.0)	139.1 (134.7, 143.7)	162.6 (158.4, 167.0)	165.2 (160.6, 170.0)	148.2 (144.5, 152.1)	114.7 (108.5, 121.3)	142.0 (137.5, 146.6)	164.8 (161.5, 168.1)	166.8 (164.4, 169.1)
urban	147.5 (143.6, 151.6)	113.2 (107.6, 118.9)	140.9 (136.6, 145.5)	164.5 (160.3, 168.8)	167.0 (162.4, 171.7)	149.2 (145.4, 153.0)	115.6 (109.3, 122.1)	142.9 (138.4, 147.6)	165.7 (162.4, 169.1)	167.7 (165.3, 170.0)
urban-rural difference	1.9 (0.4, 3.3)	1.8 (0.1, 3.5)	1.9 (0.4, 3.3)	1.9 (0.4, 3.3)	1.9 (0.2, 3.6)	0.9 (-0.4, 2.2)	0.9 (-0.7, 2.5)	0.9 (-0.4, 2.3)	0.9 (-0.4, 2.2)	0.9 (-0.5, 2.4)
Estonia										
rural	147.7 (145.9, 149.5)	112.6 (109.8, 115.4)	141.5 (139.3, 143.6)	164.8 (162.9, 166.7)	166.8 (165.0, 168.7)	150.8 (149.7, 152.0)	116.2 (114.3, 118.2)	144.9 (143.6, 146.2)	167.6 (166.2, 168.9)	168.9 (167.4, 170.6)
urban	148.5 (146.7, 150.2)	113.5 (110.8, 116.2)	142.3 (140.2, 144.4)	165.5 (163.6, 167.4)	167.4 (165.6, 169.2)	150.7 (149.5, 151.8)	116.3 (114.4, 118.2)	144.8 (143.5, 146.1)	167.4 (166.0, 168.8)	168.7 (167.1, 170.2)
urban-rural difference	0.8 (-0.1, 1.7)	1.0 (-0.2, 2.2)	0.8 (-0.1, 1.8)	0.7 (-0.2, 1.6)	0.6 (-0.3, 1.6)	-0.1 (-0.8, 0.6)	0.1 (-0.9, 1.0)	-0.1 (-0.8, 0.7)	-0.2 (-0.9, 0.5)	-0.3 (-1.1, 0.5)
Latvia										
rural	147.4 (145.0, 149.7)	112.4 (109.5, 115.3)	140.5 (138.0, 143.1)	164.6 (161.7, 167.5)	167.6 (164.4, 170.8)	150.2 (148.8, 151.7)	116.4 (114.2, 118.5)	143.8 (142.4, 145.3)	166.9 (164.9, 169.0)	169.2 (166.3, 172.2)
urban	149.0 (146.7, 151.4)	114.1 (111.3, 117.0)	142.2 (139.7, 144.8)	166.2 (163.3, 169.2)	169.2 (166.0, 172.5)	150.9 (149.5, 152.3)	117.1 (115.0, 119.2)	144.6 (143.2, 146.0)	167.6 (165.6, 169.6)	169.8 (166.9, 172.9)
urban-rural difference	1.7 (0.6, 2.8)	1.7 (0.6, 2.8)	1.7 (0.6, 2.8)	1.6 (0.4, 2.9)	1.6 (0.1, 3.2)	0.7 (-0.1, 1.5)	0.8 (-0.1, 1.6)	0.7 (0.0, 1.5)	0.7 (-0.3, 1.7)	0.7 (-0.7, 2.0)
Lithuania										
rural	147.4 (145.3, 149.5)	114.7 (111.7, 117.7)	141.1 (138.7, 143.5)	163.8 (161.3, 166.2)	165.5 (163.2, 167.8)	149.7 (148.4, 151.1)	117.8 (115.4, 120.2)	143.7 (142.3, 145.2)	165.8 (163.9, 167.6)	167.0 (164.5, 169.7)
urban	148.9 (146.9, 151.0)	116.5 (113.6, 119.6)	142.7 (140.3, 145.1)	165.2 (162.7, 167.8)	166.8 (164.4, 169.3)	150.4 (149.0, 151.8)	118.7 (116.3, 121.1)	144.5 (143.0, 145.9)	166.3 (164.5, 168.2)	167.5 (165.0, 170.2)
urban-rural difference	1.6 (0.5, 2.6)	1.8 (0.7, 2.9)	1.6 (0.6, 2.6)	1.5 (0.3, 2.6)	1.4 (-0.1, 2.8)	0.7 (-0.1, 1.5)	0.9 (0.0, 1.8)	0.7 (0.0, 1.5)	0.6 (-0.4, 1.5)	0.5 (-0.7, 1.7)
Moldova										
rural	143.5 (141.2, 145.8)	110.2 (106.7, 113.7)	137.2 (134.7, 139.6)	160.2 (157.4, 163.0)	161.7 (158.5, 164.9)	146.1 (144.1, 148.0)	113.9 (110.3, 117.5)	140.2 (137.9, 142.5)	162.2 (160.1, 164.1)	163.0 (161.1, 164.9)
urban	145.3 (143.0, 147.5)	111.9 (108.5, 115.4)	139.0 (136.5, 141.4)	161.9 (159.2, 164.6)	163.5 (160.4, 166.6)	146.9 (144.9, 148.8)	114.8 (111.2, 118.4)	141.1 (138.7, 143.3)	163.0 (161.0, 164.9)	163.9 (162.0, 165.7)
urban-rural difference	1.8 (0.7, 2.8)	1.8 (0.5, 3.1)	1.8 (0.7, 2.8)	1.8 (0.7, 2.8)	1.8 (0.6, 2.9)	0.8 (-0.1, 1.8)	0.8 (-0.5, 2.1)	0.8 (-0.2, 1.9)	0.8 (-0.1, 1.8)	0.9 (-0.2, 1.9)
Russian Federation										
rural	142.4 (141.6, 143.1)	107.3 (106.3, 108.4)	135.7 (134.8, 136.5)	159.5 (158.7, 160.4)	162.5 (161.5, 163.5)	146.6 (145.2, 148.0)	113.4 (111.4, 115.6)	140.5 (138.9, 142.1)	162.8 (161.4, 164.3)	164.7 (163.2, 166.2)
urban	144.8 (144.1, 145.5)	110.2 (109.2, 111.2)	138.2 (137.4, 139.0)	161.7 (160.9, 162.6)	164.4 (163.4, 165.4)	148.1 (146.8, 149.4)	115.4 (113.5, 117.4)	142.1 (140.7, 143.5)	164.1 (162.8, 165.4)	165.7 (164.4, 167.0)
urban-rural difference	2.4 (2.0, 2.9)	2.9 (2.2, 3.6)	2.6 (2.1, 3.1)	2.2 (1.7, 2.7)	1.9 (1.4, 2.5)	1.5 (0.6, 2.3)	2.0 (1.0, 3.0)	1.6 (0.8, 2.5)	1.3 (0.4, 2.1)	1.0 (0.1, 1.9)
Ukraine										
rural	144.8 (142.0, 147.7)	110.5 (107.3, 113.7)	138.2 (135.5, 141.0)	162.0 (158.7, 165.3)	164.1 (159.9, 168.6)	147.5 (146.4, 148.6)	114.3 (112.3, 116.2)	141.3 (140.1, 142.4)	164.1 (163.0, 165.2)	165.6 (164.2, 167.2)
urban	146.4 (143.5, 149.2)	111.7 (108.5, 114.8)	139.6 (136.9, 142.4)	163.7 (160.5, 167.0)	166.1 (161.9, 170.4)	148.0 (147.0, 149.1)	114.4 (112.4, 116.3)	141.7 (140.5, 142.8)	164.8 (163.7, 165.9)	166.5 (165.2, 168.0)
urban-rural difference	1.6 (0.4, 2.7)	1.2 (-0.1, 2.4)	1.4 (0.3, 2.6)	1.7 (0.5, 3.0)	2.0 (0.6, 3.4)	0.5 (-0.1, 1.2)	0.1 (-0.8, 1.1)	0.4 (-0.3, 1.1)	0.7 (0.0, 1.4)	0.9 (0.0, 1.9)
**Central Asia, Middle East and north Africa**										
Central Asia										
Armenia										
rural	139.2 (137.3, 141.0)	112.2 (107.9, 116.4)	132.7 (130.3, 135.0)	153.9 (152.2, 155.6)	156.0 (154.3, 157.7)	141.9 (140.6, 143.3)	114.4 (111.3, 117.6)	135.3 (133.6, 137.0)	156.9 (155.5, 158.5)	159.3 (157.8, 160.9)
urban	141.7 (139.8, 143.6)	114.9 (110.6, 119.2)	135.3 (133.0, 137.6)	156.4 (154.7, 158.0)	158.3 (156.7, 160.1)	143.3 (141.9, 144.6)	115.9 (112.7, 119.1)	136.7 (135.0, 138.3)	158.2 (156.7, 159.7)	160.5 (158.9, 162.0)
urban-rural difference	2.6 (1.7, 3.5)	2.7 (1.4, 4.1)	2.6 (1.6, 3.6)	2.5 (1.7, 3.3)	2.4 (1.5, 3.3)	1.3 (0.5, 2.2)	1.5 (0.2, 2.8)	1.4 (0.5, 2.3)	1.3 (0.5, 2.0)	1.1 (0.3, 2.0)
Azerbaijan										
rural	139.6 (137.4, 141.9)	111.4 (106.5, 116.3)	133.0 (130.1, 135.7)	154.9 (152.9, 156.9)	157.2 (155.2, 159.1)	142.7 (140.0, 145.4)	114.4 (108.6, 120.1)	136.0 (132.8, 139.3)	158.0 (156.0, 159.9)	160.2 (158.4, 161.9)
urban	141.8 (139.5, 144.1)	114.0 (109.0, 119.0)	135.2 (132.4, 138.0)	156.9 (154.8, 159.0)	158.9 (156.9, 160.9)	143.6 (140.9, 146.3)	115.8 (110.0, 121.6)	137.1 (133.8, 140.3)	158.8 (156.8, 160.7)	160.7 (159.0, 162.5)
urban-rural difference	2.2 (1.1, 3.3)	2.6 (1.0, 4.2)	2.3 (1.1, 3.5)	2.0 (1.0, 3.0)	1.8 (0.7, 2.8)	1.0 (0.0, 1.9)	1.4 (-0.1, 2.9)	1.1 (0.0, 2.2)	0.8 (-0.1, 1.7)	0.5 (-0.4, 1.5)
Georgia										
rural	141.6 (138.8, 144.4)	113.6 (109.4, 117.9)	134.3 (131.2, 137.3)	156.9 (153.6, 160.3)	160.6 (156.5, 164.6)	144.0 (142.6, 145.4)	116.3 (113.3, 119.2)	136.8 (135.0, 138.5)	159.2 (157.2, 161.3)	162.8 (160.7, 164.9)
urban	144.1 (141.2, 146.9)	116.5 (112.2, 120.8)	136.9 (133.8, 140.0)	159.2 (155.9, 162.6)	162.7 (158.7, 166.7)	145.4 (144.0, 146.7)	118.1 (115.2, 121.1)	138.2 (136.5, 139.9)	160.4 (158.4, 162.4)	163.7 (161.7, 165.8)
urban-rural difference	2.5 (1.3, 3.7)	2.9 (1.5, 4.3)	2.6 (1.4, 3.9)	2.3 (1.1, 3.6)	2.1 (0.6, 3.5)	1.3 (0.4, 2.3)	1.8 (0.6, 3.0)	1.5 (0.5, 2.4)	1.2 (0.1, 2.2)	0.9 (-0.3, 2.2)
Kazakhstan										
rural	141.0 (139.2, 142.6)	109.6 (106.2, 113.1)	134.1 (132.1, 136.2)	157.7 (156.0, 159.4)	159.3 (157.4, 161.3)	145.4 (144.2, 146.5)	113.7 (111.6, 115.8)	138.7 (137.4, 140.0)	162.3 (160.8, 163.6)	163.4 (162.0, 164.9)
urban	143.3 (141.6, 145.0)	112.8 (109.3, 116.3)	136.7 (134.7, 138.7)	159.7 (157.9, 161.4)	160.9 (159.0, 162.9)	146.5 (145.3, 147.6)	115.6 (113.6, 117.8)	140.0 (138.7, 141.4)	163.0 (161.6, 164.3)	163.7 (162.4, 165.1)
urban-rural difference	2.3 (1.4, 3.2)	3.2 (1.9, 4.4)	2.6 (1.6, 3.5)	2.0 (1.1, 2.8)	1.6 (0.6, 2.6)	1.1 (0.4, 1.8)	1.9 (0.8, 3.0)	1.3 (0.5, 2.1)	0.7 (0.0, 1.5)	0.4 (-0.6, 1.3)
Kyrgyzstan										

Girls	Mean height in 1990 (cm)					Mean height in 2020 (cm)				
	Age-standardised	5 years	10 years	15 years	19 years	Age-standardised	5 years	10 years	15 years	19 years
rural	139.6 (137.7, 141.5)	111.0 (107.3, 114.6)	132.7 (130.4, 135.0)	155.1 (153.1, 157.0)	157.4 (155.5, 159.5)	142.4 (140.9, 144.1)	113.8 (110.9, 116.7)	135.6 (133.7, 137.5)	158.0 (156.3, 159.8)	160.5 (158.6, 162.4)
urban	141.9 (140.0, 143.8)	113.7 (110.1, 117.3)	135.1 (132.9, 137.4)	157.2 (155.3, 159.2)	159.4 (157.5, 161.4)	143.5 (141.9, 145.1)	115.2 (112.3, 118.1)	136.7 (134.8, 138.7)	158.9 (157.2, 160.7)	161.2 (159.3, 163.1)
urban-rural difference	2.3 (1.4, 3.3)	2.7 (1.4, 4.0)	2.4 (1.4, 3.5)	2.2 (1.2, 3.1)	2.0 (0.9, 3.0)	1.1 (0.2, 1.9)	1.4 (0.2, 2.6)	1.2 (0.2, 2.0)	0.9 (0.1, 1.8)	0.7 (-0.3, 1.7)
Mongolia										
rural	139.9 (137.3, 142.3)	110.9 (106.0, 116.0)	133.1 (130.3, 135.8)	155.7 (153.2, 158.2)	157.3 (154.2, 160.4)	142.9 (140.6, 145.2)	114.9 (109.4, 120.4)	136.4 (133.7, 139.3)	158.3 (156.8, 159.9)	159.3 (157.9, 160.8)
urban	142.4 (139.8, 144.8)	113.9 (108.9, 118.8)	135.7 (132.9, 138.5)	158.0 (155.5, 160.5)	159.5 (156.4, 162.6)	144.2 (141.9, 146.5)	116.7 (111.2, 122.2)	137.9 (135.1, 140.7)	159.4 (157.9, 161.0)	160.2 (158.9, 161.6)
urban-rural difference	2.5 (1.4, 3.7)	3.0 (1.3, 4.6)	2.6 (1.4, 3.9)	2.3 (1.3, 3.4)	2.2 (1.1, 3.2)	1.3 (0.3, 2.3)	1.7 (0.2, 3.3)	1.4 (0.3, 2.5)	1.1 (0.3, 2.0)	0.9 (0.1, 1.8)
Tajikistan										
rural	138.3 (136.1, 140.5)	109.5 (105.5, 113.4)	131.2 (128.7, 133.7)	153.9 (151.5, 156.3)	156.9 (154.0, 159.9)	140.3 (139.2, 141.4)	112.4 (109.6, 115.3)	133.4 (131.9, 134.9)	155.6 (154.2, 156.9)	158.2 (156.7, 159.7)
urban	140.4 (138.1, 142.7)	111.9 (107.9, 115.8)	133.4 (130.8, 136.0)	155.9 (153.4, 158.4)	158.8 (155.8, 161.9)	141.2 (140.0, 142.4)	113.5 (110.6, 116.5)	134.4 (132.8, 135.9)	156.4 (155.0, 157.8)	158.9 (157.4, 160.4)
urban-rural difference	2.1 (1.1, 3.2)	2.4 (1.0, 3.7)	2.2 (1.1, 3.3)	2.0 (1.0, 3.1)	1.9 (0.7, 3.1)	0.9 (0.1, 1.7)	1.1 (-0.1, 2.3)	1.0 (0.1, 1.8)	0.8 (0.1, 1.5)	0.7 (-0.2, 1.5)
Turkmenistan										
rural	139.6 (137.2, 142.0)	110.8 (106.7, 114.9)	132.3 (129.4, 135.3)	155.2 (152.6, 157.9)	158.9 (156.5, 161.3)	143.2 (141.5, 145.0)	114.1 (110.6, 117.7)	135.9 (133.7, 138.1)	159.0 (156.8, 161.1)	162.7 (160.8, 164.5)
urban	141.9 (139.5, 144.3)	113.5 (109.5, 117.6)	134.7 (131.8, 137.7)	157.4 (154.7, 160.1)	160.8 (158.4, 163.3)	144.3 (142.6, 146.1)	115.6 (112.1, 119.1)	137.1 (134.9, 139.2)	159.9 (157.8, 162.1)	163.4 (161.6, 165.2)
urban-rural difference	2.3 (1.0, 3.6)	2.7 (1.2, 4.3)	2.4 (1.1, 3.8)	2.1 (0.8, 3.5)	1.9 (0.4, 3.5)	1.1 (0.0, 2.2)	1.5 (0.1, 2.9)	1.2 (0.1, 2.3)	0.9 (-0.2, 2.1)	0.7 (-0.6, 2.0)
Uzbekistan										
rural	140.7 (138.5, 142.8)	111.6 (106.7, 116.5)	133.7 (131.0, 136.3)	156.4 (154.5, 158.2)	159.0 (157.3, 160.7)	143.2 (140.6, 145.9)	115.4 (109.7, 121.3)	136.7 (133.4, 140.0)	158.4 (156.3, 160.4)	160.2 (158.6, 161.8)
urban	142.6 (140.5, 144.7)	114.0 (109.2, 118.8)	135.8 (133.2, 138.4)	158.1 (156.2, 160.0)	160.5 (158.8, 162.2)	144.0 (141.3, 146.7)	116.6 (110.9, 122.5)	137.6 (134.2, 141.0)	158.9 (156.9, 161.0)	160.5 (158.8, 162.1)
urban-rural difference	1.9 (0.9, 2.9)	2.4 (0.9, 4.0)	2.1 (1.0, 3.2)	1.7 (0.9, 2.6)	1.5 (0.6, 2.4)	0.7 (-0.3, 1.8)	1.2 (-0.4, 2.8)	0.9 (-0.3, 2.0)	0.5 (-0.4, 1.5)	0.3 (-0.7, 1.3)
Middle East and north Africa										
Algeria										
rural	140.6 (137.3, 144.0)	107.8 (102.6, 113.2)	135.5 (131.5, 139.5)	156.0 (152.7, 159.4)	158.0 (154.7, 161.4)	144.4 (140.9, 147.8)	111.7 (105.7, 117.6)	139.3 (135.0, 143.5)	159.8 (156.9, 162.8)	161.7 (159.6, 163.8)
urban	142.5 (139.2, 145.9)	110.1 (105.0, 115.5)	137.5 (133.6, 141.4)	157.8 (154.5, 161.1)	159.6 (156.3, 163.0)	145.6 (142.2, 149.0)	113.4 (107.3, 119.4)	140.6 (136.4, 144.7)	160.9 (158.0, 163.8)	162.6 (160.6, 164.6)
urban-rural difference	1.9 (0.6, 3.3)	2.3 (0.7, 3.9)	2.0 (0.7, 3.4)	1.8 (0.5, 3.2)	1.6 (0.1, 3.1)	1.3 (0.0, 2.6)	1.6 (0.0, 3.3)	1.4 (0.0, 2.7)	1.1 (-0.1, 2.4)	0.9 (-0.4, 2.3)
Bahrain										
rural	140.2 (137.5, 143.0)	108.6 (103.9, 113.3)	136.0 (132.9, 139.1)	155.0 (152.3, 157.6)	155.3 (152.3, 158.3)	143.9 (141.3, 146.5)	113.6 (108.5, 118.7)	140.0 (137.2, 142.9)	158.1 (155.8, 160.4)	157.7 (155.2, 160.3)
urban	142.1 (139.7, 144.6)	111.0 (106.5, 115.6)	138.1 (135.2, 141.0)	156.6 (154.3, 158.9)	156.9 (154.2, 159.6)	145.2 (142.9, 147.4)	115.4 (110.5, 120.3)	141.5 (138.9, 144.1)	159.2 (157.2, 161.1)	158.6 (156.7, 160.8)
urban-rural difference	1.9 (0.5, 3.4)	2.4 (0.7, 4.0)	2.1 (0.6, 3.6)	1.7 (0.1, 3.2)	1.6 (-0.1, 3.3)	1.3 (-0.2, 2.8)	1.8 (0.0, 3.5)	1.4 (-0.1, 2.9)	1.0 (-0.5, 2.6)	0.9 (-0.7, 2.6)
Egypt										
rural	137.6 (136.0, 139.0)	104.2 (101.6, 106.7)	131.6 (129.9, 133.3)	153.3 (151.7, 155.0)	157.5 (155.6, 159.4)	142.1 (140.1, 144.2)	109.8 (105.7, 113.9)	136.4 (134.0, 139.0)	157.5 (156.1, 159.0)	161.1 (159.7, 162.5)
urban	139.2 (137.7, 140.8)	106.4 (103.8, 109.0)	133.4 (131.7, 135.2)	154.8 (153.1, 156.4)	158.6 (156.7, 160.5)	143.2 (141.2, 145.3)	111.4 (107.2, 115.6)	137.6 (135.2, 140.2)	158.3 (156.9, 159.8)	161.6 (160.1, 163.0)
urban-rural difference	1.7 (0.7, 2.7)	2.2 (0.7, 3.7)	1.8 (0.7, 2.9)	1.4 (0.5, 2.3)	1.1 (0.2, 2.0)	1.0 (0.1, 2.0)	1.6 (0.1, 3.1)	1.2 (0.1, 2.3)	0.8 (0.0, 1.6)	0.5 (-0.3, 1.3)
Iran										
rural	138.3 (137.4, 139.2)	105.8 (104.6, 106.9)	132.6 (131.7, 133.6)	154.1 (153.2, 155.1)	156.6 (155.3, 157.8)	141.4 (140.6, 142.3)	107.5 (105.7, 109.4)	135.5 (134.5, 136.5)	157.9 (157.1, 158.7)	160.5 (159.6, 161.3)
urban	140.8 (139.9, 141.7)	108.1 (107.0, 109.2)	135.0 (134.1, 135.9)	156.6 (155.7, 157.6)	159.1 (157.8, 160.3)	143.1 (142.2, 143.9)	109.1 (107.3, 110.9)	137.1 (136.1, 138.0)	159.5 (158.7, 160.3)	162.2 (161.3, 163.0)
urban-rural difference	2.4 (1.9, 3.0)	2.4 (1.7, 3.1)	2.4 (1.8, 3.0)	2.5 (1.9, 3.0)	2.5 (1.9, 3.1)	1.6 (1.2, 2.1)	1.6 (0.9, 2.2)	1.6 (1.1, 2.1)	1.7 (1.2, 2.1)	1.7 (1.2, 2.2)
Iraq										
rural	139.7 (136.8, 142.5)	107.1 (102.2, 111.9)	134.9 (131.7, 138.2)	155.0 (152.2, 157.9)	156.5 (153.5, 159.4)	142.0 (139.2, 144.9)	110.9 (105.2, 116.5)	137.6 (134.2, 141.0)	156.7 (154.3, 159.2)	157.4 (155.3, 159.5)
urban	142.0 (139.3, 144.7)	109.7 (105.0, 114.4)	137.3 (134.1, 140.5)	157.1 (154.4, 159.9)	158.5 (155.7, 161.4)	143.6 (140.8, 146.5)	112.8 (107.1, 118.3)	139.3 (135.9, 142.7)	158.2 (155.8, 160.6)	158.8 (156.8, 160.8)
urban-rural difference	2.3 (0.9, 3.6)	2.6 (0.9, 4.2)	2.4 (1.0, 3.7)	2.1 (0.7, 3.5)	2.0 (0.4, 3.5)	1.6 (0.4, 2.9)	1.9 (0.3, 3.6)	1.7 (0.4, 3.0)	1.5 (0.2, 2.8)	1.4 (0.0, 2.8)
Jordan										
rural	140.2 (138.7, 141.9)	108.3 (105.6, 111.1)	135.3 (133.5, 137.2)	155.4 (153.6, 157.2)	157.0 (155.3, 158.8)	143.4 (141.8, 145.0)	112.6 (109.8, 115.4)	138.7 (136.7, 140.6)	158.1 (156.5, 159.6)	159.3 (157.8, 160.7)
urban	141.8 (140.2, 143.4)	109.9 (107.3, 112.7)	136.9 (135.0, 138.8)	156.9 (155.1, 158.7)	158.5 (156.8, 160.3)	144.2 (142.6, 145.8)	113.6 (110.9, 116.2)	139.5 (137.7, 141.3)	158.8 (157.3, 160.4)	160.0 (158.7, 161.4)
urban-rural difference	1.6 (0.7, 2.5)	1.7 (0.4, 3.0)	1.6 (0.6, 2.6)	1.5 (0.7, 2.3)	1.5 (0.6, 2.4)	0.8 (0.0, 1.7)	0.9 (-0.4, 2.2)	0.9 (-0.1, 1.8)	0.8 (0.0, 1.6)	0.7 (-0.1, 1.6)
Kuwait										
rural	141.2 (139.3, 143.1)	108.3 (106.1, 110.5)	137.3 (135.4, 139.3)	155.9 (153.8, 158.1)	156.6 (154.0, 159.4)	143.4 (141.3, 145.6)	111.3 (107.8, 114.7)	139.8 (137.3, 142.2)	158.0 (156.0, 159.9)	158.3 (156.3, 160.4)
urban	143.1 (141.9, 144.4)	110.8 (109.3, 112.2)	139.5 (138.2, 140.7)	157.7 (156.2, 159.2)	158.2 (156.1, 160.3)	144.8 (143.1, 146.5)	113.1 (110.0, 116.2)	141.2 (139.2, 143.2)	159.1 (157.6, 160.4)	159.2 (157.9, 160.5)
urban-rural difference	2.0 (0.5, 3.5)	2.5 (0.8, 4.1)	2.1 (0.7, 3.6)	1.8 (0.2, 3.3)	1.5 (-0.3, 3.3)	1.3 (-0.2, 2.8)	1.8 (0.1, 3.5)	1.4 (-0.1, 2.9)	1.1 (-0.4, 2.5)	0.8 (-0.8, 2.5)
Lebanon										
rural	142.9 (141.2, 144.7)	108.2 (106.1, 110.3)	138.0 (136.1, 139.9)	159.1 (157.2, 161.1)	160.5 (158.5, 162.5)	146.3 (144.1, 148.6)	112.6 (108.9, 116.4)	141.6 (138.9, 144.2)	162.1 (160.1, 164.2)	163.0 (161.0, 165.0)
urban	144.6 (142.9, 146.3)	110.1 (108.0, 112.1)	139.7 (137.9, 141.6)	160.7 (158.8, 162.7)	162.0 (160.0, 164.1)	147.4 (145.1, 149.6)	113.8 (110.1, 117.5)	142.7 (140.1, 145.3)	163.1 (161.1, 165.1)	163.9 (161.9, 165.8)

Girls	Mean height in 1990 (cm)					Mean height in 2020 (cm)				
	Age-standardised	5 years	10 years	15 years	19 years	Age-standardised	5 years	10 years	15 years	19 years
urban-rural difference	1.7 (0.8, 2.6)	1.9 (0.6, 3.0)	1.7 (0.8, 2.6)	1.6 (0.7, 2.5)	1.5 (0.5, 2.6)	1.0 (0.1, 2.0)	1.2 (-0.1, 2.5)	1.1 (0.1, 2.1)	1.0 (0.0, 1.9)	0.9 (-0.2, 2.0)
Libya										
rural	140.7 (137.2, 144.3)	107.6 (102.2, 113.1)	135.4 (131.2, 139.5)	156.3 (152.4, 159.9)	159.1 (155.1, 163.1)	143.8 (139.8, 148.0)	111.2 (104.8, 117.6)	138.6 (133.9, 143.3)	159.2 (155.5, 163.1)	161.8 (158.3, 165.4)
urban	142.7 (139.2, 146.2)	109.9 (104.6, 115.3)	137.4 (133.4, 141.5)	158.0 (154.5, 161.7)	160.6 (156.8, 164.5)	145.1 (141.1, 149.1)	112.8 (106.5, 119.1)	139.9 (135.3, 144.6)	160.3 (156.7, 164.1)	162.7 (159.4, 166.0)
urban-rural difference	1.9 (0.4, 3.4)	2.3 (0.7, 4.0)	2.0 (0.5, 3.5)	1.8 (0.2, 3.3)	1.5 (-0.2, 3.3)	1.3 (-0.3, 2.7)	1.7 (0.0, 3.4)	1.4 (-0.2, 2.9)	1.1 (-0.5, 2.6)	0.9 (-0.9, 2.6)
Morocco										
rural	139.3 (137.1, 141.6)	107.3 (103.9, 110.9)	134.2 (131.7, 136.7)	154.4 (151.9, 156.9)	156.9 (154.1, 159.8)	141.9 (140.5, 143.3)	109.7 (107.3, 112.1)	136.6 (135.0, 138.2)	157.1 (155.6, 158.8)	159.9 (158.1, 161.7)
urban	141.7 (139.4, 143.9)	110.2 (106.8, 113.8)	136.7 (134.2, 139.1)	156.5 (154.0, 159.1)	158.7 (155.9, 161.5)	143.7 (142.3, 145.1)	112.0 (109.6, 114.4)	138.5 (136.9, 140.2)	158.7 (157.1, 160.3)	161.1 (159.4, 162.9)
urban-rural difference	2.3 (1.3, 3.3)	2.9 (1.5, 4.2)	2.5 (1.5, 3.5)	2.1 (1.1, 3.0)	1.8 (0.7, 2.9)	1.8 (0.9, 2.7)	2.3 (1.0, 3.6)	1.9 (1.0, 2.9)	1.5 (0.7, 2.4)	1.2 (0.3, 2.2)
Occupied Palestinian Territory										
rural	140.5 (138.2, 142.8)	107.7 (103.3, 112.2)	135.4 (132.8, 138.1)	155.9 (153.7, 158.0)	157.9 (155.9, 160.0)	143.8 (141.0, 146.7)	111.6 (106.0, 117.3)	138.9 (135.6, 142.3)	159.1 (156.9, 161.3)	160.7 (158.5, 163.0)
urban	142.4 (140.3, 144.6)	110.1 (105.8, 114.5)	137.4 (134.9, 140.1)	157.6 (155.5, 159.7)	159.4 (157.5, 161.4)	145.1 (142.4, 147.9)	113.3 (107.6, 119.0)	140.3 (137.1, 143.6)	160.2 (158.1, 162.3)	161.6 (159.5, 163.8)
urban-rural difference	1.9 (0.7, 3.1)	2.3 (0.8, 3.9)	2.0 (0.8, 3.3)	1.7 (0.5, 2.9)	1.5 (0.2, 2.8)	1.3 (0.0, 2.6)	1.7 (0.0, 3.4)	1.4 (0.0, 2.7)	1.1 (-0.2, 2.4)	0.8 (-0.6, 2.2)
Oman										
rural	137.1 (134.5, 139.8)	104.0 (99.7, 108.5)	131.8 (128.9, 134.6)	152.7 (149.7, 155.7)	155.5 (151.9, 159.3)	140.6 (138.2, 143.1)	108.8 (103.7, 114.2)	135.6 (132.7, 138.6)	155.7 (153.8, 157.5)	157.8 (155.7, 159.8)
urban	138.8 (136.3, 141.5)	106.2 (101.9, 110.5)	133.6 (130.9, 136.4)	154.2 (151.4, 157.2)	156.8 (153.2, 160.5)	141.7 (139.4, 144.1)	110.3 (105.3, 115.7)	136.8 (134.0, 139.7)	156.6 (154.9, 158.2)	158.4 (156.6, 160.1)
urban-rural difference	1.7 (0.4, 3.0)	2.1 (0.6, 3.8)	1.8 (0.5, 3.2)	1.5 (0.2, 2.8)	1.3 (-0.2, 2.7)	1.1 (-0.2, 2.3)	1.5 (-0.2, 3.2)	1.2 (-0.2, 2.5)	0.9 (-0.4, 2.1)	0.6 (-0.7, 2.0)
Qatar										
rural	140.4 (137.3, 143.5)	107.7 (102.8, 112.6)	135.4 (132.0, 138.8)	155.7 (152.4, 159.1)	157.5 (153.6, 161.3)	143.5 (140.4, 146.6)	111.4 (105.8, 117.2)	138.6 (135.0, 142.4)	158.6 (155.9, 161.3)	160.0 (157.4, 162.7)
urban	142.3 (139.4, 145.2)	110.0 (105.5, 114.8)	137.5 (134.2, 140.7)	157.5 (154.4, 160.6)	159.0 (155.6, 162.5)	144.7 (141.8, 147.7)	113.0 (107.4, 118.8)	140.0 (136.5, 143.7)	159.7 (157.3, 162.1)	160.9 (158.8, 163.0)
urban-rural difference	1.9 (0.4, 3.3)	2.3 (0.7, 4.0)	2.0 (0.5, 3.5)	1.8 (0.2, 3.3)	1.5 (-0.2, 3.3)	1.3 (-0.2, 2.7)	1.7 (-0.1, 3.3)	1.4 (-0.1, 2.9)	1.1 (-0.4, 2.6)	0.9 (-0.8, 2.5)
Saudi Arabia										
rural	136.5 (135.1, 138.0)	105.2 (103.1, 107.2)	131.8 (130.2, 133.5)	151.2 (149.6, 152.9)	153.0 (150.9, 154.9)	141.2 (139.0, 143.4)	109.0 (105.0, 113.0)	136.1 (133.6, 138.6)	156.3 (154.5, 158.2)	158.6 (156.8, 160.5)
urban	137.9 (136.7, 139.1)	106.9 (105.1, 108.7)	133.3 (132.0, 134.7)	152.4 (151.1, 153.8)	154.0 (152.2, 155.6)	141.8 (139.8, 143.9)	110.1 (106.3, 113.9)	136.9 (134.5, 139.2)	156.8 (155.2, 158.5)	158.9 (157.3, 160.5)
urban-rural difference	1.4 (0.2, 2.5)	1.8 (0.3, 3.2)	1.5 (0.3, 2.7)	1.2 (0.0, 2.4)	1.0 (-0.3, 2.3)	0.7 (-0.5, 1.8)	1.0 (-0.5, 2.6)	0.8 (-0.4, 1.9)	0.5 (-0.6, 1.6)	0.3 (-0.9, 1.5)
Syrian Arab Republic										
rural	139.4 (134.1, 144.5)	106.6 (100.7, 112.5)	134.3 (129.2, 139.3)	154.8 (149.2, 160.3)	157.0 (149.6, 164.4)	142.7 (137.3, 147.9)	110.3 (103.5, 117.2)	137.7 (132.2, 143.0)	157.9 (152.4, 163.1)	159.8 (153.0, 166.9)
urban	141.3 (136.1, 146.4)	108.9 (103.0, 114.8)	136.3 (131.2, 141.3)	156.5 (150.9, 162.0)	158.5 (151.1, 166.0)	143.9 (138.4, 149.0)	112.0 (105.2, 118.8)	139.0 (133.5, 144.2)	159.0 (153.5, 164.3)	160.7 (154.0, 167.7)
urban-rural difference	1.9 (0.4, 3.4)	2.3 (0.6, 4.0)	2.0 (0.5, 3.5)	1.7 (0.2, 3.3)	1.5 (-0.2, 3.4)	1.3 (-0.2, 2.7)	1.6 (-0.1, 3.4)	1.4 (-0.1, 2.9)	1.1 (-0.5, 2.6)	0.9 (-0.8, 2.6)
Tunisia										
rural	140.9 (139.2, 142.6)	107.1 (105.1, 109.1)	136.2 (134.4, 138.0)	156.9 (155.0, 158.7)	157.5 (155.6, 159.4)	144.3 (142.0, 146.6)	110.3 (106.7, 113.9)	139.5 (136.9, 142.1)	160.4 (158.1, 162.5)	161.0 (158.9, 163.2)
urban	143.2 (141.5, 144.8)	109.9 (108.0, 111.8)	138.6 (136.8, 140.4)	158.9 (157.0, 160.7)	159.3 (157.4, 161.3)	145.9 (143.6, 148.1)	112.4 (108.9, 115.8)	141.2 (138.7, 143.7)	161.8 (159.7, 163.9)	162.2 (160.1, 164.3)
urban-rural difference	2.2 (1.4, 3.1)	2.7 (1.6, 3.9)	2.4 (1.5, 3.3)	2.0 (1.2, 2.9)	1.8 (0.9, 2.8)	1.6 (0.6, 2.6)	2.1 (0.7, 3.4)	1.7 (0.7, 2.8)	1.4 (0.4, 2.3)	1.2 (0.2, 2.2)
Turkey										
rural	139.5 (137.8, 141.2)	108.4 (105.3, 111.4)	134.6 (132.6, 136.7)	154.4 (152.6, 156.1)	155.7 (153.9, 157.6)	143.6 (142.3, 145.0)	111.6 (109.2, 114.1)	138.4 (136.8, 140.1)	158.9 (157.5, 160.3)	160.8 (159.3, 162.4)
urban	141.6 (140.0, 143.3)	110.9 (107.9, 114.0)	136.9 (134.8, 139.0)	156.3 (154.6, 158.0)	157.4 (155.5, 159.2)	145.2 (143.9, 146.4)	113.6 (111.3, 115.9)	140.1 (138.5, 141.7)	160.3 (158.9, 161.6)	161.9 (160.5, 163.4)
urban-rural difference	2.1 (1.2, 2.9)	2.6 (1.3, 3.8)	2.2 (1.3, 3.1)	1.9 (1.1, 2.7)	1.7 (0.8, 2.5)	1.5 (0.8, 2.3)	2.0 (0.9, 3.1)	1.7 (0.9, 2.5)	1.4 (0.7, 2.1)	1.1 (0.3, 1.9)
United Arab Emirates										
rural	141.8 (139.2, 144.5)	110.0 (105.9, 114.2)	137.2 (134.6, 139.8)	156.6 (153.6, 159.7)	158.3 (154.4, 162.3)	144.6 (142.3, 147.0)	113.8 (108.7, 118.7)	140.2 (137.5, 143.0)	159.1 (157.2, 160.9)	160.3 (158.3, 162.4)
urban	143.6 (141.2, 146.2)	112.4 (108.3, 116.4)	139.1 (136.8, 141.6)	158.2 (155.4, 161.2)	159.6 (155.9, 163.5)	145.8 (143.7, 148.0)	115.5 (110.6, 120.4)	141.6 (139.0, 144.1)	160.0 (158.5, 161.7)	160.9 (159.2, 162.8)
urban-rural difference	1.8 (0.5, 3.2)	2.4 (0.7, 4.0)	2.0 (0.6, 3.3)	1.6 (0.3, 3.0)	1.3 (-0.2, 2.8)	1.2 (-0.1, 2.6)	1.7 (0.0, 3.4)	1.3 (0.0, 2.8)	1.0 (-0.4, 2.3)	0.7 (-0.8, 2.1)
Yemen										
rural	131.9 (130.1, 133.7)	97.8 (95.4, 100.3)	125.2 (123.2, 127.2)	148.1 (146.2, 150.2)	153.6 (151.2, 156.0)	135.2 (133.1, 137.5)	103.0 (99.4, 106.9)	129.0 (126.3, 131.6)	150.8 (148.9, 152.6)	155.2 (153.4, 157.0)
urban	133.3 (131.5, 135.1)	99.6 (97.2, 102.0)	126.7 (124.8, 128.8)	149.4 (147.4, 151.5)	154.6 (152.2, 157.1)	136.0 (133.8, 138.3)	104.1 (100.5, 108.0)	129.9 (127.2, 132.6)	151.4 (149.5, 153.3)	155.7 (153.8, 157.5)
urban-rural difference	1.4 (0.5, 2.3)	1.8 (0.6, 2.9)	1.5 (0.6, 2.5)	1.3 (0.4, 2.1)	1.1 (0.1, 2.0)	0.8 (0.0, 1.6)	1.1 (0.0, 2.3)	0.9 (0.0, 1.7)	0.6 (-0.1, 1.4)	0.4 (-0.4, 1.3)
**East and southeast Asia**										
East Asia										
China										
rural	138.4 (137.7, 139.0)	104.4 (103.7, 105.2)	133.6 (132.9, 134.2)	153.9 (153.2, 154.6)	156.5 (155.6, 157.3)	147.1 (146.2, 148.0)	114.4 (112.6, 116.2)	143.0 (142.0, 144.0)	162.0 (161.2, 162.9)	163.2 (162.3, 164.1)
urban	141.5 (140.8, 142.1)	108.3 (107.5, 109.1)	136.9 (136.2, 137.5)	156.7 (156.0, 157.4)	158.8 (158.0, 159.7)	148.9 (148.1, 149.8)	117.0 (115.1, 118.7)	145.0 (144.0, 146.0)	163.6 (162.8, 164.4)	164.3 (163.4, 165.2)
urban-rural difference	3.1 (2.7, 3.5)	3.8 (3.3, 4.3)	3.3 (2.9, 3.7)	2.8 (2.4, 3.2)	2.4 (1.9, 2.9)	1.8 (1.3, 2.4)	2.6 (1.9, 3.2)	2.0 (1.5, 2.6)	1.5 (1.0, 2.0)	1.1 (0.5, 1.6)

Girls	Mean height in 1990 (cm)					Mean height in 2020 (cm)				
	Age-standardised	5 years	10 years	15 years	19 years	Age-standardised	5 years	10 years	15 years	19 years
North Korea										
rural	139.9 (133.9, 145.9)	105.9 (99.2, 112.9)	135.8 (129.9, 141.7)	155.0 (148.4, 161.4)	156.9 (148.6, 165.6)	144.6 (138.6, 150.7)	111.7 (104.2, 119.1)	141.0 (134.8, 147.0)	159.3 (152.9, 165.5)	160.4 (152.6, 168.5)
urban	142.2 (136.2, 148.2)	108.6 (101.7, 115.3)	138.2 (132.3, 144.2)	157.1 (150.4, 163.6)	158.9 (150.5, 167.7)	145.7 (139.6, 151.7)	113.2 (105.6, 120.6)	142.1 (135.9, 148.1)	160.1 (153.7, 166.4)	161.0 (153.4, 169.2)
urban-rural difference	2.3 (0.6, 4.0)	2.7 (0.9, 4.5)	2.4 (0.8, 4.1)	2.1 (0.3, 3.9)	2.0 (-0.1, 4.0)	1.0 (-0.6, 2.7)	1.4 (-0.5, 3.3)	1.1 (-0.6, 2.8)	0.8 (-0.9, 2.5)	0.7 (-1.2, 2.5)
Taiwan										
rural	142.9 (141.1, 144.6)	107.7 (105.7, 109.6)	139.2 (137.5, 141.0)	158.3 (156.2, 160.5)	159.5 (156.9, 162.3)	145.7 (144.4, 147.1)	112.6 (110.3, 114.9)	142.7 (141.2, 144.3)	160.2 (158.9, 161.4)	160.2 (158.7, 161.8)
urban	144.4 (142.8, 146.1)	109.1 (107.3, 110.9)	140.8 (139.2, 142.4)	159.9 (157.9, 161.9)	161.1 (158.6, 163.8)	145.8 (144.6, 147.1)	112.6 (110.4, 115.0)	142.8 (141.3, 144.3)	160.3 (159.2, 161.5)	160.4 (159.0, 161.9)
urban-rural difference	1.5 (0.4, 2.6)	1.5 (0.1, 2.8)	1.5 (0.4, 2.6)	1.5 (0.4, 2.7)	1.6 (0.3, 2.9)	0.1 (-0.8, 1.0)	0.1 (-1.4, 1.3)	0.1 (-0.9, 1.0)	0.1 (-0.8, 1.0)	0.2 (-0.8, 1.2)
Southeast Asia										
Brunei Darussalam										
rural	135.6 (133.0, 138.3)	103.3 (100.1, 106.5)	131.6 (129.1, 134.3)	150.5 (147.3, 153.7)	151.0 (147.0, 154.9)	139.5 (137.8, 141.2)	107.6 (104.3, 111.1)	135.7 (133.8, 137.7)	154.0 (152.5, 155.4)	154.1 (152.6, 155.8)
urban	137.4 (134.8, 140.0)	105.7 (102.6, 108.9)	133.6 (131.1, 136.2)	151.9 (148.9, 155.1)	152.2 (148.2, 156.1)	140.5 (139.0, 142.1)	109.3 (106.0, 112.7)	137.0 (135.2, 138.8)	154.7 (153.4, 156.1)	154.6 (153.3, 156.1)
urban-rural difference	1.8 (0.7, 2.9)	2.4 (1.0, 3.8)	2.0 (0.8, 3.1)	1.5 (0.3, 2.6)	1.2 (-0.1, 2.5)	1.0 (0.0, 2.1)	1.7 (0.3, 3.1)	1.2 (0.1, 2.3)	0.7 (-0.3, 1.8)	0.5 (-0.6, 1.6)
Cambodia										
rural	134.6 (132.7, 136.6)	101.4 (97.1, 105.5)	129.7 (127.4, 131.9)	150.1 (148.2, 151.9)	152.4 (150.3, 154.5)	138.3 (135.8, 140.8)	106.4 (100.9, 111.8)	133.8 (130.8, 136.8)	153.1 (151.3, 154.9)	154.5 (152.9, 156.2)
urban	136.8 (134.8, 138.8)	104.2 (99.9, 108.4)	132.0 (129.6, 134.2)	151.9 (150.0, 153.7)	153.9 (151.8, 156.0)	139.8 (137.3, 142.3)	108.6 (103.0, 114.0)	135.5 (132.5, 138.5)	154.3 (152.5, 156.1)	155.3 (153.7, 157.0)
urban-rural difference	2.1 (1.3, 3.0)	2.8 (1.4, 4.2)	2.3 (1.3, 3.3)	1.8 (1.1, 2.6)	1.4 (0.6, 2.3)	1.5 (0.6, 2.4)	2.1 (0.7, 3.6)	1.7 (0.6, 2.7)	1.2 (0.4, 1.9)	0.8 (0.0, 1.6)
Indonesia										
rural	132.9 (131.7, 134.0)	101.3 (100.0, 102.5)	127.8 (126.6, 128.9)	147.9 (146.6, 149.2)	149.8 (148.3, 151.2)	137.1 (136.1, 138.2)	106.0 (104.1, 107.8)	132.4 (131.2, 133.6)	151.9 (151.0, 152.9)	153.2 (152.2, 154.3)
urban	135.5 (134.3, 136.6)	104.3 (103.1, 105.6)	130.5 (129.3, 131.7)	150.3 (149.1, 151.6)	152.0 (150.5, 153.4)	139.1 (138.0, 140.1)	108.3 (106.5, 110.2)	134.4 (133.3, 135.6)	153.7 (152.7, 154.6)	154.7 (153.7, 155.7)
urban-rural difference	2.6 (2.1, 3.1)	3.1 (2.4, 3.7)	2.7 (2.2, 3.3)	2.4 (1.8, 3.0)	2.2 (1.5, 2.8)	1.9 (1.4, 2.4)	2.4 (1.7, 3.0)	2.0 (1.5, 2.6)	1.7 (1.2, 2.2)	1.5 (0.9, 2.1)
Lao PDR										
rural	134.1 (131.3, 137.0)	101.5 (97.0, 106.1)	129.6 (126.4, 132.8)	149.3 (146.3, 152.3)	150.6 (147.3, 154.0)	137.5 (134.3, 140.6)	106.1 (100.3, 112.0)	133.4 (129.6, 137.0)	152.0 (149.4, 154.5)	152.5 (150.5, 154.6)
urban	136.9 (134.1, 139.8)	104.7 (100.3, 109.3)	132.5 (129.3, 135.7)	151.9 (148.9, 154.9)	153.0 (149.6, 156.4)	139.5 (136.4, 142.7)	108.6 (102.8, 114.5)	135.6 (131.8, 139.3)	153.9 (151.4, 156.4)	154.2 (152.1, 156.4)
urban-rural difference	2.8 (1.6, 3.9)	3.2 (1.7, 4.7)	2.9 (1.7, 4.1)	2.6 (1.4, 3.7)	2.4 (1.1, 3.6)	2.1 (0.9, 3.3)	2.5 (1.0, 4.1)	2.2 (1.0, 3.5)	1.9 (0.7, 3.0)	1.7 (0.4, 2.9)
Malaysia										
rural	138.3 (137.0, 139.6)	106.0 (104.2, 107.9)	134.4 (132.9, 135.8)	153.0 (151.6, 154.4)	153.7 (152.2, 155.3)	140.5 (139.4, 141.5)	108.5 (107.0, 110.1)	136.7 (135.5, 137.8)	155.0 (153.9, 156.0)	155.6 (154.4, 156.8)
urban	140.6 (139.4, 141.9)	108.7 (106.9, 110.5)	136.8 (135.4, 138.3)	155.1 (153.7, 156.5)	155.7 (154.2, 157.2)	142.1 (141.0, 143.1)	110.5 (108.9, 112.0)	138.4 (137.2, 139.5)	156.4 (155.4, 157.4)	156.7 (155.7, 157.9)
urban-rural difference	2.3 (1.7, 3.0)	2.7 (1.9, 3.6)	2.4 (1.8, 3.1)	2.1 (1.5, 2.8)	1.9 (1.2, 2.7)	1.6 (1.1, 2.1)	2.0 (1.2, 2.7)	1.7 (1.2, 2.2)	1.4 (0.9, 1.9)	1.2 (0.6, 1.8)
Maldives										
rural	134.8 (132.5, 137.1)	102.5 (97.9, 107.0)	130.4 (127.6, 133.2)	149.9 (147.7, 152.0)	150.5 (148.2, 152.9)	138.9 (136.6, 141.3)	107.2 (101.9, 112.6)	134.9 (132.0, 137.7)	153.7 (152.0, 155.4)	153.9 (152.3, 155.6)
urban	137.1 (134.7, 139.4)	105.4 (100.8, 110.0)	132.9 (130.1, 135.7)	151.8 (149.7, 154.0)	152.2 (149.9, 154.5)	140.5 (138.1, 142.8)	109.4 (104.2, 114.7)	136.6 (133.7, 139.5)	155.0 (153.3, 156.7)	154.9 (153.4, 156.5)
urban-rural difference	2.3 (1.2, 3.4)	2.9 (1.4, 4.4)	2.4 (1.3, 3.7)	2.0 (0.9, 3.1)	1.7 (0.5, 2.9)	1.6 (0.6, 2.6)	2.2 (0.7, 3.8)	1.8 (0.6, 2.9)	1.3 (0.3, 2.3)	1.0 (0.0, 2.1)
Myanmar										
rural	135.0 (132.5, 137.5)	102.0 (97.5, 106.5)	130.2 (127.4, 133.0)	150.3 (147.7, 153.0)	152.6 (149.5, 155.8)	138.2 (135.7, 140.6)	106.2 (100.7, 111.4)	133.7 (130.7, 136.6)	152.9 (151.2, 154.6)	154.5 (152.9, 156.2)
urban	137.0 (134.4, 139.6)	104.7 (100.2, 109.2)	132.3 (129.5, 135.2)	152.0 (149.4, 154.6)	153.8 (150.7, 157.1)	139.5 (137.0, 141.9)	108.3 (102.8, 113.6)	135.3 (132.3, 138.1)	153.9 (152.2, 155.6)	155.1 (153.5, 156.8)
urban-rural difference	2.0 (0.9, 3.0)	2.7 (1.2, 4.2)	2.2 (1.0, 3.3)	1.6 (0.7, 2.6)	1.2 (0.2, 2.2)	1.3 (0.4, 2.3)	2.1 (0.6, 3.6)	1.5 (0.4, 2.6)	1.0 (0.1, 1.8)	0.6 (-0.3, 1.5)
Philippines										
rural	133.6 (132.2, 135.0)	101.3 (99.8, 102.8)	129.1 (127.7, 130.5)	148.5 (146.9, 150.1)	149.8 (147.6, 151.9)	137.2 (136.2, 138.3)	105.1 (103.3, 106.9)	132.9 (131.8, 134.0)	152.1 (151.2, 153.0)	153.2 (152.2, 154.4)
urban	135.8 (134.5, 137.2)	104.3 (102.8, 105.8)	131.6 (130.2, 132.9)	150.4 (148.8, 152.0)	151.3 (149.1, 153.4)	138.9 (137.9, 139.9)	107.5 (105.6, 109.3)	134.7 (133.6, 135.9)	153.4 (152.4, 154.4)	154.1 (153.0, 155.2)
urban-rural difference	2.2 (1.6, 2.9)	3.0 (2.2, 3.8)	2.4 (1.7, 3.1)	1.9 (1.2, 2.6)	1.5 (0.7, 2.3)	1.6 (1.1, 2.2)	2.3 (1.7, 3.0)	1.8 (1.3, 2.4)	1.3 (0.8, 1.9)	0.9 (0.3, 1.6)
Thailand										
rural	137.7 (136.7, 138.9)	104.5 (103.2, 105.8)	133.1 (132.0, 134.3)	153.2 (152.0, 154.5)	154.4 (153.1, 155.7)	143.6 (142.5, 144.8)	111.3 (109.0, 113.7)	139.5 (138.2, 140.8)	158.6 (157.6, 159.6)	158.8 (157.8, 160.0)
urban	139.8 (138.7, 141.0)	107.3 (106.0, 108.7)	135.4 (134.2, 136.6)	155.0 (153.8, 156.3)	155.8 (154.5, 157.2)	144.9 (143.8, 146.1)	113.3 (111.1, 115.8)	141.0 (139.7, 142.3)	159.6 (158.5, 160.6)	159.5 (158.4, 160.6)
urban-rural difference	2.1 (1.5, 2.7)	2.8 (2.0, 3.6)	2.3 (1.7, 2.9)	1.8 (1.2, 2.4)	1.4 (0.7, 2.2)	1.3 (0.6, 2.0)	2.0 (1.1, 2.9)	1.5 (0.7, 2.2)	1.0 (0.3, 1.7)	0.6 (-0.1, 1.4)
Timor-Leste										
rural	131.6 (129.1, 134.1)	97.9 (94.9, 100.9)	126.2 (123.8, 128.7)	147.2 (144.2, 150.2)	150.5 (146.9, 154.3)	135.1 (133.7, 136.5)	103.1 (100.9, 105.4)	130.3 (128.7, 131.9)	149.9 (148.6, 151.3)	152.4 (150.9, 153.8)
urban	134.1 (131.7, 136.7)	101.2 (98.0, 104.3)	129.0 (126.5, 131.5)	149.5 (146.6, 152.5)	152.4 (148.8, 156.3)	137.0 (135.5, 138.5)	105.7 (103.1, 108.3)	132.4 (130.6, 134.2)	151.5 (150.1, 153.0)	153.6 (152.0, 155.1)
urban-rural difference	2.6 (1.5, 3.6)	3.2 (1.7, 4.6)	2.8 (1.6, 3.8)	2.3 (1.3, 3.2)	1.9 (0.8, 3.0)	1.9 (0.9, 2.8)	2.5 (1.0, 4.0)	2.1 (1.0, 3.1)	1.6 (0.8, 2.4)	1.2 (0.3, 2.1)
Viet Nam										
rural	133.4 (132.5, 134.4)	100.5 (99.3, 101.7)	127.8 (126.7, 128.9)	149.0 (147.9, 150.1)	152.6 (151.3, 153.8)	141.1 (139.8, 142.5)	109.0 (106.3, 111.9)	136.1 (134.5, 137.8)	156.1 (155.0, 157.2)	158.6 (157.4, 159.8)

Girls	Mean height in 1990 (cm)					Mean height in 2020 (cm)				
	Age-standardised	5 years	10 years	15 years	19 years	Age-standardised	5 years	10 years	15 years	19 years
urban	136.4 (135.4, 137.4)	104.8 (103.6, 106.0)	131.1 (130.0, 132.2)	151.3 (150.2, 152.5)	154.2 (152.9, 155.4)	143.3 (142.0, 144.8)	112.6 (109.8, 115.4)	138.7 (137.1, 140.4)	157.8 (156.7, 158.9)	159.5 (158.3, 160.7)
urban-rural difference	2.9 (2.4, 3.5)	4.3 (3.6, 5.0)	3.3 (2.8, 3.9)	2.4 (1.8, 2.9)	1.6 (1.0, 2.2)	2.2 (1.6, 2.8)	3.6 (2.7, 4.4)	2.6 (2.0, 3.2)	1.6 (1.0, 2.3)	0.9 (0.2, 1.6)
**High-income Asia Pacific**										
Japan										
rural	142.2 (141.8, 142.5)	108.9 (108.5, 109.4)	139.2 (138.8, 139.5)	156.4 (156.0, 156.8)	157.1 (156.7, 157.6)	142.8 (142.3, 143.2)	109.6 (108.9, 110.4)	139.8 (139.3, 140.3)	157.0 (156.6, 157.4)	157.7 (157.2, 158.2)
urban	142.3 (142.0, 142.6)	108.8 (108.4, 109.2)	139.2 (138.8, 139.6)	156.7 (156.3, 157.0)	157.6 (157.1, 158.0)	143.0 (142.6, 143.4)	109.6 (108.9, 110.3)	140.0 (139.5, 140.4)	157.4 (157.0, 157.8)	158.2 (157.8, 158.7)
urban-rural difference	0.1 (-0.1, 0.4)	-0.2 (-0.4, 0.1)	0.0 (-0.2, 0.3)	0.3 (0.0, 0.5)	0.4 (0.1, 0.8)	0.2 (0.0, 0.5)	0.0 (-0.4, 0.3)	0.2 (-0.1, 0.4)	0.4 (0.1, 0.7)	0.5 (0.2, 0.9)
Singapore										
rural	143.1 (139.2, 147.0)	109.2 (103.3, 115.2)	139.8 (135.3, 144.4)	157.6 (153.6, 161.4)	159.3 (155.4, 163.2)	144.7 (140.7, 148.9)	111.1 (104.3, 118.0)	141.5 (136.6, 146.5)	159.0 (155.5, 162.6)	160.6 (157.9, 163.3)
urban	143.8 (140.1, 147.5)	110.0 (104.1, 115.9)	140.5 (136.1, 145.0)	158.2 (154.6, 161.7)	159.9 (156.6, 163.3)	145.3 (141.5, 149.3)	111.9 (105.0, 118.7)	142.1 (137.4, 147.0)	159.6 (156.4, 162.8)	161.2 (159.2, 163.1)
urban-rural difference	0.7 (-0.9, 2.3)	0.8 (-0.9, 2.6)	0.7 (-0.9, 2.3)	0.7 (-1.1, 2.4)	0.6 (-1.4, 2.6)	0.7 (-0.9, 2.3)	0.7 (-1.1, 2.6)	0.7 (-0.9, 2.3)	0.6 (-1.1, 2.3)	0.6 (-1.3, 2.5)
South Korea										
rural	142.9 (142.0, 143.8)	109.7 (108.7, 110.7)	140.1 (139.2, 141.1)	157.1 (156.1, 158.1)	157.5 (156.2, 158.7)	146.2 (145.6, 146.9)	112.0 (111.0, 113.0)	143.0 (142.4, 143.7)	160.9 (160.3, 161.6)	162.0 (161.3, 162.7)
urban	143.5 (142.6, 144.3)	110.3 (109.3, 111.2)	140.7 (139.8, 141.6)	157.7 (156.7, 158.6)	158.0 (156.9, 159.2)	146.8 (146.2, 147.4)	112.6 (111.6, 113.5)	143.6 (143.0, 144.3)	161.5 (160.9, 162.1)	162.6 (161.9, 163.3)
urban-rural difference	0.6 (0.0, 1.1)	0.6 (-0.1, 1.3)	0.6 (0.0, 1.2)	0.6 (0.0, 1.1)	0.6 (-0.1, 1.2)	0.6 (0.2, 1.0)	0.6 (0.1, 1.1)	0.6 (0.2, 1.0)	0.6 (0.2, 1.0)	0.6 (0.1, 1.1)
**High-income western**										
High-income English-speaking countries										
Australia										
rural	146.6 (145.6, 147.6)	111.7 (110.5, 112.9)	142.1 (141.1, 143.1)	162.9 (161.7, 164.0)	163.4 (161.9, 165.0)	148.6 (147.5, 149.6)	114.1 (111.9, 116.4)	144.2 (142.9, 145.5)	164.6 (163.8, 165.5)	165.0 (164.2, 166.0)
urban	146.6 (145.7, 147.5)	112.0 (110.9, 113.2)	142.2 (141.1, 143.2)	162.6 (161.5, 163.8)	163.0 (161.5, 164.6)	148.6 (147.6, 149.6)	114.5 (112.3, 116.7)	144.3 (143.1, 145.5)	164.4 (163.6, 165.3)	164.7 (163.9, 165.5)
urban-rural difference	0.0 (-0.7, 0.6)	0.3 (-0.4, 1.1)	0.1 (-0.6, 0.7)	-0.2 (-0.9, 0.5)	-0.4 (-1.2, 0.4)	0.0 (-0.5, 0.5)	0.4 (-0.4, 1.0)	0.1 (-0.4, 0.6)	-0.2 (-0.7, 0.3)	-0.4 (-1.0, 0.2)
Canada										
rural	147.7 (146.4, 149.1)	112.2 (110.1, 114.4)	143.3 (141.6, 145.0)	164.1 (162.6, 165.6)	164.7 (163.0, 166.5)	148.8 (147.4, 150.2)	112.6 (110.6, 114.6)	144.3 (142.8, 145.8)	165.4 (164.0, 166.8)	165.9 (164.4, 167.5)
urban	146.8 (145.7, 148.0)	111.9 (110.0, 113.8)	142.5 (141.2, 144.0)	162.9 (161.6, 164.2)	163.3 (161.7, 164.9)	147.9 (146.9, 149.0)	112.3 (110.7, 113.8)	143.6 (142.5, 144.8)	164.3 (163.2, 165.4)	164.6 (163.5, 165.7)
urban-rural difference	-0.9 (-2.1, 0.3)	-0.4 (-1.9, 1.2)	-0.8 (-2.0, 0.5)	-1.2 (-2.4, 0.0)	-1.4 (-2.7, -0.1)	-0.9 (-2.1, 0.4)	-0.3 (-1.9, 1.3)	-0.7 (-2.0, 0.6)	-1.1 (-2.3, 0.1)	-1.4 (-2.7, -0.1)
Ireland										
rural	145.6 (144.0, 147.2)	111.9 (110.2, 113.6)	140.9 (139.3, 142.6)	161.3 (159.3, 163.3)	162.7 (160.4, 165.0)	147.0 (146.2, 147.8)	112.8 (111.4, 114.2)	142.2 (141.3, 143.1)	162.8 (161.8, 163.8)	164.6 (163.3, 166.0)
urban	146.0 (144.5, 147.6)	112.5 (110.8, 114.3)	141.4 (139.8, 143.1)	161.6 (159.7, 163.6)	163.0 (160.7, 165.3)	147.4 (146.7, 148.2)	113.4 (112.1, 114.8)	142.7 (141.8, 143.6)	163.2 (162.2, 164.2)	164.9 (163.6, 166.2)
urban-rural difference	0.4 (-0.4, 1.3)	0.6 (-0.3, 1.5)	0.5 (-0.3, 1.3)	0.4 (-0.5, 1.3)	0.3 (-0.8, 1.4)	0.5 (-0.1, 1.0)	0.6 (-0.1, 1.3)	0.5 (0.0, 1.0)	0.4 (-0.3, 1.1)	0.3 (-0.6, 1.2)
New Zealand										
rural	148.1 (146.9, 149.2)	112.7 (111.1, 114.2)	143.5 (142.2, 144.8)	164.4 (163.1, 165.6)	165.5 (164.0, 167.1)	149.0 (148.3, 149.8)	113.6 (112.5, 114.7)	144.5 (143.7, 145.4)	165.3 (164.5, 166.1)	166.3 (165.3, 167.2)
urban	147.3 (146.2, 148.4)	112.4 (111.0, 113.8)	142.9 (141.7, 144.1)	163.4 (162.2, 164.6)	164.3 (162.8, 165.8)	148.3 (147.6, 149.0)	113.4 (112.3, 114.4)	143.9 (143.2, 144.7)	164.3 (163.6, 165.1)	165.0 (164.1, 165.8)
urban-rural difference	-0.7 (-1.4, 0.0)	-0.2 (-1.1, 0.6)	-0.6 (-1.3, 0.1)	-1.0 (-1.6, -0.2)	-1.3 (-2.1, -0.4)	-0.8 (-1.2, -0.3)	-0.2 (-0.9, 0.5)	-0.6 (-1.1, -0.1)	-1.0 (-1.5, -0.5)	-1.3 (-1.9, -0.6)
United Kingdom										
rural	147.0 (146.3, 147.5)	111.7 (111.0, 112.4)	142.5 (141.8, 143.1)	163.1 (162.4, 163.7)	164.2 (163.4, 164.9)	147.0 (146.5, 147.6)	111.3 (110.3, 112.3)	142.5 (141.9, 143.1)	163.4 (162.8, 163.9)	164.4 (163.8, 165.0)
urban	146.3 (145.8, 146.9)	111.5 (110.8, 112.2)	141.9 (141.3, 142.6)	162.3 (161.6, 162.9)	163.2 (162.4, 163.9)	146.6 (146.1, 147.1)	111.3 (110.4, 112.3)	142.2 (141.6, 142.7)	162.8 (162.3, 163.3)	163.6 (163.0, 164.2)
urban-rural difference	-0.6 (-1.1, -0.2)	-0.3 (-0.8, 0.2)	-0.5 (-1.0, -0.1)	-0.8 (-1.2, -0.4)	-1.0 (-1.5, -0.6)	-0.4 (-0.8, 0.0)	0.0 (-0.4, 0.4)	-0.3 (-0.7, 0.1)	-0.6 (-1.0, -0.2)	-0.8 (-1.3, -0.3)
United States of America										
rural	146.7 (145.9, 147.4)	112.5 (111.6, 113.3)	142.4 (141.6, 143.2)	162.2 (161.4, 163.0)	163.3 (162.3, 164.4)	147.0 (146.3, 147.8)	112.8 (111.4, 114.2)	143.1 (142.3, 143.9)	162.6 (161.9, 163.3)	163.1 (162.2, 164.1)
urban	146.6 (145.8, 147.3)	112.8 (111.9, 113.6)	142.4 (141.6, 143.2)	162.0 (161.1, 162.8)	162.9 (161.8, 163.9)	146.8 (146.1, 147.4)	112.9 (111.6, 114.2)	142.9 (142.1, 143.7)	162.2 (161.5, 162.8)	162.5 (161.7, 163.3)
urban-rural difference	-0.1 (-0.6, 0.5)	0.3 (-0.3, 1.0)	0.0 (-0.5, 0.6)	-0.2 (-0.9, 0.5)	-0.5 (-1.3, 0.4)	-0.3 (-0.8, 0.2)	0.1 (-0.5, 0.7)	-0.2 (-0.7, 0.3)	-0.4 (-1.1, 0.2)	-0.6 (-1.5, 0.2)
Northwestern Europe										
Austria										
rural	150.5 (148.7, 152.4)	117.5 (115.2, 119.9)	146.4 (144.4, 148.5)	165.7 (163.9, 167.5)	166.0 (163.9, 168.1)	148.3 (147.2, 149.4)	114.7 (112.9, 116.5)	144.0 (142.8, 145.2)	163.8 (162.5, 165.0)	164.4 (163.1, 165.8)
urban	150.6 (148.9, 152.4)	118.1 (115.9, 120.5)	146.7 (144.7, 148.7)	165.4 (163.7, 167.2)	165.7 (163.6, 167.8)	147.8 (146.7, 148.8)	114.7 (113.0, 116.6)	143.7 (142.5, 144.8)	162.9 (161.6, 164.2)	163.4 (162.3, 164.7)
urban-rural difference	0.1 (-0.8, 1.1)	0.6 (-0.4, 1.7)	0.3 (-0.7, 1.2)	-0.3 (-1.3, 0.8)	-0.3 (-1.5, 0.9)	-0.5 (-1.2, 0.2)	0.0 (-0.9, 0.9)	-0.4 (-1.1, 0.4)	-0.9 (-1.8, 0.0)	-0.9 (-1.9, -0.1)
Belgium										
rural	149.5 (147.8, 151.3)	117.5 (115.2, 119.9)	146.0 (144.1, 147.9)	164.0 (162.1, 165.9)	163.9 (161.7, 166.1)	147.7 (145.8, 149.6)	112.9 (110.0, 115.8)	143.4 (141.2, 145.6)	163.4 (161.5, 165.3)	164.8 (162.9, 166.6)
urban	148.8 (146.8, 150.8)	116.3 (113.9, 118.7)	145.1 (143.0, 147.2)	163.5 (161.3, 165.8)	163.6 (161.1, 166.1)	146.3 (144.7, 147.8)	111.0 (108.6, 113.4)	141.8 (140.0, 143.6)	162.2 (160.6, 163.8)	163.8 (162.3, 165.4)
urban-rural difference	-0.8 (-2.1, 0.5)	-1.2 (-2.8, 0.3)	-0.9 (-2.3, 0.4)	-0.5 (-1.9, 0.9)	-0.3 (-1.8, 1.3)	-1.4 (-2.8, -0.2)	-1.9 (-3.5, -0.3)	-1.6 (-2.9, -0.2)	-1.2 (-2.6, 0.2)	-1.0 (-2.5, 0.5)
Denmark										

Girls	Mean height in 1990 (cm)					Mean height in 2020 (cm)				
	Age-standardised	5 years	10 years	15 years	19 years	Age-standardised	5 years	10 years	15 years	19 years
rural	148.1 (146.9, 149.2)	114.5 (113.1, 115.8)	142.6 (141.4, 143.8)	163.8 (162.4, 165.0)	167.2 (165.6, 168.9)	150.6 (149.5, 151.7)	116.9 (114.9, 118.7)	145.2 (144.1, 146.4)	166.4 (165.2, 167.6)	169.7 (168.2, 171.1)
urban	148.1 (147.5, 148.8)	114.6 (113.7, 115.5)	142.7 (142.0, 143.5)	163.8 (163.0, 164.7)	167.3 (166.1, 168.6)	150.1 (149.1, 151.1)	116.4 (114.5, 118.2)	144.7 (143.6, 145.9)	165.9 (164.8, 166.9)	169.2 (168.0, 170.3)
urban-rural difference	0.1 (-0.8, 1.0)	0.1 (-1.0, 1.1)	0.1 (-0.8, 1.0)	0.1 (-0.9, 1.1)	0.1 (-1.1, 1.3)	-0.5 (-1.2, 0.3)	-0.5 (-1.4, 0.4)	-0.5 (-1.2, 0.2)	-0.5 (-1.3, 0.3)	-0.5 (-1.5, 0.6)
Finland										
rural	147.4 (146.5, 148.3)	112.2 (110.5, 113.8)	143.0 (141.8, 144.2)	163.5 (162.4, 164.5)	164.9 (163.6, 166.2)	148.0 (146.8, 149.2)	111.4 (109.9, 113.0)	143.3 (142.0, 144.5)	164.6 (163.4, 165.9)	166.5 (165.1, 168.0)
urban	148.1 (147.2, 149.0)	112.8 (111.2, 114.5)	143.6 (142.5, 144.8)	164.1 (163.1, 165.2)	165.5 (164.3, 166.7)	148.0 (147.0, 149.0)	111.5 (110.1, 112.8)	143.3 (142.2, 144.4)	164.6 (163.5, 165.7)	166.6 (165.2, 167.9)
urban-rural difference	0.6 (0.0, 1.3)	0.7 (-0.2, 1.5)	0.6 (-0.1, 1.3)	0.6 (-0.1, 1.4)	0.6 (-0.2, 1.5)	0.0 (-0.8, 0.9)	0.0 (-0.9, 1.1)	0.0 (-0.8, 0.9)	0.0 (-0.8, 0.9)	0.0 (-1.0, 1.0)
Germany										
rural	148.6 (146.9, 150.2)	113.8 (111.7, 116.0)	144.0 (142.2, 145.9)	164.7 (162.8, 166.5)	165.8 (164.0, 167.6)	148.6 (147.1, 150.1)	113.0 (110.8, 115.2)	143.8 (142.1, 145.4)	165.0 (163.5, 166.6)	166.6 (164.8, 168.4)
urban	148.9 (147.3, 150.5)	114.4 (112.3, 116.4)	144.4 (142.6, 146.2)	164.8 (163.0, 166.6)	165.8 (164.1, 167.6)	148.3 (146.8, 149.7)	113.0 (110.8, 115.1)	143.6 (141.9, 145.1)	164.6 (163.1, 166.1)	166.0 (164.3, 167.8)
urban-rural difference	0.3 (-0.5, 1.1)	0.6 (-0.5, 1.6)	0.4 (-0.5, 1.2)	0.2 (-0.7, 1.0)	0.0 (-1.0, 1.0)	-0.3 (-1.0, 0.5)	0.0 (-1.0, 1.0)	-0.2 (-1.0, 0.6)	-0.4 (-1.2, 0.4)	-0.6 (-1.5, 0.4)
Greenland										
rural	144.7 (141.2, 148.2)	112.1 (106.9, 117.5)	140.9 (136.8, 145.0)	159.4 (156.1, 162.7)	159.6 (155.7, 163.4)	145.3 (141.5, 149.1)	112.3 (105.8, 118.6)	141.4 (136.9, 145.9)	160.3 (157.2, 163.3)	160.6 (158.0, 163.2)
urban	145.4 (141.9, 149.0)	112.6 (107.5, 118.0)	141.5 (137.5, 145.7)	160.2 (156.9, 163.4)	160.5 (156.7, 164.3)	145.4 (141.6, 149.1)	112.2 (105.7, 118.4)	141.4 (136.9, 145.8)	160.4 (157.4, 163.4)	160.8 (158.2, 163.4)
urban-rural difference	0.7 (-0.6, 2.0)	0.5 (-1.1, 2.1)	0.7 (-0.7, 2.0)	0.8 (-0.6, 2.2)	0.9 (-0.6, 2.4)	0.1 (-1.3, 1.4)	-0.1 (-1.9, 1.6)	0.0 (-1.4, 1.4)	0.2 (-1.2, 1.5)	0.2 (-1.2, 1.7)
Iceland										
rural	149.7 (145.9, 153.5)	115.6 (110.3, 121.0)	144.9 (140.7, 149.2)	165.5 (161.4, 169.4)	167.7 (163.2, 172.2)	149.8 (145.7, 154.1)	115.2 (108.7, 121.5)	144.9 (140.2, 149.7)	165.8 (161.9, 169.8)	168.3 (164.4, 172.0)
urban	150.0 (146.4, 153.7)	115.9 (110.6, 121.4)	145.2 (141.1, 149.5)	165.7 (161.9, 169.6)	168.0 (163.7, 172.1)	149.5 (145.4, 153.6)	114.9 (108.4, 121.1)	144.5 (139.8, 149.3)	165.4 (161.6, 169.2)	167.9 (164.5, 171.4)
urban-rural difference	0.3 (-1.2, 1.8)	0.3 (-1.4, 1.9)	0.3 (-1.2, 1.8)	0.3 (-1.3, 1.9)	0.3 (-1.6, 2.1)	-0.3 (-1.9, 1.2)	-0.3 (-2.1, 1.4)	-0.3 (-1.9, 1.2)	-0.3 (-1.9, 1.2)	-0.3 (-2.1, 1.4)
Luxembourg										
rural	147.3 (143.9, 150.8)	112.8 (107.7, 118.0)	142.4 (138.4, 146.4)	163.2 (159.6, 166.7)	166.1 (162.5, 169.7)	146.6 (142.9, 150.4)	112.9 (106.7, 119.3)	141.9 (137.4, 146.4)	162.1 (158.9, 165.1)	164.6 (162.2, 167.0)
urban	147.4 (143.9, 150.7)	112.9 (107.8, 118.1)	142.4 (138.4, 146.4)	163.1 (159.6, 166.6)	166.0 (162.4, 169.7)	146.0 (142.4, 149.6)	112.4 (106.3, 118.7)	141.3 (136.9, 145.7)	161.4 (158.4, 164.4)	163.9 (161.6, 166.2)
urban-rural difference	0.0 (-1.2, 1.2)	0.1 (-1.4, 1.6)	0.0 (-1.2, 1.3)	0.0 (-1.3, 1.2)	-0.1 (-1.4, 1.3)	-0.6 (-1.8, 0.6)	-0.5 (-2.1, 1.1)	-0.6 (-1.9, 0.7)	-0.7 (-1.9, 0.5)	-0.7 (-2.0, 0.6)
Netherlands										
rural	151.9 (150.0, 153.8)	119.1 (116.7, 121.6)	147.1 (145.1, 149.1)	167.4 (165.0, 169.7)	168.7 (166.0, 171.3)	151.4 (149.7, 153.2)	117.5 (114.3, 120.7)	146.3 (144.2, 148.3)	167.4 (165.7, 169.1)	169.6 (167.3, 171.9)
urban	151.7 (149.7, 153.5)	119.0 (116.6, 121.5)	146.9 (144.8, 148.9)	167.1 (164.8, 169.4)	168.3 (165.8, 170.8)	150.6 (148.9, 152.3)	116.9 (113.6, 120.1)	145.5 (143.4, 147.6)	166.6 (164.9, 168.2)	168.7 (166.6, 170.8)
urban-rural difference	-0.2 (-1.2, 0.7)	-0.1 (-1.3, 1.0)	-0.2 (-1.2, 0.7)	-0.3 (-1.3, 0.7)	-0.3 (-1.6, 0.9)	-0.8 (-1.7, 0.1)	-0.7 (-1.9, 0.5)	-0.8 (-1.7, 0.2)	-0.8 (-1.8, 0.1)	-0.9 (-2.1, 0.3)
Norway										
rural	149.5 (147.8, 151.2)	115.0 (112.1, 117.7)	144.8 (143.0, 146.6)	165.7 (163.8, 167.5)	166.5 (164.5, 168.4)	149.5 (147.8, 151.2)	115.4 (112.2, 118.6)	144.9 (143.0, 146.9)	165.5 (163.7, 167.2)	166.1 (164.1, 168.1)
urban	149.9 (148.2, 151.7)	115.1 (112.3, 117.8)	145.1 (143.3, 147.0)	166.3 (164.3, 168.2)	167.2 (165.1, 169.4)	149.3 (147.8, 150.8)	114.9 (111.9, 118.0)	144.6 (142.9, 146.4)	165.4 (163.7, 167.0)	166.1 (164.1, 168.3)
urban-rural difference	0.4 (-0.6, 1.5)	0.1 (-1.2, 1.4)	0.3 (-0.7, 1.4)	0.6 (-0.5, 1.7)	0.7 (-0.5, 2.0)	-0.2 (-1.3, 0.8)	-0.5 (-1.9, 0.9)	-0.3 (-1.4, 0.8)	-0.1 (-1.2, 1.0)	0.1 (-1.2, 1.3)
Sweden										
rural	148.8 (147.8, 149.8)	115.0 (113.4, 116.6)	143.9 (142.8, 145.0)	164.6 (163.4, 165.8)	166.4 (164.5, 168.3)	149.1 (147.7, 150.5)	114.9 (112.7, 117.1)	144.1 (142.6, 145.6)	165.1 (163.6, 166.7)	167.1 (165.1, 169.1)
urban	149.1 (148.6, 149.6)	115.3 (114.0, 116.5)	144.2 (143.6, 145.0)	164.9 (164.2, 165.6)	166.7 (165.2, 168.2)	148.8 (147.7, 149.9)	114.6 (112.6, 116.5)	143.8 (142.5, 145.1)	164.8 (163.5, 166.0)	166.8 (165.1, 168.5)
urban-rural difference	0.3 (-0.8, 1.4)	0.3 (-1.0, 1.6)	0.3 (-0.8, 1.4)	0.3 (-0.9, 1.5)	0.3 (-1.1, 1.7)	-0.3 (-1.4, 0.8)	-0.3 (-1.6, 1.0)	-0.3 (-1.5, 0.7)	-0.3 (-1.5, 0.8)	-0.3 (-1.7, 1.1)
Switzerland										
rural	147.1 (144.7, 149.4)	113.3 (111.5, 115.3)	142.7 (140.5, 144.9)	162.3 (159.2, 165.3)	164.6 (160.6, 168.6)	147.3 (146.1, 148.5)	113.6 (111.9, 115.4)	142.9 (141.6, 144.1)	162.5 (161.2, 163.9)	164.8 (162.8, 166.9)
urban	147.7 (145.4, 150.1)	113.4 (111.5, 115.2)	143.1 (141.0, 145.3)	163.2 (160.2, 166.2)	165.8 (161.9, 169.7)	147.3 (146.1, 148.4)	112.9 (111.3, 114.7)	142.7 (141.4, 143.9)	162.7 (161.4, 164.1)	165.4 (163.5, 167.3)
urban-rural difference	0.6 (-0.2, 1.5)	0.0 (-0.9, 0.9)	0.4 (-0.4, 1.3)	0.9 (-0.1, 1.9)	1.2 (0.0, 2.5)	-0.1 (-0.7, 0.6)	-0.6 (-1.5, 0.1)	-0.2 (-0.9, 0.4)	0.2 (-0.6, 1.0)	0.6 (-0.5, 1.7)
Southwestern Europe										
Andorra										
rural	145.4 (140.0, 150.8)	112.4 (106.2, 118.5)	141.3 (135.9, 146.7)	160.3 (154.4, 166.0)	161.7 (154.3, 169.5)	146.8 (141.3, 152.3)	113.5 (106.8, 120.5)	142.6 (137.1, 148.2)	161.8 (156.0, 167.4)	163.2 (156.4, 170.6)
urban	146.2 (140.8, 151.6)	113.1 (106.9, 119.2)	142.1 (136.8, 147.4)	161.0 (155.1, 166.9)	162.5 (155.0, 170.3)	146.5 (140.9, 151.8)	113.2 (106.4, 120.1)	142.3 (136.8, 147.8)	161.5 (155.7, 167.1)	162.9 (156.0, 170.2)
urban-rural difference	0.8 (-0.8, 2.3)	0.7 (-1.0, 2.5)	0.7 (-0.8, 2.3)	0.7 (-1.0, 2.4)	0.8 (-1.1, 2.6)	-0.3 (-1.8, 1.2)	-0.3 (-2.1, 1.4)	-0.3 (-1.8, 1.2)	-0.3 (-1.9, 1.2)	-0.3 (-2.0, 1.4)
Cyprus										
rural	144.8 (142.1, 147.4)	111.8 (108.8, 114.7)	141.0 (138.3, 143.6)	159.6 (156.4, 162.8)	160.2 (155.9, 164.9)	146.6 (145.1, 148.1)	113.9 (111.7, 116.1)	142.9 (141.2, 144.5)	161.3 (159.4, 163.1)	161.6 (159.2, 164.6)
urban	145.4 (142.8, 148.1)	112.4 (109.6, 115.2)	141.6 (139.1, 144.2)	160.2 (157.1, 163.5)	160.9 (156.6, 165.5)	146.1 (144.8, 147.5)	113.4 (111.4, 115.4)	142.4 (141.0, 143.9)	160.9 (159.1, 162.6)	161.3 (158.9, 164.2)
urban-rural difference	0.6 (-0.5, 1.8)	0.6 (-0.7, 1.9)	0.6 (-0.5, 1.8)	0.6 (-0.6, 1.9)	0.7 (-0.7, 2.1)	-0.4 (-1.4, 0.5)	-0.5 (-1.7, 0.8)	-0.4 (-1.5, 0.6)	-0.4 (-1.5, 0.6)	-0.4 (-1.6, 0.8)
France										
rural	146.4 (144.4, 148.4)	111.3 (109.2, 113.3)	141.9 (140.0, 143.8)	162.5 (160.1, 165.1)	163.3 (160.1, 166.5)	147.9 (146.6, 149.1)	112.7 (110.4, 115.1)	143.4 (141.9, 144.8)	164.0 (162.7, 165.3)	164.8 (163.5, 166.1)
urban	147.1 (145.2, 149.0)	111.9 (109.9, 113.8)	142.6 (140.8, 144.5)	163.3 (160.8, 165.7)	164.1 (161.0, 167.3)	147.5 (146.3, 148.8)	112.3 (110.0, 114.6)	143.0 (141.6, 144.5)	163.7 (162.5, 164.9)	164.6 (163.5, 165.7)

Girls	Mean height in 1990 (cm)					Mean height in 2020 (cm)				
	Age-standardised	5 years	10 years	15 years	19 years	Age-standardised	5 years	10 years	15 years	19 years
urban-rural difference	0.7 (-0.2, 1.6)	0.6 (-0.5, 1.8)	0.7 (-0.2, 1.6)	0.7 (-0.2, 1.7)	0.8 (-0.4, 2.0)	-0.3 (-1.1, 0.4)	-0.4 (-1.4, 0.6)	-0.3 (-1.1, 0.4)	-0.3 (-1.1, 0.5)	-0.2 (-1.2, 0.7)
Greece										
rural	146.5 (144.9, 148.1)	112.7 (111.0, 114.3)	142.1 (140.6, 143.6)	161.6 (159.5, 163.8)	164.1 (161.3, 166.8)	149.5 (148.5, 150.5)	115.3 (113.4, 117.3)	145.0 (143.8, 146.2)	164.8 (163.7, 165.9)	167.2 (165.8, 168.6)
urban	147.4 (145.8, 149.1)	113.6 (112.0, 115.3)	143.0 (141.5, 144.5)	162.5 (160.4, 164.7)	165.0 (162.2, 167.7)	149.3 (148.3, 150.2)	115.2 (113.3, 117.1)	144.8 (143.6, 145.9)	164.6 (163.6, 165.6)	167.0 (165.7, 168.3)
urban-rural difference	0.9 (0.3, 1.6)	0.9 (0.1, 1.8)	0.9 (0.3, 1.6)	0.9 (0.2, 1.7)	0.9 (0.0, 1.9)	-0.2 (-0.8, 0.4)	-0.2 (-1.0, 0.6)	-0.2 (-0.8, 0.4)	-0.2 (-0.8, 0.4)	-0.2 (-1.0, 0.6)
Israel										
rural	146.3 (145.0, 147.7)	112.8 (109.5, 116.1)	142.4 (140.7, 144.1)	161.5 (160.4, 162.6)	162.0 (160.6, 163.3)	146.4 (145.1, 147.7)	112.4 (109.6, 115.1)	142.4 (140.8, 144.0)	161.8 (160.6, 163.1)	162.4 (161.3, 163.5)
urban	146.7 (145.4, 148.0)	113.4 (110.1, 116.6)	142.8 (141.2, 144.4)	161.8 (160.7, 162.8)	162.2 (160.9, 163.5)	145.8 (144.6, 146.9)	111.9 (109.4, 114.4)	141.8 (140.3, 143.2)	161.1 (159.9, 162.4)	161.6 (160.6, 162.7)
urban-rural difference	0.4 (-0.4, 1.2)	0.6 (-0.7, 1.8)	0.4 (-0.5, 1.3)	0.3 (-0.4, 1.0)	0.3 (-0.5, 1.0)	-0.6 (-1.4, 0.2)	-0.4 (-1.7, 0.9)	-0.6 (-1.4, 0.3)	-0.7 (-1.3, 0.0)	-0.7 (-1.4, -0.1)
Italy										
rural	144.8 (143.6, 145.9)	113.0 (111.4, 114.7)	140.6 (139.4, 141.9)	159.1 (157.8, 160.4)	160.9 (159.4, 162.5)	146.4 (145.4, 147.4)	115.5 (113.2, 117.7)	142.6 (141.4, 143.7)	160.4 (159.2, 161.6)	161.4 (159.9, 163.0)
urban	146.1 (145.0, 147.3)	114.0 (112.4, 115.6)	141.9 (140.6, 143.1)	160.7 (159.4, 161.9)	162.7 (161.2, 164.2)	146.6 (145.6, 147.6)	115.3 (113.0, 117.6)	142.7 (141.5, 143.8)	160.8 (159.7, 161.9)	162.0 (160.6, 163.4)
urban-rural difference	1.4 (0.5, 2.2)	1.0 (0.0, 1.9)	1.2 (0.4, 2.1)	1.5 (0.6, 2.5)	1.8 (0.6, 3.1)	0.2 (-0.5, 0.9)	-0.2 (-1.0, 0.6)	0.1 (-0.5, 0.8)	0.4 (-0.4, 1.2)	0.6 (-0.4, 1.7)
Malta										
rural	144.1 (141.1, 146.9)	112.4 (109.5, 115.5)	139.9 (136.9, 142.8)	158.6 (155.0, 162.1)	159.8 (155.4, 164.5)	145.3 (143.6, 147.0)	113.5 (110.8, 116.3)	141.1 (139.3, 142.8)	159.9 (157.6, 162.3)	161.3 (158.6, 164.1)
urban	144.4 (141.6, 147.2)	112.5 (109.7, 115.3)	140.1 (137.3, 143.0)	159.0 (155.5, 162.5)	160.4 (156.0, 165.0)	144.5 (143.0, 145.9)	112.4 (109.9, 114.9)	140.2 (138.6, 141.8)	159.2 (157.1, 161.3)	160.6 (158.2, 163.2)
urban-rural difference	0.3 (-1.0, 1.6)	0.1 (-1.3, 1.5)	0.2 (-1.0, 1.5)	0.4 (-1.1, 1.9)	0.5 (-1.2, 2.2)	-0.8 (-2.1, 0.3)	-1.1 (-2.4, 0.2)	-0.9 (-2.1, 0.3)	-0.7 (-2.1, 0.6)	-0.6 (-2.2, 0.9)
Portugal										
rural	143.0 (141.5, 144.5)	111.9 (110.2, 113.7)	139.4 (137.7, 141.1)	156.8 (155.1, 158.7)	158.2 (155.9, 160.5)	145.4 (144.4, 146.4)	112.5 (110.9, 114.1)	141.2 (140.0, 142.3)	160.0 (158.8, 161.2)	162.2 (160.5, 163.9)
urban	144.0 (142.5, 145.5)	113.1 (111.4, 114.9)	140.4 (138.8, 142.0)	157.7 (155.9, 159.5)	159.0 (156.7, 161.2)	145.2 (144.2, 146.2)	112.6 (111.0, 114.1)	141.1 (140.0, 142.2)	159.8 (158.6, 161.0)	161.9 (160.2, 163.6)
urban-rural difference	1.0 (0.2, 1.8)	1.2 (0.3, 2.0)	1.0 (0.2, 1.8)	0.9 (0.0, 1.8)	0.7 (-0.3, 1.8)	-0.1 (-0.8, 0.5)	0.1 (-0.6, 0.8)	-0.1 (-0.7, 0.5)	-0.2 (-1.0, 0.5)	-0.4 (-1.3, 0.6)
Spain										
rural	146.8 (145.4, 148.1)	113.9 (112.1, 115.6)	143.2 (141.6, 144.7)	161.6 (160.0, 163.1)	161.5 (159.6, 163.5)	147.0 (146.2, 147.9)	114.3 (112.8, 115.9)	143.4 (142.4, 144.3)	161.7 (160.7, 162.7)	161.8 (160.7, 163.0)
urban	147.7 (146.4, 149.0)	114.8 (113.1, 116.4)	144.1 (142.7, 145.6)	162.5 (161.0, 164.0)	162.5 (160.6, 164.4)	146.9 (146.1, 147.7)	114.2 (112.7, 115.8)	143.3 (142.4, 144.2)	161.6 (160.7, 162.5)	161.8 (160.7, 162.8)
urban-rural difference	0.9 (0.2, 1.7)	0.9 (0.0, 1.9)	0.9 (0.2, 1.7)	0.9 (0.1, 1.7)	1.0 (0.1, 1.8)	-0.1 (-0.6, 0.4)	-0.1 (-0.8, 0.6)	-0.1 (-0.6, 0.4)	-0.1 (-0.7, 0.5)	-0.1 (-0.8, 0.6)
**Latin America and Caribbean**										
Andean Latin America										
Bolivia										
rural	135.6 (133.7, 137.6)	104.1 (99.7, 108.5)	132.1 (129.9, 134.3)	149.9 (148.1, 151.7)	150.1 (148.3, 152.1)	139.3 (136.7, 141.9)	107.5 (101.8, 113.4)	135.8 (132.7, 139.0)	153.7 (151.8, 155.6)	154.0 (152.4, 155.6)
urban	138.5 (136.6, 140.4)	107.6 (103.2, 112.1)	135.1 (133.0, 137.3)	152.4 (150.7, 154.1)	152.4 (150.5, 154.4)	142.3 (139.8, 144.9)	111.0 (105.4, 116.7)	138.9 (135.9, 142.1)	156.3 (154.5, 158.2)	156.3 (154.8, 157.9)
urban-rural difference	2.8 (1.9, 3.8)	3.5 (2.1, 4.9)	3.0 (2.0, 4.1)	2.6 (1.7, 3.4)	2.2 (1.3, 3.2)	2.9 (1.9, 3.9)	3.6 (2.0, 5.1)	3.1 (2.0, 4.2)	2.6 (1.8, 3.5)	2.3 (1.5, 3.2)
Ecuador										
rural	134.8 (132.9, 136.7)	102.7 (100.2, 105.2)	131.0 (129.0, 133.1)	149.3 (147.1, 151.4)	150.4 (147.6, 153.1)	138.2 (137.1, 139.4)	105.6 (104.0, 107.2)	134.5 (133.1, 135.7)	152.9 (151.7, 154.1)	154.0 (152.7, 155.3)
urban	137.0 (135.2, 138.8)	105.1 (102.6, 107.5)	133.3 (131.3, 135.2)	151.3 (149.2, 153.4)	152.4 (149.7, 155.0)	140.3 (139.2, 141.5)	107.9 (106.3, 109.5)	136.6 (135.4, 137.9)	154.9 (153.7, 156.0)	155.9 (154.7, 157.1)
urban-rural difference	2.2 (1.1, 3.3)	2.4 (1.2, 3.6)	2.2 (1.2, 3.3)	2.1 (1.0, 3.2)	2.0 (0.8, 3.2)	2.1 (1.4, 2.8)	2.3 (1.4, 3.2)	2.2 (1.4, 2.9)	2.0 (1.3, 2.7)	1.9 (1.1, 2.7)
Peru										
rural	134.0 (133.0, 135.0)	101.5 (100.0, 103.0)	129.9 (128.7, 131.0)	148.8 (147.7, 149.9)	149.9 (148.5, 151.3)	137.4 (136.2, 138.5)	105.5 (103.0, 107.9)	133.5 (132.0, 135.0)	151.9 (151.1, 152.7)	152.5 (151.8, 153.2)
urban	137.6 (136.6, 138.6)	106.2 (104.8, 107.6)	133.8 (132.6, 134.9)	151.9 (150.9, 153.0)	152.4 (151.0, 153.8)	141.2 (140.0, 142.3)	110.4 (107.9, 112.7)	137.6 (136.1, 139.1)	155.2 (154.4, 156.0)	155.3 (154.5, 156.0)
urban-rural difference	3.6 (2.8, 4.4)	4.7 (3.7, 5.7)	3.9 (3.1, 4.7)	3.1 (2.4, 3.9)	2.5 (1.8, 3.3)	3.8 (3.3, 4.3)	4.9 (4.1, 5.7)	4.1 (3.5, 4.7)	3.3 (2.9, 3.8)	2.7 (2.3, 3.2)
Caribbean										
Antigua and Barbuda										
rural	145.4 (141.9, 148.8)	113.0 (107.7, 118.7)	141.6 (138.1, 145.0)	160.0 (156.0, 163.9)	161.2 (155.5, 167.3)	148.3 (144.8, 151.7)	115.7 (109.1, 122.5)	144.4 (140.5, 148.4)	162.9 (159.9, 165.9)	164.1 (160.1, 168.9)
urban	147.1 (143.5, 150.6)	115.0 (109.7, 120.5)	143.4 (139.8, 147.0)	161.5 (157.5, 165.5)	162.6 (157.0, 168.6)	148.8 (145.2, 152.4)	116.5 (110.0, 123.2)	145.0 (141.0, 149.1)	163.2 (160.0, 166.4)	164.3 (160.3, 169.1)
urban-rural difference	1.7 (0.1, 3.4)	2.0 (0.3, 3.9)	1.8 (0.2, 3.5)	1.5 (-0.2, 3.3)	1.4 (-0.5, 3.4)	0.5 (-1.2, 2.2)	0.8 (-1.3, 2.8)	0.6 (-1.2, 2.3)	0.3 (-1.3, 2.0)	0.2 (-1.6, 2.0)
Bahamas										
rural	144.5 (141.2, 147.9)	112.3 (106.8, 117.7)	140.9 (137.4, 144.4)	159.1 (155.5, 162.6)	159.8 (155.2, 164.6)	147.5 (144.3, 150.8)	115.3 (108.8, 121.7)	143.9 (140.2, 147.7)	162.0 (159.4, 164.7)	162.8 (159.6, 166.2)
urban	146.3 (143.1, 149.4)	114.4 (109.0, 119.6)	142.7 (139.3, 146.0)	160.6 (157.2, 164.0)	161.3 (156.8, 166.1)	148.1 (145.0, 151.1)	116.1 (109.8, 122.5)	144.5 (140.9, 148.2)	162.4 (160.0, 164.7)	163.0 (160.3, 166.2)
urban-rural difference	1.7 (0.1, 3.4)	2.1 (0.3, 3.9)	1.8 (0.2, 3.5)	1.6 (-0.1, 3.3)	1.5 (-0.4, 3.4)	0.5 (-1.1, 2.2)	0.8 (-1.1, 2.9)	0.6 (-1.1, 2.3)	0.4 (-1.3, 2.0)	0.3 (-1.5, 2.0)
Barbados										
rural	146.8 (143.8, 149.9)	114.4 (109.3, 119.6)	143.0 (139.8, 146.3)	161.4 (157.9, 164.8)	162.5 (158.1, 166.9)	149.7 (146.6, 152.8)	117.2 (110.9, 123.7)	145.9 (142.3, 149.5)	164.3 (161.9, 166.7)	165.5 (162.9, 168.4)
urban	148.5 (145.3, 151.7)	116.4 (111.3, 121.7)	144.7 (141.4, 148.2)	162.8 (159.4, 166.3)	163.8 (159.3, 168.3)	150.1 (146.9, 153.3)	117.9 (111.6, 124.5)	146.4 (142.7, 150.1)	164.5 (162.0, 167.0)	165.6 (162.8, 168.5)

Girls	Mean height in 1990 (cm)					Mean height in 2020 (cm)				
	Age-standardised	5 years	10 years	15 years	19 years	Age-standardised	5 years	10 years	15 years	19 years
urban-rural difference	1.6 (0.1, 3.2)	2.0 (0.2, 3.8)	1.7 (0.2, 3.3)	1.5 (-0.1, 3.1)	1.3 (-0.5, 3.2)	0.4 (-1.2, 2.0)	0.8 (-1.3, 2.8)	0.5 (-1.2, 2.2)	0.2 (-1.4, 1.9)	0.1 (-1.5, 1.8)
Belize										
rural	140.6 (137.6, 143.7)	108.7 (103.6, 114.0)	137.2 (133.9, 140.5)	154.9 (151.8, 158.2)	155.1 (151.2, 159.0)	143.3 (140.2, 146.5)	112.0 (105.6, 118.6)	140.1 (136.3, 143.9)	157.3 (154.8, 159.9)	157.1 (154.9, 159.4)
urban	142.4 (139.2, 145.5)	110.8 (105.6, 116.1)	139.1 (135.7, 142.3)	156.5 (153.3, 159.6)	156.6 (152.8, 160.5)	143.8 (140.7, 147.1)	112.9 (106.2, 119.5)	140.7 (136.9, 144.6)	157.7 (155.1, 160.3)	157.5 (155.3, 159.7)
urban-rural difference	1.8 (0.1, 3.4)	2.1 (0.3, 3.9)	1.8 (0.2, 3.5)	1.5 (-0.2, 3.3)	1.5 (-0.4, 3.5)	0.5 (-1.2, 2.3)	0.8 (-1.2, 2.9)	0.6 (-1.1, 2.4)	0.3 (-1.4, 2.1)	0.3 (-1.5, 2.1)
Bermuda										
rural	144.2 (138.7, 149.5)	111.9 (105.3, 118.5)	140.5 (135.2, 145.7)	158.6 (152.8, 164.4)	159.6 (152.2, 167.1)	147.0 (141.5, 152.4)	114.8 (107.4, 122.3)	143.4 (137.8, 149.0)	161.5 (155.8, 166.9)	162.4 (155.6, 169.3)
urban	145.9 (140.4, 151.1)	114.0 (107.3, 120.5)	142.3 (137.0, 147.4)	160.2 (154.3, 165.9)	161.1 (153.8, 168.6)	147.5 (141.9, 152.8)	115.6 (108.3, 122.9)	144.0 (138.4, 149.4)	161.8 (156.2, 167.3)	162.7 (156.0, 169.6)
urban-rural difference	1.7 (0.1, 3.4)	2.1 (0.2, 3.9)	1.8 (0.2, 3.5)	1.6 (-0.2, 3.3)	1.5 (-0.4, 3.4)	0.5 (-1.2, 2.2)	0.8 (-1.2, 2.9)	0.6 (-1.2, 2.4)	0.3 (-1.4, 2.1)	0.2 (-1.5, 2.1)
Cuba										
rural	142.4 (140.0, 144.8)	110.0 (104.8, 115.1)	138.7 (135.9, 141.5)	157.0 (154.8, 159.3)	157.4 (154.6, 160.3)	145.5 (142.2, 148.7)	113.9 (107.5, 120.6)	142.1 (138.3, 145.9)	159.7 (157.2, 162.2)	159.5 (157.3, 161.8)
urban	144.0 (141.8, 146.0)	111.9 (106.9, 116.9)	140.4 (137.8, 142.9)	158.5 (156.6, 160.3)	158.8 (156.4, 161.2)	145.8 (142.7, 149.0)	114.7 (108.1, 121.3)	142.6 (138.8, 146.3)	159.9 (157.6, 162.3)	159.7 (157.7, 161.7)
urban-rural difference	1.6 (0.3, 2.9)	2.0 (0.3, 3.6)	1.7 (0.3, 3.1)	1.4 (0.1, 2.8)	1.3 (-0.1, 2.8)	0.4 (-1.0, 1.8)	0.7 (-1.1, 2.7)	0.5 (-1.0, 2.0)	0.2 (-1.1, 1.5)	0.1 (-1.1, 1.5)
Dominica										
rural	147.2 (144.1, 150.2)	114.8 (109.7, 120.0)	143.4 (140.2, 146.6)	161.7 (158.4, 165.1)	162.5 (158.4, 166.7)	150.2 (146.9, 153.4)	117.4 (110.8, 123.7)	146.3 (142.4, 150.1)	165.0 (162.4, 167.6)	166.0 (163.2, 169.0)
urban	148.9 (146.0, 151.8)	116.9 (111.8, 122.0)	145.3 (142.1, 148.4)	163.4 (160.1, 166.6)	164.0 (160.0, 168.0)	150.7 (147.6, 153.9)	118.2 (111.9, 124.7)	147.0 (143.3, 150.7)	165.4 (162.9, 167.8)	166.3 (163.8, 169.2)
urban-rural difference	1.8 (0.2, 3.4)	2.1 (0.3, 3.9)	1.9 (0.3, 3.5)	1.6 (-0.1, 3.3)	1.5 (-0.4, 3.4)	0.6 (-1.1, 2.3)	0.9 (-1.1, 2.9)	0.6 (-1.1, 2.4)	0.4 (-1.3, 2.1)	0.3 (-1.5, 2.0)
Dominican Republic										
rural	142.1 (140.0, 144.2)	110.2 (105.5, 115.3)	138.5 (135.9, 141.0)	156.4 (154.7, 158.1)	157.2 (155.6, 158.8)	145.0 (142.2, 147.9)	112.8 (106.7, 119.1)	141.3 (137.9, 144.9)	159.5 (157.6, 161.5)	160.5 (158.9, 162.1)
urban	143.6 (141.6, 145.7)	112.3 (107.5, 117.3)	140.1 (137.6, 142.7)	157.7 (156.0, 159.5)	158.3 (156.6, 159.8)	145.4 (142.6, 148.3)	113.6 (107.6, 120.0)	141.8 (138.4, 145.3)	159.7 (157.7, 161.6)	160.4 (158.8, 162.0)
urban-rural difference	1.6 (0.5, 2.6)	2.0 (0.5, 3.7)	1.7 (0.5, 2.8)	1.3 (0.4, 2.3)	1.1 (0.0, 2.1)	0.3 (-0.9, 1.6)	0.8 (-1.0, 2.7)	0.5 (-0.9, 1.9)	0.1 (-0.9, 1.2)	-0.1 (-1.2, 0.9)
Grenada										
rural	146.1 (142.4, 149.8)	113.3 (107.5, 119.1)	142.0 (137.8, 146.2)	160.8 (156.8, 164.6)	162.7 (158.4, 166.8)	148.8 (144.6, 152.9)	115.9 (108.7, 122.8)	144.7 (139.8, 149.5)	163.5 (159.8, 167.2)	165.4 (162.2, 168.5)
urban	147.8 (144.0, 151.5)	115.4 (109.5, 121.4)	143.8 (139.5, 148.1)	162.4 (158.5, 166.2)	164.1 (159.7, 168.3)	149.3 (145.1, 153.3)	116.8 (109.6, 123.6)	145.3 (140.4, 150.0)	163.9 (160.1, 167.5)	165.6 (162.4, 168.9)
urban-rural difference	1.7 (0.2, 3.4)	2.0 (0.2, 3.9)	1.8 (0.2, 3.5)	1.6 (-0.1, 3.3)	1.4 (-0.5, 3.4)	0.5 (-1.2, 2.2)	0.8 (-1.2, 2.9)	0.6 (-1.2, 2.4)	0.3 (-1.3, 2.1)	0.2 (-1.6, 2.0)
Guyana										
rural	142.1 (139.3, 144.9)	110.2 (105.2, 115.2)	138.6 (135.6, 141.5)	156.6 (153.5, 159.7)	156.7 (152.9, 160.6)	144.5 (141.6, 147.5)	112.9 (106.8, 119.3)	141.2 (137.6, 144.7)	158.8 (156.7, 161.0)	158.8 (157.2, 160.5)
urban	144.6 (141.7, 147.5)	112.7 (107.7, 117.9)	141.1 (138.0, 144.1)	159.0 (155.8, 162.1)	159.2 (155.3, 163.1)	145.7 (142.8, 148.6)	114.1 (107.9, 120.5)	142.3 (138.8, 145.9)	159.9 (157.8, 162.1)	159.9 (158.3, 161.6)
urban-rural difference	2.4 (1.2, 3.7)	2.5 (0.9, 4.2)	2.5 (1.1, 3.8)	2.4 (1.1, 3.7)	2.4 (1.0, 3.9)	1.1 (-0.2, 2.5)	1.2 (-0.7, 3.1)	1.2 (-0.3, 2.6)	1.1 (-0.1, 2.3)	1.1 (0.0, 2.3)
Haiti										
rural	141.3 (139.1, 143.5)	109.0 (104.3, 113.9)	137.3 (134.7, 139.8)	155.8 (153.8, 157.7)	157.6 (155.3, 159.8)	143.6 (141.1, 146.1)	111.1 (105.2, 117.1)	139.5 (136.5, 142.6)	158.1 (156.5, 159.8)	160.1 (158.6, 161.6)
urban	142.9 (140.7, 145.0)	111.0 (106.3, 115.9)	139.0 (136.5, 141.5)	157.1 (155.2, 159.1)	158.7 (156.5, 160.9)	144.0 (141.4, 146.5)	111.9 (106.0, 117.9)	140.0 (137.0, 143.1)	158.3 (156.7, 159.9)	160.0 (158.5, 161.5)
urban-rural difference	1.6 (0.6, 2.6)	2.0 (0.6, 3.6)	1.7 (0.6, 2.8)	1.4 (0.4, 2.3)	1.1 (0.0, 2.1)	0.4 (-0.7, 1.4)	0.8 (-0.9, 2.5)	0.5 (-0.7, 1.7)	0.2 (-0.7, 1.0)	-0.1 (-0.9, 0.7)
Jamaica										
rural	146.2 (144.1, 148.3)	113.5 (109.0, 117.8)	142.3 (140.1, 144.5)	161.0 (158.8, 163.3)	162.0 (159.3, 164.6)	149.2 (146.6, 151.7)	116.7 (111.1, 122.5)	145.4 (142.4, 148.4)	163.8 (162.0, 165.7)	164.5 (162.8, 166.3)
urban	147.2 (145.1, 149.3)	114.9 (110.4, 119.3)	143.5 (141.2, 145.7)	161.9 (159.7, 164.1)	162.6 (160.0, 165.2)	148.9 (146.4, 151.4)	116.9 (111.2, 122.7)	145.3 (142.3, 148.3)	163.4 (161.6, 165.2)	163.9 (162.2, 165.6)
urban-rural difference	1.0 (-0.1, 2.1)	1.5 (-0.1, 3.0)	1.2 (0.0, 2.3)	0.8 (-0.2, 1.9)	0.6 (-0.6, 1.8)	-0.2 (-1.5, 1.0)	0.2 (-1.6, 2.0)	-0.1 (-1.5, 1.3)	-0.4 (-1.6, 0.7)	-0.7 (-1.7, 0.4)
Puerto Rico										
rural	145.2 (141.3, 149.3)	113.7 (107.9, 119.5)	142.3 (138.1, 146.5)	159.1 (154.9, 163.3)	158.6 (153.6, 163.6)	148.3 (144.2, 152.3)	116.7 (109.7, 123.6)	145.4 (140.8, 149.9)	162.2 (158.5, 165.7)	161.7 (158.3, 165.0)
urban	147.0 (143.2, 150.9)	115.9 (110.1, 121.6)	144.2 (140.1, 148.3)	160.6 (156.5, 164.6)	160.2 (155.3, 165.0)	148.9 (145.0, 152.7)	117.6 (110.6, 124.7)	146.1 (141.6, 150.5)	162.5 (159.1, 165.9)	162.1 (159.0, 165.2)
urban-rural difference	1.8 (0.1, 3.5)	2.2 (0.3, 4.0)	1.9 (0.2, 3.6)	1.5 (-0.3, 3.3)	1.6 (-0.3, 3.5)	0.6 (-1.1, 2.3)	0.9 (-1.1, 3.0)	0.7 (-1.1, 2.4)	0.3 (-1.5, 2.1)	0.4 (-1.3, 2.2)
Saint Kitts and Nevis										
rural	145.2 (142.2, 148.2)	113.6 (108.5, 118.9)	141.9 (138.6, 145.1)	159.3 (156.1, 162.6)	159.3 (155.0, 163.5)	148.6 (145.5, 151.6)	116.6 (110.1, 123.0)	145.3 (141.6, 148.9)	162.8 (160.3, 165.4)	162.9 (160.3, 165.9)
urban	146.9 (143.7, 150.0)	115.6 (110.3, 120.8)	143.7 (140.4, 147.0)	160.8 (157.5, 164.2)	160.8 (156.5, 165.0)	149.0 (146.0, 152.2)	117.4 (111.0, 123.9)	145.9 (142.2, 149.5)	163.1 (160.4, 165.9)	163.2 (160.5, 166.3)
urban-rural difference	1.7 (0.1, 3.3)	2.0 (0.2, 3.9)	1.8 (0.2, 3.4)	1.5 (-0.3, 3.3)	1.5 (-0.4, 3.4)	0.5 (-1.2, 2.2)	0.8 (-1.2, 2.9)	0.6 (-1.2, 2.3)	0.3 (-1.5, 2.0)	0.3 (-1.5, 2.0)
Saint Lucia										
rural	145.9 (142.1, 149.6)	113.1 (107.2, 119.1)	141.8 (137.5, 146.0)	160.6 (156.7, 164.4)	162.5 (158.2, 166.8)	148.6 (144.5, 152.6)	115.8 (108.8, 123.0)	144.6 (139.8, 149.3)	163.4 (159.7, 167.0)	165.2 (162.1, 168.2)
urban	147.6 (143.7, 151.4)	115.2 (109.2, 121.3)	143.6 (139.4, 147.9)	162.2 (158.1, 166.1)	163.8 (159.5, 168.3)	149.1 (144.9, 153.2)	116.7 (109.6, 123.9)	145.2 (140.3, 150.0)	163.7 (159.9, 167.5)	165.4 (162.0, 168.6)
urban-rural difference	1.7 (0.1, 3.3)	2.1 (0.2, 3.8)	1.8 (0.2, 3.4)	1.5 (-0.2, 3.3)	1.4 (-0.6, 3.3)	0.5 (-1.2, 2.2)	0.8 (-1.3, 2.9)	0.6 (-1.2, 2.4)	0.3 (-1.4, 2.0)	0.2 (-1.7, 2.0)
Saint Vincent and the Grenadines										
rural	146.2 (142.9, 149.4)	113.8 (108.4, 119.1)	142.3 (138.9, 145.8)	160.7 (157.2, 164.3)	162.0 (157.5, 166.5)	149.0 (146.6, 151.5)	116.6 (110.7, 122.4)	145.2 (142.2, 148.2)	163.6 (161.7, 165.5)	164.8 (162.7, 167.1)

Girls	Mean height in 1990 (cm)					Mean height in 2020 (cm)				
	Age-standardised	5 years	10 years	15 years	19 years	Age-standardised	5 years	10 years	15 years	19 years
urban	147.9 (144.7, 151.2)	115.9 (110.6, 121.3)	144.2 (140.7, 147.5)	162.3 (158.8, 165.8)	163.4 (158.9, 167.9)	149.5 (147.1, 152.0)	117.5 (111.6, 123.3)	145.8 (142.9, 148.7)	163.9 (162.0, 165.8)	165.0 (163.0, 167.2)
urban-rural difference	1.7 (0.2, 3.3)	2.1 (0.3, 3.9)	1.8 (0.3, 3.4)	1.6 (-0.1, 3.3)	1.4 (-0.5, 3.4)	0.5 (-1.1, 2.2)	0.8 (-1.2, 2.9)	0.6 (-1.1, 2.4)	0.4 (-1.3, 2.1)	0.2 (-1.6, 2.0)
Suriname										
rural	143.6 (139.3, 147.8)	111.2 (105.0, 117.1)	139.7 (135.2, 144.2)	158.1 (153.6, 162.6)	159.6 (154.3, 165.1)	145.8 (141.5, 149.9)	113.6 (106.6, 120.6)	142.0 (137.1, 146.8)	160.2 (156.4, 163.9)	161.6 (157.7, 165.4)
urban	145.3 (141.3, 149.3)	113.2 (107.0, 119.1)	141.5 (137.1, 145.8)	159.6 (155.4, 163.9)	161.0 (156.0, 166.1)	146.2 (142.2, 150.4)	114.4 (107.6, 121.5)	142.5 (137.8, 147.2)	160.5 (156.9, 164.0)	161.7 (158.2, 165.2)
urban-rural difference	1.7 (0.1, 3.4)	2.0 (0.3, 3.8)	1.8 (0.2, 3.4)	1.5 (-0.1, 3.3)	1.4 (-0.5, 3.3)	0.5 (-1.2, 2.1)	0.8 (-1.2, 2.8)	0.6 (-1.2, 2.3)	0.3 (-1.4, 2.0)	0.2 (-1.6, 2.0)
Trinidad and Tobago										
rural	144.6 (142.7, 146.4)	112.3 (110.3, 114.3)	140.8 (138.9, 142.8)	159.1 (156.8, 161.5)	160.2 (157.4, 163.0)	146.9 (144.1, 149.5)	114.6 (110.2, 119.1)	143.1 (139.8, 146.4)	161.3 (158.9, 163.7)	162.4 (159.9, 164.9)
urban	146.1 (144.1, 148.1)	113.9 (111.8, 115.9)	142.3 (140.3, 144.4)	160.6 (158.1, 163.2)	161.6 (158.7, 164.7)	147.1 (144.3, 149.9)	114.9 (110.3, 119.4)	143.4 (140.0, 146.7)	161.5 (159.1, 163.9)	162.6 (160.1, 165.2)
urban-rural difference	1.5 (0.5, 2.5)	1.5 (0.5, 2.6)	1.5 (0.5, 2.5)	1.5 (0.2, 2.7)	1.5 (-0.1, 3.0)	0.2 (-0.9, 1.4)	0.3 (-1.1, 1.7)	0.3 (-0.9, 1.4)	0.2 (-1.0, 1.4)	0.2 (-1.2, 1.6)
Central Latin America										
Colombia										
rural	137.8 (136.5, 139.1)	104.4 (102.8, 106.0)	133.9 (132.5, 135.3)	152.9 (151.4, 154.3)	153.7 (151.8, 155.5)	141.6 (140.4, 142.8)	108.0 (106.0, 110.0)	137.9 (136.5, 139.2)	156.7 (155.5, 158.0)	157.2 (155.9, 158.5)
urban	140.2 (138.8, 141.4)	107.2 (105.5, 108.8)	136.4 (135.0, 137.8)	155.0 (153.5, 156.5)	155.6 (153.7, 157.5)	143.1 (141.9, 144.3)	110.0 (108.0, 111.9)	139.5 (138.2, 140.8)	158.1 (156.9, 159.3)	158.3 (157.1, 159.5)
urban-rural difference	2.3 (1.6, 3.0)	2.8 (1.9, 3.6)	2.5 (1.8, 3.2)	2.1 (1.5, 2.8)	1.9 (1.1, 2.7)	1.5 (0.9, 2.1)	2.0 (1.2, 2.7)	1.6 (1.0, 2.3)	1.3 (0.7, 1.9)	1.1 (0.4, 1.8)
Costa Rica										
rural	139.4 (136.5, 142.3)	107.0 (102.5, 111.7)	136.0 (132.9, 139.1)	153.7 (150.5, 156.8)	154.5 (150.6, 158.3)	142.4 (139.5, 145.3)	109.5 (103.8, 115.2)	138.9 (135.5, 142.4)	156.9 (154.6, 159.2)	157.7 (155.5, 160.1)
urban	142.0 (139.3, 144.7)	110.1 (105.6, 114.6)	138.7 (135.7, 141.7)	156.1 (153.2, 158.9)	156.6 (153.0, 160.2)	144.1 (141.4, 146.9)	111.7 (106.1, 117.4)	140.8 (137.5, 144.1)	158.4 (156.3, 160.4)	159.0 (157.1, 160.9)
urban-rural difference	2.6 (1.1, 4.0)	3.1 (1.4, 4.7)	2.7 (1.2, 4.2)	2.3 (0.7, 3.9)	2.1 (0.3, 3.9)	1.7 (0.2, 3.2)	2.2 (0.4, 3.9)	1.9 (0.3, 3.3)	1.5 (-0.1, 3.0)	1.3 (-0.5, 2.9)
El Salvador										
rural	135.9 (133.6, 138.2)	103.5 (99.1, 107.9)	132.2 (129.7, 134.8)	150.1 (147.8, 152.5)	151.8 (149.2, 154.4)	140.5 (137.9, 143.1)	108.0 (102.6, 113.4)	137.0 (133.9, 140.0)	154.7 (152.8, 156.7)	156.1 (154.2, 158.0)
urban	138.3 (136.0, 140.6)	106.4 (102.0, 110.7)	134.8 (132.2, 137.3)	152.3 (150.1, 154.6)	153.7 (151.2, 156.4)	142.0 (139.5, 144.6)	110.0 (104.7, 115.6)	138.7 (135.6, 141.8)	156.1 (154.2, 158.0)	157.2 (155.3, 159.0)
urban-rural difference	2.4 (1.4, 3.4)	2.8 (1.3, 4.4)	2.5 (1.4, 3.6)	2.2 (1.2, 3.1)	1.9 (0.9, 2.9)	1.6 (0.5, 2.6)	2.0 (0.4, 3.6)	1.7 (0.5, 2.9)	1.4 (0.4, 2.3)	1.1 (0.1, 2.1)
Guatemala										
rural	133.3 (131.7, 134.9)	102.8 (99.9, 105.7)	130.0 (128.0, 131.9)	146.7 (145.2, 148.3)	147.6 (145.7, 149.6)	136.4 (135.3, 137.4)	106.6 (104.8, 108.5)	133.4 (132.0, 134.7)	149.4 (148.5, 150.4)	149.9 (148.9, 150.8)
urban	136.5 (134.9, 138.1)	106.3 (103.3, 109.2)	133.3 (131.3, 135.3)	149.8 (148.3, 151.4)	150.5 (148.6, 152.5)	138.7 (137.7, 139.8)	109.3 (107.4, 111.2)	135.8 (134.4, 137.2)	151.7 (150.7, 152.6)	152.0 (151.0, 153.0)
urban-rural difference	3.2 (2.3, 4.1)	3.5 (2.1, 4.9)	3.3 (2.3, 4.3)	3.1 (2.3, 3.9)	2.9 (2.1, 3.8)	2.4 (1.5, 3.2)	2.6 (1.2, 4.0)	2.5 (1.5, 3.4)	2.3 (1.6, 2.9)	2.1 (1.5, 2.7)
Honduras										
rural	136.1 (133.8, 138.3)	104.0 (99.4, 108.6)	132.7 (130.1, 135.3)	150.2 (148.2, 152.3)	150.7 (148.4, 153.0)	139.5 (136.7, 142.1)	107.3 (101.6, 112.8)	136.2 (133.0, 139.5)	153.7 (151.7, 155.6)	154.0 (152.1, 155.9)
urban	139.1 (136.9, 141.3)	107.4 (102.9, 111.9)	135.9 (133.3, 138.4)	153.1 (151.1, 155.1)	153.5 (151.2, 155.8)	141.7 (138.9, 144.4)	109.8 (104.3, 115.3)	138.6 (135.3, 141.8)	155.7 (153.7, 157.7)	155.9 (154.1, 157.8)
urban-rural difference	3.1 (2.0, 4.1)	3.4 (1.9, 4.8)	3.2 (2.0, 4.3)	2.9 (1.9, 3.8)	2.8 (1.7, 3.8)	2.2 (1.2, 3.2)	2.6 (1.0, 4.1)	2.3 (1.2, 3.4)	2.1 (1.2, 2.9)	1.9 (1.0, 2.8)
Mexico										
rural	137.3 (136.3, 138.2)	104.8 (103.7, 106.0)	134.0 (133.0, 135.0)	151.4 (150.3, 152.4)	152.2 (151.0, 153.4)	141.4 (140.4, 142.3)	108.4 (107.0, 109.7)	138.2 (137.2, 139.3)	155.7 (154.8, 156.7)	156.3 (155.2, 157.4)
urban	140.4 (139.5, 141.3)	108.2 (107.0, 109.3)	137.2 (136.2, 138.2)	154.4 (153.4, 155.4)	155.2 (154.0, 156.4)	143.6 (142.6, 144.6)	110.8 (109.5, 112.1)	140.5 (139.5, 141.5)	157.9 (156.9, 158.9)	158.3 (157.3, 159.4)
urban-rural difference	3.1 (2.6, 3.7)	3.3 (2.6, 4.0)	3.2 (2.6, 3.8)	3.0 (2.5, 3.6)	2.9 (2.3, 3.6)	2.2 (1.7, 2.7)	2.4 (1.8, 3.1)	2.3 (1.7, 2.8)	2.1 (1.6, 2.7)	2.0 (1.4, 2.6)
Nicaragua										
rural	137.1 (135.2, 139.0)	104.3 (100.0, 108.6)	133.5 (131.3, 135.8)	151.6 (149.9, 153.2)	152.7 (150.9, 154.3)	139.9 (137.2, 142.6)	107.4 (101.8, 112.9)	136.5 (133.3, 139.7)	154.2 (152.3, 156.2)	155.0 (153.2, 156.8)
urban	139.5 (137.5, 141.4)	107.2 (102.8, 111.5)	136.0 (133.8, 138.3)	153.7 (152.1, 155.3)	154.6 (152.8, 156.3)	141.5 (138.7, 144.2)	109.5 (103.7, 115.0)	138.2 (135.0, 141.4)	155.6 (153.6, 157.6)	156.1 (154.3, 157.9)
urban-rural difference	2.4 (1.5, 3.2)	2.9 (1.6, 4.3)	2.5 (1.6, 3.5)	2.2 (1.4, 2.9)	1.9 (1.1, 2.7)	1.6 (0.6, 2.5)	2.1 (0.6, 3.5)	1.7 (0.7, 2.8)	1.4 (0.6, 2.2)	1.1 (0.2, 1.9)
Panama										
rural	137.0 (134.9, 139.2)	104.5 (102.1, 106.9)	133.5 (131.3, 135.7)	151.3 (148.9, 153.7)	152.6 (149.9, 155.3)	139.8 (138.0, 141.5)	107.3 (103.8, 110.7)	136.3 (134.2, 138.5)	154.1 (152.7, 155.4)	155.2 (153.7, 156.7)
urban	141.3 (139.2, 143.5)	109.2 (106.9, 111.7)	137.9 (135.8, 140.1)	155.4 (153.0, 157.8)	156.5 (153.7, 159.2)	143.3 (141.5, 145.0)	111.2 (107.6, 114.7)	139.9 (137.8, 142.0)	157.3 (156.0, 158.7)	158.2 (156.8, 159.7)
urban-rural difference	4.3 (3.4, 5.2)	4.7 (3.6, 5.9)	4.4 (3.5, 5.4)	4.1 (3.2, 5.0)	3.9 (2.8, 4.9)	3.5 (2.6, 4.4)	3.9 (2.7, 5.1)	3.6 (2.7, 4.6)	3.3 (2.4, 4.2)	3.0 (2.1, 4.0)
Venezuela										
rural	138.7 (135.9, 141.4)	106.1 (101.9, 110.4)	135.0 (131.9, 138.1)	153.0 (150.0, 156.0)	154.5 (151.2, 157.8)	142.4 (139.8, 145.1)	108.9 (104.0, 113.9)	138.6 (135.4, 141.7)	157.1 (154.7, 159.5)	159.0 (157.0, 161.0)
urban	141.1 (138.5, 143.6)	109.1 (105.1, 113.1)	137.6 (134.7, 140.4)	155.2 (152.3, 157.9)	156.4 (153.4, 159.5)	144.0 (141.6, 146.4)	111.0 (106.2, 115.9)	140.4 (137.4, 143.2)	158.5 (156.3, 160.7)	160.1 (158.4, 161.9)
urban-rural difference	2.5 (1.1, 3.8)	3.0 (1.4, 4.6)	2.6 (1.3, 4.0)	2.2 (0.9, 3.6)	1.9 (0.4, 3.4)	1.6 (0.3, 2.9)	2.2 (0.5, 3.8)	1.8 (0.4, 3.1)	1.4 (0.0, 2.7)	1.1 (-0.4, 2.5)
Southern Latin America										
Argentina										
rural	141.1 (138.8, 143.5)	107.5 (104.7, 110.3)	137.7 (135.4, 140.1)	155.9 (153.2, 158.6)	156.8 (153.6, 160.0)	145.3 (143.3, 147.2)	111.3 (108.1, 114.4)	141.9 (139.6, 144.2)	160.1 (158.3, 161.9)	161.0 (158.9, 162.9)
urban	143.5 (141.4, 145.5)	110.0 (107.6, 112.5)	140.1 (138.1, 142.2)	158.2 (155.7, 160.6)	158.9 (156.0, 161.7)	145.5 (144.0, 147.0)	111.8 (108.9, 114.6)	142.3 (140.4, 144.1)	160.3 (159.0, 161.6)	161.0 (159.6, 162.3)

Girls	Mean height in 1990 (cm)					Mean height in 2020 (cm)				
	Age-standardised	5 years	10 years	15 years	19 years	Age-standardised	5 years	10 years	15 years	19 years
urban-rural difference	2.3 (0.8, 3.9)	2.6 (0.9, 4.3)	2.4 (0.9, 4.0)	2.2 (0.6, 3.9)	2.1 (0.3, 3.9)	0.3 (-1.2, 1.8)	0.5 (-1.2, 2.2)	0.3 (-1.2, 1.8)	0.2 (-1.3, 1.7)	0.0 (-1.5, 1.6)
Brazil										
rural	141.5 (140.6, 142.3)	108.9 (107.9, 109.9)	137.6 (136.7, 138.5)	156.2 (155.3, 157.2)	157.2 (156.2, 158.2)	147.3 (146.6, 148.1)	115.2 (113.7, 116.6)	143.9 (143.1, 144.7)	161.8 (161.1, 162.5)	162.1 (161.4, 163.0)
urban	143.9 (143.1, 144.6)	111.3 (110.4, 112.2)	140.0 (139.2, 140.8)	158.7 (157.8, 159.5)	159.6 (158.8, 160.5)	147.5 (146.9, 148.1)	115.3 (114.0, 116.7)	144.1 (143.4, 144.8)	162.0 (161.4, 162.5)	162.3 (161.6, 163.0)
urban-rural difference	2.4 (1.8, 3.1)	2.4 (1.7, 3.1)	2.4 (1.8, 3.1)	2.4 (1.8, 3.1)	2.4 (1.7, 3.1)	0.2 (-0.3, 0.7)	0.2 (-0.5, 0.8)	0.2 (-0.4, 0.7)	0.2 (-0.3, 0.7)	0.2 (-0.4, 0.8)
Chile										
rural	141.0 (139.2, 142.9)	108.6 (105.8, 111.5)	138.1 (135.9, 140.3)	155.2 (153.3, 157.2)	155.1 (152.7, 157.4)	144.9 (143.0, 146.7)	112.2 (109.0, 115.4)	142.0 (139.7, 144.2)	159.2 (157.6, 160.9)	159.2 (157.6, 160.8)
urban	143.4 (141.7, 145.1)	111.4 (108.6, 114.2)	140.6 (138.6, 142.6)	157.4 (155.8, 159.1)	157.2 (155.0, 159.2)	145.2 (143.4, 147.0)	112.9 (109.8, 115.9)	142.4 (140.2, 144.5)	159.3 (157.8, 160.9)	159.2 (157.9, 160.6)
urban-rural difference	2.4 (1.3, 3.5)	2.8 (1.4, 4.2)	2.5 (1.3, 3.6)	2.2 (1.0, 3.3)	2.1 (0.8, 3.3)	0.3 (-0.7, 1.3)	0.7 (-0.8, 2.1)	0.4 (-0.7, 1.5)	0.1 (-0.9, 1.0)	0.0 (-1.0, 1.0)
Paraguay										
rural	141.4 (138.4, 144.3)	109.3 (104.6, 114.0)	138.1 (134.9, 141.1)	155.6 (152.3, 159.0)	156.2 (152.1, 160.5)	146.2 (144.0, 148.4)	114.9 (109.8, 119.9)	143.3 (140.8, 145.9)	160.1 (158.3, 161.8)	160.0 (158.0, 162.1)
urban	143.9 (141.0, 146.7)	111.9 (107.3, 116.5)	140.6 (137.6, 143.7)	158.0 (154.7, 161.3)	158.6 (154.6, 162.7)	146.6 (144.5, 148.8)	115.4 (110.4, 120.5)	143.8 (141.2, 146.3)	160.4 (158.6, 162.1)	160.3 (158.4, 162.3)
urban-rural difference	2.5 (1.2, 3.7)	2.6 (1.1, 4.2)	2.5 (1.2, 3.8)	2.4 (1.1, 3.7)	2.4 (0.8, 3.9)	0.4 (-0.7, 1.5)	0.5 (-1.0, 2.1)	0.4 (-0.8, 1.6)	0.3 (-0.8, 1.3)	0.3 (-0.9, 1.5)
Uruguay										
rural	141.8 (139.1, 144.5)	109.3 (106.3, 112.2)	138.1 (135.4, 140.7)	156.4 (153.2, 159.7)	157.5 (153.6, 161.4)	145.9 (143.8, 148.0)	113.4 (109.6, 117.1)	142.3 (139.9, 144.8)	160.5 (158.5, 162.4)	161.3 (158.8, 163.8)
urban	144.3 (142.0, 146.6)	111.9 (109.4, 114.3)	140.6 (138.3, 142.8)	158.8 (155.9, 161.7)	159.8 (156.4, 163.3)	146.3 (144.7, 147.8)	113.9 (110.6, 117.2)	142.7 (140.7, 144.7)	160.8 (159.5, 162.1)	161.6 (160.0, 163.3)
urban-rural difference	2.5 (0.9, 4.1)	2.6 (0.9, 4.4)	2.5 (0.9, 4.2)	2.4 (0.7, 4.2)	2.3 (0.3, 4.4)	0.4 (-1.2, 2.0)	0.5 (-1.3, 2.4)	0.4 (-1.2, 2.1)	0.3 (-1.3, 2.0)	0.3 (-1.5, 2.0)
**Oceania**										
Melanesia										
Fiji										
rural	143.9 (141.3, 146.5)	111.7 (106.5, 117.1)	139.5 (136.6, 142.4)	158.4 (155.6, 161.1)	161.2 (157.9, 164.3)	146.5 (143.7, 149.3)	113.5 (107.3, 119.8)	141.9 (138.4, 145.3)	161.4 (159.3, 163.5)	164.6 (162.5, 166.9)
urban	144.2 (141.6, 146.8)	112.9 (107.5, 118.3)	140.0 (137.2, 142.9)	158.3 (155.5, 161.1)	160.6 (157.3, 163.8)	146.7 (143.9, 149.5)	114.6 (108.2, 120.9)	142.3 (138.9, 145.7)	161.2 (159.2, 163.2)	164.0 (161.9, 166.1)
urban-rural difference	0.3 (-1.2, 1.8)	1.2 (-0.8, 3.2)	0.5 (-1.0, 2.2)	-0.1 (-1.6, 1.4)	-0.6 (-2.3, 1.1)	0.2 (-1.6, 2.1)	1.1 (-1.4, 3.5)	0.5 (-1.5, 2.4)	-0.2 (-1.8, 1.6)	-0.6 (-2.4, 1.1)
Papua New Guinea										
rural	138.6 (135.2, 142.1)	106.3 (100.3, 112.3)	134.4 (130.4, 138.3)	153.3 (149.6, 156.9)	155.2 (151.0, 159.4)	140.8 (137.0, 144.6)	108.9 (101.6, 116.1)	136.7 (132.0, 141.1)	155.3 (152.2, 158.4)	157.0 (154.1, 160.0)
urban	139.5 (135.8, 143.3)	107.8 (101.7, 114.0)	135.5 (131.3, 139.6)	153.9 (149.9, 157.9)	155.5 (151.0, 160.0)	141.6 (137.7, 145.6)	110.3 (103.1, 117.7)	137.7 (133.0, 142.3)	155.8 (152.4, 159.2)	157.2 (153.9, 160.6)
urban-rural difference	0.9 (-1.1, 3.0)	1.5 (-0.7, 3.8)	1.1 (-0.9, 3.2)	0.7 (-1.5, 2.8)	0.3 (-2.1, 2.7)	0.8 (-1.3, 3.0)	1.4 (-1.2, 4.1)	1.0 (-1.2, 3.3)	0.5 (-1.5, 2.7)	0.2 (-2.0, 2.4)
Solomon Islands										
rural	137.1 (134.3, 139.8)	103.8 (100.4, 107.3)	132.4 (129.5, 135.3)	152.1 (148.8, 155.3)	155.1 (151.4, 158.7)	138.9 (136.3, 141.5)	105.8 (101.9, 109.8)	134.3 (131.1, 137.5)	153.8 (151.2, 156.3)	156.8 (154.5, 159.1)
urban	138.5 (135.6, 141.2)	105.8 (102.3, 109.2)	134.0 (130.9, 137.0)	153.3 (149.9, 156.6)	156.0 (152.3, 159.6)	140.2 (137.3, 142.9)	107.7 (103.9, 111.5)	135.7 (132.4, 139.0)	154.9 (152.2, 157.6)	157.6 (155.0, 160.1)
urban-rural difference	1.4 (-0.3, 3.1)	1.9 (-0.1, 3.9)	1.6 (-0.2, 3.3)	1.2 (-0.6, 3.0)	0.9 (-1.1, 2.9)	1.3 (-0.5, 3.1)	1.8 (-0.5, 4.1)	1.4 (-0.5, 3.3)	1.1 (-0.7, 2.8)	0.8 (-1.1, 2.6)
Vanuatu										
rural	140.2 (136.6, 143.7)	107.8 (101.6, 113.8)	135.8 (131.6, 139.9)	154.9 (151.2, 158.7)	157.5 (153.7, 161.3)	142.6 (138.7, 146.7)	110.0 (102.9, 117.4)	138.2 (133.4, 143.1)	157.4 (154.0, 160.8)	160.1 (157.1, 163.1)
urban	141.1 (137.4, 144.8)	109.3 (103.2, 115.5)	136.9 (132.6, 141.2)	155.5 (151.7, 159.4)	157.8 (153.7, 161.9)	143.4 (139.2, 147.6)	111.4 (104.2, 119.1)	139.1 (134.2, 144.1)	157.9 (154.3, 161.5)	160.3 (156.9, 163.7)
urban-rural difference	0.9 (-1.1, 3.0)	1.5 (-0.7, 3.8)	1.1 (-1.0, 3.1)	0.6 (-1.5, 2.8)	0.3 (-2.1, 2.7)	0.8 (-1.3, 2.9)	1.4 (-1.2, 4.0)	1.0 (-1.2, 3.2)	0.5 (-1.5, 2.6)	0.2 (-2.0, 2.5)
Polynesia and Micronesia										
American Samoa										
rural	146.2 (143.6, 148.7)	113.4 (109.2, 117.4)	141.2 (137.8, 144.5)	161.3 (158.2, 164.5)	164.4 (160.8, 168.0)	148.4 (144.4, 152.4)	116.3 (110.4, 122.1)	143.7 (139.0, 148.2)	163.3 (159.1, 167.2)	166.0 (161.9, 170.1)
urban	147.1 (144.4, 149.7)	114.8 (110.0, 119.3)	142.3 (138.8, 145.8)	162.0 (159.0, 165.1)	164.8 (161.6, 168.0)	148.7 (144.7, 152.7)	117.0 (110.9, 122.9)	144.1 (139.4, 148.7)	163.3 (159.3, 167.2)	165.8 (162.0, 169.5)
urban-rural difference	0.9 (-1.1, 3.0)	1.4 (-0.9, 3.6)	1.1 (-1.0, 3.1)	0.7 (-1.4, 2.9)	0.5 (-2.0, 2.9)	0.3 (-1.9, 2.5)	0.7 (-2.1, 3.4)	0.4 (-1.9, 2.7)	0.1 (-2.0, 2.3)	-0.2 (-2.4, 2.1)
Cook Islands										
rural	146.9 (144.1, 149.8)	114.7 (109.1, 120.4)	142.3 (139.0, 145.6)	161.9 (158.8, 164.9)	163.6 (160.1, 166.9)	150.2 (147.2, 153.3)	117.7 (111.0, 124.6)	145.5 (142.0, 149.2)	165.3 (162.9, 167.8)	167.2 (164.5, 170.0)
urban	147.8 (145.1, 150.6)	116.1 (110.4, 121.8)	143.4 (140.1, 146.6)	162.6 (159.8, 165.4)	164.1 (160.9, 167.2)	150.5 (147.7, 153.3)	118.5 (111.8, 125.4)	146.0 (142.6, 149.3)	165.4 (163.4, 167.4)	167.1 (164.8, 169.4)
urban-rural difference	0.9 (-1.1, 3.0)	1.4 (-0.9, 3.7)	1.1 (-1.0, 3.1)	0.7 (-1.4, 2.9)	0.5 (-1.9, 2.9)	0.3 (-2.0, 2.5)	0.8 (-2.1, 3.5)	0.4 (-2.0, 2.7)	0.1 (-2.2, 2.2)	-0.2 (-2.4, 2.1)
French Polynesia										
rural	147.6 (144.6, 150.7)	115.8 (110.3, 121.4)	143.1 (139.9, 146.5)	162.3 (158.9, 165.8)	164.2 (159.7, 168.6)	150.6 (147.6, 153.5)	119.4 (112.8, 125.9)	146.3 (142.9, 149.6)	165.0 (162.7, 167.3)	166.6 (164.0, 169.2)
urban	148.5 (145.4, 151.6)	117.2 (111.6, 122.8)	144.2 (140.8, 147.5)	163.0 (159.5, 166.5)	164.6 (160.2, 168.9)	150.9 (148.0, 153.6)	120.2 (113.7, 126.7)	146.7 (143.4, 149.9)	165.1 (162.9, 167.2)	166.4 (164.0, 169.0)
urban-rural difference	0.9 (-1.1, 3.0)	1.4 (-0.9, 3.7)	1.0 (-1.0, 3.1)	0.7 (-1.4, 2.9)	0.4 (-2.0, 3.0)	0.3 (-2.0, 2.4)	0.7 (-2.0, 3.4)	0.4 (-2.0, 2.6)	0.0 (-2.1, 2.2)	-0.2 (-2.5, 2.1)
Kiribati										
rural	142.0 (139.1, 144.9)	110.4 (104.8, 116.0)	137.7 (134.4, 141.0)	156.7 (153.7, 159.8)	157.8 (154.4, 161.4)	145.2 (141.9, 148.6)	114.0 (106.8, 121.1)	141.0 (137.1, 145.0)	159.8 (157.1, 162.4)	160.7 (158.3, 163.2)
urban	142.9 (139.9, 146.0)	111.8 (105.9, 117.5)	138.7 (135.4, 142.2)	157.4 (154.3, 160.4)	158.3 (154.6, 161.9)	145.5 (142.1, 148.8)	114.7 (107.6, 121.8)	141.4 (137.4, 145.4)	159.8 (157.2, 162.5)	160.5 (158.1, 162.9)

Girls	Mean height in 1990 (cm)					Mean height in 2020 (cm)				
	Age-standardised	5 years	10 years	15 years	19 years	Age-standardised	5 years	10 years	15 years	19 years
urban-rural difference	0.9 (-1.1, 2.9)	1.4 (-0.9, 3.6)	1.0 (-1.0, 3.0)	0.7 (-1.4, 2.8)	0.4 (-1.9, 2.8)	0.2 (-2.0, 2.5)	0.7 (-2.1, 3.5)	0.4 (-2.0, 2.8)	0.0 (-2.1, 2.2)	-0.2 (-2.4, 2.0)
Marshall Islands										
rural	137.7 (134.7, 140.7)	106.5 (100.9, 112.3)	133.7 (130.3, 137.3)	152.1 (149.1, 155.1)	152.6 (149.2, 156.1)	140.7 (137.0, 144.3)	110.8 (103.5, 118.1)	137.1 (132.7, 141.4)	154.5 (151.5, 157.3)	154.3 (151.9, 156.8)
urban	138.5 (135.5, 141.4)	107.8 (102.1, 113.9)	134.7 (131.1, 138.1)	152.6 (149.8, 155.3)	153.0 (149.8, 156.2)	140.8 (137.3, 144.2)	111.5 (104.2, 118.7)	137.4 (133.1, 141.6)	154.3 (151.7, 156.9)	154.0 (152.0, 156.1)
urban-rural difference	0.8 (-1.2, 2.8)	1.3 (-0.9, 3.6)	0.9 (-1.1, 3.0)	0.5 (-1.6, 2.6)	0.4 (-2.0, 2.7)	0.2 (-2.0, 2.3)	0.7 (-2.1, 3.3)	0.3 (-2.0, 2.6)	-0.1 (-2.3, 2.0)	-0.3 (-2.4, 1.9)
Micronesia (Federated States of)										
rural	141.1 (138.2, 143.9)	109.1 (103.5, 115.0)	136.5 (133.1, 140.0)	155.9 (153.0, 158.7)	157.9 (155.0, 160.8)	143.8 (140.4, 147.3)	112.8 (105.8, 120.0)	139.5 (135.4, 143.8)	158.2 (155.5, 160.8)	159.7 (157.3, 162.2)
urban	141.9 (138.9, 144.9)	110.5 (104.7, 116.6)	137.5 (134.0, 141.0)	156.4 (153.4, 159.3)	158.2 (155.1, 161.1)	144.0 (140.4, 147.5)	113.5 (106.3, 120.7)	139.8 (135.7, 144.0)	158.1 (155.3, 160.9)	159.4 (156.8, 161.9)
urban-rural difference	0.8 (-0.8, 2.4)	1.4 (-0.7, 3.5)	1.0 (-0.7, 2.6)	0.6 (-1.0, 2.1)	0.3 (-1.5, 2.0)	0.2 (-1.8, 2.1)	0.7 (-1.9, 3.3)	0.3 (-1.8, 2.4)	-0.1 (-1.8, 1.7)	-0.4 (-2.1, 1.4)
Nauru										
rural	140.5 (137.5, 143.6)	108.4 (102.8, 114.2)	136.1 (132.8, 139.5)	155.5 (152.1, 158.7)	156.9 (153.0, 160.7)	142.8 (139.2, 146.3)	111.8 (104.5, 118.9)	138.6 (134.6, 142.8)	157.2 (154.2, 160.1)	158.0 (155.2, 160.9)
urban	141.3 (138.6, 144.0)	109.7 (104.1, 115.5)	137.0 (134.0, 140.1)	156.0 (153.2, 158.7)	157.1 (154.0, 160.3)	142.9 (139.7, 145.9)	112.4 (105.4, 119.5)	138.9 (135.2, 142.7)	157.0 (154.7, 159.3)	157.6 (155.7, 159.5)
urban-rural difference	0.7 (-1.3, 2.7)	1.2 (-1.0, 3.5)	0.9 (-1.2, 2.8)	0.5 (-1.6, 2.6)	0.3 (-2.1, 2.6)	0.1 (-2.1, 2.3)	0.6 (-2.2, 3.3)	0.2 (-2.1, 2.5)	-0.1 (-2.3, 2.0)	-0.4 (-2.6, 1.8)
Niue										
rural	146.9 (143.7, 150.1)	115.5 (109.9, 121.1)	142.8 (139.3, 146.1)	161.5 (158.0, 164.9)	162.5 (157.9, 167.0)	149.5 (146.9, 152.1)	118.2 (111.8, 124.6)	145.4 (142.3, 148.5)	164.0 (162.1, 166.0)	165.2 (162.8, 167.6)
urban	147.7 (144.4, 151.0)	116.8 (111.0, 122.7)	143.7 (140.3, 147.3)	162.0 (158.5, 165.5)	162.9 (158.4, 167.3)	149.7 (147.1, 152.5)	118.8 (112.3, 125.5)	145.7 (142.5, 148.9)	164.0 (161.9, 166.0)	164.9 (162.5, 167.4)
urban-rural difference	0.8 (-1.2, 2.9)	1.3 (-1.0, 3.6)	1.0 (-1.1, 3.0)	0.6 (-1.5, 2.7)	0.4 (-2.0, 2.7)	0.2 (-2.1, 2.4)	0.7 (-2.1, 3.4)	0.3 (-2.0, 2.6)	-0.1 (-2.2, 2.1)	-0.3 (-2.6, 1.9)
Palau										
rural	141.3 (137.5, 145.2)	110.0 (103.8, 116.3)	137.3 (132.8, 141.8)	155.8 (151.7, 159.7)	156.5 (152.0, 161.0)	143.8 (139.7, 148.0)	113.1 (105.7, 120.7)	139.9 (135.0, 144.9)	158.0 (154.4, 161.4)	158.4 (155.7, 161.2)
urban	142.2 (138.4, 145.9)	111.4 (104.8, 118.0)	138.3 (134.0, 142.6)	156.3 (152.6, 159.9)	156.9 (152.6, 161.1)	144.0 (139.9, 148.0)	113.8 (106.4, 121.4)	140.2 (135.3, 145.0)	157.9 (154.6, 161.0)	158.2 (155.8, 160.5)
urban-rural difference	0.8 (-1.2, 2.9)	1.3 (-1.0, 3.7)	1.0 (-1.1, 3.0)	0.6 (-1.6, 2.7)	0.4 (-2.0, 2.8)	0.2 (-2.1, 2.4)	0.7 (-2.1, 3.4)	0.3 (-2.1, 2.6)	-0.1 (-2.3, 2.1)	-0.2 (-2.5, 2.0)
Samoa										
rural	142.2 (140.6, 143.9)	110.1 (106.8, 113.3)	137.2 (134.8, 139.6)	157.1 (154.9, 159.4)	160.5 (158.0, 163.0)	145.3 (142.1, 148.6)	113.1 (107.7, 118.7)	140.2 (136.5, 144.2)	160.1 (157.1, 163.2)	163.7 (161.4, 166.0)
urban	143.3 (141.2, 145.5)	111.5 (107.8, 115.4)	138.4 (135.7, 141.2)	158.0 (155.4, 160.6)	161.1 (158.1, 164.0)	145.6 (142.2, 149.1)	113.9 (108.2, 119.8)	140.7 (136.6, 144.8)	160.3 (157.1, 163.5)	163.6 (161.0, 166.3)
urban-rural difference	1.0 (-0.9, 3.0)	1.5 (-0.8, 3.7)	1.2 (-0.8, 3.1)	0.9 (-1.1, 2.8)	0.6 (-1.5, 2.8)	0.4 (-1.8, 2.6)	0.8 (-1.9, 3.5)	0.5 (-1.8, 2.8)	0.2 (-1.9, 2.3)	-0.1 (-2.1, 2.0)
Tokelau										
rural	146.5 (143.2, 149.6)	114.6 (108.7, 120.7)	142.0 (138.3, 145.7)	161.3 (158.0, 164.5)	162.8 (159.0, 166.4)	149.3 (145.6, 153.1)	117.2 (109.8, 124.8)	144.8 (140.3, 149.3)	164.3 (161.1, 167.3)	166.0 (163.5, 168.4)
urban	147.2 (143.6, 150.8)	115.9 (109.6, 122.1)	142.9 (138.9, 146.9)	161.8 (158.1, 165.5)	163.1 (158.9, 167.3)	149.5 (145.4, 153.5)	117.8 (110.3, 125.4)	145.1 (140.4, 149.8)	164.1 (160.8, 167.6)	165.6 (162.5, 168.7)
urban-rural difference	0.8 (-1.2, 2.8)	1.2 (-1.0, 3.5)	0.9 (-1.1, 2.9)	0.5 (-1.6, 2.6)	0.3 (-2.1, 2.7)	0.1 (-2.1, 2.3)	0.6 (-2.2, 3.3)	0.2 (-2.1, 2.6)	-0.1 (-2.2, 2.1)	-0.4 (-2.5, 1.9)
Tonga										
rural	147.2 (144.7, 149.7)	115.2 (110.1, 120.6)	142.6 (139.9, 145.1)	162.1 (159.3, 165.0)	164.2 (160.7, 167.7)	149.0 (146.6, 151.4)	116.8 (110.5, 123.2)	144.2 (141.3, 147.1)	164.0 (162.4, 165.7)	166.5 (164.8, 168.2)
urban	148.0 (145.1, 151.0)	116.4 (110.9, 122.0)	143.5 (140.5, 146.5)	162.8 (159.5, 166.0)	164.6 (160.7, 168.5)	149.2 (146.3, 152.0)	117.3 (110.8, 124.0)	144.5 (141.2, 147.7)	164.0 (161.7, 166.3)	166.2 (163.9, 168.6)
urban-rural difference	0.8 (-1.2, 2.9)	1.2 (-1.1, 3.5)	0.9 (-1.1, 3.0)	0.7 (-1.4, 2.8)	0.4 (-1.9, 2.8)	0.2 (-2.1, 2.4)	0.6 (-2.2, 3.2)	0.3 (-2.1, 2.5)	0.0 (-2.1, 2.2)	-0.2 (-2.4, 2.1)
Tuvalu										
rural	144.3 (141.0, 147.7)	112.4 (106.7, 118.1)	139.8 (136.3, 143.5)	159.1 (155.3, 162.8)	160.9 (155.9, 165.8)	147.1 (143.9, 150.3)	115.5 (108.6, 122.6)	142.7 (138.9, 146.5)	161.7 (159.1, 164.3)	163.4 (160.8, 166.0)
urban	145.2 (141.9, 148.7)	113.8 (107.9, 119.7)	140.9 (137.4, 144.5)	159.8 (156.1, 163.5)	161.4 (156.5, 166.2)	147.3 (144.2, 150.5)	116.3 (109.4, 123.2)	143.1 (139.3, 146.9)	161.7 (159.4, 164.1)	163.2 (160.8, 165.6)
urban-rural difference	0.9 (-1.1, 2.9)	1.4 (-0.9, 3.7)	1.1 (-1.0, 3.1)	0.7 (-1.4, 2.8)	0.4 (-2.0, 2.8)	0.3 (-2.0, 2.4)	0.7 (-2.0, 3.5)	0.4 (-1.9, 2.7)	0.0 (-2.1, 2.1)	-0.2 (-2.5, 2.0)
**South Asia**										
Afghanistan										
rural	135.3 (132.5, 138.1)	102.3 (98.2, 106.4)	129.9 (127.2, 132.7)	150.6 (147.2, 153.9)	154.5 (150.3, 158.5)	136.3 (134.2, 138.3)	103.8 (99.4, 108.4)	130.9 (128.6, 133.2)	151.2 (149.5, 153.0)	155.1 (153.5, 156.8)
urban	137.4 (134.6, 140.3)	105.7 (101.6, 109.8)	132.4 (129.6, 135.1)	152.1 (148.8, 155.5)	155.3 (151.1, 159.5)	138.4 (136.3, 140.4)	107.2 (102.9, 111.8)	133.4 (131.1, 135.7)	152.8 (151.1, 154.5)	155.9 (154.2, 157.6)
urban-rural difference	2.2 (1.1, 3.2)	3.5 (2.0, 4.9)	2.5 (1.4, 3.6)	1.6 (0.5, 2.6)	0.8 (-0.4, 2.0)	2.1 (1.2, 3.0)	3.4 (2.1, 4.9)	2.5 (1.5, 3.5)	1.5 (0.7, 2.4)	0.8 (-0.2, 1.7)
Bangladesh										
rural	137.2 (135.6, 138.9)	108.0 (103.9, 112.3)	133.8 (131.9, 135.8)	150.6 (149.1, 151.9)	150.4 (148.8, 152.0)	139.4 (138.1, 140.8)	110.9 (106.9, 115.1)	136.3 (134.8, 137.7)	152.4 (151.3, 153.5)	152.1 (151.2, 153.1)
urban	138.8 (137.1, 140.6)	110.5 (106.3, 114.8)	135.7 (133.7, 137.8)	151.7 (150.3, 152.9)	151.3 (149.7, 152.9)	140.9 (139.4, 142.4)	113.3 (109.2, 117.5)	137.9 (136.3, 139.6)	153.4 (152.1, 154.6)	152.8 (151.9, 153.8)
urban-rural difference	1.6 (0.7, 2.6)	2.5 (1.0, 4.0)	1.8 (0.8, 2.9)	1.1 (0.3, 1.9)	0.9 (0.1, 1.7)	1.4 (0.6, 2.3)	2.3 (0.9, 3.8)	1.7 (0.7, 2.7)	0.9 (0.2, 1.7)	0.8 (0.1, 1.4)
Bhutan										
rural	137.0 (133.7, 140.2)	105.3 (100.1, 110.7)	132.6 (129.0, 136.2)	151.7 (148.3, 154.9)	152.6 (148.8, 156.6)	138.9 (136.2, 141.7)	108.1 (102.4, 114.0)	134.8 (131.5, 138.2)	153.2 (151.2, 155.3)	153.9 (152.2, 155.7)
urban	138.5 (135.2, 141.6)	107.6 (102.6, 113.1)	134.3 (130.8, 137.8)	152.8 (149.6, 156.0)	153.4 (149.5, 157.2)	140.2 (137.6, 142.9)	110.2 (104.6, 116.1)	136.3 (133.1, 139.6)	154.2 (152.3, 156.1)	154.5 (152.9, 156.2)
urban-rural difference	1.5 (0.2, 2.8)	2.3 (0.7, 4.0)	1.7 (0.4, 3.1)	1.1 (-0.2, 2.5)	0.8 (-0.7, 2.2)	1.3 (0.1, 2.5)	2.2 (0.5, 3.8)	1.6 (0.3, 2.8)	0.9 (-0.2, 2.1)	0.6 (-0.7, 1.8)
India										

Girls	Mean height in 1990 (cm)					Mean height in 2020 (cm)				
	Age-standardised	5 years	10 years	15 years	19 years	Age-standardised	5 years	10 years	15 years	19 years
rural	133.2 (132.8, 133.6)	99.8 (99.3, 100.4)	127.6 (127.1, 128.2)	149.0 (148.5, 149.6)	151.9 (151.4, 152.5)	137.6 (136.8, 138.4)	106.6 (104.9, 108.3)	132.6 (131.7, 133.5)	152.2 (151.5, 152.8)	154.5 (153.9, 155.2)
urban	134.9 (134.2, 135.6)	102.5 (101.6, 103.4)	129.6 (128.8, 130.4)	150.2 (149.5, 151.0)	152.5 (151.8, 153.3)	138.9 (138.1, 139.7)	108.9 (107.4, 110.6)	134.2 (133.4, 135.2)	153.1 (152.4, 153.8)	154.8 (154.1, 155.6)
urban-rural difference	1.6 (1.0, 2.3)	2.7 (1.9, 3.5)	1.9 (1.3, 2.6)	1.2 (0.6, 1.8)	0.6 (0.0, 1.2)	1.3 (0.9, 1.8)	2.4 (1.7, 3.0)	1.6 (1.1, 2.1)	0.9 (0.4, 1.3)	0.3 (-0.2, 0.8)
Nepal										
rural	135.5 (133.7, 137.4)	104.6 (99.9, 109.4)	131.5 (129.3, 133.8)	149.8 (148.4, 151.2)	150.4 (148.8, 152.0)	137.4 (135.2, 139.6)	107.2 (101.8, 112.8)	133.5 (130.9, 136.2)	151.3 (150.1, 152.6)	151.8 (150.8, 152.9)
urban	137.0 (135.2, 138.9)	106.8 (102.2, 111.5)	133.2 (130.9, 135.5)	150.9 (149.5, 152.4)	151.2 (149.6, 152.8)	138.7 (136.5, 140.9)	109.2 (103.7, 114.9)	135.0 (132.3, 137.7)	152.3 (151.0, 153.6)	152.5 (151.4, 153.5)
urban-rural difference	1.5 (0.6, 2.4)	2.2 (0.7, 3.7)	1.7 (0.6, 2.7)	1.2 (0.4, 1.9)	0.8 (0.1, 1.6)	1.3 (0.4, 2.1)	2.0 (0.5, 3.5)	1.5 (0.5, 2.5)	1.0 (0.3, 1.6)	0.6 (0.0, 1.2)
Pakistan										
rural	136.6 (135.2, 138.0)	103.6 (102.0, 105.2)	131.9 (130.5, 133.3)	151.7 (150.3, 153.2)	154.0 (152.3, 155.8)	135.6 (134.3, 136.9)	102.4 (100.1, 104.8)	130.6 (129.2, 132.1)	150.8 (149.6, 151.9)	153.8 (152.5, 155.0)
urban	137.7 (136.3, 139.1)	105.3 (103.7, 106.9)	133.2 (131.7, 134.6)	152.6 (151.1, 154.1)	154.5 (152.7, 156.3)	136.8 (135.5, 138.1)	104.2 (101.8, 106.6)	132.0 (130.5, 133.4)	151.7 (150.5, 152.9)	154.3 (153.1, 155.6)
urban-rural difference	1.1 (0.4, 1.8)	1.7 (0.7, 2.7)	1.3 (0.5, 2.0)	0.8 (0.1, 1.6)	0.5 (-0.4, 1.4)	1.2 (0.5, 1.8)	1.7 (0.8, 2.8)	1.3 (0.6, 2.1)	0.9 (0.3, 1.6)	0.6 (-0.2, 1.3)
Sri Lanka										
rural	136.8 (133.9, 139.6)	105.2 (100.5, 110.0)	132.2 (129.2, 135.2)	151.4 (148.4, 154.4)	152.9 (149.3, 156.4)	138.8 (136.3, 141.3)	107.2 (101.6, 112.8)	134.3 (131.2, 137.3)	153.5 (151.6, 155.2)	155.1 (153.4, 157.0)
urban	138.3 (135.3, 141.3)	107.5 (102.7, 112.3)	134.0 (130.7, 137.2)	152.6 (149.4, 155.8)	153.6 (149.8, 157.4)	140.2 (137.4, 142.9)	109.4 (103.7, 115.0)	135.9 (132.6, 139.1)	154.5 (152.3, 156.6)	155.7 (153.6, 158.0)
urban-rural difference	1.5 (0.0, 3.1)	2.3 (0.6, 4.1)	1.8 (0.3, 3.4)	1.2 (-0.4, 2.8)	0.8 (-1.1, 2.6)	1.4 (-0.1, 2.9)	2.2 (0.4, 3.9)	1.6 (0.1, 3.1)	1.0 (-0.5, 2.6)	0.6 (-1.1, 2.4)
**Sub-Saharan Africa**										
Central Africa										
Angola										
rural	138.6 (134.5, 142.7)	105.1 (99.4, 111.0)	133.7 (129.4, 138.0)	153.9 (149.6, 158.1)	156.1 (151.1, 161.3)	139.3 (135.3, 143.1)	106.7 (99.8, 113.7)	134.5 (130.1, 139.0)	154.2 (150.7, 157.5)	156.1 (152.5, 159.7)
urban	140.9 (136.9, 144.8)	108.2 (102.4, 113.9)	136.2 (132.0, 140.3)	155.9 (151.7, 160.1)	157.7 (152.7, 162.7)	141.4 (137.5, 145.2)	109.5 (102.8, 116.4)	136.8 (132.4, 141.3)	155.9 (152.6, 159.2)	157.4 (154.0, 160.9)
urban-rural difference	2.3 (0.6, 3.9)	3.0 (1.0, 5.0)	2.5 (0.8, 4.2)	2.0 (0.3, 3.7)	1.6 (-0.3, 3.5)	2.1 (0.4, 3.8)	2.8 (0.6, 5.0)	2.3 (0.5, 4.1)	1.8 (0.1, 3.4)	1.4 (-0.3, 3.0)
Central African Republic										
rural	140.9 (137.1, 144.9)	106.9 (100.8, 113.0)	135.5 (131.0, 140.1)	156.5 (152.5, 160.4)	160.3 (156.0, 164.6)	141.5 (137.0, 146.0)	108.0 (100.7, 115.6)	136.1 (130.8, 141.6)	156.7 (152.7, 160.8)	160.3 (157.1, 163.5)
urban	143.6 (139.7, 147.6)	110.1 (104.0, 116.1)	138.2 (133.7, 143.0)	158.8 (154.9, 162.9)	162.3 (158.0, 166.8)	143.9 (139.5, 148.4)	111.0 (103.7, 118.5)	138.7 (133.6, 144.0)	158.9 (154.9, 162.9)	162.2 (158.9, 165.4)
urban-rural difference	2.6 (0.9, 4.4)	3.2 (1.2, 5.1)	2.8 (1.0, 4.5)	2.4 (0.5, 4.2)	2.0 (0.0, 4.1)	2.4 (0.5, 4.2)	3.0 (0.7, 5.2)	2.6 (0.7, 4.5)	2.2 (0.3, 4.0)	1.8 (-0.1, 3.8)
Congo										
rural	140.0 (138.5, 141.6)	105.6 (103.4, 107.8)	134.8 (133.0, 136.6)	155.8 (154.2, 157.4)	158.5 (156.8, 160.2)	140.3 (137.4, 143.1)	107.2 (102.4, 112.1)	135.4 (132.1, 138.7)	155.4 (153.2, 157.7)	157.6 (155.6, 159.7)
urban	142.6 (141.2, 144.1)	108.7 (107.0, 110.5)	137.5 (135.9, 139.2)	158.1 (156.6, 159.6)	160.5 (158.9, 162.1)	142.7 (139.9, 145.5)	110.2 (105.5, 114.8)	138.0 (134.7, 141.2)	157.6 (155.4, 159.8)	159.5 (157.4, 161.5)
urban-rural difference	2.6 (1.4, 3.7)	3.1 (1.4, 4.7)	2.7 (1.4, 4.0)	2.3 (1.2, 3.5)	2.0 (0.7, 3.3)	2.4 (1.1, 3.8)	2.9 (0.9, 4.9)	2.6 (1.1, 4.1)	2.2 (1.0, 3.4)	1.9 (0.8, 3.0)
DR Congo										
rural	137.3 (135.0, 139.5)	104.2 (99.4, 109.1)	132.2 (129.6, 134.8)	152.3 (150.2, 154.5)	155.1 (152.6, 157.6)	137.6 (134.7, 140.5)	105.3 (99.1, 111.6)	132.7 (129.3, 136.2)	152.3 (150.3, 154.2)	154.8 (153.0, 156.6)
urban	141.1 (138.9, 143.2)	108.4 (103.7, 113.2)	136.1 (133.6, 138.6)	156.0 (153.9, 158.2)	158.6 (156.1, 161.0)	141.2 (138.4, 144.0)	109.3 (103.2, 115.4)	136.4 (133.0, 139.7)	155.8 (153.8, 157.7)	158.1 (156.3, 159.8)
urban-rural difference	3.8 (2.6, 4.9)	4.1 (2.4, 5.8)	3.9 (2.6, 5.1)	3.7 (2.6, 4.7)	3.5 (2.3, 4.6)	3.6 (2.4, 4.9)	3.9 (2.0, 5.9)	3.7 (2.3, 5.1)	3.5 (2.4, 4.6)	3.3 (2.3, 4.3)
Equatorial Guinea										
rural	139.5 (133.9, 145.0)	105.8 (98.9, 112.8)	134.3 (128.8, 139.8)	154.8 (148.9, 160.6)	157.8 (149.9, 165.6)	140.1 (134.4, 145.9)	107.3 (99.6, 115.0)	135.2 (129.2, 141.1)	155.1 (149.3, 160.8)	157.9 (150.6, 165.3)
urban	142.1 (136.6, 147.7)	109.1 (102.4, 115.8)	137.1 (131.7, 142.5)	157.2 (151.2, 163.0)	159.8 (152.0, 167.7)	142.6 (136.8, 148.3)	110.3 (102.4, 118.0)	137.8 (131.8, 143.6)	157.3 (151.6, 162.9)	159.7 (152.6, 167.2)
urban-rural difference	2.6 (0.9, 4.4)	3.2 (1.2, 5.2)	2.8 (1.0, 4.5)	2.4 (0.5, 4.2)	2.1 (0.0, 4.2)	2.4 (0.6, 4.2)	3.0 (0.8, 5.3)	2.6 (0.7, 4.5)	2.2 (0.3, 4.0)	1.9 (-0.1, 3.8)
Gabon										
rural	139.6 (137.0, 142.2)	106.2 (100.7, 111.6)	134.6 (131.5, 137.7)	154.9 (152.4, 157.2)	157.4 (154.8, 159.9)	140.3 (137.2, 143.5)	107.3 (100.6, 114.1)	135.3 (131.5, 139.1)	155.4 (153.0, 157.7)	157.9 (155.6, 160.1)
urban	142.5 (139.9, 145.0)	109.5 (104.2, 114.8)	137.6 (134.5, 140.6)	157.6 (155.2, 159.8)	159.8 (157.3, 162.3)	143.0 (139.8, 146.1)	110.4 (103.9, 117.1)	138.1 (134.2, 141.8)	157.8 (155.6, 160.1)	160.1 (157.9, 162.3)
urban-rural difference	2.9 (1.6, 4.2)	3.4 (1.6, 5.1)	3.0 (1.7, 4.4)	2.7 (1.4, 3.9)	2.4 (1.0, 3.8)	2.7 (1.3, 4.1)	3.2 (1.1, 5.2)	2.8 (1.3, 4.4)	2.5 (1.2, 3.7)	2.2 (1.0, 3.4)
East Africa										
Burundi										
rural	138.3 (135.4, 141.1)	104.8 (100.0, 109.6)	133.9 (130.9, 136.9)	153.2 (150.1, 156.2)	155.8 (152.0, 159.6)	138.8 (136.3, 141.3)	107.0 (101.5, 112.9)	134.7 (131.8, 137.8)	152.9 (151.1, 154.6)	155.0 (153.3, 156.7)
urban	140.7 (137.9, 143.6)	107.5 (102.8, 112.3)	136.4 (133.3, 139.4)	155.4 (152.3, 158.5)	157.8 (154.0, 161.6)	141.1 (138.6, 143.6)	109.6 (104.2, 115.6)	137.1 (134.1, 140.2)	154.9 (153.2, 156.7)	156.9 (155.2, 158.6)
urban-rural difference	2.4 (1.3, 3.5)	2.7 (1.2, 4.3)	2.5 (1.3, 3.7)	2.2 (1.2, 3.2)	2.0 (0.9, 3.2)	2.3 (1.3, 3.3)	2.6 (1.1, 4.2)	2.4 (1.2, 3.5)	2.1 (1.2, 3.0)	1.9 (0.9, 2.9)
Comoros										
rural	138.8 (136.4, 141.1)	106.0 (101.1, 110.9)	134.7 (131.7, 137.6)	153.3 (151.2, 155.4)	155.0 (153.1, 156.9)	140.5 (137.6, 143.3)	108.2 (102.3, 114.0)	136.4 (132.8, 139.8)	154.8 (152.5, 156.9)	156.7 (154.7, 158.8)
urban	140.0 (137.6, 142.5)	107.9 (103.0, 112.8)	136.1 (133.1, 139.1)	154.3 (152.1, 156.4)	155.6 (153.7, 157.6)	141.6 (138.7, 144.4)	110.0 (104.0, 115.9)	137.7 (134.2, 141.1)	155.6 (153.4, 157.7)	157.2 (155.2, 159.3)
urban-rural difference	1.3 (0.2, 2.3)	1.9 (0.5, 3.5)	1.4 (0.3, 2.6)	1.0 (0.0, 2.0)	0.6 (-0.5, 1.8)	1.1 (0.0, 2.2)	1.8 (0.2, 3.4)	1.3 (0.1, 2.5)	0.8 (-0.2, 1.8)	0.5 (-0.6, 1.6)
Djibouti										

Girls	Mean height in 1990 (cm)					Mean height in 2020 (cm)				
	Age-standardised	5 years	10 years	15 years	19 years	Age-standardised	5 years	10 years	15 years	19 years
rural	140.2 (135.0, 145.6)	106.7 (100.4, 113.1)	135.8 (130.7, 141.0)	155.1 (149.4, 160.8)	157.6 (150.0, 165.3)	141.3 (135.9, 146.6)	109.0 (101.9, 116.1)	137.1 (131.8, 142.4)	155.6 (150.0, 160.9)	157.8 (150.9, 164.9)
urban	141.7 (136.6, 147.1)	108.8 (102.5, 115.2)	137.5 (132.4, 142.6)	156.3 (150.7, 162.2)	158.5 (151.1, 166.2)	142.7 (137.3, 147.9)	111.0 (103.9, 118.0)	138.7 (133.4, 144.0)	156.7 (151.2, 162.0)	158.6 (151.9, 165.6)
urban-rural difference	1.5 (0.1, 3.0)	2.2 (0.5, 3.9)	1.7 (0.2, 3.2)	1.2 (-0.3, 2.8)	0.9 (-0.8, 2.7)	1.4 (0.0, 2.9)	2.0 (0.3, 3.8)	1.6 (0.1, 3.1)	1.1 (-0.4, 2.6)	0.8 (-0.9, 2.4)
Eritrea										
rural	139.9 (137.2, 142.7)	106.8 (101.9, 111.9)	135.8 (132.5, 139.2)	154.6 (152.1, 157.2)	156.2 (154.3, 158.2)	141.2 (137.8, 144.6)	108.9 (102.6, 115.2)	137.2 (133.1, 141.2)	155.6 (152.6, 158.4)	157.2 (154.7, 159.8)
urban	141.4 (138.7, 144.3)	109.0 (103.9, 114.1)	137.5 (134.1, 141.0)	155.9 (153.2, 158.6)	157.2 (154.9, 159.5)	142.6 (139.1, 146.2)	111.0 (104.5, 117.3)	138.8 (134.5, 143.0)	156.7 (153.7, 159.7)	158.0 (155.4, 160.7)
urban-rural difference	1.5 (0.1, 3.0)	2.2 (0.5, 3.9)	1.7 (0.3, 3.2)	1.3 (-0.2, 2.8)	0.9 (-0.8, 2.7)	1.4 (0.0, 2.9)	2.0 (0.3, 3.8)	1.6 (0.1, 3.1)	1.1 (-0.3, 2.6)	0.8 (-0.8, 2.5)
Ethiopia										
rural	138.8 (136.8, 140.7)	105.0 (100.4, 109.5)	134.2 (131.8, 136.4)	153.9 (152.1, 155.6)	156.7 (154.6, 158.7)	140.7 (138.3, 143.0)	108.2 (102.4, 113.6)	136.4 (133.4, 139.1)	155.2 (153.7, 156.7)	157.6 (156.3, 158.9)
urban	140.0 (138.0, 142.0)	106.9 (102.3, 111.6)	135.6 (133.2, 137.9)	154.8 (153.0, 156.5)	157.2 (155.2, 159.2)	141.7 (139.4, 144.0)	110.0 (104.2, 115.5)	137.6 (134.6, 140.4)	155.9 (154.4, 157.3)	157.9 (156.6, 159.2)
urban-rural difference	1.2 (0.3, 2.1)	1.9 (0.5, 3.5)	1.4 (0.4, 2.5)	0.9 (0.1, 1.7)	0.5 (-0.3, 1.3)	1.0 (0.1, 1.9)	1.7 (0.3, 3.2)	1.2 (0.2, 2.2)	0.7 (0.0, 1.4)	0.3 (-0.4, 1.0)
Kenya										
rural	142.8 (140.8, 144.6)	109.2 (104.9, 113.6)	138.3 (136.0, 140.6)	157.7 (156.0, 159.4)	160.2 (158.3, 162.1)	143.5 (141.1, 145.9)	111.9 (106.5, 117.4)	139.5 (136.6, 142.4)	157.5 (155.8, 159.2)	159.5 (157.9, 161.0)
urban	143.8 (141.9, 145.7)	111.2 (106.9, 115.6)	139.6 (137.4, 141.9)	158.4 (156.6, 160.1)	160.4 (158.5, 162.3)	144.5 (142.1, 146.8)	113.8 (108.4, 119.1)	140.7 (137.9, 143.6)	158.1 (156.4, 159.8)	159.5 (158.0, 161.1)
urban-rural difference	1.1 (0.2, 2.0)	2.0 (0.5, 3.4)	1.3 (0.3, 2.4)	0.7 (-0.1, 1.5)	0.2 (-0.7, 1.0)	1.0 (0.1, 1.9)	1.9 (0.4, 3.4)	1.2 (0.2, 2.3)	0.6 (-0.2, 1.3)	0.1 (-0.7, 0.9)
Madagascar										
rural	136.1 (134.1, 138.3)	102.9 (98.3, 107.7)	131.9 (129.4, 134.5)	150.9 (149.1, 152.8)	153.0 (151.3, 154.8)	137.1 (134.2, 140.1)	105.6 (99.6, 111.6)	133.2 (129.7, 136.8)	151.1 (148.9, 153.4)	152.8 (150.7, 155.0)
urban	137.7 (135.6, 140.0)	105.1 (100.5, 109.9)	133.7 (131.1, 136.3)	152.2 (150.3, 154.1)	154.0 (152.1, 155.8)	138.6 (135.6, 141.6)	107.7 (101.8, 113.8)	134.8 (131.3, 138.4)	152.3 (150.1, 154.5)	153.7 (151.6, 155.8)
urban-rural difference	1.6 (0.6, 2.6)	2.2 (0.7, 3.8)	1.8 (0.7, 2.9)	1.3 (0.4, 2.2)	1.0 (0.0, 1.9)	1.5 (0.4, 2.5)	2.1 (0.5, 3.7)	1.6 (0.5, 2.8)	1.2 (0.3, 2.1)	0.8 (-0.1, 1.8)
Malawi										
rural	138.5 (136.5, 140.5)	105.0 (100.4, 109.7)	134.0 (131.7, 136.4)	153.4 (151.6, 155.2)	155.9 (153.8, 157.9)	139.8 (137.4, 142.3)	107.8 (102.2, 113.4)	135.7 (132.6, 138.7)	154.0 (152.5, 155.6)	156.1 (154.8, 157.4)
urban	140.2 (138.1, 142.2)	107.2 (102.6, 111.9)	135.8 (133.4, 138.3)	154.8 (153.0, 156.6)	157.0 (155.0, 159.1)	141.3 (138.9, 143.7)	109.8 (104.3, 115.5)	137.3 (134.4, 140.3)	155.3 (153.7, 156.9)	157.1 (155.8, 158.5)
urban-rural difference	1.7 (0.8, 2.6)	2.2 (0.7, 3.7)	1.8 (0.8, 2.9)	1.5 (0.7, 2.2)	1.2 (0.3, 2.0)	1.5 (0.6, 2.4)	2.0 (0.5, 3.5)	1.7 (0.6, 2.7)	1.3 (0.5, 2.0)	1.0 (0.2, 1.7)
Mauritius										
rural	143.4 (141.2, 145.6)	111.7 (107.2, 116.3)	140.1 (137.6, 142.7)	157.5 (155.4, 159.5)	157.4 (155.3, 159.5)	145.8 (143.8, 147.8)	114.8 (109.8, 120.0)	142.7 (140.4, 145.0)	159.6 (158.0, 161.1)	159.5 (158.1, 161.0)
urban	144.9 (142.7, 147.1)	113.7 (109.3, 118.4)	141.8 (139.2, 144.5)	158.7 (156.6, 160.6)	158.4 (156.3, 160.6)	147.2 (145.1, 149.3)	116.8 (111.6, 121.9)	144.2 (141.8, 146.7)	160.6 (158.9, 162.2)	160.4 (158.9, 161.9)
urban-rural difference	1.5 (0.3, 2.7)	2.1 (0.5, 3.7)	1.7 (0.4, 3.0)	1.2 (0.0, 2.4)	1.1 (-0.2, 2.3)	1.4 (0.1, 2.6)	1.9 (0.3, 3.6)	1.5 (0.2, 2.8)	1.0 (-0.2, 2.3)	0.9 (-0.3, 2.1)
Mozambique										
rural	140.1 (138.3, 141.9)	110.2 (106.3, 114.2)	136.6 (134.6, 138.7)	153.2 (151.5, 154.9)	154.7 (152.9, 156.4)	140.4 (138.3, 142.4)	111.6 (106.8, 116.4)	137.0 (134.5, 139.5)	152.9 (151.3, 154.5)	154.3 (152.7, 156.0)
urban	142.8 (141.1, 144.5)	113.4 (109.5, 117.4)	139.4 (137.4, 141.4)	155.6 (154.0, 157.3)	156.8 (155.1, 158.5)	142.9 (140.9, 144.9)	114.7 (110.0, 119.2)	139.7 (137.3, 142.1)	155.2 (153.6, 156.8)	156.3 (154.8, 157.9)
urban-rural difference	2.7 (1.7, 3.6)	3.2 (1.8, 4.7)	2.8 (1.8, 3.9)	2.4 (1.6, 3.2)	2.1 (1.2, 3.0)	2.5 (1.6, 3.5)	3.1 (1.6, 4.6)	2.7 (1.6, 3.8)	2.3 (1.5, 3.1)	2.0 (1.1, 2.8)
Rwanda										
rural	138.5 (136.5, 140.5)	104.0 (99.3, 108.7)	133.5 (131.2, 135.8)	153.8 (152.0, 155.6)	157.5 (155.5, 159.6)	139.4 (137.3, 141.5)	107.3 (101.9, 112.7)	135.0 (132.4, 137.6)	153.6 (152.4, 154.9)	156.4 (155.2, 157.6)
urban	140.6 (138.6, 142.7)	106.6 (102.0, 111.5)	135.8 (133.4, 138.2)	155.8 (154.0, 157.6)	159.2 (157.2, 161.3)	141.5 (139.3, 143.6)	109.9 (104.5, 115.4)	137.2 (134.5, 139.9)	155.5 (154.2, 156.8)	158.0 (156.7, 159.2)
urban-rural difference	2.2 (1.3, 3.1)	2.7 (1.2, 4.1)	2.3 (1.3, 3.4)	2.0 (1.2, 2.7)	1.7 (0.9, 2.5)	2.1 (1.2, 2.9)	2.6 (1.1, 4.1)	2.2 (1.2, 3.2)	1.9 (1.1, 2.5)	1.6 (0.9, 2.3)
Seychelles										
rural	145.6 (144.3, 147.0)	110.6 (109.5, 111.7)	142.5 (141.2, 143.8)	160.7 (159.0, 162.5)	160.8 (158.3, 163.3)	147.1 (146.4, 147.9)	114.0 (112.7, 115.4)	144.3 (143.5, 145.0)	161.3 (160.5, 162.1)	161.3 (160.4, 162.5)
urban	145.2 (143.7, 146.5)	110.4 (109.2, 111.5)	142.0 (140.8, 143.3)	160.1 (158.4, 161.9)	160.2 (157.6, 162.7)	146.6 (145.9, 147.4)	113.7 (112.4, 115.1)	143.8 (143.0, 144.6)	160.7 (159.8, 161.5)	160.7 (159.7, 161.8)
urban-rural difference	-0.5 (-1.0, 0.1)	-0.3 (-0.9, 0.4)	-0.4 (-1.0, 0.1)	-0.6 (-1.2, 0.0)	-0.6 (-1.4, 0.1)	-0.5 (-0.9, -0.1)	-0.3 (-0.9, 0.3)	-0.4 (-0.9, 0.0)	-0.6 (-1.2, -0.1)	-0.7 (-1.2, -0.1)
Somalia										
rural	141.4 (137.2, 145.4)	107.5 (101.6, 113.3)	136.7 (132.3, 141.0)	156.4 (152.0, 160.6)	159.6 (154.3, 164.9)	142.4 (138.2, 146.5)	109.7 (103.0, 116.2)	138.0 (133.2, 142.5)	156.9 (153.0, 160.7)	159.9 (156.0, 163.7)
urban	143.0 (138.9, 146.9)	109.7 (103.8, 115.4)	138.5 (134.2, 142.7)	157.7 (153.4, 161.9)	160.5 (155.4, 165.7)	143.8 (139.6, 147.8)	111.7 (105.0, 118.3)	139.6 (134.8, 144.1)	158.0 (154.3, 161.7)	160.7 (157.1, 164.2)
urban-rural difference	1.6 (0.1, 3.0)	2.2 (0.5, 3.9)	1.7 (0.3, 3.2)	1.3 (-0.3, 2.8)	1.0 (-0.8, 2.7)	1.4 (0.0, 2.9)	2.1 (0.3, 3.8)	1.6 (0.1, 3.1)	1.2 (-0.3, 2.7)	0.8 (-0.9, 2.5)
South Sudan										
rural	143.7 (139.5, 148.1)	109.4 (103.3, 115.3)	138.8 (134.3, 143.4)	158.9 (154.5, 163.4)	162.8 (157.4, 168.3)	145.1 (140.9, 149.4)	111.7 (104.9, 118.6)	140.3 (135.6, 145.2)	159.9 (155.9, 163.8)	163.7 (159.7, 167.9)
urban	145.4 (141.4, 149.7)	111.7 (105.6, 117.6)	140.6 (136.2, 145.3)	160.3 (156.2, 164.8)	163.9 (158.7, 169.3)	146.7 (142.5, 150.9)	113.9 (107.0, 120.6)	142.0 (137.4, 146.9)	161.2 (157.5, 165.0)	164.7 (160.8, 168.6)
urban-rural difference	1.7 (0.2, 3.2)	2.3 (0.6, 4.0)	1.8 (0.4, 3.4)	1.4 (-0.1, 3.0)	1.1 (-0.6, 2.9)	1.5 (0.1, 3.0)	2.1 (0.3, 3.9)	1.7 (0.2, 3.2)	1.3 (-0.2, 2.8)	1.0 (-0.7, 2.6)
Sudan										
rural	141.4 (138.0, 144.7)	107.3 (101.9, 112.8)	136.5 (132.5, 140.4)	156.4 (153.1, 159.7)	160.4 (157.1, 163.7)	141.9 (138.5, 145.4)	109.0 (102.6, 115.3)	137.2 (133.0, 141.5)	156.4 (153.6, 159.4)	160.4 (158.4, 162.5)
urban	142.8 (139.4, 146.2)	109.4 (104.0, 114.7)	138.1 (134.1, 142.1)	157.5 (154.2, 160.9)	161.2 (157.9, 164.6)	143.2 (139.7, 146.8)	110.9 (104.4, 117.3)	138.7 (134.4, 143.1)	157.4 (154.5, 160.3)	161.1 (159.0, 163.1)
urban-rural difference	1.4 (0.2, 2.7)	2.1 (0.5, 3.8)	1.6 (0.3, 3.0)	1.1 (-0.1, 2.4)	0.8 (-0.6, 2.2)	1.3 (0.0, 2.6)	2.0 (0.2, 3.7)	1.5 (0.1, 2.8)	1.0 (-0.2, 2.2)	0.6 (-0.6, 1.9)

Girls	Mean height in 1990 (cm)					Mean height in 2020 (cm)				
	Age-standardised	5 years	10 years	15 years	19 years	Age-standardised	5 years	10 years	15 years	19 years
Tanzania										
rural	137.8 (136.1, 139.5)	103.0 (99.3, 106.6)	132.8 (130.7, 134.9)	153.4 (151.7, 155.1)	156.6 (155.0, 158.3)	139.0 (136.9, 141.2)	105.7 (100.9, 110.5)	134.3 (131.8, 136.9)	154.0 (152.4, 155.6)	156.9 (155.4, 158.5)
urban	138.7 (137.0, 140.5)	104.9 (101.1, 108.8)	134.0 (131.9, 136.2)	153.9 (152.3, 155.6)	156.6 (155.0, 158.2)	139.9 (137.7, 142.0)	107.4 (102.6, 112.3)	135.4 (132.8, 138.1)	154.4 (152.8, 156.0)	156.7 (155.1, 158.3)
urban-rural difference	1.0 (0.0, 1.9)	1.9 (0.5, 3.4)	1.2 (0.2, 2.3)	0.5 (-0.3, 1.4)	0.0 (-0.9, 0.9)	0.8 (-0.1, 1.8)	1.8 (0.3, 3.3)	1.1 (0.0, 2.2)	0.4 (-0.4, 1.2)	-0.1 (-0.9, 0.6)
Uganda										
rural	139.2 (137.6, 140.8)	103.4 (100.9, 105.9)	133.8 (132.0, 135.7)	155.5 (153.7, 157.4)	158.5 (156.3, 160.7)	139.8 (138.1, 141.5)	105.3 (102.4, 108.3)	134.7 (132.5, 136.8)	155.5 (154.0, 157.0)	158.2 (156.9, 159.5)
urban	140.5 (138.8, 142.2)	105.8 (103.0, 108.6)	135.4 (133.5, 137.4)	156.3 (154.5, 158.2)	158.7 (156.5, 161.0)	141.0 (139.2, 142.8)	107.6 (104.3, 111.0)	136.3 (134.0, 138.6)	156.3 (154.8, 157.8)	158.4 (157.1, 159.7)
urban-rural difference	1.3 (0.4, 2.2)	2.4 (0.9, 3.9)	1.6 (0.5, 2.7)	0.8 (0.0, 1.6)	0.2 (-0.6, 1.0)	1.3 (0.4, 2.2)	2.3 (0.9, 3.9)	1.6 (0.6, 2.6)	0.8 (0.0, 1.5)	0.2 (-0.6, 0.9)
Zambia										
rural	139.6 (137.6, 141.5)	105.6 (101.1, 110.3)	135.0 (132.6, 137.4)	154.7 (153.1, 156.3)	157.4 (155.8, 159.0)	140.2 (137.7, 142.8)	108.0 (102.5, 113.8)	136.0 (132.9, 139.2)	154.6 (152.8, 156.3)	156.8 (155.3, 158.3)
urban	141.8 (139.8, 143.7)	108.3 (103.8, 113.0)	137.3 (135.0, 139.7)	156.6 (155.1, 158.2)	159.0 (157.4, 160.6)	142.2 (139.7, 144.8)	110.5 (104.9, 116.3)	138.2 (135.1, 141.3)	156.3 (154.6, 158.1)	158.2 (156.7, 159.7)
urban-rural difference	2.2 (1.3, 3.1)	2.7 (1.3, 4.2)	2.3 (1.3, 3.4)	1.9 (1.2, 2.7)	1.6 (0.8, 2.4)	2.0 (1.1, 2.9)	2.6 (1.0, 4.1)	2.2 (1.1, 3.2)	1.7 (1.0, 2.5)	1.4 (0.6, 2.2)
Southern Africa										
Botswana										
rural	142.8 (139.6, 145.9)	108.8 (103.3, 114.2)	137.1 (133.4, 140.7)	158.6 (155.5, 161.8)	161.7 (158.1, 165.4)	143.0 (139.8, 146.3)	109.8 (103.4, 116.4)	137.5 (133.6, 141.4)	158.5 (155.9, 161.2)	161.3 (158.9, 163.8)
urban	144.5 (141.4, 147.6)	110.9 (105.3, 116.4)	138.9 (135.2, 142.4)	160.1 (157.0, 163.3)	163.0 (159.4, 166.6)	143.5 (140.3, 146.7)	110.6 (104.1, 117.1)	138.1 (134.1, 141.9)	158.8 (156.3, 161.4)	161.4 (159.1, 163.6)
urban-rural difference	1.7 (0.1, 3.4)	2.1 (0.3, 3.9)	1.8 (0.2, 3.5)	1.5 (-0.2, 3.3)	1.3 (-0.7, 3.3)	0.5 (-1.1, 2.0)	0.9 (-1.0, 2.7)	0.6 (-1.0, 2.2)	0.3 (-1.4, 1.9)	0.1 (-1.7, 1.9)
Lesotho										
rural	139.5 (136.9, 142.0)	106.2 (101.2, 111.1)	134.1 (131.3, 136.9)	155.0 (152.3, 157.5)	157.3 (154.2, 160.4)	139.9 (137.1, 142.7)	107.5 (101.4, 113.8)	134.8 (131.4, 138.3)	154.9 (153.0, 156.9)	156.8 (155.0, 158.6)
urban	140.9 (138.2, 143.4)	108.0 (103.0, 113.2)	135.7 (132.8, 138.5)	156.1 (153.5, 158.7)	158.2 (155.2, 161.4)	140.1 (137.2, 143.0)	108.2 (102.0, 114.6)	135.1 (131.6, 138.7)	154.9 (152.9, 156.9)	156.5 (154.7, 158.3)
urban-rural difference	1.4 (0.3, 2.5)	1.9 (0.3, 3.5)	1.5 (0.3, 2.7)	1.2 (0.1, 2.3)	0.9 (-0.2, 2.1)	0.2 (-0.9, 1.3)	0.6 (-1.1, 2.4)	0.3 (-1.0, 1.5)	0.0 (-1.0, 0.9)	-0.3 (-1.2, 0.6)
Namibia										
rural	141.4 (139.0, 143.9)	108.1 (103.3, 113.2)	136.0 (133.4, 138.8)	157.0 (154.5, 159.4)	159.3 (156.3, 162.2)	142.1 (139.2, 145.0)	108.7 (102.5, 115.0)	136.6 (133.2, 140.1)	157.7 (155.6, 159.7)	160.2 (158.3, 162.2)
urban	142.9 (140.5, 145.3)	110.0 (105.2, 114.9)	137.6 (134.9, 140.3)	158.3 (155.9, 160.7)	160.4 (157.4, 163.3)	142.3 (139.4, 145.2)	109.3 (103.1, 115.5)	136.9 (133.5, 140.3)	157.7 (155.6, 159.8)	160.0 (158.1, 162.0)
urban-rural difference	1.5 (0.3, 2.6)	1.8 (0.3, 3.4)	1.6 (0.3, 2.8)	1.3 (0.2, 2.4)	1.1 (-0.1, 2.3)	0.2 (-1.0, 1.3)	0.5 (-1.2, 2.3)	0.3 (-1.0, 1.5)	0.0 (-1.0, 1.0)	-0.2 (-1.2, 0.8)
South Africa										
rural	141.1 (139.9, 142.3)	109.2 (107.9, 110.6)	135.9 (134.7, 137.2)	155.9 (154.4, 157.3)	158.0 (156.2, 159.7)	140.6 (139.6, 141.6)	107.7 (106.2, 109.3)	135.2 (134.1, 136.3)	155.9 (155.0, 156.8)	158.5 (157.4, 159.5)
urban	142.8 (141.5, 144.1)	111.3 (109.8, 112.8)	137.8 (136.4, 139.1)	157.4 (155.9, 158.9)	159.3 (157.6, 161.1)	141.0 (140.1, 142.0)	108.5 (106.9, 110.0)	135.7 (134.6, 136.8)	156.2 (155.2, 157.1)	158.5 (157.5, 159.6)
urban-rural difference	1.7 (0.9, 2.6)	2.1 (1.0, 3.1)	1.8 (1.0, 2.7)	1.6 (0.8, 2.4)	1.4 (0.5, 2.3)	0.4 (-0.2, 1.0)	0.7 (0.0, 1.5)	0.5 (-0.1, 1.1)	0.3 (-0.3, 0.8)	0.1 (-0.6, 0.7)
Eswatini										
rural	140.6 (137.8, 143.5)	107.5 (102.3, 112.7)	135.3 (132.1, 138.5)	156.0 (153.1, 159.1)	158.2 (154.7, 161.7)	141.5 (138.5, 144.5)	108.6 (102.1, 114.9)	136.2 (132.6, 139.9)	156.8 (154.6, 159.0)	159.0 (157.0, 161.0)
urban	142.3 (139.4, 145.2)	109.6 (104.4, 114.8)	137.1 (133.9, 140.3)	157.6 (154.5, 160.7)	159.5 (156.0, 163.0)	141.9 (138.8, 145.1)	109.4 (102.8, 115.8)	136.8 (133.0, 140.5)	157.1 (154.7, 159.5)	159.1 (157.0, 161.2)
urban-rural difference	1.7 (0.5, 3.0)	2.1 (0.5, 3.7)	1.8 (0.5, 3.1)	1.5 (0.3, 2.8)	1.3 (0.0, 2.7)	0.5 (-0.8, 1.8)	0.8 (-1.0, 2.6)	0.6 (-0.8, 2.0)	0.3 (-0.9, 1.5)	0.1 (-1.2, 1.3)
Zimbabwe										
rural	140.7 (138.6, 142.9)	107.3 (102.3, 112.3)	135.1 (132.6, 137.7)	156.3 (154.5, 158.2)	159.3 (157.2, 161.4)	141.4 (138.7, 144.1)	108.3 (102.2, 114.3)	135.9 (132.6, 139.1)	156.8 (155.0, 158.6)	159.6 (158.0, 161.2)
urban	142.5 (140.3, 144.7)	109.4 (104.5, 114.4)	137.0 (134.4, 139.6)	157.9 (156.1, 159.8)	160.7 (158.6, 162.8)	141.9 (139.2, 144.7)	109.1 (103.2, 115.2)	136.5 (133.2, 139.8)	157.2 (155.4, 159.0)	159.7 (158.1, 161.4)
urban-rural difference	1.8 (0.8, 2.8)	2.1 (0.6, 3.7)	1.8 (0.8, 3.0)	1.6 (0.7, 2.5)	1.4 (0.4, 2.4)	0.5 (-0.5, 1.5)	0.8 (-0.8, 2.4)	0.6 (-0.5, 1.8)	0.3 (-0.5, 1.2)	0.2 (-0.6, 0.9)
West Africa										
Benin										
rural	139.4 (137.5, 141.3)	106.9 (102.1, 111.4)	133.4 (130.9, 135.8)	155.0 (153.5, 156.5)	158.1 (156.6, 159.7)	139.7 (137.4, 141.9)	107.9 (102.5, 113.4)	133.8 (131.0, 136.6)	155.0 (153.5, 156.4)	158.0 (156.7, 159.3)
urban	141.1 (139.2, 143.0)	109.3 (104.6, 113.9)	135.3 (132.9, 137.7)	156.4 (155.0, 157.9)	159.2 (157.6, 160.7)	141.0 (138.7, 143.2)	109.9 (104.3, 115.3)	135.3 (132.4, 138.1)	155.9 (154.5, 157.3)	158.6 (157.2, 159.9)
urban-rural difference	1.8 (0.9, 2.7)	2.5 (1.0, 4.0)	2.0 (0.9, 3.0)	1.4 (0.7, 2.2)	1.0 (0.2, 1.8)	1.3 (0.4, 2.2)	2.0 (0.5, 3.6)	1.5 (0.5, 2.5)	1.0 (0.2, 1.7)	0.6 (-0.2, 1.3)
Burkina Faso										
rural	140.8 (139.1, 142.6)	106.2 (103.6, 108.8)	134.4 (132.4, 136.5)	157.3 (155.5, 159.1)	161.2 (159.4, 163.1)	141.1 (138.6, 143.7)	107.3 (102.8, 111.8)	134.9 (131.8, 137.9)	157.2 (155.1, 159.3)	160.9 (158.9, 162.9)
urban	142.9 (141.0, 144.7)	109.0 (106.1, 111.9)	136.7 (134.5, 138.9)	159.0 (157.2, 160.8)	162.5 (160.6, 164.5)	142.7 (140.2, 145.4)	109.7 (105.1, 114.2)	136.7 (133.6, 139.9)	158.5 (156.4, 160.6)	161.8 (159.8, 163.8)
urban-rural difference	2.0 (1.1, 3.0)	2.8 (1.3, 4.3)	2.3 (1.2, 3.4)	1.7 (0.9, 2.6)	1.3 (0.4, 2.2)	1.6 (0.6, 2.6)	2.4 (0.8, 4.0)	1.8 (0.7, 3.0)	1.3 (0.4, 2.2)	0.9 (-0.1, 1.8)
Cabo Verde										
rural	142.6 (139.3, 146.0)	109.7 (104.2, 115.4)	136.4 (132.5, 140.5)	158.6 (155.3, 162.1)	161.6 (158.0, 165.0)	145.7 (142.3, 149.2)	113.5 (107.1, 119.9)	139.8 (135.6, 144.1)	161.3 (158.4, 164.2)	163.9 (161.9, 165.9)
urban	145.0 (141.6, 148.4)	112.6 (107.0, 118.2)	138.9 (134.9, 143.0)	160.7 (157.4, 164.1)	163.4 (160.0, 166.9)	147.6 (144.3, 151.0)	116.0 (109.6, 122.3)	141.8 (137.8, 146.1)	163.0 (160.2, 165.9)	165.3 (163.4, 167.2)
urban-rural difference	2.4 (1.0, 3.7)	2.9 (1.2, 4.6)	2.5 (1.1, 3.9)	2.1 (0.8, 3.5)	1.8 (0.4, 3.3)	1.9 (0.7, 3.1)	2.4 (0.7, 4.1)	2.1 (0.7, 3.4)	1.7 (0.5, 2.9)	1.4 (0.1, 2.7)
Cameroon										

Girls	Mean height in 1990 (cm)					Mean height in 2020 (cm)				
	Age-standardised	5 years	10 years	15 years	19 years	Age-standardised	5 years	10 years	15 years	19 years
rural	141.4 (139.4, 143.4)	108.6 (104.0, 113.3)	135.4 (132.9, 137.9)	157.1 (155.5, 158.7)	160.2 (158.5, 161.8)	141.7 (139.4, 144.0)	109.7 (104.4, 115.2)	135.8 (133.0, 138.8)	157.1 (155.7, 158.5)	159.9 (158.6, 161.2)
urban	143.1 (141.1, 145.0)	111.0 (106.3, 115.7)	137.2 (134.8, 139.8)	158.5 (156.9, 160.1)	161.1 (159.4, 162.8)	142.9 (140.6, 145.2)	111.7 (106.2, 117.2)	137.3 (134.4, 140.1)	158.0 (156.6, 159.3)	160.4 (159.0, 161.7)
urban-rural difference	1.7 (0.8, 2.6)	2.4 (1.0, 4.0)	1.9 (0.9, 3.0)	1.4 (0.6, 2.1)	0.9 (0.1, 1.7)	1.2 (0.3, 2.1)	1.9 (0.4, 3.5)	1.4 (0.4, 2.5)	0.9 (0.2, 1.6)	0.5 (-0.3, 1.1)
Chad										
rural	142.6 (140.1, 144.9)	109.4 (104.4, 114.4)	136.3 (133.3, 139.3)	158.6 (156.5, 160.5)	161.9 (159.9, 163.8)	143.0 (140.4, 145.7)	110.5 (104.7, 116.3)	136.9 (133.6, 140.1)	158.8 (156.8, 160.7)	162.0 (160.1, 164.0)
urban	144.7 (142.4, 147.1)	112.2 (107.2, 117.3)	138.6 (135.7, 141.6)	160.4 (158.4, 162.4)	163.3 (161.4, 165.2)	144.8 (142.1, 147.4)	112.9 (107.1, 118.8)	138.8 (135.6, 142.0)	160.2 (158.3, 162.1)	163.1 (161.2, 165.0)
urban-rural difference	2.1 (1.0, 3.2)	2.8 (1.2, 4.4)	2.3 (1.1, 3.5)	1.8 (0.8, 2.9)	1.5 (0.3, 2.6)	1.7 (0.7, 2.8)	2.4 (0.8, 4.0)	1.9 (0.8, 3.1)	1.5 (0.5, 2.4)	1.1 (0.1, 2.1)
Côte d'Ivoire										
rural	140.5 (138.3, 142.8)	107.6 (102.9, 112.3)	134.6 (131.8, 137.3)	156.4 (154.5, 158.5)	159.0 (156.9, 161.1)	140.6 (137.8, 143.5)	109.0 (103.3, 115.0)	135.0 (131.5, 138.5)	155.9 (153.8, 158.1)	158.0 (156.0, 160.0)
urban	142.9 (140.6, 145.1)	110.6 (105.9, 115.5)	137.1 (134.4, 139.8)	158.5 (156.5, 160.6)	160.7 (158.6, 162.8)	142.6 (139.7, 145.5)	111.6 (105.7, 117.7)	137.1 (133.6, 140.6)	157.6 (155.4, 159.7)	159.3 (157.3, 161.3)
urban-rural difference	2.3 (1.4, 3.3)	3.0 (1.5, 4.5)	2.5 (1.4, 3.6)	2.0 (1.1, 3.0)	1.7 (0.7, 2.7)	1.9 (0.9, 3.0)	2.6 (1.1, 4.2)	2.1 (1.0, 3.3)	1.7 (0.8, 2.6)	1.3 (0.3, 2.2)
Gambia										
rural	143.1 (140.3, 145.8)	110.6 (105.7, 115.5)	137.3 (134.3, 140.3)	158.8 (155.9, 161.5)	160.8 (157.0, 164.3)	144.0 (141.9, 146.2)	111.9 (106.5, 117.5)	138.3 (135.7, 141.0)	159.6 (158.3, 160.8)	161.6 (160.4, 162.9)
urban	144.6 (141.9, 147.3)	112.9 (107.9, 117.8)	139.1 (136.1, 142.0)	160.0 (157.2, 162.8)	161.6 (158.0, 165.2)	145.1 (143.0, 147.3)	113.8 (108.3, 119.3)	139.6 (136.9, 142.4)	160.3 (159.0, 161.6)	162.0 (160.8, 163.2)
urban-rural difference	1.6 (0.5, 2.7)	2.3 (0.7, 3.9)	1.8 (0.6, 3.0)	1.2 (0.2, 2.3)	0.8 (-0.2, 1.9)	1.1 (0.2, 2.0)	1.8 (0.3, 3.4)	1.3 (0.3, 2.4)	0.8 (0.0, 1.5)	0.4 (-0.4, 1.2)
Ghana										
rural	139.4 (137.9, 141.0)	107.6 (104.6, 110.6)	133.3 (131.4, 135.2)	154.8 (153.3, 156.4)	157.9 (156.1, 159.7)	140.8 (139.6, 141.9)	109.3 (107.2, 111.3)	134.7 (133.3, 136.2)	156.0 (154.9, 157.2)	159.3 (158.1, 160.4)
urban	141.1 (139.5, 142.6)	110.2 (107.2, 113.2)	135.2 (133.4, 137.2)	156.1 (154.6, 157.7)	158.7 (156.9, 160.5)	142.0 (140.9, 143.2)	111.4 (109.3, 113.5)	136.2 (134.8, 137.7)	156.9 (155.8, 158.1)	159.6 (158.5, 160.8)
urban-rural difference	1.7 (0.8, 2.5)	2.5 (1.2, 3.8)	1.9 (0.9, 2.9)	1.3 (0.5, 2.1)	0.8 (0.0, 1.6)	1.2 (0.5, 1.9)	2.1 (0.9, 3.3)	1.5 (0.7, 2.3)	0.9 (0.2, 1.5)	0.4 (-0.3, 1.0)
Guinea										
rural	139.9 (137.8, 142.0)	107.7 (102.8, 112.8)	134.4 (131.1, 137.6)	155.8 (152.5, 159.0)	157.8 (154.0, 161.7)	139.8 (137.5, 142.3)	109.5 (103.4, 115.8)	135.5 (131.9, 139.2)	156.4 (153.9, 158.9)	158.2 (155.9, 160.6)
urban	142.3 (140.1, 144.4)	110.6 (105.6, 115.7)	136.8 (133.5, 140.1)	157.8 (154.5, 161.0)	159.5 (155.7, 163.3)	141.9 (139.5, 144.3)	112.0 (105.7, 118.4)	137.6 (133.8, 141.3)	158.0 (155.5, 160.4)	159.5 (157.2, 161.8)
urban-rural difference	2.4 (1.4, 3.4)	2.9 (1.3, 4.5)	2.4 (1.2, 3.7)	2.0 (0.9, 3.1)	1.7 (0.5, 2.9)	2.0 (1.1, 3.0)	2.4 (0.9, 4.0)	2.0 (0.8, 3.2)	1.6 (0.5, 2.6)	1.3 (0.2, 2.4)
Guinea Bissau										
rural	140.1 (137.1, 143.2)	107.8 (103.3, 112.6)	134.2 (131.5, 136.7)	155.4 (153.6, 157.3)	157.8 (155.7, 159.8)	141.1 (138.0, 144.2)	107.5 (102.1, 113.2)	133.9 (131.0, 136.9)	155.4 (153.9, 156.9)	158.2 (156.7, 159.7)
urban	142.4 (139.3, 145.4)	110.8 (106.1, 115.7)	136.7 (134.0, 139.4)	157.5 (155.7, 159.4)	159.6 (157.5, 161.7)	143.0 (139.8, 146.2)	110.2 (104.7, 115.8)	136.2 (133.2, 139.1)	157.1 (155.6, 158.6)	159.6 (158.1, 161.1)
urban-rural difference	2.3 (1.1, 3.4)	3.0 (1.5, 4.6)	2.6 (1.5, 3.7)	2.1 (1.2, 3.0)	1.7 (0.8, 2.7)	1.9 (0.7, 2.9)	2.7 (1.1, 4.2)	2.2 (1.1, 3.3)	1.8 (1.0, 2.5)	1.4 (0.6, 2.2)
Liberia										
rural	137.3 (134.8, 139.9)	104.8 (99.9, 109.6)	131.2 (128.4, 134.2)	153.0 (150.4, 155.6)	156.1 (153.0, 159.4)	138.4 (136.0, 140.7)	106.6 (101.1, 112.1)	132.5 (129.5, 135.3)	153.7 (152.2, 155.2)	156.6 (155.1, 158.1)
urban	139.3 (136.8, 141.9)	107.4 (102.5, 112.3)	133.4 (130.6, 136.4)	154.7 (152.2, 157.3)	157.5 (154.4, 160.7)	140.0 (137.6, 142.3)	108.7 (103.2, 114.3)	134.2 (131.2, 137.1)	155.0 (153.5, 156.5)	157.6 (156.1, 159.1)
urban-rural difference	2.0 (1.0, 3.0)	2.6 (1.1, 4.1)	2.2 (1.0, 3.3)	1.7 (0.8, 2.6)	1.4 (0.3, 2.4)	1.6 (0.7, 2.5)	2.2 (0.7, 3.7)	1.8 (0.7, 2.8)	1.3 (0.5, 2.1)	1.0 (0.2, 1.8)
Mali										
rural	142.4 (140.4, 144.2)	109.3 (104.7, 114.0)	136.2 (133.7, 138.6)	158.2 (156.8, 159.7)	161.6 (160.2, 163.0)	143.1 (140.8, 145.5)	110.8 (105.2, 116.5)	137.1 (134.2, 140.1)	158.6 (157.1, 160.1)	161.7 (160.3, 163.1)
urban	144.5 (142.5, 146.4)	112.1 (107.4, 116.9)	138.5 (135.9, 140.9)	160.0 (158.6, 161.5)	163.0 (161.6, 164.4)	144.8 (142.5, 147.2)	113.2 (107.6, 118.9)	139.0 (136.1, 142.0)	160.0 (158.5, 161.5)	162.7 (161.3, 164.1)
urban-rural difference	2.1 (1.2, 3.0)	2.8 (1.3, 4.3)	2.3 (1.3, 3.4)	1.8 (1.0, 2.6)	1.4 (0.6, 2.2)	1.7 (0.8, 2.6)	2.4 (0.9, 3.9)	1.9 (0.8, 2.9)	1.4 (0.7, 2.1)	1.0 (0.3, 1.7)
Mauritania										
rural	141.7 (139.2, 144.3)	108.9 (103.8, 114.0)	135.7 (132.5, 138.7)	157.5 (155.2, 159.8)	160.3 (157.8, 162.8)	142.7 (140.3, 145.0)	110.7 (105.1, 116.1)	136.9 (133.9, 139.7)	158.1 (156.5, 159.8)	160.8 (159.0, 162.6)
urban	143.1 (140.5, 145.6)	111.2 (106.0, 116.3)	137.3 (134.2, 140.4)	158.5 (156.2, 160.8)	160.8 (158.4, 163.3)	143.6 (141.2, 146.0)	112.4 (107.0, 117.9)	138.0 (135.1, 141.0)	158.7 (157.0, 160.3)	160.8 (159.1, 162.5)
urban-rural difference	1.4 (0.2, 2.6)	2.2 (0.7, 3.9)	1.6 (0.3, 2.9)	1.0 (-0.2, 2.2)	0.5 (-0.8, 1.8)	0.9 (-0.1, 2.0)	1.8 (0.2, 3.4)	1.1 (0.0, 2.3)	0.5 (-0.5, 1.5)	0.0 (-1.1, 1.1)
Niger										
rural	141.0 (138.8, 143.2)	108.0 (103.3, 112.8)	134.8 (132.1, 137.7)	156.8 (154.8, 158.8)	160.2 (158.2, 162.2)	141.2 (138.5, 144.1)	109.4 (103.5, 115.4)	135.3 (131.9, 138.7)	156.6 (154.5, 158.6)	159.7 (157.7, 161.6)
urban	143.4 (141.1, 145.6)	110.9 (106.0, 115.9)	137.4 (134.6, 140.2)	158.9 (156.9, 160.9)	162.0 (160.0, 164.0)	143.2 (140.4, 146.0)	111.9 (106.1, 118.0)	137.4 (134.0, 140.9)	158.3 (156.2, 160.3)	161.0 (159.0, 163.0)
urban-rural difference	2.4 (1.3, 3.4)	3.0 (1.4, 4.5)	2.5 (1.4, 3.7)	2.1 (1.2, 3.0)	1.8 (0.8, 2.7)	2.0 (0.9, 3.0)	2.6 (1.0, 4.2)	2.1 (1.0, 3.3)	1.7 (0.8, 2.6)	1.3 (0.4, 2.3)
Nigeria										
rural	137.7 (136.2, 139.2)	104.5 (102.0, 106.9)	130.9 (129.1, 132.8)	153.9 (152.2, 155.6)	158.2 (156.2, 160.3)	139.5 (137.7, 141.3)	107.8 (104.7, 110.9)	133.1 (130.9, 135.3)	155.0 (153.4, 156.6)	158.7 (157.2, 160.1)
urban	140.1 (138.5, 141.8)	107.7 (105.1, 110.2)	133.6 (131.7, 135.5)	156.0 (154.3, 157.7)	159.8 (157.8, 161.9)	141.5 (139.6, 143.5)	110.6 (107.1, 114.0)	135.4 (133.0, 137.8)	156.7 (155.1, 158.3)	159.9 (158.5, 161.4)
urban-rural difference	2.4 (1.5, 3.3)	3.2 (1.8, 4.7)	2.6 (1.6, 3.7)	2.1 (1.3, 2.9)	1.6 (0.8, 2.4)	2.0 (1.1, 2.9)	2.8 (1.4, 4.3)	2.2 (1.2, 3.3)	1.7 (0.9, 2.4)	1.2 (0.4, 2.0)
Sao Tome and Principe										
rural	141.2 (138.2, 144.2)	108.2 (103.0, 113.3)	135.1 (131.7, 138.5)	157.2 (154.1, 160.3)	160.0 (156.7, 163.6)	143.7 (140.7, 146.8)	111.8 (105.8, 118.1)	137.9 (134.2, 141.7)	159.1 (156.8, 161.4)	161.4 (159.6, 163.2)
urban	142.6 (139.6, 145.5)	110.5 (105.4, 115.7)	136.7 (133.4, 140.1)	158.1 (155.2, 161.2)	160.5 (157.1, 163.9)	144.6 (141.5, 147.7)	113.6 (107.5, 119.9)	139.1 (135.3, 142.9)	159.6 (157.4, 161.9)	161.4 (159.6, 163.2)
urban-rural difference	1.4 (0.2, 2.6)	2.3 (0.7, 4.0)	1.6 (0.4, 2.9)	1.0 (-0.2, 2.1)	0.5 (-0.8, 1.7)	0.9 (-0.2, 2.1)	1.9 (0.1, 3.5)	1.2 (-0.1, 2.4)	0.5 (-0.6, 1.6)	0.0 (-1.2, 1.2)

Girls	Mean height in 1990 (cm)					Mean height in 2020 (cm)				
	Age-standardised	5 years	10 years	15 years	19 years	Age-standardised	5 years	10 years	15 years	19 years
Senegal										
rural	143.2 (140.7, 145.7)	110.4 (105.4, 115.2)	137.0 (134.2, 139.9)	159.1 (156.6, 161.6)	162.2 (159.2, 165.0)	144.3 (141.4, 147.0)	111.5 (105.4, 117.5)	138.0 (134.6, 141.4)	160.1 (158.0, 162.2)	163.4 (161.6, 165.1)
urban	145.4 (142.9, 147.9)	113.1 (108.1, 117.9)	139.4 (136.6, 142.3)	161.0 (158.6, 163.6)	163.8 (160.9, 166.8)	146.0 (143.2, 148.9)	113.8 (107.7, 119.7)	140.0 (136.5, 143.5)	161.6 (159.6, 163.7)	164.6 (162.9, 166.4)
urban-rural difference	2.2 (1.2, 3.2)	2.7 (1.2, 4.3)	2.3 (1.2, 3.4)	2.0 (1.1, 2.9)	1.7 (0.7, 2.7)	1.8 (0.8, 2.8)	2.3 (0.7, 3.9)	1.9 (0.8, 3.1)	1.5 (0.7, 2.4)	1.3 (0.4, 2.1)
Sierra Leone										
rural	138.3 (135.7, 140.8)	105.6 (100.8, 110.4)	132.2 (129.4, 135.0)	153.9 (151.3, 156.5)	157.2 (153.9, 160.6)	139.1 (136.8, 141.3)	107.8 (102.2, 113.3)	133.3 (130.5, 136.2)	154.1 (152.7, 155.6)	156.9 (155.5, 158.4)
urban	140.6 (138.1, 143.2)	108.5 (103.6, 113.4)	134.7 (131.8, 137.6)	156.1 (153.5, 158.7)	159.1 (155.8, 162.5)	141.0 (138.7, 143.3)	110.2 (104.7, 115.6)	135.4 (132.5, 138.2)	155.8 (154.4, 157.3)	158.3 (156.9, 159.8)
urban-rural difference	2.4 (1.3, 3.4)	2.9 (1.3, 4.4)	2.5 (1.4, 3.7)	2.2 (1.2, 3.2)	1.9 (0.8, 3.0)	1.9 (1.0, 2.8)	2.4 (0.9, 3.9)	2.0 (1.0, 3.1)	1.7 (0.9, 2.5)	1.4 (0.6, 2.3)
Togo										
rural	140.8 (138.5, 143.2)	108.4 (103.6, 113.5)	135.1 (132.1, 138.0)	156.5 (154.4, 158.5)	158.5 (156.4, 160.7)	141.7 (139.0, 144.4)	109.7 (103.9, 115.6)	136.0 (132.7, 139.3)	157.2 (155.3, 159.1)	159.3 (157.5, 161.2)
urban	142.2 (139.8, 144.6)	110.6 (105.7, 115.6)	136.6 (133.7, 139.6)	157.5 (155.4, 159.6)	159.2 (157.1, 161.3)	142.6 (139.9, 145.3)	111.3 (105.6, 117.3)	137.1 (133.8, 140.5)	157.8 (155.8, 159.7)	159.5 (157.7, 161.3)
urban-rural difference	1.4 (0.3, 2.5)	2.1 (0.5, 3.8)	1.6 (0.4, 2.8)	1.0 (0.0, 2.1)	0.6 (-0.5, 1.8)	0.9 (-0.2, 2.0)	1.6 (0.0, 3.3)	1.1 (-0.1, 2.3)	0.6 (-0.5, 1.5)	0.2 (-0.9, 1.2)

Boys	Mean height in 1990 (cm)					Mean height in 2020 (cm)				
	Age-standardised	5 years	10 years	15 years	19 years	Age-standardised	5 years	10 years	15 years	19 years
**Central and eastern Europe**										
Central Europe										
Albania										
rural	148.8 (146.0, 151.6)	113.3 (109.8, 116.8)	139.0 (136.4, 141.7)	168.9 (165.3, 172.6)	174.1 (169.0, 179.1)	150.2 (148.6, 152.0)	117.2 (114.2, 120.2)	141.1 (139.0, 143.2)	169.3 (167.7, 170.8)	173.2 (171.5, 175.1)
urban	151.0 (148.2, 153.7)	115.7 (112.2, 119.1)	141.2 (138.6, 143.9)	171.0 (167.4, 174.6)	176.0 (170.9, 181.0)	151.9 (150.3, 153.6)	119.1 (116.2, 122.1)	142.8 (140.7, 145.0)	170.9 (169.3, 172.5)	174.7 (172.9, 176.5)
urban-rural difference	2.1 (0.7, 3.4)	2.3 (0.8, 3.8)	2.2 (0.7, 3.5)	2.0 (0.5, 3.4)	1.9 (0.2, 3.5)	1.7 (0.9, 2.6)	1.9 (0.7, 3.2)	1.8 (0.9, 2.7)	1.6 (0.8, 2.5)	1.5 (0.5, 2.5)
Bosnia and Herzegovina										
rural	153.6 (149.4, 157.7)	117.8 (109.0, 127.1)	143.5 (139.1, 147.9)	173.6 (169.7, 177.6)	180.0 (175.6, 184.6)	155.6 (151.1, 160.2)	120.2 (110.1, 130.5)	145.6 (140.4, 150.8)	175.5 (171.7, 179.3)	181.6 (178.1, 185.2)
urban	155.3 (151.2, 159.4)	119.4 (110.6, 128.6)	145.2 (140.7, 149.6)	175.5 (171.6, 179.4)	182.0 (177.5, 186.6)	156.5 (151.9, 161.3)	120.9 (110.9, 131.1)	146.5 (141.2, 151.7)	176.5 (172.6, 180.5)	182.8 (179.2, 186.5)
urban-rural difference	1.8 (0.1, 3.4)	1.5 (-0.4, 3.6)	1.7 (0.0, 3.4)	1.9 (0.2, 3.6)	2.0 (0.2, 3.8)	0.9 (-0.8, 2.6)	0.7 (-1.5, 2.9)	0.8 (-0.9, 2.7)	1.0 (-0.6, 2.6)	1.1 (-0.5, 2.8)
Bulgaria										
rural	150.1 (147.5, 152.5)	116.3 (113.6, 119.0)	139.7 (137.4, 142.0)	169.7 (166.4, 172.8)	176.1 (171.4, 180.5)	151.2 (149.7, 152.8)	119.5 (116.9, 122.1)	141.2 (139.4, 143.0)	170.0 (167.8, 172.1)	175.8 (173.2, 178.4)
urban	152.5 (150.2, 154.7)	118.5 (116.1, 120.9)	142.1 (140.0, 144.1)	172.2 (169.3, 175.1)	178.7 (174.2, 183.2)	153.0 (151.5, 154.4)	121.0 (118.6, 123.5)	142.9 (141.2, 144.7)	171.8 (169.8, 173.8)	177.7 (175.2, 180.3)
urban-rural difference	2.4 (0.9, 3.9)	2.2 (0.7, 3.8)	2.4 (0.9, 3.9)	2.5 (0.8, 4.2)	2.7 (0.7, 4.5)	1.7 (0.6, 2.9)	1.5 (0.2, 2.9)	1.7 (0.5, 2.8)	1.8 (0.6, 3.0)	1.9 (0.5, 3.4)
Croatia										
rural	153.5 (151.4, 155.5)	117.6 (114.9, 120.5)	144.2 (142.0, 146.2)	173.7 (171.1, 176.3)	177.3 (173.7, 181.1)	155.3 (154.0, 156.6)	118.5 (116.0, 120.9)	145.7 (144.1, 147.2)	176.0 (174.3, 177.8)	180.2 (178.1, 182.3)
urban	154.9 (152.9, 157.0)	118.8 (116.0, 121.6)	145.5 (143.4, 147.7)	175.3 (172.7, 177.9)	179.1 (175.4, 182.9)	155.9 (154.6, 157.2)	118.8 (116.3, 121.2)	146.2 (144.7, 147.7)	176.8 (175.1, 178.5)	181.1 (179.1, 183.1)
urban-rural difference	1.5 (0.4, 2.5)	1.2 (-0.1, 2.4)	1.4 (0.3, 2.5)	1.6 (0.4, 2.7)	1.8 (0.4, 3.1)	0.6 (-0.3, 1.5)	0.3 (-0.8, 1.4)	0.5 (-0.4, 1.4)	0.7 (-0.2, 1.7)	0.9 (-0.2, 2.0)
Czechia										
rural	151.7 (150.4, 153.1)	115.4 (113.6, 117.1)	141.4 (139.8, 143.1)	172.2 (170.6, 173.8)	178.7 (176.9, 180.4)	154.0 (152.7, 155.3)	118.8 (116.7, 121.0)	144.0 (142.5, 145.5)	174.1 (172.3, 175.9)	180.0 (178.1, 181.9)
urban	153.0 (151.8, 154.1)	116.1 (114.4, 117.6)	142.5 (141.1, 143.9)	173.7 (172.3, 175.1)	180.5 (178.9, 182.0)	154.3 (153.1, 155.5)	118.5 (116.3, 120.7)	144.1 (142.6, 145.5)	174.6 (172.9, 176.2)	180.8 (179.0, 182.6)
urban-rural difference	1.3 (0.1, 2.4)	0.7 (-0.6, 2.0)	1.1 (-0.1, 2.3)	1.5 (0.3, 2.8)	1.8 (0.4, 3.3)	0.2 (-0.7, 1.2)	-0.3 (-1.4, 0.8)	0.1 (-0.8, 1.0)	0.5 (-0.5, 1.5)	0.8 (-0.4, 2.1)
Hungary										
rural	151.2 (148.0, 154.4)	113.8 (110.4, 117.2)	142.5 (139.4, 145.5)	171.5 (167.3, 175.4)	175.3 (169.8, 180.6)	154.0 (153.0, 154.9)	119.2 (117.0, 121.4)	146.1 (144.9, 147.2)	173.0 (172.0, 174.0)	175.3 (174.0, 176.7)
urban	153.9 (150.6, 157.0)	115.9 (112.6, 119.2)	145.0 (142.0, 147.9)	174.3 (170.3, 178.2)	178.5 (173.0, 183.9)	155.6 (154.7, 156.6)	120.3 (118.1, 122.6)	147.6 (146.5, 148.7)	174.9 (174.0, 175.8)	177.5 (176.3, 178.7)
urban-rural difference	2.6 (1.2, 4.1)	2.1 (0.6, 3.6)	2.5 (1.1, 3.9)	2.9 (1.3, 4.5)	3.2 (1.4, 5.0)	1.7 (1.1, 2.3)	1.1 (0.2, 2.1)	1.5 (0.9, 2.2)	1.9 (1.2, 2.6)	2.2 (1.2, 3.2)
North Macedonia										
rural	148.6 (144.9, 152.4)	115.9 (112.1, 119.9)	138.3 (134.8, 141.9)	167.8 (163.1, 172.7)	173.9 (167.1, 180.7)	150.4 (148.2, 152.5)	118.7 (115.4, 122.2)	140.3 (138.1, 142.5)	169.1 (165.8, 172.4)	174.9 (169.9, 179.7)
urban	150.7 (147.1, 154.5)	117.9 (114.0, 121.9)	140.4 (136.9, 144.1)	170.0 (165.2, 175.0)	176.2 (169.4, 183.1)	151.7 (149.6, 153.9)	119.9 (116.5, 123.3)	141.6 (139.4, 143.8)	170.6 (167.2, 173.9)	176.4 (171.4, 181.4)
urban-rural difference	2.1 (0.6, 3.7)	1.9 (0.5, 3.4)	2.1 (0.6, 3.6)	2.2 (0.5, 3.9)	2.3 (0.4, 4.4)	1.3 (0.3, 2.4)	1.1 (0.0, 2.4)	1.3 (0.3, 2.4)	1.4 (0.2, 2.7)	1.5 (0.1, 3.1)
Montenegro										
rural	153.4 (150.1, 156.7)	118.8 (114.3, 123.3)	143.2 (139.9, 146.5)	173.4 (169.2, 177.4)	179.0 (173.1, 184.8)	155.6 (154.5, 156.7)	119.8 (117.9, 121.6)	145.0 (143.6, 146.4)	176.3 (174.7, 177.8)	182.6 (180.6, 184.7)
urban	155.4 (152.2, 158.8)	120.7 (116.2, 125.1)	145.2 (142.0, 148.5)	175.5 (171.4, 179.5)	181.3 (175.5, 186.9)	156.8 (155.8, 157.9)	120.8 (119.0, 122.6)	146.2 (144.8, 147.5)	177.6 (176.1, 179.0)	184.0 (182.0, 186.0)
urban-rural difference	2.0 (0.5, 3.6)	1.9 (0.3, 3.4)	2.0 (0.5, 3.5)	2.1 (0.4, 3.8)	2.2 (0.2, 4.2)	1.2 (0.4, 2.1)	1.1 (0.1, 2.1)	1.2 (0.3, 2.0)	1.3 (0.3, 2.3)	1.4 (0.1, 2.7)
Poland										
rural	150.2 (148.9, 151.5)	112.8 (111.1, 114.6)	140.6 (139.2, 141.9)	170.6 (169.1, 172.0)	176.7 (175.3, 178.2)	152.3 (151.3, 153.3)	113.9 (112.2, 115.6)	142.6 (141.5, 143.8)	173.0 (171.9, 174.0)	179.0 (177.8, 180.2)
urban	151.2 (150.0, 152.5)	113.6 (111.9, 115.3)	141.5 (140.2, 142.8)	171.7 (170.3, 173.1)	178.0 (176.6, 179.4)	152.6 (151.7, 153.5)	114.1 (112.4, 115.7)	142.9 (141.8, 144.0)	173.4 (172.4, 174.5)	179.6 (178.4, 180.8)
urban-rural difference	1.0 (0.1, 1.9)	0.8 (-0.3, 1.8)	0.9 (0.0, 1.8)	1.1 (0.2, 2.0)	1.2 (0.3, 2.2)	0.3 (-0.4, 1.1)	0.1 (-0.8, 1.1)	0.3 (-0.5, 1.0)	0.4 (-0.3, 1.2)	0.6 (-0.2, 1.4)
Romania										
rural	146.5 (144.3, 148.8)	109.5 (107.4, 111.7)	137.1 (135.0, 139.3)	166.5 (163.8, 169.3)	172.6 (168.5, 176.6)	150.3 (149.2, 151.4)	113.7 (111.7, 115.5)	141.2 (139.9, 142.4)	170.1 (168.8, 171.4)	175.5 (173.7, 177.2)
urban	150.7 (148.5, 153.0)	112.7 (110.5, 114.9)	141.0 (138.8, 143.2)	171.2 (168.4, 174.0)	177.9 (173.9, 181.9)	152.7 (151.6, 153.7)	115.0 (113.2, 116.8)	143.3 (142.1, 144.4)	172.9 (171.6, 174.3)	178.9 (177.2, 180.7)
urban-rural difference	4.2 (3.1, 5.5)	3.2 (1.9, 4.5)	3.9 (2.7, 5.2)	4.7 (3.4, 6.0)	5.3 (3.9, 6.8)	2.4 (1.5, 3.2)	1.3 (0.3, 2.3)	2.1 (1.2, 2.9)	2.8 (1.9, 3.8)	3.4 (2.4, 4.6)
Serbia										
rural	152.9 (150.9, 154.8)	118.1 (115.3, 121.0)	143.0 (141.0, 145.1)	172.5 (170.3, 174.8)	178.2 (175.6, 180.9)	154.0 (152.6, 155.3)	118.6 (115.9, 121.3)	143.9 (142.3, 145.4)	174.0 (172.4, 175.6)	180.3 (178.3, 182.2)
urban	154.5 (152.6, 156.5)	119.5 (116.7, 122.5)	144.7 (142.7, 146.7)	174.3 (172.1, 176.6)	180.2 (177.4, 182.8)	154.9 (153.7, 156.3)	119.3 (116.7, 122.0)	144.8 (143.2, 146.3)	175.0 (173.6, 176.6)	181.4 (179.6, 183.3)
urban-rural difference	1.7 (0.6, 2.7)	1.5 (0.2, 2.7)	1.6 (0.5, 2.7)	1.8 (0.7, 2.9)	1.9 (0.7, 3.1)	0.9 (0.1, 1.8)	0.7 (-0.4, 1.8)	0.9 (0.0, 1.8)	1.1 (0.2, 1.9)	1.2 (0.2, 2.2)
Slovakia										
rural	151.4 (149.8, 152.9)	114.5 (112.3, 116.6)	141.1 (139.4, 142.8)	172.2 (170.3, 174.0)	177.9 (175.4, 180.4)	154.3 (152.9, 155.7)	117.7 (114.7, 120.7)	144.2 (142.6, 145.8)	175.0 (173.3, 176.8)	180.4 (178.4, 182.5)
urban	152.9 (151.4, 154.3)	115.9 (113.8, 117.9)	142.5 (140.9, 144.1)	173.8 (171.9, 175.5)	179.7 (177.2, 182.1)	155.2 (153.7, 156.6)	118.4 (115.4, 121.4)	145.0 (143.4, 146.5)	176.0 (174.2, 177.7)	181.5 (179.5, 183.6)
urban-rural difference	1.5 (0.3, 2.6)	1.3 (0.1, 2.5)	1.5 (0.3, 2.5)	1.6 (0.3, 2.8)	1.7 (0.2, 3.2)	0.9 (-0.2, 2.0)	0.7 (-0.6, 2.0)	0.8 (-0.2, 1.9)	1.0 (-0.2, 2.1)	1.1 (-0.2, 2.4)
Slovenia										

Boys	Mean height in 1990 (cm)					Mean height in 2020 (cm)				
	Age-standardised	5 years	10 years	15 years	19 years	Age-standardised	5 years	10 years	15 years	19 years
rural	152.3 (151.7, 152.9)	117.0 (116.0, 117.9)	142.5 (141.8, 143.1)	172.6 (171.9, 173.3)	177.1 (176.1, 178.1)	155.0 (154.1, 155.8)	118.5 (116.8, 120.3)	144.9 (143.9, 145.8)	175.8 (174.9, 176.6)	180.7 (179.8, 181.7)
urban	153.3 (152.7, 153.9)	117.8 (116.9, 118.7)	143.4 (142.8, 144.0)	173.7 (173.0, 174.4)	178.3 (177.4, 179.2)	155.3 (154.4, 156.1)	118.6 (116.8, 120.3)	145.1 (144.2, 146.0)	176.2 (175.4, 177.0)	181.2 (180.3, 182.1)
urban-rural difference	1.0 (0.2, 1.9)	0.8 (-0.2, 1.8)	1.0 (0.1, 1.8)	1.1 (0.1, 2.1)	1.2 (-0.1, 2.5)	0.3 (-0.6, 1.2)	0.1 (-1.2, 1.3)	0.2 (-0.7, 1.2)	0.4 (-0.6, 1.3)	0.5 (-0.7, 1.6)
Eastern Europe										
Belarus										
rural	147.7 (142.6, 153.0)	110.6 (101.2, 119.8)	137.2 (131.9, 142.6)	167.7 (162.2, 173.4)	175.9 (169.4, 182.6)	151.8 (147.0, 156.6)	115.8 (105.0, 126.3)	141.7 (136.1, 147.2)	171.3 (167.5, 175.1)	178.6 (175.8, 181.4)
urban	149.8 (144.7, 155.0)	112.4 (102.9, 121.6)	139.2 (133.8, 144.5)	169.9 (164.5, 175.5)	178.3 (171.6, 184.9)	153.0 (148.2, 157.8)	116.8 (105.8, 127.5)	142.8 (137.3, 148.3)	172.6 (168.8, 176.4)	180.1 (177.2, 182.8)
urban-rural difference	2.1 (0.3, 3.9)	1.8 (-0.2, 3.9)	2.0 (0.1, 3.9)	2.2 (0.3, 4.1)	2.4 (0.3, 4.4)	1.2 (-0.5, 3.0)	1.0 (-1.3, 3.2)	1.1 (-0.7, 3.0)	1.3 (-0.3, 3.0)	1.5 (-0.2, 3.1)
Estonia										
rural	151.4 (149.2, 153.5)	111.5 (107.9, 114.9)	141.0 (138.2, 143.5)	172.2 (169.9, 174.6)	180.5 (178.2, 182.8)	155.9 (154.4, 157.3)	117.3 (114.7, 119.7)	146.0 (144.4, 147.7)	176.1 (174.4, 177.8)	183.1 (181.0, 185.2)
urban	152.5 (150.4, 154.5)	112.2 (108.7, 115.5)	141.9 (139.4, 144.5)	173.5 (171.3, 175.9)	182.0 (179.7, 184.3)	155.9 (154.5, 157.3)	116.9 (114.3, 119.5)	145.9 (144.3, 147.6)	176.3 (174.7, 177.9)	183.5 (181.5, 185.6)
urban-rural difference	1.1 (-0.1, 2.3)	0.7 (-0.9, 2.2)	1.0 (-0.3, 2.3)	1.3 (0.1, 2.5)	1.5 (0.3, 2.8)	0.0 (-0.8, 0.9)	-0.4 (-1.6, 0.8)	-0.1 (-1.0, 0.8)	0.2 (-0.6, 1.1)	0.4 (-0.5, 1.4)
Latvia										
rural	149.9 (147.1, 152.8)	112.9 (109.2, 116.5)	138.9 (135.9, 142.0)	169.8 (166.2, 173.6)	179.2 (174.7, 183.7)	153.8 (152.1, 155.5)	118.3 (115.7, 121.0)	143.4 (141.5, 145.2)	173.2 (170.7, 175.6)	181.4 (178.0, 184.9)
urban	151.8 (149.1, 154.7)	114.6 (111.0, 118.2)	140.8 (137.8, 143.8)	172.0 (168.4, 175.7)	181.4 (176.9, 185.9)	154.8 (153.2, 156.5)	119.0 (116.3, 121.6)	144.3 (142.4, 146.1)	174.3 (171.9, 176.7)	182.6 (179.3, 186.1)
urban-rural difference	2.0 (0.5, 3.5)	1.7 (0.2, 3.2)	1.9 (0.5, 3.4)	2.1 (0.5, 3.8)	2.3 (0.5, 4.2)	1.0 (0.0, 1.9)	0.7 (-0.5, 1.8)	0.9 (-0.1, 1.8)	1.1 (0.0, 2.2)	1.3 (-0.1, 2.7)
Lithuania										
rural	150.0 (147.5, 152.5)	115.7 (111.7, 119.7)	139.3 (136.3, 142.2)	169.2 (166.2, 172.2)	177.6 (174.8, 180.6)	154.0 (152.4, 155.5)	119.8 (116.8, 123.0)	143.3 (141.5, 145.1)	173.0 (170.8, 175.2)	181.3 (178.1, 184.6)
urban	152.2 (149.6, 154.7)	117.4 (113.4, 121.4)	141.3 (138.3, 144.3)	171.5 (168.5, 174.6)	180.1 (177.0, 183.4)	155.0 (153.5, 156.6)	120.5 (117.4, 123.7)	144.3 (142.5, 146.1)	174.3 (172.0, 176.5)	182.7 (179.6, 186.1)
urban-rural difference	2.1 (0.7, 3.6)	1.7 (0.3, 3.2)	2.0 (0.6, 3.4)	2.3 (0.7, 3.8)	2.5 (0.7, 4.3)	1.1 (0.1, 2.0)	0.7 (-0.4, 1.8)	1.0 (0.0, 1.9)	1.2 (0.2, 2.3)	1.5 (0.1, 2.8)
Moldova										
rural	146.2 (142.6, 149.8)	110.6 (105.4, 116.0)	135.4 (131.7, 139.0)	165.7 (160.9, 170.6)	174.3 (167.8, 180.6)	149.9 (147.2, 152.6)	116.5 (110.9, 122.2)	139.8 (136.5, 143.1)	168.6 (165.5, 171.5)	175.8 (173.1, 178.6)
urban	148.5 (144.9, 152.1)	112.7 (107.4, 118.1)	137.7 (134.1, 141.2)	168.2 (163.3, 173.0)	176.9 (170.4, 183.1)	151.4 (148.7, 154.0)	117.7 (112.1, 123.3)	141.2 (137.8, 144.5)	170.1 (167.0, 173.0)	177.5 (174.7, 180.3)
urban-rural difference	2.3 (0.7, 4.0)	2.1 (0.4, 3.9)	2.3 (0.7, 3.9)	2.4 (0.8, 4.2)	2.6 (0.7, 4.6)	1.4 (0.1, 2.8)	1.2 (-0.6, 2.9)	1.4 (-0.1, 2.8)	1.5 (0.2, 2.9)	1.7 (0.2, 3.2)
Russian Federation										
rural	144.7 (143.8, 145.6)	106.9 (105.7, 108.1)	134.4 (133.4, 135.3)	164.4 (163.4, 165.4)	173.9 (172.6, 175.1)	149.5 (147.7, 151.3)	112.4 (110.0, 114.9)	139.5 (137.5, 141.5)	168.9 (167.1, 170.8)	177.6 (175.8, 179.4)
urban	148.1 (147.2, 148.9)	110.2 (109.0, 111.3)	137.7 (136.7, 138.6)	167.8 (166.7, 168.8)	177.3 (176.0, 178.5)	152.4 (150.8, 153.9)	115.2 (113.1, 117.5)	142.3 (140.6, 144.1)	171.8 (170.2, 173.4)	180.6 (178.9, 182.2)
urban-rural difference	3.3 (2.8, 3.9)	3.3 (2.5, 4.0)	3.3 (2.7, 3.9)	3.4 (2.8, 4.0)	3.4 (2.7, 4.1)	2.9 (1.8, 4.0)	2.8 (1.6, 4.1)	2.8 (1.7, 4.0)	2.9 (1.8, 4.0)	2.9 (1.8, 4.1)
Ukraine										
rural	147.5 (144.1, 150.8)	109.7 (106.1, 113.6)	136.3 (133.1, 139.6)	167.8 (163.6, 171.9)	177.6 (171.9, 183.2)	152.4 (151.0, 153.7)	115.4 (113.0, 117.9)	141.7 (140.2, 143.1)	172.3 (170.9, 173.6)	181.1 (179.2, 183.0)
urban	148.7 (145.4, 152.1)	110.8 (107.0, 114.5)	137.5 (134.2, 140.7)	169.2 (165.1, 173.2)	179.0 (173.3, 184.8)	152.5 (151.3, 153.8)	115.4 (113.0, 117.8)	141.8 (140.4, 143.2)	172.5 (171.3, 173.8)	181.5 (179.6, 183.4)
urban-rural difference	1.2 (-0.3, 2.9)	1.0 (-0.6, 2.7)	1.2 (-0.4, 2.8)	1.3 (-0.3, 3.0)	1.4 (-0.4, 3.3)	0.2 (-0.7, 1.0)	0.0 (-1.2, 1.1)	0.1 (-0.8, 1.0)	0.3 (-0.6, 1.1)	0.4 (-0.7, 1.4)
**Central Asia, Middle East and north Africa**										
Central Asia										
Armenia										
rural	145.4 (142.2, 148.6)	116.9 (110.1, 123.7)	135.2 (131.9, 138.7)	163.0 (159.1, 166.8)	169.4 (164.6, 174.1)	146.1 (144.0, 148.1)	116.1 (111.6, 120.7)	135.3 (132.8, 137.8)	164.5 (161.6, 167.3)	172.0 (169.1, 174.9)
urban	146.8 (143.8, 150.0)	118.4 (111.6, 125.3)	136.7 (133.3, 140.1)	164.4 (160.6, 168.2)	170.8 (166.0, 175.4)	147.7 (145.6, 149.7)	117.7 (113.2, 122.4)	136.9 (134.5, 139.3)	166.0 (163.2, 168.8)	173.5 (170.7, 176.3)
urban-rural difference	1.4 (-0.1, 3.0)	1.5 (-0.3, 3.3)	1.5 (-0.1, 3.1)	1.4 (-0.2, 3.1)	1.4 (-0.4, 3.2)	1.6 (0.3, 2.9)	1.7 (-0.1, 3.3)	1.6 (0.2, 2.9)	1.5 (0.3, 2.8)	1.5 (0.1, 2.9)
Azerbaijan										
rural	146.0 (142.7, 149.4)	115.4 (106.2, 124.8)	135.5 (131.6, 139.4)	164.3 (161.4, 167.2)	171.7 (169.0, 174.3)	147.2 (143.0, 151.3)	117.0 (106.4, 127.3)	136.6 (131.7, 141.4)	165.4 (162.3, 168.5)	172.8 (170.2, 175.3)
urban	147.2 (143.6, 150.7)	116.9 (107.7, 126.2)	136.8 (132.7, 140.8)	165.4 (162.3, 168.4)	172.6 (169.8, 175.4)	148.7 (144.6, 153.0)	118.8 (108.3, 129.2)	138.2 (133.5, 143.1)	166.9 (163.8, 170.0)	174.1 (171.6, 176.6)
urban-rural difference	1.2 (-0.4, 2.7)	1.4 (-0.6, 3.4)	1.2 (-0.4, 2.9)	1.1 (-0.5, 2.6)	0.9 (-0.7, 2.6)	1.6 (0.0, 3.2)	1.8 (-0.4, 4.0)	1.6 (-0.1, 3.4)	1.5 (0.0, 2.9)	1.3 (-0.1, 2.8)
Georgia										
rural	146.8 (143.5, 150.4)	118.7 (112.6, 125.0)	136.0 (132.4, 139.7)	164.4 (159.9, 169.0)	172.6 (166.6, 178.4)	147.0 (145.1, 148.9)	118.4 (114.5, 122.4)	135.7 (133.4, 138.1)	164.8 (162.0, 167.7)	173.9 (171.3, 176.4)
urban	148.3 (144.9, 151.7)	120.2 (114.2, 126.5)	137.4 (133.8, 141.1)	165.7 (161.2, 170.4)	173.9 (168.0, 179.7)	148.6 (146.8, 150.5)	120.1 (116.3, 124.1)	137.4 (135.0, 139.8)	166.4 (163.6, 169.3)	175.4 (172.8, 177.9)
urban-rural difference	1.4 (-0.3, 3.1)	1.5 (-0.3, 3.3)	1.4 (-0.2, 3.1)	1.4 (-0.4, 3.0)	1.3 (-0.6, 3.2)	1.6 (0.5, 2.8)	1.8 (0.3, 3.2)	1.7 (0.5, 2.8)	1.6 (0.4, 2.8)	1.5 (0.2, 2.9)
Kazakhstan										
rural	146.8 (143.1, 150.5)	112.8 (107.4, 118.3)	136.9 (133.3, 140.6)	166.4 (161.8, 171.0)	171.9 (165.9, 178.1)	148.2 (146.7, 149.7)	113.7 (111.1, 116.3)	138.2 (136.5, 139.8)	168.0 (166.2, 169.8)	173.5 (171.5, 175.6)
urban	148.8 (145.2, 152.5)	114.9 (109.4, 120.6)	139.0 (135.3, 142.7)	168.4 (163.9, 172.9)	173.9 (167.8, 179.9)	150.6 (149.1, 152.0)	116.2 (113.6, 118.8)	140.6 (139.0, 142.2)	170.3 (168.6, 172.1)	175.8 (173.8, 177.8)
urban-rural difference	2.0 (0.2, 3.7)	2.1 (0.2, 3.9)	2.0 (0.3, 3.7)	2.0 (0.1, 3.8)	1.9 (-0.1, 3.9)	2.4 (1.4, 3.3)	2.5 (1.3, 3.7)	2.4 (1.4, 3.4)	2.3 (1.3, 3.3)	2.3 (1.0, 3.5)
Kyrgyzstan										

Boys	Mean height in 1990 (cm)					Mean height in 2020 (cm)				
	Age-standardised	5 years	10 years	15 years	19 years	Age-standardised	5 years	10 years	15 years	19 years
rural	144.3 (140.9, 147.8)	114.2 (108.9, 119.6)	133.8 (130.2, 137.6)	162.4 (157.9, 167.2)	169.7 (163.5, 175.9)	145.1 (142.7, 147.4)	115.1 (111.2, 118.8)	134.5 (131.8, 137.2)	163.2 (160.3, 166.2)	170.6 (167.3, 174.0)
urban	146.0 (142.5, 149.6)	116.1 (110.7, 121.5)	135.6 (131.9, 139.4)	164.1 (159.5, 168.8)	171.2 (164.9, 177.5)	147.1 (144.7, 149.4)	117.3 (113.3, 121.1)	136.6 (133.8, 139.3)	165.2 (162.2, 168.2)	172.4 (169.1, 176.0)
urban-rural difference	1.7 (-0.2, 3.5)	1.9 (0.0, 3.8)	1.8 (-0.1, 3.5)	1.6 (-0.4, 3.6)	1.5 (-0.8, 3.7)	2.0 (0.7, 3.3)	2.2 (0.7, 3.8)	2.1 (0.8, 3.4)	1.9 (0.6, 3.3)	1.8 (0.2, 3.4)
Mongolia										
rural	142.9 (139.3, 146.3)	112.3 (103.2, 121.6)	132.5 (128.9, 136.1)	161.3 (158.0, 164.6)	168.0 (163.6, 172.3)	144.3 (141.1, 147.6)	113.5 (103.7, 123.5)	133.8 (130.0, 137.7)	162.9 (160.9, 164.8)	169.6 (167.8, 171.4)
urban	144.7 (141.2, 148.1)	114.2 (104.9, 123.5)	134.3 (130.7, 137.9)	163.1 (159.7, 166.3)	169.6 (165.2, 174.1)	146.3 (143.0, 149.6)	115.7 (105.7, 125.7)	135.8 (131.9, 139.8)	164.8 (162.8, 166.8)	171.5 (169.7, 173.4)
urban-rural difference	1.8 (0.3, 3.2)	1.9 (-0.1, 3.8)	1.8 (0.3, 3.3)	1.7 (0.3, 3.1)	1.6 (0.1, 3.2)	2.0 (0.7, 3.4)	2.1 (0.1, 4.2)	2.0 (0.5, 3.6)	1.9 (0.8, 3.1)	1.9 (0.8, 2.9)
Tajikistan										
rural	142.4 (138.7, 146.1)	112.7 (106.6, 118.9)	131.9 (128.0, 135.8)	160.5 (155.6, 165.3)	167.7 (161.4, 174.1)	143.0 (141.1, 145.0)	114.1 (110.0, 118.2)	132.5 (130.1, 135.0)	160.9 (157.8, 163.8)	167.9 (165.3, 170.6)
urban	143.4 (139.6, 147.3)	113.6 (107.6, 119.8)	132.9 (128.9, 136.9)	161.5 (156.5, 166.4)	168.7 (162.3, 175.1)	144.0 (142.0, 146.1)	115.1 (111.0, 119.1)	133.5 (131.0, 136.0)	161.9 (158.8, 164.9)	169.0 (166.1, 171.8)
urban-rural difference	1.0 (-1.0, 2.9)	0.9 (-1.0, 2.9)	1.0 (-0.9, 2.9)	1.0 (-1.0, 3.1)	1.0 (-1.3, 3.3)	1.0 (-0.4, 2.4)	1.0 (-0.7, 2.6)	1.0 (-0.4, 2.4)	1.0 (-0.4, 2.4)	1.0 (-0.6, 2.7)
Turkmenistan										
rural	145.0 (141.2, 148.5)	115.5 (109.2, 122.0)	134.3 (130.4, 138.1)	162.9 (157.9, 167.7)	170.7 (164.2, 177.0)	145.8 (143.5, 148.1)	115.5 (110.5, 120.6)	134.8 (131.9, 137.7)	164.1 (161.1, 167.1)	172.7 (170.3, 175.0)
urban	146.2 (142.5, 149.9)	116.8 (110.6, 123.4)	135.6 (131.7, 139.5)	164.1 (159.2, 169.0)	171.9 (165.5, 178.3)	147.2 (145.0, 149.6)	117.0 (112.1, 122.2)	136.2 (133.3, 139.2)	165.5 (162.6, 168.5)	174.0 (171.7, 176.4)
urban-rural difference	1.3 (-0.5, 3.1)	1.4 (-0.6, 3.3)	1.3 (-0.5, 3.1)	1.2 (-0.7, 3.1)	1.2 (-0.9, 3.3)	1.4 (0.1, 2.8)	1.5 (-0.2, 3.3)	1.5 (0.0, 2.9)	1.4 (0.1, 2.8)	1.3 (-0.1, 2.8)
Uzbekistan										
rural	145.4 (141.9, 149.1)	114.5 (105.4, 123.5)	135.0 (131.1, 138.9)	163.9 (160.7, 167.3)	170.6 (166.9, 174.3)	145.6 (141.5, 149.7)	115.8 (105.6, 126.2)	135.4 (130.6, 140.3)	163.7 (160.6, 166.7)	169.9 (167.7, 172.0)
urban	146.3 (142.8, 149.9)	115.6 (106.5, 124.7)	136.0 (132.3, 139.9)	164.8 (161.5, 168.2)	171.3 (167.7, 175.1)	147.1 (143.0, 151.2)	117.5 (107.3, 127.8)	136.9 (132.1, 141.8)	165.0 (162.0, 168.1)	171.1 (168.8, 173.4)
urban-rural difference	1.0 (-0.6, 2.4)	1.2 (-0.8, 3.1)	1.0 (-0.6, 2.5)	0.9 (-0.6, 2.3)	0.8 (-0.9, 2.3)	1.5 (-0.1, 3.0)	1.6 (-0.6, 3.9)	1.5 (-0.2, 3.2)	1.4 (0.0, 2.8)	1.3 (-0.1, 2.6)
Middle East and north Africa										
Algeria										
rural	143.4 (139.2, 147.7)	108.6 (99.9, 117.6)	134.2 (129.6, 138.9)	161.9 (157.9, 166.0)	169.5 (164.8, 174.3)	146.4 (142.1, 150.9)	110.5 (100.2, 120.6)	136.8 (131.7, 142.1)	165.5 (161.9, 169.0)	173.6 (171.0, 176.3)
urban	145.0 (141.0, 149.1)	110.2 (101.6, 119.2)	135.8 (131.4, 140.4)	163.4 (159.5, 167.5)	171.0 (166.3, 175.8)	148.0 (143.6, 152.4)	112.2 (102.1, 122.3)	138.4 (133.4, 143.7)	167.0 (163.7, 170.5)	175.2 (172.7, 177.6)
urban-rural difference	1.6 (-0.1, 3.3)	1.6 (-0.4, 3.7)	1.6 (-0.2, 3.4)	1.5 (-0.2, 3.3)	1.5 (-0.4, 3.4)	1.6 (0.0, 3.3)	1.7 (-0.5, 4.0)	1.6 (-0.1, 3.5)	1.6 (0.1, 3.1)	1.6 (0.0, 3.1)
Bahrain										
rural	143.2 (139.7, 146.8)	109.2 (100.5, 118.0)	134.6 (130.8, 138.5)	161.4 (158.0, 164.7)	167.0 (163.0, 171.1)	147.5 (143.9, 151.0)	111.8 (102.5, 120.9)	138.3 (134.4, 142.2)	166.6 (163.9, 169.1)	173.0 (169.8, 176.2)
urban	144.7 (141.5, 148.0)	110.9 (102.3, 119.3)	136.2 (132.6, 139.9)	162.8 (159.8, 165.9)	168.4 (164.6, 172.0)	149.2 (146.1, 152.4)	113.6 (104.6, 122.5)	140.0 (136.5, 143.7)	168.2 (166.1, 170.3)	174.5 (171.7, 177.3)
urban-rural difference	1.5 (-0.4, 3.5)	1.6 (-0.6, 3.9)	1.5 (-0.5, 3.6)	1.5 (-0.5, 3.5)	1.4 (-0.8, 3.5)	1.7 (-0.3, 3.6)	1.8 (-0.6, 4.2)	1.7 (-0.3, 3.7)	1.6 (-0.3, 3.4)	1.5 (-0.4, 3.4)
Egypt										
rural	142.4 (140.2, 144.6)	107.6 (104.0, 111.3)	131.5 (129.4, 133.7)	161.0 (158.5, 163.6)	172.6 (169.2, 176.1)	147.3 (144.6, 150.1)	116.0 (109.7, 122.4)	137.5 (134.3, 140.8)	164.6 (162.7, 166.5)	173.7 (172.1, 175.4)
urban	143.6 (141.3, 145.9)	109.0 (105.4, 112.8)	132.9 (130.6, 135.0)	162.2 (159.6, 164.8)	173.8 (170.3, 177.4)	148.7 (146.1, 151.4)	117.5 (111.5, 123.7)	138.9 (135.7, 142.2)	165.9 (164.0, 167.8)	175.0 (173.3, 176.6)
urban-rural difference	1.3 (-0.1, 2.6)	1.4 (-0.3, 3.0)	1.3 (-0.1, 2.7)	1.2 (-0.2, 2.5)	1.2 (-0.4, 2.7)	1.4 (0.3, 2.5)	1.5 (-0.2, 3.3)	1.4 (0.2, 2.7)	1.3 (0.3, 2.3)	1.3 (0.2, 2.3)
Iran										
rural	142.4 (141.3, 143.5)	106.9 (105.5, 108.2)	132.5 (131.3, 133.7)	161.3 (160.1, 162.5)	170.3 (168.6, 172.0)	145.1 (144.1, 146.2)	108.1 (105.7, 110.4)	135.0 (133.8, 136.1)	164.9 (163.9, 165.8)	173.7 (172.7, 174.8)
urban	144.9 (143.8, 145.9)	108.9 (107.6, 110.2)	134.8 (133.7, 136.0)	163.9 (162.8, 165.1)	173.2 (171.5, 174.8)	147.5 (146.5, 148.6)	110.0 (107.7, 112.5)	137.3 (136.1, 138.4)	167.5 (166.5, 168.4)	176.5 (175.5, 177.6)
urban-rural difference	2.5 (1.8, 3.2)	2.0 (1.2, 2.9)	2.3 (1.6, 3.1)	2.6 (2.0, 3.3)	2.9 (2.1, 3.6)	2.4 (1.8, 3.0)	2.0 (1.2, 2.8)	2.3 (1.7, 2.9)	2.6 (2.0, 3.2)	2.8 (2.2, 3.4)
Iraq										
rural	145.1 (141.6, 148.6)	110.1 (101.6, 118.6)	136.3 (132.5, 140.2)	163.7 (160.4, 167.1)	169.9 (165.9, 174.0)	148.3 (144.4, 152.2)	113.5 (104.1, 122.8)	139.4 (135.0, 143.9)	166.9 (163.9, 170.0)	172.9 (170.4, 175.5)
urban	146.4 (143.0, 149.9)	111.6 (103.2, 120.2)	137.6 (134.1, 141.4)	164.9 (161.8, 168.1)	171.0 (167.1, 174.9)	149.8 (146.1, 153.5)	115.2 (105.9, 124.5)	141.0 (136.7, 145.2)	168.3 (165.6, 171.1)	174.2 (171.8, 176.6)
urban-rural difference	1.3 (-0.5, 3.1)	1.5 (-0.5, 3.5)	1.4 (-0.4, 3.2)	1.3 (-0.6, 3.0)	1.1 (-0.8, 3.1)	1.5 (-0.2, 3.2)	1.7 (-0.5, 4.0)	1.5 (-0.3, 3.3)	1.4 (-0.2, 2.9)	1.3 (-0.3, 2.9)
Jordan										
rural	144.2 (141.6, 146.6)	104.8 (100.9, 108.5)	134.6 (132.1, 137.2)	164.2 (160.9, 167.4)	173.0 (168.5, 177.2)	147.5 (145.3, 149.7)	110.2 (106.0, 114.4)	138.4 (135.8, 141.1)	166.7 (164.6, 168.7)	174.0 (172.1, 175.9)
urban	145.3 (142.6, 147.8)	106.0 (102.3, 109.7)	135.8 (133.1, 138.3)	165.2 (161.8, 168.6)	173.9 (169.3, 178.4)	148.8 (146.8, 150.9)	111.7 (107.9, 115.6)	139.9 (137.4, 142.4)	168.0 (166.1, 169.9)	175.2 (173.6, 177.0)
urban-rural difference	1.1 (-0.4, 2.4)	1.3 (-0.4, 2.8)	1.1 (-0.3, 2.5)	1.0 (-0.5, 2.4)	0.9 (-0.8, 2.4)	1.4 (0.2, 2.6)	1.6 (-0.1, 3.2)	1.4 (0.1, 2.8)	1.3 (0.2, 2.5)	1.2 (0.0, 2.4)
Kuwait										
rural	145.9 (143.6, 148.2)	109.7 (106.9, 112.5)	137.2 (134.8, 139.6)	165.4 (162.7, 168.0)	169.8 (166.2, 173.4)	147.7 (144.9, 150.5)	111.5 (106.6, 116.5)	138.9 (135.6, 142.2)	167.3 (164.9, 169.7)	171.7 (169.4, 174.1)
urban	147.6 (146.2, 149.0)	111.5 (109.8, 113.2)	139.0 (137.6, 140.4)	167.1 (165.2, 169.0)	171.4 (168.5, 174.3)	149.4 (147.2, 151.6)	113.2 (108.7, 117.7)	140.6 (137.8, 143.3)	168.9 (167.3, 170.6)	173.3 (171.9, 174.8)
urban-rural difference	1.7 (-0.3, 3.7)	1.8 (-0.4, 4.0)	1.7 (-0.3, 3.8)	1.7 (-0.3, 3.7)	1.6 (-0.5, 3.9)	1.6 (-0.3, 3.6)	1.7 (-0.6, 4.1)	1.7 (-0.3, 3.7)	1.6 (-0.2, 3.5)	1.6 (-0.3, 3.5)
Lebanon										
rural	146.8 (144.7, 149.0)	108.5 (105.9, 111.0)	137.9 (135.6, 140.1)	166.5 (164.0, 169.0)	173.4 (170.8, 176.0)	151.1 (148.4, 153.8)	112.9 (108.0, 117.9)	142.1 (138.8, 145.4)	170.8 (168.4, 173.2)	177.4 (175.1, 179.7)
urban	148.6 (146.5, 150.6)	110.1 (107.4, 112.6)	139.6 (137.4, 141.7)	168.3 (165.9, 170.6)	175.3 (172.8, 177.8)	152.6 (150.0, 155.2)	114.3 (109.4, 119.3)	143.6 (140.4, 146.8)	172.4 (170.2, 174.6)	179.1 (176.8, 181.3)

Boys	Mean height in 1990 (cm)					Mean height in 2020 (cm)				
	Age-standardised	5 years	10 years	15 years	19 years	Age-standardised	5 years	10 years	15 years	19 years
urban-rural difference	1.7 (0.6, 2.9)	1.6 (0.2, 3.0)	1.7 (0.5, 2.9)	1.8 (0.6, 3.0)	1.9 (0.5, 3.3)	1.5 (0.2, 2.8)	1.4 (-0.3, 3.1)	1.5 (0.1, 2.9)	1.6 (0.3, 2.9)	1.7 (0.4, 3.0)
Libya										
rural	145.0 (140.6, 149.3)	109.3 (100.4, 118.2)	135.6 (131.1, 140.1)	163.8 (159.2, 168.2)	171.8 (166.4, 177.4)	147.9 (143.0, 152.7)	112.2 (102.1, 122.6)	138.3 (132.9, 143.9)	166.8 (162.5, 171.1)	174.7 (170.7, 178.6)
urban	146.6 (142.2, 150.7)	111.0 (102.2, 119.8)	137.2 (132.7, 141.6)	165.4 (160.9, 169.7)	173.3 (168.0, 178.6)	149.5 (144.7, 154.3)	113.8 (103.9, 124.2)	140.0 (134.6, 145.6)	168.4 (164.2, 172.5)	176.2 (172.4, 180.1)
urban-rural difference	1.6 (-0.3, 3.5)	1.7 (-0.5, 3.9)	1.6 (-0.3, 3.6)	1.5 (-0.4, 3.5)	1.5 (-0.7, 3.7)	1.6 (-0.3, 3.5)	1.7 (-0.7, 4.0)	1.6 (-0.4, 3.6)	1.6 (-0.2, 3.4)	1.5 (-0.3, 3.5)
Morocco										
rural	143.3 (139.8, 146.8)	110.5 (104.9, 116.1)	133.5 (129.9, 137.1)	161.1 (156.9, 165.4)	170.2 (164.3, 176.3)	145.9 (144.1, 147.7)	112.4 (109.5, 115.3)	135.7 (133.6, 137.8)	164.2 (162.1, 166.3)	173.9 (171.5, 176.2)
urban	145.1 (141.6, 148.6)	112.5 (107.0, 118.0)	135.4 (131.7, 138.9)	162.9 (158.7, 167.1)	171.9 (165.9, 177.9)	147.9 (146.1, 149.7)	114.6 (111.7, 117.5)	137.7 (135.7, 139.9)	166.2 (164.1, 168.3)	175.8 (173.3, 178.1)
urban-rural difference	1.8 (0.1, 3.5)	2.0 (0.2, 3.8)	1.9 (0.2, 3.6)	1.8 (0.0, 3.5)	1.7 (-0.3, 3.6)	2.0 (0.8, 3.2)	2.2 (0.6, 3.7)	2.1 (0.8, 3.3)	1.9 (0.8, 3.1)	1.9 (0.5, 3.2)
Occupied Palestinian Territory										
rural	144.3 (141.2, 147.5)	108.2 (99.8, 116.2)	134.8 (131.4, 138.2)	163.3 (160.5, 166.0)	171.6 (168.4, 174.7)	146.8 (142.9, 150.6)	110.9 (101.4, 120.3)	137.1 (132.8, 141.5)	165.8 (162.9, 168.6)	174.0 (171.1, 176.8)
urban	145.0 (141.9, 148.2)	109.1 (100.8, 117.4)	135.6 (132.3, 139.0)	163.9 (161.2, 166.7)	172.1 (168.8, 175.3)	147.8 (144.0, 151.5)	112.2 (102.9, 121.6)	138.2 (133.8, 142.5)	166.7 (164.0, 169.3)	174.8 (172.1, 177.3)
urban-rural difference	0.7 (-1.0, 2.4)	1.0 (-1.0, 3.0)	0.8 (-1.0, 2.5)	0.6 (-1.1, 2.3)	0.5 (-1.3, 2.2)	1.0 (-0.8, 2.8)	1.2 (-1.1, 3.6)	1.1 (-0.9, 3.0)	0.9 (-0.8, 2.6)	0.8 (-0.9, 2.5)
Oman										
rural	141.0 (137.3, 144.7)	105.2 (97.0, 113.9)	131.3 (127.7, 135.0)	159.9 (156.0, 163.8)	168.5 (163.3, 173.9)	144.3 (140.7, 147.8)	110.5 (101.1, 120.0)	135.1 (131.1, 139.3)	162.4 (160.0, 164.8)	169.7 (167.2, 172.2)
urban	142.5 (138.9, 146.1)	106.8 (98.6, 115.3)	132.8 (129.3, 136.3)	161.3 (157.5, 165.3)	169.9 (164.7, 175.4)	145.8 (142.4, 149.4)	112.1 (102.7, 121.9)	136.7 (132.7, 140.7)	163.9 (161.7, 166.0)	171.1 (168.9, 173.3)
urban-rural difference	1.5 (-0.2, 3.1)	1.6 (-0.5, 3.5)	1.5 (-0.3, 3.1)	1.4 (-0.3, 3.0)	1.4 (-0.5, 3.1)	1.5 (-0.2, 3.3)	1.6 (-0.6, 3.9)	1.5 (-0.3, 3.4)	1.5 (-0.1, 3.1)	1.4 (-0.2, 3.0)
Qatar										
rural	142.8 (138.7, 146.8)	107.3 (98.6, 115.7)	133.6 (129.4, 137.8)	161.6 (157.4, 166.0)	168.6 (163.1, 174.2)	146.1 (141.8, 150.4)	110.6 (100.7, 120.3)	136.8 (131.8, 141.9)	165.0 (161.6, 168.4)	171.8 (168.8, 174.9)
urban	144.3 (140.6, 148.0)	109.0 (100.5, 117.3)	135.2 (131.4, 139.0)	163.0 (159.1, 167.1)	170.0 (164.9, 175.3)	147.6 (143.5, 151.7)	112.3 (102.5, 122.1)	138.4 (133.6, 143.2)	166.5 (163.4, 169.5)	173.3 (170.7, 176.0)
urban-rural difference	1.5 (-0.4, 3.5)	1.6 (-0.6, 3.8)	1.6 (-0.4, 3.5)	1.5 (-0.5, 3.5)	1.4 (-0.7, 3.6)	1.6 (-0.3, 3.5)	1.7 (-0.7, 4.1)	1.6 (-0.4, 3.6)	1.5 (-0.3, 3.4)	1.5 (-0.4, 3.4)
Saudi Arabia										
rural	140.1 (138.4, 141.9)	106.6 (104.2, 109.1)	132.2 (130.3, 134.1)	157.8 (155.9, 159.7)	162.6 (160.2, 165.0)	142.7 (140.3, 145.3)	106.2 (101.4, 110.8)	133.4 (130.5, 136.4)	162.0 (160.0, 164.1)	169.4 (167.3, 171.4)
urban	141.3 (139.9, 142.7)	107.5 (105.4, 109.6)	133.3 (131.6, 135.0)	159.1 (157.4, 160.6)	164.0 (161.8, 166.2)	143.6 (141.4, 145.8)	106.7 (102.4, 111.1)	134.1 (131.4, 136.8)	163.0 (161.1, 164.9)	170.5 (168.6, 172.4)
urban-rural difference	1.2 (-0.3, 2.7)	0.9 (-1.0, 2.7)	1.1 (-0.5, 2.6)	1.3 (-0.2, 2.8)	1.5 (-0.1, 3.2)	0.8 (-0.6, 2.2)	0.5 (-1.5, 2.5)	0.8 (-0.8, 2.3)	1.0 (-0.3, 2.2)	1.2 (-0.1, 2.4)
Syrian Arab Republic										
rural	143.4 (136.8, 150.0)	107.8 (97.6, 117.9)	134.1 (127.5, 140.6)	162.3 (155.5, 168.9)	169.9 (161.6, 178.0)	146.4 (139.8, 153.2)	110.9 (99.9, 121.9)	137.0 (130.2, 144.0)	165.4 (159.1, 171.7)	172.8 (165.8, 180.0)
urban	145.0 (138.3, 151.6)	109.5 (99.3, 119.4)	135.7 (129.2, 142.1)	163.8 (157.0, 170.4)	171.4 (163.2, 179.6)	148.0 (141.3, 154.6)	112.6 (101.4, 123.4)	138.7 (131.8, 145.6)	166.9 (160.6, 173.2)	174.3 (167.3, 181.4)
urban-rural difference	1.6 (-0.4, 3.6)	1.7 (-0.6, 3.9)	1.6 (-0.4, 3.6)	1.5 (-0.5, 3.5)	1.5 (-0.7, 3.7)	1.6 (-0.3, 3.5)	1.7 (-0.7, 4.1)	1.6 (-0.3, 3.7)	1.6 (-0.3, 3.4)	1.5 (-0.4, 3.4)
Tunisia										
rural	144.1 (142.0, 146.1)	108.1 (105.7, 110.4)	134.9 (132.8, 137.1)	163.0 (160.6, 165.2)	170.3 (167.7, 172.8)	147.1 (144.3, 150.0)	109.3 (104.4, 114.1)	137.5 (134.2, 140.8)	166.9 (164.3, 169.5)	174.9 (172.0, 177.7)
urban	146.8 (144.8, 148.8)	110.7 (108.4, 113.0)	137.7 (135.5, 139.8)	165.7 (163.4, 168.0)	173.1 (170.5, 175.6)	149.5 (146.8, 152.2)	111.6 (106.8, 116.2)	139.9 (136.7, 143.0)	169.3 (166.7, 171.8)	177.3 (174.5, 180.1)
urban-rural difference	2.7 (1.6, 3.9)	2.7 (1.3, 4.1)	2.7 (1.6, 3.9)	2.8 (1.6, 4.0)	2.8 (1.4, 4.3)	2.4 (0.9, 3.7)	2.3 (0.4, 4.1)	2.4 (0.8, 3.8)	2.4 (1.0, 3.7)	2.4 (1.0, 3.8)
Turkey										
rural	145.9 (143.7, 148.2)	110.2 (106.0, 114.6)	136.5 (133.9, 139.1)	165.1 (162.3, 167.9)	172.2 (168.5, 176.0)	148.2 (146.4, 149.9)	111.9 (108.6, 115.2)	138.5 (136.0, 140.8)	167.7 (165.6, 169.6)	175.0 (173.0, 177.1)
urban	147.5 (145.2, 149.8)	112.2 (107.9, 116.6)	138.1 (135.5, 140.7)	166.4 (163.5, 169.3)	173.3 (169.4, 177.1)	150.1 (148.4, 151.8)	114.3 (111.0, 117.5)	140.5 (138.2, 142.8)	169.4 (167.4, 171.3)	176.5 (174.5, 178.4)
urban-rural difference	1.5 (0.2, 2.8)	2.0 (0.5, 3.6)	1.7 (0.3, 3.0)	1.3 (0.0, 2.6)	1.1 (-0.4, 2.5)	1.9 (0.8, 3.0)	2.4 (1.0, 3.8)	2.0 (0.9, 3.2)	1.7 (0.7, 2.7)	1.4 (0.3, 2.6)
United Arab Emirates										
rural	146.3 (142.8, 149.9)	113.1 (105.2, 121.3)	137.7 (134.4, 141.0)	164.2 (160.2, 168.3)	170.0 (164.4, 175.5)	150.1 (146.7, 153.6)	116.7 (107.7, 125.9)	141.4 (137.5, 145.3)	168.2 (165.8, 170.7)	173.8 (171.4, 176.3)
urban	147.5 (144.1, 150.9)	114.5 (106.7, 122.6)	138.9 (135.8, 142.1)	165.3 (161.3, 169.3)	170.9 (165.4, 176.4)	151.5 (148.3, 154.6)	118.2 (109.3, 127.3)	142.8 (139.2, 146.5)	169.5 (167.4, 171.4)	174.9 (172.9, 177.1)
urban-rural difference	1.2 (-0.7, 3.0)	1.4 (-0.6, 3.6)	1.3 (-0.6, 3.1)	1.1 (-0.8, 2.8)	1.0 (-1.0, 2.9)	1.4 (-0.4, 3.2)	1.6 (-0.6, 4.0)	1.4 (-0.4, 3.3)	1.2 (-0.5, 2.9)	1.1 (-0.6, 2.8)
Yemen										
rural	135.8 (133.0, 138.6)	98.1 (94.9, 101.3)	126.0 (123.3, 128.8)	155.2 (151.5, 158.8)	165.0 (160.3, 169.8)	138.0 (134.8, 141.1)	103.7 (98.4, 109.2)	129.0 (125.3, 132.8)	155.9 (153.0, 158.8)	163.9 (161.1, 166.7)
urban	136.9 (134.1, 139.7)	99.3 (96.1, 102.5)	127.2 (124.4, 129.9)	156.3 (152.6, 159.9)	166.1 (161.4, 170.9)	139.1 (136.0, 142.3)	104.9 (99.6, 110.4)	130.2 (126.4, 134.0)	157.0 (154.2, 159.8)	165.0 (162.3, 167.8)
urban-rural difference	1.1 (-0.1, 2.4)	1.2 (-0.2, 2.6)	1.2 (-0.1, 2.4)	1.1 (-0.2, 2.4)	1.1 (-0.4, 2.6)	1.2 (0.0, 2.3)	1.2 (-0.3, 2.7)	1.2 (0.0, 2.4)	1.1 (0.1, 2.3)	1.1 (-0.1, 2.3)
**East and southeast Asia**										
East Asia										
China										
rural	141.5 (140.8, 142.3)	105.5 (104.7, 106.4)	132.4 (131.6, 133.2)	161.4 (160.5, 162.2)	166.0 (165.0, 167.1)	150.2 (149.1, 151.3)	112.6 (110.2, 115.1)	140.9 (139.7, 142.1)	170.7 (169.7, 171.6)	175.4 (174.4, 176.5)
urban	144.9 (144.2, 145.7)	109.2 (108.3, 110.0)	135.9 (135.1, 136.7)	164.6 (163.8, 165.5)	169.1 (168.0, 170.1)	152.2 (151.1, 153.3)	115.0 (112.5, 117.4)	143.0 (141.8, 144.2)	172.6 (171.6, 173.5)	177.2 (176.2, 178.2)
urban-rural difference	3.4 (2.9, 3.9)	3.7 (3.1, 4.2)	3.5 (3.0, 3.9)	3.3 (2.8, 3.8)	3.1 (2.5, 3.7)	2.1 (1.4, 2.7)	2.3 (1.6, 3.1)	2.1 (1.5, 2.8)	1.9 (1.3, 2.5)	1.8 (1.1, 2.4)

Boys	Mean height in 1990 (cm)					Mean height in 2020 (cm)				
	Age-standardised	5 years	10 years	15 years	19 years	Age-standardised	5 years	10 years	15 years	19 years
North Korea										
rural	142.9 (135.3, 150.4)	106.5 (94.7, 118.3)	134.1 (126.4, 141.6)	162.8 (154.9, 170.4)	166.9 (157.4, 176.3)	148.4 (140.7, 156.0)	111.9 (99.5, 124.6)	139.7 (131.9, 147.5)	168.3 (160.9, 175.5)	172.2 (163.8, 180.3)
urban	145.6 (137.9, 153.1)	109.3 (97.6, 121.0)	136.8 (129.4, 144.3)	165.4 (157.5, 173.1)	169.4 (159.7, 178.9)	150.0 (142.3, 157.6)	113.8 (101.5, 126.4)	141.4 (133.5, 149.2)	169.8 (162.6, 176.9)	173.6 (165.4, 181.7)
urban-rural difference	2.7 (0.5, 4.7)	2.8 (0.6, 5.1)	2.7 (0.6, 4.8)	2.6 (0.4, 4.7)	2.5 (0.1, 4.9)	1.6 (-0.5, 3.7)	1.8 (-0.8, 4.4)	1.7 (-0.5, 3.9)	1.5 (-0.5, 3.6)	1.4 (-0.6, 3.5)
Taiwan										
rural	146.1 (143.9, 148.2)	108.1 (105.7, 110.6)	137.6 (135.6, 139.5)	166.5 (163.9, 169.1)	169.5 (165.6, 173.4)	150.3 (148.7, 152.0)	113.1 (110.0, 116.2)	142.1 (140.1, 144.1)	170.5 (169.0, 171.9)	172.9 (171.1, 174.8)
urban	148.2 (146.2, 150.2)	110.5 (108.3, 112.8)	139.8 (138.0, 141.7)	168.6 (166.2, 171.1)	171.5 (167.7, 175.3)	151.4 (149.8, 152.9)	114.3 (111.2, 117.4)	143.2 (141.3, 145.1)	171.4 (170.0, 172.7)	173.7 (172.0, 175.5)
urban-rural difference	2.2 (0.6, 3.6)	2.4 (0.7, 4.1)	2.2 (0.7, 3.7)	2.1 (0.4, 3.6)	1.9 (0.1, 3.7)	1.0 (-0.1, 2.1)	1.3 (-0.3, 2.8)	1.1 (-0.1, 2.3)	0.9 (-0.1, 1.9)	0.8 (-0.3, 1.9)
Southeast Asia										
Brunei Darussalam										
rural	140.8 (137.7, 144.1)	105.0 (101.1, 109.0)	132.3 (129.2, 135.5)	160.3 (156.3, 164.2)	163.3 (157.9, 168.6)	144.9 (142.8, 147.1)	110.5 (106.1, 115.2)	137.0 (134.4, 139.7)	163.7 (162.0, 165.5)	165.7 (164.0, 167.6)
urban	142.7 (139.4, 145.9)	107.0 (103.2, 111.0)	134.2 (131.1, 137.4)	162.1 (158.1, 166.0)	165.1 (159.7, 170.4)	145.9 (143.9, 148.0)	111.6 (107.2, 116.2)	138.0 (135.6, 140.5)	164.6 (163.0, 166.2)	166.6 (164.9, 168.3)
urban-rural difference	1.9 (0.4, 3.3)	2.0 (0.3, 3.7)	1.9 (0.5, 3.4)	1.8 (0.3, 3.4)	1.8 (0.1, 3.5)	1.0 (-0.3, 2.3)	1.1 (-0.7, 2.9)	1.0 (-0.4, 2.4)	0.9 (-0.3, 2.1)	0.9 (-0.4, 2.1)
Cambodia										
rural	137.3 (133.0, 141.5)	102.5 (94.0, 111.1)	128.1 (123.7, 132.5)	156.4 (151.9, 160.9)	161.2 (155.6, 167.0)	141.8 (136.9, 146.6)	107.1 (97.0, 117.1)	132.9 (127.4, 138.4)	160.8 (156.7, 165.0)	164.9 (161.3, 168.7)
urban	139.9 (135.6, 144.3)	105.4 (96.7, 114.0)	130.7 (126.2, 135.4)	158.9 (154.4, 163.6)	163.6 (157.9, 169.7)	143.7 (138.8, 148.7)	109.2 (99.2, 119.5)	134.8 (129.3, 140.5)	162.6 (158.4, 166.8)	166.6 (162.7, 170.4)
urban-rural difference	2.6 (0.7, 4.5)	2.8 (0.7, 4.9)	2.7 (0.7, 4.6)	2.5 (0.5, 4.5)	2.4 (0.2, 4.5)	1.9 (0.0, 3.8)	2.1 (-0.2, 4.5)	2.0 (0.0, 3.9)	1.8 (0.0, 3.6)	1.7 (-0.2, 3.5)
Indonesia										
rural	135.9 (134.6, 137.2)	102.3 (100.8, 103.8)	126.2 (124.8, 127.5)	154.7 (153.2, 156.1)	160.7 (158.8, 162.6)	140.1 (138.8, 141.3)	105.5 (102.9, 108.1)	130.4 (129.0, 131.8)	159.3 (158.2, 160.4)	164.7 (163.5, 166.0)
urban	139.1 (137.8, 140.5)	105.6 (104.1, 107.0)	129.4 (128.0, 130.8)	157.9 (156.4, 159.4)	163.9 (162.0, 165.7)	142.7 (141.5, 144.0)	108.2 (105.6, 110.7)	133.1 (131.6, 134.5)	162.0 (160.9, 163.1)	167.3 (166.1, 168.6)
urban-rural difference	3.2 (2.5, 3.9)	3.2 (2.4, 4.1)	3.2 (2.5, 4.0)	3.2 (2.4, 4.0)	3.2 (2.3, 4.0)	2.7 (2.0, 3.3)	2.7 (1.9, 3.5)	2.7 (2.0, 3.4)	2.6 (2.0, 3.3)	2.6 (1.8, 3.4)
Lao PDR										
rural	135.9 (131.6, 140.5)	100.5 (91.6, 109.8)	126.6 (122.0, 131.3)	155.3 (150.8, 160.1)	160.2 (154.4, 166.1)	140.2 (135.6, 144.9)	106.2 (96.1, 116.6)	131.6 (126.2, 136.9)	159.0 (155.0, 163.0)	162.4 (159.4, 165.5)
urban	138.5 (134.2, 142.9)	103.4 (94.6, 112.5)	129.2 (124.7, 133.9)	157.8 (153.2, 162.4)	162.6 (156.8, 168.5)	142.1 (137.4, 146.9)	108.4 (98.3, 118.8)	133.6 (128.2, 139.0)	160.8 (156.9, 164.9)	164.2 (160.9, 167.3)
urban-rural difference	2.6 (0.7, 4.5)	2.9 (0.7, 4.9)	2.7 (0.8, 4.6)	2.5 (0.7, 4.4)	2.4 (0.3, 4.5)	1.9 (0.1, 3.7)	2.2 (-0.1, 4.4)	2.0 (0.1, 3.9)	1.8 (0.0, 3.6)	1.7 (-0.1, 3.5)
Malaysia										
rural	141.7 (140.3, 143.1)	105.8 (103.7, 107.8)	132.9 (131.2, 134.4)	161.2 (159.6, 162.8)	165.0 (163.1, 166.8)	144.8 (143.6, 146.0)	108.7 (106.9, 110.6)	136.2 (134.9, 137.5)	164.4 (163.1, 165.6)	167.6 (166.4, 168.9)
urban	144.4 (143.0, 145.8)	108.6 (106.5, 110.6)	135.6 (134.1, 137.2)	163.9 (162.3, 165.5)	167.6 (165.8, 169.5)	146.7 (145.5, 147.9)	110.7 (108.8, 112.6)	138.1 (136.8, 139.4)	166.2 (165.0, 167.4)	169.5 (168.2, 170.7)
urban-rural difference	2.7 (1.8, 3.6)	2.8 (1.7, 3.9)	2.7 (1.8, 3.7)	2.7 (1.8, 3.6)	2.7 (1.7, 3.6)	1.9 (1.2, 2.5)	2.0 (1.1, 2.8)	1.9 (1.2, 2.6)	1.9 (1.2, 2.5)	1.8 (1.1, 2.6)
Maldives										
rural	141.0 (137.6, 144.4)	108.0 (99.6, 116.5)	132.6 (129.2, 136.0)	159.4 (156.1, 162.8)	162.2 (157.8, 166.5)	145.5 (142.1, 148.9)	110.5 (101.1, 120.0)	136.8 (133.0, 140.9)	164.8 (162.8, 166.7)	168.0 (166.1, 169.9)
urban	143.4 (140.1, 146.7)	110.8 (102.3, 119.2)	135.1 (131.7, 138.5)	161.7 (158.5, 164.8)	164.3 (160.1, 168.4)	147.3 (143.8, 150.8)	112.6 (102.9, 122.3)	138.7 (134.8, 142.7)	166.4 (164.3, 168.4)	169.5 (167.6, 171.4)
urban-rural difference	2.4 (0.8, 4.1)	2.7 (0.7, 4.7)	2.5 (0.8, 4.2)	2.3 (0.6, 3.9)	2.1 (0.3, 3.9)	1.8 (0.3, 3.2)	2.1 (0.0, 4.1)	1.8 (0.2, 3.5)	1.6 (0.3, 3.0)	1.5 (0.2, 2.8)
Myanmar										
rural	137.4 (133.7, 141.2)	102.5 (94.0, 111.1)	127.8 (123.8, 131.6)	156.6 (152.8, 160.3)	162.9 (158.1, 167.6)	141.5 (137.1, 145.8)	106.9 (96.7, 117.0)	132.1 (127.1, 137.1)	160.4 (157.3, 163.4)	165.9 (163.3, 168.5)
urban	139.5 (135.7, 143.2)	105.0 (96.4, 113.6)	130.0 (126.0, 133.8)	158.5 (154.7, 162.1)	164.5 (159.9, 169.2)	143.0 (138.6, 147.3)	108.8 (98.7, 119.0)	133.8 (128.7, 138.8)	161.7 (158.5, 164.8)	167.1 (164.4, 169.7)
urban-rural difference	2.1 (0.6, 3.6)	2.5 (0.6, 4.4)	2.2 (0.6, 3.8)	1.9 (0.4, 3.3)	1.6 (0.0, 3.3)	1.5 (0.0, 3.2)	1.9 (-0.2, 4.1)	1.7 (-0.1, 3.4)	1.4 (0.0, 2.8)	1.1 (-0.3, 2.5)
Philippines										
rural	136.3 (134.6, 138.1)	101.5 (99.7, 103.3)	126.6 (124.9, 128.3)	155.6 (153.4, 157.8)	161.7 (158.6, 165.0)	139.7 (138.4, 141.0)	104.8 (102.3, 107.3)	130.2 (128.8, 131.6)	159.0 (157.8, 160.2)	164.6 (163.3, 166.0)
urban	138.8 (137.1, 140.6)	104.3 (102.5, 106.2)	129.1 (127.5, 130.8)	157.9 (155.7, 160.0)	163.8 (160.6, 167.1)	141.8 (140.5, 143.1)	107.2 (104.7, 109.7)	132.3 (130.9, 133.8)	160.8 (159.6, 162.1)	166.3 (164.8, 167.7)
urban-rural difference	2.5 (1.4, 3.5)	2.8 (1.7, 4.0)	2.6 (1.5, 3.6)	2.3 (1.2, 3.3)	2.1 (0.9, 3.2)	2.0 (1.3, 2.8)	2.4 (1.5, 3.3)	2.1 (1.4, 2.9)	1.9 (1.1, 2.6)	1.6 (0.8, 2.5)
Thailand										
rural	140.9 (139.7, 142.2)	105.6 (104.0, 107.1)	131.5 (130.2, 132.8)	160.5 (159.2, 162.0)	165.1 (163.5, 166.7)	147.4 (146.0, 148.9)	111.6 (108.5, 114.8)	138.4 (136.7, 140.1)	167.1 (165.9, 168.3)	170.7 (169.3, 172.0)
urban	143.6 (142.3, 144.9)	108.6 (107.0, 110.1)	134.2 (132.9, 135.6)	163.1 (161.7, 164.6)	167.5 (165.8, 169.1)	149.2 (147.8, 150.7)	113.8 (110.7, 116.9)	140.3 (138.6, 142.0)	168.8 (167.6, 170.0)	172.2 (170.9, 173.6)
urban-rural difference	2.7 (1.9, 3.4)	3.0 (2.0, 3.9)	2.8 (2.0, 3.6)	2.5 (1.7, 3.3)	2.4 (1.4, 3.3)	1.8 (0.9, 2.8)	2.1 (0.9, 3.4)	1.9 (0.9, 2.9)	1.7 (0.8, 2.6)	1.5 (0.6, 2.5)
Timor-Leste										
rural	133.3 (130.0, 136.7)	99.7 (95.6, 104.0)	123.5 (120.3, 126.9)	152.0 (147.7, 156.3)	158.6 (152.7, 164.6)	136.4 (134.5, 138.2)	104.0 (101.2, 106.8)	127.1 (125.0, 129.2)	154.4 (152.6, 156.2)	160.0 (158.1, 162.0)
urban	136.5 (133.1, 140.1)	103.1 (98.7, 107.6)	126.8 (123.5, 130.3)	155.1 (150.8, 159.5)	161.6 (155.7, 167.6)	139.0 (136.9, 141.2)	106.8 (103.5, 110.1)	129.8 (127.3, 132.3)	157.0 (155.0, 159.0)	162.5 (160.3, 164.6)
urban-rural difference	3.2 (1.6, 4.8)	3.4 (1.5, 5.3)	3.3 (1.6, 4.9)	3.1 (1.5, 4.7)	3.0 (1.2, 4.8)	2.6 (1.3, 4.1)	2.9 (0.8, 4.9)	2.7 (1.2, 4.3)	2.5 (1.3, 3.9)	2.4 (1.2, 3.8)
Viet Nam										
rural	135.9 (134.8, 137.1)	101.9 (100.5, 103.3)	126.0 (124.8, 127.3)	154.4 (153.1, 155.7)	162.3 (160.6, 164.0)	143.2 (141.5, 144.9)	108.5 (104.7, 112.3)	133.8 (131.7, 136.0)	161.8 (160.5, 163.1)	168.5 (167.1, 169.9)

Boys	Mean height in 1990 (cm)					Mean height in 2020 (cm)				
	Age-standardised	5 years	10 years	15 years	19 years	Age-standardised	5 years	10 years	15 years	19 years
urban	139.2 (138.1, 140.4)	106.0 (104.6, 107.3)	129.6 (128.3, 130.8)	157.4 (156.1, 158.7)	164.9 (163.2, 166.6)	145.7 (144.0, 147.5)	111.8 (108.0, 115.6)	136.6 (134.5, 138.7)	164.0 (162.7, 165.3)	170.3 (168.9, 171.7)
urban-rural difference	3.3 (2.6, 4.0)	4.0 (3.2, 4.9)	3.5 (2.8, 4.2)	3.0 (2.3, 3.7)	2.5 (1.7, 3.3)	2.5 (1.7, 3.4)	3.3 (2.3, 4.3)	2.7 (1.9, 3.6)	2.2 (1.4, 3.0)	1.8 (0.9, 2.6)
**High-income Asia Pacific**										
Japan										
rural	146.8 (146.4, 147.2)	109.5 (109.0, 110.0)	138.1 (137.6, 138.5)	166.9 (166.4, 167.4)	170.1 (169.5, 170.6)	147.7 (147.2, 148.2)	110.0 (109.2, 110.8)	138.7 (138.2, 139.3)	168.1 (167.6, 168.6)	171.6 (171.0, 172.2)
urban	147.0 (146.7, 147.4)	109.6 (109.1, 110.1)	138.3 (137.8, 138.7)	167.2 (166.8, 167.7)	170.5 (170.0, 171.0)	148.0 (147.5, 148.5)	110.1 (109.3, 111.0)	139.0 (138.4, 139.5)	168.5 (168.0, 169.0)	172.1 (171.5, 172.7)
urban-rural difference	0.2 (0.0, 0.5)	0.1 (-0.2, 0.4)	0.2 (-0.1, 0.4)	0.3 (0.0, 0.6)	0.4 (0.1, 0.8)	0.3 (0.0, 0.6)	0.1 (-0.3, 0.5)	0.2 (-0.1, 0.5)	0.4 (0.1, 0.7)	0.5 (0.1, 0.9)
Singapore										
rural	147.7 (143.0, 152.5)	109.6 (99.1, 119.8)	138.8 (133.6, 143.9)	168.2 (163.5, 172.8)	171.8 (166.5, 177.1)	149.5 (144.2, 154.5)	112.3 (100.8, 123.8)	140.7 (134.7, 146.4)	169.5 (165.3, 173.6)	172.8 (169.7, 175.9)
urban	148.7 (144.3, 153.1)	110.7 (100.4, 120.9)	139.8 (135.0, 144.8)	169.1 (164.8, 173.3)	172.7 (167.8, 177.5)	150.1 (145.2, 154.9)	113.1 (101.7, 124.2)	141.4 (135.7, 146.9)	170.2 (166.4, 174.0)	173.4 (171.0, 175.8)
urban-rural difference	1.0 (-1.1, 3.1)	1.1 (-1.2, 3.3)	1.0 (-1.1, 3.1)	0.9 (-1.2, 3.1)	0.9 (-1.4, 3.3)	0.7 (-1.4, 2.7)	0.7 (-1.8, 3.2)	0.7 (-1.5, 2.8)	0.6 (-1.4, 2.6)	0.6 (-1.4, 2.6)
South Korea										
rural	147.5 (146.5, 148.5)	110.1 (108.9, 111.3)	139.7 (138.6, 140.8)	167.5 (166.3, 168.6)	168.6 (167.1, 170.1)	151.6 (151.0, 152.3)	112.6 (111.5, 113.8)	142.9 (142.2, 143.7)	172.4 (171.7, 173.1)	175.2 (174.5, 176.0)
urban	148.2 (147.2, 149.1)	111.0 (110.0, 112.1)	140.4 (139.4, 141.5)	168.0 (166.9, 169.2)	169.0 (167.5, 170.4)	151.9 (151.3, 152.5)	113.2 (112.0, 114.3)	143.3 (142.6, 144.0)	172.6 (171.9, 173.2)	175.3 (174.6, 176.0)
urban-rural difference	0.7 (-0.1, 1.5)	0.9 (0.0, 1.8)	0.8 (0.0, 1.6)	0.6 (-0.2, 1.4)	0.5 (-0.4, 1.3)	0.3 (-0.2, 0.7)	0.5 (-0.1, 1.1)	0.3 (-0.2, 0.8)	0.2 (-0.3, 0.7)	0.0 (-0.6, 0.6)
**High-income western**										
High-income English-speaking countries										
Australia										
rural	151.5 (150.3, 152.7)	112.8 (111.3, 114.2)	142.0 (140.7, 143.3)	172.7 (171.3, 174.1)	177.2 (175.2, 179.2)	154.3 (153.1, 155.5)	116.1 (113.3, 119.0)	144.9 (143.5, 146.4)	175.2 (174.2, 176.2)	179.5 (178.5, 180.6)
urban	151.3 (150.2, 152.4)	112.7 (111.3, 114.1)	141.8 (140.7, 143.0)	172.4 (171.1, 173.7)	176.8 (174.9, 178.7)	153.7 (152.5, 154.9)	115.7 (112.9, 118.6)	144.4 (142.9, 145.8)	174.6 (173.6, 175.5)	178.8 (177.7, 179.9)
urban-rural difference	-0.2 (-1.1, 0.7)	0.0 (-1.0, 0.9)	-0.2 (-1.0, 0.7)	-0.3 (-1.2, 0.7)	-0.4 (-1.5, 0.8)	-0.6 (-1.2, 0.0)	-0.4 (-1.3, 0.4)	-0.5 (-1.2, 0.1)	-0.6 (-1.3, 0.0)	-0.7 (-1.5, 0.0)
Canada										
rural	151.1 (149.3, 152.8)	111.5 (108.6, 114.2)	141.9 (139.8, 144.0)	172.3 (170.3, 174.1)	176.7 (174.4, 179.0)	152.9 (151.1, 154.6)	113.1 (110.5, 115.7)	143.6 (141.7, 145.6)	174.3 (172.6, 176.0)	178.8 (177.0, 180.7)
urban	151.5 (150.1, 152.9)	112.1 (109.6, 114.6)	142.4 (140.7, 144.2)	172.6 (171.0, 174.2)	177.0 (174.9, 179.0)	153.3 (152.1, 154.5)	113.7 (111.8, 115.7)	144.0 (142.7, 145.4)	174.6 (173.3, 175.8)	178.9 (177.5, 180.4)
urban-rural difference	0.5 (-1.1, 2.0)	0.7 (-1.2, 2.6)	0.5 (-1.0, 2.1)	0.4 (-1.2, 1.9)	0.2 (-1.5, 1.9)	0.3 (-1.3, 2.0)	0.6 (-1.7, 2.8)	0.4 (-1.4, 2.2)	0.3 (-1.3, 1.9)	0.1 (-1.4, 1.7)
Ireland										
rural	151.6 (149.6, 153.6)	113.3 (111.3, 115.4)	142.1 (140.1, 144.1)	172.5 (169.9, 175.1)	177.8 (174.4, 181.3)	152.8 (151.9, 153.8)	114.7 (113.0, 116.5)	143.3 (142.2, 144.5)	173.6 (172.5, 174.8)	178.8 (177.3, 180.4)
urban	151.4 (149.5, 153.4)	113.3 (111.2, 115.3)	141.9 (139.9, 143.9)	172.2 (169.7, 174.8)	177.4 (174.1, 180.9)	152.5 (151.6, 153.4)	114.5 (112.8, 116.3)	143.0 (141.9, 144.1)	173.2 (172.1, 174.4)	178.3 (176.8, 179.9)
urban-rural difference	-0.2 (-1.4, 0.9)	-0.1 (-1.2, 1.1)	-0.2 (-1.3, 0.9)	-0.3 (-1.6, 0.9)	-0.4 (-1.9, 1.0)	-0.3 (-1.0, 0.3)	-0.2 (-1.0, 0.7)	-0.3 (-0.9, 0.4)	-0.4 (-1.1, 0.4)	-0.5 (-1.4, 0.5)
New Zealand										
rural	152.4 (151.0, 153.8)	113.0 (111.1, 114.8)	143.0 (141.5, 144.5)	173.6 (172.1, 175.2)	178.2 (176.3, 180.2)	153.1 (152.2, 154.0)	113.5 (112.1, 114.8)	143.9 (142.9, 144.9)	174.5 (173.6, 175.5)	178.6 (177.4, 179.7)
urban	152.4 (151.2, 153.7)	113.5 (111.7, 115.2)	143.2 (141.7, 144.6)	173.5 (172.1, 174.9)	177.9 (176.1, 179.7)	152.9 (152.1, 153.7)	113.7 (112.5, 114.9)	143.8 (142.9, 144.7)	174.2 (173.3, 175.0)	177.9 (176.9, 179.0)
urban-rural difference	0.1 (-0.9, 1.0)	0.5 (-0.6, 1.6)	0.2 (-0.8, 1.2)	-0.1 (-1.1, 0.9)	-0.3 (-1.4, 0.7)	-0.2 (-0.8, 0.4)	0.2 (-0.6, 1.0)	-0.1 (-0.7, 0.5)	-0.4 (-1.0, 0.2)	-0.6 (-1.3, 0.1)
United Kingdom										
rural	150.9 (150.2, 151.5)	111.9 (111.1, 112.7)	141.5 (140.7, 142.3)	171.6 (170.9, 172.4)	177.4 (176.5, 178.4)	151.8 (151.2, 152.5)	111.8 (110.5, 113.0)	142.3 (141.6, 143.0)	173.1 (172.5, 173.8)	179.1 (178.4, 179.8)
urban	150.5 (149.8, 151.1)	111.8 (111.1, 112.6)	141.2 (140.4, 141.9)	171.1 (170.3, 171.8)	176.7 (175.8, 177.6)	151.3 (150.7, 151.8)	111.6 (110.4, 112.8)	141.8 (141.1, 142.4)	172.4 (171.8, 173.0)	178.1 (177.4, 178.8)
urban-rural difference	-0.4 (-0.9, 0.1)	0.0 (-0.6, 0.5)	-0.3 (-0.8, 0.2)	-0.6 (-1.0, -0.1)	-0.8 (-1.3, -0.3)	-0.6 (-1.0, -0.2)	-0.2 (-0.7, 0.2)	-0.5 (-0.9, -0.1)	-0.8 (-1.2, -0.3)	-1.0 (-1.5, -0.4)
United States of America										
rural	151.7 (150.8, 152.5)	113.9 (112.9, 114.8)	142.5 (141.6, 143.4)	172.6 (171.7, 173.6)	176.0 (174.8, 177.2)	151.8 (150.9, 152.6)	113.6 (111.9, 115.4)	142.7 (141.7, 143.6)	172.9 (172.0, 173.7)	175.9 (174.9, 177.0)
urban	151.8 (150.9, 152.7)	114.3 (113.3, 115.3)	142.7 (141.7, 143.6)	172.7 (171.6, 173.7)	175.9 (174.6, 177.2)	151.7 (150.9, 152.5)	113.8 (112.1, 115.5)	142.7 (141.8, 143.6)	172.7 (171.9, 173.5)	175.6 (174.7, 176.5)
urban-rural difference	0.2 (-0.5, 0.8)	0.4 (-0.4, 1.1)	0.2 (-0.4, 0.9)	0.0 (-0.7, 0.8)	-0.1 (-1.0, 0.9)	-0.1 (-0.7, 0.5)	0.1 (-0.5, 0.8)	0.0 (-0.6, 0.6)	-0.2 (-0.9, 0.5)	-0.3 (-1.2, 0.5)
Northwestern Europe										
Austria										
rural	153.8 (152.6, 155.1)	117.1 (115.0, 119.1)	145.2 (143.7, 146.7)	173.7 (172.4, 174.9)	177.3 (175.7, 178.9)	153.9 (152.8, 155.1)	116.2 (113.8, 118.6)	144.9 (143.6, 146.3)	174.3 (173.1, 175.5)	178.6 (177.4, 179.9)
urban	153.9 (152.8, 154.9)	117.1 (115.1, 119.0)	145.2 (143.9, 146.6)	173.7 (172.6, 174.8)	177.3 (176.0, 178.7)	153.7 (152.6, 154.8)	115.9 (113.5, 118.4)	144.7 (143.4, 146.0)	174.0 (172.8, 175.1)	178.3 (177.2, 179.5)
urban-rural difference	0.0 (-1.3, 1.4)	0.0 (-1.3, 1.4)	0.0 (-1.2, 1.4)	0.0 (-1.3, 1.5)	0.0 (-1.5, 1.7)	-0.3 (-1.2, 0.6)	-0.3 (-1.4, 0.8)	-0.3 (-1.2, 0.6)	-0.3 (-1.2, 0.6)	-0.3 (-1.3, 0.7)
Belgium										
rural	152.8 (150.8, 154.8)	115.9 (113.1, 118.7)	144.1 (142.0, 146.3)	172.5 (170.3, 174.9)	176.4 (174.0, 178.9)	153.1 (150.8, 155.3)	114.4 (110.6, 118.0)	143.5 (140.9, 146.1)	173.8 (171.7, 176.0)	179.5 (177.3, 181.7)
urban	152.4 (150.1, 154.6)	115.7 (112.9, 118.4)	143.8 (141.4, 146.2)	172.1 (169.4, 174.8)	175.9 (173.0, 178.8)	152.6 (150.8, 154.3)	114.0 (110.9, 117.1)	143.0 (140.8, 145.1)	173.3 (171.5, 175.0)	178.9 (177.1, 180.6)
urban-rural difference	-0.4 (-2.1, 1.3)	-0.2 (-2.1, 1.7)	-0.3 (-2.0, 1.4)	-0.4 (-2.2, 1.3)	-0.5 (-2.5, 1.5)	-0.5 (-2.2, 1.1)	-0.4 (-2.6, 1.7)	-0.5 (-2.3, 1.2)	-0.6 (-2.2, 1.0)	-0.7 (-2.3, 0.9)
Denmark										

Boys	Mean height in 1990 (cm)					Mean height in 2020 (cm)				
	Age-standardised	5 years	10 years	15 years	19 years	Age-standardised	5 years	10 years	15 years	19 years
rural	152.4 (151.2, 153.7)	115.6 (114.1, 117.2)	142.6 (141.3, 143.9)	172.6 (171.2, 174.0)	178.8 (177.1, 180.4)	154.9 (153.8, 156.0)	117.2 (114.8, 119.6)	144.7 (143.3, 146.1)	175.5 (174.5, 176.6)	182.3 (181.3, 183.3)
urban	152.6 (151.8, 153.3)	116.0 (115.1, 117.0)	142.8 (141.9, 143.7)	172.6 (171.6, 173.5)	178.6 (177.3, 179.9)	155.1 (154.1, 156.2)	117.7 (115.4, 120.1)	145.0 (143.6, 146.3)	175.6 (174.6, 176.6)	182.2 (181.3, 183.1)
urban-rural difference	0.1 (-1.0, 1.1)	0.4 (-0.9, 1.7)	0.2 (-0.9, 1.3)	0.0 (-1.1, 1.0)	-0.2 (-1.3, 0.8)	0.2 (-0.5, 1.0)	0.5 (-0.6, 1.7)	0.3 (-0.5, 1.2)	0.1 (-0.5, 0.8)	-0.1 (-0.7, 0.6)
Finland										
rural	152.4 (151.3, 153.4)	113.5 (111.4, 115.5)	143.4 (142.0, 144.8)	173.0 (171.7, 174.3)	177.5 (176.0, 179.0)	153.0 (151.6, 154.4)	112.6 (110.7, 114.5)	143.5 (142.0, 145.0)	174.3 (172.9, 175.7)	179.9 (178.4, 181.6)
urban	153.1 (152.1, 154.2)	114.1 (112.0, 116.1)	144.1 (142.7, 145.4)	173.8 (172.6, 175.1)	178.4 (177.0, 179.8)	153.3 (152.1, 154.5)	112.8 (111.0, 114.5)	143.8 (142.5, 145.1)	174.7 (173.4, 175.9)	180.4 (178.9, 181.9)
urban-rural difference	0.7 (-0.1, 1.6)	0.6 (-0.4, 1.6)	0.7 (-0.1, 1.5)	0.8 (-0.1, 1.7)	0.9 (-0.1, 1.9)	0.3 (-0.7, 1.3)	0.2 (-1.1, 1.4)	0.3 (-0.8, 1.3)	0.4 (-0.6, 1.4)	0.5 (-0.6, 1.5)
Germany										
rural	153.3 (151.7, 154.8)	114.0 (111.6, 116.4)	143.7 (141.8, 145.6)	174.4 (172.6, 176.1)	179.6 (178.0, 181.3)	154.0 (152.3, 155.7)	114.7 (112.0, 117.4)	144.4 (142.5, 146.2)	175.1 (173.3, 176.9)	180.4 (178.7, 182.3)
urban	153.4 (151.9, 154.9)	114.4 (112.1, 116.8)	143.9 (142.0, 145.7)	174.4 (172.8, 176.0)	179.5 (178.1, 180.9)	154.1 (152.4, 155.7)	115.1 (112.5, 117.7)	144.5 (142.6, 146.3)	175.0 (173.3, 176.8)	180.2 (178.5, 181.8)
urban-rural difference	0.1 (-0.9, 1.2)	0.4 (-0.8, 1.7)	0.2 (-0.9, 1.3)	0.0 (-1.1, 1.1)	-0.2 (-1.4, 1.1)	0.0 (-0.9, 1.0)	0.4 (-0.8, 1.6)	0.1 (-0.8, 1.1)	-0.1 (-1.0, 0.8)	-0.3 (-1.3, 0.8)
Greenland										
rural	147.4 (143.1, 151.8)	109.3 (100.5, 118.1)	138.0 (133.5, 142.6)	168.0 (163.4, 172.4)	172.7 (167.2, 178.1)	148.6 (143.8, 153.4)	111.1 (101.2, 121.5)	139.4 (133.9, 144.8)	169.0 (164.9, 173.2)	173.7 (170.4, 177.0)
urban	148.0 (143.8, 152.5)	109.9 (101.3, 118.9)	138.6 (134.2, 143.2)	168.6 (164.3, 173.1)	173.4 (168.0, 178.9)	149.0 (144.3, 153.8)	111.3 (101.4, 121.9)	139.7 (134.3, 145.2)	169.4 (165.4, 173.5)	174.0 (170.9, 177.3)
urban-rural difference	0.7 (-1.0, 2.4)	0.6 (-1.4, 2.6)	0.6 (-1.1, 2.4)	0.7 (-1.0, 2.4)	0.7 (-1.1, 2.6)	0.3 (-1.5, 2.1)	0.2 (-2.1, 2.5)	0.3 (-1.6, 2.2)	0.3 (-1.3, 2.0)	0.4 (-1.2, 2.0)
Iceland										
rural	154.1 (149.4, 158.8)	116.2 (107.2, 125.0)	144.6 (139.7, 149.4)	174.6 (169.7, 179.6)	180.0 (174.0, 186.3)	154.8 (149.7, 159.8)	116.6 (106.3, 126.5)	145.1 (139.4, 150.8)	175.5 (170.9, 180.0)	181.4 (177.1, 185.5)
urban	154.4 (149.9, 159.0)	116.5 (107.7, 125.3)	144.9 (140.2, 149.6)	174.9 (170.2, 179.6)	180.3 (174.6, 186.2)	154.9 (149.9, 159.8)	116.7 (106.3, 126.5)	145.2 (139.6, 150.7)	175.5 (171.1, 179.9)	181.4 (177.3, 185.3)
urban-rural difference	0.3 (-1.6, 2.3)	0.4 (-1.8, 2.5)	0.3 (-1.6, 2.3)	0.3 (-1.6, 2.3)	0.3 (-1.8, 2.5)	0.1 (-1.9, 2.0)	0.1 (-2.3, 2.4)	0.1 (-2.0, 2.0)	0.1 (-1.8, 1.8)	0.1 (-1.8, 1.9)
Luxembourg										
rural	151.9 (147.6, 156.1)	113.4 (104.7, 122.0)	142.2 (137.7, 146.6)	172.5 (168.1, 177.0)	178.3 (173.1, 183.8)	152.5 (147.8, 157.1)	114.9 (104.8, 125.1)	143.0 (137.6, 148.2)	172.9 (169.0, 176.7)	178.5 (175.7, 181.3)
urban	151.6 (147.4, 155.9)	113.3 (104.6, 121.8)	142.0 (137.6, 146.3)	172.3 (167.9, 176.8)	178.0 (172.8, 183.4)	152.0 (147.3, 156.6)	114.5 (104.4, 124.6)	142.5 (137.1, 147.8)	172.3 (168.4, 176.2)	177.9 (175.0, 180.7)
urban-rural difference	-0.2 (-1.8, 1.3)	-0.1 (-2.1, 1.8)	-0.2 (-1.8, 1.4)	-0.3 (-1.8, 1.3)	-0.3 (-2.0, 1.4)	-0.5 (-2.2, 1.2)	-0.4 (-2.6, 1.9)	-0.5 (-2.3, 1.4)	-0.5 (-2.1, 1.0)	-0.6 (-2.1, 0.9)
Netherlands										
rural	156.1 (153.8, 158.5)	119.4 (116.2, 122.7)	146.4 (143.9, 148.9)	176.2 (173.3, 179.0)	181.9 (178.6, 185.2)	156.6 (154.4, 158.7)	119.4 (115.1, 123.7)	146.5 (143.8, 149.2)	176.9 (174.9, 179.0)	183.6 (181.0, 186.1)
urban	156.1 (153.9, 158.3)	119.3 (116.1, 122.6)	146.4 (144.0, 148.8)	176.2 (173.4, 179.0)	181.9 (178.8, 185.1)	156.3 (154.2, 158.5)	119.1 (114.6, 123.5)	146.2 (143.5, 149.0)	176.7 (174.8, 178.7)	183.4 (181.0, 185.7)
urban-rural difference	0.0 (-1.3, 1.3)	-0.1 (-1.5, 1.3)	-0.1 (-1.3, 1.3)	0.0 (-1.4, 1.4)	0.0 (-1.6, 1.7)	-0.2 (-1.4, 0.9)	-0.3 (-1.8, 1.2)	-0.3 (-1.5, 1.0)	-0.2 (-1.3, 1.0)	-0.2 (-1.5, 1.1)
Norway										
rural	154.2 (152.2, 156.2)	115.1 (110.9, 119.4)	144.5 (142.3, 146.7)	175.3 (173.1, 177.6)	180.4 (178.0, 182.9)	154.7 (152.6, 156.7)	116.7 (112.2, 121.5)	145.3 (142.8, 147.7)	175.3 (173.1, 177.5)	180.0 (177.5, 182.4)
urban	154.6 (152.5, 156.7)	115.4 (111.4, 119.7)	144.9 (142.7, 147.1)	175.8 (173.4, 178.3)	181.0 (178.3, 183.7)	154.8 (153.0, 156.5)	116.7 (112.3, 121.3)	145.3 (143.1, 147.7)	175.4 (173.4, 177.5)	180.2 (177.8, 182.7)
urban-rural difference	0.4 (-0.9, 1.7)	0.3 (-1.2, 1.9)	0.4 (-0.9, 1.7)	0.5 (-0.9, 1.8)	0.5 (-1.0, 2.1)	0.1 (-1.3, 1.5)	0.0 (-1.8, 1.8)	0.1 (-1.4, 1.6)	0.2 (-1.1, 1.5)	0.2 (-1.1, 1.6)
Sweden										
rural	153.2 (152.5, 154.0)	114.9 (113.1, 116.7)	143.3 (142.3, 144.4)	174.4 (173.6, 175.2)	179.3 (178.2, 180.5)	154.0 (152.6, 155.5)	116.2 (113.4, 119.0)	144.1 (142.4, 145.8)	175.0 (173.5, 176.4)	180.2 (178.6, 181.7)
urban	153.1 (152.6, 153.5)	114.4 (113.0, 115.7)	143.1 (142.3, 143.8)	174.4 (173.7, 175.0)	179.5 (178.4, 180.6)	153.6 (152.5, 154.7)	115.4 (113.0, 117.8)	143.6 (142.2, 145.0)	174.7 (173.4, 175.9)	180.1 (178.6, 181.5)
urban-rural difference	-0.2 (-0.9, 0.6)	-0.5 (-1.9, 0.8)	-0.3 (-1.2, 0.6)	0.0 (-0.6, 0.6)	0.1 (-0.5, 0.8)	-0.4 (-1.6, 0.7)	-0.7 (-2.3, 0.8)	-0.5 (-1.7, 0.7)	-0.3 (-1.3, 0.7)	-0.1 (-1.2, 0.9)
Switzerland										
rural	151.0 (149.1, 152.9)	113.8 (111.9, 115.7)	141.7 (139.8, 143.6)	171.1 (168.7, 173.5)	176.1 (172.9, 179.5)	151.7 (150.5, 153.0)	113.1 (111.0, 115.4)	141.9 (140.5, 143.5)	172.5 (171.2, 173.8)	178.5 (177.2, 179.7)
urban	151.6 (149.8, 153.3)	114.3 (112.4, 116.1)	142.3 (140.5, 144.1)	171.7 (169.4, 174.0)	176.8 (173.7, 180.0)	152.1 (150.9, 153.3)	113.4 (111.4, 115.6)	142.3 (140.9, 143.7)	172.9 (171.6, 174.2)	178.9 (177.7, 180.2)
urban-rural difference	0.6 (-0.5, 1.6)	0.5 (-0.6, 1.6)	0.6 (-0.5, 1.6)	0.6 (-0.5, 1.7)	0.6 (-0.6, 1.9)	0.4 (-0.3, 1.1)	0.3 (-0.6, 1.3)	0.4 (-0.3, 1.1)	0.4 (-0.2, 1.1)	0.5 (-0.2, 1.2)
Southwestern Europe										
Andorra										
rural	149.8 (143.0, 156.7)	113.3 (103.2, 123.5)	140.6 (134.0, 147.6)	169.9 (163.2, 177.0)	174.2 (165.8, 182.7)	151.4 (144.8, 158.2)	115.1 (104.0, 126.2)	142.3 (135.4, 149.3)	171.5 (165.3, 177.9)	176.0 (168.9, 183.4)
urban	150.5 (143.9, 157.4)	113.9 (103.8, 124.1)	141.3 (134.9, 148.0)	170.7 (164.0, 177.6)	175.1 (166.9, 183.5)	151.4 (144.8, 158.1)	114.9 (103.9, 126.1)	142.2 (135.4, 149.1)	171.5 (165.2, 177.8)	176.1 (168.9, 183.4)
urban-rural difference	0.7 (-1.2, 2.7)	0.6 (-1.6, 2.7)	0.7 (-1.3, 2.7)	0.8 (-1.2, 2.8)	0.9 (-1.3, 3.1)	-0.1 (-2.0, 1.9)	-0.2 (-2.5, 2.2)	-0.1 (-2.1, 1.9)	0.0 (-1.9, 1.8)	0.1 (-1.8, 1.9)
Cyprus										
rural	149.4 (146.3, 152.6)	113.3 (109.7, 116.8)	140.4 (137.3, 143.4)	169.5 (165.5, 173.6)	173.2 (167.2, 179.2)	151.2 (149.4, 153.0)	116.0 (113.3, 118.8)	142.3 (140.3, 144.3)	170.8 (168.7, 173.0)	174.3 (171.3, 177.6)
urban	150.1 (146.9, 153.2)	113.8 (110.3, 117.3)	141.0 (137.9, 144.0)	170.3 (166.3, 174.3)	174.0 (168.2, 179.9)	151.0 (149.4, 152.6)	115.7 (113.1, 118.3)	142.1 (140.3, 143.9)	170.7 (168.8, 172.7)	174.3 (171.3, 177.4)
urban-rural difference	0.7 (-0.8, 2.2)	0.6 (-1.0, 2.2)	0.7 (-0.9, 2.2)	0.7 (-0.9, 2.3)	0.8 (-1.1, 2.6)	-0.2 (-1.3, 1.0)	-0.3 (-1.8, 1.2)	-0.2 (-1.4, 1.0)	-0.1 (-1.3, 1.1)	0.0 (-1.4, 1.3)
France										
rural	150.1 (147.7, 152.4)	112.6 (110.1, 115.1)	140.9 (138.7, 143.1)	170.6 (167.4, 173.6)	174.6 (170.2, 179.0)	152.1 (150.5, 153.7)	114.6 (111.4, 117.9)	142.9 (141.0, 144.7)	172.6 (171.0, 174.1)	177.2 (175.5, 178.8)
urban	151.1 (148.8, 153.4)	113.0 (110.7, 115.4)	141.8 (139.7, 143.9)	172.0 (168.8, 175.0)	176.4 (171.9, 180.7)	151.9 (150.4, 153.5)	113.8 (110.6, 117.1)	142.5 (140.7, 144.3)	172.8 (171.3, 174.2)	177.7 (176.2, 179.2)

Boys	Mean height in 1990 (cm)					Mean height in 2020 (cm)				
	Age-standardised	5 years	10 years	15 years	19 years	Age-standardised	5 years	10 years	15 years	19 years
urban-rural difference	1.1 (-0.1, 2.3)	0.4 (-1.0, 1.7)	0.9 (-0.3, 2.1)	1.4 (0.1, 2.7)	1.7 (0.3, 3.3)	-0.2 (-1.1, 0.8)	-0.8 (-2.1, 0.4)	-0.4 (-1.4, 0.6)	0.1 (-0.8, 1.1)	0.5 (-0.6, 1.7)
Greece										
rural	152.4 (150.5, 154.4)	115.6 (113.8, 117.5)	143.0 (141.1, 144.9)	172.5 (170.0, 175.2)	178.1 (174.6, 181.6)	154.4 (153.2, 155.5)	118.7 (116.5, 121.0)	145.1 (143.7, 146.5)	174.0 (172.7, 175.3)	179.4 (177.8, 180.9)
urban	153.6 (151.6, 155.6)	116.4 (114.5, 118.2)	144.0 (142.2, 145.9)	173.8 (171.2, 176.5)	179.7 (176.1, 183.3)	154.3 (153.2, 155.4)	118.2 (116.0, 120.6)	144.9 (143.5, 146.2)	174.1 (172.9, 175.4)	179.8 (178.3, 181.2)
urban-rural difference	1.2 (0.3, 2.2)	0.7 (-0.3, 1.8)	1.0 (0.2, 2.0)	1.4 (0.4, 2.4)	1.6 (0.4, 2.9)	-0.1 (-0.8, 0.6)	-0.5 (-1.5, 0.5)	-0.2 (-1.0, 0.6)	0.1 (-0.7, 0.9)	0.4 (-0.6, 1.3)
Israel										
rural	150.7 (149.1, 152.4)	112.9 (107.8, 118.1)	141.9 (139.9, 143.9)	171.5 (170.2, 172.8)	174.3 (172.7, 175.9)	150.9 (149.4, 152.5)	113.3 (109.5, 117.0)	142.0 (139.9, 144.1)	171.7 (170.1, 173.2)	174.7 (173.4, 176.1)
urban	151.0 (149.4, 152.6)	113.2 (108.3, 118.2)	142.2 (140.3, 144.0)	171.8 (170.5, 173.0)	174.6 (173.0, 176.1)	150.6 (149.1, 152.0)	112.9 (109.6, 116.4)	141.6 (139.8, 143.5)	171.3 (169.8, 172.8)	174.3 (173.1, 175.6)
urban-rural difference	0.3 (-0.7, 1.2)	0.3 (-1.2, 1.9)	0.3 (-0.8, 1.4)	0.3 (-0.6, 1.1)	0.3 (-0.7, 1.1)	-0.4 (-1.4, 0.7)	-0.3 (-2.0, 1.4)	-0.3 (-1.5, 0.9)	-0.4 (-1.2, 0.5)	-0.4 (-1.2, 0.4)
Italy										
rural	149.5 (148.2, 150.9)	113.6 (111.7, 115.5)	140.1 (138.6, 141.5)	169.5 (168.0, 171.1)	174.4 (172.6, 176.2)	151.6 (150.4, 152.9)	116.8 (113.8, 119.9)	142.7 (141.2, 144.1)	171.0 (169.7, 172.4)	174.9 (173.2, 176.7)
urban	150.6 (149.3, 152.0)	114.6 (112.7, 116.6)	141.2 (139.7, 142.6)	170.7 (169.2, 172.2)	175.6 (173.9, 177.3)	151.9 (150.7, 153.2)	117.1 (114.0, 120.1)	143.0 (141.6, 144.5)	171.4 (170.1, 172.8)	175.4 (173.8, 176.9)
urban-rural difference	1.1 (0.0, 2.3)	1.0 (-0.1, 2.2)	1.1 (0.0, 2.2)	1.2 (-0.1, 2.4)	1.2 (-0.3, 2.7)	0.3 (-0.5, 1.1)	0.3 (-0.7, 1.3)	0.3 (-0.5, 1.1)	0.4 (-0.5, 1.2)	0.4 (-0.7, 1.5)
Malta										
rural	147.7 (144.4, 151.1)	112.6 (108.8, 116.3)	138.2 (134.7, 141.6)	167.6 (163.2, 172.0)	172.3 (166.6, 178.2)	149.7 (147.8, 151.6)	113.8 (110.3, 117.4)	139.9 (137.7, 142.0)	169.9 (167.1, 172.5)	175.3 (172.2, 178.3)
urban	148.6 (145.3, 151.8)	113.2 (109.6, 116.8)	139.0 (135.7, 142.2)	168.6 (164.2, 172.9)	173.4 (167.7, 179.3)	149.8 (148.0, 151.4)	113.7 (110.3, 117.0)	139.9 (137.9, 141.9)	170.0 (167.5, 172.4)	175.6 (172.9, 178.4)
urban-rural difference	0.9 (-0.8, 2.5)	0.6 (-1.1, 2.3)	0.8 (-0.8, 2.4)	1.0 (-0.8, 2.8)	1.1 (-0.9, 3.1)	0.1 (-1.3, 1.4)	-0.2 (-1.8, 1.4)	0.0 (-1.4, 1.3)	0.2 (-1.2, 1.5)	0.3 (-1.2, 1.9)
Portugal										
rural	147.3 (146.2, 148.4)	113.1 (111.2, 115.1)	138.4 (136.9, 139.9)	166.4 (165.3, 167.5)	170.8 (169.8, 171.9)	149.8 (148.7, 151.0)	113.9 (111.8, 115.9)	140.4 (139.0, 141.8)	169.6 (168.2, 170.9)	175.4 (173.7, 177.0)
urban	148.2 (147.0, 149.3)	113.9 (111.9, 115.9)	139.2 (137.7, 140.7)	167.2 (166.1, 168.4)	171.7 (170.7, 172.7)	149.8 (148.6, 150.9)	113.7 (111.7, 115.7)	140.3 (139.0, 141.7)	169.5 (168.2, 170.9)	175.3 (173.7, 177.0)
urban-rural difference	0.8 (0.2, 1.5)	0.8 (-0.2, 1.8)	0.8 (0.1, 1.5)	0.8 (0.3, 1.4)	0.9 (0.3, 1.4)	-0.1 (-0.8, 0.6)	-0.1 (-0.9, 0.7)	-0.1 (-0.8, 0.6)	-0.1 (-0.8, 0.7)	0.0 (-0.9, 0.9)
Spain										
rural	150.7 (149.1, 152.3)	114.1 (112.1, 116.3)	142.2 (140.6, 144.0)	170.3 (168.4, 172.2)	174.2 (171.8, 176.7)	151.6 (150.6, 152.6)	115.2 (113.3, 117.1)	143.0 (141.9, 144.1)	171.1 (170.0, 172.2)	175.5 (174.0, 176.9)
urban	151.5 (150.0, 153.0)	114.7 (112.8, 116.7)	143.0 (141.3, 144.6)	171.2 (169.4, 173.0)	175.2 (172.9, 177.5)	151.8 (150.8, 152.7)	115.2 (113.4, 117.1)	143.2 (142.1, 144.2)	171.4 (170.3, 172.5)	175.9 (174.4, 177.3)
urban-rural difference	0.8 (-0.2, 1.8)	0.6 (-0.6, 1.7)	0.7 (-0.3, 1.7)	0.9 (-0.1, 1.9)	1.0 (-0.1, 2.1)	0.2 (-0.4, 0.9)	0.0 (-0.9, 0.8)	0.1 (-0.5, 0.8)	0.3 (-0.4, 1.0)	0.4 (-0.4, 1.3)
**Latin America and Caribbean**										
Andean Latin America										
Bolivia										
rural	140.7 (136.2, 145.2)	105.6 (96.9, 114.6)	132.5 (128.2, 136.7)	159.6 (154.6, 164.5)	163.0 (156.5, 169.6)	143.5 (139.4, 147.8)	108.7 (98.3, 119.6)	135.4 (130.6, 140.2)	162.3 (159.4, 165.2)	165.5 (163.3, 167.7)
urban	144.1 (139.6, 148.5)	109.2 (100.5, 118.2)	135.9 (131.7, 140.1)	162.8 (157.9, 167.8)	166.1 (159.7, 172.6)	146.6 (142.4, 150.7)	112.0 (101.6, 122.5)	138.5 (133.7, 143.3)	165.3 (162.4, 168.1)	168.4 (166.2, 170.5)
urban-rural difference	3.4 (1.6, 5.1)	3.6 (1.6, 5.6)	3.4 (1.6, 5.2)	3.3 (1.4, 5.1)	3.1 (1.0, 5.2)	3.1 (1.5, 4.7)	3.3 (1.1, 5.6)	3.1 (1.4, 4.9)	3.0 (1.6, 4.4)	2.9 (1.5, 4.2)
Ecuador										
rural	139.7 (136.9, 142.7)	104.0 (100.7, 107.5)	131.1 (128.3, 134.0)	158.8 (155.3, 162.3)	163.2 (158.4, 168.1)	142.4 (141.0, 143.8)	107.2 (105.2, 109.2)	133.9 (132.3, 135.6)	161.2 (159.7, 162.7)	165.4 (163.9, 167.1)
urban	142.7 (139.9, 145.4)	106.9 (103.6, 110.2)	134.1 (131.3, 136.8)	161.8 (158.4, 165.2)	166.2 (161.6, 171.0)	144.9 (143.5, 146.3)	109.7 (107.7, 111.7)	136.4 (134.8, 138.0)	163.7 (162.3, 165.1)	167.9 (166.4, 169.5)
urban-rural difference	3.0 (1.2, 4.6)	2.9 (1.2, 4.7)	3.0 (1.2, 4.6)	3.0 (1.2, 4.7)	3.0 (1.1, 4.9)	2.5 (1.7, 3.3)	2.4 (1.3, 3.6)	2.5 (1.6, 3.4)	2.5 (1.7, 3.3)	2.5 (1.6, 3.4)
Peru										
rural	138.0 (136.1, 140.1)	102.7 (100.4, 104.8)	129.1 (127.2, 131.1)	157.0 (154.6, 159.5)	162.4 (158.7, 166.3)	141.2 (139.6, 142.8)	106.9 (103.2, 110.6)	132.7 (130.5, 134.8)	159.7 (158.6, 160.7)	164.3 (163.2, 165.3)
urban	142.4 (140.5, 144.2)	107.8 (105.6, 109.9)	133.7 (131.9, 135.4)	161.0 (158.7, 163.3)	166.0 (162.4, 169.7)	145.9 (144.3, 147.5)	112.4 (108.7, 116.1)	137.6 (135.5, 139.7)	164.0 (163.0, 165.1)	168.2 (167.2, 169.2)
urban-rural difference	4.3 (2.9, 5.8)	5.1 (3.6, 6.6)	4.6 (3.1, 6.0)	4.0 (2.5, 5.5)	3.6 (2.0, 5.1)	4.7 (4.0, 5.5)	5.5 (4.4, 6.6)	4.9 (4.1, 5.8)	4.4 (3.7, 5.0)	3.9 (3.3, 4.6)
Caribbean										
Antigua and Barbuda										
rural	150.0 (145.0, 154.8)	115.3 (105.3, 125.8)	141.6 (137.1, 146.2)	168.9 (163.7, 174.2)	172.5 (165.1, 179.9)	153.9 (149.0, 159.0)	118.6 (106.8, 130.8)	145.4 (139.9, 151.1)	173.1 (169.3, 177.0)	177.0 (172.4, 181.8)
urban	152.2 (147.2, 157.2)	117.6 (107.6, 128.4)	143.9 (139.2, 148.7)	171.1 (165.9, 176.4)	174.7 (167.1, 182.0)	155.4 (150.4, 160.6)	120.3 (108.6, 132.3)	147.0 (141.4, 152.6)	174.6 (170.6, 178.6)	178.4 (173.7, 183.3)
urban-rural difference	2.3 (0.2, 4.4)	2.4 (0.1, 4.6)	2.3 (0.2, 4.4)	2.2 (0.0, 4.4)	2.2 (-0.3, 4.6)	1.5 (-0.7, 3.7)	1.6 (-1.2, 4.3)	1.5 (-0.7, 3.9)	1.5 (-0.6, 3.6)	1.4 (-0.6, 3.5)
Bahamas										
rural	146.4 (142.0, 150.9)	111.1 (101.5, 120.9)	138.0 (133.7, 142.4)	165.6 (160.8, 170.5)	169.2 (162.8, 175.8)	150.4 (145.8, 154.8)	115.4 (104.0, 126.6)	142.1 (136.7, 147.1)	169.4 (166.2, 172.7)	172.8 (169.7, 176.3)
urban	148.7 (144.5, 153.0)	113.5 (103.8, 123.2)	140.3 (136.2, 144.4)	167.9 (163.3, 172.4)	171.4 (165.2, 177.7)	151.9 (147.5, 156.2)	117.0 (105.5, 128.3)	143.6 (138.6, 148.5)	170.9 (168.0, 173.9)	174.3 (171.4, 177.3)
urban-rural difference	2.3 (0.1, 4.4)	2.4 (0.1, 4.6)	2.3 (0.2, 4.4)	2.3 (0.0, 4.4)	2.2 (-0.2, 4.6)	1.5 (-0.5, 3.6)	1.6 (-1.1, 4.3)	1.6 (-0.7, 3.8)	1.5 (-0.5, 3.5)	1.4 (-0.5, 3.4)
Barbados										
rural	149.0 (144.6, 153.5)	114.1 (104.0, 124.1)	140.7 (136.5, 144.9)	168.1 (163.3, 172.7)	171.7 (165.1, 178.0)	153.0 (148.6, 157.6)	117.7 (105.9, 129.3)	144.6 (139.6, 149.8)	172.2 (169.1, 175.4)	176.0 (172.8, 179.4)
urban	151.7 (147.1, 156.2)	116.7 (106.5, 126.8)	143.3 (139.0, 147.6)	170.7 (165.9, 175.4)	174.3 (167.8, 180.9)	154.9 (150.3, 159.4)	119.5 (108.0, 131.3)	146.4 (141.2, 151.5)	174.0 (170.7, 177.3)	177.8 (174.4, 181.2)

Boys	Mean height in 1990 (cm)					Mean height in 2020 (cm)				
	Age-standardised	5 years	10 years	15 years	19 years	Age-standardised	5 years	10 years	15 years	19 years
urban-rural difference	2.6 (0.6, 4.7)	2.6 (0.5, 4.9)	2.6 (0.6, 4.7)	2.6 (0.5, 4.8)	2.6 (0.2, 5.0)	1.8 (-0.3, 4.0)	1.8 (-0.8, 4.5)	1.8 (-0.4, 4.1)	1.8 (-0.1, 3.8)	1.8 (-0.1, 3.8)
Belize										
rural	143.9 (139.8, 148.2)	108.5 (98.7, 118.7)	135.7 (131.7, 140.0)	163.2 (158.9, 167.6)	166.1 (160.5, 171.9)	148.0 (143.5, 152.6)	113.4 (102.0, 125.3)	140.0 (134.8, 145.2)	166.9 (163.6, 170.2)	169.3 (166.4, 172.2)
urban	146.3 (142.0, 150.4)	110.9 (101.1, 121.3)	138.1 (133.9, 142.4)	165.5 (161.1, 169.9)	168.4 (162.7, 174.1)	149.6 (145.0, 154.1)	115.0 (103.5, 126.8)	141.6 (136.5, 146.7)	168.5 (165.3, 171.7)	170.8 (168.0, 173.7)
urban-rural difference	2.3 (0.2, 4.4)	2.4 (0.2, 4.6)	2.4 (0.3, 4.4)	2.3 (0.1, 4.5)	2.3 (-0.2, 4.7)	1.6 (-0.5, 3.7)	1.7 (-1.0, 4.4)	1.6 (-0.6, 3.8)	1.6 (-0.5, 3.5)	1.5 (-0.5, 3.5)
Bermuda										
rural	147.5 (140.7, 154.1)	112.2 (100.7, 123.6)	139.0 (132.3, 145.5)	166.6 (160.0, 173.4)	170.6 (162.3, 178.9)	151.4 (144.4, 158.3)	116.3 (103.8, 129.0)	142.9 (135.7, 150.2)	170.4 (164.1, 176.8)	174.3 (167.1, 181.4)
urban	149.8 (143.0, 156.5)	114.6 (103.0, 126.1)	141.3 (134.6, 147.9)	168.9 (162.2, 175.8)	172.8 (164.6, 181.3)	152.9 (146.0, 159.8)	117.9 (105.2, 130.5)	144.5 (137.3, 151.7)	172.0 (165.6, 178.2)	175.8 (168.7, 182.9)
urban-rural difference	2.3 (0.2, 4.5)	2.4 (0.2, 4.8)	2.3 (0.2, 4.5)	2.3 (0.1, 4.5)	2.2 (-0.2, 4.7)	1.6 (-0.6, 3.7)	1.7 (-1.1, 4.4)	1.6 (-0.7, 3.8)	1.5 (-0.5, 3.5)	1.5 (-0.5, 3.5)
Cuba										
rural	143.9 (140.5, 147.3)	108.4 (98.7, 118.5)	134.9 (131.4, 138.3)	163.2 (160.4, 166.1)	168.3 (164.7, 172.1)	149.2 (144.7, 153.7)	113.6 (102.1, 125.3)	140.4 (135.4, 145.6)	168.4 (165.4, 171.5)	172.9 (170.1, 175.8)
urban	145.6 (142.5, 148.7)	110.2 (100.5, 120.3)	136.6 (133.4, 139.8)	164.9 (162.5, 167.2)	169.9 (166.8, 173.1)	150.2 (145.9, 154.6)	114.8 (103.4, 126.5)	141.6 (136.6, 146.6)	169.4 (166.6, 172.4)	173.9 (171.2, 176.7)
urban-rural difference	1.7 (0.0, 3.4)	1.8 (-0.2, 3.8)	1.7 (-0.1, 3.5)	1.6 (-0.2, 3.4)	1.6 (-0.5, 3.6)	1.1 (-0.8, 2.9)	1.2 (-1.3, 3.8)	1.1 (-0.9, 3.2)	1.0 (-0.6, 2.7)	1.0 (-0.6, 2.6)
Dominica										
rural	149.9 (145.6, 154.2)	114.7 (104.7, 124.9)	141.3 (137.1, 145.5)	169.0 (164.4, 173.5)	173.4 (167.4, 179.4)	153.6 (148.8, 158.4)	117.7 (106.1, 129.7)	144.8 (139.3, 150.3)	173.1 (169.7, 176.5)	177.9 (174.5, 181.4)
urban	152.3 (148.0, 156.4)	117.2 (107.3, 127.1)	143.7 (139.5, 147.9)	171.4 (166.9, 175.8)	175.7 (169.7, 181.5)	155.2 (150.6, 160.0)	119.3 (107.8, 131.3)	146.4 (141.1, 151.8)	174.7 (171.3, 178.0)	179.4 (176.1, 182.8)
urban-rural difference	2.4 (0.2, 4.5)	2.4 (0.2, 4.7)	2.4 (0.3, 4.5)	2.3 (0.2, 4.6)	2.3 (-0.1, 4.8)	1.6 (-0.5, 3.8)	1.7 (-1.0, 4.4)	1.6 (-0.6, 3.9)	1.6 (-0.4, 3.7)	1.5 (-0.5, 3.6)
Dominican Republic										
rural	147.1 (143.6, 150.6)	111.7 (101.8, 121.3)	138.9 (135.1, 142.7)	166.2 (163.3, 169.1)	169.5 (166.2, 172.8)	150.0 (145.9, 154.1)	114.9 (103.8, 125.8)	141.5 (136.8, 146.3)	169.1 (166.4, 171.7)	172.9 (170.8, 175.1)
urban	149.0 (145.5, 152.3)	113.8 (104.0, 123.7)	140.8 (137.0, 144.6)	168.0 (165.2, 170.8)	171.1 (167.9, 174.4)	151.2 (147.1, 155.4)	116.2 (105.2, 127.3)	142.8 (138.2, 147.7)	170.2 (167.5, 172.9)	173.9 (171.8, 176.0)
urban-rural difference	1.9 (0.1, 3.5)	2.0 (0.0, 4.0)	1.9 (0.1, 3.6)	1.8 (0.0, 3.4)	1.7 (-0.3, 3.5)	1.2 (-0.4, 2.8)	1.4 (-1.0, 3.9)	1.2 (-0.5, 3.1)	1.1 (-0.2, 2.5)	1.0 (-0.2, 2.2)
Grenada										
rural	149.9 (145.2, 154.8)	114.7 (104.6, 125.6)	141.3 (136.4, 146.2)	169.0 (164.1, 174.1)	173.5 (167.5, 179.6)	153.6 (148.2, 158.9)	118.2 (106.6, 130.2)	144.9 (138.8, 150.8)	172.8 (168.4, 177.1)	177.3 (173.3, 181.4)
urban	152.2 (147.5, 157.1)	117.1 (106.9, 127.9)	143.6 (138.7, 148.5)	171.3 (166.4, 176.3)	175.7 (169.5, 181.9)	155.1 (149.8, 160.5)	119.9 (108.3, 131.9)	146.5 (140.5, 152.3)	174.3 (169.7, 178.7)	178.8 (174.8, 182.9)
urban-rural difference	2.3 (0.1, 4.4)	2.4 (0.1, 4.6)	2.3 (0.2, 4.4)	2.2 (0.0, 4.5)	2.2 (-0.2, 4.7)	1.6 (-0.5, 3.7)	1.7 (-1.0, 4.4)	1.6 (-0.7, 3.9)	1.5 (-0.5, 3.5)	1.5 (-0.6, 3.5)
Guyana										
rural	145.2 (141.1, 149.3)	110.0 (100.3, 119.8)	136.6 (132.7, 140.4)	164.2 (159.8, 168.6)	168.6 (162.9, 174.3)	148.9 (144.5, 153.2)	114.2 (102.9, 125.8)	140.4 (135.4, 145.3)	167.7 (164.8, 170.5)	171.9 (169.5, 174.1)
urban	148.1 (144.0, 152.3)	113.0 (103.1, 122.9)	139.5 (135.6, 143.4)	167.2 (162.8, 171.5)	171.6 (165.9, 177.4)	151.1 (146.8, 155.3)	116.4 (105.0, 128.0)	142.6 (137.7, 147.6)	170.0 (167.0, 172.8)	174.1 (171.7, 176.5)
urban-rural difference	3.0 (1.3, 4.6)	2.9 (1.0, 4.9)	2.9 (1.2, 4.7)	3.0 (1.2, 4.6)	3.0 (1.0, 4.9)	2.2 (0.5, 4.0)	2.2 (-0.3, 4.7)	2.2 (0.3, 4.2)	2.2 (0.7, 3.8)	2.2 (0.8, 3.7)
Haiti										
rural	146.9 (141.6, 152.2)	111.6 (101.2, 122.0)	138.5 (133.1, 143.6)	166.1 (160.7, 171.7)	170.0 (163.4, 177.1)	150.8 (145.2, 156.3)	115.7 (103.8, 127.6)	142.4 (136.2, 148.6)	169.9 (165.3, 174.5)	173.6 (169.5, 177.8)
urban	149.1 (143.9, 154.3)	113.9 (103.6, 124.3)	140.7 (135.4, 145.9)	168.3 (162.9, 173.7)	172.1 (165.4, 178.9)	152.3 (146.8, 157.7)	117.3 (105.3, 129.3)	143.9 (137.7, 150.0)	171.3 (166.8, 175.8)	175.0 (170.9, 179.0)
urban-rural difference	2.2 (0.1, 4.2)	2.3 (0.0, 4.5)	2.2 (0.2, 4.2)	2.1 (0.0, 4.2)	2.1 (-0.3, 4.5)	1.5 (-0.7, 3.5)	1.6 (-1.1, 4.2)	1.5 (-0.8, 3.7)	1.4 (-0.6, 3.4)	1.4 (-0.6, 3.3)
Jamaica										
rural	149.0 (146.0, 152.1)	112.3 (104.3, 120.8)	140.2 (137.3, 143.1)	168.7 (165.7, 171.8)	173.4 (169.5, 177.3)	152.1 (148.5, 155.9)	116.8 (107.1, 126.9)	143.6 (139.5, 148.1)	171.1 (168.6, 173.8)	175.2 (172.9, 177.6)
urban	150.8 (147.9, 153.8)	114.2 (106.3, 122.5)	142.0 (139.0, 144.9)	170.4 (167.5, 173.5)	175.1 (171.4, 178.9)	153.0 (149.3, 156.8)	117.9 (108.3, 127.8)	144.6 (140.3, 149.0)	172.0 (169.4, 174.8)	176.1 (173.7, 178.4)
urban-rural difference	1.8 (0.2, 3.3)	1.9 (0.0, 3.9)	1.8 (0.2, 3.4)	1.7 (0.2, 3.3)	1.7 (-0.1, 3.4)	1.0 (-0.8, 2.7)	1.1 (-1.4, 3.6)	1.0 (-0.9, 2.9)	0.9 (-0.6, 2.4)	0.8 (-0.6, 2.2)
Puerto Rico										
rural	147.5 (140.4, 154.2)	112.1 (100.6, 123.6)	139.0 (132.2, 145.6)	166.7 (159.7, 173.5)	170.7 (162.2, 179.2)	151.3 (144.5, 158.1)	116.1 (103.5, 128.8)	142.8 (135.8, 149.9)	170.4 (164.0, 176.5)	174.3 (167.3, 181.3)
urban	149.8 (143.0, 156.7)	114.5 (102.8, 126.1)	141.3 (134.4, 148.1)	169.0 (161.9, 175.9)	172.9 (164.3, 181.5)	152.9 (146.1, 159.6)	117.8 (105.1, 130.6)	144.5 (137.3, 151.5)	172.0 (165.5, 178.3)	175.8 (168.9, 182.9)
urban-rural difference	2.3 (0.2, 4.5)	2.4 (0.1, 4.8)	2.4 (0.2, 4.5)	2.3 (0.1, 4.5)	2.2 (-0.3, 4.7)	1.6 (-0.6, 3.8)	1.7 (-1.1, 4.5)	1.6 (-0.7, 3.9)	1.6 (-0.5, 3.6)	1.5 (-0.5, 3.5)
Saint Kitts and Nevis										
rural	148.3 (144.1, 152.5)	114.1 (104.6, 124.6)	140.3 (136.2, 144.4)	167.1 (162.6, 171.4)	169.7 (163.7, 175.7)	152.9 (148.4, 157.3)	118.3 (106.9, 130.0)	144.8 (139.7, 149.9)	171.8 (168.7, 174.8)	174.5 (171.3, 178.1)
urban	150.6 (146.4, 155.0)	116.5 (106.9, 126.9)	142.6 (138.5, 146.9)	169.4 (164.9, 173.8)	172.0 (166.0, 178.0)	154.4 (149.7, 158.9)	119.9 (108.4, 131.3)	146.3 (141.1, 151.4)	173.3 (170.1, 176.5)	176.0 (172.6, 179.6)
urban-rural difference	2.3 (0.2, 4.4)	2.4 (0.0, 4.6)	2.3 (0.2, 4.4)	2.3 (0.1, 4.5)	2.3 (-0.2, 4.7)	1.5 (-0.6, 3.7)	1.6 (-1.2, 4.3)	1.5 (-0.8, 3.8)	1.5 (-0.5, 3.6)	1.5 (-0.5, 3.5)
Saint Lucia										
rural	148.7 (144.0, 153.4)	113.4 (103.2, 123.8)	140.2 (135.4, 145.0)	167.9 (162.8, 172.7)	172.0 (165.8, 178.1)	152.4 (147.2, 157.6)	117.1 (105.0, 128.9)	143.9 (138.0, 149.7)	171.6 (167.2, 176.0)	175.8 (171.8, 179.7)
urban	151.0 (146.0, 155.9)	115.8 (105.5, 126.3)	142.5 (137.5, 147.4)	170.1 (164.9, 175.1)	174.2 (167.8, 180.5)	154.0 (148.6, 159.3)	118.7 (106.5, 130.4)	145.4 (139.4, 151.4)	173.1 (168.4, 177.6)	177.3 (173.3, 181.2)
urban-rural difference	2.3 (0.1, 4.4)	2.4 (0.0, 4.6)	2.3 (0.1, 4.4)	2.2 (0.0, 4.4)	2.2 (-0.4, 4.7)	1.5 (-0.6, 3.7)	1.6 (-1.1, 4.4)	1.6 (-0.7, 3.9)	1.5 (-0.5, 3.5)	1.5 (-0.6, 3.5)
Saint Vincent and the Grenadines										
rural	150.0 (145.5, 154.6)	115.3 (105.2, 125.6)	141.6 (137.3, 146.1)	168.9 (164.2, 173.7)	172.7 (166.1, 179.2)	153.9 (150.2, 157.8)	119.4 (108.3, 130.7)	145.6 (141.5, 149.9)	172.8 (170.4, 175.1)	176.5 (174.0, 179.0)

Boys	Mean height in 1990 (cm)					Mean height in 2020 (cm)				
	Age-standardised	5 years	10 years	15 years	19 years	Age-standardised	5 years	10 years	15 years	19 years
urban	152.3 (147.9, 156.9)	117.7 (107.9, 128.0)	144.0 (139.7, 148.3)	171.2 (166.3, 175.9)	175.0 (168.4, 181.3)	155.5 (151.7, 159.3)	121.1 (110.0, 132.4)	147.2 (143.0, 151.5)	174.3 (172.0, 176.6)	178.0 (175.6, 180.5)
urban-rural difference	2.3 (0.2, 4.5)	2.4 (0.1, 4.7)	2.3 (0.3, 4.5)	2.3 (0.0, 4.6)	2.2 (-0.2, 4.7)	1.6 (-0.6, 3.7)	1.7 (-1.0, 4.4)	1.6 (-0.7, 3.9)	1.5 (-0.5, 3.6)	1.5 (-0.6, 3.5)
Suriname										
rural	146.8 (141.3, 152.4)	111.4 (100.8, 122.3)	138.3 (132.9, 143.9)	166.0 (160.2, 171.8)	169.8 (162.6, 177.1)	150.6 (144.8, 156.3)	115.6 (103.5, 127.7)	142.2 (135.7, 148.5)	169.6 (164.6, 174.5)	173.2 (168.6, 177.8)
urban	149.1 (143.7, 154.6)	113.8 (103.3, 124.5)	140.6 (135.3, 146.1)	168.2 (162.6, 173.8)	172.0 (165.0, 179.1)	152.1 (146.3, 157.7)	117.3 (105.1, 129.3)	143.8 (137.4, 149.9)	171.1 (166.4, 175.8)	174.7 (170.2, 179.0)
urban-rural difference	2.3 (0.2, 4.4)	2.4 (0.2, 4.7)	2.3 (0.2, 4.4)	2.3 (0.0, 4.5)	2.2 (-0.2, 4.6)	1.6 (-0.5, 3.7)	1.7 (-1.0, 4.4)	1.6 (-0.7, 3.9)	1.5 (-0.4, 3.5)	1.5 (-0.5, 3.4)
Trinidad and Tobago										
rural	147.9 (145.1, 150.7)	111.9 (109.5, 114.4)	139.3 (136.7, 142.0)	167.3 (163.8, 171.0)	171.3 (166.5, 176.2)	151.7 (147.9, 155.5)	115.3 (108.7, 122.0)	143.0 (138.5, 147.6)	171.4 (168.2, 174.5)	175.6 (172.6, 178.8)
urban	149.8 (146.9, 152.7)	113.9 (111.4, 116.4)	141.2 (138.5, 143.9)	169.2 (165.6, 173.0)	173.1 (168.2, 178.1)	152.8 (149.0, 156.7)	116.5 (109.9, 123.0)	144.1 (139.6, 148.7)	172.5 (169.3, 175.7)	176.7 (173.7, 179.8)
urban-rural difference	1.9 (0.4, 3.3)	2.0 (0.6, 3.3)	1.9 (0.5, 3.2)	1.9 (0.2, 3.5)	1.8 (-0.2, 3.8)	1.1 (-0.4, 2.7)	1.2 (-0.7, 3.2)	1.1 (-0.4, 2.8)	1.1 (-0.4, 2.6)	1.1 (-0.5, 2.7)
Central Latin America										
Colombia										
rural	140.1 (138.2, 142.0)	104.1 (102.1, 106.3)	130.7 (128.9, 132.6)	159.5 (157.3, 161.8)	165.6 (162.3, 169.0)	144.6 (143.2, 146.2)	107.0 (104.4, 109.5)	135.1 (133.5, 136.8)	164.9 (163.4, 166.3)	170.9 (169.4, 172.5)
urban	143.3 (141.5, 145.1)	107.4 (105.4, 109.5)	134.0 (132.2, 135.8)	162.7 (160.6, 164.9)	168.7 (165.5, 171.9)	146.5 (145.1, 147.8)	108.9 (106.4, 111.3)	137.0 (135.4, 138.5)	166.7 (165.3, 168.0)	172.6 (171.2, 174.1)
urban-rural difference	3.2 (2.1, 4.3)	3.3 (2.1, 4.5)	3.2 (2.1, 4.3)	3.2 (2.0, 4.3)	3.1 (1.9, 4.3)	1.8 (1.0, 2.7)	1.9 (0.9, 3.0)	1.9 (1.0, 2.7)	1.8 (1.0, 2.6)	1.7 (0.8, 2.6)
Costa Rica										
rural	144.8 (140.8, 148.9)	110.5 (101.7, 119.5)	136.8 (132.7, 140.7)	163.5 (159.1, 167.8)	166.4 (160.6, 172.1)	147.3 (143.1, 151.4)	112.4 (102.2, 123.0)	139.0 (134.3, 143.7)	166.2 (163.2, 169.2)	169.4 (166.7, 172.2)
urban	147.5 (143.7, 151.3)	113.3 (104.6, 122.1)	139.5 (135.7, 143.3)	166.1 (161.9, 170.1)	168.9 (163.4, 174.3)	148.5 (144.5, 152.4)	113.8 (103.4, 124.2)	140.3 (135.7, 144.8)	167.4 (164.7, 170.1)	170.6 (168.3, 172.9)
urban-rural difference	2.7 (0.6, 4.7)	2.8 (0.6, 5.0)	2.7 (0.6, 4.7)	2.6 (0.5, 4.7)	2.6 (0.3, 4.8)	1.3 (-0.7, 3.1)	1.4 (-1.1, 3.8)	1.3 (-0.8, 3.3)	1.2 (-0.7, 3.0)	1.1 (-0.9, 3.0)
El Salvador										
rural	143.0 (138.5, 147.7)	107.4 (98.1, 116.6)	134.4 (129.6, 139.3)	162.3 (157.3, 167.0)	166.4 (160.5, 172.0)	145.9 (140.9, 150.9)	110.9 (100.2, 121.5)	137.5 (131.8, 143.3)	164.9 (160.6, 168.9)	168.5 (165.3, 171.5)
urban	146.0 (141.5, 150.6)	110.4 (101.0, 119.7)	137.5 (132.7, 142.2)	165.3 (160.5, 169.9)	169.4 (163.6, 174.8)	147.5 (142.6, 152.5)	112.5 (101.7, 123.3)	139.2 (133.4, 144.8)	166.5 (162.4, 170.6)	170.1 (167.1, 173.0)
urban-rural difference	3.0 (1.2, 4.9)	3.1 (0.9, 5.1)	3.0 (1.2, 4.9)	3.0 (1.1, 4.9)	3.0 (0.9, 5.0)	1.6 (-0.3, 3.5)	1.7 (-0.8, 4.1)	1.6 (-0.4, 3.6)	1.6 (-0.2, 3.3)	1.6 (-0.1, 3.2)
Guatemala										
rural	137.9 (135.7, 140.2)	104.4 (100.3, 108.5)	129.5 (126.8, 132.0)	156.4 (153.8, 159.0)	160.3 (157.0, 163.6)	140.4 (138.8, 142.0)	108.0 (105.6, 110.4)	132.3 (130.4, 134.2)	158.3 (156.6, 160.0)	161.7 (159.6, 163.8)
urban	141.2 (138.9, 143.6)	107.7 (103.6, 111.9)	132.8 (130.1, 135.4)	159.6 (157.0, 162.2)	163.5 (160.2, 166.9)	142.4 (140.7, 144.1)	110.1 (107.7, 112.6)	134.4 (132.4, 136.3)	160.3 (158.5, 162.0)	163.6 (161.5, 165.7)
urban-rural difference	3.3 (2.0, 4.6)	3.3 (1.6, 5.2)	3.3 (1.9, 4.8)	3.3 (2.0, 4.6)	3.2 (1.8, 4.7)	2.0 (0.5, 3.6)	2.1 (-0.1, 4.3)	2.0 (0.4, 3.8)	2.0 (0.7, 3.4)	2.0 (0.7, 3.2)
Honduras										
rural	141.9 (136.9, 146.8)	106.5 (96.5, 116.2)	133.4 (128.2, 138.5)	161.1 (156.0, 166.1)	164.9 (158.7, 170.9)	145.0 (139.4, 150.4)	109.8 (99.0, 120.7)	136.6 (130.6, 142.5)	164.0 (159.1, 169.0)	167.5 (162.5, 172.6)
urban	144.8 (140.1, 149.7)	109.5 (99.7, 119.1)	136.4 (131.3, 141.5)	164.0 (159.2, 168.8)	167.7 (161.8, 173.6)	146.6 (141.1, 152.0)	111.4 (100.6, 122.5)	138.2 (132.3, 144.2)	165.6 (160.6, 170.4)	169.0 (164.0, 174.1)
urban-rural difference	2.9 (0.9, 4.9)	3.0 (0.8, 5.2)	3.0 (0.9, 4.9)	2.9 (0.8, 4.9)	2.9 (0.6, 5.1)	1.6 (-0.4, 3.6)	1.7 (-0.8, 4.1)	1.6 (-0.5, 3.7)	1.6 (-0.4, 3.5)	1.5 (-0.4, 3.5)
Mexico										
rural	141.8 (140.3, 143.3)	105.8 (104.2, 107.5)	133.6 (132.1, 135.1)	160.9 (159.1, 162.7)	164.3 (161.6, 166.9)	145.2 (144.0, 146.3)	108.7 (107.0, 110.4)	137.0 (135.8, 138.3)	164.6 (163.4, 165.8)	167.9 (166.6, 169.3)
urban	145.6 (144.1, 147.0)	109.6 (108.0, 111.2)	137.4 (135.9, 138.8)	164.7 (162.9, 166.4)	168.1 (165.5, 170.7)	147.3 (146.1, 148.5)	110.8 (109.1, 112.5)	139.1 (137.9, 140.4)	166.7 (165.5, 167.9)	170.1 (168.8, 171.5)
urban-rural difference	3.8 (2.9, 4.7)	3.7 (2.8, 4.7)	3.8 (2.9, 4.7)	3.8 (2.9, 4.8)	3.8 (2.8, 4.9)	2.1 (1.4, 2.8)	2.1 (1.2, 2.9)	2.1 (1.4, 2.8)	2.1 (1.4, 2.8)	2.2 (1.4, 2.9)
Nicaragua										
rural	141.9 (136.7, 146.9)	106.5 (96.7, 116.3)	133.4 (128.0, 138.7)	161.1 (155.9, 166.1)	164.8 (158.6, 170.8)	145.0 (139.6, 150.3)	109.9 (99.3, 120.5)	136.6 (130.7, 142.5)	164.1 (159.1, 169.0)	167.5 (162.4, 172.6)
urban	144.9 (139.8, 149.6)	109.5 (99.8, 119.3)	136.4 (131.1, 141.6)	164.0 (159.0, 168.8)	167.7 (161.9, 173.5)	146.6 (141.3, 152.0)	111.5 (100.8, 122.1)	138.3 (132.4, 144.0)	165.6 (160.8, 170.6)	169.0 (164.2, 174.0)
urban-rural difference	3.0 (0.9, 5.0)	3.0 (0.8, 5.3)	3.0 (0.9, 5.0)	2.9 (0.8, 5.0)	2.9 (0.6, 5.1)	1.6 (-0.4, 3.6)	1.7 (-0.8, 4.2)	1.6 (-0.5, 3.7)	1.6 (-0.3, 3.5)	1.5 (-0.4, 3.5)
Panama										
rural	141.7 (139.1, 144.3)	106.2 (103.1, 109.3)	133.0 (130.3, 135.6)	161.0 (158.0, 164.0)	165.2 (161.3, 168.9)	144.6 (142.3, 147.0)	110.3 (105.3, 115.5)	136.2 (133.3, 139.2)	163.4 (161.7, 165.1)	166.8 (165.0, 168.6)
urban	146.7 (144.1, 149.2)	111.3 (108.2, 114.4)	138.0 (135.3, 140.6)	165.9 (162.9, 169.0)	170.1 (166.2, 173.8)	148.0 (145.7, 150.4)	113.8 (108.8, 118.9)	139.6 (136.7, 142.6)	166.7 (165.0, 168.4)	170.1 (168.3, 172.0)
urban-rural difference	5.0 (3.7, 6.3)	5.1 (3.6, 6.5)	5.0 (3.7, 6.3)	4.9 (3.6, 6.3)	4.9 (3.4, 6.4)	3.4 (2.1, 4.6)	3.5 (1.8, 5.1)	3.4 (2.1, 4.8)	3.3 (2.2, 4.5)	3.3 (2.1, 4.5)
Venezuela										
rural	146.4 (143.0, 150.0)	109.2 (102.0, 116.3)	138.1 (134.5, 141.7)	166.4 (162.6, 170.3)	169.3 (164.3, 174.1)	148.2 (144.6, 151.6)	112.8 (104.7, 120.5)	140.3 (136.3, 144.3)	167.4 (164.5, 170.2)	169.3 (166.9, 171.8)
urban	147.5 (144.4, 150.7)	110.7 (103.9, 117.5)	139.3 (136.1, 142.6)	167.3 (163.8, 170.9)	170.0 (165.4, 174.6)	147.8 (144.6, 150.9)	112.8 (105.1, 120.4)	140.0 (136.4, 143.7)	166.8 (164.3, 169.2)	168.6 (166.6, 170.6)
urban-rural difference	1.1 (-0.9, 3.0)	1.5 (-0.8, 3.8)	1.2 (-0.9, 3.2)	0.9 (-1.1, 2.8)	0.7 (-1.4, 2.9)	-0.4 (-2.3, 1.6)	0.1 (-2.5, 2.7)	-0.2 (-2.3, 1.8)	-0.5 (-2.4, 1.3)	-0.7 (-2.6, 1.1)
Southern Latin America										
Argentina										
rural	146.4 (143.2, 149.6)	107.7 (103.7, 111.7)	138.1 (134.7, 141.5)	166.4 (162.7, 170.3)	170.5 (165.6, 175.5)	151.1 (148.4, 153.7)	113.8 (109.1, 118.5)	143.2 (140.0, 146.4)	170.6 (168.2, 173.0)	173.7 (171.3, 176.2)
urban	149.0 (146.2, 151.9)	110.2 (106.5, 114.0)	140.7 (137.8, 143.7)	169.2 (165.8, 172.6)	173.3 (168.8, 177.7)	151.9 (149.8, 154.0)	114.5 (110.4, 118.9)	144.1 (141.4, 146.7)	171.5 (169.8, 173.1)	174.7 (172.9, 176.4)

Boys	Mean height in 1990 (cm)					Mean height in 2020 (cm)				
	Age-standardised	5 years	10 years	15 years	19 years	Age-standardised	5 years	10 years	15 years	19 years
urban-rural difference	2.7 (0.6, 4.6)	2.6 (0.4, 4.7)	2.6 (0.6, 4.6)	2.7 (0.5, 4.8)	2.8 (0.4, 5.0)	0.9 (-1.1, 2.7)	0.7 (-1.6, 3.1)	0.8 (-1.2, 2.8)	0.9 (-0.9, 2.7)	1.0 (-0.9, 2.8)
Brazil										
rural	145.1 (144.0, 146.2)	110.4 (109.2, 111.6)	136.2 (135.1, 137.3)	164.0 (162.8, 165.2)	169.2 (167.9, 170.5)	151.4 (150.6, 152.3)	116.1 (114.4, 117.8)	142.8 (141.8, 143.7)	170.7 (169.8, 171.5)	174.9 (174.0, 175.9)
urban	148.2 (147.3, 149.1)	112.8 (111.7, 113.9)	139.0 (138.0, 140.0)	167.4 (166.3, 168.5)	173.1 (171.9, 174.3)	151.8 (151.1, 152.5)	115.7 (114.1, 117.3)	142.9 (142.1, 143.7)	171.3 (170.6, 172.0)	176.0 (175.2, 176.8)
urban-rural difference	3.1 (2.3, 3.9)	2.4 (1.5, 3.2)	2.9 (2.1, 3.7)	3.4 (2.6, 4.2)	3.8 (2.9, 4.7)	0.3 (-0.3, 1.0)	-0.4 (-1.2, 0.4)	0.1 (-0.5, 0.8)	0.7 (0.1, 1.3)	1.1 (0.4, 1.8)
Chile										
rural	145.5 (143.3, 147.7)	107.3 (103.5, 111.0)	137.5 (134.9, 140.1)	165.6 (163.2, 168.0)	168.1 (165.1, 171.2)	151.4 (149.0, 153.8)	114.1 (109.7, 118.5)	143.7 (140.6, 146.8)	171.2 (169.2, 173.3)	172.9 (171.2, 174.8)
urban	147.6 (145.6, 149.6)	109.1 (105.4, 112.8)	139.6 (137.1, 142.0)	167.9 (165.7, 170.0)	170.6 (168.0, 173.3)	151.6 (149.3, 153.9)	113.9 (109.6, 118.3)	143.8 (140.9, 146.8)	171.6 (169.7, 173.5)	173.5 (171.8, 175.2)
urban-rural difference	2.2 (0.7, 3.6)	1.8 (0.2, 3.4)	2.1 (0.6, 3.5)	2.3 (0.8, 3.7)	2.5 (0.8, 4.1)	0.2 (-1.0, 1.4)	-0.1 (-1.9, 1.6)	0.1 (-1.2, 1.4)	0.3 (-0.8, 1.5)	0.5 (-0.6, 1.7)
Paraguay										
rural	146.6 (142.6, 150.8)	110.7 (102.4, 119.3)	138.0 (134.1, 142.0)	165.9 (161.4, 170.3)	170.5 (164.5, 176.5)	152.2 (149.0, 155.6)	118.6 (109.5, 127.9)	144.4 (140.7, 148.1)	170.5 (168.3, 172.7)	173.4 (170.8, 175.9)
urban	149.3 (145.4, 153.3)	113.2 (104.8, 121.9)	140.7 (136.9, 144.6)	168.7 (164.3, 173.0)	173.4 (167.4, 179.2)	153.1 (150.0, 156.4)	119.3 (110.2, 128.7)	145.3 (141.7, 149.0)	171.5 (169.4, 173.6)	174.5 (172.0, 177.0)
urban-rural difference	2.7 (0.9, 4.4)	2.5 (0.6, 4.5)	2.7 (0.9, 4.4)	2.8 (0.9, 4.5)	2.9 (0.8, 4.9)	0.9 (-0.5, 2.4)	0.7 (-1.4, 2.9)	0.9 (-0.7, 2.5)	1.0 (-0.3, 2.3)	1.1 (-0.2, 2.4)
Uruguay										
rural	145.0 (141.7, 148.3)	108.0 (104.1, 112.0)	136.3 (132.9, 139.7)	164.8 (160.7, 168.8)	168.9 (163.7, 174.3)	151.0 (148.3, 153.8)	114.7 (109.7, 119.8)	142.7 (139.5, 146.0)	170.5 (168.0, 173.0)	173.7 (170.9, 176.6)
urban	147.8 (144.8, 150.6)	110.5 (107.3, 113.9)	139.1 (136.2, 142.0)	167.7 (163.9, 171.4)	172.0 (167.0, 176.9)	152.0 (150.0, 154.1)	115.5 (111.1, 120.0)	143.7 (141.0, 146.3)	171.7 (170.0, 173.4)	175.0 (172.9, 177.1)
urban-rural difference	2.8 (0.7, 5.0)	2.6 (0.4, 4.8)	2.8 (0.6, 4.9)	2.9 (0.7, 5.1)	3.1 (0.6, 5.4)	1.1 (-1.0, 3.1)	0.8 (-1.7, 3.4)	1.0 (-1.1, 3.1)	1.2 (-0.8, 3.1)	1.3 (-0.6, 3.3)
**Oceania**										
Melanesia										
Fiji										
rural	150.2 (146.6, 154.1)	114.5 (104.2, 125.1)	141.8 (138.1, 145.9)	168.6 (165.3, 172.2)	174.3 (170.1, 178.6)	149.3 (145.2, 153.4)	114.0 (102.3, 125.8)	141.0 (136.2, 145.7)	167.5 (164.8, 170.1)	173.1 (170.6, 175.7)
urban	151.3 (147.8, 155.1)	115.9 (105.6, 126.6)	143.1 (139.3, 147.0)	169.6 (166.2, 173.3)	175.1 (170.9, 179.7)	150.2 (146.0, 154.4)	115.3 (103.4, 127.0)	142.0 (137.1, 146.7)	168.3 (165.7, 170.8)	173.7 (171.2, 176.2)
urban-rural difference	1.2 (-0.6, 3.0)	1.5 (-0.7, 3.7)	1.2 (-0.6, 3.1)	1.0 (-0.8, 2.8)	0.8 (-1.2, 2.8)	1.0 (-1.3, 3.2)	1.3 (-1.7, 4.3)	1.1 (-1.4, 3.5)	0.8 (-1.2, 2.8)	0.6 (-1.3, 2.5)
Papua New Guinea										
rural	141.6 (137.0, 146.4)	104.1 (93.2, 115.2)	133.0 (128.2, 137.9)	160.8 (156.1, 165.7)	166.9 (160.9, 172.7)	140.5 (134.9, 146.0)	106.7 (94.0, 119.1)	132.8 (126.5, 139.1)	158.2 (154.0, 162.3)	162.4 (158.7, 166.1)
urban	143.0 (138.1, 147.9)	105.6 (94.6, 116.7)	134.4 (129.3, 139.5)	162.2 (157.1, 167.3)	168.1 (162.0, 174.3)	141.6 (135.8, 147.2)	107.8 (95.1, 120.2)	133.9 (127.4, 140.3)	159.1 (154.7, 163.6)	163.4 (159.3, 167.5)
urban-rural difference	1.4 (-0.9, 3.7)	1.5 (-1.0, 4.1)	1.4 (-0.8, 3.8)	1.3 (-1.0, 3.7)	1.3 (-1.4, 3.9)	1.0 (-1.5, 3.5)	1.2 (-2.0, 4.3)	1.1 (-1.6, 3.7)	1.0 (-1.3, 3.4)	0.9 (-1.4, 3.2)
Solomon Islands										
rural	143.2 (139.7, 146.8)	105.1 (100.6, 109.9)	134.8 (131.1, 138.7)	162.7 (158.6, 167.0)	167.9 (162.8, 173.2)	140.9 (137.1, 144.5)	106.4 (100.5, 112.3)	133.3 (128.7, 137.8)	158.8 (155.3, 162.3)	162.6 (159.9, 165.4)
urban	144.8 (141.1, 148.4)	106.4 (101.7, 111.5)	136.3 (132.5, 140.3)	164.4 (160.0, 168.8)	169.7 (164.6, 175.0)	141.8 (137.9, 145.6)	107.0 (101.0, 113.1)	134.1 (129.5, 138.9)	159.8 (156.2, 163.4)	163.8 (160.8, 166.9)
urban-rural difference	1.6 (-0.4, 3.6)	1.3 (-0.9, 3.5)	1.5 (-0.5, 3.5)	1.7 (-0.3, 3.7)	1.8 (-0.4, 4.1)	0.9 (-1.2, 2.9)	0.7 (-2.0, 3.2)	0.8 (-1.4, 3.0)	1.0 (-1.0, 2.9)	1.2 (-0.8, 3.1)
Vanuatu										
rural	145.2 (140.5, 149.7)	109.4 (98.5, 120.3)	137.3 (132.4, 142.2)	163.7 (159.1, 168.3)	168.0 (162.7, 173.3)	144.8 (139.3, 150.3)	109.1 (96.7, 121.6)	136.9 (130.5, 143.2)	163.3 (158.8, 167.6)	167.7 (163.7, 171.4)
urban	146.4 (141.6, 151.2)	110.7 (99.6, 121.9)	138.5 (133.5, 143.6)	164.9 (160.1, 169.9)	169.1 (163.6, 174.8)	145.7 (140.0, 151.4)	110.1 (97.7, 122.6)	137.7 (131.2, 144.3)	164.1 (159.4, 168.6)	168.4 (164.3, 172.5)
urban-rural difference	1.2 (-1.1, 3.7)	1.4 (-1.2, 3.9)	1.3 (-1.1, 3.7)	1.2 (-1.2, 3.6)	1.2 (-1.5, 3.8)	0.9 (-1.6, 3.4)	1.0 (-2.2, 4.0)	0.9 (-1.8, 3.5)	0.8 (-1.6, 3.2)	0.8 (-1.5, 3.0)
Polynesia and Micronesia										
American Samoa										
rural	150.5 (147.5, 153.4)	114.0 (108.1, 119.7)	142.5 (138.3, 146.5)	169.1 (165.3, 173.0)	174.2 (169.8, 178.7)	151.3 (146.4, 156.1)	115.3 (107.7, 122.8)	143.3 (137.7, 148.8)	169.7 (164.9, 174.6)	174.8 (170.0, 179.6)
urban	151.4 (148.3, 154.5)	115.2 (108.9, 121.5)	143.5 (139.2, 147.6)	169.9 (166.2, 173.7)	174.8 (170.7, 179.1)	152.2 (147.5, 157.0)	116.6 (108.8, 124.5)	144.3 (138.8, 149.8)	170.5 (165.7, 175.4)	175.5 (170.9, 180.0)
urban-rural difference	0.9 (-1.5, 3.3)	1.2 (-1.4, 3.8)	1.0 (-1.4, 3.4)	0.8 (-1.6, 3.3)	0.6 (-2.0, 3.3)	1.0 (-1.6, 3.5)	1.3 (-1.9, 4.6)	1.1 (-1.7, 3.8)	0.8 (-1.6, 3.2)	0.7 (-1.7, 3.0)
Cook Islands										
rural	152.3 (148.5, 156.0)	117.6 (107.9, 127.7)	144.6 (140.5, 148.7)	170.3 (166.7, 173.9)	174.4 (170.0, 178.8)	153.8 (149.5, 158.1)	117.7 (106.5, 129.4)	145.6 (140.6, 150.6)	172.4 (169.6, 175.4)	177.6 (174.8, 180.4)
urban	153.2 (149.6, 156.9)	118.8 (109.1, 129.0)	145.6 (141.7, 149.6)	171.0 (167.8, 174.4)	175.0 (170.9, 179.2)	154.8 (150.7, 159.0)	119.1 (107.8, 130.5)	146.7 (141.9, 151.6)	173.3 (170.7, 176.0)	178.3 (175.8, 180.8)
urban-rural difference	0.9 (-1.5, 3.2)	1.2 (-1.3, 3.9)	1.0 (-1.4, 3.4)	0.8 (-1.7, 3.2)	0.6 (-2.1, 3.2)	1.0 (-1.6, 3.6)	1.3 (-1.9, 4.6)	1.1 (-1.7, 3.8)	0.9 (-1.6, 3.3)	0.7 (-1.6, 3.0)
French Polynesia										
rural	154.2 (150.0, 158.4)	120.0 (110.2, 129.9)	146.7 (142.6, 151.0)	172.0 (167.3, 176.7)	175.6 (169.4, 181.8)	156.2 (152.3, 160.4)	121.6 (110.5, 132.7)	148.6 (144.1, 153.2)	174.3 (171.5, 177.0)	178.2 (175.5, 181.0)
urban	155.1 (151.0, 159.2)	121.2 (111.6, 131.4)	147.7 (143.7, 152.0)	172.8 (168.2, 177.3)	176.2 (170.0, 182.1)	157.2 (153.4, 161.1)	122.9 (112.0, 134.1)	149.7 (145.3, 154.1)	175.1 (172.6, 177.7)	178.9 (176.4, 181.5)
urban-rural difference	0.9 (-1.5, 3.2)	1.2 (-1.3, 3.9)	1.0 (-1.3, 3.3)	0.7 (-1.7, 3.1)	0.6 (-2.1, 3.2)	1.0 (-1.5, 3.6)	1.3 (-1.9, 4.6)	1.1 (-1.6, 3.8)	0.8 (-1.5, 3.2)	0.7 (-1.6, 3.0)
Kiribati										
rural	146.4 (142.3, 150.5)	110.8 (100.9, 120.4)	138.7 (134.3, 143.1)	164.8 (160.7, 169.0)	168.9 (164.1, 173.9)	147.4 (142.5, 152.3)	112.6 (100.8, 124.5)	139.7 (134.0, 145.6)	165.5 (161.8, 169.3)	169.5 (166.6, 172.4)
urban	147.2 (143.1, 151.5)	112.0 (101.9, 121.9)	139.6 (135.2, 144.1)	165.5 (161.4, 169.7)	169.4 (164.4, 174.6)	148.3 (143.4, 153.3)	113.8 (102.1, 125.7)	140.8 (135.0, 146.6)	166.3 (162.6, 170.0)	170.1 (167.1, 173.1)

Boys	Mean height in 1990 (cm)					Mean height in 2020 (cm)				
	Age-standardised	5 years	10 years	15 years	19 years	Age-standardised	5 years	10 years	15 years	19 years
urban-rural difference	0.8 (-1.6, 3.2)	1.2 (-1.4, 3.8)	0.9 (-1.5, 3.3)	0.7 (-1.8, 3.1)	0.5 (-2.3, 3.2)	0.9 (-1.7, 3.5)	1.2 (-1.9, 4.5)	1.0 (-1.7, 3.8)	0.8 (-1.7, 3.2)	0.6 (-1.8, 3.0)
Marshall Islands										
rural	140.9 (136.8, 145.1)	104.4 (94.3, 114.1)	132.7 (128.4, 137.1)	159.7 (155.8, 163.7)	165.0 (160.4, 169.7)	141.7 (136.9, 146.7)	107.9 (96.1, 119.6)	134.2 (128.7, 139.9)	159.3 (155.8, 163.0)	163.5 (160.6, 166.4)
urban	141.6 (137.6, 145.6)	105.4 (95.3, 115.4)	133.5 (129.3, 137.8)	160.3 (156.6, 164.0)	165.4 (161.0, 169.8)	142.5 (137.8, 147.4)	108.9 (97.1, 120.9)	135.0 (129.6, 140.7)	159.9 (156.5, 163.3)	163.9 (161.4, 166.4)
urban-rural difference	0.7 (-1.7, 3.0)	1.0 (-1.6, 3.6)	0.8 (-1.6, 3.1)	0.5 (-1.9, 3.0)	0.4 (-2.4, 3.1)	0.7 (-1.8, 3.3)	1.0 (-2.2, 4.4)	0.8 (-1.9, 3.6)	0.6 (-1.9, 3.0)	0.4 (-2.0, 2.8)
Micronesia (Federated States of)										
rural	146.5 (142.8, 150.3)	110.2 (100.5, 120.0)	138.5 (134.4, 142.6)	165.2 (161.7, 168.8)	170.2 (165.9, 174.5)	147.2 (142.5, 151.8)	112.6 (101.2, 124.1)	139.5 (134.0, 144.9)	165.1 (161.7, 168.6)	169.5 (166.7, 172.4)
urban	147.2 (143.3, 151.1)	111.3 (101.5, 121.2)	139.3 (135.0, 143.6)	165.7 (162.0, 169.4)	170.3 (165.9, 174.8)	148.1 (143.1, 152.8)	113.9 (102.5, 125.4)	140.5 (134.7, 146.0)	165.8 (162.2, 169.5)	169.9 (166.9, 173.0)
urban-rural difference	0.6 (-1.3, 2.5)	1.1 (-1.2, 3.5)	0.8 (-1.2, 2.7)	0.4 (-1.6, 2.4)	0.2 (-2.0, 2.3)	0.9 (-1.4, 3.3)	1.3 (-1.7, 4.5)	1.0 (-1.5, 3.6)	0.7 (-1.4, 2.8)	0.4 (-1.5, 2.4)
Nauru										
rural	145.7 (141.6, 149.8)	110.4 (100.1, 120.8)	138.2 (133.8, 142.6)	164.1 (159.8, 168.2)	167.6 (162.4, 172.7)	147.0 (142.0, 152.0)	111.9 (99.9, 123.8)	139.5 (133.7, 145.1)	165.3 (161.4, 169.2)	168.9 (165.7, 172.2)
urban	146.5 (142.7, 150.3)	111.5 (101.3, 121.9)	139.0 (135.0, 143.1)	164.6 (160.9, 168.3)	168.0 (163.2, 172.6)	147.9 (143.1, 152.6)	113.1 (100.9, 124.9)	140.4 (134.9, 146.0)	166.0 (162.5, 169.4)	169.4 (166.9, 172.0)
urban-rural difference	0.7 (-1.6, 3.1)	1.1 (-1.5, 3.7)	0.8 (-1.5, 3.2)	0.6 (-1.9, 3.0)	0.4 (-2.3, 3.0)	0.8 (-1.8, 3.4)	1.2 (-2.0, 4.4)	0.9 (-1.9, 3.6)	0.7 (-1.8, 3.0)	0.5 (-1.9, 2.8)
Niue										
rural	153.4 (149.1, 157.6)	119.1 (109.2, 129.2)	146.1 (141.9, 150.4)	171.2 (166.7, 175.8)	174.2 (168.2, 180.4)	154.8 (151.1, 158.6)	119.8 (109.0, 130.9)	147.2 (142.8, 151.6)	173.0 (170.6, 175.4)	176.8 (174.3, 179.3)
urban	154.1 (149.9, 158.5)	120.2 (110.2, 130.3)	147.0 (142.7, 151.3)	171.9 (167.2, 176.4)	174.7 (168.5, 180.9)	155.6 (151.7, 159.6)	120.9 (110.1, 131.9)	148.1 (143.5, 152.6)	173.7 (171.2, 176.3)	177.3 (174.8, 180.0)
urban-rural difference	0.8 (-1.6, 3.1)	1.1 (-1.5, 3.7)	0.9 (-1.5, 3.2)	0.6 (-1.9, 3.0)	0.5 (-2.2, 3.1)	0.8 (-1.8, 3.4)	1.2 (-2.1, 4.4)	0.9 (-1.9, 3.7)	0.7 (-1.7, 3.1)	0.5 (-1.8, 2.9)
Palau										
rural	146.6 (141.7, 151.5)	110.8 (100.5, 121.1)	138.9 (133.8, 144.0)	165.1 (160.1, 170.2)	169.2 (162.7, 175.7)	148.3 (143.0, 153.5)	113.2 (101.1, 125.2)	140.6 (134.7, 146.6)	166.5 (162.3, 170.7)	170.3 (167.1, 173.4)
urban	147.4 (142.7, 152.2)	112.0 (101.5, 122.4)	139.7 (134.7, 144.7)	165.8 (160.8, 170.7)	169.6 (163.4, 175.9)	149.2 (143.9, 154.3)	114.4 (102.1, 126.5)	141.6 (135.6, 147.5)	167.3 (163.1, 171.3)	170.9 (168.0, 173.8)
urban-rural difference	0.8 (-1.6, 3.2)	1.1 (-1.5, 3.8)	0.9 (-1.5, 3.3)	0.6 (-1.8, 3.1)	0.5 (-2.2, 3.1)	0.9 (-1.7, 3.4)	1.2 (-2.0, 4.5)	1.0 (-1.8, 3.7)	0.7 (-1.6, 3.1)	0.6 (-1.8, 2.8)
Samoa										
rural	146.3 (144.4, 148.1)	110.1 (106.3, 113.8)	138.2 (135.3, 141.1)	164.8 (162.2, 167.4)	170.3 (167.3, 173.4)	147.8 (143.8, 151.9)	110.1 (102.9, 117.1)	139.0 (134.2, 143.9)	167.0 (163.4, 170.9)	174.1 (171.3, 176.9)
urban	146.9 (144.6, 149.2)	111.1 (106.9, 115.3)	138.9 (135.8, 142.1)	165.2 (162.3, 168.1)	170.5 (167.0, 173.9)	148.5 (144.3, 152.8)	111.3 (103.7, 118.4)	139.8 (134.9, 144.6)	167.6 (163.7, 171.5)	174.3 (171.2, 177.4)
urban-rural difference	0.6 (-1.5, 2.7)	1.0 (-1.4, 3.6)	0.7 (-1.4, 2.8)	0.4 (-1.8, 2.5)	0.2 (-2.3, 2.5)	0.7 (-1.7, 3.2)	1.1 (-2.0, 4.3)	0.8 (-1.7, 3.5)	0.5 (-1.7, 2.7)	0.3 (-1.9, 2.4)
Tokelau										
rural	151.5 (147.4, 155.5)	116.3 (106.1, 126.3)	143.8 (139.4, 148.0)	169.7 (165.5, 173.9)	173.7 (168.6, 178.9)	152.7 (147.9, 157.5)	116.7 (104.6, 128.6)	144.7 (139.0, 150.3)	171.3 (167.8, 174.9)	176.1 (173.3, 178.9)
urban	152.2 (147.6, 156.9)	117.3 (107.1, 127.7)	144.6 (139.7, 149.4)	170.3 (165.5, 175.0)	174.1 (168.5, 179.9)	153.5 (148.4, 158.6)	117.8 (105.7, 129.9)	145.6 (139.7, 151.4)	172.0 (168.0, 175.9)	176.5 (173.1, 180.1)
urban-rural difference	0.7 (-1.7, 3.1)	1.0 (-1.6, 3.7)	0.8 (-1.6, 3.2)	0.6 (-1.9, 3.0)	0.4 (-2.3, 3.0)	0.8 (-1.8, 3.3)	1.1 (-2.2, 4.3)	0.8 (-1.9, 3.6)	0.6 (-1.8, 3.0)	0.4 (-1.9, 2.7)
Tonga										
rural	151.0 (147.6, 154.5)	116.2 (107.5, 125.4)	143.1 (139.9, 146.5)	169.0 (165.3, 172.7)	174.1 (169.0, 179.1)	150.9 (147.5, 154.2)	115.7 (105.6, 126.2)	142.6 (138.8, 146.4)	169.0 (166.9, 171.1)	175.0 (173.1, 176.9)
urban	151.7 (147.9, 155.5)	117.2 (108.1, 126.7)	143.8 (140.2, 147.6)	169.5 (165.4, 173.5)	174.5 (169.0, 179.7)	151.7 (147.9, 155.6)	116.9 (106.5, 127.6)	143.5 (139.2, 147.8)	169.7 (167.0, 172.4)	175.4 (172.9, 178.0)
urban-rural difference	0.7 (-1.7, 3.0)	1.0 (-1.7, 3.6)	0.8 (-1.7, 3.1)	0.5 (-2.0, 2.9)	0.3 (-2.4, 3.0)	0.8 (-1.8, 3.3)	1.1 (-2.1, 4.3)	0.9 (-1.8, 3.6)	0.6 (-1.8, 3.0)	0.5 (-1.9, 2.8)
Tuvalu										
rural	148.3 (143.6, 153.0)	113.0 (103.1, 122.9)	140.6 (136.0, 145.2)	166.5 (161.5, 171.4)	170.8 (163.9, 177.6)	148.6 (144.0, 153.3)	114.0 (102.4, 125.8)	140.9 (135.6, 146.5)	166.6 (163.4, 169.9)	170.9 (168.1, 173.7)
urban	149.1 (144.6, 153.8)	114.1 (104.3, 124.3)	141.5 (137.0, 146.3)	167.3 (162.2, 172.2)	171.3 (164.5, 178.0)	149.6 (145.1, 154.2)	115.3 (103.9, 127.0)	141.9 (136.7, 147.4)	167.4 (164.2, 170.5)	171.5 (168.9, 174.1)
urban-rural difference	0.9 (-1.5, 3.3)	1.2 (-1.4, 3.9)	0.9 (-1.4, 3.3)	0.7 (-1.7, 3.2)	0.5 (-2.2, 3.2)	0.9 (-1.7, 3.6)	1.2 (-2.0, 4.7)	1.0 (-1.8, 3.9)	0.8 (-1.7, 3.2)	0.6 (-1.9, 3.0)
**South Asia**										
Afghanistan										
rural	141.0 (136.0, 146.0)	104.7 (94.6, 114.8)	132.6 (127.6, 137.8)	159.6 (154.4, 164.7)	165.7 (159.2, 172.1)	143.0 (138.5, 147.7)	106.8 (95.9, 117.8)	134.7 (129.3, 140.1)	161.5 (158.0, 165.1)	167.8 (165.3, 170.3)
urban	142.3 (137.2, 147.5)	106.4 (96.4, 116.5)	134.0 (128.9, 139.3)	160.7 (155.5, 166.0)	166.7 (160.1, 173.2)	144.1 (139.4, 148.7)	108.2 (97.1, 119.2)	135.8 (130.4, 141.3)	162.4 (158.9, 165.9)	168.5 (166.1, 171.0)
urban-rural difference	1.3 (-0.5, 3.1)	1.6 (-0.4, 3.8)	1.4 (-0.5, 3.2)	1.2 (-0.7, 2.9)	1.0 (-1.1, 2.9)	1.1 (-0.7, 2.8)	1.4 (-1.0, 3.8)	1.2 (-0.8, 3.1)	0.9 (-0.7, 2.5)	0.7 (-0.9, 2.3)
Bangladesh										
rural	138.5 (135.9, 141.1)	99.4 (91.6, 107.0)	130.3 (127.4, 133.0)	158.4 (156.0, 160.7)	163.7 (160.5, 166.9)	142.0 (140.1, 144.0)	104.8 (97.7, 111.6)	134.5 (132.6, 136.5)	160.9 (159.4, 162.4)	164.7 (163.2, 166.2)
urban	140.4 (137.7, 143.0)	101.7 (93.8, 109.2)	132.2 (129.3, 135.1)	160.1 (157.7, 162.4)	165.2 (162.1, 168.4)	143.6 (141.5, 145.6)	106.8 (99.6, 113.7)	136.2 (134.2, 138.2)	162.3 (160.8, 163.9)	165.8 (164.3, 167.4)
urban-rural difference	1.9 (0.6, 3.3)	2.3 (0.6, 4.1)	2.0 (0.6, 3.5)	1.7 (0.4, 3.0)	1.5 (0.1, 2.9)	1.6 (0.4, 2.7)	2.0 (0.3, 3.8)	1.7 (0.4, 3.0)	1.4 (0.4, 2.4)	1.2 (0.2, 2.1)
Bhutan										
rural	138.3 (134.1, 142.5)	102.1 (92.2, 111.4)	130.0 (125.6, 134.4)	157.0 (152.7, 161.2)	162.8 (157.3, 168.4)	140.9 (137.0, 144.7)	104.7 (93.7, 115.1)	132.6 (128.3, 137.0)	159.4 (157.0, 161.9)	165.1 (163.0, 167.2)
urban	140.2 (136.0, 144.3)	104.2 (94.2, 113.6)	131.9 (127.5, 136.2)	158.7 (154.6, 162.8)	164.4 (159.0, 169.9)	142.6 (138.7, 146.4)	106.8 (95.8, 117.3)	134.5 (130.1, 138.9)	161.1 (158.6, 163.6)	166.6 (164.5, 168.7)
urban-rural difference	1.8 (0.1, 3.6)	2.1 (0.1, 4.2)	1.9 (0.1, 3.7)	1.7 (-0.1, 3.5)	1.6 (-0.5, 3.5)	1.8 (0.1, 3.4)	2.0 (-0.2, 4.3)	1.9 (0.0, 3.6)	1.7 (0.1, 3.2)	1.5 (0.0, 3.0)
India										

Boys	Mean height in 1990 (cm)					Mean height in 2020 (cm)				
	Age-standardised	5 years	10 years	15 years	19 years	Age-standardised	5 years	10 years	15 years	19 years
rural	137.0 (136.5, 137.5)	101.4 (100.7, 102.0)	127.8 (127.1, 128.4)	155.7 (155.1, 156.3)	163.5 (162.8, 164.2)	141.6 (140.7, 142.6)	107.6 (105.3, 109.8)	133.2 (132.1, 134.3)	159.4 (158.6, 160.2)	166.1 (165.3, 166.9)
urban	139.2 (138.3, 140.1)	104.0 (102.9, 105.2)	130.1 (129.1, 131.1)	157.6 (156.7, 158.6)	165.1 (164.1, 166.2)	143.0 (142.0, 144.0)	109.4 (107.2, 111.6)	134.7 (133.6, 135.8)	160.5 (159.7, 161.4)	167.0 (166.1, 167.8)
urban-rural difference	2.2 (1.4, 3.0)	2.6 (1.7, 3.7)	2.3 (1.5, 3.2)	1.9 (1.2, 2.8)	1.7 (0.9, 2.5)	1.4 (0.7, 2.0)	1.8 (1.0, 2.7)	1.5 (0.8, 2.1)	1.1 (0.5, 1.7)	0.9 (0.2, 1.5)
Nepal										
rural	138.5 (135.1, 142.0)	102.0 (92.5, 111.2)	130.5 (127.0, 134.0)	157.3 (154.2, 160.4)	162.2 (157.8, 166.5)	140.9 (137.5, 144.3)	105.6 (95.2, 115.9)	133.3 (129.4, 137.2)	159.0 (157.3, 160.7)	163.1 (161.8, 164.6)
urban	141.2 (137.8, 144.7)	105.2 (95.6, 114.3)	133.4 (129.9, 136.8)	159.8 (156.8, 163.0)	164.4 (160.1, 168.7)	143.9 (140.5, 147.3)	109.1 (98.6, 119.1)	136.5 (132.7, 140.3)	161.9 (160.2, 163.6)	165.8 (164.4, 167.2)
urban-rural difference	2.7 (1.3, 4.0)	3.1 (1.4, 5.0)	2.8 (1.3, 4.3)	2.5 (1.0, 3.8)	2.3 (0.6, 3.7)	3.0 (1.8, 4.3)	3.5 (1.5, 5.6)	3.2 (1.8, 4.7)	2.8 (1.9, 3.9)	2.6 (1.7, 3.5)
Pakistan										
rural	141.2 (139.6, 143.0)	104.1 (102.2, 106.1)	132.4 (130.7, 134.2)	160.3 (158.5, 162.2)	167.3 (165.0, 169.6)	140.3 (138.5, 142.0)	106.0 (102.4, 109.5)	132.2 (130.2, 134.1)	158.0 (156.4, 159.5)	163.9 (162.3, 165.6)
urban	142.5 (140.8, 144.3)	105.4 (103.5, 107.3)	133.7 (132.0, 135.4)	161.6 (159.8, 163.5)	168.6 (166.3, 171.0)	142.1 (140.3, 143.8)	107.8 (104.2, 111.3)	134.0 (132.0, 135.9)	159.8 (158.2, 161.4)	165.8 (164.1, 167.4)
urban-rural difference	1.3 (0.2, 2.2)	1.3 (0.1, 2.4)	1.3 (0.2, 2.2)	1.3 (0.2, 2.3)	1.3 (-0.1, 2.5)	1.8 (0.9, 2.8)	1.8 (0.5, 3.2)	1.8 (0.8, 2.9)	1.8 (1.0, 2.7)	1.8 (0.9, 2.8)
Sri Lanka										
rural	139.4 (135.6, 143.1)	103.2 (93.7, 112.5)	131.0 (127.3, 134.8)	157.9 (154.1, 161.8)	164.2 (159.3, 169.1)	141.9 (138.3, 145.5)	105.1 (94.4, 115.5)	133.4 (129.2, 137.5)	160.6 (158.3, 162.9)	167.1 (164.9, 169.3)
urban	141.2 (137.2, 145.2)	105.4 (95.8, 114.8)	132.9 (128.9, 136.8)	159.7 (155.7, 163.7)	165.7 (160.7, 170.8)	143.5 (139.6, 147.4)	107.0 (96.4, 117.5)	135.1 (130.6, 139.5)	162.2 (159.5, 164.9)	168.5 (165.9, 171.2)
urban-rural difference	1.8 (-0.2, 3.9)	2.1 (-0.1, 4.4)	1.9 (-0.1, 4.0)	1.7 (-0.4, 3.8)	1.6 (-0.7, 3.8)	1.7 (-0.4, 3.6)	1.9 (-0.6, 4.4)	1.7 (-0.4, 3.8)	1.5 (-0.4, 3.4)	1.4 (-0.5, 3.3)
**Sub-Saharan Africa**										
Central Africa										
Angola										
rural	140.8 (135.1, 146.3)	106.8 (96.0, 117.6)	131.9 (126.4, 137.3)	158.3 (152.3, 164.0)	166.8 (159.6, 173.9)	142.1 (136.6, 147.5)	109.6 (97.4, 121.8)	133.5 (127.5, 139.6)	158.9 (154.5, 163.1)	166.8 (162.6, 170.9)
urban	143.0 (137.4, 148.4)	109.3 (98.4, 120.1)	134.2 (128.8, 139.5)	160.3 (154.5, 166.0)	168.7 (161.5, 175.9)	144.2 (138.9, 149.6)	112.1 (99.9, 124.2)	135.8 (129.9, 141.9)	160.9 (156.7, 165.1)	168.7 (164.5, 172.8)
urban-rural difference	2.2 (0.1, 4.4)	2.5 (0.2, 4.9)	2.3 (0.2, 4.4)	2.1 (-0.1, 4.3)	1.9 (-0.5, 4.3)	2.2 (0.1, 4.2)	2.5 (-0.2, 5.2)	2.2 (0.1, 4.5)	2.0 (0.2, 3.9)	1.9 (0.0, 3.7)
Central African Republic										
rural	142.4 (137.2, 147.7)	108.8 (97.8, 119.9)	133.4 (128.1, 138.9)	159.7 (154.2, 165.2)	168.7 (162.2, 175.1)	143.5 (137.6, 149.5)	111.0 (98.5, 123.7)	134.8 (128.2, 141.6)	160.3 (155.5, 165.3)	168.9 (164.8, 173.1)
urban	144.7 (139.5, 150.0)	111.4 (100.4, 122.4)	135.8 (130.4, 141.3)	161.8 (156.5, 167.5)	170.7 (164.2, 177.0)	145.7 (140.0, 151.6)	113.5 (101.1, 125.9)	137.1 (130.6, 143.6)	162.4 (157.5, 167.4)	170.9 (166.7, 175.0)
urban-rural difference	2.3 (0.1, 4.5)	2.5 (0.2, 5.0)	2.4 (0.2, 4.6)	2.2 (-0.1, 4.5)	2.0 (-0.6, 4.6)	2.2 (0.0, 4.5)	2.5 (-0.3, 5.3)	2.3 (0.0, 4.7)	2.1 (0.1, 4.3)	2.0 (-0.1, 4.1)
Congo										
rural	140.8 (137.6, 143.8)	106.2 (102.8, 109.5)	131.4 (128.1, 134.5)	158.5 (155.1, 161.8)	168.8 (165.1, 172.4)	141.9 (137.1, 146.7)	109.0 (101.7, 116.5)	132.7 (127.4, 138.2)	158.9 (154.3, 163.4)	168.6 (163.6, 173.6)
urban	143.0 (140.7, 145.3)	108.6 (106.1, 111.0)	133.6 (131.2, 136.0)	160.6 (158.1, 163.1)	170.8 (168.3, 173.4)	144.0 (139.5, 148.7)	111.3 (104.3, 118.6)	134.9 (129.7, 140.2)	160.9 (156.7, 165.3)	170.6 (165.7, 175.4)
urban-rural difference	2.2 (0.1, 4.4)	2.4 (0.1, 4.8)	2.2 (0.1, 4.4)	2.1 (-0.1, 4.4)	2.0 (-0.5, 4.6)	2.2 (0.0, 4.4)	2.4 (-0.5, 5.2)	2.2 (-0.1, 4.6)	2.1 (0.0, 4.1)	2.0 (-0.1, 4.0)
DR Congo										
rural	140.8 (136.4, 145.1)	108.6 (98.9, 118.3)	132.2 (127.9, 136.4)	157.5 (152.8, 162.0)	165.8 (160.3, 171.2)	142.0 (137.5, 146.7)	111.3 (99.8, 122.4)	133.8 (128.6, 139.0)	158.1 (154.5, 161.8)	165.8 (162.5, 169.1)
urban	143.5 (139.5, 147.5)	111.6 (102.1, 121.2)	135.0 (131.0, 138.8)	160.1 (155.7, 164.4)	168.3 (163.0, 173.5)	144.7 (140.3, 149.2)	114.2 (102.8, 125.1)	136.5 (131.4, 141.6)	160.7 (157.3, 164.1)	168.3 (165.1, 171.3)
urban-rural difference	2.7 (0.7, 4.7)	3.0 (0.7, 5.2)	2.8 (0.8, 4.8)	2.6 (0.6, 4.7)	2.5 (0.2, 4.8)	2.7 (0.6, 4.7)	2.9 (0.2, 5.7)	2.7 (0.5, 5.0)	2.6 (0.7, 4.5)	2.4 (0.7, 4.2)
Equatorial Guinea										
rural	141.9 (134.6, 149.4)	108.2 (96.2, 120.5)	132.9 (125.7, 140.3)	159.2 (152.0, 166.7)	168.1 (159.1, 177.2)	143.0 (135.6, 150.5)	110.6 (97.5, 123.8)	134.3 (126.5, 142.1)	159.8 (153.0, 166.6)	168.2 (160.6, 176.1)
urban	144.1 (137.0, 151.4)	110.8 (98.5, 122.9)	135.3 (128.1, 142.5)	161.4 (154.1, 168.9)	170.2 (161.4, 179.2)	145.2 (137.8, 152.8)	113.1 (100.0, 126.3)	136.6 (128.9, 144.6)	161.9 (155.0, 168.9)	170.2 (162.6, 178.0)
urban-rural difference	2.3 (0.0, 4.6)	2.5 (0.1, 5.0)	2.3 (0.1, 4.7)	2.2 (-0.2, 4.6)	2.0 (-0.6, 4.7)	2.2 (0.0, 4.5)	2.5 (-0.4, 5.4)	2.3 (-0.1, 4.7)	2.1 (0.0, 4.2)	2.0 (-0.1, 4.1)
Gabon										
rural	143.5 (138.1, 149.1)	109.7 (98.7, 120.4)	134.7 (129.3, 140.2)	161.0 (155.2, 166.9)	169.5 (162.7, 176.5)	144.4 (138.6, 150.4)	112.3 (99.5, 124.7)	136.0 (129.4, 142.4)	161.2 (156.5, 166.1)	169.0 (164.3, 173.9)
urban	145.9 (140.5, 151.2)	112.3 (101.4, 122.9)	137.1 (131.8, 142.5)	163.2 (157.8, 168.8)	171.6 (165.1, 178.2)	146.8 (140.9, 152.6)	114.8 (102.1, 127.1)	138.3 (132.0, 144.8)	163.4 (158.8, 168.2)	171.1 (166.7, 175.7)
urban-rural difference	2.4 (0.1, 4.6)	2.6 (0.2, 5.1)	2.4 (0.2, 4.7)	2.2 (-0.1, 4.6)	2.1 (-0.5, 4.7)	2.3 (0.0, 4.5)	2.6 (-0.4, 5.5)	2.4 (-0.1, 4.8)	2.2 (0.1, 4.2)	2.0 (0.0, 4.1)
East Africa										
Burundi										
rural	142.9 (136.1, 149.6)	108.9 (97.8, 119.5)	133.9 (127.1, 140.6)	160.9 (154.2, 167.6)	168.2 (159.8, 176.8)	144.2 (137.3, 151.0)	111.7 (99.4, 123.5)	135.5 (128.3, 142.5)	161.6 (155.0, 167.8)	168.6 (161.4, 175.8)
urban	144.7 (137.9, 151.4)	111.2 (100.1, 121.9)	135.9 (129.2, 142.5)	162.5 (155.8, 169.4)	169.6 (161.3, 178.3)	146.8 (139.9, 153.5)	114.7 (102.5, 126.4)	138.2 (131.1, 145.2)	164.0 (157.5, 170.1)	170.7 (163.6, 177.9)
urban-rural difference	1.9 (-0.2, 4.0)	2.3 (0.0, 4.7)	2.0 (-0.1, 4.1)	1.7 (-0.5, 3.8)	1.4 (-0.9, 3.7)	2.6 (0.6, 4.6)	3.0 (0.5, 5.6)	2.7 (0.6, 4.9)	2.4 (0.5, 4.3)	2.1 (0.2, 4.0)
Comoros										
rural	141.9 (137.4, 146.4)	107.9 (98.6, 117.3)	133.0 (128.3, 137.7)	159.9 (155.2, 164.7)	167.1 (161.1, 173.1)	143.3 (138.3, 148.2)	110.9 (100.1, 122.0)	134.6 (129.0, 140.2)	160.7 (156.4, 165.0)	167.4 (163.6, 171.1)
urban	143.8 (139.0, 148.4)	110.2 (100.8, 119.7)	135.0 (130.1, 139.7)	161.6 (156.7, 166.6)	168.6 (162.4, 174.6)	145.9 (140.8, 151.0)	113.9 (103.0, 124.9)	137.4 (131.6, 143.1)	163.0 (158.7, 167.6)	169.6 (165.6, 173.5)
urban-rural difference	1.9 (-0.1, 3.9)	2.3 (0.1, 4.6)	2.0 (0.0, 4.1)	1.7 (-0.4, 3.8)	1.4 (-0.8, 3.7)	2.6 (0.6, 4.6)	3.0 (0.5, 5.6)	2.7 (0.6, 4.9)	2.4 (0.5, 4.3)	2.1 (0.2, 4.1)
Djibouti										

Boys	Mean height in 1990 (cm)					Mean height in 2020 (cm)				
	Age-standardised	5 years	10 years	15 years	19 years	Age-standardised	5 years	10 years	15 years	19 years
rural	142.9 (136.1, 149.7)	109.0 (98.6, 119.8)	133.9 (127.0, 140.7)	160.9 (154.1, 167.6)	168.3 (160.1, 176.7)	144.3 (137.4, 151.1)	111.8 (100.3, 123.3)	135.5 (128.3, 142.6)	161.6 (155.2, 168.1)	168.7 (161.6, 175.9)
urban	144.8 (137.9, 151.7)	111.3 (100.8, 122.3)	135.9 (129.1, 142.7)	162.6 (155.7, 169.5)	169.7 (161.3, 178.2)	146.8 (140.1, 153.7)	114.8 (103.5, 126.3)	138.2 (131.2, 145.2)	164.0 (157.6, 170.6)	170.8 (163.6, 178.0)
urban-rural difference	1.9 (-0.1, 3.9)	2.3 (0.1, 4.7)	2.0 (-0.1, 4.1)	1.7 (-0.4, 3.7)	1.4 (-0.9, 3.7)	2.6 (0.6, 4.6)	3.0 (0.6, 5.6)	2.7 (0.6, 4.9)	2.4 (0.6, 4.3)	2.1 (0.2, 4.0)
Eritrea										
rural	144.0 (140.2, 147.9)	110.7 (101.8, 120.2)	135.3 (131.4, 139.4)	161.7 (157.8, 165.6)	168.3 (163.5, 173.0)	145.4 (140.9, 150.0)	112.4 (101.8, 123.4)	136.6 (131.3, 141.9)	163.1 (159.5, 166.7)	170.0 (166.6, 173.3)
urban	145.8 (141.9, 149.9)	113.0 (103.9, 122.5)	137.2 (133.2, 141.4)	163.4 (159.3, 167.4)	169.7 (164.8, 174.5)	148.0 (143.2, 152.6)	115.3 (104.6, 126.3)	139.3 (133.8, 144.4)	165.4 (161.7, 169.0)	172.1 (168.6, 175.6)
urban-rural difference	1.8 (-0.3, 3.9)	2.3 (0.0, 4.6)	1.9 (-0.1, 4.0)	1.6 (-0.5, 3.7)	1.4 (-0.9, 3.6)	2.5 (0.5, 4.5)	3.0 (0.4, 5.5)	2.7 (0.5, 4.8)	2.3 (0.4, 4.2)	2.1 (0.1, 4.0)
Ethiopia										
rural	141.4 (137.6, 145.0)	106.7 (97.6, 116.1)	131.8 (128.0, 135.5)	159.7 (155.8, 163.5)	169.1 (163.7, 174.4)	142.6 (139.0, 146.3)	110.4 (100.1, 121.3)	133.4 (129.3, 137.7)	159.7 (157.6, 161.9)	168.2 (166.3, 170.1)
urban	143.5 (139.8, 147.2)	109.4 (100.2, 118.9)	134.0 (130.3, 137.8)	161.5 (157.8, 165.2)	170.5 (165.3, 175.9)	145.6 (141.9, 149.3)	114.0 (103.8, 124.8)	136.6 (132.4, 140.9)	162.4 (160.2, 164.6)	170.6 (168.6, 172.5)
urban-rural difference	2.1 (0.5, 3.6)	2.7 (0.8, 4.8)	2.2 (0.6, 3.9)	1.8 (0.2, 3.3)	1.4 (-0.3, 3.0)	3.0 (1.7, 4.4)	3.6 (1.6, 5.9)	3.2 (1.6, 4.8)	2.7 (1.6, 3.9)	2.4 (1.3, 3.4)
Kenya										
rural	144.5 (140.3, 148.7)	112.1 (103.9, 120.4)	135.4 (131.2, 139.5)	162.0 (157.1, 166.9)	169.7 (163.2, 176.1)	146.3 (142.3, 150.2)	115.6 (106.3, 125.5)	137.5 (132.8, 142.1)	163.0 (159.7, 166.3)	170.1 (167.4, 172.7)
urban	146.2 (142.0, 150.4)	114.3 (106.3, 122.4)	137.2 (133.1, 141.3)	163.4 (158.4, 168.4)	170.8 (164.3, 177.2)	148.7 (144.9, 152.5)	118.6 (109.3, 128.1)	140.1 (135.6, 144.6)	165.2 (162.1, 168.4)	172.0 (169.4, 174.5)
urban-rural difference	1.6 (-0.2, 3.4)	2.2 (0.0, 4.4)	1.8 (-0.1, 3.7)	1.4 (-0.4, 3.2)	1.1 (-1.0, 3.1)	2.4 (0.6, 4.2)	3.0 (0.6, 5.5)	2.6 (0.7, 4.5)	2.2 (0.6, 3.8)	1.9 (0.3, 3.5)
Madagascar										
rural	140.1 (135.5, 144.7)	106.5 (96.8, 116.4)	131.4 (126.4, 136.3)	158.0 (153.6, 162.6)	164.6 (159.3, 170.0)	141.8 (136.5, 147.1)	109.3 (98.5, 120.5)	133.2 (127.5, 139.1)	159.2 (154.5, 164.0)	165.5 (160.8, 170.4)
urban	142.0 (137.2, 146.8)	108.8 (99.0, 118.7)	133.4 (128.2, 138.3)	159.7 (155.0, 164.4)	166.0 (160.6, 171.5)	144.4 (139.0, 149.8)	112.4 (101.6, 123.4)	135.9 (130.0, 141.9)	161.5 (156.7, 166.3)	167.6 (162.8, 172.6)
urban-rural difference	1.9 (-0.2, 4.0)	2.3 (0.1, 4.7)	2.0 (0.0, 4.2)	1.7 (-0.4, 3.8)	1.4 (-0.8, 3.7)	2.6 (0.6, 4.6)	3.0 (0.5, 5.6)	2.7 (0.6, 4.9)	2.4 (0.5, 4.3)	2.1 (0.3, 4.0)
Malawi										
rural	140.4 (136.2, 144.7)	106.5 (97.5, 116.1)	131.6 (127.2, 136.0)	158.4 (154.0, 162.6)	164.8 (159.2, 170.2)	142.1 (137.9, 146.3)	109.6 (99.2, 120.3)	133.6 (128.6, 138.5)	159.5 (156.3, 162.6)	165.5 (163.4, 167.6)
urban	142.0 (137.8, 146.3)	108.7 (99.3, 118.3)	133.4 (129.0, 137.9)	159.8 (155.3, 164.0)	165.9 (160.2, 171.3)	144.3 (140.0, 148.6)	112.4 (101.8, 123.1)	136.0 (130.9, 141.1)	161.5 (158.2, 164.7)	167.2 (165.1, 169.3)
urban-rural difference	1.6 (-0.1, 3.3)	2.1 (0.1, 4.3)	1.8 (0.0, 3.5)	1.4 (-0.3, 3.1)	1.1 (-0.7, 2.9)	2.3 (0.6, 3.9)	2.8 (0.4, 5.1)	2.4 (0.6, 4.3)	2.0 (0.5, 3.5)	1.7 (0.3, 3.1)
Mauritius										
rural	148.9 (145.8, 152.0)	117.1 (108.7, 126.2)	141.0 (137.7, 144.4)	166.1 (163.3, 168.7)	170.4 (167.9, 172.9)	151.8 (148.9, 154.8)	120.0 (110.9, 129.3)	143.7 (140.5, 147.0)	169.0 (167.1, 170.9)	173.7 (171.7, 175.9)
urban	150.6 (147.5, 153.7)	119.3 (110.8, 128.5)	142.8 (139.4, 146.3)	167.6 (164.8, 170.3)	171.7 (169.2, 174.3)	154.1 (151.0, 157.2)	122.7 (113.5, 132.2)	146.1 (142.7, 149.6)	171.1 (169.1, 173.1)	175.6 (173.5, 177.9)
urban-rural difference	1.7 (0.1, 3.3)	2.1 (0.1, 4.3)	1.8 (0.1, 3.6)	1.5 (-0.1, 3.1)	1.3 (-0.4, 3.0)	2.3 (0.5, 4.0)	2.7 (0.2, 5.1)	2.4 (0.4, 4.3)	2.1 (0.5, 3.7)	1.9 (0.3, 3.4)
Mozambique										
rural	142.1 (139.0, 145.1)	108.9 (102.2, 115.5)	133.8 (130.8, 137.0)	159.8 (156.5, 163.1)	165.0 (160.8, 169.4)	142.8 (139.9, 145.6)	112.0 (104.7, 119.5)	135.0 (131.6, 138.3)	159.4 (157.2, 161.7)	163.9 (161.5, 166.4)
urban	144.9 (141.9, 147.8)	112.2 (105.8, 118.8)	136.8 (133.8, 139.8)	162.4 (159.2, 165.6)	167.3 (163.1, 171.6)	146.3 (143.4, 149.1)	116.1 (108.8, 123.4)	138.7 (135.3, 142.0)	162.7 (160.4, 164.9)	166.8 (164.4, 169.3)
urban-rural difference	2.8 (1.2, 4.5)	3.4 (1.4, 5.5)	3.0 (1.3, 4.7)	2.6 (1.0, 4.3)	2.3 (0.5, 4.2)	3.5 (2.0, 5.1)	4.1 (1.9, 6.3)	3.7 (2.0, 5.4)	3.2 (1.9, 4.6)	2.9 (1.6, 4.3)
Rwanda										
rural	137.9 (134.0, 141.9)	103.0 (93.8, 112.9)	127.9 (124.0, 131.7)	156.2 (152.2, 160.3)	166.9 (161.3, 172.6)	138.2 (134.1, 142.4)	106.4 (95.7, 117.6)	128.7 (124.1, 133.4)	155.0 (152.6, 157.5)	164.7 (162.7, 166.8)
urban	141.1 (137.1, 145.1)	106.8 (97.5, 116.8)	131.2 (127.2, 135.2)	159.1 (154.9, 163.2)	169.5 (163.8, 175.2)	142.3 (138.3, 146.6)	111.2 (100.3, 122.4)	133.1 (128.4, 137.8)	159.0 (156.5, 161.5)	168.4 (166.2, 170.6)
urban-rural difference	3.2 (1.5, 4.7)	3.7 (1.7, 5.8)	3.3 (1.6, 5.0)	2.9 (1.3, 4.5)	2.6 (0.8, 4.3)	4.2 (2.7, 5.7)	4.7 (2.6, 7.1)	4.3 (2.7, 6.1)	4.0 (2.7, 5.3)	3.6 (2.4, 4.8)
Seychelles										
rural	148.9 (147.1, 150.7)	110.7 (109.4, 112.0)	140.4 (138.9, 142.0)	168.7 (166.2, 171.3)	173.9 (170.0, 177.7)	151.1 (150.2, 152.0)	114.8 (113.0, 116.6)	142.8 (141.9, 143.6)	170.0 (169.1, 170.8)	175.2 (173.6, 177.0)
urban	148.4 (146.6, 150.2)	110.0 (108.7, 111.4)	139.8 (138.3, 141.4)	168.2 (165.7, 170.8)	173.5 (169.6, 177.3)	151.2 (150.3, 152.1)	114.7 (112.9, 116.6)	142.8 (141.9, 143.8)	170.2 (169.3, 171.0)	175.4 (173.8, 177.3)
urban-rural difference	-0.6 (-1.4, 0.3)	-0.7 (-1.6, 0.1)	-0.6 (-1.4, 0.2)	-0.5 (-1.4, 0.4)	-0.4 (-1.4, 0.6)	0.1 (-0.5, 0.7)	0.0 (-0.7, 0.7)	0.1 (-0.5, 0.6)	0.2 (-0.5, 0.8)	0.2 (-0.5, 1.0)
Somalia										
rural	144.0 (138.5, 149.6)	110.1 (100.0, 120.2)	135.0 (129.4, 140.6)	162.0 (156.3, 167.8)	169.5 (162.4, 176.6)	145.4 (139.9, 151.0)	112.6 (101.3, 123.8)	136.5 (130.4, 142.6)	162.8 (158.2, 167.6)	170.3 (165.6, 175.0)
urban	145.9 (140.5, 151.4)	112.5 (102.5, 122.5)	137.0 (131.5, 142.4)	163.7 (158.0, 169.3)	171.0 (164.0, 178.0)	148.0 (142.6, 153.6)	115.6 (104.4, 127.0)	139.2 (133.2, 145.4)	165.2 (160.6, 170.0)	172.4 (168.1, 176.9)
urban-rural difference	1.9 (-0.1, 3.9)	2.4 (0.1, 4.7)	2.0 (0.0, 4.1)	1.7 (-0.3, 3.8)	1.4 (-0.8, 3.7)	2.6 (0.6, 4.6)	3.1 (0.5, 5.6)	2.7 (0.6, 4.9)	2.4 (0.5, 4.3)	2.2 (0.2, 4.0)
South Sudan										
rural	145.2 (139.7, 150.8)	111.6 (101.6, 121.8)	136.1 (130.6, 141.7)	163.0 (157.2, 168.9)	170.8 (163.7, 177.9)	146.5 (141.0, 151.8)	113.6 (102.4, 124.9)	137.5 (131.4, 143.5)	164.0 (159.2, 168.7)	171.9 (167.5, 176.3)
urban	147.2 (141.8, 152.7)	114.0 (104.2, 124.5)	138.2 (132.8, 143.6)	164.8 (159.2, 170.5)	172.3 (165.4, 179.3)	149.2 (143.7, 154.5)	116.7 (105.5, 128.0)	140.2 (134.2, 146.3)	166.5 (161.9, 171.0)	174.1 (170.0, 178.2)
urban-rural difference	2.0 (0.0, 3.9)	2.4 (0.2, 4.8)	2.1 (0.1, 4.1)	1.8 (-0.2, 3.8)	1.5 (-0.7, 3.8)	2.7 (0.7, 4.7)	3.1 (0.6, 5.7)	2.8 (0.7, 4.9)	2.5 (0.6, 4.4)	2.2 (0.3, 4.2)
Sudan										
rural	145.2 (140.9, 149.3)	110.9 (101.8, 120.5)	135.9 (131.2, 140.4)	163.3 (159.0, 167.4)	171.7 (167.1, 176.0)	145.9 (141.5, 150.4)	113.6 (103.2, 124.3)	137.0 (131.7, 142.3)	163.1 (159.7, 166.6)	171.0 (168.5, 173.5)
urban	147.0 (142.7, 151.2)	113.2 (103.8, 122.8)	137.8 (133.1, 142.4)	164.9 (160.7, 169.1)	173.0 (168.3, 177.6)	148.4 (143.9, 152.8)	116.5 (105.9, 127.4)	139.6 (134.3, 144.7)	165.4 (161.9, 168.9)	173.0 (170.5, 175.5)
urban-rural difference	1.8 (0.0, 3.7)	2.3 (0.2, 4.5)	1.9 (0.1, 3.8)	1.6 (-0.2, 3.5)	1.3 (-0.7, 3.4)	2.5 (0.7, 4.3)	3.0 (0.5, 5.4)	2.6 (0.7, 4.6)	2.3 (0.7, 4.0)	2.0 (0.5, 3.6)

Boys	Mean height in 1990 (cm)					Mean height in 2020 (cm)				
	Age-standardised	5 years	10 years	15 years	19 years	Age-standardised	5 years	10 years	15 years	19 years
Tanzania										
rural	139.5 (136.4, 142.7)	107.4 (101.2, 113.7)	130.6 (127.2, 134.0)	156.7 (153.0, 160.3)	164.4 (160.3, 168.6)	141.1 (137.6, 144.5)	109.5 (102.3, 116.8)	131.9 (127.9, 135.9)	158.1 (154.8, 161.4)	166.2 (163.1, 169.4)
urban	141.2 (138.0, 144.4)	109.7 (103.3, 116.2)	132.4 (129.0, 136.0)	158.2 (154.7, 161.7)	165.6 (161.6, 169.4)	143.6 (139.9, 147.2)	112.6 (105.3, 120.2)	134.6 (130.3, 138.9)	160.4 (156.8, 163.9)	168.2 (164.8, 171.5)
urban-rural difference	1.7 (-0.3, 3.7)	2.3 (0.1, 4.6)	1.9 (-0.1, 3.9)	1.5 (-0.5, 3.5)	1.2 (-1.0, 3.5)	2.5 (0.6, 4.5)	3.1 (0.5, 5.5)	2.7 (0.6, 4.7)	2.3 (0.4, 4.2)	2.0 (0.1, 3.9)
Uganda										
rural	139.7 (137.0, 142.3)	102.9 (99.3, 106.4)	129.7 (127.0, 132.2)	158.9 (155.5, 162.2)	168.6 (163.9, 173.3)	140.4 (138.1, 142.7)	106.1 (101.8, 110.3)	130.9 (128.0, 133.8)	158.6 (156.6, 160.5)	167.5 (165.8, 169.2)
urban	141.5 (138.8, 144.2)	105.7 (101.9, 109.7)	131.8 (129.0, 134.5)	160.3 (157.0, 163.7)	169.5 (164.9, 174.2)	143.4 (140.9, 145.9)	109.9 (105.3, 114.5)	134.1 (131.0, 137.4)	161.1 (159.0, 163.2)	169.5 (167.7, 171.2)
urban-rural difference	1.9 (0.3, 3.3)	2.8 (0.9, 5.0)	2.1 (0.6, 3.8)	1.4 (0.0, 2.8)	0.9 (-0.7, 2.4)	2.9 (1.6, 4.4)	3.9 (1.8, 6.2)	3.2 (1.7, 4.9)	2.5 (1.4, 3.7)	2.0 (1.0, 3.0)
Zambia										
rural	141.7 (137.1, 146.4)	107.5 (98.1, 117.2)	132.7 (127.8, 137.6)	159.8 (155.1, 164.7)	167.3 (161.4, 173.1)	142.7 (138.4, 147.2)	110.6 (99.9, 121.5)	134.1 (129.0, 139.4)	159.9 (156.5, 163.3)	166.8 (164.2, 169.3)
urban	143.8 (139.3, 148.3)	110.1 (100.6, 119.8)	134.9 (130.2, 139.6)	161.7 (157.0, 166.4)	169.0 (163.3, 174.6)	145.6 (141.1, 150.0)	113.9 (103.1, 124.9)	137.0 (131.9, 142.2)	162.5 (159.2, 165.9)	169.2 (166.8, 171.7)
urban-rural difference	2.1 (0.3, 3.9)	2.5 (0.4, 4.6)	2.2 (0.3, 4.0)	1.9 (0.0, 3.8)	1.7 (-0.4, 3.7)	2.8 (1.1, 4.5)	3.2 (0.9, 5.6)	2.9 (1.1, 4.8)	2.6 (1.1, 4.2)	2.4 (0.9, 3.9)
Southern Africa										
Botswana										
rural	146.6 (142.4, 150.8)	111.5 (101.6, 121.7)	137.6 (133.2, 141.8)	164.5 (160.3, 168.7)	173.1 (167.8, 178.4)	145.8 (141.2, 150.1)	112.3 (101.1, 123.7)	137.1 (131.9, 142.1)	163.0 (159.7, 166.1)	170.9 (168.0, 173.8)
urban	148.8 (144.8, 152.9)	114.0 (104.2, 124.0)	139.8 (135.6, 144.2)	166.6 (162.5, 170.6)	175.0 (169.8, 180.3)	148.0 (143.6, 152.3)	114.8 (103.5, 126.1)	139.4 (134.3, 144.4)	165.1 (162.0, 168.2)	172.8 (170.1, 175.7)
urban-rural difference	2.2 (0.1, 4.3)	2.5 (0.2, 4.8)	2.3 (0.2, 4.4)	2.1 (-0.1, 4.2)	1.9 (-0.5, 4.2)	2.2 (0.3, 4.3)	2.5 (0.1, 5.1)	2.3 (0.3, 4.5)	2.1 (0.3, 4.1)	2.0 (0.1, 3.9)
Lesotho										
rural	142.3 (138.1, 146.5)	107.5 (97.6, 117.9)	133.3 (129.2, 137.3)	160.0 (155.6, 164.4)	168.5 (162.7, 174.4)	141.8 (137.5, 146.2)	108.2 (96.9, 119.9)	133.0 (128.1, 138.0)	159.1 (156.4, 161.7)	167.1 (164.8, 169.4)
urban	144.5 (140.1, 148.7)	110.2 (100.2, 120.7)	135.6 (131.4, 139.7)	162.1 (157.7, 166.3)	170.3 (164.4, 176.2)	144.2 (139.8, 148.5)	111.0 (99.7, 122.4)	135.5 (130.4, 140.4)	161.3 (158.5, 164.0)	169.1 (166.6, 171.4)
urban-rural difference	2.2 (0.5, 3.8)	2.6 (0.6, 4.7)	2.3 (0.6, 4.0)	2.0 (0.4, 3.6)	1.8 (-0.1, 3.5)	2.4 (0.9, 3.9)	2.8 (0.6, 5.1)	2.5 (0.8, 4.2)	2.2 (0.9, 3.5)	2.0 (0.8, 3.2)
Namibia										
rural	143.5 (139.5, 147.5)	108.8 (98.7, 119.2)	134.2 (130.2, 138.5)	161.2 (157.5, 165.0)	170.6 (166.0, 175.3)	142.9 (138.2, 147.6)	110.0 (98.7, 121.4)	134.0 (128.8, 139.2)	159.8 (156.1, 163.5)	168.3 (164.5, 172.2)
urban	145.6 (141.6, 149.7)	111.2 (101.1, 121.5)	136.4 (132.3, 140.6)	163.2 (159.4, 167.1)	172.4 (167.7, 177.2)	145.0 (140.4, 149.6)	112.4 (101.3, 123.7)	136.2 (131.1, 141.3)	161.8 (158.2, 165.5)	170.2 (166.5, 174.0)
urban-rural difference	2.1 (0.1, 4.2)	2.4 (0.1, 4.7)	2.2 (0.1, 4.3)	2.0 (-0.1, 4.1)	1.9 (-0.4, 4.2)	2.2 (0.1, 4.1)	2.4 (-0.2, 4.9)	2.2 (0.1, 4.3)	2.0 (0.1, 3.9)	1.9 (0.0, 3.8)
South Africa										
rural	143.1 (141.7, 144.6)	109.9 (108.3, 111.5)	134.7 (133.3, 136.2)	160.3 (158.5, 161.9)	167.4 (165.1, 169.6)	142.2 (141.0, 143.4)	107.2 (105.2, 109.3)	133.2 (131.9, 134.5)	160.1 (159.0, 161.3)	168.3 (167.0, 169.6)
urban	145.1 (143.6, 146.7)	111.5 (109.7, 113.3)	136.6 (135.0, 138.3)	162.4 (160.6, 164.2)	169.7 (167.4, 171.9)	143.7 (142.6, 144.9)	108.3 (106.3, 110.3)	134.6 (133.3, 135.8)	161.8 (160.7, 162.9)	170.2 (168.9, 171.4)
urban-rural difference	2.0 (0.9, 3.1)	1.6 (0.3, 2.9)	1.9 (0.7, 3.0)	2.1 (1.0, 3.3)	2.4 (1.2, 3.5)	1.5 (0.8, 2.2)	1.1 (0.2, 2.0)	1.4 (0.7, 2.1)	1.6 (1.0, 2.4)	1.9 (1.1, 2.7)
Eswatini										
rural	144.5 (140.4, 148.7)	109.8 (99.4, 120.3)	135.7 (131.6, 139.7)	162.3 (158.1, 166.5)	170.1 (164.9, 175.3)	144.1 (139.5, 148.8)	110.4 (98.5, 122.4)	135.5 (130.2, 140.7)	161.5 (158.5, 164.5)	168.8 (166.3, 171.4)
urban	147.1 (142.9, 151.5)	112.7 (102.3, 123.2)	138.4 (134.3, 142.6)	164.8 (160.5, 168.9)	172.4 (167.0, 177.6)	146.9 (142.3, 151.4)	113.4 (101.7, 125.4)	138.3 (133.1, 143.6)	164.1 (161.0, 167.2)	171.3 (168.7, 174.0)
urban-rural difference	2.6 (1.0, 4.3)	2.9 (0.9, 5.0)	2.7 (1.0, 4.4)	2.5 (0.8, 4.1)	2.3 (0.5, 4.1)	2.7 (1.1, 4.5)	3.1 (0.7, 5.6)	2.8 (1.0, 4.8)	2.6 (1.1, 4.2)	2.4 (1.0, 3.9)
Zimbabwe										
rural	144.2 (140.0, 148.6)	109.0 (99.0, 119.3)	134.9 (130.9, 139.0)	162.1 (157.8, 166.6)	171.5 (165.4, 177.7)	143.2 (139.1, 147.5)	109.4 (98.1, 120.7)	134.1 (129.4, 138.9)	160.6 (158.1, 163.0)	169.5 (167.1, 171.7)
urban	146.5 (142.3, 150.8)	111.8 (101.9, 122.1)	137.4 (133.2, 141.5)	164.2 (159.9, 168.7)	173.3 (167.3, 179.6)	145.9 (141.7, 150.1)	112.5 (101.2, 124.0)	137.0 (132.3, 141.7)	163.1 (160.5, 165.5)	171.7 (169.4, 174.0)
urban-rural difference	2.3 (0.7, 3.9)	2.8 (0.8, 4.9)	2.5 (0.8, 4.1)	2.1 (0.4, 3.6)	1.8 (0.0, 3.5)	2.7 (1.3, 4.1)	3.2 (1.0, 5.5)	2.8 (1.3, 4.5)	2.5 (1.3, 3.7)	2.2 (1.1, 3.3)
West Africa										
Benin										
rural	143.2 (138.9, 147.6)	106.9 (97.2, 116.5)	134.2 (129.6, 138.8)	161.5 (157.4, 165.8)	170.5 (165.4, 175.9)	142.9 (138.0, 147.5)	109.4 (98.1, 120.2)	134.6 (129.0, 140.2)	160.0 (156.2, 163.6)	167.5 (164.8, 170.2)
urban	145.2 (141.0, 149.4)	109.3 (99.5, 118.8)	136.3 (131.7, 140.8)	163.3 (159.2, 167.6)	172.0 (167.0, 177.3)	144.8 (139.9, 149.6)	111.7 (100.5, 122.5)	136.7 (131.0, 142.2)	161.7 (158.0, 165.4)	169.0 (166.4, 171.7)
urban-rural difference	1.9 (0.0, 3.9)	2.4 (0.1, 4.7)	2.1 (0.1, 4.0)	1.8 (-0.2, 3.7)	1.5 (-0.6, 3.6)	2.0 (0.1, 3.8)	2.4 (0.0, 5.0)	2.1 (0.2, 4.1)	1.8 (0.1, 3.4)	1.5 (-0.1, 3.2)
Burkina Faso										
rural	143.9 (140.3, 147.5)	105.1 (101.1, 109.0)	134.5 (130.8, 138.2)	162.9 (158.7, 167.0)	174.0 (169.4, 178.4)	143.6 (139.4, 147.9)	108.0 (101.1, 115.0)	135.1 (130.3, 140.2)	161.1 (157.3, 165.0)	170.4 (167.0, 173.7)
urban	146.1 (142.2, 149.9)	107.7 (103.3, 112.1)	136.8 (132.8, 140.8)	164.9 (160.6, 169.2)	175.8 (170.8, 180.7)	145.8 (141.4, 150.2)	110.7 (103.4, 117.9)	137.5 (132.4, 142.6)	163.1 (159.2, 167.2)	172.2 (168.7, 175.9)
urban-rural difference	2.2 (0.1, 4.2)	2.6 (0.3, 5.0)	2.3 (0.2, 4.4)	2.0 (-0.1, 4.1)	1.7 (-0.6, 4.0)	2.3 (0.3, 4.2)	2.7 (0.2, 5.2)	2.4 (0.3, 4.5)	2.1 (0.2, 3.9)	1.8 (-0.1, 3.7)
Cabo Verde										
rural	147.5 (143.3, 151.6)	112.2 (102.7, 121.8)	138.4 (133.8, 142.9)	165.2 (161.0, 169.4)	175.0 (169.7, 180.0)	147.9 (143.3, 152.4)	113.0 (102.2, 123.6)	139.0 (133.4, 144.2)	165.6 (162.1, 169.0)	175.1 (172.9, 177.5)
urban	149.4 (145.4, 153.6)	114.6 (105.2, 124.1)	140.5 (136.0, 145.0)	167.0 (162.9, 171.1)	176.5 (171.3, 181.4)	150.0 (145.4, 154.5)	115.4 (104.5, 126.2)	141.1 (135.6, 146.5)	167.4 (164.0, 170.9)	176.7 (174.5, 179.1)
urban-rural difference	2.0 (0.1, 3.8)	2.4 (0.2, 4.7)	2.1 (0.2, 4.0)	1.8 (0.0, 3.6)	1.5 (-0.5, 3.6)	2.0 (0.3, 3.8)	2.4 (0.0, 5.0)	2.1 (0.2, 4.1)	1.8 (0.2, 3.4)	1.6 (0.0, 3.1)
Cameroon										

Boys	Mean height in 1990 (cm)					Mean height in 2020 (cm)				
	Age-standardised	5 years	10 years	15 years	19 years	Age-standardised	5 years	10 years	15 years	19 years
rural	142.8 (139.0, 146.5)	107.2 (97.8, 116.7)	133.5 (129.5, 137.6)	160.7 (157.3, 164.2)	171.1 (167.7, 174.5)	143.5 (138.9, 148.0)	108.8 (98.1, 119.5)	134.6 (129.2, 139.7)	161.0 (157.5, 164.4)	170.7 (167.8, 173.4)
urban	145.5 (142.0, 149.2)	110.4 (100.9, 120.0)	136.4 (132.5, 140.3)	163.3 (160.0, 166.6)	173.4 (170.2, 176.7)	146.2 (141.7, 150.7)	111.9 (101.2, 122.7)	137.4 (132.2, 142.5)	163.5 (160.0, 167.0)	173.0 (170.2, 175.6)
urban-rural difference	2.7 (1.0, 4.5)	3.2 (1.1, 5.4)	2.9 (1.1, 4.7)	2.6 (0.9, 4.2)	2.3 (0.5, 4.1)	2.7 (0.9, 4.5)	3.1 (0.6, 5.7)	2.8 (0.9, 4.9)	2.5 (0.9, 4.2)	2.3 (0.8, 3.9)
Chad										
rural	145.0 (140.0, 149.9)	109.2 (99.3, 119.1)	135.9 (130.7, 141.0)	163.0 (157.9, 168.1)	172.8 (166.6, 179.1)	144.9 (139.4, 150.3)	110.7 (99.7, 121.4)	136.2 (130.1, 142.0)	162.2 (157.1, 167.0)	171.1 (166.2, 176.1)
urban	147.2 (142.3, 152.1)	111.7 (102.2, 121.7)	138.2 (133.2, 143.3)	165.0 (160.1, 170.0)	174.6 (168.6, 180.8)	147.1 (141.7, 152.5)	113.3 (102.4, 124.1)	138.6 (132.7, 144.5)	164.3 (159.4, 169.1)	173.0 (168.1, 177.9)
urban-rural difference	2.2 (0.1, 4.3)	2.6 (0.3, 5.0)	2.3 (0.2, 4.5)	2.1 (-0.1, 4.2)	1.8 (-0.5, 4.2)	2.3 (0.2, 4.3)	2.7 (0.0, 5.2)	2.4 (0.2, 4.5)	2.1 (0.2, 4.0)	1.9 (0.0, 3.8)
Côte d'Ivoire										
rural	143.6 (139.0, 148.0)	108.3 (98.5, 117.9)	135.1 (130.4, 139.9)	161.5 (156.9, 166.1)	169.0 (163.9, 174.2)	143.9 (138.8, 149.0)	109.6 (98.6, 120.4)	135.7 (129.9, 141.4)	161.4 (157.2, 165.9)	168.4 (164.2, 172.7)
urban	146.0 (141.5, 150.7)	111.2 (101.5, 120.6)	137.7 (133.1, 142.5)	163.8 (159.3, 168.4)	171.0 (165.8, 176.2)	146.4 (141.2, 151.7)	112.5 (101.5, 123.3)	138.4 (132.6, 144.2)	163.8 (159.5, 168.2)	170.5 (166.2, 174.8)
urban-rural difference	2.4 (0.6, 4.3)	2.8 (0.7, 5.1)	2.6 (0.7, 4.4)	2.3 (0.4, 4.1)	2.0 (0.0, 4.0)	2.5 (0.7, 4.4)	2.9 (0.5, 5.5)	2.6 (0.6, 4.7)	2.4 (0.6, 4.1)	2.1 (0.4, 3.8)
Gambia										
rural	141.6 (136.9, 146.2)	105.9 (96.0, 115.7)	132.7 (127.8, 137.6)	159.6 (154.6, 164.4)	168.6 (162.4, 174.7)	141.9 (136.6, 147.1)	107.9 (96.3, 119.2)	133.5 (127.6, 139.4)	159.1 (154.6, 163.7)	167.1 (162.8, 171.2)
urban	143.7 (138.9, 148.3)	108.4 (98.6, 118.1)	134.9 (130.0, 139.8)	161.6 (156.5, 166.4)	170.3 (164.3, 176.3)	144.1 (138.6, 149.5)	110.5 (98.7, 121.8)	135.8 (129.8, 141.9)	161.2 (156.6, 165.7)	168.9 (164.5, 173.1)
urban-rural difference	2.1 (0.0, 4.2)	2.5 (0.2, 4.9)	2.2 (0.1, 4.3)	2.0 (-0.2, 4.1)	1.7 (-0.6, 4.0)	2.2 (0.1, 4.2)	2.6 (0.0, 5.3)	2.3 (0.1, 4.4)	2.0 (0.1, 4.0)	1.8 (-0.1, 3.8)
Ghana										
rural	145.1 (142.1, 148.1)	109.6 (104.8, 114.4)	136.7 (133.8, 139.9)	163.2 (159.4, 166.9)	170.0 (164.7, 175.4)	144.1 (142.4, 145.7)	110.9 (108.2, 113.5)	136.2 (134.3, 138.2)	161.2 (159.6, 162.8)	167.2 (165.4, 168.9)
urban	146.7 (143.7, 149.8)	111.7 (106.9, 116.5)	138.5 (135.5, 141.6)	164.6 (161.0, 168.2)	171.2 (165.9, 176.6)	145.9 (144.2, 147.5)	113.1 (110.4, 115.9)	138.2 (136.1, 140.2)	162.8 (161.2, 164.4)	168.5 (166.8, 170.2)
urban-rural difference	1.6 (0.0, 3.3)	2.1 (0.3, 4.0)	1.8 (0.1, 3.4)	1.4 (-0.2, 3.1)	1.2 (-0.6, 2.9)	1.8 (0.8, 2.8)	2.3 (0.7, 3.9)	1.9 (0.8, 3.1)	1.6 (0.7, 2.5)	1.3 (0.4, 2.3)
Guinea										
rural	144.5 (139.5, 149.5)	107.9 (97.0, 118.6)	134.5 (127.6, 141.3)	161.4 (154.6, 168.3)	170.9 (162.4, 179.3)	144.4 (139.1, 149.5)	109.5 (97.9, 120.9)	135.0 (128.0, 142.1)	160.9 (154.6, 167.2)	169.5 (162.3, 176.5)
urban	146.6 (141.5, 151.6)	110.4 (99.3, 121.4)	136.8 (130.0, 143.6)	163.4 (156.7, 170.3)	172.6 (164.2, 181.0)	146.6 (141.4, 151.9)	112.1 (100.2, 123.5)	137.4 (130.4, 144.4)	162.9 (156.6, 169.3)	171.3 (164.1, 178.4)
urban-rural difference	2.1 (0.1, 4.3)	2.6 (0.4, 4.9)	2.3 (0.3, 4.4)	2.0 (-0.1, 4.2)	1.8 (-0.5, 4.1)	2.2 (0.3, 4.2)	2.6 (0.1, 5.2)	2.3 (0.3, 4.5)	2.1 (0.2, 4.0)	1.8 (-0.1, 3.7)
Guinea Bissau										
rural	143.5 (136.7, 150.3)	108.6 (98.7, 118.3)	135.5 (130.6, 140.5)	162.5 (157.4, 167.7)	171.7 (165.2, 178.2)	143.6 (136.9, 150.4)	110.2 (99.0, 121.1)	135.9 (130.2, 141.7)	161.7 (157.4, 165.9)	169.9 (165.7, 174.1)
urban	145.7 (138.9, 152.5)	111.1 (101.1, 120.8)	137.8 (132.7, 142.9)	164.5 (159.3, 169.7)	173.4 (166.8, 180.1)	145.8 (139.2, 152.6)	112.8 (101.3, 123.7)	138.3 (132.4, 144.2)	163.8 (159.5, 168.0)	171.8 (167.5, 176.1)
urban-rural difference	2.2 (0.2, 4.3)	2.5 (0.2, 4.9)	2.3 (0.1, 4.4)	2.0 (-0.2, 4.1)	1.8 (-0.6, 4.1)	2.2 (0.3, 4.2)	2.6 (0.1, 5.2)	2.3 (0.2, 4.5)	2.0 (0.2, 3.9)	1.8 (0.0, 3.7)
Liberia										
rural	138.5 (134.2, 142.9)	102.9 (93.4, 112.4)	129.4 (125.2, 133.6)	156.5 (152.1, 161.0)	166.5 (160.6, 172.6)	138.5 (134.3, 142.8)	104.6 (93.9, 115.1)	129.8 (124.9, 134.8)	155.6 (152.7, 158.5)	164.7 (162.1, 167.4)
urban	140.4 (136.0, 144.8)	105.2 (95.8, 114.7)	131.3 (127.0, 135.6)	158.1 (153.7, 162.7)	167.9 (162.0, 174.0)	140.4 (136.3, 144.7)	107.0 (96.4, 117.8)	131.8 (127.0, 136.9)	157.3 (154.3, 160.3)	166.1 (163.6, 168.7)
urban-rural difference	1.8 (0.1, 3.5)	2.3 (0.2, 4.5)	2.0 (0.2, 3.7)	1.6 (-0.2, 3.4)	1.4 (-0.7, 3.3)	1.9 (0.3, 3.5)	2.4 (0.0, 4.8)	2.0 (0.3, 3.9)	1.7 (0.3, 3.1)	1.4 (0.1, 2.8)
Mali										
rural	147.2 (143.3, 151.2)	110.9 (101.5, 120.3)	137.8 (133.7, 142.0)	165.5 (161.9, 169.2)	175.7 (172.1, 179.5)	147.7 (143.1, 152.3)	113.4 (102.6, 124.4)	139.0 (133.8, 144.2)	165.0 (161.6, 168.5)	173.7 (170.4, 177.1)
urban	149.6 (145.4, 153.7)	113.6 (104.0, 122.9)	140.3 (135.9, 144.6)	167.8 (163.9, 171.6)	177.7 (173.6, 181.7)	150.1 (145.5, 154.8)	116.2 (105.5, 127.2)	141.6 (136.3, 146.9)	167.3 (163.7, 170.9)	175.8 (172.4, 179.1)
urban-rural difference	2.4 (0.4, 4.3)	2.7 (0.5, 5.0)	2.5 (0.5, 4.5)	2.2 (0.2, 4.2)	2.0 (-0.2, 4.2)	2.4 (0.6, 4.4)	2.8 (0.3, 5.4)	2.5 (0.5, 4.6)	2.3 (0.6, 4.0)	2.1 (0.4, 3.8)
Mauritania										
rural	141.6 (136.6, 146.7)	105.5 (95.8, 115.5)	132.8 (127.7, 138.0)	159.9 (154.8, 165.0)	168.6 (162.3, 174.7)	141.8 (136.2, 147.1)	108.2 (97.3, 119.4)	133.6 (127.6, 139.6)	158.9 (154.3, 163.4)	166.1 (161.5, 170.7)
urban	143.7 (138.9, 148.7)	108.0 (98.2, 118.0)	135.0 (130.2, 140.1)	161.8 (156.9, 166.7)	170.3 (164.3, 176.0)	143.9 (138.6, 149.3)	110.8 (100.0, 122.0)	135.9 (130.0, 141.9)	160.8 (156.4, 165.2)	167.8 (163.4, 172.3)
urban-rural difference	2.1 (0.1, 4.1)	2.5 (0.2, 4.9)	2.2 (0.1, 4.3)	1.9 (-0.1, 4.0)	1.7 (-0.6, 4.0)	2.2 (0.1, 4.2)	2.6 (0.0, 5.1)	2.3 (0.1, 4.4)	2.0 (0.1, 3.8)	1.7 (-0.2, 3.6)
Niger										
rural	142.9 (138.5, 147.1)	107.1 (97.7, 116.6)	133.7 (129.3, 137.9)	160.9 (156.3, 165.3)	171.0 (165.4, 176.7)	142.9 (138.1, 147.7)	108.9 (97.8, 119.7)	134.2 (128.7, 139.6)	160.1 (156.4, 163.9)	169.4 (166.0, 172.8)
urban	145.0 (140.6, 149.5)	109.7 (100.2, 119.2)	136.0 (131.4, 140.4)	162.9 (158.2, 167.6)	172.8 (167.0, 178.7)	145.2 (140.2, 150.1)	111.5 (100.6, 122.5)	136.6 (130.8, 142.1)	162.2 (158.2, 166.1)	171.2 (167.6, 174.9)
urban-rural difference	2.2 (0.1, 4.2)	2.6 (0.3, 4.9)	2.3 (0.2, 4.4)	2.0 (-0.1, 4.0)	1.8 (-0.6, 4.0)	2.2 (0.3, 4.2)	2.7 (0.1, 5.2)	2.4 (0.2, 4.5)	2.1 (0.2, 3.9)	1.8 (0.0, 3.7)
Nigeria										
rural	141.7 (138.3, 145.2)	108.2 (104.5, 111.9)	132.2 (129.0, 135.5)	158.8 (154.5, 163.2)	170.1 (164.2, 175.8)	141.4 (138.7, 144.1)	108.8 (104.4, 113.3)	132.2 (129.0, 135.5)	158.1 (155.6, 160.6)	168.8 (166.7, 171.0)
urban	143.8 (140.3, 147.4)	110.8 (106.9, 114.5)	134.5 (131.1, 137.9)	160.7 (156.3, 165.0)	171.6 (165.8, 177.3)	143.6 (140.6, 146.6)	111.5 (106.6, 116.4)	134.5 (131.0, 138.0)	160.1 (157.5, 162.6)	170.5 (168.2, 172.7)
urban-rural difference	2.1 (0.1, 3.9)	2.6 (0.5, 4.7)	2.2 (0.2, 4.1)	1.8 (-0.1, 3.7)	1.5 (-0.6, 3.5)	2.2 (0.5, 3.9)	2.7 (0.5, 5.0)	2.3 (0.6, 4.2)	1.9 (0.4, 3.5)	1.6 (0.1, 3.2)
Sao Tome and Principe										
rural	143.5 (139.4, 147.7)	107.6 (98.5, 117.0)	134.2 (130.1, 138.4)	161.6 (157.3, 165.9)	172.0 (166.8, 177.3)	144.2 (140.0, 148.6)	110.4 (100.0, 121.1)	135.5 (130.6, 140.6)	161.4 (158.5, 164.4)	170.5 (168.3, 172.7)
urban	145.2 (141.2, 149.3)	109.7 (100.7, 119.2)	136.0 (131.9, 140.2)	163.0 (158.7, 167.4)	173.2 (167.8, 178.6)	145.9 (141.6, 150.1)	112.5 (102.1, 123.1)	137.3 (132.4, 142.3)	162.8 (159.9, 165.9)	171.7 (169.7, 173.9)
urban-rural difference	1.7 (0.0, 3.4)	2.2 (0.1, 4.3)	1.8 (0.1, 3.6)	1.5 (-0.2, 3.2)	1.2 (-0.6, 3.1)	1.6 (0.0, 3.3)	2.1 (-0.3, 4.5)	1.8 (-0.1, 3.6)	1.4 (0.0, 2.9)	1.2 (-0.2, 2.5)

Boys	Mean height in 1990 (cm) Age-standardised	5 years	10 years	15 years	19 years	Mean height in 2020 (cm) Age-standardised	5 years	10 years	15 years	19 years
Senegal										
rural	147.4 (143.1, 151.5)	111.8 (102.4, 121.3)	138.3 (134.1, 142.3)	165.3 (160.7, 169.7)	175.3 (169.4, 181.3)	146.9 (142.4, 151.4)	112.6 (101.5, 123.8)	138.0 (132.9, 143.3)	164.2 (161.1, 167.2)	173.7 (171.2, 176.1)
urban	150.2 (146.1, 154.4)	114.9 (105.5, 124.5)	141.2 (137.0, 145.3)	167.9 (163.5, 172.4)	177.8 (171.9, 183.7)	149.7 (145.2, 154.3)	115.8 (104.7, 126.9)	141.0 (135.8, 146.2)	166.9 (163.8, 170.0)	176.2 (173.8, 178.7)
urban-rural difference	2.8 (1.2, 4.5)	3.1 (1.1, 5.2)	2.9 (1.2, 4.6)	2.7 (1.1, 4.3)	2.5 (0.8, 4.3)	2.9 (1.3, 4.5)	3.2 (0.9, 5.5)	3.0 (1.2, 4.7)	2.7 (1.4, 4.1)	2.6 (1.3, 3.8)
Sierra Leone										
rural	140.3 (136.5, 144.2)	105.1 (96.0, 114.3)	131.6 (127.7, 135.4)	158.1 (154.3, 162.0)	166.8 (161.2, 172.3)	140.2 (136.7, 143.7)	106.4 (96.3, 116.6)	131.9 (127.8, 135.9)	157.4 (155.6, 159.3)	165.4 (163.6, 167.2)
urban	142.7 (138.8, 146.6)	107.9 (98.7, 117.1)	134.1 (130.2, 138.0)	160.4 (156.5, 164.3)	168.8 (163.3, 174.4)	142.7 (139.2, 146.2)	109.2 (99.0, 119.4)	134.5 (130.5, 138.5)	159.7 (157.7, 161.7)	167.5 (165.6, 169.4)
urban-rural difference	2.4 (0.8, 4.2)	2.8 (0.7, 4.9)	2.5 (0.8, 4.3)	2.3 (0.6, 4.0)	2.1 (0.2, 3.9)	2.5 (1.2, 3.9)	2.9 (0.7, 5.1)	2.6 (1.0, 4.2)	2.3 (1.2, 3.5)	2.1 (1.0, 3.2)
Togo										
rural	144.0 (139.6, 148.5)	108.4 (99.0, 117.6)	135.2 (130.6, 139.7)	162.0 (157.3, 166.9)	170.7 (164.7, 176.9)	144.2 (139.4, 149.0)	110.1 (98.9, 121.2)	135.8 (130.4, 141.3)	161.5 (158.0, 165.0)	169.4 (166.3, 172.6)
urban	146.2 (141.6, 150.7)	111.0 (101.4, 120.2)	137.5 (132.9, 142.1)	164.0 (159.2, 168.9)	172.5 (166.3, 178.8)	146.4 (141.6, 151.2)	112.7 (101.9, 123.5)	138.2 (132.6, 143.7)	163.6 (159.9, 167.2)	171.2 (168.0, 174.5)
urban-rural difference	2.2 (0.0, 4.3)	2.5 (0.3, 4.9)	2.3 (0.1, 4.4)	2.0 (-0.2, 4.1)	1.8 (-0.6, 4.1)	2.2 (0.2, 4.2)	2.6 (0.1, 5.2)	2.3 (0.2, 4.4)	2.0 (0.1, 3.9)	1.8 (-0.1, 3.7)

Supplementary Table 4. Urban and rural mean body-mass index (BMI) and urban-rural difference in mean BMI in 1990 and 2020 by sex, age-standardised and at ages 5, 10, 15 and 19 years.

Girls	Mean BMI in 1990 (kg/m^2)					Mean BMI in 2020 (kg/m^2)				
	Age-standardised	5 years	10 years	15 years	19 years	Age-standardised	5 years	10 years	15 years	19 years
**Central and eastern Europe**										
Central Europe										
Albania										
rural	18.2 (16.8, 19.5)	14.9 (13.4, 16.3)	16.7 (15.3, 18.1)	20.3 (18.9, 21.7)	21.6 (20.1, 23.0)	18.7 (18.1, 19.3)	15.4 (14.7, 16.1)	17.3 (16.7, 17.9)	20.8 (20.2, 21.4)	22.1 (21.3, 22.8)
urban	18.4 (17.0, 19.8)	15.4 (14.0, 16.8)	17.0 (15.6, 18.4)	20.4 (19.0, 21.8)	21.5 (20.1, 22.9)	19.1 (18.5, 19.7)	16.0 (15.3, 16.7)	17.7 (17.1, 18.3)	21.1 (20.5, 21.7)	22.2 (21.4, 22.9)
urban-rural difference	0.2 (-0.4, 0.8)	0.5 (-0.2, 1.2)	0.3 (-0.4, 0.9)	0.1 (-0.6, 0.7)	-0.1 (-0.8, 0.6)	0.4 (0.0, 0.7)	0.6 (0.2, 1.1)	0.4 (0.1, 0.8)	0.2 (-0.1, 0.6)	0.1 (-0.4, 0.5)
Bosnia and Herzegovina										
rural	18.0 (16.4, 19.7)	14.7 (12.7, 16.8)	17.0 (15.3, 18.7)	20.1 (18.5, 21.7)	20.5 (18.7, 22.3)	18.9 (17.5, 20.3)	15.6 (13.7, 17.5)	17.8 (16.3, 19.4)	20.9 (19.5, 22.4)	21.3 (19.8, 22.8)
urban	18.0 (16.4, 19.6)	15.0 (13.0, 17.0)	17.1 (15.4, 18.8)	19.9 (18.4, 21.5)	20.2 (18.4, 22.0)	18.7 (17.2, 20.1)	15.7 (13.8, 17.6)	17.7 (16.1, 19.3)	20.6 (19.2, 22.0)	20.8 (19.3, 22.4)
urban-rural difference	0.0 (-0.6, 0.7)	0.3 (-0.5, 1.1)	0.1 (-0.6, 0.7)	-0.1 (-0.8, 0.6)	-0.3 (-1.1, 0.5)	-0.2 (-0.8, 0.4)	0.1 (-0.7, 0.8)	-0.1 (-0.8, 0.4)	-0.3 (-1.0, 0.3)	-0.5 (-1.3, 0.3)
Bulgaria										
rural	18.0 (17.0, 19.1)	14.7 (13.5, 15.8)	17.2 (16.2, 18.2)	20.0 (18.9, 21.2)	19.9 (18.1, 21.6)	19.0 (18.3, 19.7)	15.6 (14.9, 16.3)	18.1 (17.5, 18.8)	21.0 (20.1, 21.8)	20.8 (19.3, 22.4)
urban	18.3 (17.3, 19.2)	15.2 (14.2, 16.2)	17.5 (16.6, 18.4)	20.1 (19.1, 21.1)	19.8 (18.3, 21.5)	19.0 (18.4, 19.6)	16.0 (15.4, 16.6)	18.3 (17.7, 18.9)	20.9 (20.1, 21.6)	20.6 (19.1, 22.0)
urban-rural difference	0.3 (-0.4, 0.8)	0.5 (-0.1, 1.2)	0.3 (-0.2, 0.9)	0.1 (-0.6, 0.8)	0.0 (-0.9, 0.8)	0.0 (-0.4, 0.5)	0.3 (-0.1, 0.8)	0.1 (-0.3, 0.5)	-0.1 (-0.7, 0.5)	-0.3 (-1.1, 0.6)
Croatia										
rural	18.7 (17.4, 19.9)	15.3 (14.0, 16.6)	17.8 (16.4, 19.0)	20.7 (19.4, 22.0)	20.6 (19.3, 22.0)	19.6 (19.1, 20.1)	16.3 (15.7, 16.9)	18.7 (18.2, 19.2)	21.7 (21.1, 22.3)	21.6 (20.9, 22.3)
urban	18.7 (17.4, 19.9)	15.4 (14.0, 16.7)	17.8 (16.5, 19.1)	20.7 (19.4, 22.0)	20.6 (19.2, 21.9)	19.3 (18.8, 19.8)	16.0 (15.4, 16.5)	18.4 (17.8, 18.9)	21.3 (20.8, 21.8)	21.2 (20.5, 21.9)
urban-rural difference	0.0 (-0.5, 0.6)	0.0 (-0.5, 0.7)	0.0 (-0.5, 0.6)	0.0 (-0.5, 0.5)	0.0 (-0.6, 0.6)	-0.4 (-0.7, 0.0)	-0.3 (-0.7, 0.1)	-0.3 (-0.7, 0.0)	-0.4 (-0.8, 0.0)	-0.4 (-0.9, 0.1)
Czechia										
rural	18.3 (17.0, 19.6)	15.2 (13.8, 16.5)	17.1 (15.8, 18.4)	20.2 (18.8, 21.5)	21.3 (19.6, 23.0)	18.7 (18.1, 19.4)	15.6 (15.1, 16.2)	17.6 (17.0, 18.2)	20.6 (19.9, 21.4)	21.8 (20.5, 23.1)
urban	18.3 (17.0, 19.5)	15.3 (14.0, 16.6)	17.1 (15.8, 18.4)	20.1 (18.8, 21.4)	21.2 (19.6, 22.8)	18.6 (17.9, 19.1)	15.6 (15.0, 16.1)	17.4 (16.8, 18.0)	20.4 (19.7, 21.1)	21.5 (20.3, 22.7)
urban-rural difference	0.0 (-0.5, 0.6)	0.1 (-0.4, 0.7)	0.1 (-0.5, 0.6)	0.0 (-0.7, 0.6)	-0.1 (-0.9, 0.8)	-0.2 (-0.6, 0.2)	-0.1 (-0.5, 0.3)	-0.2 (-0.5, 0.2)	-0.2 (-0.8, 0.3)	-0.3 (-1.1, 0.5)
Hungary										
rural	18.8 (17.4, 20.2)	15.1 (13.7, 16.5)	17.8 (16.4, 19.2)	20.9 (19.5, 22.3)	21.4 (19.9, 22.8)	20.3 (20.0, 20.7)	16.6 (16.1, 17.2)	19.3 (19.0, 19.7)	22.4 (22.0, 22.8)	22.9 (22.3, 23.4)
urban	18.9 (17.4, 20.2)	15.2 (13.8, 16.6)	17.9 (16.5, 19.2)	20.9 (19.5, 22.3)	21.4 (19.9, 22.7)	20.0 (19.7, 20.3)	16.3 (15.8, 16.9)	19.0 (18.7, 19.4)	22.1 (21.7, 22.4)	22.5 (22.1, 22.9)
urban-rural difference	0.0 (-0.5, 0.7)	0.1 (-0.5, 0.8)	0.1 (-0.5, 0.7)	0.0 (-0.6, 0.7)	0.0 (-0.7, 0.8)	-0.3 (-0.5, -0.1)	-0.3 (-0.7, 0.1)	-0.3 (-0.5, -0.1)	-0.3 (-0.6, 0.0)	-0.4 (-0.8, 0.1)
North Macedonia										
rural	18.3 (17.1, 19.6)	15.0 (13.6, 16.5)	17.1 (15.8, 18.4)	20.4 (19.1, 21.7)	21.4 (19.9, 22.9)	19.2 (18.5, 19.9)	15.9 (15.1, 16.6)	18.0 (17.3, 18.6)	21.2 (20.3, 22.1)	22.3 (20.9, 23.6)
urban	18.5 (17.3, 19.8)	15.5 (14.1, 16.9)	17.3 (16.0, 18.7)	20.4 (19.1, 21.8)	21.3 (19.8, 22.8)	19.3 (18.6, 19.9)	16.2 (15.5, 17.0)	18.1 (17.4, 18.7)	21.2 (20.3, 22.0)	22.1 (20.7, 23.4)
urban-rural difference	0.2 (-0.4, 0.8)	0.4 (-0.2, 1.1)	0.3 (-0.4, 0.8)	0.1 (-0.6, 0.7)	-0.1 (-1.0, 0.7)	0.1 (-0.4, 0.5)	0.3 (-0.1, 0.8)	0.1 (-0.2, 0.5)	0.0 (-0.6, 0.5)	-0.2 (-1.0, 0.6)
Montenegro										
rural	18.1 (16.7, 19.5)	15.0 (13.5, 16.5)	17.0 (15.6, 18.4)	20.1 (18.7, 21.5)	20.5 (19.0, 22.1)	18.8 (18.4, 19.2)	15.8 (15.2, 16.3)	17.7 (17.3, 18.2)	20.8 (20.3, 21.3)	21.3 (20.5, 22.0)
urban	18.3 (16.9, 19.7)	15.3 (13.8, 16.7)	17.2 (15.8, 18.6)	20.2 (18.8, 21.6)	20.6 (19.1, 22.2)	19.0 (18.6, 19.3)	16.0 (15.5, 16.4)	17.9 (17.5, 18.3)	20.9 (20.4, 21.4)	21.3 (20.6, 22.0)
urban-rural difference	0.2 (-0.4, 0.8)	0.3 (-0.4, 0.9)	0.2 (-0.4, 0.8)	0.2 (-0.5, 0.8)	0.1 (-0.6, 0.9)	0.1 (-0.2, 0.4)	0.2 (-0.2, 0.6)	0.1 (-0.1, 0.4)	0.1 (-0.3, 0.5)	0.0 (-0.5, 0.6)
Poland										
rural	18.3 (17.1, 19.4)	15.0 (13.8, 16.2)	17.3 (16.1, 18.4)	20.3 (19.2, 21.5)	20.7 (19.5, 21.9)	19.0 (18.6, 19.5)	15.8 (15.3, 16.3)	18.0 (17.5, 18.4)	21.0 (20.6, 21.5)	21.5 (20.9, 22.1)
urban	18.3 (17.2, 19.5)	15.1 (13.9, 16.2)	17.3 (16.2, 18.4)	20.4 (19.2, 21.5)	20.8 (19.6, 22.0)	18.8 (18.4, 19.3)	15.5 (15.1, 16.0)	17.8 (17.4, 18.2)	20.8 (20.4, 21.3)	21.3 (20.7, 21.9)
urban-rural difference	0.0 (-0.4, 0.5)	0.0 (-0.5, 0.5)	0.0 (-0.4, 0.5)	0.0 (-0.4, 0.5)	0.0 (-0.5, 0.6)	-0.2 (-0.5, 0.1)	-0.2 (-0.5, 0.1)	-0.2 (-0.5, 0.1)	-0.2 (-0.5, 0.1)	-0.2 (-0.6, 0.2)
Romania										
rural	17.8 (16.6, 18.9)	14.4 (13.3, 15.6)	16.6 (15.5, 17.8)	20.0 (18.8, 21.1)	20.1 (18.8, 21.3)	18.8 (18.3, 19.3)	15.4 (14.9, 15.9)	17.6 (17.1, 18.1)	20.9 (20.4, 21.5)	21.0 (20.3, 21.8)
urban	18.0 (16.8, 19.1)	15.2 (14.1, 16.4)	17.0 (15.8, 18.2)	19.9 (18.7, 21.1)	19.6 (18.4, 20.9)	18.7 (18.2, 19.2)	15.9 (15.5, 16.4)	17.7 (17.2, 18.2)	20.6 (20.1, 21.1)	20.4 (19.7, 21.0)
urban-rural difference	0.2 (-0.2, 0.7)	0.8 (0.3, 1.3)	0.4 (0.0, 0.8)	-0.1 (-0.5, 0.4)	-0.4 (-0.9, 0.1)	-0.1 (-0.4, 0.2)	0.5 (0.2, 0.9)	0.1 (-0.2, 0.4)	-0.3 (-0.7, 0.0)	-0.7 (-1.2, -0.2)
Serbia										
rural	18.2 (16.9, 19.3)	15.0 (13.7, 16.3)	17.1 (15.9, 18.3)	20.1 (18.8, 21.3)	20.7 (19.4, 22.0)	19.5 (18.9, 20.0)	16.3 (15.6, 17.0)	18.4 (17.8, 19.0)	21.4 (20.8, 22.0)	22.0 (21.2, 22.8)
urban	18.4 (17.1, 19.6)	15.5 (14.1, 16.8)	17.4 (16.1, 18.6)	20.2 (18.9, 21.4)	20.6 (19.3, 21.9)	19.3 (18.7, 19.9)	16.4 (15.7, 17.1)	18.3 (17.8, 18.9)	21.1 (20.5, 21.7)	21.6 (20.9, 22.3)
urban-rural difference	0.2 (-0.3, 0.7)	0.4 (-0.1, 1.1)	0.3 (-0.2, 0.8)	0.1 (-0.4, 0.6)	-0.1 (-0.6, 0.5)	-0.2 (-0.5, 0.2)	0.1 (-0.4, 0.5)	-0.1 (-0.5, 0.2)	-0.3 (-0.7, 0.1)	-0.4 (-1.0, 0.1)
Slovakia										
rural	18.4 (17.5, 19.2)	15.1 (14.2, 15.9)	17.2 (16.4, 18.0)	20.4 (19.5, 21.2)	21.2 (20.3, 22.1)	19.4 (18.7, 20.0)	16.1 (15.4, 16.8)	18.2 (17.6, 18.8)	21.4 (20.7, 22.0)	22.2 (21.4, 23.0)
urban	18.3 (17.5, 19.1)	15.3 (14.4, 16.1)	17.2 (16.4, 18.0)	20.2 (19.3, 21.0)	20.8 (19.9, 21.7)	18.9 (18.3, 19.6)	15.9 (15.2, 16.6)	17.8 (17.2, 18.5)	20.8 (20.2, 21.5)	21.5 (20.7, 22.3)
urban-rural difference	-0.1 (-0.5, 0.4)	0.2 (-0.3, 0.8)	0.0 (-0.4, 0.5)	-0.2 (-0.7, 0.3)	-0.4 (-0.9, 0.2)	-0.4 (-0.9, 0.0)	-0.1 (-0.6, 0.3)	-0.4 (-0.8, 0.1)	-0.6 (-1.1, -0.1)	-0.7 (-1.3, -0.2)
Slovenia										

Girls	Mean BMI in 1990 (kg/m^2)					Mean BMI in 2020 (kg/m^2)				
	Age-standardised	5 years	10 years	15 years	19 years	Age-standardised	5 years	10 years	15 years	19 years
rural	18.8 (18.5, 19.1)	15.3 (14.9, 15.6)	17.8 (17.5, 18.1)	20.8 (20.5, 21.1)	21.2 (20.8, 21.6)	19.5 (19.1, 19.9)	16.0 (15.5, 16.5)	18.5 (18.1, 19.0)	21.5 (21.1, 22.0)	22.0 (21.5, 22.5)
urban	19.0 (18.7, 19.3)	15.3 (14.9, 15.6)	17.9 (17.6, 18.2)	21.1 (20.8, 21.4)	21.6 (21.2, 22.0)	19.7 (19.3, 20.1)	16.0 (15.6, 16.5)	18.7 (18.3, 19.1)	21.8 (21.4, 22.3)	22.4 (21.9, 22.8)
urban-rural difference	0.2 (-0.3, 0.6)	0.0 (-0.5, 0.5)	0.1 (-0.3, 0.6)	0.3 (-0.2, 0.8)	0.4 (-0.2, 1.0)	0.2 (-0.2, 0.6)	0.0 (-0.5, 0.5)	0.1 (-0.2, 0.6)	0.3 (-0.2, 0.7)	0.4 (-0.2, 1.0)
Eastern Europe										
Belarus										
rural	18.2 (16.5, 19.9)	15.2 (13.1, 17.3)	17.0 (15.2, 18.8)	20.1 (18.5, 21.9)	20.9 (19.0, 22.8)	18.8 (17.4, 20.1)	15.8 (13.9, 17.7)	17.6 (16.0, 19.1)	20.8 (19.4, 22.1)	21.5 (20.1, 22.9)
urban	18.1 (16.4, 19.8)	15.3 (13.3, 17.4)	17.0 (15.2, 18.8)	20.0 (18.3, 21.7)	20.6 (18.8, 22.4)	18.8 (17.5, 20.0)	16.0 (14.1, 17.8)	17.6 (16.1, 19.1)	20.6 (19.4, 21.9)	21.2 (20.1, 22.4)
urban-rural difference	-0.1 (-0.7, 0.6)	0.1 (-0.7, 0.9)	0.0 (-0.7, 0.7)	-0.1 (-0.8, 0.6)	-0.2 (-1.1, 0.7)	0.0 (-0.7, 0.6)	0.2 (-0.6, 0.9)	0.0 (-0.6, 0.6)	-0.1 (-0.8, 0.6)	-0.2 (-1.1, 0.6)
Estonia										
rural	18.5 (17.2, 19.8)	15.1 (13.7, 16.4)	17.3 (15.9, 18.6)	20.5 (19.2, 21.9)	21.4 (20.0, 22.7)	19.4 (18.8, 20.0)	16.0 (15.3, 16.6)	18.2 (17.6, 18.7)	21.5 (20.8, 22.1)	22.3 (21.5, 23.2)
urban	18.4 (17.2, 19.7)	14.9 (13.6, 16.3)	17.2 (15.9, 18.5)	20.6 (19.3, 21.9)	21.5 (20.2, 22.8)	19.4 (18.8, 19.9)	15.9 (15.2, 16.5)	18.1 (17.6, 18.7)	21.5 (20.8, 22.1)	22.4 (21.5, 23.2)
urban-rural difference	0.0 (-0.6, 0.6)	-0.1 (-0.8, 0.6)	-0.1 (-0.6, 0.5)	0.0 (-0.6, 0.6)	0.1 (-0.5, 0.7)	0.0 (-0.4, 0.3)	-0.1 (-0.5, 0.3)	0.0 (-0.4, 0.3)	0.0 (-0.4, 0.4)	0.1 (-0.4, 0.6)
Latvia										
rural	18.3 (16.8, 19.8)	15.2 (13.7, 16.7)	17.0 (15.6, 18.4)	20.2 (18.6, 21.9)	21.1 (18.5, 23.7)	18.9 (18.1, 19.7)	15.9 (15.2, 16.6)	17.7 (17.1, 18.3)	20.9 (19.9, 21.9)	21.8 (19.4, 24.0)
urban	18.3 (16.8, 19.8)	15.4 (13.9, 16.8)	17.1 (15.7, 18.5)	20.2 (18.6, 21.8)	21.0 (18.3, 23.6)	19.0 (18.2, 19.7)	16.0 (15.4, 16.7)	17.8 (17.2, 18.4)	20.9 (19.9, 21.9)	21.7 (19.3, 23.9)
urban-rural difference	0.0 (-0.6, 0.7)	0.1 (-0.5, 0.8)	0.1 (-0.5, 0.7)	0.0 (-0.7, 0.7)	-0.1 (-1.0, 0.8)	0.0 (-0.4, 0.4)	0.1 (-0.3, 0.5)	0.1 (-0.3, 0.4)	0.0 (-0.6, 0.5)	-0.1 (-0.9, 0.7)
Lithuania										
rural	18.2 (16.7, 19.7)	15.4 (13.9, 17.0)	17.0 (15.5, 18.4)	20.1 (18.6, 21.6)	20.9 (18.7, 23.1)	18.9 (18.2, 19.5)	16.1 (15.3, 16.9)	17.6 (17.0, 18.3)	20.7 (19.9, 21.6)	21.6 (19.8, 23.3)
urban	18.2 (16.8, 19.7)	15.6 (14.1, 17.1)	17.0 (15.6, 18.5)	20.0 (18.5, 21.5)	20.7 (18.5, 22.9)	18.8 (18.2, 19.5)	16.2 (15.5, 17.0)	17.7 (17.0, 18.3)	20.6 (19.8, 21.5)	21.4 (19.6, 23.1)
urban-rural difference	0.0 (-0.6, 0.6)	0.2 (-0.5, 0.8)	0.0 (-0.6, 0.7)	-0.1 (-0.7, 0.6)	-0.2 (-1.0, 0.7)	0.0 (-0.4, 0.4)	0.1 (-0.3, 0.5)	0.0 (-0.3, 0.4)	-0.1 (-0.6, 0.4)	-0.2 (-1.0, 0.6)
Moldova										
rural	18.1 (16.7, 19.5)	14.8 (13.2, 16.5)	16.6 (15.2, 18.1)	20.2 (18.8, 21.6)	21.4 (19.9, 22.9)	18.5 (17.5, 19.6)	15.2 (14.0, 16.5)	17.1 (16.0, 18.2)	20.6 (19.5, 21.7)	21.8 (20.7, 23.0)
urban	18.0 (16.6, 19.4)	15.1 (13.4, 16.7)	16.6 (15.2, 18.1)	20.0 (18.6, 21.4)	21.0 (19.5, 22.4)	18.5 (17.4, 19.5)	15.5 (14.3, 16.8)	17.1 (16.1, 18.2)	20.4 (19.3, 21.5)	21.4 (20.3, 22.6)
urban-rural difference	-0.1 (-0.6, 0.5)	0.3 (-0.4, 1.0)	0.0 (-0.6, 0.6)	-0.2 (-0.8, 0.3)	-0.4 (-1.1, 0.2)	0.0 (-0.5, 0.4)	0.3 (-0.3, 1.0)	0.1 (-0.4, 0.5)	-0.2 (-0.7, 0.3)	-0.4 (-1.0, 0.2)
Russian Federation										
rural	19.0 (18.4, 19.5)	16.5 (15.9, 17.0)	17.4 (16.9, 17.9)	20.8 (20.3, 21.4)	22.3 (21.7, 22.9)	18.8 (18.0, 19.5)	16.3 (15.5, 17.1)	17.2 (16.4, 18.0)	20.7 (19.9, 21.4)	22.1 (21.3, 22.9)
urban	18.6 (18.1, 19.1)	16.3 (15.7, 16.8)	17.1 (16.6, 17.6)	20.4 (19.9, 21.0)	21.8 (21.2, 22.3)	18.8 (18.1, 19.4)	16.4 (15.7, 17.1)	17.2 (16.6, 17.9)	20.6 (19.9, 21.2)	21.9 (21.2, 22.6)
urban-rural difference	-0.3 (-0.6, -0.1)	-0.2 (-0.5, 0.1)	-0.3 (-0.5, 0.0)	-0.4 (-0.7, -0.1)	-0.5 (-0.8, -0.2)	0.0 (-0.5, 0.5)	0.1 (-0.4, 0.7)	0.0 (-0.4, 0.5)	-0.1 (-0.5, 0.4)	-0.2 (-0.7, 0.4)
Ukraine										
rural	17.6 (16.2, 19.1)	15.2 (13.8, 16.8)	16.5 (15.1, 18.0)	19.2 (17.8, 20.7)	20.4 (18.8, 22.0)	17.8 (17.4, 18.3)	15.4 (14.8, 16.0)	16.7 (16.3, 17.2)	19.4 (18.9, 19.9)	20.6 (19.7, 21.4)
urban	17.7 (16.3, 19.2)	15.4 (14.0, 16.9)	16.6 (15.3, 18.1)	19.3 (17.9, 20.8)	20.4 (18.8, 22.1)	18.2 (17.7, 18.6)	15.8 (15.2, 16.4)	17.1 (16.6, 17.5)	19.7 (19.3, 20.2)	20.8 (20.0, 21.7)
urban-rural difference	0.1 (-0.6, 0.7)	0.2 (-0.6, 0.8)	0.1 (-0.5, 0.7)	0.1 (-0.6, 0.7)	0.0 (-0.7, 0.7)	0.3 (0.0, 0.6)	0.4 (0.0, 0.8)	0.3 (0.0, 0.7)	0.3 (0.0, 0.6)	0.3 (-0.2, 0.7)
**Central Asia, Middle East and north Africa**										
Central Asia										
Armenia										
rural	19.1 (17.9, 20.4)	15.7 (14.1, 17.3)	17.6 (16.3, 18.9)	21.4 (20.2, 22.7)	22.6 (21.4, 23.9)	19.0 (18.4, 19.6)	15.6 (14.6, 16.6)	17.5 (16.8, 18.1)	21.3 (20.6, 22.0)	22.5 (21.7, 23.2)
urban	19.0 (17.8, 20.3)	16.1 (14.5, 17.7)	17.6 (16.3, 19.0)	21.1 (19.8, 22.4)	22.0 (20.8, 23.3)	19.1 (18.5, 19.7)	16.2 (15.2, 17.1)	17.7 (17.1, 18.3)	21.2 (20.5, 21.9)	22.1 (21.4, 22.9)
urban-rural difference	-0.1 (-0.7, 0.4)	0.3 (-0.5, 1.1)	0.0 (-0.6, 0.6)	-0.3 (-0.8, 0.2)	-0.6 (-1.1, -0.1)	0.1 (-0.3, 0.5)	0.6 (0.0, 1.2)	0.3 (-0.2, 0.7)	-0.1 (-0.5, 0.3)	-0.3 (-0.9, 0.2)
Azerbaijan										
rural	19.0 (17.6, 20.5)	15.6 (13.7, 17.5)	17.6 (16.1, 19.1)	21.3 (19.9, 22.7)	22.4 (21.0, 23.8)	19.1 (18.0, 20.2)	15.6 (13.9, 17.4)	17.6 (16.4, 18.8)	21.3 (20.3, 22.3)	22.4 (21.4, 23.4)
urban	18.9 (17.5, 20.4)	15.8 (13.8, 17.8)	17.6 (16.0, 19.1)	21.1 (19.7, 22.4)	22.0 (20.6, 23.4)	19.1 (18.0, 20.2)	16.0 (14.2, 17.7)	17.7 (16.5, 18.9)	21.2 (20.2, 22.2)	22.1 (21.1, 23.2)
urban-rural difference	-0.1 (-0.7, 0.5)	0.2 (-0.7, 1.1)	0.0 (-0.7, 0.6)	-0.2 (-0.8, 0.3)	-0.4 (-1.1, 0.2)	0.0 (-0.5, 0.6)	0.3 (-0.5, 1.1)	0.1 (-0.5, 0.7)	-0.1 (-0.6, 0.4)	-0.3 (-0.9, 0.3)
Georgia										
rural	18.5 (17.0, 20.0)	15.2 (13.4, 16.9)	17.1 (15.6, 18.6)	20.8 (19.2, 22.3)	21.6 (19.9, 23.3)	19.1 (18.4, 19.8)	15.8 (14.9, 16.6)	17.7 (17.0, 18.4)	21.4 (20.5, 22.3)	22.2 (20.9, 23.4)
urban	18.5 (17.0, 20.0)	15.6 (13.9, 17.3)	17.2 (15.6, 18.7)	20.6 (19.0, 22.1)	21.1 (19.4, 22.8)	19.2 (18.6, 19.9)	16.3 (15.4, 17.1)	17.9 (17.3, 18.6)	21.3 (20.4, 22.2)	21.9 (20.7, 23.1)
urban-rural difference	0.0 (-0.7, 0.6)	0.4 (-0.4, 1.2)	0.1 (-0.6, 0.7)	-0.2 (-0.9, 0.5)	-0.5 (-1.3, 0.3)	0.1 (-0.3, 0.5)	0.5 (0.0, 1.0)	0.2 (-0.2, 0.6)	-0.1 (-0.6, 0.5)	-0.3 (-1.1, 0.4)
Kazakhstan										
rural	18.6 (17.6, 19.7)	15.4 (14.2, 16.7)	17.3 (16.2, 18.4)	20.6 (19.6, 21.7)	21.8 (20.6, 22.9)	18.2 (17.7, 18.7)	15.0 (14.3, 15.7)	16.9 (16.4, 17.4)	20.2 (19.6, 20.8)	21.4 (20.6, 22.1)
urban	18.7 (17.7, 19.7)	15.8 (14.7, 17.0)	17.5 (16.4, 18.6)	20.6 (19.5, 21.6)	21.5 (20.4, 22.6)	18.4 (18.0, 18.9)	15.6 (14.9, 16.2)	17.2 (16.8, 17.7)	20.3 (19.8, 20.9)	21.3 (20.5, 22.0)
urban-rural difference	0.1 (-0.5, 0.6)	0.4 (-0.3, 1.1)	0.2 (-0.4, 0.7)	-0.1 (-0.6, 0.5)	-0.2 (-0.8, 0.3)	0.2 (-0.1, 0.6)	0.5 (0.1, 1.0)	0.3 (0.0, 0.7)	0.1 (-0.3, 0.5)	-0.1 (-0.7, 0.5)
Kyrgyzstan										

Girls	Mean BMI in 1990 (kg/m^2)					Mean BMI in 2020 (kg/m^2)				
	Age-standardised	5 years	10 years	15 years	19 years	Age-standardised	5 years	10 years	15 years	19 years
rural	18.2 (17.1, 19.4)	14.9 (13.6, 16.4)	16.7 (15.5, 17.9)	20.4 (19.2, 21.5)	21.8 (20.6, 23.0)	18.3 (17.4, 19.1)	15.0 (14.0, 16.0)	16.8 (15.9, 17.6)	20.4 (19.5, 21.4)	21.8 (20.7, 22.9)
urban	18.2 (17.0, 19.3)	15.2 (13.8, 16.6)	16.7 (15.5, 18.0)	20.2 (19.0, 21.3)	21.4 (20.2, 22.6)	18.2 (17.4, 19.1)	15.3 (14.3, 16.2)	16.8 (16.0, 17.7)	20.3 (19.3, 21.2)	21.5 (20.4, 22.6)
urban-rural difference	-0.1 (-0.6, 0.5)	0.2 (-0.5, 1.0)	0.0 (-0.5, 0.6)	-0.2 (-0.7, 0.3)	-0.4 (-0.9, 0.2)	0.0 (-0.5, 0.4)	0.3 (-0.3, 0.8)	0.0 (-0.4, 0.4)	-0.2 (-0.6, 0.3)	-0.3 (-0.9, 0.2)
Mongolia										
rural	18.5 (17.0, 20.0)	15.0 (13.0, 17.0)	17.0 (15.4, 18.5)	20.7 (19.3, 22.2)	22.1 (20.6, 23.6)	19.1 (18.4, 19.8)	15.6 (14.1, 17.1)	17.6 (16.8, 18.4)	21.3 (20.7, 21.9)	22.7 (22.0, 23.4)
urban	18.4 (16.8, 19.9)	15.2 (13.2, 17.2)	16.9 (15.4, 18.5)	20.5 (19.0, 22.0)	21.7 (20.2, 23.2)	19.0 (18.3, 19.8)	15.8 (14.3, 17.3)	17.6 (16.8, 18.4)	21.2 (20.5, 21.8)	22.4 (21.6, 23.2)
urban-rural difference	-0.1 (-0.7, 0.5)	0.2 (-0.7, 1.0)	0.0 (-0.7, 0.6)	-0.2 (-0.8, 0.4)	-0.4 (-1.0, 0.3)	0.0 (-0.5, 0.4)	0.2 (-0.5, 1.0)	0.0 (-0.5, 0.6)	-0.1 (-0.6, 0.3)	-0.3 (-0.8, 0.2)
Tajikistan										
rural	17.8 (16.4, 19.3)	14.2 (12.5, 15.8)	16.3 (14.9, 17.8)	20.2 (18.8, 21.6)	21.4 (20.0, 22.8)	18.1 (17.6, 18.6)	14.4 (13.6, 15.3)	16.6 (16.0, 17.2)	20.5 (19.8, 21.1)	21.7 (21.0, 22.4)
urban	17.8 (16.4, 19.3)	14.4 (12.8, 16.1)	16.4 (14.9, 17.8)	20.0 (18.6, 21.4)	21.0 (19.5, 22.5)	18.0 (17.5, 18.5)	14.6 (13.8, 15.5)	16.6 (16.0, 17.2)	20.2 (19.6, 20.8)	21.2 (20.5, 21.9)
urban-rural difference	-0.1 (-0.7, 0.6)	0.2 (-0.5, 1.1)	0.0 (-0.6, 0.7)	-0.2 (-0.8, 0.5)	-0.4 (-1.0, 0.4)	-0.1 (-0.5, 0.2)	0.2 (-0.3, 0.7)	0.0 (-0.4, 0.3)	-0.2 (-0.6, 0.1)	-0.4 (-0.9, 0.0)
Turkmenistan										
rural	18.1 (16.6, 19.6)	14.6 (12.9, 16.4)	16.6 (15.0, 18.2)	20.3 (18.8, 21.9)	21.7 (20.0, 23.3)	18.3 (17.5, 19.1)	14.9 (13.8, 15.9)	16.8 (15.9, 17.7)	20.6 (19.6, 21.5)	21.9 (21.0, 22.9)
urban	18.1 (16.6, 19.7)	14.9 (13.2, 16.7)	16.7 (15.2, 18.3)	20.2 (18.7, 21.9)	21.4 (19.9, 23.1)	18.5 (17.7, 19.2)	15.3 (14.2, 16.3)	17.0 (16.2, 17.9)	20.6 (19.7, 21.5)	21.8 (20.8, 22.7)
urban-rural difference	0.0 (-0.6, 0.7)	0.3 (-0.5, 1.1)	0.1 (-0.5, 0.8)	-0.1 (-0.8, 0.6)	-0.2 (-1.1, 0.6)	0.1 (-0.3, 0.6)	0.4 (-0.2, 1.0)	0.2 (-0.3, 0.7)	0.0 (-0.6, 0.6)	-0.1 (-0.9, 0.6)
Uzbekistan										
rural	18.2 (17.0, 19.4)	14.8 (13.0, 16.6)	16.8 (15.5, 18.1)	20.5 (19.4, 21.5)	21.4 (20.2, 22.5)	19.3 (18.3, 20.3)	15.9 (14.2, 17.6)	17.9 (16.8, 19.1)	21.6 (20.7, 22.5)	22.4 (21.6, 23.3)
urban	18.3 (17.1, 19.5)	15.2 (13.4, 17.0)	17.0 (15.7, 18.3)	20.4 (19.3, 21.5)	21.1 (20.0, 22.2)	19.4 (18.4, 20.4)	16.3 (14.7, 18.0)	18.1 (17.0, 19.2)	21.5 (20.6, 22.4)	22.2 (21.3, 23.1)
urban-rural difference	0.1 (-0.5, 0.6)	0.4 (-0.4, 1.3)	0.2 (-0.4, 0.8)	-0.1 (-0.6, 0.4)	-0.3 (-0.8, 0.3)	0.1 (-0.5, 0.6)	0.4 (-0.3, 1.2)	0.2 (-0.4, 0.8)	-0.1 (-0.6, 0.4)	-0.3 (-0.9, 0.3)
Middle East and north Africa										
Algeria										
rural	19.0 (17.4, 20.7)	15.4 (13.4, 17.4)	17.4 (15.6, 19.1)	21.5 (19.8, 23.1)	22.8 (21.0, 24.7)	20.0 (18.8, 21.4)	16.4 (14.7, 18.2)	18.4 (16.9, 19.9)	22.5 (21.2, 23.8)	23.8 (22.5, 25.2)
urban	19.2 (17.6, 20.8)	15.6 (13.6, 17.6)	17.5 (15.8, 19.3)	21.6 (20.0, 23.2)	22.9 (21.2, 24.8)	20.5 (19.2, 21.7)	16.9 (15.1, 18.6)	18.8 (17.4, 20.3)	22.9 (21.6, 24.2)	24.2 (22.9, 25.5)
urban-rural difference	0.1 (-0.5, 0.8)	0.1 (-0.7, 0.9)	0.1 (-0.6, 0.8)	0.1 (-0.6, 0.8)	0.1 (-0.8, 1.0)	0.4 (-0.2, 1.0)	0.4 (-0.4, 1.2)	0.4 (-0.2, 1.1)	0.4 (-0.2, 1.1)	0.4 (-0.4, 1.2)
Bahrain										
rural	19.3 (17.9, 20.8)	15.4 (13.7, 17.0)	17.6 (16.2, 19.1)	21.9 (20.4, 23.3)	23.6 (21.7, 25.5)	20.7 (19.7, 21.7)	16.7 (15.3, 18.1)	19.0 (18.0, 20.0)	23.2 (22.2, 24.2)	24.9 (23.4, 26.6)
urban	19.5 (18.1, 20.9)	15.5 (13.9, 17.1)	17.8 (16.4, 19.1)	22.0 (20.7, 23.4)	23.8 (22.0, 25.6)	21.2 (20.3, 22.1)	17.2 (15.9, 18.4)	19.5 (18.6, 20.4)	23.7 (22.9, 24.6)	25.5 (24.1, 27.0)
urban-rural difference	0.1 (-0.5, 0.8)	0.1 (-0.7, 0.9)	0.1 (-0.5, 0.8)	0.2 (-0.6, 0.9)	0.2 (-0.7, 1.1)	0.5 (-0.2, 1.1)	0.4 (-0.4, 1.2)	0.5 (-0.2, 1.1)	0.5 (-0.2, 1.2)	0.6 (-0.3, 1.4)
Egypt										
rural	20.4 (19.0, 21.7)	16.9 (15.3, 18.4)	18.5 (17.1, 19.8)	22.9 (21.6, 24.2)	24.6 (23.1, 26.0)	21.1 (20.2, 21.9)	17.6 (16.4, 18.7)	19.2 (18.3, 20.1)	23.6 (22.8, 24.4)	25.3 (24.4, 26.2)
urban	20.5 (19.1, 21.8)	17.0 (15.4, 18.6)	18.6 (17.2, 19.9)	23.0 (21.6, 24.3)	24.6 (23.2, 26.0)	21.4 (20.6, 22.3)	18.0 (16.7, 19.2)	19.6 (18.6, 20.5)	24.0 (23.1, 24.8)	25.6 (24.7, 26.5)
urban-rural difference	0.1 (-0.6, 0.7)	0.1 (-0.7, 0.9)	0.1 (-0.6, 0.8)	0.0 (-0.6, 0.7)	0.0 (-0.8, 0.9)	0.4 (-0.2, 0.9)	0.4 (-0.4, 1.2)	0.4 (-0.2, 0.9)	0.3 (-0.2, 0.8)	0.3 (-0.3, 0.9)
Iran										
rural	17.5 (16.9, 18.1)	14.2 (13.6, 14.8)	15.9 (15.3, 16.5)	19.8 (19.2, 20.4)	21.0 (20.4, 21.6)	18.7 (18.1, 19.3)	15.4 (14.8, 16.1)	17.1 (16.5, 17.7)	21.1 (20.5, 21.7)	22.2 (21.6, 22.8)
urban	17.7 (17.1, 18.2)	14.4 (13.8, 15.0)	16.1 (15.5, 16.6)	20.0 (19.4, 20.6)	21.2 (20.6, 21.7)	19.4 (18.8, 20.0)	16.1 (15.5, 16.7)	17.8 (17.2, 18.4)	21.7 (21.1, 22.3)	22.9 (22.3, 23.5)
urban-rural difference	0.2 (-0.1, 0.5)	0.2 (-0.2, 0.5)	0.2 (-0.1, 0.5)	0.2 (-0.2, 0.5)	0.2 (-0.2, 0.5)	0.7 (0.4, 0.9)	0.7 (0.4, 1.0)	0.7 (0.4, 0.9)	0.7 (0.4, 0.9)	0.7 (0.4, 0.9)
Iraq										
rural	19.5 (18.0, 21.1)	15.9 (13.9, 18.0)	17.8 (16.2, 19.5)	21.9 (20.3, 23.5)	23.7 (22.0, 25.5)	20.7 (19.4, 21.9)	17.1 (15.3, 18.8)	19.0 (17.6, 20.3)	23.0 (21.8, 24.3)	24.9 (23.5, 26.3)
urban	19.7 (18.1, 21.2)	16.1 (14.1, 18.0)	18.0 (16.3, 19.6)	22.0 (20.4, 23.6)	23.8 (22.1, 25.6)	21.1 (19.9, 22.3)	17.5 (15.8, 19.3)	19.4 (18.1, 20.7)	23.4 (22.2, 24.6)	25.2 (23.9, 26.6)
urban-rural difference	0.1 (-0.5, 0.8)	0.2 (-0.6, 1.0)	0.1 (-0.5, 0.8)	0.1 (-0.6, 0.8)	0.1 (-0.8, 1.0)	0.4 (-0.2, 1.0)	0.5 (-0.3, 1.2)	0.4 (-0.2, 1.0)	0.4 (-0.3, 1.1)	0.4 (-0.4, 1.2)
Jordan										
rural	18.8 (17.3, 20.2)	15.4 (13.7, 17.0)	17.2 (15.8, 18.7)	21.0 (19.6, 22.5)	22.7 (21.1, 24.2)	19.7 (18.9, 20.5)	16.3 (15.1, 17.4)	18.1 (17.3, 19.0)	21.9 (21.1, 22.8)	23.6 (22.6, 24.6)
urban	18.9 (17.5, 20.3)	15.3 (13.8, 16.9)	17.3 (15.9, 18.7)	21.2 (19.8, 22.6)	22.9 (21.4, 24.5)	20.1 (19.4, 20.9)	16.6 (15.6, 17.6)	18.5 (17.7, 19.3)	22.4 (21.7, 23.2)	24.2 (23.3, 25.0)
urban-rural difference	0.1 (-0.5, 0.7)	0.0 (-0.7, 0.8)	0.1 (-0.5, 0.7)	0.2 (-0.4, 0.8)	0.3 (-0.5, 1.0)	0.4 (0.0, 0.9)	0.3 (-0.4, 1.0)	0.4 (-0.1, 0.9)	0.5 (0.0, 1.0)	0.6 (-0.1, 1.2)
Kuwait										
rural	19.9 (18.5, 21.3)	14.7 (13.3, 16.1)	18.6 (17.2, 19.9)	22.9 (21.5, 24.3)	23.5 (22.0, 25.1)	20.6 (19.5, 21.7)	15.4 (14.2, 16.6)	19.2 (18.1, 20.3)	23.6 (22.4, 24.7)	24.2 (22.8, 25.5)
urban	20.2 (18.9, 21.4)	14.7 (13.4, 16.0)	18.8 (17.4, 20.0)	23.3 (22.0, 24.6)	24.1 (22.8, 25.4)	21.2 (20.2, 22.1)	15.7 (14.7, 16.6)	19.7 (18.8, 20.6)	24.3 (23.3, 25.2)	25.1 (24.0, 26.0)
urban-rural difference	0.3 (-0.4, 1.0)	0.0 (-0.8, 0.8)	0.2 (-0.5, 0.9)	0.4 (-0.3, 1.1)	0.6 (-0.3, 1.5)	0.5 (-0.1, 1.2)	0.3 (-0.5, 1.1)	0.5 (-0.2, 1.1)	0.7 (0.0, 1.4)	0.8 (0.0, 1.7)
Lebanon										
rural	18.3 (17.2, 19.3)	15.0 (13.9, 16.1)	17.1 (16.0, 18.2)	20.5 (19.4, 21.6)	21.0 (19.7, 22.2)	19.8 (18.6, 21.0)	16.5 (15.3, 17.8)	18.6 (17.4, 19.8)	22.0 (20.8, 23.3)	22.5 (21.2, 23.8)
urban	18.5 (17.4, 19.6)	15.3 (14.1, 16.5)	17.3 (16.2, 18.4)	20.7 (19.6, 21.8)	21.1 (19.9, 22.3)	20.3 (19.1, 21.4)	17.1 (15.9, 18.3)	19.1 (17.9, 20.3)	22.4 (21.3, 23.6)	22.8 (21.6, 24.1)

Girls	Mean BMI in 1990 (kg/m^2)					Mean BMI in 2020 (kg/m^2)				
	Age-standardised	5 years	10 years	15 years	19 years	Age-standardised	5 years	10 years	15 years	19 years
urban-rural difference	0.2 (-0.3, 0.7)	0.3 (-0.3, 0.9)	0.2 (-0.3, 0.7)	0.1 (-0.4, 0.7)	0.1 (-0.6, 0.7)	0.5 (-0.1, 1.0)	0.6 (-0.1, 1.2)	0.5 (0.0, 1.0)	0.4 (-0.2, 1.0)	0.4 (-0.3, 1.0)
Libya										
rural	18.8 (16.8, 20.8)	15.2 (13.0, 17.5)	17.2 (15.3, 19.1)	21.2 (19.1, 23.2)	22.3 (19.5, 25.1)	19.7 (17.8, 21.6)	16.1 (14.0, 18.3)	18.1 (16.3, 20.0)	22.1 (20.2, 24.1)	23.2 (20.5, 26.0)
urban	18.9 (16.9, 20.9)	15.3 (13.1, 17.6)	17.4 (15.4, 19.3)	21.3 (19.3, 23.4)	22.5 (19.7, 25.3)	20.2 (18.2, 22.0)	16.6 (14.4, 18.7)	18.6 (16.7, 20.5)	22.6 (20.6, 24.5)	23.7 (21.0, 26.4)
urban-rural difference	0.1 (-0.5, 0.8)	0.1 (-0.7, 1.0)	0.1 (-0.5, 0.8)	0.1 (-0.6, 0.9)	0.2 (-0.7, 1.1)	0.5 (-0.2, 1.1)	0.4 (-0.4, 1.2)	0.4 (-0.2, 1.1)	0.5 (-0.2, 1.2)	0.5 (-0.4, 1.4)
Morocco										
rural	18.1 (16.8, 19.5)	14.7 (13.1, 16.2)	16.5 (15.1, 17.9)	20.5 (19.1, 21.8)	21.7 (20.3, 23.1)	18.9 (18.3, 19.4)	15.4 (14.7, 16.2)	17.3 (16.7, 17.8)	21.2 (20.6, 21.9)	22.5 (21.7, 23.3)
urban	18.1 (16.8, 19.5)	14.9 (13.4, 16.4)	16.6 (15.2, 18.0)	20.4 (19.1, 21.7)	21.5 (20.1, 22.9)	19.2 (18.6, 19.7)	16.0 (15.2, 16.7)	17.6 (17.1, 18.2)	21.5 (20.8, 22.1)	22.6 (21.8, 23.4)
urban-rural difference	0.0 (-0.5, 0.6)	0.2 (-0.5, 1.0)	0.1 (-0.5, 0.6)	-0.1 (-0.6, 0.5)	-0.2 (-0.8, 0.5)	0.3 (-0.1, 0.7)	0.5 (0.0, 1.1)	0.4 (-0.1, 0.8)	0.2 (-0.2, 0.7)	0.1 (-0.5, 0.7)
Occupied Palestinian Territory										
rural	19.5 (18.2, 20.9)	15.9 (14.0, 17.7)	18.1 (16.6, 19.5)	22.0 (20.7, 23.3)	22.5 (21.1, 24.0)	20.0 (18.7, 21.4)	16.4 (14.6, 18.2)	18.6 (17.3, 20.0)	22.5 (21.3, 23.8)	23.0 (21.6, 24.5)
urban	19.5 (18.1, 20.9)	15.8 (14.0, 17.7)	18.0 (16.6, 19.5)	22.0 (20.7, 23.3)	22.5 (21.2, 23.9)	20.4 (19.1, 21.6)	16.7 (15.0, 18.4)	18.9 (17.6, 20.2)	22.9 (21.6, 24.1)	23.4 (22.1, 24.8)
urban-rural difference	0.0 (-0.7, 0.6)	-0.1 (-0.9, 0.7)	0.0 (-0.7, 0.6)	0.0 (-0.7, 0.6)	0.0 (-0.8, 0.8)	0.3 (-0.3, 0.9)	0.3 (-0.5, 1.1)	0.3 (-0.3, 0.9)	0.3 (-0.3, 1.0)	0.4 (-0.4, 1.2)
Oman										
rural	18.1 (16.6, 19.6)	14.7 (12.8, 16.6)	16.6 (15.1, 18.1)	20.5 (19.0, 21.9)	21.6 (19.9, 23.3)	19.4 (18.3, 20.4)	15.9 (14.3, 17.6)	17.8 (16.7, 19.0)	21.7 (20.7, 22.7)	22.8 (21.5, 24.1)
urban	18.3 (16.9, 19.8)	14.8 (12.9, 16.7)	16.8 (15.2, 18.3)	20.7 (19.3, 22.2)	21.9 (20.2, 23.6)	19.9 (19.0, 20.8)	16.4 (14.8, 17.9)	18.4 (17.4, 19.4)	22.3 (21.4, 23.2)	23.5 (22.3, 24.6)
urban-rural difference	0.2 (-0.5, 0.8)	0.1 (-0.7, 0.9)	0.2 (-0.5, 0.8)	0.2 (-0.5, 1.0)	0.3 (-0.5, 1.2)	0.6 (-0.1, 1.2)	0.4 (-0.4, 1.2)	0.5 (-0.1, 1.2)	0.6 (-0.1, 1.3)	0.7 (-0.2, 1.5)
Qatar										
rural	19.4 (17.9, 21.0)	15.8 (13.9, 17.8)	17.8 (16.2, 19.4)	21.9 (20.3, 23.5)	23.3 (21.5, 25.2)	20.3 (18.9, 21.7)	16.7 (14.9, 18.6)	18.7 (17.2, 20.1)	22.8 (21.3, 24.2)	24.2 (22.5, 25.9)
urban	19.6 (18.1, 21.1)	15.9 (14.0, 17.9)	17.9 (16.3, 19.5)	22.1 (20.5, 23.5)	23.5 (21.8, 25.2)	20.8 (19.5, 22.1)	17.2 (15.4, 18.9)	19.2 (17.7, 20.5)	23.3 (21.9, 24.6)	24.7 (23.2, 26.3)
urban-rural difference	0.2 (-0.5, 0.8)	0.1 (-0.7, 0.9)	0.1 (-0.5, 0.8)	0.2 (-0.5, 0.9)	0.2 (-0.7, 1.1)	0.5 (-0.2, 1.1)	0.4 (-0.4, 1.2)	0.5 (-0.2, 1.1)	0.5 (-0.2, 1.2)	0.5 (-0.4, 1.4)
Saudi Arabia										
rural	19.2 (18.1, 20.2)	14.9 (13.7, 16.2)	17.5 (16.4, 18.6)	21.8 (20.7, 22.9)	23.6 (22.4, 24.7)	20.0 (18.9, 21.2)	15.8 (14.5, 17.1)	18.3 (17.2, 19.5)	22.6 (21.5, 23.8)	24.4 (23.2, 25.7)
urban	19.2 (18.2, 20.1)	15.0 (13.9, 16.1)	17.5 (16.5, 18.5)	21.8 (20.8, 22.8)	23.5 (22.5, 24.5)	20.3 (19.3, 21.3)	16.1 (14.9, 17.4)	18.6 (17.5, 19.7)	22.9 (21.8, 23.9)	24.6 (23.5, 25.7)
urban-rural difference	0.0 (-0.6, 0.7)	0.1 (-0.7, 0.9)	0.0 (-0.6, 0.7)	0.0 (-0.7, 0.7)	-0.1 (-0.9, 0.8)	0.3 (-0.3, 0.8)	0.4 (-0.4, 1.1)	0.3 (-0.3, 0.9)	0.2 (-0.4, 0.8)	0.2 (-0.6, 0.9)
Syrian Arab Republic										
rural	18.8 (16.9, 20.7)	15.2 (13.1, 17.4)	17.2 (15.4, 19.1)	21.2 (19.2, 23.2)	22.3 (19.5, 25.1)	19.7 (17.8, 21.6)	16.2 (14.0, 18.3)	18.2 (16.3, 20.0)	22.1 (20.2, 24.1)	23.2 (20.5, 26.0)
urban	18.9 (17.0, 20.8)	15.3 (13.2, 17.5)	17.4 (15.5, 19.3)	21.3 (19.3, 23.3)	22.4 (19.6, 25.2)	20.2 (18.3, 22.0)	16.6 (14.4, 18.8)	18.6 (16.8, 20.4)	22.6 (20.6, 24.5)	23.7 (21.0, 26.4)
urban-rural difference	0.1 (-0.5, 0.8)	0.1 (-0.7, 0.9)	0.1 (-0.5, 0.8)	0.1 (-0.6, 0.9)	0.2 (-0.8, 1.0)	0.4 (-0.2, 1.1)	0.4 (-0.4, 1.2)	0.4 (-0.2, 1.1)	0.5 (-0.3, 1.2)	0.5 (-0.4, 1.3)
Tunisia										
rural	17.9 (16.8, 19.0)	14.8 (13.7, 16.0)	16.0 (14.9, 17.1)	20.2 (19.1, 21.4)	22.0 (20.8, 23.2)	19.2 (17.9, 20.4)	16.1 (14.8, 17.4)	17.3 (16.0, 18.6)	21.5 (20.2, 22.8)	23.3 (21.9, 24.6)
urban	18.0 (16.9, 19.1)	14.8 (13.7, 16.0)	16.0 (15.0, 17.1)	20.3 (19.2, 21.4)	22.1 (20.9, 23.3)	19.5 (18.2, 20.7)	16.4 (15.1, 17.7)	17.6 (16.3, 18.8)	21.8 (20.6, 23.1)	23.6 (22.3, 25.0)
urban-rural difference	0.1 (-0.4, 0.5)	0.0 (-0.5, 0.5)	0.0 (-0.4, 0.5)	0.1 (-0.4, 0.6)	0.1 (-0.4, 0.7)	0.3 (-0.2, 0.9)	0.3 (-0.4, 0.9)	0.3 (-0.3, 0.9)	0.3 (-0.2, 0.9)	0.4 (-0.2, 1.0)
Turkey										
rural	18.2 (16.9, 19.6)	14.4 (13.0, 15.9)	16.8 (15.4, 18.2)	20.7 (19.4, 22.1)	21.5 (20.2, 23.0)	19.2 (18.4, 19.9)	15.4 (14.5, 16.2)	17.7 (17.0, 18.5)	21.6 (20.8, 22.5)	22.5 (21.6, 23.4)
urban	18.3 (17.0, 19.6)	14.7 (13.2, 16.2)	16.9 (15.6, 18.3)	20.6 (19.3, 22.0)	21.3 (20.0, 22.8)	19.6 (18.9, 20.3)	16.0 (15.2, 16.8)	18.2 (17.4, 19.0)	21.9 (21.2, 22.7)	22.6 (21.7, 23.5)
urban-rural difference	0.1 (-0.5, 0.6)	0.3 (-0.4, 1.0)	0.1 (-0.5, 0.7)	0.0 (-0.6, 0.5)	-0.2 (-0.9, 0.5)	0.4 (0.0, 0.8)	0.6 (0.2, 1.1)	0.5 (0.1, 0.8)	0.3 (-0.1, 0.7)	0.2 (-0.4, 0.7)
United Arab Emirates										
rural	18.9 (17.5, 20.3)	15.8 (13.9, 17.7)	17.9 (16.4, 19.3)	21.1 (19.7, 22.6)	20.5 (18.9, 22.0)	20.2 (19.2, 21.2)	17.1 (15.5, 18.6)	19.1 (18.1, 20.2)	22.4 (21.4, 23.4)	21.7 (20.7, 22.8)
urban	19.5 (18.1, 20.9)	15.9 (14.0, 17.7)	18.4 (17.0, 19.8)	22.1 (20.7, 23.4)	21.7 (20.2, 23.3)	21.3 (20.4, 22.2)	17.6 (16.1, 19.1)	20.1 (19.2, 21.0)	23.8 (23.0, 24.6)	23.5 (22.4, 24.6)
urban-rural difference	0.7 (0.0, 1.4)	0.1 (-0.9, 1.0)	0.5 (-0.2, 1.2)	0.9 (0.2, 1.6)	1.3 (0.4, 2.1)	1.1 (0.5, 1.8)	0.5 (-0.3, 1.4)	1.0 (0.3, 1.7)	1.4 (0.8, 2.1)	1.7 (1.0, 2.6)
Yemen										
rural	17.4 (16.0, 18.8)	14.9 (13.4, 16.3)	15.9 (14.5, 17.3)	19.4 (18.0, 20.8)	20.5 (19.1, 21.9)	17.8 (16.7, 18.8)	15.2 (14.1, 16.4)	16.3 (15.2, 17.3)	19.7 (18.7, 20.8)	20.9 (19.8, 22.0)
urban	17.6 (16.3, 19.0)	14.9 (13.5, 16.4)	16.1 (14.7, 17.5)	19.7 (18.4, 21.1)	21.0 (19.6, 22.5)	18.5 (17.4, 19.6)	15.8 (14.6, 16.9)	16.9 (15.9, 18.0)	20.6 (19.5, 21.7)	21.9 (20.7, 23.0)
urban-rural difference	0.3 (-0.3, 0.8)	0.1 (-0.6, 0.7)	0.2 (-0.4, 0.7)	0.4 (-0.2, 0.9)	0.5 (-0.2, 1.1)	0.7 (0.3, 1.2)	0.5 (-0.1, 1.1)	0.7 (0.2, 1.2)	0.8 (0.4, 1.3)	0.9 (0.5, 1.5)
**East and southeast Asia**										
East Asia										
China										
rural	17.6 (17.2, 17.9)	14.9 (14.5, 15.2)	16.2 (15.8, 16.5)	19.6 (19.2, 19.9)	20.6 (20.2, 21.0)	18.9 (18.1, 19.6)	16.1 (15.4, 16.9)	17.4 (16.7, 18.2)	20.9 (20.1, 21.6)	21.9 (21.2, 22.7)
urban	17.8 (17.4, 18.1)	15.3 (14.9, 15.7)	16.4 (16.1, 16.8)	19.7 (19.3, 20.0)	20.6 (20.2, 21.0)	19.2 (18.5, 20.0)	16.7 (16.0, 17.5)	17.9 (17.1, 18.6)	21.1 (20.4, 21.9)	22.0 (21.3, 22.8)
urban-rural difference	0.2 (0.0, 0.4)	0.4 (0.2, 0.7)	0.3 (0.1, 0.5)	0.1 (-0.1, 0.3)	0.0 (-0.3, 0.2)	0.4 (0.1, 0.6)	0.6 (0.3, 0.9)	0.4 (0.2, 0.7)	0.3 (0.0, 0.5)	0.1 (-0.1, 0.4)

Girls	Mean BMI in 1990 (kg/m²) Age-standardised	5 years	10 years	15 years	19 years	Mean BMI in 2020 (kg/m²) Age-standardised	5 years	10 years	15 years	19 years
North Korea										
rural	17.6 (15.5, 19.7)	14.5 (12.1, 17.0)	16.2 (14.1, 18.2)	19.6 (17.5, 21.8)	20.7 (17.6, 23.7)	18.6 (16.5, 20.7)	15.6 (13.2, 18.0)	17.3 (15.2, 19.3)	20.7 (18.6, 22.8)	21.8 (18.7, 24.8)
urban	17.8 (15.7, 19.9)	14.9 (12.6, 17.3)	16.5 (14.4, 18.6)	19.8 (17.6, 21.9)	20.7 (17.7, 23.8)	19.0 (16.9, 21.0)	16.1 (13.7, 18.5)	17.6 (15.6, 19.7)	20.9 (18.8, 23.1)	21.9 (18.8, 25.0)
urban-rural difference	0.2 (-0.5, 0.9)	0.4 (-0.4, 1.2)	0.3 (-0.4, 1.0)	0.1 (-0.6, 0.9)	0.0 (-0.9, 1.0)	0.3 (-0.3, 1.0)	0.5 (-0.3, 1.3)	0.4 (-0.3, 1.0)	0.2 (-0.5, 1.0)	0.1 (-0.8, 1.0)
Taiwan										
rural	17.9 (16.9, 18.9)	14.4 (13.4, 15.5)	16.7 (15.7, 17.7)	20.0 (19.0, 21.0)	21.0 (19.7, 22.1)	18.9 (18.3, 19.5)	15.4 (14.7, 16.2)	17.7 (17.0, 18.4)	21.0 (20.4, 21.5)	21.9 (21.0, 22.9)
urban	18.0 (17.1, 19.0)	14.7 (13.7, 15.7)	16.9 (16.0, 17.8)	20.1 (19.1, 21.0)	21.0 (19.8, 22.1)	19.0 (18.4, 19.6)	15.6 (14.9, 16.4)	17.9 (17.2, 18.5)	21.0 (20.4, 21.6)	21.9 (21.0, 22.8)
urban-rural difference	0.2 (-0.5, 0.8)	0.3 (-0.5, 1.0)	0.2 (-0.4, 0.9)	0.1 (-0.6, 0.8)	0.0 (-0.7, 0.8)	0.1 (-0.3, 0.5)	0.2 (-0.4, 0.8)	0.1 (-0.3, 0.6)	0.1 (-0.4, 0.5)	0.0 (-0.6, 0.6)
Southeast Asia										
Brunei Darussalam										
rural	18.4 (16.9, 19.8)	14.7 (13.1, 16.2)	17.0 (15.5, 18.4)	20.7 (19.3, 22.1)	21.5 (19.7, 23.1)	20.2 (19.4, 20.9)	16.5 (15.5, 17.5)	18.8 (18.0, 19.6)	22.5 (21.8, 23.3)	23.3 (22.2, 24.4)
urban	18.7 (17.2, 20.1)	15.2 (13.6, 16.7)	17.4 (15.9, 18.8)	20.9 (19.4, 22.4)	21.5 (19.8, 23.2)	20.8 (20.1, 21.4)	17.3 (16.3, 18.3)	19.5 (18.8, 20.2)	23.1 (22.4, 23.7)	23.7 (22.6, 24.7)
urban-rural difference	0.3 (-0.3, 0.9)	0.5 (-0.2, 1.2)	0.3 (-0.2, 1.0)	0.2 (-0.5, 0.9)	0.1 (-0.7, 0.9)	0.6 (0.1, 1.1)	0.8 (0.2, 1.5)	0.7 (0.1, 1.2)	0.5 (-0.1, 1.1)	0.4 (-0.4, 1.1)
Cambodia										
rural	16.7 (15.4, 18.0)	13.5 (11.7, 15.3)	15.1 (13.8, 16.4)	18.9 (17.6, 20.1)	20.0 (18.8, 21.3)	17.5 (16.5, 18.5)	14.3 (12.7, 15.9)	15.9 (14.8, 17.0)	19.7 (18.7, 20.6)	20.9 (19.9, 21.8)
urban	16.8 (15.5, 18.1)	14.1 (12.3, 15.8)	15.4 (14.0, 16.7)	18.8 (17.6, 20.1)	19.7 (18.5, 21.0)	17.8 (16.8, 18.9)	15.1 (13.5, 16.8)	16.4 (15.3, 17.5)	19.9 (18.9, 20.8)	20.8 (19.8, 21.8)
urban-rural difference	0.1 (-0.3, 0.7)	0.6 (-0.1, 1.4)	0.3 (-0.3, 0.8)	0.0 (-0.5, 0.4)	-0.3 (-0.8, 0.2)	0.4 (-0.1, 0.8)	0.8 (0.1, 1.6)	0.5 (0.0, 1.0)	0.2 (-0.2, 0.6)	-0.1 (-0.5, 0.4)
Indonesia										
rural	17.2 (16.4, 18.0)	14.3 (13.6, 15.1)	15.7 (14.9, 16.4)	19.3 (18.5, 20.1)	20.2 (19.4, 21.0)	18.7 (18.1, 19.3)	15.9 (15.2, 16.5)	17.2 (16.6, 17.8)	20.8 (20.2, 21.4)	21.7 (21.1, 22.4)
urban	17.4 (16.7, 18.2)	14.6 (13.9, 15.4)	15.9 (15.2, 16.7)	19.5 (18.8, 20.3)	20.5 (19.7, 21.3)	19.1 (18.5, 19.7)	16.3 (15.7, 16.9)	17.6 (17.0, 18.2)	21.2 (20.6, 21.8)	22.2 (21.5, 22.8)
urban-rural difference	0.3 (-0.1, 0.6)	0.3 (-0.1, 0.6)	0.3 (-0.1, 0.6)	0.3 (-0.1, 0.6)	0.2 (-0.1, 0.6)	0.4 (0.1, 0.7)	0.4 (0.1, 0.7)	0.4 (0.1, 0.7)	0.4 (0.1, 0.7)	0.4 (0.1, 0.7)
Lao PDR										
rural	16.9 (15.5, 18.4)	13.8 (11.9, 15.7)	15.4 (13.9, 17.0)	19.1 (17.7, 20.5)	20.3 (18.8, 21.8)	17.8 (16.6, 19.1)	14.7 (12.8, 16.5)	16.3 (14.9, 17.7)	20.0 (18.8, 21.2)	21.2 (19.9, 22.4)
urban	17.0 (15.6, 18.4)	14.2 (12.3, 16.1)	15.6 (14.1, 17.1)	19.0 (17.6, 20.5)	20.0 (18.5, 21.6)	18.1 (16.8, 19.4)	15.3 (13.5, 17.1)	16.7 (15.3, 18.1)	20.1 (18.9, 21.4)	21.2 (19.8, 22.4)
urban-rural difference	0.1 (-0.5, 0.7)	0.4 (-0.4, 1.1)	0.2 (-0.5, 0.7)	-0.1 (-0.7, 0.5)	-0.3 (-1.0, 0.5)	0.3 (-0.3, 0.9)	0.6 (-0.1, 1.4)	0.4 (-0.2, 1.0)	0.2 (-0.4, 0.7)	0.0 (-0.8, 0.7)
Malaysia										
rural	17.6 (16.6, 18.7)	13.5 (12.4, 14.6)	16.3 (15.2, 17.4)	20.1 (19.0, 21.2)	21.1 (20.1, 22.2)	19.3 (18.7, 19.8)	15.2 (14.6, 15.8)	17.9 (17.4, 18.5)	21.7 (21.2, 22.3)	22.7 (22.1, 23.4)
urban	17.8 (16.7, 18.8)	13.9 (12.8, 15.0)	16.5 (15.4, 17.5)	20.1 (19.1, 21.1)	20.9 (20.0, 22.0)	19.6 (19.0, 20.1)	15.7 (15.1, 16.3)	18.3 (17.7, 18.8)	21.9 (21.4, 22.5)	22.8 (22.1, 23.4)
urban-rural difference	0.1 (-0.3, 0.6)	0.4 (-0.1, 0.9)	0.2 (-0.2, 0.7)	0.0 (-0.4, 0.5)	-0.1 (-0.6, 0.4)	0.3 (0.0, 0.6)	0.5 (0.2, 0.9)	0.4 (0.1, 0.6)	0.2 (-0.2, 0.5)	0.0 (-0.4, 0.4)
Maldives										
rural	17.0 (15.5, 18.4)	13.8 (11.9, 15.7)	15.5 (14.0, 17.0)	19.1 (17.7, 20.5)	20.3 (18.8, 21.9)	18.2 (17.2, 19.1)	15.0 (13.5, 16.6)	16.7 (15.6, 17.8)	20.3 (19.4, 21.1)	21.5 (20.5, 22.5)
urban	17.0 (15.6, 18.5)	14.3 (12.4, 16.2)	15.7 (14.2, 17.2)	19.0 (17.6, 20.4)	20.0 (18.4, 21.6)	18.4 (17.4, 19.4)	15.6 (14.1, 17.3)	17.1 (15.9, 18.2)	20.4 (19.4, 21.2)	21.4 (20.3, 22.4)
urban-rural difference	0.1 (-0.5, 0.7)	0.5 (-0.3, 1.3)	0.2 (-0.4, 0.9)	-0.1 (-0.7, 0.6)	-0.3 (-1.1, 0.5)	0.2 (-0.3, 0.8)	0.6 (-0.1, 1.4)	0.4 (-0.2, 0.9)	0.1 (-0.5, 0.6)	-0.1 (-0.8, 0.5)
Myanmar										
rural	16.7 (15.3, 18.1)	13.7 (11.7, 15.5)	15.3 (13.9, 16.8)	18.8 (17.4, 20.2)	19.5 (18.1, 20.9)	17.8 (16.8, 18.8)	14.8 (13.2, 16.4)	16.5 (15.4, 17.6)	19.9 (19.1, 20.8)	20.6 (19.7, 21.5)
urban	16.9 (15.5, 18.3)	14.1 (12.2, 16.1)	15.6 (14.1, 17.1)	18.9 (17.5, 20.3)	19.5 (18.0, 20.9)	18.3 (17.4, 19.3)	15.5 (13.9, 17.2)	17.1 (16.0, 18.2)	20.3 (19.5, 21.2)	20.9 (20.0, 21.8)
urban-rural difference	0.2 (-0.3, 0.8)	0.5 (-0.3, 1.2)	0.3 (-0.3, 0.9)	0.1 (-0.5, 0.7)	0.0 (-0.7, 0.7)	0.5 (0.1, 1.0)	0.8 (0.0, 1.5)	0.6 (0.1, 1.1)	0.4 (0.0, 0.9)	0.3 (-0.2, 0.8)
Philippines										
rural	17.0 (16.1, 17.9)	14.2 (13.2, 15.1)	15.4 (14.5, 16.3)	19.0 (18.1, 20.0)	20.2 (19.2, 21.1)	17.8 (17.3, 18.3)	15.0 (14.5, 15.6)	16.2 (15.7, 16.8)	19.9 (19.3, 20.4)	21.0 (20.4, 21.6)
urban	17.1 (16.2, 18.1)	14.5 (13.6, 15.5)	15.6 (14.7, 16.6)	19.1 (18.2, 20.1)	20.2 (19.2, 21.1)	18.3 (17.7, 18.8)	15.7 (15.1, 16.2)	16.7 (16.2, 17.3)	20.3 (19.7, 20.8)	21.3 (20.7, 21.9)
urban-rural difference	0.2 (-0.3, 0.6)	0.3 (-0.1, 0.8)	0.2 (-0.2, 0.6)	0.1 (-0.4, 0.5)	0.0 (-0.5, 0.4)	0.5 (0.2, 0.8)	0.6 (0.3, 1.0)	0.5 (0.2, 0.8)	0.4 (0.1, 0.7)	0.3 (-0.1, 0.6)
Thailand										
rural	17.3 (16.7, 17.9)	13.9 (13.3, 14.5)	15.8 (15.3, 16.4)	19.5 (18.9, 20.1)	20.7 (20.1, 21.4)	18.8 (18.3, 19.3)	15.4 (14.8, 15.9)	17.3 (16.8, 17.8)	21.0 (20.5, 21.5)	22.2 (21.6, 22.8)
urban	17.6 (17.1, 18.2)	14.5 (13.9, 15.1)	16.2 (15.7, 16.8)	19.7 (19.1, 20.2)	20.8 (20.1, 21.4)	19.2 (18.7, 19.7)	16.1 (15.5, 16.7)	17.9 (17.3, 18.4)	21.3 (20.8, 21.8)	22.4 (21.8, 23.0)
urban-rural difference	0.3 (0.0, 0.6)	0.6 (0.2, 1.0)	0.4 (0.1, 0.7)	0.2 (-0.2, 0.5)	0.0 (-0.4, 0.5)	0.4 (0.1, 0.8)	0.7 (0.3, 1.1)	0.5 (0.1, 0.9)	0.3 (-0.1, 0.7)	0.2 (-0.3, 0.6)
Timor-Leste										
rural	16.1 (14.7, 17.6)	13.7 (12.3, 15.2)	14.5 (13.2, 16.0)	18.1 (16.8, 19.6)	19.1 (17.7, 20.6)	16.6 (16.0, 17.3)	14.2 (13.4, 15.0)	15.1 (14.3, 15.7)	18.6 (17.9, 19.3)	19.6 (18.9, 20.3)
urban	16.2 (14.9, 17.7)	14.1 (12.6, 15.6)	14.7 (13.3, 16.2)	18.1 (16.7, 19.5)	18.9 (17.5, 20.4)	17.0 (16.2, 17.7)	14.9 (13.9, 15.9)	15.5 (14.7, 16.3)	18.9 (18.1, 19.6)	19.7 (18.9, 20.4)
urban-rural difference	0.1 (-0.5, 0.7)	0.4 (-0.3, 1.2)	0.2 (-0.4, 0.8)	0.0 (-0.6, 0.6)	-0.2 (-0.8, 0.4)	0.4 (-0.1, 0.8)	0.7 (0.0, 1.4)	0.4 (-0.1, 0.9)	0.2 (-0.2, 0.6)	0.0 (-0.4, 0.5)
Viet Nam										
rural	15.9 (15.3, 16.4)	13.5 (13.0, 14.1)	14.3 (13.8, 14.9)	17.7 (17.2, 18.3)	19.0 (18.5, 19.6)	16.8 (16.3, 17.4)	14.5 (13.9, 15.1)	15.3 (14.7, 15.9)	18.7 (18.1, 19.3)	20.0 (19.4, 20.6)

Girls	Mean BMI in 1990 (kg/m^2)					Mean BMI in 2020 (kg/m^2)				
	Age-standardised	5 years	10 years	15 years	19 years	Age-standardised	5 years	10 years	15 years	19 years
urban	16.0 (15.5, 16.6)	14.0 (13.4, 14.5)	14.5 (14.0, 15.1)	17.7 (17.2, 18.3)	18.9 (18.3, 19.5)	17.7 (17.1, 18.2)	15.6 (15.0, 16.2)	16.2 (15.6, 16.8)	19.4 (18.8, 20.0)	20.5 (19.9, 21.1)
urban-rural difference	0.1 (-0.2, 0.5)	0.4 (0.0, 0.8)	0.2 (-0.1, 0.5)	0.0 (-0.3, 0.4)	-0.1 (-0.5, 0.2)	0.8 (0.5, 1.2)	1.1 (0.7, 1.5)	0.9 (0.6, 1.3)	0.7 (0.4, 1.1)	0.6 (0.2, 0.9)
**High-income Asia Pacific**										
Japan										
rural	18.5 (18.3, 18.8)	15.3 (15.1, 15.5)	17.3 (17.1, 17.5)	20.8 (20.6, 21.0)	20.8 (20.5, 21.1)	18.7 (18.4, 19.0)	15.5 (15.2, 15.8)	17.5 (17.2, 17.7)	21.0 (20.7, 21.2)	21.0 (20.7, 21.3)
urban	18.4 (18.2, 18.6)	15.4 (15.2, 15.6)	17.2 (17.0, 17.4)	20.6 (20.3, 20.8)	20.4 (20.2, 20.7)	18.5 (18.3, 18.8)	15.5 (15.3, 15.8)	17.4 (17.1, 17.6)	20.7 (20.4, 21.0)	20.6 (20.3, 20.9)
urban-rural difference	-0.1 (-0.3, 0.0)	0.1 (-0.1, 0.2)	-0.1 (-0.2, 0.1)	-0.2 (-0.4, -0.1)	-0.4 (-0.5, -0.2)	-0.2 (-0.3, -0.1)	0.0 (-0.1, 0.2)	-0.1 (-0.3, 0.0)	-0.3 (-0.4, -0.2)	-0.4 (-0.6, -0.3)
Singapore										
rural	18.2 (16.4, 19.9)	15.0 (12.8, 17.1)	17.0 (15.1, 18.8)	20.3 (18.5, 22.0)	20.8 (18.8, 22.9)	19.0 (17.4, 20.6)	15.8 (13.7, 17.8)	17.8 (16.0, 19.6)	21.2 (19.5, 22.7)	21.7 (19.8, 23.5)
urban	18.3 (16.5, 20.0)	15.1 (12.9, 17.3)	17.1 (15.2, 19.0)	20.4 (18.7, 22.1)	20.9 (18.9, 22.9)	19.1 (17.6, 20.6)	15.9 (13.8, 17.9)	17.9 (16.2, 19.6)	21.2 (19.7, 22.7)	21.7 (20.1, 23.3)
urban-rural difference	0.1 (-0.6, 0.8)	0.2 (-0.7, 1.0)	0.1 (-0.6, 0.8)	0.1 (-0.7, 0.8)	0.1 (-0.8, 1.0)	0.1 (-0.6, 0.8)	0.1 (-0.7, 1.0)	0.1 (-0.6, 0.8)	0.1 (-0.6, 0.8)	0.0 (-0.8, 0.9)
South Korea										
rural	18.3 (17.3, 19.3)	15.2 (14.2, 16.1)	17.3 (16.3, 18.3)	20.2 (19.3, 21.2)	20.6 (19.6, 21.6)	19.2 (18.8, 19.5)	16.0 (15.6, 16.4)	18.2 (17.8, 18.5)	21.1 (20.7, 21.5)	21.5 (21.1, 21.9)
urban	18.6 (17.6, 19.5)	15.2 (14.2, 16.1)	17.5 (16.6, 18.5)	20.6 (19.7, 21.6)	21.1 (20.1, 22.1)	19.3 (19.0, 19.7)	16.0 (15.6, 16.3)	18.3 (18.0, 18.6)	21.4 (21.1, 21.7)	21.9 (21.5, 22.3)
urban-rural difference	0.3 (-0.1, 0.6)	0.0 (-0.4, 0.4)	0.2 (-0.2, 0.6)	0.4 (0.0, 0.7)	0.5 (0.1, 0.9)	0.2 (0.0, 0.4)	-0.1 (-0.3, 0.2)	0.1 (-0.1, 0.3)	0.3 (0.0, 0.5)	0.4 (0.1, 0.7)
**High-income western**										
High-income English-speaking countries										
Australia										
rural	19.6 (18.8, 20.5)	15.7 (14.8, 16.6)	18.3 (17.4, 19.1)	21.9 (21.1, 22.8)	23.2 (22.3, 24.2)	20.6 (20.1, 21.1)	16.6 (16.0, 17.2)	19.2 (18.6, 19.7)	22.9 (22.3, 23.4)	24.1 (23.5, 24.8)
urban	19.5 (18.7, 20.4)	15.6 (14.7, 16.4)	18.1 (17.3, 19.0)	21.8 (21.0, 22.7)	23.1 (22.2, 24.1)	20.7 (20.2, 21.2)	16.8 (16.2, 17.4)	19.3 (18.8, 19.9)	23.1 (22.6, 23.6)	24.4 (23.8, 25.0)
urban-rural difference	-0.1 (-0.5, 0.3)	-0.2 (-0.6, 0.3)	-0.1 (-0.5, 0.3)	-0.1 (-0.5, 0.4)	-0.1 (-0.6, 0.5)	0.2 (-0.1, 0.5)	0.1 (-0.2, 0.5)	0.2 (-0.1, 0.4)	0.2 (-0.1, 0.5)	0.2 (-0.2, 0.6)
Canada										
rural	19.0 (18.2, 19.8)	15.0 (14.1, 16.0)	17.5 (16.7, 18.4)	21.4 (20.6, 22.2)	22.8 (22.0, 23.7)	19.7 (19.0, 20.4)	15.7 (14.9, 16.5)	18.2 (17.5, 18.9)	22.0 (21.3, 22.8)	23.5 (22.7, 24.4)
urban	18.7 (18.0, 19.5)	14.8 (14.0, 15.6)	17.3 (16.5, 18.0)	21.1 (20.3, 21.8)	22.5 (21.7, 23.3)	19.7 (19.2, 20.2)	15.8 (15.2, 16.3)	18.2 (17.7, 18.7)	22.0 (21.5, 22.6)	23.5 (22.8, 24.1)
urban-rural difference	-0.3 (-0.8, 0.3)	-0.2 (-1.0, 0.6)	-0.3 (-0.8, 0.3)	-0.3 (-0.9, 0.3)	-0.4 (-1.0, 0.3)	0.0 (-0.6, 0.6)	0.1 (-0.7, 0.8)	0.0 (-0.6, 0.6)	0.0 (-0.6, 0.6)	-0.1 (-0.8, 0.7)
Ireland										
rural	18.9 (17.7, 20.0)	15.4 (14.2, 16.7)	17.5 (16.3, 18.7)	21.1 (19.8, 22.3)	22.1 (20.8, 23.3)	19.5 (19.1, 19.9)	16.1 (15.6, 16.5)	18.1 (17.7, 18.5)	21.7 (21.2, 22.2)	22.7 (21.9, 23.5)
urban	18.9 (17.7, 20.0)	15.2 (14.0, 16.4)	17.4 (16.3, 18.6)	21.2 (19.9, 22.3)	22.3 (21.0, 23.5)	19.8 (19.4, 20.1)	16.1 (15.7, 16.6)	18.3 (17.9, 18.7)	22.0 (21.6, 22.5)	23.2 (22.4, 23.9)
urban-rural difference	0.0 (-0.5, 0.5)	-0.2 (-0.8, 0.3)	-0.1 (-0.5, 0.4)	0.1 (-0.4, 0.6)	0.2 (-0.4, 0.9)	0.3 (0.0, 0.6)	0.1 (-0.3, 0.4)	0.2 (-0.1, 0.5)	0.4 (0.0, 0.7)	0.5 (-0.1, 1.0)
New Zealand										
rural	19.0 (18.3, 19.7)	15.0 (14.3, 15.7)	17.6 (16.8, 18.3)	21.4 (20.7, 22.1)	22.4 (21.6, 23.2)	20.3 (19.9, 20.6)	16.3 (15.9, 16.7)	18.8 (18.4, 19.2)	22.7 (22.2, 23.1)	23.7 (23.1, 24.3)
urban	19.2 (18.6, 19.8)	14.7 (14.1, 15.3)	17.6 (17.0, 18.2)	21.8 (21.2, 22.4)	23.1 (22.5, 23.8)	21.1 (20.7, 21.4)	16.6 (16.2, 17.0)	19.5 (19.2, 19.9)	23.7 (23.4, 24.1)	25.0 (24.5, 25.6)
urban-rural difference	0.2 (-0.3, 0.7)	-0.3 (-0.9, 0.2)	0.1 (-0.5, 0.5)	0.4 (-0.1, 0.9)	0.7 (0.1, 1.3)	0.8 (0.6, 1.1)	0.3 (0.0, 0.6)	0.7 (0.4, 1.0)	1.1 (0.7, 1.4)	1.4 (0.9, 1.8)
United Kingdom										
rural	19.3 (18.8, 19.8)	15.5 (15.0, 16.1)	17.9 (17.4, 18.4)	21.5 (21.0, 22.1)	22.8 (22.3, 23.3)	19.7 (19.3, 20.1)	16.0 (15.6, 16.4)	18.3 (17.9, 18.7)	21.9 (21.5, 22.3)	23.2 (22.8, 23.6)
urban	19.3 (18.8, 19.7)	15.3 (14.9, 15.8)	17.8 (17.4, 18.3)	21.6 (21.1, 22.1)	23.0 (22.5, 23.5)	20.2 (19.8, 20.5)	16.2 (15.9, 16.6)	18.7 (18.3, 19.1)	22.5 (22.1, 22.9)	23.9 (23.5, 24.3)
urban-rural difference	0.0 (-0.2, 0.2)	-0.2 (-0.4, 0.0)	-0.1 (-0.3, 0.2)	0.1 (-0.2, 0.3)	0.2 (-0.1, 0.5)	0.5 (0.3, 0.7)	0.3 (0.1, 0.5)	0.4 (0.3, 0.6)	0.6 (0.4, 0.8)	0.7 (0.4, 0.9)
United States of America										
rural	20.1 (19.6, 20.6)	15.3 (14.7, 15.8)	18.7 (18.2, 19.3)	22.7 (22.2, 23.3)	24.2 (23.5, 24.8)	21.4 (20.9, 21.9)	16.6 (16.0, 17.1)	20.0 (19.5, 20.6)	24.0 (23.5, 24.6)	25.4 (24.8, 26.1)
urban	20.0 (19.4, 20.5)	15.2 (14.6, 15.7)	18.6 (18.1, 19.2)	22.6 (22.0, 23.1)	24.0 (23.4, 24.6)	21.3 (20.8, 21.8)	16.5 (16.0, 17.0)	20.0 (19.5, 20.5)	23.9 (23.4, 24.5)	25.3 (24.8, 25.9)
urban-rural difference	-0.1 (-0.4, 0.2)	-0.1 (-0.4, 0.2)	-0.1 (-0.4, 0.2)	-0.1 (-0.5, 0.2)	-0.2 (-0.5, 0.2)	-0.1 (-0.3, 0.2)	0.0 (-0.3, 0.2)	-0.1 (-0.3, 0.2)	-0.1 (-0.4, 0.2)	-0.1 (-0.5, 0.3)
Northwestern Europe										
Austria										
rural	18.1 (17.0, 19.2)	14.3 (13.2, 15.5)	17.0 (15.9, 18.1)	20.4 (19.3, 21.5)	20.8 (19.7, 21.9)	18.9 (18.4, 19.5)	15.1 (14.5, 15.7)	17.8 (17.3, 18.4)	21.2 (20.7, 21.8)	21.6 (20.8, 22.5)
urban	18.4 (17.2, 19.5)	14.3 (13.1, 15.4)	17.2 (16.0, 18.3)	20.8 (19.6, 21.9)	21.3 (20.2, 22.4)	19.4 (18.8, 19.9)	15.3 (14.7, 15.9)	18.2 (17.6, 18.7)	21.8 (21.2, 22.3)	22.3 (21.5, 23.2)
urban-rural difference	0.2 (-0.4, 0.8)	0.0 (-0.7, 0.6)	0.1 (-0.4, 0.7)	0.3 (-0.3, 0.9)	0.5 (-0.2, 1.1)	0.4 (0.1, 0.8)	0.2 (-0.3, 0.6)	0.4 (0.0, 0.7)	0.5 (0.2, 0.9)	0.7 (0.2, 1.2)
Belgium										
rural	18.9 (17.7, 20.1)	15.6 (14.2, 17.0)	17.9 (16.6, 19.1)	20.7 (19.4, 21.9)	22.2 (20.9, 23.5)	19.8 (18.9, 20.7)	16.6 (15.6, 17.6)	18.8 (17.9, 19.7)	21.6 (20.7, 22.6)	23.2 (22.2, 24.2)
urban	18.8 (17.6, 20.1)	15.1 (13.8, 16.4)	17.6 (16.4, 18.9)	20.8 (19.5, 22.1)	22.6 (21.3, 24.1)	19.7 (19.1, 20.5)	16.0 (15.2, 16.8)	18.6 (17.8, 19.3)	21.7 (21.0, 22.4)	23.6 (22.7, 24.4)
urban-rural difference	-0.1 (-0.7, 0.5)	-0.6 (-1.3, 0.2)	-0.2 (-0.9, 0.4)	0.1 (-0.5, 0.8)	0.4 (-0.4, 1.2)	-0.1 (-0.7, 0.5)	-0.6 (-1.4, 0.1)	-0.2 (-0.9, 0.3)	0.1 (-0.5, 0.7)	0.4 (-0.4, 1.1)
Denmark										

Girls	Mean BMI in 1990 (kg/m^2)					Mean BMI in 2020 (kg/m^2)				
	Age-standardised	5 years	10 years	15 years	19 years	Age-standardised	5 years	10 years	15 years	19 years
rural	18.8 (17.9, 19.6)	15.8 (14.9, 16.7)	17.8 (16.9, 18.6)	20.6 (19.7, 21.5)	21.4 (20.3, 22.5)	18.6 (18.1, 19.2)	15.6 (15.0, 16.2)	17.6 (17.1, 18.2)	20.5 (19.8, 21.1)	21.2 (20.3, 22.2)
urban	18.8 (18.1, 19.5)	15.4 (14.7, 16.1)	17.7 (17.0, 18.3)	20.8 (20.1, 21.5)	21.8 (20.8, 22.8)	18.6 (18.1, 19.2)	15.2 (14.7, 15.8)	17.5 (17.0, 18.1)	20.6 (20.0, 21.2)	21.6 (20.7, 22.5)
urban-rural difference	0.0 (-0.5, 0.6)	-0.4 (-1.0, 0.3)	-0.1 (-0.7, 0.5)	0.2 (-0.4, 0.8)	0.4 (-0.3, 1.1)	0.0 (-0.4, 0.4)	-0.4 (-0.8, 0.0)	-0.1 (-0.5, 0.2)	0.2 (-0.3, 0.6)	0.4 (-0.2, 1.0)
Finland										
rural	19.1 (17.7, 20.5)	15.2 (13.7, 16.6)	17.6 (16.2, 18.9)	21.4 (20.0, 22.7)	23.4 (21.7, 25.0)	20.2 (19.6, 20.8)	16.2 (15.6, 16.8)	18.6 (18.1, 19.2)	22.4 (21.8, 23.0)	24.4 (23.4, 25.4)
urban	19.0 (17.6, 20.4)	15.1 (13.7, 16.5)	17.5 (16.1, 18.9)	21.2 (19.9, 22.6)	23.2 (21.5, 24.8)	20.0 (19.5, 20.5)	16.1 (15.6, 16.7)	18.5 (18.0, 19.0)	22.3 (21.7, 22.8)	24.2 (23.2, 25.2)
urban-rural difference	-0.1 (-0.7, 0.5)	-0.1 (-0.7, 0.6)	-0.1 (-0.7, 0.5)	-0.1 (-0.8, 0.5)	-0.2 (-0.8, 0.5)	-0.1 (-0.6, 0.3)	-0.1 (-0.6, 0.3)	-0.1 (-0.6, 0.3)	-0.2 (-0.6, 0.3)	-0.2 (-0.7, 0.3)
Germany										
rural	19.0 (17.8, 20.2)	15.2 (14.0, 16.5)	17.8 (16.6, 19.1)	21.2 (20.0, 22.4)	21.8 (20.5, 23.0)	19.2 (18.5, 20.0)	15.5 (14.7, 16.3)	18.1 (17.3, 18.8)	21.4 (20.6, 22.2)	22.0 (21.0, 23.0)
urban	19.1 (17.9, 20.2)	15.0 (13.8, 16.3)	17.8 (16.6, 19.0)	21.4 (20.2, 22.6)	22.2 (20.9, 23.4)	19.5 (18.8, 20.2)	15.4 (14.7, 16.2)	18.3 (17.5, 19.0)	21.8 (21.1, 22.6)	22.6 (21.6, 23.5)
urban-rural difference	0.1 (-0.4, 0.6)	-0.2 (-0.8, 0.4)	0.0 (-0.5, 0.5)	0.2 (-0.3, 0.7)	0.4 (-0.2, 1.0)	0.3 (-0.1, 0.7)	0.0 (-0.5, 0.4)	0.2 (-0.2, 0.6)	0.4 (0.0, 0.9)	0.6 (0.0, 1.1)
Greenland										
rural	19.6 (18.0, 21.3)	16.1 (14.0, 18.1)	18.3 (16.5, 20.1)	21.7 (20.0, 23.4)	23.2 (21.4, 25.0)	20.3 (18.7, 21.8)	16.7 (14.7, 18.6)	18.9 (17.2, 20.6)	22.3 (20.7, 23.9)	23.8 (22.2, 25.4)
urban	19.7 (18.0, 21.4)	15.9 (13.8, 17.9)	18.3 (16.5, 20.1)	21.9 (20.1, 23.6)	23.5 (21.7, 25.4)	20.4 (18.8, 22.0)	16.6 (14.7, 18.6)	19.0 (17.3, 20.7)	22.6 (21.0, 24.2)	24.2 (22.5, 25.9)
urban-rural difference	0.1 (-0.6, 0.7)	-0.2 (-1.0, 0.6)	0.0 (-0.7, 0.7)	0.2 (-0.5, 0.9)	0.3 (-0.5, 1.2)	0.2 (-0.4, 0.8)	-0.1 (-0.9, 0.7)	0.1 (-0.5, 0.7)	0.3 (-0.4, 0.9)	0.4 (-0.4, 1.2)
Iceland										
rural	18.8 (16.8, 20.8)	15.4 (13.2, 17.6)	17.7 (15.7, 19.5)	20.8 (18.8, 22.8)	21.9 (19.1, 24.8)	19.4 (17.5, 21.3)	16.0 (13.8, 18.1)	18.2 (16.3, 20.1)	21.4 (19.4, 23.4)	22.5 (19.7, 25.3)
urban	18.9 (16.9, 20.8)	15.2 (13.1, 17.4)	17.6 (15.7, 19.5)	21.0 (19.0, 22.9)	22.2 (19.4, 25.0)	19.6 (17.6, 21.5)	15.9 (13.8, 18.0)	18.3 (16.5, 20.2)	21.7 (19.7, 23.6)	22.9 (20.1, 25.7)
urban-rural difference	0.0 (-0.6, 0.7)	-0.2 (-1.0, 0.6)	0.0 (-0.7, 0.6)	0.1 (-0.6, 0.9)	0.2 (-0.7, 1.2)	0.1 (-0.5, 0.8)	-0.1 (-0.9, 0.7)	0.1 (-0.6, 0.7)	0.2 (-0.5, 0.9)	0.4 (-0.5, 1.2)
Luxembourg										
rural	18.7 (17.1, 20.4)	15.4 (13.3, 17.3)	17.7 (16.0, 19.4)	20.7 (19.1, 22.4)	21.4 (19.7, 23.0)	19.3 (17.8, 20.8)	15.9 (14.0, 17.9)	18.2 (16.6, 19.8)	21.3 (19.8, 22.8)	22.0 (20.4, 23.5)
urban	18.8 (17.2, 20.4)	15.2 (13.1, 17.2)	17.7 (16.0, 19.3)	21.0 (19.4, 22.5)	21.8 (20.1, 23.4)	19.5 (18.1, 21.0)	15.9 (14.0, 17.8)	18.4 (16.8, 20.0)	21.6 (20.3, 23.1)	22.5 (21.0, 24.0)
urban-rural difference	0.1 (-0.5, 0.7)	-0.2 (-1.0, 0.6)	0.0 (-0.6, 0.7)	0.2 (-0.4, 0.9)	0.4 (-0.4, 1.2)	0.2 (-0.4, 0.8)	-0.1 (-0.8, 0.7)	0.1 (-0.5, 0.7)	0.3 (-0.3, 1.0)	0.5 (-0.2, 1.3)
Netherlands										
rural	18.6 (17.3, 19.9)	15.5 (14.1, 16.9)	17.3 (16.0, 18.6)	20.4 (19.1, 21.8)	22.0 (20.4, 23.5)	18.9 (18.0, 19.8)	15.8 (14.7, 16.9)	17.6 (16.7, 18.5)	20.8 (19.8, 21.7)	22.3 (21.0, 23.5)
urban	18.6 (17.4, 19.9)	15.3 (14.0, 16.8)	17.3 (16.0, 18.6)	20.6 (19.3, 21.9)	22.2 (20.8, 23.6)	19.0 (18.1, 19.9)	15.7 (14.6, 16.8)	17.7 (16.7, 18.6)	20.9 (20.0, 21.9)	22.6 (21.4, 23.8)
urban-rural difference	0.1 (-0.5, 0.6)	-0.1 (-0.8, 0.5)	0.0 (-0.5, 0.6)	0.1 (-0.5, 0.8)	0.2 (-0.5, 1.0)	0.1 (-0.3, 0.5)	-0.1 (-0.7, 0.5)	0.0 (-0.4, 0.5)	0.2 (-0.3, 0.7)	0.3 (-0.4, 1.0)
Norway										
rural	18.9 (17.7, 20.1)	15.7 (14.3, 17.2)	17.5 (16.3, 18.7)	20.8 (19.6, 22.0)	22.5 (21.3, 23.8)	19.4 (18.4, 20.3)	16.2 (15.0, 17.4)	18.0 (17.0, 18.9)	21.3 (20.2, 22.3)	23.0 (21.9, 24.1)
urban	18.7 (17.5, 19.9)	15.5 (14.2, 16.9)	17.3 (16.1, 18.5)	20.6 (19.4, 21.9)	22.4 (21.1, 23.8)	19.3 (18.4, 20.1)	16.1 (15.1, 17.1)	17.9 (16.9, 18.7)	21.2 (20.2, 22.1)	23.0 (21.8, 24.1)
urban-rural difference	-0.2 (-0.7, 0.4)	-0.2 (-0.9, 0.6)	-0.2 (-0.8, 0.4)	-0.1 (-0.8, 0.5)	-0.1 (-0.9, 0.6)	-0.1 (-0.6, 0.4)	-0.1 (-0.8, 0.6)	-0.1 (-0.7, 0.4)	-0.1 (-0.7, 0.5)	-0.1 (-0.8, 0.7)
Sweden										
rural	19.1 (18.6, 19.7)	15.8 (15.1, 16.5)	17.9 (17.4, 18.5)	21.1 (20.5, 21.7)	22.3 (21.2, 23.5)	19.6 (18.9, 20.3)	16.3 (15.5, 17.1)	18.4 (17.7, 19.1)	21.6 (20.8, 22.4)	22.8 (21.5, 24.1)
urban	19.0 (18.8, 19.3)	15.5 (15.1, 15.9)	17.8 (17.5, 18.1)	21.1 (20.8, 21.4)	22.4 (21.5, 23.4)	19.6 (19.0, 20.2)	16.1 (15.5, 16.6)	18.3 (17.8, 18.9)	21.7 (21.0, 22.3)	23.0 (21.9, 24.1)
urban-rural difference	-0.1 (-0.7, 0.5)	-0.3 (-1.0, 0.4)	-0.1 (-0.7, 0.5)	0.0 (-0.7, 0.7)	0.1 (-0.7, 1.0)	0.0 (-0.6, 0.5)	-0.2 (-0.9, 0.4)	-0.1 (-0.6, 0.4)	0.1 (-0.6, 0.7)	0.2 (-0.6, 1.0)
Switzerland										
rural	18.4 (17.1, 19.7)	15.4 (14.1, 16.7)	17.5 (16.3, 18.8)	20.2 (18.9, 21.5)	20.6 (19.0, 22.1)	18.6 (18.0, 19.2)	15.6 (15.0, 16.2)	17.8 (17.2, 18.4)	20.4 (19.7, 21.1)	20.8 (19.8, 21.8)
urban	18.5 (17.2, 19.8)	15.1 (13.8, 16.4)	17.6 (16.3, 18.8)	20.5 (19.2, 21.8)	21.1 (19.6, 22.6)	19.0 (18.4, 19.6)	15.6 (15.0, 16.2)	18.0 (17.5, 18.6)	21.0 (20.3, 21.7)	21.6 (20.6, 22.6)
urban-rural difference	0.1 (-0.4, 0.6)	-0.2 (-0.8, 0.3)	0.0 (-0.5, 0.5)	0.3 (-0.2, 0.8)	0.5 (-0.1, 1.2)	0.4 (0.0, 0.7)	0.0 (-0.4, 0.4)	0.3 (0.0, 0.6)	0.6 (0.1, 1.0)	0.8 (0.2, 1.3)
Southwestern Europe										
Andorra										
rural	19.1 (17.2, 21.0)	15.6 (13.4, 17.9)	18.1 (16.2, 19.9)	21.1 (19.1, 23.1)	21.8 (19.1, 24.6)	19.6 (17.7, 21.4)	16.1 (13.9, 18.3)	18.5 (16.7, 20.4)	21.6 (19.6, 23.5)	22.3 (19.6, 25.1)
urban	19.0 (17.1, 21.0)	15.5 (13.3, 17.7)	18.0 (16.1, 19.8)	21.1 (19.1, 23.1)	21.9 (19.1, 24.7)	19.8 (17.8, 21.6)	16.2 (14.0, 18.3)	18.7 (16.9, 20.5)	21.8 (19.8, 23.7)	22.6 (19.8, 25.3)
urban-rural difference	0.0 (-0.7, 0.6)	-0.1 (-0.9, 0.6)	-0.1 (-0.7, 0.6)	0.0 (-0.7, 0.7)	0.1 (-0.8, 0.9)	0.2 (-0.4, 0.8)	0.1 (-0.7, 0.8)	0.2 (-0.4, 0.8)	0.2 (-0.4, 0.9)	0.3 (-0.6, 1.1)
Cyprus										
rural	19.0 (17.6, 20.4)	15.1 (13.6, 16.5)	18.3 (16.9, 19.7)	21.1 (19.7, 22.5)	21.1 (19.3, 22.9)	19.5 (18.8, 20.2)	15.6 (14.8, 16.4)	18.8 (18.1, 19.5)	21.6 (20.8, 22.4)	21.6 (20.1, 23.1)
urban	18.9 (17.6, 20.3)	14.9 (13.5, 16.3)	18.2 (16.8, 19.5)	21.1 (19.7, 22.4)	21.2 (19.3, 23.0)	19.7 (19.1, 20.4)	15.7 (15.0, 16.4)	19.0 (18.4, 19.6)	21.9 (21.1, 22.6)	22.0 (20.5, 23.5)
urban-rural difference	-0.1 (-0.7, 0.5)	-0.2 (-0.9, 0.5)	-0.1 (-0.7, 0.4)	0.0 (-0.6, 0.6)	0.1 (-0.7, 0.8)	0.2 (-0.2, 0.7)	0.1 (-0.5, 0.7)	0.2 (-0.2, 0.6)	0.3 (-0.2, 0.8)	0.4 (-0.3, 1.0)
France										
rural	18.3 (17.0, 19.5)	15.0 (13.7, 16.3)	17.3 (16.0, 18.6)	20.1 (18.8, 21.4)	21.1 (19.7, 22.5)	18.7 (18.0, 19.3)	15.4 (14.7, 16.1)	17.7 (17.0, 18.3)	20.5 (19.9, 21.2)	21.5 (20.7, 22.3)
urban	18.4 (17.1, 19.7)	14.9 (13.5, 16.2)	17.3 (16.0, 18.6)	20.4 (19.1, 21.7)	21.5 (20.2, 22.9)	19.1 (18.5, 19.7)	15.5 (14.8, 16.2)	18.0 (17.4, 18.7)	21.1 (20.4, 21.7)	22.2 (21.6, 22.9)

Girls	Mean BMI in 1990 (kg/m^2)					Mean BMI in 2020 (kg/m^2)				
	Age-standardised	5 years	10 years	15 years	19 years	Age-standardised	5 years	10 years	15 years	19 years
urban-rural difference	0.1 (-0.4, 0.6)	-0.1 (-0.7, 0.4)	0.1 (-0.4, 0.6)	0.3 (-0.3, 0.8)	0.4 (-0.2, 1.1)	0.4 (0.0, 0.8)	0.1 (-0.3, 0.6)	0.3 (0.0, 0.7)	0.5 (0.1, 1.0)	0.7 (0.1, 1.3)
Greece										
rural	18.9 (17.9, 19.8)	15.4 (14.3, 16.4)	18.1 (17.1, 19.1)	20.8 (19.7, 21.8)	21.2 (20.1, 22.3)	19.7 (19.2, 20.3)	16.2 (15.6, 16.8)	18.9 (18.4, 19.5)	21.7 (21.1, 22.2)	22.0 (21.3, 22.7)
urban	18.9 (17.9, 19.9)	15.3 (14.2, 16.3)	18.1 (17.1, 19.0)	20.8 (19.8, 21.9)	21.3 (20.2, 22.3)	19.9 (19.4, 20.4)	16.3 (15.7, 16.9)	19.1 (18.6, 19.6)	21.9 (21.3, 22.4)	22.3 (21.7, 22.9)
urban-rural difference	0.0 (-0.3, 0.4)	-0.1 (-0.5, 0.3)	0.0 (-0.3, 0.3)	0.1 (-0.3, 0.4)	0.1 (-0.4, 0.6)	0.2 (-0.1, 0.5)	0.1 (-0.3, 0.4)	0.1 (-0.2, 0.4)	0.2 (-0.1, 0.5)	0.3 (-0.2, 0.7)
Israel										
rural	19.1 (18.5, 19.6)	15.0 (13.9, 16.0)	17.9 (17.3, 18.6)	21.4 (20.9, 21.8)	22.3 (21.8, 22.8)	19.6 (19.1, 20.2)	15.5 (14.5, 16.4)	18.5 (17.9, 19.1)	21.9 (21.3, 22.5)	22.8 (22.2, 23.4)
urban	18.9 (18.4, 19.4)	14.9 (14.0, 15.9)	17.8 (17.2, 18.4)	21.1 (20.7, 21.6)	22.0 (21.5, 22.4)	19.7 (19.3, 20.2)	15.8 (15.0, 16.6)	18.6 (18.1, 19.2)	22.0 (21.4, 22.5)	22.8 (22.2, 23.4)
urban-rural difference	-0.2 (-0.6, 0.2)	0.0 (-0.7, 0.7)	-0.1 (-0.6, 0.3)	-0.2 (-0.5, 0.1)	-0.3 (-0.6, 0.0)	0.1 (-0.2, 0.5)	0.3 (-0.3, 0.9)	0.2 (-0.2, 0.6)	0.1 (-0.2, 0.4)	0.0 (-0.4, 0.3)
Italy										
rural	19.5 (18.7, 20.3)	16.1 (15.1, 17.1)	18.3 (17.5, 19.2)	21.5 (20.7, 22.3)	22.1 (21.2, 23.1)	19.6 (19.0, 20.1)	16.2 (15.5, 16.9)	18.4 (17.9, 19.0)	21.6 (21.0, 22.2)	22.2 (21.4, 23.0)
urban	19.3 (18.5, 20.1)	16.1 (15.2, 17.1)	18.2 (17.4, 19.0)	21.3 (20.5, 22.1)	21.8 (20.9, 22.7)	19.5 (19.0, 20.0)	16.3 (15.7, 17.1)	18.4 (17.9, 19.0)	21.5 (20.9, 22.1)	22.0 (21.2, 22.7)
urban-rural difference	-0.1 (-0.6, 0.4)	0.0 (-0.5, 0.6)	-0.1 (-0.6, 0.4)	-0.2 (-0.8, 0.3)	-0.3 (-1.0, 0.3)	-0.1 (-0.4, 0.3)	0.1 (-0.2, 0.5)	0.0 (-0.3, 0.3)	-0.1 (-0.6, 0.3)	-0.2 (-0.8, 0.3)
Malta										
rural	19.4 (17.9, 20.8)	16.0 (14.5, 17.4)	18.1 (16.7, 19.6)	21.3 (19.8, 22.9)	22.7 (20.5, 24.8)	19.8 (19.0, 20.6)	16.4 (15.4, 17.3)	18.6 (17.8, 19.3)	21.8 (20.7, 22.8)	23.1 (21.3, 25.0)
urban	19.3 (17.9, 20.7)	15.8 (14.4, 17.2)	18.1 (16.6, 19.4)	21.3 (19.8, 22.9)	22.8 (20.6, 24.9)	19.9 (19.2, 20.6)	16.4 (15.6, 17.2)	18.7 (18.0, 19.3)	22.0 (21.0, 22.9)	23.4 (21.7, 25.1)
urban-rural difference	-0.1 (-0.7, 0.6)	-0.2 (-0.9, 0.5)	-0.1 (-0.7, 0.5)	0.0 (-0.7, 0.7)	0.1 (-0.8, 1.0)	0.2 (-0.4, 0.7)	0.0 (-0.6, 0.6)	0.1 (-0.4, 0.6)	0.2 (-0.4, 0.9)	0.3 (-0.5, 1.2)
Portugal										
rural	19.1 (17.8, 20.3)	15.9 (14.6, 17.2)	18.2 (17.0, 19.5)	21.0 (19.7, 22.3)	21.1 (19.8, 22.5)	19.2 (18.7, 19.8)	16.0 (15.5, 16.6)	18.4 (17.8, 18.9)	21.1 (20.6, 21.7)	21.3 (20.5, 22.0)
urban	19.1 (17.8, 20.3)	15.7 (14.4, 17.0)	18.2 (16.9, 19.4)	21.1 (19.9, 22.4)	21.3 (20.0, 22.7)	19.4 (18.9, 19.9)	16.0 (15.5, 16.5)	18.5 (18.0, 19.0)	21.4 (20.8, 22.0)	21.7 (20.9, 22.4)
urban-rural difference	0.0 (-0.4, 0.5)	-0.2 (-0.7, 0.4)	0.0 (-0.5, 0.4)	0.1 (-0.4, 0.6)	0.2 (-0.3, 0.8)	0.2 (-0.1, 0.5)	0.0 (-0.3, 0.3)	0.1 (-0.2, 0.4)	0.3 (-0.1, 0.6)	0.4 (-0.1, 0.9)
Spain										
rural	19.6 (18.6, 20.6)	16.6 (15.6, 17.7)	18.7 (17.7, 19.8)	21.3 (20.3, 22.4)	21.7 (20.7, 22.8)	19.6 (19.1, 20.0)	16.6 (16.1, 17.1)	18.7 (18.3, 19.1)	21.3 (20.8, 21.8)	21.7 (21.1, 22.4)
urban	19.4 (18.4, 20.5)	16.1 (15.1, 17.2)	18.5 (17.5, 19.5)	21.3 (20.3, 22.3)	21.9 (20.8, 22.9)	19.9 (19.5, 20.3)	16.6 (16.1, 17.1)	19.0 (18.6, 19.4)	21.8 (21.3, 22.2)	22.3 (21.7, 22.9)
urban-rural difference	-0.1 (-0.6, 0.3)	-0.4 (-1.0, 0.1)	-0.2 (-0.7, 0.2)	0.0 (-0.5, 0.5)	0.2 (-0.4, 0.7)	0.3 (0.0, 0.6)	0.0 (-0.3, 0.3)	0.2 (0.0, 0.5)	0.4 (0.1, 0.8)	0.6 (0.2, 1.1)
**Latin America and Caribbean**										
Andean Latin America										
Bolivia										
rural	19.4 (17.9, 20.9)	15.4 (13.4, 17.5)	17.8 (16.3, 19.4)	21.8 (20.4, 23.3)	23.3 (21.7, 24.8)	20.5 (19.5, 21.5)	16.5 (14.8, 18.1)	18.9 (17.8, 20.0)	22.9 (22.0, 23.8)	24.3 (23.4, 25.2)
urban	19.5 (18.0, 21.0)	16.0 (14.0, 18.1)	18.1 (16.6, 19.6)	21.7 (20.2, 23.2)	22.8 (21.3, 24.3)	20.6 (19.6, 21.6)	17.1 (15.4, 18.8)	19.2 (18.1, 20.2)	22.8 (21.9, 23.7)	23.9 (23.0, 24.8)
urban-rural difference	0.1 (-0.5, 0.7)	0.6 (-0.2, 1.4)	0.2 (-0.4, 0.9)	-0.1 (-0.7, 0.4)	-0.4 (-1.0, 0.2)	0.1 (-0.4, 0.7)	0.7 (-0.1, 1.5)	0.3 (-0.3, 0.9)	-0.1 (-0.6, 0.4)	-0.4 (-0.9, 0.1)
Ecuador										
rural	19.3 (17.8, 20.8)	15.3 (13.8, 16.9)	17.8 (16.3, 19.3)	21.6 (20.1, 23.1)	23.3 (21.8, 24.8)	19.9 (19.3, 20.5)	15.9 (15.3, 16.6)	18.4 (17.8, 19.0)	22.2 (21.6, 22.8)	23.9 (23.2, 24.6)
urban	19.6 (18.1, 21.1)	15.9 (14.4, 17.5)	18.2 (16.7, 19.7)	21.7 (20.2, 23.2)	23.2 (21.7, 24.7)	20.2 (19.7, 20.8)	16.6 (16.0, 17.3)	18.9 (18.3, 19.4)	22.4 (21.8, 22.9)	23.9 (23.2, 24.5)
urban-rural difference	0.3 (-0.3, 0.9)	0.6 (0.0, 1.3)	0.4 (-0.2, 1.0)	0.1 (-0.5, 0.7)	-0.1 (-0.7, 0.6)	0.3 (0.0, 0.7)	0.7 (0.3, 1.1)	0.4 (0.1, 0.8)	0.2 (-0.2, 0.6)	0.0 (-0.5, 0.4)
Peru										
rural	19.4 (18.1, 20.7)	15.7 (14.3, 17.1)	17.7 (16.3, 19.0)	21.8 (20.4, 23.2)	23.4 (22.1, 24.8)	20.1 (19.7, 20.4)	16.3 (15.9, 16.8)	18.3 (17.9, 18.7)	22.5 (22.1, 22.8)	24.1 (23.7, 24.4)
urban	19.8 (18.4, 21.1)	16.6 (15.3, 18.0)	18.2 (16.8, 19.6)	21.9 (20.5, 23.2)	23.2 (21.8, 24.5)	20.9 (20.5, 21.3)	17.8 (17.3, 18.3)	19.3 (18.9, 19.7)	23.0 (22.7, 23.4)	24.3 (23.9, 24.7)
urban-rural difference	0.4 (-0.1, 0.8)	1.0 (0.4, 1.5)	0.5 (0.0, 1.0)	0.1 (-0.4, 0.5)	-0.3 (-0.8, 0.2)	0.8 (0.6, 1.1)	1.4 (1.0, 1.8)	1.0 (0.7, 1.3)	0.6 (0.3, 0.8)	0.2 (0.0, 0.5)
Caribbean										
Antigua and Barbuda										
rural	19.3 (17.6, 21.1)	15.2 (13.0, 17.4)	17.6 (15.9, 19.4)	21.9 (20.2, 23.6)	23.4 (20.8, 26.0)	21.1 (19.5, 22.8)	17.0 (14.9, 19.1)	19.4 (17.7, 21.1)	23.7 (22.0, 25.3)	25.2 (22.7, 27.6)
urban	19.9 (18.1, 21.6)	15.9 (13.7, 18.1)	18.2 (16.4, 20.0)	22.3 (20.6, 24.1)	23.7 (21.2, 26.3)	21.4 (19.7, 23.0)	17.4 (15.2, 19.5)	19.7 (18.1, 21.3)	23.8 (22.2, 25.5)	25.2 (22.7, 27.7)
urban-rural difference	0.5 (-0.2, 1.2)	0.7 (-0.2, 1.5)	0.6 (-0.1, 1.2)	0.4 (-0.3, 1.2)	0.3 (-0.6, 1.2)	0.3 (-0.5, 0.9)	0.4 (-0.5, 1.3)	0.3 (-0.4, 1.0)	0.2 (-0.6, 0.9)	0.1 (-0.8, 1.0)
Bahamas										
rural	19.8 (18.1, 21.5)	15.7 (13.6, 17.9)	18.2 (16.5, 19.9)	22.3 (20.6, 24.0)	23.5 (21.1, 26.0)	21.8 (20.4, 23.2)	17.7 (15.7, 19.7)	20.2 (18.8, 21.6)	24.3 (22.9, 25.7)	25.5 (23.2, 27.8)
urban	20.4 (18.7, 22.0)	16.4 (14.3, 18.6)	18.8 (17.1, 20.4)	22.8 (21.1, 24.4)	24.0 (21.5, 26.4)	22.1 (20.8, 23.4)	18.2 (16.3, 20.2)	20.5 (19.2, 21.9)	24.6 (23.3, 25.8)	25.7 (23.5, 27.9)
urban-rural difference	0.6 (-0.1, 1.2)	0.7 (-0.2, 1.6)	0.6 (-0.1, 1.3)	0.5 (-0.3, 1.2)	0.4 (-0.5, 1.3)	0.3 (-0.4, 1.0)	0.5 (-0.4, 1.3)	0.4 (-0.3, 1.1)	0.3 (-0.5, 1.0)	0.2 (-0.7, 1.1)
Barbados										
rural	19.2 (17.5, 20.8)	15.1 (12.9, 17.2)	17.4 (15.8, 19.1)	21.7 (20.1, 23.3)	23.2 (20.8, 25.6)	20.9 (19.5, 22.2)	16.8 (14.8, 18.7)	19.2 (17.8, 20.6)	23.4 (22.1, 24.8)	25.0 (22.7, 27.1)
urban	19.7 (18.0, 21.3)	15.7 (13.6, 17.9)	18.0 (16.4, 19.7)	22.2 (20.5, 23.8)	23.6 (21.2, 26.0)	21.2 (19.8, 22.6)	17.2 (15.2, 19.2)	19.5 (18.1, 20.9)	23.6 (22.3, 25.0)	25.1 (22.8, 27.3)

Girls	Mean BMI in 1990 (kg/m^2) Age-standardised	5 years	10 years	15 years	19 years	Mean BMI in 2020 (kg/m^2) Age-standardised	5 years	10 years	15 years	19 years
urban-rural difference	0.5 (-0.2, 1.2)	0.7 (-0.2, 1.5)	0.6 (-0.1, 1.3)	0.4 (-0.3, 1.2)	0.4 (-0.6, 1.3)	0.3 (-0.4, 0.9)	0.4 (-0.5, 1.3)	0.3 (-0.4, 1.0)	0.2 (-0.5, 0.9)	0.1 (-0.8, 1.0)
Belize										
rural	19.2 (17.5, 20.9)	15.1 (13.0, 17.3)	17.6 (15.8, 19.2)	21.7 (20.0, 23.5)	23.1 (20.5, 25.8)	21.0 (19.5, 22.5)	16.9 (14.9, 19.0)	19.4 (17.8, 20.9)	23.5 (22.0, 25.0)	24.9 (22.5, 27.4)
urban	19.7 (18.0, 21.4)	15.8 (13.6, 18.0)	18.1 (16.4, 19.8)	22.2 (20.5, 23.9)	23.5 (20.9, 26.2)	21.3 (19.7, 22.8)	17.3 (15.3, 19.4)	19.7 (18.2, 21.2)	23.8 (22.2, 25.3)	25.1 (22.6, 27.5)
urban-rural difference	0.5 (-0.2, 1.2)	0.7 (-0.2, 1.5)	0.6 (-0.1, 1.3)	0.5 (-0.3, 1.2)	0.4 (-0.6, 1.3)	0.3 (-0.4, 0.9)	0.4 (-0.5, 1.3)	0.3 (-0.4, 1.0)	0.2 (-0.6, 0.9)	0.1 (-0.8, 1.0)
Bermuda										
rural	18.5 (16.5, 20.5)	14.5 (12.1, 16.9)	16.9 (14.9, 18.9)	21.0 (18.9, 23.1)	22.3 (19.4, 25.2)	20.2 (18.2, 22.2)	16.2 (13.8, 18.6)	18.6 (16.6, 20.5)	22.7 (20.7, 24.7)	24.0 (21.1, 26.8)
urban	19.1 (17.0, 21.0)	15.2 (12.8, 17.6)	17.5 (15.5, 19.4)	21.5 (19.4, 23.5)	22.7 (19.8, 25.6)	20.5 (18.5, 22.5)	16.6 (14.2, 19.0)	18.9 (16.9, 20.8)	22.9 (20.9, 24.9)	24.2 (21.2, 27.0)
urban-rural difference	0.5 (-0.2, 1.2)	0.7 (-0.2, 1.5)	0.6 (-0.1, 1.3)	0.5 (-0.3, 1.2)	0.4 (-0.5, 1.4)	0.3 (-0.4, 1.0)	0.4 (-0.5, 1.3)	0.3 (-0.4, 1.0)	0.2 (-0.5, 1.0)	0.1 (-0.8, 1.1)
Cuba										
rural	17.4 (16.0, 18.7)	13.4 (11.4, 15.4)	15.5 (14.0, 16.9)	19.9 (18.7, 21.2)	21.6 (20.3, 23.0)	18.7 (17.3, 20.1)	14.7 (12.7, 16.7)	16.8 (15.3, 18.3)	21.3 (19.9, 22.6)	23.0 (21.5, 24.4)
urban	17.8 (16.6, 19.0)	14.0 (12.1, 15.9)	15.9 (14.6, 17.2)	20.3 (19.2, 21.4)	21.9 (20.9, 23.1)	18.8 (17.4, 20.2)	15.0 (13.0, 17.0)	16.9 (15.5, 18.4)	21.3 (20.0, 22.6)	22.9 (21.6, 24.2)
urban-rural difference	0.4 (-0.2, 1.1)	0.6 (-0.3, 1.4)	0.5 (-0.2, 1.2)	0.4 (-0.3, 1.1)	0.3 (-0.5, 1.1)	0.1 (-0.6, 0.7)	0.2 (-0.6, 1.1)	0.1 (-0.5, 0.8)	0.0 (-0.6, 0.7)	0.0 (-0.8, 0.8)
Dominica										
rural	19.0 (17.4, 20.6)	14.9 (12.8, 17.0)	17.3 (15.7, 18.9)	21.5 (20.0, 23.1)	23.0 (21.0, 25.1)	20.7 (19.2, 22.2)	16.6 (14.6, 18.6)	19.0 (17.5, 20.6)	23.2 (21.8, 24.7)	24.7 (22.8, 26.7)
urban	19.5 (18.0, 21.1)	15.6 (13.5, 17.7)	17.9 (16.3, 19.5)	22.0 (20.5, 23.5)	23.4 (21.4, 25.4)	21.0 (19.6, 22.4)	17.1 (15.0, 19.0)	19.3 (17.9, 20.9)	23.5 (22.1, 24.9)	24.9 (23.0, 26.8)
urban-rural difference	0.5 (-0.1, 1.2)	0.7 (-0.2, 1.6)	0.6 (-0.1, 1.3)	0.5 (-0.3, 1.2)	0.4 (-0.5, 1.3)	0.3 (-0.4, 1.0)	0.4 (-0.5, 1.3)	0.3 (-0.4, 1.0)	0.2 (-0.5, 1.0)	0.1 (-0.8, 1.1)
Dominican Republic										
rural	18.0 (16.8, 19.2)	14.0 (12.1, 15.9)	16.3 (15.0, 17.7)	20.5 (19.4, 21.6)	21.8 (20.7, 23.0)	20.7 (19.6, 21.8)	16.7 (14.8, 18.5)	19.1 (17.8, 20.3)	23.2 (22.2, 24.3)	24.6 (23.6, 25.6)
urban	18.5 (17.3, 19.7)	14.7 (12.9, 16.7)	16.9 (15.6, 18.2)	20.9 (19.7, 22.0)	22.1 (20.9, 23.2)	20.9 (19.7, 22.0)	17.1 (15.2, 18.9)	19.3 (18.0, 20.6)	23.3 (22.2, 24.4)	24.5 (23.4, 25.6)
urban-rural difference	0.5 (0.0, 1.0)	0.7 (-0.1, 1.6)	0.6 (0.0, 1.2)	0.4 (-0.1, 0.9)	0.2 (-0.4, 0.9)	0.2 (-0.4, 0.7)	0.4 (-0.4, 1.2)	0.2 (-0.4, 0.8)	0.1 (-0.5, 0.6)	-0.1 (-0.7, 0.5)
Grenada										
rural	18.5 (16.5, 20.6)	14.5 (12.1, 16.9)	16.9 (15.0, 18.9)	21.0 (19.0, 23.1)	22.4 (19.5, 25.2)	20.2 (18.2, 22.2)	16.2 (13.8, 18.5)	18.6 (16.6, 20.5)	22.7 (20.7, 24.7)	24.0 (21.2, 26.9)
urban	19.1 (17.1, 21.1)	15.2 (12.8, 17.6)	17.5 (15.5, 19.4)	21.5 (19.5, 23.6)	22.7 (19.9, 25.6)	20.5 (18.5, 22.4)	16.6 (14.2, 18.9)	18.9 (16.9, 20.8)	22.9 (20.9, 24.9)	24.2 (21.4, 26.9)
urban-rural difference	0.5 (-0.1, 1.2)	0.7 (-0.2, 1.5)	0.6 (-0.1, 1.3)	0.5 (-0.3, 1.2)	0.4 (-0.5, 1.3)	0.3 (-0.4, 1.0)	0.4 (-0.5, 1.3)	0.3 (-0.4, 1.0)	0.2 (-0.5, 0.9)	0.1 (-0.8, 1.0)
Guyana										
rural	17.9 (16.4, 19.5)	14.0 (12.0, 16.0)	16.3 (14.7, 18.0)	20.4 (18.9, 21.9)	21.8 (20.2, 23.4)	19.8 (18.6, 21.1)	15.9 (14.0, 17.8)	18.2 (16.9, 19.6)	22.3 (21.2, 23.5)	23.7 (22.4, 24.9)
urban	18.5 (17.0, 20.1)	14.7 (12.6, 16.8)	16.9 (15.3, 18.6)	20.9 (19.4, 22.5)	22.3 (20.6, 23.9)	20.2 (18.9, 21.4)	16.4 (14.5, 18.3)	18.6 (17.3, 20.0)	22.6 (21.4, 23.8)	23.9 (22.7, 25.3)
urban-rural difference	0.6 (-0.1, 1.3)	0.7 (-0.2, 1.5)	0.6 (-0.1, 1.3)	0.5 (-0.1, 1.3)	0.5 (-0.3, 1.3)	0.4 (-0.2, 1.0)	0.5 (-0.4, 1.3)	0.4 (-0.3, 1.0)	0.3 (-0.3, 0.9)	0.3 (-0.5, 1.0)
Haiti										
rural	16.9 (15.5, 18.3)	13.1 (11.1, 15.1)	15.3 (13.8, 16.8)	19.3 (17.9, 20.6)	20.8 (19.4, 22.2)	18.0 (17.1, 18.9)	14.1 (12.4, 15.8)	16.4 (15.3, 17.4)	20.3 (19.6, 21.1)	21.9 (21.1, 22.7)
urban	17.7 (16.3, 19.2)	13.9 (11.9, 16.0)	16.1 (14.6, 17.6)	20.0 (18.7, 21.4)	21.5 (20.1, 23.0)	18.3 (17.4, 19.2)	14.6 (12.9, 16.3)	16.7 (15.7, 17.8)	20.7 (19.9, 21.4)	22.2 (21.3, 22.9)
urban-rural difference	0.8 (0.3, 1.4)	0.9 (0.1, 1.7)	0.8 (0.2, 1.5)	0.7 (0.3, 1.3)	0.7 (0.1, 1.3)	0.3 (-0.1, 0.8)	0.4 (-0.4, 1.2)	0.4 (-0.2, 0.9)	0.3 (-0.1, 0.7)	0.3 (-0.2, 0.7)
Jamaica										
rural	18.3 (16.8, 19.7)	14.0 (12.1, 15.9)	16.7 (15.2, 18.2)	20.9 (19.5, 22.3)	22.0 (20.5, 23.6)	19.7 (18.6, 20.8)	15.5 (13.7, 17.1)	18.1 (16.9, 19.3)	22.3 (21.3, 23.4)	23.5 (22.3, 24.7)
urban	19.0 (17.6, 20.4)	14.9 (13.0, 16.8)	17.4 (16.0, 18.9)	21.6 (20.2, 23.0)	22.7 (21.1, 24.2)	20.1 (19.0, 21.2)	15.9 (14.2, 17.6)	18.5 (17.3, 19.6)	22.7 (21.6, 23.8)	23.8 (22.5, 25.0)
urban-rural difference	0.7 (0.2, 1.4)	0.8 (0.0, 1.7)	0.8 (0.1, 1.4)	0.7 (0.1, 1.3)	0.7 (-0.1, 1.4)	0.4 (-0.2, 1.0)	0.5 (-0.4, 1.3)	0.4 (-0.2, 1.1)	0.4 (-0.3, 0.9)	0.3 (-0.4, 1.0)
Puerto Rico										
rural	18.6 (16.9, 20.3)	14.6 (12.3, 16.7)	17.0 (15.3, 18.8)	21.1 (19.4, 22.9)	22.1 (19.8, 24.3)	20.2 (18.7, 21.8)	16.2 (14.2, 18.3)	18.7 (17.1, 20.3)	22.8 (21.3, 24.3)	23.7 (21.7, 25.8)
urban	19.1 (17.4, 20.8)	15.2 (13.0, 17.4)	17.6 (15.9, 19.3)	21.5 (19.9, 23.2)	22.4 (20.2, 24.6)	20.5 (19.0, 22.0)	16.7 (14.6, 18.7)	19.0 (17.4, 20.6)	23.0 (21.5, 24.4)	23.9 (21.9, 25.8)
urban-rural difference	0.5 (-0.2, 1.2)	0.7 (-0.2, 1.5)	0.6 (-0.1, 1.3)	0.5 (-0.3, 1.2)	0.4 (-0.5, 1.3)	0.3 (-0.4, 1.0)	0.4 (-0.5, 1.2)	0.3 (-0.4, 1.0)	0.2 (-0.5, 0.9)	0.1 (-0.7, 1.0)
Saint Kitts and Nevis										
rural	19.2 (17.6, 20.8)	15.1 (13.0, 17.3)	17.6 (15.9, 19.2)	21.7 (20.1, 23.3)	23.0 (20.7, 25.3)	21.0 (19.6, 22.3)	16.9 (15.0, 18.9)	19.3 (18.0, 20.7)	23.5 (22.2, 24.8)	24.8 (22.6, 27.0)
urban	19.7 (18.1, 21.3)	15.8 (13.7, 17.9)	18.1 (16.5, 19.8)	22.2 (20.6, 23.7)	23.4 (21.0, 25.8)	21.2 (19.8, 22.6)	17.3 (15.4, 19.4)	19.6 (18.2, 21.1)	23.7 (22.3, 25.0)	24.9 (22.7, 27.2)
urban-rural difference	0.5 (-0.1, 1.2)	0.7 (-0.2, 1.5)	0.6 (-0.1, 1.2)	0.4 (-0.3, 1.2)	0.4 (-0.6, 1.3)	0.3 (-0.4, 0.9)	0.4 (-0.5, 1.3)	0.3 (-0.4, 1.0)	0.2 (-0.6, 0.9)	0.1 (-0.8, 1.0)
Saint Lucia										
rural	18.6 (16.5, 20.5)	14.6 (12.1, 16.9)	16.9 (14.9, 18.9)	21.1 (19.0, 23.1)	22.4 (19.5, 25.3)	20.2 (18.2, 22.2)	16.2 (13.8, 18.5)	18.6 (16.6, 20.5)	22.7 (20.7, 24.7)	24.0 (21.2, 26.9)
urban	19.1 (17.1, 21.1)	15.2 (12.8, 17.6)	17.5 (15.5, 19.4)	21.5 (19.5, 23.6)	22.7 (19.9, 25.7)	20.5 (18.5, 22.4)	16.6 (14.2, 18.9)	18.9 (17.0, 20.8)	22.9 (20.9, 24.9)	24.1 (21.3, 27.0)
urban-rural difference	0.5 (-0.1, 1.2)	0.7 (-0.2, 1.5)	0.6 (-0.1, 1.3)	0.5 (-0.3, 1.2)	0.4 (-0.5, 1.3)	0.3 (-0.4, 0.9)	0.4 (-0.5, 1.3)	0.3 (-0.4, 1.0)	0.2 (-0.5, 0.9)	0.1 (-0.8, 1.0)
Saint Vincent and the Grenadines										
rural	19.0 (17.5, 20.6)	15.0 (12.8, 17.1)	17.3 (15.7, 18.9)	21.6 (20.0, 23.1)	23.2 (21.0, 25.3)	20.8 (19.9, 21.8)	16.8 (15.0, 18.5)	19.1 (18.0, 20.2)	23.4 (22.5, 24.2)	25.0 (23.3, 26.6)

Girls	Mean BMI in 1990 (kg/m^2)					Mean BMI in 2020 (kg/m^2)				
	Age-standardised	5 years	10 years	15 years	19 years	Age-standardised	5 years	10 years	15 years	19 years
urban	19.5 (17.9, 21.1)	15.6 (13.5, 17.7)	17.9 (16.2, 19.5)	22.0 (20.4, 23.5)	23.5 (21.4, 25.6)	21.1 (20.1, 22.1)	17.2 (15.5, 18.9)	19.4 (18.3, 20.5)	23.6 (22.7, 24.4)	25.1 (23.5, 26.7)
urban-rural difference	0.5 (-0.2, 1.2)	0.7 (-0.2, 1.5)	0.6 (-0.1, 1.3)	0.5 (-0.3, 1.2)	0.4 (-0.5, 1.3)	0.3 (-0.4, 1.0)	0.4 (-0.5, 1.3)	0.3 (-0.4, 1.0)	0.2 (-0.5, 0.9)	0.1 (-0.8, 1.0)
Suriname										
rural	18.2 (16.3, 20.1)	14.2 (11.9, 16.5)	16.7 (14.8, 18.6)	20.7 (18.8, 22.5)	21.4 (19.1, 23.8)	19.8 (18.1, 21.5)	15.8 (13.7, 18.0)	18.3 (16.6, 20.1)	22.3 (20.6, 24.0)	23.0 (20.9, 25.2)
urban	18.7 (16.8, 20.5)	14.9 (12.6, 17.2)	17.2 (15.3, 19.1)	21.1 (19.3, 23.0)	21.8 (19.5, 24.1)	20.0 (18.3, 21.7)	16.2 (14.1, 18.4)	18.6 (16.8, 20.3)	22.5 (20.7, 24.1)	23.1 (21.0, 25.3)
urban-rural difference	0.5 (-0.2, 1.2)	0.7 (-0.2, 1.6)	0.6 (-0.1, 1.3)	0.4 (-0.3, 1.2)	0.4 (-0.6, 1.3)	0.3 (-0.4, 0.9)	0.4 (-0.5, 1.3)	0.3 (-0.4, 1.0)	0.2 (-0.6, 0.9)	0.1 (-0.8, 1.0)
Trinidad and Tobago										
rural	17.8 (16.4, 19.0)	14.0 (12.7, 15.2)	16.3 (15.0, 17.6)	20.2 (18.8, 21.6)	21.1 (19.2, 23.1)	19.6 (18.3, 21.0)	15.8 (14.4, 17.2)	18.1 (16.8, 19.5)	22.0 (20.7, 23.4)	23.0 (21.2, 24.8)
urban	18.3 (17.0, 19.6)	14.5 (13.2, 15.7)	16.8 (15.5, 18.1)	20.7 (19.4, 22.1)	21.6 (19.7, 23.6)	19.9 (18.6, 21.3)	16.1 (14.7, 17.6)	18.4 (17.1, 19.8)	22.3 (21.0, 23.7)	23.3 (21.4, 25.1)
urban-rural difference	0.5 (0.0, 1.1)	0.5 (0.0, 1.0)	0.5 (0.0, 1.0)	0.5 (-0.1, 1.1)	0.5 (-0.3, 1.3)	0.3 (-0.3, 0.9)	0.3 (-0.4, 1.0)	0.3 (-0.3, 0.9)	0.3 (-0.4, 1.0)	0.3 (-0.5, 1.1)
Central Latin America										
Colombia										
rural	17.8 (16.4, 19.2)	14.2 (12.8, 15.6)	16.1 (14.7, 17.5)	20.2 (18.9, 21.6)	21.7 (20.3, 23.1)	19.7 (18.9, 20.5)	16.1 (15.3, 16.9)	18.0 (17.2, 18.8)	22.1 (21.3, 22.9)	23.6 (22.7, 24.4)
urban	18.1 (16.8, 19.5)	14.9 (13.5, 16.3)	16.5 (15.1, 17.9)	20.4 (19.0, 21.7)	21.6 (20.2, 23.0)	19.8 (19.0, 20.5)	16.6 (15.8, 17.4)	18.2 (17.4, 18.9)	22.0 (21.3, 22.8)	23.3 (22.4, 24.0)
urban-rural difference	0.3 (-0.1, 0.8)	0.7 (0.2, 1.2)	0.4 (0.0, 0.9)	0.1 (-0.3, 0.7)	-0.1 (-0.6, 0.5)	0.1 (-0.3, 0.4)	0.5 (0.1, 0.9)	0.2 (-0.2, 0.6)	-0.1 (-0.4, 0.3)	-0.3 (-0.7, 0.1)
Costa Rica										
rural	18.3 (16.8, 19.9)	14.5 (12.6, 16.5)	16.9 (15.3, 18.5)	20.8 (19.2, 22.4)	21.7 (19.6, 23.7)	20.0 (18.7, 21.2)	16.2 (14.4, 17.9)	18.5 (17.1, 19.8)	22.4 (21.1, 23.6)	23.3 (21.5, 25.0)
urban	18.8 (17.2, 20.3)	15.2 (13.2, 17.2)	17.4 (15.8, 19.0)	21.1 (19.6, 22.7)	21.9 (19.9, 23.9)	20.3 (19.0, 21.4)	16.7 (14.9, 18.4)	18.9 (17.5, 20.1)	22.6 (21.4, 23.7)	23.4 (21.8, 25.0)
urban-rural difference	0.4 (-0.2, 1.1)	0.6 (-0.2, 1.4)	0.5 (-0.2, 1.1)	0.3 (-0.4, 1.1)	0.2 (-0.6, 1.1)	0.3 (-0.3, 0.9)	0.5 (-0.3, 1.3)	0.4 (-0.3, 1.0)	0.2 (-0.4, 0.9)	0.1 (-0.7, 1.0)
El Salvador										
rural	18.5 (17.0, 19.9)	14.7 (12.7, 16.7)	16.8 (15.3, 18.4)	20.9 (19.4, 22.3)	22.5 (21.0, 24.0)	20.1 (18.9, 21.3)	16.3 (14.5, 18.0)	18.4 (17.3, 19.7)	22.5 (21.4, 23.6)	24.1 (22.8, 25.5)
urban	19.0 (17.6, 20.5)	15.3 (13.3, 17.3)	17.4 (15.9, 18.9)	21.4 (19.9, 22.8)	23.0 (21.4, 24.5)	20.5 (19.4, 21.7)	16.8 (15.0, 18.5)	18.9 (17.7, 20.1)	22.9 (21.8, 24.0)	24.5 (23.2, 25.8)
urban-rural difference	0.5 (0.0, 1.1)	0.6 (-0.2, 1.4)	0.6 (0.0, 1.2)	0.5 (-0.1, 1.1)	0.5 (-0.2, 1.2)	0.4 (-0.1, 1.0)	0.5 (-0.3, 1.3)	0.4 (-0.1, 1.0)	0.4 (-0.1, 0.9)	0.4 (-0.3, 1.0)
Guatemala										
rural	18.4 (17.0, 19.8)	14.4 (12.9, 15.9)	16.7 (15.3, 18.2)	20.9 (19.5, 22.3)	22.3 (20.9, 23.7)	19.6 (19.2, 20.1)	15.6 (15.0, 16.3)	18.0 (17.5, 18.5)	22.1 (21.7, 22.5)	23.5 (23.0, 24.0)
urban	18.9 (17.5, 20.3)	15.0 (13.5, 16.5)	17.3 (15.9, 18.7)	21.3 (20.0, 22.8)	22.7 (21.3, 24.1)	19.9 (19.5, 20.4)	16.1 (15.4, 16.7)	18.3 (17.8, 18.8)	22.4 (21.9, 22.8)	23.7 (23.2, 24.3)
urban-rural difference	0.5 (0.0, 1.1)	0.6 (-0.1, 1.4)	0.5 (0.0, 1.1)	0.5 (-0.1, 1.0)	0.4 (-0.2, 1.0)	0.3 (-0.1, 0.7)	0.4 (-0.3, 1.1)	0.4 (-0.1, 0.8)	0.3 (-0.1, 0.6)	0.2 (-0.2, 0.6)
Honduras										
rural	18.1 (16.6, 19.6)	14.3 (12.3, 16.3)	16.5 (15.0, 18.1)	20.5 (19.0, 22.0)	22.0 (20.4, 23.5)	19.6 (18.4, 20.8)	15.8 (14.1, 17.6)	18.0 (16.8, 19.3)	22.0 (20.9, 23.2)	23.5 (22.3, 24.7)
urban	18.6 (17.2, 20.1)	14.9 (13.0, 16.9)	17.1 (15.5, 18.6)	21.0 (19.6, 22.5)	22.4 (20.9, 23.9)	20.0 (18.8, 21.2)	16.3 (14.5, 18.1)	18.5 (17.2, 19.7)	22.4 (21.3, 23.5)	23.8 (22.6, 25.0)
urban-rural difference	0.5 (-0.1, 1.1)	0.6 (-0.2, 1.4)	0.5 (-0.1, 1.1)	0.5 (-0.1, 1.0)	0.4 (-0.2, 1.0)	0.4 (-0.1, 0.9)	0.5 (-0.3, 1.3)	0.4 (-0.1, 1.0)	0.4 (-0.1, 0.8)	0.3 (-0.2, 0.8)
Mexico										
rural	17.5 (16.9, 18.0)	13.2 (12.7, 13.8)	16.0 (15.4, 16.5)	20.1 (19.5, 20.6)	21.3 (20.7, 21.9)	20.2 (19.7, 20.7)	16.0 (15.5, 16.5)	18.7 (18.2, 19.2)	22.8 (22.3, 23.3)	24.0 (23.5, 24.6)
urban	18.1 (17.5, 18.6)	13.9 (13.3, 14.5)	16.6 (16.0, 17.1)	20.6 (20.1, 21.1)	21.8 (21.2, 22.3)	20.8 (20.2, 21.3)	16.6 (16.1, 17.1)	19.3 (18.7, 19.8)	23.3 (22.8, 23.8)	24.5 (23.9, 25.0)
urban-rural difference	0.6 (0.3, 0.9)	0.7 (0.3, 1.0)	0.6 (0.3, 0.9)	0.5 (0.3, 0.8)	0.5 (0.2, 0.8)	0.5 (0.3, 0.8)	0.6 (0.3, 0.9)	0.5 (0.3, 0.8)	0.5 (0.2, 0.8)	0.5 (0.1, 0.8)
Nicaragua										
rural	18.7 (17.4, 19.9)	14.8 (13.0, 16.5)	17.1 (15.8, 18.4)	21.1 (19.9, 22.3)	22.3 (21.1, 23.5)	20.1 (18.9, 21.3)	16.2 (14.5, 18.0)	18.6 (17.3, 19.8)	22.6 (21.5, 23.7)	23.8 (22.6, 24.9)
urban	19.0 (17.8, 20.3)	15.3 (13.6, 17.1)	17.5 (16.2, 18.9)	21.3 (20.1, 22.6)	22.4 (21.2, 23.6)	20.4 (19.2, 21.6)	16.8 (15.0, 18.6)	19.0 (17.7, 20.2)	22.8 (21.7, 23.9)	23.8 (22.7, 25.0)
urban-rural difference	0.3 (-0.2, 0.8)	0.6 (-0.2, 1.3)	0.4 (-0.2, 0.9)	0.2 (-0.2, 0.7)	0.1 (-0.4, 0.6)	0.3 (-0.2, 0.8)	0.5 (-0.2, 1.3)	0.4 (-0.2, 0.9)	0.2 (-0.2, 0.7)	0.1 (-0.4, 0.6)
Panama										
rural	18.8 (17.4, 20.2)	15.0 (13.5, 16.4)	17.1 (15.7, 18.5)	21.3 (19.9, 22.7)	22.9 (21.5, 24.4)	21.0 (20.4, 21.6)	17.1 (16.4, 17.9)	19.3 (18.6, 19.9)	23.4 (22.8, 24.1)	25.1 (24.3, 25.9)
urban	19.1 (17.8, 20.5)	15.5 (14.0, 16.9)	17.5 (16.1, 18.9)	21.5 (20.1, 22.9)	23.1 (21.6, 24.6)	21.1 (20.5, 21.7)	17.4 (16.6, 18.2)	19.4 (18.8, 20.1)	23.5 (22.9, 24.1)	25.0 (24.2, 25.8)
urban-rural difference	0.3 (-0.2, 0.8)	0.5 (-0.1, 1.1)	0.4 (-0.1, 0.9)	0.2 (-0.3, 0.8)	0.1 (-0.5, 0.8)	0.1 (-0.4, 0.6)	0.3 (-0.3, 0.9)	0.2 (-0.4, 0.6)	0.0 (-0.5, 0.5)	-0.1 (-0.6, 0.5)
Venezuela										
rural	17.8 (16.2, 19.4)	14.0 (12.1, 16.0)	16.1 (14.5, 17.7)	20.2 (18.6, 21.8)	22.2 (20.3, 24.2)	19.3 (18.0, 20.5)	15.5 (13.7, 17.1)	17.5 (16.2, 18.8)	21.6 (20.4, 22.9)	23.7 (22.0, 25.3)
urban	18.2 (16.7, 19.8)	14.6 (12.7, 16.5)	16.5 (14.9, 18.1)	20.5 (18.9, 22.0)	22.4 (20.5, 24.3)	19.5 (18.4, 20.6)	15.9 (14.3, 17.5)	17.8 (16.7, 19.0)	21.8 (20.7, 22.9)	23.7 (22.3, 25.2)
urban-rural difference	0.4 (-0.3, 1.1)	0.6 (-0.2, 1.4)	0.5 (-0.2, 1.1)	0.3 (-0.4, 1.0)	0.2 (-0.7, 1.1)	0.3 (-0.4, 0.9)	0.4 (-0.3, 1.2)	0.3 (-0.3, 0.9)	0.2 (-0.5, 0.9)	0.1 (-0.8, 0.9)
Southern Latin America										
Argentina										
rural	18.7 (17.3, 20.2)	15.1 (13.6, 16.6)	17.6 (16.2, 19.0)	20.9 (19.5, 22.4)	21.4 (19.8, 23.0)	20.1 (19.3, 20.9)	16.5 (15.6, 17.5)	19.0 (18.2, 19.8)	22.3 (21.5, 23.1)	22.8 (21.8, 23.8)
urban	19.1 (17.7, 20.4)	15.4 (14.0, 16.8)	17.9 (16.5, 19.3)	21.3 (19.9, 22.7)	21.9 (20.5, 23.3)	20.4 (19.8, 21.0)	16.7 (16.0, 17.5)	19.3 (18.7, 19.9)	22.7 (22.1, 23.3)	23.2 (22.5, 23.9)

Girls	Mean BMI in 1990 (kg/m^2)					Mean BMI in 2020 (kg/m^2)				
	Age-standardised	5 years	10 years	15 years	19 years	Age-standardised	5 years	10 years	15 years	19 years
urban-rural difference	0.4 (-0.3, 1.0)	0.3 (-0.5, 1.0)	0.3 (-0.3, 1.0)	0.4 (-0.3, 1.1)	0.5 (-0.3, 1.3)	0.3 (-0.3, 0.9)	0.2 (-0.5, 1.0)	0.3 (-0.3, 0.9)	0.4 (-0.3, 1.0)	0.4 (-0.3, 1.2)
Brazil										
rural	18.4 (17.9, 18.9)	15.1 (14.6, 15.6)	17.0 (16.5, 17.5)	20.5 (20.0, 21.0)	22.0 (21.5, 22.5)	20.0 (19.7, 20.4)	16.7 (16.3, 17.1)	18.6 (18.3, 19.0)	22.1 (21.8, 22.5)	23.6 (23.2, 24.1)
urban	18.7 (18.2, 19.1)	15.8 (15.3, 16.3)	17.4 (16.9, 17.9)	20.6 (20.1, 21.1)	21.8 (21.3, 22.4)	20.2 (19.9, 20.5)	17.3 (16.9, 17.7)	18.9 (18.6, 19.2)	22.1 (21.8, 22.4)	23.4 (23.0, 23.7)
urban-rural difference	0.3 (0.0, 0.5)	0.7 (0.4, 1.0)	0.4 (0.1, 0.7)	0.1 (-0.2, 0.4)	-0.2 (-0.5, 0.2)	0.1 (-0.1, 0.4)	0.6 (0.3, 0.8)	0.3 (0.0, 0.5)	0.0 (-0.3, 0.2)	-0.3 (-0.5, 0.0)
Chile										
rural	19.9 (18.6, 21.2)	15.5 (14.0, 16.9)	18.5 (17.2, 19.9)	22.3 (21.0, 23.6)	24.4 (22.9, 25.8)	21.2 (20.3, 22.1)	16.8 (15.7, 17.9)	19.8 (18.8, 20.8)	23.6 (22.7, 24.5)	25.7 (24.6, 26.8)
urban	20.2 (19.0, 21.5)	16.3 (14.8, 17.7)	18.9 (17.6, 20.3)	22.4 (21.1, 23.6)	24.2 (22.9, 25.5)	21.6 (20.8, 22.5)	17.7 (16.6, 18.7)	20.4 (19.4, 21.2)	23.8 (22.9, 24.6)	25.6 (24.6, 26.6)
urban-rural difference	0.3 (-0.4, 0.9)	0.8 (0.0, 1.5)	0.4 (-0.2, 1.0)	0.1 (-0.6, 0.7)	-0.2 (-1.0, 0.6)	0.4 (-0.1, 0.9)	0.9 (0.2, 1.6)	0.5 (0.0, 1.1)	0.2 (-0.3, 0.7)	-0.1 (-0.8, 0.6)
Paraguay										
rural	18.7 (17.2, 20.2)	15.2 (13.3, 17.2)	17.5 (15.9, 19.0)	20.9 (19.4, 22.3)	21.7 (19.9, 23.6)	20.0 (19.1, 20.9)	16.5 (15.0, 18.1)	18.8 (17.8, 19.7)	22.2 (21.4, 23.0)	23.0 (21.7, 24.4)
urban	19.0 (17.5, 20.5)	15.8 (13.9, 17.8)	17.8 (16.3, 19.4)	21.0 (19.5, 22.5)	21.7 (20.0, 23.4)	20.2 (19.4, 21.1)	17.1 (15.6, 18.6)	19.1 (18.2, 20.0)	22.2 (21.4, 23.0)	22.9 (21.6, 24.2)
urban-rural difference	0.3 (-0.4, 0.9)	0.6 (-0.2, 1.4)	0.4 (-0.3, 1.0)	0.1 (-0.6, 0.8)	-0.1 (-0.9, 0.7)	0.2 (-0.3, 0.7)	0.6 (-0.2, 1.3)	0.3 (-0.2, 0.8)	0.1 (-0.5, 0.6)	-0.1 (-0.8, 0.5)
Uruguay										
rural	19.0 (17.6, 20.4)	15.8 (14.2, 17.3)	17.5 (16.1, 19.0)	21.1 (19.6, 22.6)	22.5 (20.7, 24.4)	20.6 (19.7, 21.4)	17.3 (16.1, 18.5)	19.1 (18.1, 20.0)	22.7 (21.8, 23.6)	24.1 (22.7, 25.5)
urban	19.3 (17.9, 20.7)	16.4 (15.0, 17.9)	17.9 (16.6, 19.4)	21.3 (19.9, 22.8)	22.5 (20.8, 24.3)	20.9 (20.2, 21.5)	17.9 (17.0, 18.9)	19.5 (18.7, 20.2)	22.9 (22.3, 23.5)	24.1 (22.9, 25.2)
urban-rural difference	0.3 (-0.3, 1.0)	0.6 (-0.2, 1.4)	0.4 (-0.2, 1.1)	0.2 (-0.5, 0.9)	0.0 (-0.9, 0.9)	0.3 (-0.3, 1.0)	0.6 (-0.2, 1.4)	0.4 (-0.3, 1.1)	0.2 (-0.5, 0.9)	0.0 (-0.8, 0.9)
**Oceania**										
Melanesia										
Fiji										
rural	18.8 (17.2, 20.3)	14.8 (12.4, 17.1)	16.4 (14.8, 18.1)	21.3 (19.8, 22.8)	24.5 (22.9, 26.2)	19.9 (18.9, 21.0)	16.0 (13.9, 18.0)	17.6 (16.5, 18.8)	22.5 (21.5, 23.5)	25.7 (24.5, 26.9)
urban	18.7 (17.1, 20.3)	15.2 (12.8, 17.5)	16.5 (14.9, 18.2)	21.1 (19.6, 22.7)	24.1 (22.4, 25.7)	20.0 (19.0, 21.1)	16.4 (14.4, 18.4)	17.8 (16.7, 18.9)	22.4 (21.5, 23.4)	25.4 (24.2, 26.5)
urban-rural difference	0.0 (-0.8, 0.7)	0.4 (-0.6, 1.4)	0.1 (-0.7, 0.9)	-0.2 (-1.0, 0.5)	-0.4 (-1.3, 0.4)	0.1 (-0.7, 0.8)	0.5 (-0.5, 1.5)	0.2 (-0.6, 1.0)	-0.1 (-0.9, 0.7)	-0.4 (-1.3, 0.6)
Papua New Guinea										
rural	19.8 (18.0, 21.6)	15.7 (13.3, 18.1)	17.6 (15.7, 19.6)	22.7 (20.9, 24.4)	24.4 (22.5, 26.3)	21.0 (19.4, 22.6)	16.9 (14.6, 19.2)	18.8 (17.1, 20.6)	23.9 (22.3, 25.4)	25.6 (23.9, 27.3)
urban	19.9 (18.1, 21.9)	16.1 (13.6, 18.6)	17.8 (15.9, 19.8)	22.7 (20.9, 24.6)	24.2 (22.2, 26.4)	21.2 (19.5, 22.9)	17.4 (15.0, 19.7)	19.1 (17.3, 20.9)	23.9 (22.3, 25.6)	25.5 (23.7, 27.4)
urban-rural difference	0.1 (-0.7, 1.0)	0.4 (-0.6, 1.5)	0.2 (-0.6, 1.1)	0.0 (-0.9, 0.9)	-0.1 (-1.3, 1.0)	0.2 (-0.6, 1.0)	0.5 (-0.5, 1.5)	0.3 (-0.5, 1.1)	0.1 (-0.8, 1.0)	-0.1 (-1.2, 1.0)
Solomon Islands										
rural	19.3 (17.6, 20.9)	14.8 (13.0, 16.6)	16.8 (15.1, 18.5)	22.5 (20.8, 24.2)	24.2 (22.4, 25.9)	20.3 (19.0, 21.7)	15.9 (14.5, 17.3)	17.8 (16.5, 19.2)	23.6 (22.3, 24.9)	25.2 (23.9, 26.6)
urban	19.5 (17.8, 21.2)	15.3 (13.6, 17.1)	17.1 (15.4, 18.8)	22.5 (20.8, 24.3)	24.0 (22.2, 25.8)	20.6 (19.3, 21.9)	16.5 (15.0, 17.9)	18.2 (16.8, 19.6)	23.7 (22.2, 25.1)	25.1 (23.6, 26.7)
urban-rural difference	0.2 (-0.6, 1.0)	0.5 (-0.4, 1.4)	0.3 (-0.5, 1.1)	0.0 (-0.9, 0.9)	-0.2 (-1.2, 0.8)	0.3 (-0.4, 1.0)	0.6 (-0.2, 1.5)	0.4 (-0.3, 1.1)	0.1 (-0.7, 0.9)	-0.1 (-1.1, 0.9)
Vanuatu										
rural	19.5 (17.6, 21.3)	15.5 (12.9, 18.0)	17.5 (15.5, 19.4)	22.3 (20.5, 24.1)	23.7 (21.6, 25.8)	20.7 (18.9, 22.4)	16.7 (14.3, 19.1)	18.6 (16.8, 20.5)	23.5 (21.8, 25.1)	24.9 (22.9, 26.9)
urban	19.7 (17.8, 21.6)	15.9 (13.3, 18.4)	17.7 (15.7, 19.7)	22.3 (20.5, 24.2)	23.6 (21.4, 25.8)	20.9 (19.1, 22.7)	17.1 (14.8, 19.6)	18.9 (17.1, 20.8)	23.6 (21.8, 25.3)	24.8 (22.7, 26.9)
urban-rural difference	0.2 (-0.7, 1.0)	0.4 (-0.6, 1.5)	0.2 (-0.6, 1.1)	0.1 (-0.9, 0.9)	-0.1 (-1.2, 1.0)	0.2 (-0.6, 1.0)	0.5 (-0.5, 1.5)	0.3 (-0.5, 1.1)	0.1 (-0.8, 1.0)	0.0 (-1.1, 1.0)
Polynesia and Micronesia										
American Samoa										
rural	21.0 (18.7, 23.3)	17.2 (14.2, 20.2)	18.9 (16.7, 21.1)	23.5 (21.3, 25.8)	25.9 (22.9, 28.8)	22.5 (20.4, 24.5)	18.7 (16.0, 21.5)	20.4 (18.3, 22.4)	25.0 (22.9, 27.1)	27.3 (24.5, 30.2)
urban	21.2 (19.0, 23.5)	17.6 (14.6, 20.6)	19.2 (16.9, 21.4)	23.7 (21.4, 26.0)	26.0 (23.0, 29.0)	22.7 (20.6, 24.7)	19.1 (16.3, 21.9)	20.7 (18.6, 22.6)	25.2 (23.1, 27.3)	27.5 (24.6, 30.3)
urban-rural difference	0.2 (-0.6, 1.1)	0.4 (-0.7, 1.5)	0.3 (-0.6, 1.1)	0.2 (-0.7, 1.1)	0.1 (-1.0, 1.2)	0.2 (-0.6, 1.1)	0.4 (-0.7, 1.4)	0.3 (-0.6, 1.1)	0.2 (-0.7, 1.1)	0.1 (-1.0, 1.3)
Cook Islands										
rural	22.0 (20.2, 23.8)	18.1 (15.3, 20.9)	19.7 (17.9, 21.5)	24.6 (22.8, 26.3)	27.3 (25.0, 29.5)	23.7 (22.5, 25.0)	19.9 (17.6, 22.3)	21.5 (20.1, 22.9)	26.4 (25.2, 27.6)	29.1 (27.4, 30.9)
urban	22.2 (20.3, 23.9)	18.4 (15.6, 21.2)	20.0 (18.1, 21.8)	24.8 (23.0, 26.5)	27.5 (25.2, 29.5)	24.0 (22.9, 25.2)	20.2 (17.9, 22.6)	21.8 (20.5, 23.1)	26.6 (25.6, 27.7)	29.3 (27.7, 30.8)
urban-rural difference	0.3 (-0.6, 1.1)	0.3 (-0.8, 1.4)	0.3 (-0.6, 1.1)	0.2 (-0.7, 1.1)	0.2 (-1.0, 1.3)	0.3 (-0.5, 1.1)	0.4 (-0.7, 1.4)	0.3 (-0.5, 1.1)	0.2 (-0.6, 1.1)	0.2 (-0.9, 1.3)
French Polynesia										
rural	21.0 (19.1, 22.7)	17.4 (14.7, 20.1)	19.1 (17.2, 20.9)	23.4 (21.6, 25.1)	25.2 (23.1, 27.3)	22.4 (21.2, 23.6)	18.8 (16.5, 21.2)	20.5 (19.2, 21.8)	24.8 (23.7, 26.0)	26.7 (25.0, 28.3)
urban	21.2 (19.4, 22.9)	17.8 (15.0, 20.5)	19.4 (17.5, 21.2)	23.6 (21.8, 25.2)	25.3 (23.3, 27.4)	22.6 (21.5, 23.8)	19.2 (16.9, 21.6)	20.8 (19.6, 22.0)	25.0 (24.0, 26.1)	26.8 (25.2, 28.4)
urban-rural difference	0.2 (-0.6, 1.1)	0.4 (-0.7, 1.4)	0.3 (-0.6, 1.1)	0.2 (-0.7, 1.1)	0.1 (-1.0, 1.2)	0.3 (-0.6, 1.1)	0.4 (-0.7, 1.4)	0.3 (-0.6, 1.1)	0.2 (-0.7, 1.1)	0.1 (-1.0, 1.2)
Kiribati										
rural	20.6 (18.7, 22.4)	16.9 (14.1, 19.6)	18.6 (16.8, 20.5)	23.1 (21.4, 24.9)	24.8 (22.9, 26.6)	21.7 (20.3, 23.1)	18.0 (15.5, 20.5)	19.8 (18.3, 21.3)	24.2 (22.9, 25.6)	26.0 (24.5, 27.4)
urban	20.8 (19.0, 22.6)	17.2 (14.4, 20.1)	18.9 (17.1, 20.8)	23.3 (21.5, 25.0)	24.9 (23.0, 26.8)	21.9 (20.6, 23.3)	18.4 (15.9, 20.9)	20.1 (18.6, 21.5)	24.4 (23.1, 25.8)	26.1 (24.7, 27.5)

Girls	Mean BMI in 1990 (kg/m^2)					Mean BMI in 2020 (kg/m^2)				
	Age-standardised	5 years	10 years	15 years	19 years	Age-standardised	5 years	10 years	15 years	19 years
urban-rural difference	0.2 (-0.6, 1.1)	0.4 (-0.7, 1.4)	0.3 (-0.6, 1.1)	0.2 (-0.7, 1.1)	0.1 (-1.0, 1.2)	0.2 (-0.6, 1.1)	0.4 (-0.7, 1.4)	0.3 (-0.6, 1.1)	0.2 (-0.7, 1.1)	0.1 (-1.0, 1.2)
Marshall Islands										
rural	19.6 (17.8, 21.4)	16.1 (13.2, 18.8)	17.8 (15.9, 19.7)	22.1 (20.4, 23.7)	23.7 (21.8, 25.5)	21.1 (19.6, 22.5)	17.5 (15.0, 20.1)	19.3 (17.6, 20.9)	23.5 (22.1, 24.9)	25.1 (23.7, 26.5)
urban	19.8 (18.0, 21.6)	16.4 (13.6, 19.2)	18.1 (16.1, 19.9)	22.2 (20.5, 23.8)	23.7 (21.9, 25.4)	21.3 (19.9, 22.7)	17.9 (15.3, 20.5)	19.5 (17.9, 21.1)	23.7 (22.3, 25.0)	25.2 (23.9, 26.4)
urban-rural difference	0.2 (-0.6, 1.1)	0.4 (-0.7, 1.4)	0.2 (-0.6, 1.1)	0.1 (-0.8, 1.1)	0.1 (-1.0, 1.2)	0.2 (-0.6, 1.0)	0.4 (-0.7, 1.4)	0.3 (-0.6, 1.1)	0.2 (-0.7, 1.0)	0.1 (-1.0, 1.1)
Micronesia (Federated States of)										
rural	20.0 (18.2, 21.9)	16.5 (13.7, 19.3)	18.1 (16.1, 20.1)	22.4 (20.7, 24.2)	24.7 (22.7, 26.5)	21.3 (19.8, 22.8)	17.8 (15.1, 20.4)	19.4 (17.7, 21.1)	23.7 (22.3, 25.2)	25.9 (24.5, 27.3)
urban	20.4 (18.6, 22.3)	16.8 (14.0, 19.8)	18.5 (16.5, 20.5)	22.9 (21.1, 24.6)	25.1 (23.2, 27.0)	21.8 (20.2, 23.3)	18.2 (15.5, 20.8)	19.8 (18.0, 21.5)	24.2 (22.7, 25.7)	26.4 (24.9, 27.9)
urban-rural difference	0.4 (-0.4, 1.2)	0.4 (-0.7, 1.5)	0.4 (-0.4, 1.3)	0.4 (-0.4, 1.3)	0.4 (-0.6, 1.4)	0.4 (-0.3, 1.3)	0.4 (-0.6, 1.5)	0.4 (-0.4, 1.3)	0.5 (-0.3, 1.3)	0.5 (-0.5, 1.5)
Nauru										
rural	21.4 (19.5, 23.2)	17.5 (14.8, 20.3)	19.1 (17.2, 20.9)	24.0 (22.2, 25.7)	27.0 (24.8, 29.1)	22.8 (21.4, 24.4)	19.0 (16.4, 21.6)	20.5 (19.0, 22.1)	25.4 (24.0, 26.9)	28.4 (26.5, 30.4)
urban	21.7 (19.8, 23.4)	17.9 (15.1, 20.6)	19.4 (17.4, 21.2)	24.2 (22.5, 25.8)	27.2 (25.2, 29.1)	23.1 (21.7, 24.5)	19.3 (16.8, 21.9)	20.8 (19.3, 22.3)	25.7 (24.4, 26.9)	28.6 (26.9, 30.4)
urban-rural difference	0.3 (-0.6, 1.1)	0.3 (-0.7, 1.4)	0.3 (-0.6, 1.1)	0.2 (-0.7, 1.1)	0.2 (-0.9, 1.3)	0.3 (-0.6, 1.1)	0.3 (-0.7, 1.3)	0.3 (-0.6, 1.1)	0.2 (-0.6, 1.1)	0.2 (-0.9, 1.2)
Niue										
rural	22.4 (20.6, 24.2)	18.6 (15.8, 21.3)	20.3 (18.3, 22.1)	25.0 (23.2, 26.8)	27.6 (25.3, 29.9)	24.7 (23.5, 25.9)	20.8 (18.4, 23.2)	22.5 (21.2, 23.8)	27.3 (26.2, 28.4)	29.8 (28.0, 31.7)
urban	22.7 (20.7, 24.5)	18.9 (16.1, 21.7)	20.6 (18.6, 22.4)	25.2 (23.3, 27.0)	27.7 (25.4, 30.0)	24.9 (23.7, 26.1)	21.1 (18.7, 23.6)	22.8 (21.5, 24.1)	27.4 (26.3, 28.6)	29.9 (28.1, 31.8)
urban-rural difference	0.2 (-0.6, 1.1)	0.4 (-0.7, 1.5)	0.3 (-0.6, 1.1)	0.2 (-0.7, 1.1)	0.1 (-1.0, 1.2)	0.2 (-0.6, 1.1)	0.4 (-0.7, 1.4)	0.3 (-0.6, 1.1)	0.2 (-0.7, 1.0)	0.1 (-1.0, 1.2)
Palau										
rural	20.0 (18.1, 22.0)	16.4 (13.5, 19.3)	18.1 (16.1, 20.2)	22.5 (20.5, 24.4)	24.4 (22.0, 26.7)	21.3 (19.6, 22.9)	17.6 (15.0, 20.3)	19.4 (17.6, 21.2)	23.7 (22.1, 25.3)	25.6 (23.7, 27.6)
urban	20.3 (18.3, 22.2)	16.8 (13.8, 19.7)	18.4 (16.3, 20.5)	22.6 (20.7, 24.6)	24.5 (22.2, 26.8)	21.5 (19.9, 23.0)	18.0 (15.4, 20.7)	19.6 (17.9, 21.4)	23.9 (22.3, 25.4)	25.7 (23.8, 27.5)
urban-rural difference	0.2 (-0.6, 1.1)	0.4 (-0.7, 1.5)	0.3 (-0.6, 1.1)	0.1 (-0.7, 1.0)	0.1 (-1.0, 1.2)	0.2 (-0.6, 1.0)	0.4 (-0.7, 1.4)	0.2 (-0.6, 1.1)	0.1 (-0.7, 1.0)	0.1 (-1.0, 1.1)
Samoa										
rural	21.4 (19.4, 23.4)	17.6 (14.7, 20.5)	19.2 (17.1, 21.3)	24.0 (22.0, 25.9)	26.8 (24.5, 29.0)	23.0 (21.3, 24.8)	19.2 (16.4, 22.0)	20.8 (19.0, 22.6)	25.6 (23.9, 27.3)	28.4 (26.4, 30.4)
urban	21.7 (19.6, 23.7)	18.0 (15.1, 20.8)	19.5 (17.4, 21.6)	24.2 (22.2, 26.1)	26.9 (24.6, 29.2)	23.2 (21.4, 25.1)	19.5 (16.8, 22.4)	21.1 (19.2, 23.0)	25.7 (24.0, 27.5)	28.5 (26.4, 30.6)
urban-rural difference	0.2 (-0.7, 1.1)	0.4 (-0.7, 1.4)	0.3 (-0.6, 1.1)	0.2 (-0.8, 1.1)	0.1 (-1.0, 1.2)	0.2 (-0.6, 1.1)	0.4 (-0.7, 1.4)	0.3 (-0.6, 1.1)	0.2 (-0.7, 1.1)	0.1 (-1.0, 1.2)
Tokelau										
rural	21.8 (19.8, 23.6)	17.9 (15.1, 20.7)	19.6 (17.5, 21.5)	24.3 (22.5, 26.1)	26.9 (25.0, 28.9)	23.3 (21.7, 24.8)	19.4 (16.7, 22.0)	21.1 (19.3, 22.8)	25.8 (24.4, 27.3)	28.4 (26.9, 30.0)
urban	22.0 (20.0, 23.8)	18.2 (15.4, 21.0)	19.8 (17.8, 21.8)	24.5 (22.6, 26.3)	27.0 (24.8, 29.1)	23.5 (21.8, 25.1)	19.8 (17.1, 22.4)	21.3 (19.6, 23.1)	26.0 (24.4, 27.6)	28.5 (26.7, 30.2)
urban-rural difference	0.2 (-0.6, 1.0)	0.3 (-0.7, 1.4)	0.2 (-0.6, 1.1)	0.1 (-0.8, 1.0)	0.0 (-1.1, 1.1)	0.2 (-0.6, 1.0)	0.4 (-0.7, 1.4)	0.2 (-0.6, 1.0)	0.1 (-0.7, 1.0)	0.0 (-1.0, 1.1)
Tonga										
rural	22.1 (20.3, 23.7)	18.2 (15.5, 20.8)	19.8 (18.0, 21.5)	24.6 (22.9, 26.3)	27.6 (25.9, 29.3)	24.0 (23.1, 25.0)	20.1 (17.9, 22.3)	21.8 (20.8, 22.7)	26.6 (25.8, 27.4)	29.6 (28.6, 30.6)
urban	22.3 (20.5, 24.0)	18.5 (15.8, 21.2)	20.0 (18.2, 21.7)	24.8 (23.0, 26.5)	27.7 (25.8, 29.6)	24.2 (23.2, 25.3)	20.5 (18.2, 22.7)	22.0 (20.9, 23.1)	26.7 (25.8, 27.8)	29.7 (28.4, 31.0)
urban-rural difference	0.2 (-0.6, 1.1)	0.3 (-0.7, 1.4)	0.3 (-0.6, 1.1)	0.2 (-0.7, 1.1)	0.1 (-1.0, 1.2)	0.2 (-0.6, 1.0)	0.3 (-0.7, 1.4)	0.2 (-0.6, 1.1)	0.1 (-0.7, 1.0)	0.1 (-1.0, 1.1)
Tuvalu										
rural	21.4 (19.5, 23.2)	17.6 (14.8, 20.4)	19.2 (17.3, 21.1)	23.9 (22.0, 25.7)	26.3 (23.8, 28.7)	22.9 (21.5, 24.3)	19.1 (16.6, 21.6)	20.8 (19.3, 22.3)	25.4 (24.1, 26.7)	27.8 (25.8, 29.8)
urban	21.6 (19.8, 23.4)	17.9 (15.2, 20.7)	19.5 (17.6, 21.4)	24.1 (22.3, 25.8)	26.4 (24.0, 28.8)	23.1 (21.7, 24.5)	19.5 (17.0, 22.0)	21.1 (19.6, 22.5)	25.6 (24.3, 26.9)	27.9 (26.0, 29.9)
urban-rural difference	0.2 (-0.6, 1.1)	0.4 (-0.7, 1.4)	0.3 (-0.6, 1.1)	0.2 (-0.7, 1.1)	0.1 (-1.0, 1.2)	0.3 (-0.6, 1.1)	0.4 (-0.7, 1.4)	0.3 (-0.6, 1.1)	0.2 (-0.7, 1.1)	0.1 (-0.9, 1.3)
**South Asia**										
Afghanistan										
rural	17.2 (15.7, 18.6)	14.3 (12.4, 16.1)	15.4 (14.0, 16.9)	19.3 (17.9, 20.8)	21.1 (19.6, 22.6)	18.9 (18.0, 19.8)	16.0 (14.6, 17.4)	17.1 (16.2, 18.1)	21.0 (20.2, 21.9)	22.8 (21.9, 23.7)
urban	17.6 (16.2, 19.1)	14.6 (12.7, 16.5)	15.8 (14.4, 17.3)	19.8 (18.3, 21.2)	21.6 (20.1, 23.2)	19.2 (18.4, 20.1)	16.2 (14.8, 17.7)	17.4 (16.5, 18.4)	21.4 (20.6, 22.3)	23.3 (22.4, 24.2)
urban-rural difference	0.4 (-0.2, 1.0)	0.3 (-0.5, 1.1)	0.4 (-0.2, 1.0)	0.5 (-0.2, 1.1)	0.5 (-0.2, 1.3)	0.3 (-0.1, 0.8)	0.2 (-0.5, 0.9)	0.3 (-0.2, 0.8)	0.4 (-0.1, 0.9)	0.5 (-0.1, 1.1)
Bangladesh										
rural	16.4 (15.0, 17.9)	13.2 (11.4, 15.1)	14.9 (13.5, 16.4)	18.7 (17.2, 20.2)	19.7 (18.3, 21.2)	17.4 (16.9, 18.0)	14.2 (12.9, 15.5)	15.9 (15.3, 16.5)	19.7 (19.1, 20.2)	20.7 (20.1, 21.3)
urban	16.9 (15.5, 18.4)	13.8 (12.0, 15.6)	15.4 (14.0, 16.9)	19.1 (17.7, 20.6)	20.1 (18.6, 21.6)	17.9 (17.4, 18.6)	14.8 (13.4, 16.1)	16.5 (15.8, 17.1)	20.1 (19.6, 20.7)	21.1 (20.4, 21.8)
urban-rural difference	0.5 (-0.2, 1.1)	0.6 (-0.2, 1.4)	0.5 (-0.2, 1.2)	0.4 (-0.2, 1.1)	0.4 (-0.4, 1.1)	0.5 (0.1, 0.9)	0.6 (-0.1, 1.3)	0.5 (0.1, 1.0)	0.5 (0.1, 0.9)	0.4 (-0.1, 1.0)
Bhutan										
rural	17.4 (15.9, 19.0)	14.4 (12.4, 16.5)	15.8 (14.2, 17.5)	19.6 (18.1, 21.1)	20.8 (19.1, 22.4)	18.8 (17.8, 19.8)	15.8 (14.1, 17.6)	17.2 (16.0, 18.5)	21.0 (20.1, 21.9)	22.2 (21.2, 23.2)
urban	17.9 (16.3, 19.4)	14.8 (12.8, 16.9)	16.2 (14.6, 17.9)	20.0 (18.5, 21.5)	21.3 (19.6, 22.9)	19.2 (18.3, 20.2)	16.2 (14.5, 17.9)	17.6 (16.4, 18.8)	21.4 (20.5, 22.3)	22.6 (21.6, 23.6)
urban-rural difference	0.4 (-0.2, 1.1)	0.4 (-0.4, 1.2)	0.4 (-0.2, 1.1)	0.4 (-0.3, 1.1)	0.4 (-0.4, 1.3)	0.4 (-0.2, 1.0)	0.4 (-0.4, 1.2)	0.4 (-0.2, 1.0)	0.4 (-0.2, 1.0)	0.4 (-0.3, 1.2)
India										

Girls	Mean BMI in 1990 (kg/m^2)					Mean BMI in 2020 (kg/m^2)				
	Age-standardised	5 years	10 years	15 years	19 years	Age-standardised	5 years	10 years	15 years	19 years
rural	15.6 (15.1, 16.1)	13.1 (12.6, 13.6)	14.2 (13.7, 14.7)	17.5 (17.0, 18.0)	18.7 (18.2, 19.2)	17.0 (16.5, 17.4)	14.5 (14.0, 14.9)	15.5 (15.1, 16.0)	18.9 (18.4, 19.3)	20.1 (19.6, 20.5)
urban	16.2 (15.6, 16.9)	13.5 (12.9, 14.2)	14.7 (14.1, 15.4)	18.1 (17.5, 18.8)	19.5 (18.8, 20.1)	17.4 (17.0, 17.8)	14.7 (14.2, 15.1)	15.9 (15.5, 16.3)	19.3 (18.9, 19.7)	20.6 (20.2, 21.1)
urban-rural difference	0.6 (0.2, 1.0)	0.4 (0.0, 0.9)	0.6 (0.2, 1.0)	0.7 (0.3, 1.1)	0.7 (0.3, 1.2)	0.4 (0.1, 0.6)	0.2 (0.0, 0.5)	0.4 (0.1, 0.6)	0.5 (0.2, 0.7)	0.6 (0.3, 0.8)
Nepal										
rural	16.8 (15.4, 18.1)	13.8 (11.9, 15.8)	15.4 (13.9, 16.8)	18.8 (17.5, 20.2)	19.8 (18.4, 21.1)	18.1 (17.4, 18.8)	15.1 (13.6, 16.7)	16.7 (15.9, 17.5)	20.1 (19.6, 20.7)	21.1 (20.5, 21.7)
urban	17.2 (15.8, 18.6)	14.3 (12.4, 16.3)	15.8 (14.3, 17.2)	19.2 (17.8, 20.5)	20.1 (18.8, 21.5)	18.3 (17.6, 19.1)	15.5 (13.9, 17.0)	16.9 (16.1, 17.8)	20.4 (19.8, 20.9)	21.3 (20.7, 21.9)
urban-rural difference	0.4 (-0.1, 1.0)	0.5 (-0.3, 1.3)	0.4 (-0.2, 1.0)	0.4 (-0.2, 0.9)	0.3 (-0.3, 0.9)	0.3 (-0.2, 0.7)	0.3 (-0.4, 1.1)	0.3 (-0.2, 0.8)	0.2 (-0.1, 0.6)	0.2 (-0.2, 0.5)
Pakistan										
rural	16.3 (15.7, 16.9)	13.8 (13.2, 14.5)	14.7 (14.0, 15.3)	18.3 (17.7, 18.9)	19.8 (19.1, 20.5)	18.0 (17.4, 18.6)	15.5 (14.9, 16.2)	16.4 (15.7, 17.0)	20.0 (19.4, 20.6)	21.5 (20.9, 22.1)
urban	16.6 (16.0, 17.3)	14.0 (13.3, 14.6)	14.9 (14.3, 15.5)	18.7 (18.0, 19.3)	20.3 (19.6, 20.9)	18.3 (17.7, 18.9)	15.6 (14.9, 16.3)	16.6 (15.9, 17.2)	20.3 (19.7, 20.9)	21.9 (21.2, 22.5)
urban-rural difference	0.3 (-0.1, 0.7)	0.2 (-0.3, 0.6)	0.3 (-0.1, 0.6)	0.4 (0.0, 0.7)	0.4 (0.0, 0.9)	0.2 (-0.1, 0.6)	0.1 (-0.3, 0.5)	0.2 (-0.1, 0.5)	0.3 (0.0, 0.7)	0.4 (0.0, 0.8)
Sri Lanka										
rural	16.4 (15.0, 17.8)	13.5 (11.6, 15.4)	14.9 (13.4, 16.4)	18.5 (17.1, 19.8)	19.5 (17.9, 21.0)	17.7 (16.7, 18.7)	14.9 (13.2, 16.6)	16.3 (15.2, 17.4)	19.8 (18.9, 20.7)	20.8 (19.7, 21.9)
urban	16.8 (15.4, 18.3)	13.9 (12.0, 15.8)	15.3 (13.8, 16.9)	18.9 (17.4, 20.3)	19.9 (18.2, 21.5)	18.1 (16.9, 19.2)	15.2 (13.5, 16.9)	16.6 (15.4, 17.8)	20.2 (19.1, 21.2)	21.2 (19.9, 22.5)
urban-rural difference	0.4 (-0.3, 1.1)	0.4 (-0.4, 1.2)	0.4 (-0.3, 1.1)	0.4 (-0.3, 1.1)	0.4 (-0.5, 1.3)	0.4 (-0.3, 1.0)	0.4 (-0.4, 1.2)	0.4 (-0.3, 1.0)	0.4 (-0.3, 1.1)	0.4 (-0.5, 1.2)
**Sub-Saharan Africa**										
Central Africa										
Angola										
rural	17.0 (15.2, 18.7)	14.1 (11.9, 16.4)	15.3 (13.5, 17.1)	19.1 (17.4, 20.8)	20.6 (18.7, 22.5)	17.8 (16.3, 19.3)	14.9 (12.8, 17.0)	16.1 (14.5, 17.7)	19.9 (18.4, 21.4)	21.4 (19.7, 23.1)
urban	17.7 (16.0, 19.5)	15.0 (12.8, 17.2)	16.0 (14.3, 17.9)	19.7 (18.0, 21.5)	21.1 (19.2, 23.1)	18.4 (17.0, 19.9)	15.8 (13.7, 17.8)	16.8 (15.2, 18.4)	20.5 (19.0, 21.9)	21.9 (20.2, 23.5)
urban-rural difference	0.7 (0.0, 1.4)	0.9 (0.0, 1.8)	0.8 (0.0, 1.5)	0.7 (-0.1, 1.4)	0.6 (-0.3, 1.4)	0.6 (0.0, 1.3)	0.8 (-0.1, 1.7)	0.7 (0.0, 1.4)	0.6 (-0.1, 1.3)	0.5 (-0.4, 1.3)
Central African Republic										
rural	17.3 (15.3, 19.3)	14.4 (12.0, 16.8)	15.6 (13.6, 17.6)	19.4 (17.3, 21.5)	20.7 (17.8, 23.7)	18.2 (16.2, 20.3)	15.4 (13.0, 17.8)	16.6 (14.6, 18.6)	20.4 (18.4, 22.4)	21.7 (18.8, 24.6)
urban	18.0 (16.0, 20.1)	15.3 (12.9, 17.8)	16.4 (14.4, 18.4)	20.1 (18.0, 22.2)	21.3 (18.3, 24.3)	18.9 (16.9, 21.0)	16.2 (13.8, 18.6)	17.3 (15.3, 19.3)	21.0 (18.9, 23.1)	22.2 (19.3, 25.1)
urban-rural difference	0.7 (0.0, 1.4)	0.9 (0.0, 1.8)	0.8 (0.0, 1.5)	0.7 (-0.1, 1.4)	0.6 (-0.3, 1.5)	0.7 (0.0, 1.3)	0.8 (0.0, 1.7)	0.7 (0.0, 1.4)	0.6 (-0.1, 1.3)	0.5 (-0.4, 1.4)
Congo										
rural	17.1 (16.4, 17.8)	13.9 (12.9, 14.9)	15.4 (14.6, 16.1)	19.4 (18.8, 20.0)	20.7 (20.1, 21.3)	17.7 (16.5, 18.9)	14.5 (13.1, 16.0)	16.0 (14.7, 17.3)	20.0 (18.9, 21.2)	21.3 (20.1, 22.5)
urban	17.6 (16.9, 18.3)	14.6 (13.8, 15.4)	15.9 (15.2, 16.7)	19.8 (19.1, 20.5)	21.0 (20.2, 21.7)	18.1 (16.9, 19.3)	15.2 (13.9, 16.4)	16.4 (15.2, 17.7)	20.3 (19.1, 21.5)	21.5 (20.3, 22.7)
urban-rural difference	0.5 (-0.1, 1.1)	0.7 (-0.2, 1.6)	0.6 (-0.1, 1.2)	0.4 (-0.2, 1.0)	0.3 (-0.4, 0.9)	0.4 (-0.1, 1.0)	0.6 (-0.2, 1.5)	0.5 (-0.1, 1.1)	0.3 (-0.2, 0.8)	0.2 (-0.4, 0.8)
DR Congo										
rural	17.4 (16.0, 18.8)	14.8 (12.7, 16.9)	16.0 (14.5, 17.5)	19.4 (18.1, 20.8)	20.3 (19.0, 21.7)	18.6 (17.6, 19.7)	15.9 (14.1, 17.8)	17.1 (16.0, 18.4)	20.6 (19.6, 21.6)	21.5 (20.5, 22.5)
urban	18.2 (16.8, 19.7)	15.8 (13.8, 17.8)	16.8 (15.3, 18.3)	20.1 (18.8, 21.5)	20.9 (19.5, 22.3)	19.5 (18.4, 20.6)	17.0 (15.3, 18.9)	18.1 (17.0, 19.3)	21.4 (20.4, 22.4)	22.1 (21.1, 23.2)
urban-rural difference	0.8 (0.1, 1.4)	1.0 (0.1, 1.9)	0.8 (0.2, 1.5)	0.7 (0.1, 1.2)	0.5 (-0.1, 1.1)	0.9 (0.4, 1.4)	1.1 (0.3, 2.0)	0.9 (0.4, 1.6)	0.8 (0.3, 1.3)	0.7 (0.1, 1.2)
Equatorial Guinea										
rural	17.3 (15.3, 19.4)	14.4 (12.0, 16.9)	15.7 (13.7, 17.7)	19.5 (17.4, 21.6)	20.7 (17.9, 23.7)	18.3 (16.2, 20.3)	15.4 (12.9, 17.8)	16.6 (14.6, 18.6)	20.4 (18.4, 22.4)	21.7 (18.7, 24.6)
urban	18.0 (16.0, 20.1)	15.3 (12.9, 17.8)	16.4 (14.4, 18.5)	20.1 (18.0, 22.3)	21.3 (18.4, 24.4)	18.9 (16.9, 21.0)	16.2 (13.7, 18.6)	17.3 (15.3, 19.3)	21.0 (18.9, 23.1)	22.2 (19.2, 25.1)
urban-rural difference	0.7 (0.0, 1.4)	0.9 (0.0, 1.8)	0.8 (0.0, 1.5)	0.7 (-0.1, 1.4)	0.6 (-0.4, 1.5)	0.7 (0.0, 1.4)	0.8 (-0.1, 1.7)	0.7 (0.0, 1.4)	0.6 (-0.1, 1.3)	0.5 (-0.4, 1.4)
Gabon										
rural	17.6 (16.1, 19.2)	14.7 (12.6, 16.9)	16.0 (14.4, 17.6)	19.8 (18.3, 21.3)	21.0 (19.3, 22.6)	18.6 (17.3, 19.9)	15.7 (13.8, 17.7)	17.0 (15.6, 18.4)	20.8 (19.6, 22.1)	22.0 (20.7, 23.3)
urban	18.4 (16.8, 19.9)	15.6 (13.5, 17.7)	16.7 (15.1, 18.4)	20.5 (19.0, 22.0)	21.6 (19.9, 23.1)	19.3 (18.0, 20.6)	16.5 (14.6, 18.4)	17.7 (16.3, 19.1)	21.4 (20.2, 22.6)	22.5 (21.1, 23.8)
urban-rural difference	0.7 (0.0, 1.4)	0.8 (-0.1, 1.7)	0.8 (0.0, 1.4)	0.7 (-0.1, 1.4)	0.6 (-0.2, 1.4)	0.6 (0.1, 1.2)	0.8 (-0.1, 1.6)	0.7 (0.1, 1.3)	0.6 (0.0, 1.2)	0.5 (-0.2, 1.2)
East Africa										
Burundi										
rural	16.6 (15.2, 18.2)	13.6 (11.6, 15.6)	14.7 (13.1, 16.3)	18.9 (17.4, 20.3)	20.7 (19.3, 22.3)	17.4 (16.5, 18.2)	14.3 (12.7, 15.9)	15.4 (14.4, 16.4)	19.6 (18.9, 20.4)	21.5 (20.7, 22.3)
urban	17.7 (16.2, 19.2)	14.9 (12.9, 17.0)	15.8 (14.3, 17.4)	19.8 (18.3, 21.3)	21.5 (20.0, 23.1)	18.2 (17.3, 19.1)	15.4 (13.8, 17.1)	16.4 (15.3, 17.4)	20.3 (19.5, 21.1)	22.0 (21.2, 22.8)
urban-rural difference	1.0 (0.4, 1.7)	1.3 (0.5, 2.2)	1.1 (0.5, 1.8)	0.9 (0.3, 1.5)	0.8 (0.0, 1.5)	0.8 (0.4, 1.3)	1.1 (0.4, 1.9)	0.9 (0.4, 1.5)	0.7 (0.3, 1.1)	0.5 (0.1, 1.0)
Comoros										
rural	17.7 (16.1, 19.2)	14.6 (12.6, 16.6)	15.9 (14.4, 17.6)	19.9 (18.4, 21.5)	21.2 (19.6, 22.7)	19.0 (17.8, 20.2)	15.9 (14.1, 17.7)	17.2 (15.9, 18.6)	21.2 (20.1, 22.4)	22.5 (21.3, 23.7)
urban	18.5 (17.0, 20.1)	15.7 (13.7, 17.7)	16.8 (15.3, 18.4)	20.7 (19.1, 22.2)	21.7 (20.1, 23.4)	19.5 (18.3, 20.7)	16.6 (14.8, 18.4)	17.8 (16.5, 19.1)	21.6 (20.5, 22.8)	22.7 (21.4, 23.9)
urban-rural difference	0.8 (0.2, 1.4)	1.1 (0.3, 1.9)	0.9 (0.3, 1.6)	0.7 (0.1, 1.3)	0.6 (-0.2, 1.3)	0.5 (0.0, 1.0)	0.7 (0.0, 1.5)	0.6 (0.0, 1.1)	0.4 (-0.1, 0.9)	0.2 (-0.4, 0.9)
Djibouti										

Girls	Mean BMI in 1990 (kg/m^2)					Mean BMI in 2020 (kg/m^2)				
	Age-standardised	5 years	10 years	15 years	19 years	Age-standardised	5 years	10 years	15 years	19 years
rural	17.1 (15.1, 19.2)	14.2 (11.9, 16.4)	15.4 (13.4, 17.4)	19.4 (17.3, 21.4)	20.7 (17.8, 23.5)	18.3 (16.4, 20.2)	15.3 (13.2, 17.6)	16.6 (14.7, 18.4)	20.5 (18.5, 22.5)	21.9 (19.1, 24.6)
urban	18.1 (16.1, 20.1)	15.3 (12.9, 17.6)	16.4 (14.4, 18.4)	20.3 (18.2, 22.3)	21.5 (18.6, 24.3)	19.0 (17.1, 20.9)	16.2 (13.9, 18.3)	17.3 (15.4, 19.2)	21.2 (19.2, 23.1)	22.5 (19.6, 25.2)
urban-rural difference	1.0 (0.3, 1.6)	1.1 (0.3, 1.9)	1.0 (0.4, 1.7)	0.9 (0.2, 1.6)	0.8 (0.0, 1.7)	0.7 (0.1, 1.3)	0.8 (0.0, 1.6)	0.7 (0.1, 1.4)	0.6 (0.0, 1.3)	0.6 (-0.3, 1.4)
Eritrea										
rural	16.5 (14.9, 18.1)	13.6 (11.5, 15.6)	14.8 (13.1, 16.5)	18.7 (17.1, 20.2)	19.8 (18.0, 21.6)	17.7 (16.1, 19.2)	14.8 (12.7, 16.7)	16.0 (14.4, 17.6)	19.8 (18.3, 21.3)	21.0 (19.2, 22.7)
urban	17.5 (15.8, 19.1)	14.7 (12.6, 16.7)	15.8 (14.1, 17.5)	19.6 (18.0, 21.1)	20.7 (18.8, 22.5)	18.4 (16.9, 19.9)	15.6 (13.6, 17.6)	16.7 (15.1, 18.3)	20.5 (19.0, 22.0)	21.6 (19.7, 23.3)
urban-rural difference	1.0 (0.3, 1.6)	1.1 (0.3, 2.0)	1.0 (0.4, 1.7)	0.9 (0.2, 1.6)	0.8 (0.0, 1.7)	0.7 (0.1, 1.3)	0.8 (0.1, 1.7)	0.7 (0.1, 1.4)	0.7 (0.0, 1.3)	0.6 (-0.3, 1.4)
Ethiopia										
rural	16.0 (14.6, 17.3)	13.1 (11.2, 15.0)	14.2 (12.8, 15.6)	18.1 (16.8, 19.4)	19.6 (18.4, 20.9)	17.0 (16.2, 17.8)	14.1 (12.5, 15.7)	15.3 (14.3, 16.2)	19.2 (18.5, 19.8)	20.7 (20.0, 21.4)
urban	17.2 (15.9, 18.5)	14.4 (12.6, 16.4)	15.5 (14.0, 16.9)	19.3 (18.0, 20.6)	20.7 (19.5, 22.0)	17.9 (17.0, 18.7)	15.1 (13.5, 16.7)	16.1 (15.2, 17.1)	20.0 (19.2, 20.7)	21.4 (20.7, 22.1)
urban-rural difference	1.2 (0.7, 1.8)	1.4 (0.6, 2.2)	1.3 (0.7, 1.9)	1.2 (0.7, 1.7)	1.1 (0.6, 1.6)	0.9 (0.4, 1.3)	1.0 (0.2, 1.7)	0.9 (0.4, 1.4)	0.8 (0.4, 1.2)	0.7 (0.3, 1.1)
Kenya										
rural	17.2 (15.7, 18.7)	14.3 (12.3, 16.2)	15.3 (13.8, 16.9)	19.4 (18.0, 20.8)	21.3 (19.8, 22.7)	18.5 (17.5, 19.5)	15.6 (14.0, 17.2)	16.6 (15.6, 17.7)	20.7 (19.8, 21.6)	22.6 (21.7, 23.4)
urban	18.4 (16.9, 19.9)	15.5 (13.6, 17.4)	16.5 (15.0, 18.1)	20.5 (19.1, 22.0)	22.4 (21.0, 23.8)	19.4 (18.5, 20.4)	16.5 (14.9, 18.1)	17.5 (16.5, 18.6)	21.5 (20.7, 22.4)	23.4 (22.5, 24.3)
urban-rural difference	1.2 (0.6, 1.7)	1.2 (0.5, 2.0)	1.2 (0.6, 1.8)	1.2 (0.6, 1.7)	1.1 (0.6, 1.7)	0.9 (0.4, 1.4)	0.9 (0.2, 1.7)	0.9 (0.4, 1.4)	0.9 (0.5, 1.3)	0.8 (0.4, 1.3)
Madagascar										
rural	16.7 (15.2, 18.2)	13.7 (11.7, 15.8)	15.1 (13.6, 16.7)	18.8 (17.3, 20.3)	19.7 (18.3, 21.2)	17.6 (16.3, 18.9)	14.7 (12.8, 16.6)	16.0 (14.7, 17.4)	19.8 (18.5, 21.0)	20.7 (19.4, 21.9)
urban	17.5 (16.0, 19.0)	14.8 (12.7, 16.8)	16.0 (14.4, 17.5)	19.5 (18.1, 21.0)	20.3 (18.9, 21.8)	18.1 (16.8, 19.4)	15.4 (13.6, 17.3)	16.6 (15.2, 17.9)	20.2 (18.9, 21.4)	21.0 (19.7, 22.2)
urban-rural difference	0.8 (0.3, 1.3)	1.0 (0.2, 1.8)	0.9 (0.3, 1.5)	0.7 (0.2, 1.2)	0.6 (0.0, 1.2)	0.5 (0.0, 1.0)	0.7 (0.0, 1.6)	0.6 (0.0, 1.2)	0.4 (-0.1, 0.9)	0.3 (-0.3, 0.8)
Malawi										
rural	17.3 (16.0, 18.6)	14.3 (12.4, 16.2)	15.4 (14.0, 16.8)	19.5 (18.3, 20.8)	21.1 (19.8, 22.3)	18.6 (17.8, 19.4)	15.6 (14.0, 17.2)	16.8 (15.8, 17.7)	20.9 (20.1, 21.6)	22.4 (21.7, 23.1)
urban	18.4 (17.1, 19.8)	15.5 (13.5, 17.4)	16.6 (15.2, 18.0)	20.7 (19.4, 21.9)	22.1 (20.8, 23.4)	19.4 (18.5, 20.3)	16.5 (14.9, 18.1)	17.6 (16.6, 18.6)	21.6 (20.9, 22.4)	23.1 (22.3, 23.9)
urban-rural difference	1.2 (0.7, 1.7)	1.2 (0.5, 2.0)	1.2 (0.6, 1.7)	1.1 (0.7, 1.6)	1.1 (0.6, 1.6)	0.8 (0.3, 1.3)	0.9 (0.1, 1.6)	0.8 (0.3, 1.4)	0.8 (0.4, 1.2)	0.7 (0.3, 1.2)
Mauritius										
rural	17.6 (16.1, 19.1)	14.7 (12.7, 16.7)	16.0 (14.5, 17.5)	19.7 (18.3, 21.2)	20.7 (18.9, 22.6)	19.1 (18.3, 19.9)	16.2 (14.7, 17.7)	17.5 (16.7, 18.3)	21.2 (20.6, 21.9)	22.3 (20.9, 23.5)
urban	18.5 (17.0, 20.0)	15.8 (13.8, 17.7)	17.0 (15.5, 18.4)	20.6 (19.1, 22.1)	21.5 (19.6, 23.4)	19.7 (18.9, 20.5)	17.0 (15.4, 18.5)	18.2 (17.3, 19.0)	21.8 (21.0, 22.6)	22.7 (21.3, 24.2)
urban-rural difference	0.9 (0.3, 1.6)	1.1 (0.3, 1.9)	1.0 (0.3, 1.6)	0.9 (0.2, 1.6)	0.8 (-0.1, 1.6)	0.6 (0.0, 1.2)	0.7 (-0.1, 1.6)	0.6 (0.0, 1.3)	0.5 (-0.1, 1.2)	0.5 (-0.4, 1.3)
Mozambique										
rural	17.4 (16.0, 18.8)	14.6 (12.8, 16.4)	15.8 (14.4, 17.2)	19.5 (18.1, 20.9)	20.7 (19.3, 22.1)	18.4 (17.4, 19.2)	15.5 (14.1, 17.0)	16.8 (15.8, 17.7)	20.5 (19.5, 21.3)	21.7 (20.7, 22.6)
urban	18.5 (17.2, 19.9)	15.7 (14.0, 17.5)	16.9 (15.5, 18.3)	20.6 (19.2, 22.0)	21.8 (20.4, 23.2)	19.0 (18.1, 19.9)	16.2 (14.8, 17.6)	17.4 (16.5, 18.3)	21.1 (20.2, 22.0)	22.3 (21.4, 23.2)
urban-rural difference	1.1 (0.6, 1.7)	1.1 (0.4, 1.9)	1.1 (0.5, 1.7)	1.1 (0.6, 1.6)	1.1 (0.5, 1.7)	0.7 (0.2, 1.1)	0.7 (0.0, 1.4)	0.7 (0.2, 1.2)	0.6 (0.2, 1.1)	0.6 (0.1, 1.1)
Rwanda										
rural	17.3 (16.0, 18.6)	14.2 (12.3, 16.0)	15.2 (13.8, 16.5)	19.7 (18.4, 20.9)	22.0 (20.7, 23.3)	18.6 (17.9, 19.2)	15.4 (13.9, 16.9)	16.4 (15.6, 17.1)	20.9 (20.4, 21.4)	23.2 (22.7, 23.8)
urban	18.5 (17.1, 19.7)	15.5 (13.6, 17.4)	16.4 (15.0, 17.7)	20.7 (19.4, 21.9)	22.9 (21.6, 24.2)	19.5 (18.8, 20.2)	16.5 (15.0, 18.1)	17.4 (16.6, 18.2)	21.7 (21.2, 22.3)	24.0 (23.4, 24.5)
urban-rural difference	1.1 (0.6, 1.6)	1.3 (0.6, 2.1)	1.2 (0.6, 1.7)	1.0 (0.5, 1.5)	0.9 (0.4, 1.4)	0.9 (0.5, 1.4)	1.1 (0.4, 1.9)	1.0 (0.5, 1.5)	0.8 (0.5, 1.2)	0.7 (0.3, 1.1)
Seychelles										
rural	18.1 (17.0, 19.2)	14.3 (13.2, 15.4)	17.0 (15.8, 18.0)	20.7 (19.6, 21.8)	20.2 (18.7, 21.7)	20.2 (19.8, 20.6)	16.3 (16.0, 16.7)	19.0 (18.7, 19.4)	22.7 (22.4, 23.1)	22.3 (21.1, 23.4)
urban	18.6 (17.5, 19.7)	14.8 (13.7, 15.9)	17.4 (16.3, 18.5)	21.1 (20.0, 22.2)	20.6 (19.1, 22.1)	20.3 (19.9, 20.7)	16.5 (16.1, 16.9)	19.1 (18.8, 19.5)	22.8 (22.4, 23.2)	22.3 (21.2, 23.5)
urban-rural difference	0.5 (0.1, 0.8)	0.5 (0.2, 0.9)	0.5 (0.2, 0.8)	0.4 (0.1, 0.8)	0.4 (0.0, 0.8)	0.1 (-0.1, 0.4)	0.2 (-0.1, 0.4)	0.1 (-0.1, 0.4)	0.1 (-0.2, 0.3)	0.0 (-0.3, 0.4)
Somalia										
rural	17.2 (15.2, 19.2)	14.2 (11.9, 16.5)	15.4 (13.4, 17.3)	19.4 (17.3, 21.4)	20.7 (17.8, 23.6)	18.3 (16.4, 20.3)	15.3 (13.2, 17.5)	16.6 (14.7, 18.4)	20.5 (18.6, 22.5)	21.9 (19.1, 24.7)
urban	18.1 (16.1, 20.1)	15.3 (12.9, 17.6)	16.4 (14.4, 18.4)	20.3 (18.2, 22.3)	21.5 (18.7, 24.4)	19.0 (17.1, 20.9)	16.2 (14.0, 18.3)	17.3 (15.4, 19.2)	21.2 (19.2, 23.1)	22.4 (19.6, 25.3)
urban-rural difference	1.0 (0.3, 1.6)	1.1 (0.3, 1.9)	1.0 (0.4, 1.7)	0.9 (0.2, 1.6)	0.8 (0.0, 1.7)	0.7 (0.1, 1.3)	0.8 (0.0, 1.6)	0.7 (0.1, 1.4)	0.6 (-0.1, 1.3)	0.6 (-0.3, 1.4)
South Sudan										
rural	17.3 (15.6, 19.1)	14.3 (12.2, 16.5)	15.5 (13.7, 17.3)	19.5 (17.8, 21.3)	21.1 (19.1, 23.3)	18.6 (17.2, 20.0)	15.5 (13.6, 17.5)	16.8 (15.2, 18.3)	20.8 (19.4, 22.2)	22.4 (20.5, 24.1)
urban	18.3 (16.6, 20.0)	15.4 (13.3, 17.6)	16.5 (14.7, 18.3)	20.5 (18.8, 22.2)	22.0 (19.9, 24.1)	19.3 (17.9, 20.6)	16.4 (14.4, 18.3)	17.5 (16.0, 19.0)	21.4 (20.1, 22.7)	22.9 (21.3, 24.6)
urban-rural difference	1.0 (0.3, 1.6)	1.1 (0.3, 1.9)	1.0 (0.4, 1.7)	0.9 (0.3, 1.6)	0.8 (0.0, 1.7)	0.7 (0.1, 1.4)	0.8 (0.1, 1.6)	0.7 (0.1, 1.4)	0.6 (0.0, 1.3)	0.6 (-0.2, 1.4)
Sudan										
rural	16.6 (14.9, 18.2)	13.7 (11.6, 15.8)	14.9 (13.2, 16.7)	18.7 (17.1, 20.3)	19.8 (18.0, 21.4)	17.5 (16.3, 18.8)	14.7 (12.9, 16.5)	15.9 (14.4, 17.4)	19.7 (18.5, 20.9)	20.7 (19.7, 21.8)
urban	17.6 (15.9, 19.3)	14.8 (12.7, 16.9)	16.0 (14.2, 17.8)	19.7 (18.1, 21.4)	20.8 (19.0, 22.5)	18.4 (17.1, 19.6)	15.5 (13.7, 17.4)	16.8 (15.3, 18.3)	20.5 (19.2, 21.7)	21.5 (20.3, 22.7)
urban-rural difference	1.0 (0.4, 1.7)	1.1 (0.3, 1.9)	1.0 (0.4, 1.7)	1.0 (0.3, 1.7)	1.0 (0.2, 1.8)	0.8 (0.3, 1.4)	0.9 (0.1, 1.7)	0.8 (0.2, 1.5)	0.8 (0.2, 1.4)	0.8 (0.1, 1.5)

Girls	Mean BMI in 1990 (kg/m^2)					Mean BMI in 2020 (kg/m^2)				
	Age-standardised	5 years	10 years	15 years	19 years	Age-standardised	5 years	10 years	15 years	19 years
Tanzania										
rural	17.0 (15.5, 18.4)	14.2 (12.5, 15.9)	14.9 (13.5, 16.4)	19.2 (17.8, 20.6)	21.2 (19.8, 22.7)	18.0 (17.2, 18.9)	15.3 (14.0, 16.6)	16.0 (15.1, 16.8)	20.2 (19.4, 21.0)	22.3 (21.4, 23.1)
urban	18.1 (16.6, 19.5)	15.5 (13.6, 17.2)	16.1 (14.6, 17.5)	20.2 (18.8, 21.6)	22.2 (20.8, 23.6)	18.9 (18.1, 19.8)	16.3 (14.9, 17.7)	16.9 (16.0, 17.9)	21.1 (20.3, 21.9)	23.1 (22.2, 23.9)
urban-rural difference	1.1 (0.5, 1.7)	1.2 (0.4, 2.0)	1.1 (0.5, 1.7)	1.0 (0.5, 1.6)	1.0 (0.3, 1.6)	0.9 (0.5, 1.4)	1.1 (0.3, 1.8)	1.0 (0.5, 1.5)	0.9 (0.5, 1.3)	0.8 (0.4, 1.3)
Uganda										
rural	17.6 (16.3, 18.9)	15.0 (13.5, 16.4)	15.7 (14.4, 17.0)	19.7 (18.4, 21.1)	21.2 (19.8, 22.5)	18.5 (17.7, 19.2)	15.8 (14.9, 16.7)	16.6 (15.7, 17.4)	20.6 (19.8, 21.4)	22.1 (21.2, 22.8)
urban	18.8 (17.4, 20.1)	16.4 (14.9, 18.0)	17.0 (15.6, 18.3)	20.8 (19.5, 22.1)	22.1 (20.7, 23.4)	19.3 (18.5, 20.2)	17.0 (15.9, 18.1)	17.5 (16.6, 18.4)	21.3 (20.5, 22.1)	22.6 (21.8, 23.4)
urban-rural difference	1.2 (0.7, 1.7)	1.5 (0.7, 2.3)	1.3 (0.7, 1.9)	1.0 (0.6, 1.6)	0.9 (0.3, 1.4)	0.9 (0.4, 1.3)	1.1 (0.4, 1.9)	0.9 (0.4, 1.4)	0.7 (0.3, 1.1)	0.6 (0.1, 1.0)
Zambia										
rural	17.0 (15.6, 18.4)	14.1 (12.1, 16.0)	15.3 (13.8, 16.7)	19.2 (17.8, 20.6)	20.7 (19.3, 22.1)	18.5 (17.6, 19.4)	15.5 (13.9, 17.2)	16.7 (15.7, 17.8)	20.7 (19.9, 21.5)	22.2 (21.4, 23.0)
urban	18.1 (16.7, 19.5)	15.3 (13.3, 17.2)	16.4 (14.9, 17.8)	20.2 (18.9, 21.6)	21.6 (20.3, 23.0)	19.3 (18.4, 20.3)	16.5 (14.8, 18.2)	17.6 (16.5, 18.6)	21.5 (20.6, 22.3)	22.9 (22.0, 23.7)
urban-rural difference	1.1 (0.5, 1.6)	1.2 (0.4, 2.0)	1.1 (0.5, 1.7)	1.0 (0.5, 1.5)	0.9 (0.4, 1.5)	0.8 (0.4, 1.3)	0.9 (0.2, 1.7)	0.9 (0.3, 1.4)	0.8 (0.4, 1.2)	0.7 (0.2, 1.1)
Southern Africa										
Botswana										
rural	16.7 (15.1, 18.4)	13.4 (11.2, 15.6)	15.0 (13.2, 16.7)	18.9 (17.3, 20.5)	20.8 (18.9, 22.7)	17.6 (16.2, 18.9)	14.3 (12.3, 16.2)	15.9 (14.3, 17.3)	19.8 (18.5, 21.0)	21.6 (20.0, 23.2)
urban	17.6 (15.9, 19.2)	14.3 (12.2, 16.5)	15.9 (14.1, 17.6)	19.7 (18.1, 21.4)	21.6 (19.7, 23.5)	18.2 (16.9, 19.5)	14.9 (13.0, 16.9)	16.5 (15.0, 17.9)	20.4 (19.1, 21.6)	22.2 (20.7, 23.7)
urban-rural difference	0.9 (0.2, 1.5)	0.9 (0.1, 1.8)	0.9 (0.2, 1.5)	0.8 (0.1, 1.6)	0.8 (-0.1, 1.7)	0.6 (0.0, 1.3)	0.6 (-0.2, 1.5)	0.6 (0.0, 1.3)	0.6 (-0.1, 1.3)	0.6 (-0.3, 1.5)
Lesotho										
rural	17.9 (16.3, 19.4)	14.4 (12.2, 16.5)	15.9 (14.2, 17.5)	20.1 (18.7, 21.6)	22.6 (21.1, 24.1)	18.6 (17.5, 19.8)	15.2 (13.3, 17.0)	16.6 (15.4, 17.9)	20.9 (19.9, 22.0)	23.4 (22.3, 24.5)
urban	18.7 (17.1, 20.2)	15.3 (13.2, 17.4)	16.7 (15.1, 18.3)	20.9 (19.4, 22.3)	23.3 (21.8, 24.8)	19.1 (18.0, 20.3)	15.8 (13.9, 17.6)	17.2 (15.9, 18.4)	21.4 (20.3, 22.4)	23.8 (22.7, 24.9)
urban-rural difference	0.8 (0.2, 1.4)	0.9 (0.1, 1.8)	0.8 (0.2, 1.5)	0.8 (0.2, 1.4)	0.7 (0.0, 1.4)	0.5 (0.0, 1.0)	0.6 (-0.2, 1.4)	0.5 (-0.1, 1.1)	0.4 (-0.1, 0.9)	0.4 (-0.2, 0.9)
Namibia										
rural	16.3 (14.7, 17.8)	13.0 (10.8, 15.1)	14.5 (12.9, 16.0)	18.4 (17.0, 19.9)	20.5 (19.0, 22.0)	17.1 (15.9, 18.3)	13.8 (11.9, 15.7)	15.3 (14.0, 16.6)	19.3 (18.2, 20.4)	21.3 (20.2, 22.5)
urban	17.3 (15.8, 18.8)	14.0 (11.9, 16.1)	15.5 (13.9, 17.0)	19.4 (18.0, 20.9)	21.5 (19.9, 23.0)	18.0 (16.7, 19.2)	14.7 (12.8, 16.6)	16.2 (14.8, 17.4)	20.1 (19.0, 21.3)	22.1 (21.0, 23.3)
urban-rural difference	1.0 (0.4, 1.6)	1.0 (0.2, 1.9)	1.0 (0.3, 1.7)	1.0 (0.4, 1.6)	1.0 (0.3, 1.7)	0.8 (0.3, 1.4)	0.9 (0.1, 1.7)	0.8 (0.3, 1.4)	0.8 (0.3, 1.3)	0.8 (0.2, 1.3)
South Africa										
rural	17.0 (15.9, 18.2)	13.7 (12.5, 14.9)	15.1 (13.9, 16.2)	19.2 (18.1, 20.4)	21.6 (20.5, 22.8)	19.5 (18.9, 20.1)	16.1 (15.5, 16.7)	17.5 (16.9, 18.1)	21.7 (21.1, 22.3)	24.1 (23.5, 24.7)
urban	18.0 (16.8, 19.2)	14.3 (13.1, 15.6)	15.9 (14.8, 17.2)	20.3 (19.1, 21.5)	22.9 (21.7, 24.1)	19.8 (19.2, 20.4)	16.2 (15.5, 16.8)	17.8 (17.2, 18.4)	22.1 (21.5, 22.7)	24.7 (24.0, 25.4)
urban-rural difference	1.0 (0.5, 1.5)	0.7 (0.1, 1.3)	0.9 (0.4, 1.4)	1.1 (0.6, 1.6)	1.2 (0.7, 1.8)	0.3 (0.0, 0.6)	0.0 (-0.3, 0.4)	0.2 (-0.1, 0.5)	0.4 (0.1, 0.8)	0.6 (0.2, 1.0)
Eswatini										
rural	18.5 (17.0, 20.1)	15.0 (12.8, 17.1)	16.6 (15.0, 18.3)	20.9 (19.4, 22.3)	22.9 (21.4, 24.5)	19.7 (18.5, 20.9)	16.2 (14.3, 18.1)	17.8 (16.5, 19.2)	22.1 (20.9, 23.2)	24.1 (22.9, 25.3)
urban	19.3 (17.7, 20.8)	15.9 (13.7, 17.9)	17.4 (15.8, 19.0)	21.6 (20.0, 23.1)	23.6 (22.0, 25.1)	20.2 (19.0, 21.5)	16.8 (14.9, 18.7)	18.3 (17.0, 19.7)	22.5 (21.3, 23.7)	24.5 (23.2, 25.8)
urban-rural difference	0.8 (0.1, 1.4)	0.9 (0.0, 1.8)	0.8 (0.1, 1.5)	0.7 (0.1, 1.4)	0.6 (-0.1, 1.4)	0.5 (-0.1, 1.1)	0.6 (-0.2, 1.4)	0.5 (-0.1, 1.1)	0.4 (-0.2, 1.0)	0.4 (-0.4, 1.1)
Zimbabwe										
rural	18.3 (17.2, 19.5)	14.9 (13.0, 16.8)	16.5 (15.3, 17.7)	20.5 (19.5, 21.7)	22.5 (21.5, 23.6)	18.4 (17.4, 19.4)	15.0 (13.2, 16.8)	16.6 (15.5, 17.7)	20.7 (19.7, 21.6)	22.6 (21.7, 23.6)
urban	19.2 (18.0, 20.4)	15.8 (13.9, 17.7)	17.4 (16.2, 18.7)	21.4 (20.3, 22.6)	23.4 (22.3, 24.5)	19.3 (18.3, 20.4)	15.9 (14.0, 17.7)	17.5 (16.3, 18.6)	21.5 (20.6, 22.5)	23.5 (22.5, 24.5)
urban-rural difference	0.9 (0.3, 1.5)	0.9 (0.1, 1.7)	0.9 (0.3, 1.5)	0.9 (0.3, 1.4)	0.9 (0.3, 1.5)	0.9 (0.4, 1.4)	0.9 (0.1, 1.7)	0.9 (0.4, 1.5)	0.9 (0.5, 1.3)	0.9 (0.4, 1.4)
West Africa										
Benin										
rural	17.4 (16.0, 18.7)	14.6 (12.7, 16.5)	15.8 (14.4, 17.2)	19.4 (18.1, 20.7)	20.6 (19.3, 21.9)	18.2 (17.4, 18.9)	15.4 (13.8, 17.0)	16.6 (15.7, 17.5)	20.2 (19.5, 20.8)	21.4 (20.8, 22.1)
urban	18.2 (16.8, 19.6)	15.5 (13.6, 17.5)	16.7 (15.2, 18.1)	20.1 (18.9, 21.5)	21.3 (20.0, 22.7)	18.9 (18.1, 19.7)	16.2 (14.6, 17.8)	17.4 (16.5, 18.3)	20.9 (20.2, 21.5)	22.0 (21.3, 22.7)
urban-rural difference	0.8 (0.3, 1.4)	0.9 (0.2, 1.7)	0.9 (0.3, 1.4)	0.8 (0.3, 1.3)	0.7 (0.2, 1.2)	0.7 (0.3, 1.2)	0.8 (0.1, 1.6)	0.8 (0.3, 1.3)	0.7 (0.3, 1.0)	0.6 (0.2, 1.0)
Burkina Faso										
rural	16.7 (15.3, 18.2)	14.6 (13.2, 16.1)	15.1 (13.6, 16.5)	18.5 (17.1, 19.9)	20.0 (18.6, 21.5)	17.8 (16.5, 19.0)	15.7 (14.4, 17.0)	16.1 (14.9, 17.4)	19.5 (18.3, 20.7)	21.1 (19.8, 22.3)
urban	18.0 (16.6, 19.5)	15.7 (14.1, 17.3)	16.3 (14.8, 17.8)	19.9 (18.5, 21.3)	21.6 (20.2, 23.1)	18.8 (17.6, 20.1)	16.5 (15.0, 18.0)	17.1 (15.8, 18.4)	20.7 (19.5, 21.9)	22.4 (21.2, 23.7)
urban-rural difference	1.3 (0.8, 1.9)	1.1 (0.2, 1.9)	1.2 (0.6, 1.9)	1.4 (0.9, 2.0)	1.6 (1.0, 2.2)	1.1 (0.6, 1.6)	0.8 (0.0, 1.6)	1.0 (0.4, 1.6)	1.2 (0.7, 1.7)	1.3 (0.8, 1.9)
Cabo Verde										
rural	17.0 (15.3, 18.7)	14.2 (12.1, 16.4)	15.4 (13.6, 17.1)	19.0 (17.3, 20.7)	20.4 (18.5, 22.4)	17.9 (16.6, 19.2)	15.1 (13.3, 17.0)	16.3 (14.8, 17.7)	19.9 (18.6, 21.1)	21.3 (20.0, 22.7)
urban	17.8 (16.1, 19.5)	15.2 (13.1, 17.3)	16.2 (14.4, 18.0)	19.7 (18.0, 21.4)	21.1 (19.1, 23.0)	18.4 (17.2, 19.7)	15.8 (14.0, 17.7)	16.9 (15.4, 18.3)	20.3 (19.1, 21.5)	21.7 (20.4, 23.0)
urban-rural difference	0.8 (0.1, 1.5)	1.0 (0.1, 1.8)	0.8 (0.2, 1.5)	0.7 (0.0, 1.4)	0.6 (-0.3, 1.5)	0.5 (-0.1, 1.1)	0.7 (-0.1, 1.5)	0.6 (-0.1, 1.2)	0.4 (-0.2, 1.1)	0.3 (-0.5, 1.2)
Cameroon										

Girls	Mean BMI in 1990 (kg/m^2)					Mean BMI in 2020 (kg/m^2)				
	Age-standardised	5 years	10 years	15 years	19 years	Age-standardised	5 years	10 years	15 years	19 years
rural	18.0 (16.7, 19.4)	15.3 (13.4, 17.1)	16.4 (15.0, 17.8)	20.0 (18.7, 21.4)	21.6 (20.3, 23.0)	19.0 (18.3, 19.8)	16.3 (14.8, 17.9)	17.4 (16.5, 18.2)	21.0 (20.4, 21.7)	22.6 (22.0, 23.3)
urban	19.0 (17.6, 20.4)	16.2 (14.3, 18.1)	17.3 (15.9, 18.8)	21.0 (19.7, 22.4)	22.6 (21.3, 24.0)	19.9 (19.2, 20.7)	17.1 (15.6, 18.6)	18.2 (17.4, 19.1)	21.9 (21.3, 22.6)	23.6 (23.0, 24.2)
urban-rural difference	1.0 (0.4, 1.5)	0.9 (0.1, 1.7)	1.0 (0.4, 1.5)	1.0 (0.5, 1.5)	1.0 (0.4, 1.6)	0.9 (0.5, 1.3)	0.8 (0.1, 1.6)	0.9 (0.4, 1.4)	0.9 (0.5, 1.3)	0.9 (0.5, 1.4)
Chad										
rural	16.9 (15.3, 18.4)	14.2 (12.1, 16.2)	15.4 (13.7, 17.0)	18.9 (17.4, 20.4)	20.0 (18.4, 21.6)	17.8 (16.8, 18.8)	15.0 (13.4, 16.7)	16.3 (15.1, 17.4)	19.7 (18.8, 20.6)	20.8 (19.9, 21.8)
urban	17.7 (16.1, 19.2)	15.1 (13.0, 17.2)	16.2 (14.6, 17.8)	19.6 (18.1, 21.1)	20.6 (19.0, 22.2)	18.4 (17.3, 19.4)	15.8 (14.1, 17.5)	16.9 (15.8, 18.1)	20.3 (19.3, 21.2)	21.3 (20.3, 22.3)
urban-rural difference	0.8 (0.2, 1.4)	0.9 (0.1, 1.7)	0.8 (0.2, 1.5)	0.7 (0.1, 1.4)	0.6 (-0.1, 1.4)	0.6 (0.1, 1.1)	0.7 (0.0, 1.5)	0.6 (0.1, 1.2)	0.5 (0.1, 1.0)	0.4 (-0.1, 1.0)
Côte d'Ivoire										
rural	17.9 (16.5, 19.2)	15.1 (13.2, 16.9)	16.3 (14.9, 17.7)	19.9 (18.6, 21.2)	21.0 (19.6, 22.3)	18.5 (17.3, 19.7)	15.7 (13.9, 17.5)	16.9 (15.6, 18.2)	20.5 (19.4, 21.7)	21.6 (20.4, 22.8)
urban	18.7 (17.4, 20.1)	16.1 (14.2, 17.9)	17.2 (15.8, 18.6)	20.7 (19.4, 22.0)	21.7 (20.3, 23.0)	19.1 (17.9, 20.3)	16.5 (14.7, 18.2)	17.6 (16.3, 18.9)	21.1 (20.0, 22.3)	22.1 (20.9, 23.2)
urban-rural difference	0.8 (0.3, 1.4)	1.0 (0.2, 1.8)	0.9 (0.3, 1.5)	0.8 (0.3, 1.3)	0.7 (0.1, 1.3)	0.6 (0.1, 1.2)	0.8 (0.0, 1.6)	0.7 (0.1, 1.3)	0.6 (0.1, 1.1)	0.5 (-0.1, 1.1)
Gambia										
rural	16.8 (15.3, 18.3)	14.1 (12.1, 16.1)	15.2 (13.7, 16.8)	18.7 (17.3, 20.2)	20.1 (18.6, 21.6)	17.6 (16.9, 18.2)	14.9 (13.4, 16.3)	16.0 (15.2, 16.8)	19.5 (19.0, 20.0)	20.9 (20.3, 21.5)
urban	17.6 (16.1, 19.2)	15.0 (13.0, 17.0)	16.1 (14.6, 17.7)	19.5 (18.1, 21.1)	20.9 (19.3, 22.5)	18.2 (17.5, 18.9)	15.6 (14.1, 17.1)	16.7 (15.8, 17.5)	20.1 (19.6, 20.6)	21.5 (20.8, 22.1)
urban-rural difference	0.8 (0.2, 1.4)	0.9 (0.1, 1.7)	0.9 (0.2, 1.5)	0.8 (0.2, 1.4)	0.8 (0.0, 1.5)	0.7 (0.2, 1.1)	0.7 (0.0, 1.5)	0.7 (0.2, 1.2)	0.6 (0.2, 1.0)	0.6 (0.1, 1.1)
Ghana										
rural	17.4 (16.0, 18.7)	14.4 (12.9, 15.8)	15.8 (14.4, 17.2)	19.4 (18.0, 20.8)	21.0 (19.6, 22.4)	18.6 (18.1, 19.2)	15.6 (14.9, 16.4)	17.0 (16.4, 17.6)	20.6 (20.1, 21.2)	22.2 (21.6, 22.8)
urban	18.4 (17.0, 19.8)	15.6 (14.1, 17.1)	16.9 (15.5, 18.3)	20.3 (19.0, 21.7)	21.8 (20.4, 23.2)	19.3 (18.7, 19.8)	16.5 (15.7, 17.2)	17.8 (17.2, 18.4)	21.2 (20.7, 21.8)	22.7 (22.1, 23.3)
urban-rural difference	1.0 (0.5, 1.6)	1.2 (0.5, 1.9)	1.1 (0.5, 1.7)	0.9 (0.5, 1.5)	0.8 (0.3, 1.4)	0.7 (0.3, 1.0)	0.9 (0.3, 1.4)	0.7 (0.4, 1.1)	0.6 (0.3, 0.9)	0.5 (0.1, 0.9)
Guinea										
rural	17.4 (16.0, 18.9)	14.4 (12.4, 16.5)	15.5 (13.9, 17.2)	19.2 (17.7, 20.8)	20.7 (19.1, 22.3)	18.6 (17.8, 19.4)	15.4 (13.5, 17.2)	16.5 (15.1, 17.8)	20.2 (18.9, 21.4)	21.7 (20.4, 22.9)
urban	18.2 (16.8, 19.7)	15.3 (13.3, 17.4)	16.3 (14.8, 18.0)	19.9 (18.4, 21.5)	21.4 (19.8, 23.0)	19.3 (18.5, 20.1)	16.1 (14.2, 17.9)	17.1 (15.7, 18.5)	20.7 (19.5, 21.9)	22.1 (20.8, 23.4)
urban-rural difference	0.8 (0.2, 1.3)	0.9 (0.1, 1.7)	0.8 (0.2, 1.4)	0.7 (0.1, 1.3)	0.7 (0.0, 1.4)	0.6 (0.2, 1.1)	0.7 (-0.1, 1.5)	0.6 (0.0, 1.2)	0.5 (0.0, 1.0)	0.5 (-0.1, 1.0)
Guinea Bissau										
rural	17.2 (15.7, 18.8)	14.7 (12.8, 16.7)	15.9 (14.4, 17.5)	19.4 (18.0, 20.8)	20.7 (19.3, 22.2)	18.1 (16.8, 19.4)	15.9 (14.4, 17.5)	17.1 (16.2, 18.0)	20.6 (19.9, 21.2)	21.9 (21.1, 22.6)
urban	18.0 (16.4, 19.5)	15.5 (13.6, 17.6)	16.7 (15.2, 18.3)	20.1 (18.7, 21.6)	21.4 (20.0, 22.9)	18.7 (17.4, 20.0)	16.6 (15.1, 18.2)	17.8 (16.8, 18.7)	21.2 (20.5, 21.9)	22.5 (21.7, 23.2)
urban-rural difference	0.8 (0.2, 1.4)	0.8 (0.0, 1.6)	0.8 (0.2, 1.4)	0.8 (0.2, 1.3)	0.7 (0.1, 1.4)	0.6 (0.1, 1.1)	0.7 (-0.1, 1.4)	0.6 (0.1, 1.2)	0.6 (0.2, 1.0)	0.6 (0.1, 1.1)
Liberia										
rural	17.3 (15.8, 18.7)	14.5 (12.5, 16.5)	15.7 (14.1, 17.2)	19.3 (17.8, 20.7)	20.7 (19.3, 22.2)	18.5 (17.7, 19.2)	15.7 (14.1, 17.2)	16.9 (16.0, 17.8)	20.5 (19.9, 21.1)	21.9 (21.2, 22.5)
urban	18.0 (16.6, 19.5)	15.4 (13.4, 17.4)	16.5 (14.9, 18.0)	20.0 (18.5, 21.4)	21.3 (19.8, 22.8)	19.0 (18.3, 19.8)	16.4 (14.9, 17.9)	17.5 (16.6, 18.4)	21.0 (20.3, 21.6)	22.3 (21.6, 22.9)
urban-rural difference	0.8 (0.2, 1.4)	0.9 (0.2, 1.7)	0.8 (0.2, 1.4)	0.7 (0.1, 1.3)	0.6 (-0.1, 1.3)	0.6 (0.1, 1.0)	0.7 (0.0, 1.5)	0.6 (0.1, 1.1)	0.5 (0.1, 0.9)	0.4 (0.0, 0.8)
Mali										
rural	17.0 (15.7, 18.3)	14.3 (12.4, 16.2)	15.4 (14.0, 16.8)	19.0 (17.7, 20.2)	20.4 (19.1, 21.7)	18.3 (17.6, 19.1)	15.6 (14.1, 17.2)	16.7 (15.9, 17.6)	20.3 (19.6, 20.9)	21.7 (21.0, 22.4)
urban	18.0 (16.6, 19.3)	15.3 (13.4, 17.2)	16.4 (14.9, 17.8)	19.9 (18.6, 21.2)	21.3 (20.0, 22.6)	19.0 (18.2, 19.8)	16.3 (14.7, 17.9)	17.4 (16.5, 18.3)	21.0 (20.3, 21.6)	22.4 (21.6, 23.1)
urban-rural difference	0.9 (0.4, 1.5)	1.0 (0.2, 1.8)	1.0 (0.4, 1.5)	0.9 (0.4, 1.4)	0.9 (0.4, 1.5)	0.7 (0.2, 1.1)	0.7 (-0.1, 1.4)	0.7 (0.2, 1.2)	0.7 (0.3, 1.1)	0.7 (0.2, 1.1)
Mauritania										
rural	17.9 (16.4, 19.5)	15.1 (13.1, 17.2)	16.1 (14.5, 17.8)	19.9 (18.4, 21.4)	21.9 (20.3, 23.5)	19.1 (18.4, 19.9)	16.4 (14.8, 17.9)	17.4 (16.4, 18.3)	21.1 (20.5, 21.8)	23.1 (22.3, 23.9)
urban	18.9 (17.3, 20.4)	16.0 (14.0, 18.1)	17.1 (15.4, 18.7)	20.9 (19.3, 22.4)	22.9 (21.3, 24.5)	20.1 (19.3, 20.9)	17.3 (15.7, 18.9)	18.3 (17.3, 19.3)	22.1 (21.4, 22.9)	24.2 (23.3, 25.0)
urban-rural difference	1.0 (0.3, 1.6)	0.9 (0.1, 1.7)	0.9 (0.2, 1.6)	1.0 (0.3, 1.6)	1.0 (0.2, 1.8)	1.0 (0.5, 1.5)	0.9 (0.1, 1.8)	1.0 (0.4, 1.5)	1.0 (0.5, 1.5)	1.0 (0.4, 1.7)
Niger										
rural	16.4 (14.9, 17.9)	13.7 (11.7, 15.7)	14.6 (13.1, 16.2)	18.3 (16.8, 19.8)	20.6 (19.0, 22.1)	17.4 (16.2, 18.6)	14.6 (12.8, 16.4)	15.6 (14.3, 16.9)	19.3 (18.1, 20.4)	21.5 (20.3, 22.7)
urban	17.2 (15.7, 18.7)	14.6 (12.6, 16.6)	15.5 (13.9, 17.1)	19.1 (17.6, 20.6)	21.2 (19.7, 22.8)	18.0 (16.8, 19.2)	15.4 (13.6, 17.2)	16.2 (15.0, 17.6)	19.8 (18.7, 21.0)	22.0 (20.7, 23.2)
urban-rural difference	0.8 (0.2, 1.4)	0.9 (0.1, 1.8)	0.8 (0.2, 1.5)	0.7 (0.2, 1.3)	0.7 (0.0, 1.3)	0.6 (0.1, 1.1)	0.8 (0.0, 1.5)	0.7 (0.1, 1.2)	0.6 (0.1, 1.0)	0.5 (-0.1, 1.0)
Nigeria										
rural	17.0 (15.5, 18.4)	14.1 (12.7, 15.6)	15.3 (13.9, 16.8)	19.0 (17.6, 20.4)	20.4 (19.0, 21.9)	17.6 (16.9, 18.4)	14.8 (14.0, 15.6)	16.0 (15.2, 16.8)	19.6 (18.9, 20.4)	21.1 (20.3, 21.8)
urban	17.4 (16.0, 18.8)	14.7 (13.2, 16.1)	15.8 (14.3, 17.2)	19.4 (18.0, 20.8)	20.8 (19.3, 22.3)	18.0 (17.2, 18.8)	15.3 (14.3, 16.2)	16.4 (15.5, 17.2)	20.0 (19.2, 20.8)	21.4 (20.6, 22.2)
urban-rural difference	0.4 (-0.1, 1.0)	0.5 (-0.2, 1.2)	0.5 (-0.1, 1.0)	0.4 (-0.1, 0.9)	0.4 (-0.2, 0.9)	0.4 (-0.1, 0.8)	0.4 (-0.2, 1.1)	0.4 (-0.1, 0.9)	0.3 (-0.1, 0.7)	0.3 (-0.1, 0.7)
Sao Tome and Principe										
rural	17.7 (16.1, 19.2)	14.7 (12.7, 16.8)	16.0 (14.4, 17.7)	19.8 (18.3, 21.3)	21.1 (19.4, 22.8)	18.5 (17.4, 19.5)	15.5 (13.8, 17.2)	16.9 (15.6, 18.1)	20.6 (19.6, 21.5)	21.9 (21.0, 22.8)
urban	18.2 (16.6, 19.8)	15.7 (13.7, 17.8)	16.7 (15.1, 18.4)	20.1 (18.6, 21.7)	21.2 (19.6, 22.8)	18.7 (17.7, 19.6)	16.2 (14.5, 17.8)	17.2 (16.0, 18.3)	20.6 (19.7, 21.5)	21.7 (20.8, 22.5)
urban-rural difference	0.6 (-0.1, 1.2)	1.0 (0.1, 1.8)	0.7 (0.0, 1.4)	0.4 (-0.3, 1.1)	0.1 (-0.6, 0.9)	0.2 (-0.4, 0.7)	0.6 (-0.2, 1.4)	0.3 (-0.3, 0.9)	0.0 (-0.6, 0.5)	-0.2 (-0.9, 0.4)

Girls	Mean BMI in 1990 (kg/m²)					Mean BMI in 2020 (kg/m²)				
	Age-standardised	5 years	10 years	15 years	19 years	Age-standardised	5 years	10 years	15 years	19 years
Senegal										
rural	16.5 (15.0, 18.0)	13.8 (11.9, 15.9)	14.9 (13.4, 16.5)	18.4 (16.9, 19.9)	19.8 (18.2, 21.3)	17.2 (16.0, 18.4)	14.5 (12.8, 16.3)	15.6 (14.3, 17.0)	19.1 (17.9, 20.3)	20.5 (19.3, 21.7)
urban	17.2 (15.7, 18.8)	14.7 (12.6, 16.7)	15.7 (14.1, 17.3)	19.1 (17.6, 20.6)	20.5 (18.9, 22.0)	17.8 (16.6, 19.1)	15.3 (13.5, 17.1)	16.3 (15.0, 17.6)	19.7 (18.6, 20.9)	21.1 (19.8, 22.3)
urban-rural difference	0.8 (0.2, 1.4)	0.8 (0.0, 1.7)	0.8 (0.2, 1.4)	0.8 (0.2, 1.3)	0.7 (0.1, 1.3)	0.7 (0.2, 1.2)	0.7 (0.0, 1.5)	0.7 (0.1, 1.3)	0.6 (0.2, 1.1)	0.6 (0.1, 1.2)
Sierra Leone										
rural	17.8 (16.2, 19.3)	15.0 (13.0, 17.0)	16.2 (14.6, 17.8)	19.8 (18.3, 21.3)	21.2 (19.7, 22.7)	18.5 (17.7, 19.2)	15.7 (14.2, 17.3)	16.9 (16.0, 17.8)	20.5 (19.9, 21.1)	21.9 (21.2, 22.5)
urban	18.6 (17.1, 20.1)	15.9 (13.9, 17.9)	17.0 (15.4, 18.6)	20.6 (19.1, 22.0)	21.9 (20.4, 23.5)	19.1 (18.4, 19.9)	16.4 (14.8, 18.0)	17.5 (16.7, 18.5)	21.1 (20.4, 21.7)	22.4 (21.7, 23.1)
urban-rural difference	0.8 (0.2, 1.4)	0.9 (0.1, 1.7)	0.8 (0.2, 1.5)	0.8 (0.2, 1.4)	0.8 (0.1, 1.5)	0.6 (0.2, 1.1)	0.7 (0.0, 1.5)	0.7 (0.2, 1.2)	0.6 (0.2, 1.0)	0.6 (0.1, 1.0)
Togo										
rural	17.6 (16.1, 19.1)	14.8 (12.9, 16.9)	16.0 (14.4, 17.6)	19.6 (18.1, 21.2)	20.9 (19.4, 22.4)	18.6 (17.5, 19.7)	15.8 (14.1, 17.6)	17.0 (15.8, 18.2)	20.6 (19.6, 21.6)	21.9 (20.8, 22.9)
urban	18.5 (17.0, 20.1)	15.7 (13.7, 17.8)	16.9 (15.3, 18.5)	20.5 (19.0, 22.1)	21.8 (20.2, 23.4)	19.4 (18.3, 20.5)	16.6 (14.8, 18.4)	17.8 (16.5, 19.0)	21.4 (20.4, 22.5)	22.7 (21.6, 23.8)
urban-rural difference	0.9 (0.3, 1.6)	0.9 (0.1, 1.7)	0.9 (0.3, 1.6)	0.9 (0.3, 1.6)	1.0 (0.2, 1.7)	0.8 (0.3, 1.3)	0.8 (0.0, 1.6)	0.8 (0.3, 1.4)	0.8 (0.3, 1.3)	0.8 (0.3, 1.4)

Boys	Mean BMI in 1990 (kg/m^2)					Mean BMI in 2020 (kg/m^2)				
	Age-standardised	5 years	10 years	15 years	19 years	Age-standardised	5 years	10 years	15 years	19 years
**Central and eastern Europe**										
Central Europe										
Albania										
rural	18.3 (17.0, 19.7)	15.0 (13.6, 16.4)	17.0 (15.6, 18.4)	20.1 (18.8, 21.5)	22.3 (20.9, 23.7)	18.8 (18.2, 19.5)	15.5 (14.7, 16.2)	17.5 (16.9, 18.1)	20.6 (20.0, 21.3)	22.8 (22.0, 23.5)
urban	19.1 (17.8, 20.5)	16.0 (14.7, 17.4)	17.8 (16.5, 19.2)	20.7 (19.4, 22.1)	22.7 (21.4, 24.1)	19.4 (18.7, 20.0)	16.3 (15.6, 17.0)	18.1 (17.4, 18.8)	21.0 (20.3, 21.7)	23.0 (22.2, 23.8)
urban-rural difference	0.8 (0.1, 1.3)	1.1 (0.3, 1.7)	0.8 (0.2, 1.4)	0.6 (0.0, 1.2)	0.4 (-0.3, 1.1)	0.5 (0.2, 0.9)	0.8 (0.3, 1.3)	0.6 (0.2, 1.0)	0.4 (0.0, 0.8)	0.2 (-0.2, 0.7)
Bosnia and Herzegovina										
rural	18.6 (16.8, 20.5)	15.1 (12.2, 18.1)	17.5 (15.6, 19.4)	20.3 (18.6, 22.1)	22.1 (20.3, 23.9)	19.6 (18.0, 21.3)	16.2 (13.3, 19.0)	18.5 (16.8, 20.2)	21.4 (19.8, 23.0)	23.1 (21.5, 24.7)
urban	19.0 (17.1, 20.8)	15.6 (12.6, 18.5)	17.9 (16.0, 19.7)	20.7 (18.9, 22.4)	22.4 (20.6, 24.2)	19.7 (18.1, 21.3)	16.3 (13.4, 19.1)	18.6 (16.9, 20.3)	21.4 (19.8, 23.0)	23.1 (21.5, 24.7)
urban-rural difference	0.4 (-0.3, 1.0)	0.4 (-0.5, 1.3)	0.4 (-0.3, 1.1)	0.4 (-0.3, 1.0)	0.3 (-0.4, 1.0)	0.0 (-0.6, 0.7)	0.1 (-0.8, 1.0)	0.1 (-0.6, 0.7)	0.0 (-0.6, 0.6)	0.0 (-0.7, 0.7)
Bulgaria										
rural	18.5 (17.5, 19.6)	15.1 (14.0, 16.3)	17.6 (16.5, 18.7)	20.2 (19.1, 21.3)	21.5 (19.8, 23.1)	19.6 (18.9, 20.2)	16.2 (15.4, 16.9)	18.6 (17.9, 19.3)	21.2 (20.4, 22.0)	22.5 (21.0, 24.1)
urban	18.8 (17.9, 19.8)	15.6 (14.5, 16.6)	17.9 (16.9, 18.8)	20.4 (19.5, 21.4)	21.7 (20.2, 23.2)	19.5 (18.8, 20.1)	16.2 (15.5, 16.9)	18.5 (17.9, 19.2)	21.1 (20.3, 21.8)	22.4 (21.0, 23.7)
urban-rural difference	0.3 (-0.2, 0.9)	0.4 (-0.2, 1.1)	0.3 (-0.2, 0.9)	0.3 (-0.3, 0.9)	0.2 (-0.5, 0.9)	-0.1 (-0.5, 0.3)	0.0 (-0.5, 0.5)	-0.1 (-0.5, 0.3)	-0.1 (-0.6, 0.4)	-0.2 (-0.8, 0.5)
Croatia										
rural	18.7 (17.4, 20.0)	15.4 (14.1, 16.7)	17.7 (16.4, 19.0)	20.5 (19.2, 21.8)	21.6 (20.2, 23.0)	19.7 (19.2, 20.3)	16.4 (15.8, 17.0)	18.7 (18.1, 19.2)	21.5 (20.9, 22.1)	22.6 (21.8, 23.4)
urban	19.1 (17.8, 20.4)	15.6 (14.3, 16.9)	18.0 (16.7, 19.3)	20.9 (19.6, 22.3)	22.1 (20.7, 23.5)	19.7 (19.1, 20.2)	16.2 (15.6, 16.8)	18.5 (18.0, 19.1)	21.5 (20.9, 22.1)	22.6 (21.8, 23.4)
urban-rural difference	0.3 (-0.2, 0.8)	0.2 (-0.4, 0.8)	0.3 (-0.2, 0.8)	0.4 (-0.1, 0.9)	0.5 (-0.1, 1.1)	-0.1 (-0.5, 0.3)	-0.2 (-0.6, 0.2)	-0.1 (-0.5, 0.2)	0.0 (-0.4, 0.4)	0.0 (-0.5, 0.5)
Czechia										
rural	18.4 (17.1, 19.6)	15.2 (13.8, 16.5)	17.3 (16.0, 18.6)	20.0 (18.6, 21.3)	21.7 (20.0, 23.3)	19.0 (18.3, 19.6)	15.8 (15.2, 16.4)	17.9 (17.3, 18.5)	20.6 (19.8, 21.4)	22.4 (21.1, 23.6)
urban	18.6 (17.3, 19.8)	15.3 (14.1, 16.6)	17.5 (16.3, 18.7)	20.2 (18.9, 21.5)	22.0 (20.3, 23.5)	18.8 (18.2, 19.4)	15.6 (15.0, 16.1)	17.7 (17.1, 18.3)	20.4 (19.7, 21.1)	22.2 (21.0, 23.4)
urban-rural difference	0.2 (-0.3, 0.7)	0.2 (-0.4, 0.8)	0.2 (-0.3, 0.7)	0.2 (-0.4, 0.8)	0.2 (-0.5, 1.0)	-0.2 (-0.6, 0.2)	-0.2 (-0.7, 0.2)	-0.2 (-0.6, 0.2)	-0.2 (-0.7, 0.3)	-0.2 (-0.9, 0.5)
Hungary										
rural	18.7 (17.3, 20.1)	14.8 (13.2, 16.2)	17.9 (16.5, 19.2)	20.5 (19.1, 21.8)	21.8 (20.3, 23.2)	20.4 (20.0, 20.8)	16.5 (15.9, 17.1)	19.6 (19.2, 20.0)	22.2 (21.8, 22.6)	23.5 (22.9, 24.0)
urban	19.1 (17.7, 20.4)	15.0 (13.6, 16.4)	18.2 (16.8, 19.6)	20.9 (19.5, 22.3)	22.3 (20.8, 23.6)	20.4 (20.0, 20.7)	16.3 (15.8, 16.9)	19.5 (19.2, 19.9)	22.2 (21.9, 22.6)	23.5 (23.1, 24.0)
urban-rural difference	0.4 (-0.2, 0.9)	0.3 (-0.4, 0.9)	0.3 (-0.2, 0.9)	0.4 (-0.1, 1.0)	0.5 (-0.1, 1.1)	-0.1 (-0.3, 0.2)	-0.2 (-0.6, 0.3)	-0.1 (-0.4, 0.2)	0.0 (-0.3, 0.3)	0.0 (-0.4, 0.5)
North Macedonia										
rural	18.4 (17.0, 19.8)	15.0 (13.4, 16.5)	17.2 (15.8, 18.6)	20.1 (18.6, 21.7)	21.9 (19.8, 24.1)	19.4 (18.6, 20.2)	16.0 (15.1, 16.9)	18.2 (17.5, 19.0)	21.1 (20.1, 22.2)	22.9 (21.1, 24.7)
urban	19.0 (17.6, 20.4)	15.7 (14.3, 17.2)	17.9 (16.5, 19.3)	20.6 (19.0, 22.2)	22.3 (20.1, 24.4)	19.7 (18.9, 20.5)	16.4 (15.5, 17.3)	18.6 (17.8, 19.3)	21.3 (20.2, 22.4)	23.0 (21.1, 24.8)
urban-rural difference	0.6 (0.0, 1.1)	0.8 (0.1, 1.4)	0.6 (0.0, 1.2)	0.5 (-0.1, 1.1)	0.4 (-0.4, 1.1)	0.3 (-0.2, 0.7)	0.5 (0.0, 0.9)	0.3 (-0.1, 0.7)	0.2 (-0.3, 0.7)	0.1 (-0.6, 0.7)
Montenegro										
rural	18.5 (17.8, 19.2)	14.8 (13.9, 15.7)	17.3 (16.6, 18.1)	20.3 (19.7, 21.0)	22.1 (21.5, 22.7)	19.6 (19.2, 20.0)	15.9 (15.3, 16.4)	18.4 (17.9, 18.8)	21.4 (20.9, 21.9)	23.2 (22.5, 23.9)
urban	19.0 (18.3, 19.7)	15.5 (14.6, 16.4)	17.9 (17.1, 18.7)	20.8 (20.1, 21.5)	22.5 (21.8, 23.1)	19.8 (19.4, 20.2)	16.3 (15.7, 16.8)	18.7 (18.3, 19.1)	21.6 (21.1, 22.0)	23.2 (22.6, 23.9)
urban-rural difference	0.5 (-0.1, 1.1)	0.7 (0.0, 1.4)	0.6 (0.0, 1.2)	0.4 (-0.2, 1.1)	0.3 (-0.4, 1.0)	0.2 (-0.1, 0.6)	0.4 (0.0, 0.8)	0.3 (0.0, 0.6)	0.1 (-0.3, 0.6)	0.0 (-0.5, 0.6)
Poland										
rural	18.6 (17.6, 19.5)	15.2 (14.2, 16.2)	17.4 (16.5, 18.4)	20.3 (19.3, 21.2)	21.9 (21.0, 22.9)	19.4 (19.0, 19.9)	16.0 (15.5, 16.6)	18.3 (17.8, 18.8)	21.2 (20.7, 21.6)	22.8 (22.3, 23.3)
urban	18.7 (17.7, 19.6)	15.3 (14.3, 16.3)	17.6 (16.6, 18.5)	20.4 (19.5, 21.4)	22.1 (21.1, 23.0)	19.3 (18.8, 19.7)	15.8 (15.4, 16.3)	18.1 (17.7, 18.6)	21.0 (20.5, 21.5)	22.6 (22.1, 23.2)
urban-rural difference	0.1 (-0.3, 0.5)	0.1 (-0.4, 0.6)	0.1 (-0.3, 0.6)	0.1 (-0.3, 0.6)	0.2 (-0.3, 0.6)	-0.2 (-0.5, 0.1)	-0.2 (-0.6, 0.2)	-0.2 (-0.5, 0.1)	-0.2 (-0.5, 0.2)	-0.2 (-0.5, 0.2)
Romania										
rural	17.9 (16.8, 19.1)	14.5 (13.4, 15.7)	16.7 (15.6, 17.8)	19.6 (18.4, 20.7)	21.9 (20.7, 23.2)	19.0 (18.5, 19.5)	15.6 (15.0, 16.1)	17.7 (17.2, 18.2)	20.6 (20.1, 21.1)	23.0 (22.2, 23.7)
urban	18.5 (17.4, 19.6)	15.4 (14.2, 16.5)	17.3 (16.2, 18.5)	20.0 (18.9, 21.2)	22.2 (21.0, 23.5)	19.2 (18.7, 19.7)	16.1 (15.6, 16.6)	18.0 (17.5, 18.5)	20.7 (20.2, 21.3)	22.9 (22.2, 23.6)
urban-rural difference	0.6 (0.1, 1.0)	0.8 (0.3, 1.4)	0.6 (0.2, 1.1)	0.5 (0.0, 0.9)	0.3 (-0.2, 0.8)	0.2 (-0.1, 0.5)	0.5 (0.2, 0.8)	0.3 (0.0, 0.6)	0.1 (-0.3, 0.5)	0.0 (-0.5, 0.4)
Serbia										
rural	18.8 (17.6, 20.0)	15.0 (13.7, 16.3)	17.7 (16.4, 18.9)	20.6 (19.4, 21.8)	22.5 (21.2, 23.8)	20.2 (19.6, 20.8)	16.5 (15.7, 17.2)	19.1 (18.5, 19.7)	22.0 (21.4, 22.7)	24.0 (23.2, 24.7)
urban	19.0 (17.8, 20.2)	15.3 (13.9, 16.6)	17.9 (16.7, 19.1)	20.8 (19.6, 22.0)	22.7 (21.4, 23.9)	20.0 (19.4, 20.6)	16.3 (15.6, 17.0)	18.9 (18.3, 19.5)	21.8 (21.2, 22.4)	23.7 (23.0, 24.4)
urban-rural difference	0.2 (-0.3, 0.7)	0.2 (-0.4, 0.8)	0.2 (-0.3, 0.7)	0.2 (-0.3, 0.7)	0.1 (-0.4, 0.7)	-0.2 (-0.6, 0.1)	-0.2 (-0.6, 0.3)	-0.2 (-0.6, 0.2)	-0.2 (-0.6, 0.2)	-0.3 (-0.8, 0.2)
Slovakia										
rural	18.7 (17.9, 19.6)	15.5 (14.6, 16.5)	17.5 (16.6, 18.4)	20.4 (19.5, 21.3)	22.6 (21.6, 23.6)	19.7 (19.1, 20.3)	16.5 (15.8, 17.2)	18.5 (17.8, 19.1)	21.3 (20.7, 22.0)	23.6 (22.7, 24.4)
urban	18.9 (18.1, 19.8)	15.7 (14.8, 16.7)	17.7 (16.9, 18.6)	20.5 (19.7, 21.4)	22.8 (21.8, 23.7)	19.5 (18.8, 20.1)	16.3 (15.6, 17.0)	18.2 (17.6, 18.9)	21.1 (20.4, 21.8)	23.3 (22.5, 24.1)
urban-rural difference	0.2 (-0.3, 0.7)	0.2 (-0.3, 0.7)	0.2 (-0.3, 0.7)	0.2 (-0.3, 0.7)	0.2 (-0.4, 0.7)	-0.2 (-0.7, 0.2)	-0.2 (-0.7, 0.3)	-0.2 (-0.6, 0.2)	-0.2 (-0.7, 0.2)	-0.3 (-0.8, 0.3)
Slovenia										

Boys	Mean BMI in 1990 (kg/m^2)					Mean BMI in 2020 (kg/m^2)				
	Age-standardised	5 years	10 years	15 years	19 years	Age-standardised	5 years	10 years	15 years	19 years
rural	18.9 (18.5, 19.2)	15.0 (14.6, 15.4)	17.7 (17.4, 18.0)	20.8 (20.4, 21.1)	22.5 (22.1, 22.8)	20.0 (19.5, 20.4)	16.1 (15.6, 16.6)	18.8 (18.4, 19.3)	21.9 (21.4, 22.3)	23.6 (23.1, 24.1)
urban	19.2 (18.9, 19.5)	15.4 (15.1, 15.8)	18.1 (17.8, 18.4)	21.0 (20.7, 21.4)	22.6 (22.3, 23.0)	19.8 (19.4, 20.3)	16.1 (15.6, 16.6)	18.7 (18.3, 19.2)	21.7 (21.2, 22.1)	23.3 (22.8, 23.8)
urban-rural difference	0.3 (-0.1, 0.8)	0.5 (-0.1, 1.0)	0.4 (-0.1, 0.8)	0.2 (-0.2, 0.7)	0.2 (-0.4, 0.7)	-0.1 (-0.6, 0.3)	0.0 (-0.5, 0.6)	-0.1 (-0.5, 0.3)	-0.2 (-0.6, 0.2)	-0.3 (-0.8, 0.3)
Eastern Europe										
Belarus										
rural	18.7 (16.8, 20.7)	15.8 (12.7, 18.9)	17.5 (15.5, 19.5)	20.3 (18.4, 22.1)	22.5 (20.5, 24.5)	19.5 (17.9, 21.1)	16.5 (13.6, 19.5)	18.2 (16.5, 20.0)	21.0 (19.4, 22.6)	23.2 (21.6, 24.9)
urban	18.8 (16.9, 20.7)	15.9 (12.8, 19.0)	17.6 (15.6, 19.5)	20.3 (18.5, 22.2)	22.6 (20.5, 24.7)	19.5 (17.9, 21.2)	16.6 (13.7, 19.5)	18.3 (16.6, 20.0)	21.1 (19.5, 22.6)	23.3 (21.7, 24.9)
urban-rural difference	0.1 (-0.7, 0.8)	0.1 (-0.9, 1.0)	0.1 (-0.7, 0.8)	0.1 (-0.7, 0.7)	0.1 (-0.7, 0.8)	0.1 (-0.6, 0.7)	0.1 (-0.8, 1.0)	0.1 (-0.6, 0.8)	0.1 (-0.6, 0.7)	0.1 (-0.7, 0.8)
Estonia										
rural	19.0 (17.6, 20.3)	15.5 (14.1, 16.9)	18.0 (16.6, 19.3)	20.7 (19.3, 22.1)	22.2 (20.9, 23.5)	19.8 (19.2, 20.4)	16.3 (15.6, 17.0)	18.7 (18.2, 19.3)	21.5 (20.8, 22.2)	23.0 (22.1, 23.8)
urban	18.9 (17.5, 20.1)	15.2 (13.8, 16.6)	17.8 (16.4, 19.1)	20.7 (19.3, 22.0)	22.2 (20.9, 23.6)	19.7 (19.1, 20.3)	16.1 (15.4, 16.7)	18.6 (18.0, 19.2)	21.5 (20.8, 22.1)	23.1 (22.2, 23.9)
urban-rural difference	-0.1 (-0.7, 0.4)	-0.3 (-1.0, 0.4)	-0.2 (-0.8, 0.4)	-0.1 (-0.6, 0.5)	0.0 (-0.6, 0.6)	-0.1 (-0.4, 0.3)	-0.2 (-0.7, 0.3)	-0.1 (-0.5, 0.2)	0.0 (-0.4, 0.4)	0.1 (-0.4, 0.5)
Latvia										
rural	18.4 (16.9, 19.8)	15.5 (14.1, 17.0)	17.2 (15.8, 18.6)	19.9 (18.4, 21.4)	22.0 (19.8, 24.2)	19.1 (18.4, 19.8)	16.2 (15.5, 16.9)	17.9 (17.3, 18.5)	20.6 (19.7, 21.5)	22.7 (20.9, 24.5)
urban	18.5 (17.1, 20.0)	15.7 (14.2, 17.2)	17.3 (15.9, 18.7)	20.1 (18.5, 21.6)	22.1 (20.0, 24.3)	19.2 (18.5, 19.9)	16.4 (15.6, 17.1)	18.0 (17.4, 18.6)	20.7 (19.9, 21.6)	22.8 (21.1, 24.6)
urban-rural difference	0.1 (-0.4, 0.7)	0.1 (-0.5, 0.8)	0.1 (-0.4, 0.7)	0.1 (-0.5, 0.8)	0.1 (-0.6, 0.9)	0.1 (-0.3, 0.5)	0.1 (-0.3, 0.6)	0.1 (-0.3, 0.5)	0.1 (-0.4, 0.6)	0.1 (-0.6, 0.8)
Lithuania										
rural	18.5 (17.1, 20.0)	15.7 (14.1, 17.3)	17.3 (15.8, 18.7)	20.0 (18.6, 21.6)	22.2 (20.2, 24.3)	19.1 (18.4, 19.8)	16.3 (15.5, 17.1)	17.8 (17.2, 18.5)	20.6 (19.8, 21.4)	22.8 (21.1, 24.4)
urban	18.6 (17.2, 20.0)	15.7 (14.2, 17.3)	17.3 (15.9, 18.8)	20.1 (18.6, 21.6)	22.3 (20.2, 24.2)	19.1 (18.4, 19.8)	16.3 (15.5, 17.0)	17.9 (17.2, 18.5)	20.6 (19.8, 21.5)	22.8 (21.2, 24.4)
urban-rural difference	0.0 (-0.5, 0.6)	0.0 (-0.6, 0.7)	0.0 (-0.5, 0.6)	0.1 (-0.6, 0.7)	0.1 (-0.6, 0.8)	0.0 (-0.4, 0.4)	0.0 (-0.4, 0.4)	0.0 (-0.3, 0.4)	0.0 (-0.4, 0.5)	0.1 (-0.6, 0.7)
Moldova										
rural	18.1 (16.5, 19.6)	15.1 (13.3, 17.0)	16.8 (15.2, 18.3)	19.6 (18.0, 21.2)	21.8 (20.1, 23.5)	18.7 (17.6, 19.7)	15.7 (14.3, 17.1)	17.4 (16.3, 18.5)	20.2 (19.0, 21.6)	22.4 (21.1, 23.8)
urban	18.2 (16.7, 19.7)	15.3 (13.6, 17.1)	17.0 (15.4, 18.5)	19.8 (18.2, 21.4)	22.0 (20.3, 23.7)	18.9 (17.8, 20.0)	16.0 (14.5, 17.4)	17.6 (16.5, 18.8)	20.5 (19.2, 21.7)	22.7 (21.4, 24.0)
urban-rural difference	0.2 (-0.5, 0.8)	0.2 (-0.6, 1.0)	0.2 (-0.5, 0.8)	0.2 (-0.5, 0.8)	0.2 (-0.6, 0.9)	0.2 (-0.3, 0.7)	0.2 (-0.5, 0.9)	0.2 (-0.3, 0.7)	0.2 (-0.3, 0.8)	0.2 (-0.5, 0.9)
Russian Federation										
rural	19.2 (18.6, 19.8)	17.1 (16.5, 17.7)	17.9 (17.4, 18.5)	20.5 (19.9, 21.1)	22.6 (22.0, 23.2)	19.2 (18.3, 20.0)	17.0 (16.2, 17.9)	17.9 (17.1, 18.7)	20.5 (19.7, 21.3)	22.5 (21.7, 23.4)
urban	19.0 (18.4, 19.6)	16.7 (16.1, 17.3)	17.7 (17.1, 18.3)	20.4 (19.8, 21.0)	22.5 (21.9, 23.1)	19.1 (18.4, 19.8)	16.8 (16.1, 17.5)	17.8 (17.1, 18.5)	20.5 (19.8, 21.2)	22.6 (21.9, 23.4)
urban-rural difference	-0.2 (-0.5, 0.1)	-0.4 (-0.7, -0.1)	-0.3 (-0.6, 0.0)	-0.1 (-0.4, 0.1)	-0.1 (-0.4, 0.2)	-0.1 (-0.5, 0.4)	-0.2 (-0.7, 0.3)	-0.1 (-0.6, 0.4)	0.0 (-0.5, 0.5)	0.1 (-0.4, 0.6)
Ukraine										
rural	18.2 (16.8, 19.6)	15.3 (13.9, 16.8)	17.0 (15.6, 18.5)	19.6 (18.2, 21.1)	22.0 (20.5, 23.7)	18.7 (18.3, 19.2)	15.9 (15.3, 16.5)	17.6 (17.1, 18.1)	20.1 (19.6, 20.6)	22.6 (21.8, 23.4)
urban	18.3 (16.9, 19.7)	15.6 (14.2, 17.1)	17.2 (15.8, 18.6)	19.6 (18.2, 21.1)	22.0 (20.5, 23.6)	18.9 (18.4, 19.4)	16.2 (15.6, 16.8)	17.8 (17.3, 18.3)	20.2 (19.7, 20.7)	22.6 (21.8, 23.4)
urban-rural difference	0.1 (-0.5, 0.8)	0.3 (-0.4, 1.0)	0.2 (-0.5, 0.8)	0.0 (-0.6, 0.7)	0.0 (-0.7, 0.6)	0.1 (-0.2, 0.5)	0.3 (-0.2, 0.8)	0.2 (-0.2, 0.6)	0.1 (-0.3, 0.5)	0.0 (-0.5, 0.4)
**Central Asia, Middle East and north Africa**										
Central Asia										
Armenia										
rural	17.3 (15.8, 18.8)	14.4 (12.4, 16.5)	16.1 (14.6, 17.7)	19.1 (17.5, 20.7)	19.8 (18.2, 21.4)	18.9 (18.2, 19.7)	16.1 (14.8, 17.3)	17.8 (17.0, 18.6)	20.7 (19.7, 21.9)	21.5 (20.3, 22.7)
urban	18.0 (16.5, 19.5)	15.0 (13.1, 17.1)	16.8 (15.3, 18.4)	19.8 (18.2, 21.4)	20.5 (18.9, 22.2)	19.6 (18.8, 20.3)	16.7 (15.5, 17.9)	18.4 (17.6, 19.2)	21.4 (20.4, 22.5)	22.1 (20.9, 23.3)
urban-rural difference	0.7 (-0.1, 1.4)	0.6 (-0.3, 1.6)	0.7 (-0.1, 1.4)	0.7 (0.0, 1.4)	0.7 (0.0, 1.5)	0.6 (0.1, 1.2)	0.6 (-0.1, 1.3)	0.6 (0.1, 1.2)	0.7 (0.1, 1.2)	0.7 (0.0, 1.4)
Azerbaijan										
rural	17.8 (16.1, 19.5)	14.6 (11.7, 17.6)	16.4 (14.7, 18.2)	19.6 (17.9, 21.1)	21.6 (19.9, 23.2)	19.4 (18.0, 20.8)	16.3 (13.4, 19.0)	18.0 (16.5, 19.5)	21.2 (19.9, 22.5)	23.2 (21.9, 24.5)
urban	18.1 (16.4, 19.8)	15.1 (12.1, 18.0)	16.8 (15.0, 18.6)	19.8 (18.2, 21.4)	21.8 (20.1, 23.4)	19.6 (18.3, 21.0)	16.6 (13.7, 19.3)	18.3 (16.8, 19.7)	21.4 (20.1, 22.6)	23.3 (22.0, 24.6)
urban-rural difference	0.3 (-0.4, 1.1)	0.4 (-0.6, 1.5)	0.4 (-0.4, 1.2)	0.3 (-0.4, 1.0)	0.2 (-0.5, 1.0)	0.2 (-0.4, 0.8)	0.3 (-0.6, 1.2)	0.3 (-0.4, 0.9)	0.2 (-0.4, 0.8)	0.1 (-0.6, 0.8)
Georgia										
rural	17.5 (16.0, 19.1)	14.7 (12.8, 16.6)	16.1 (14.6, 17.7)	19.2 (17.6, 20.9)	20.9 (19.2, 22.7)	19.2 (18.5, 20.0)	16.4 (15.4, 17.4)	17.9 (17.1, 18.7)	21.0 (19.9, 22.0)	22.7 (21.4, 24.0)
urban	17.9 (16.4, 19.4)	15.1 (13.2, 17.0)	16.5 (15.0, 18.1)	19.5 (17.9, 21.2)	21.2 (19.5, 22.9)	19.5 (18.8, 20.3)	16.8 (15.8, 17.8)	18.2 (17.4, 19.0)	21.2 (20.1, 22.3)	22.9 (21.6, 24.1)
urban-rural difference	0.4 (-0.4, 1.1)	0.5 (-0.4, 1.4)	0.4 (-0.4, 1.2)	0.3 (-0.4, 1.0)	0.3 (-0.6, 1.1)	0.3 (-0.2, 0.7)	0.4 (-0.2, 1.0)	0.3 (-0.1, 0.8)	0.3 (-0.3, 0.8)	0.2 (-0.5, 0.9)
Kazakhstan										
rural	17.0 (15.4, 18.6)	13.9 (12.2, 15.6)	15.6 (14.1, 17.2)	18.6 (17.0, 20.2)	21.1 (19.3, 22.9)	18.6 (18.1, 19.1)	15.5 (14.7, 16.2)	17.2 (16.7, 17.7)	20.2 (19.6, 20.8)	22.6 (21.5, 23.7)
urban	17.6 (16.0, 19.2)	14.6 (13.0, 16.3)	16.3 (14.7, 17.9)	19.1 (17.6, 20.8)	21.5 (19.7, 23.4)	19.1 (18.6, 19.7)	16.2 (15.4, 16.9)	17.8 (17.3, 18.3)	20.7 (20.1, 21.3)	23.1 (21.9, 24.3)
urban-rural difference	0.6 (-0.1, 1.3)	0.7 (-0.2, 1.6)	0.6 (-0.1, 1.4)	0.5 (-0.2, 1.3)	0.5 (-0.3, 1.3)	0.6 (0.2, 1.0)	0.7 (0.2, 1.2)	0.6 (0.2, 1.0)	0.5 (0.0, 1.0)	0.5 (-0.2, 1.1)
Kyrgyzstan										

Boys	Mean BMI in 1990 (kg/m^2)					Mean BMI in 2020 (kg/m^2)				
	Age-standardised	5 years	10 years	15 years	19 years	Age-standardised	5 years	10 years	15 years	19 years
rural	16.7 (15.1, 18.4)	14.2 (12.5, 15.9)	15.4 (13.8, 17.0)	18.3 (16.6, 20.0)	20.2 (17.9, 22.4)	18.3 (17.3, 19.3)	15.7 (14.7, 16.7)	16.9 (16.0, 17.8)	19.9 (18.7, 21.0)	21.7 (19.8, 23.6)
urban	17.1 (15.6, 18.8)	14.5 (12.8, 16.2)	15.7 (14.2, 17.4)	18.7 (17.1, 20.5)	20.6 (18.3, 22.9)	18.6 (17.6, 19.5)	16.0 (14.9, 17.0)	17.2 (16.3, 18.1)	20.2 (19.0, 21.4)	22.1 (20.2, 24.0)
urban-rural difference	0.4 (-0.3, 1.1)	0.3 (-0.5, 1.3)	0.4 (-0.3, 1.1)	0.4 (-0.3, 1.2)	0.4 (-0.4, 1.3)	0.3 (-0.2, 0.8)	0.3 (-0.4, 0.9)	0.3 (-0.2, 0.8)	0.3 (-0.3, 0.9)	0.4 (-0.4, 1.1)
Mongolia										
rural	16.6 (15.0, 18.2)	13.6 (10.6, 16.6)	15.3 (13.7, 17.0)	18.3 (16.9, 19.9)	20.1 (18.5, 21.6)	18.2 (17.2, 19.1)	15.1 (12.5, 17.8)	16.9 (15.9, 17.9)	19.9 (19.1, 20.6)	21.6 (20.8, 22.4)
urban	16.8 (15.2, 18.4)	13.9 (10.9, 16.9)	15.5 (13.9, 17.2)	18.5 (17.0, 20.0)	20.2 (18.7, 21.8)	18.3 (17.3, 19.2)	15.3 (12.6, 17.8)	17.0 (16.0, 18.0)	19.9 (19.2, 20.6)	21.6 (20.8, 22.4)
urban-rural difference	0.2 (-0.5, 0.9)	0.2 (-0.8, 1.3)	0.2 (-0.6, 1.0)	0.2 (-0.5, 0.8)	0.1 (-0.5, 0.8)	0.1 (-0.4, 0.6)	0.2 (-0.7, 1.0)	0.1 (-0.5, 0.7)	0.1 (-0.4, 0.5)	0.0 (-0.5, 0.6)
Tajikistan										
rural	16.7 (15.1, 18.3)	13.5 (11.6, 15.4)	15.3 (13.6, 16.9)	18.5 (16.8, 20.2)	20.8 (19.0, 22.6)	18.1 (17.4, 18.8)	14.9 (13.8, 16.0)	16.6 (15.8, 17.4)	19.9 (18.8, 20.9)	22.2 (21.1, 23.3)
urban	16.9 (15.3, 18.6)	13.7 (11.8, 15.6)	15.5 (13.8, 17.1)	18.7 (17.0, 20.5)	21.1 (19.3, 22.9)	18.2 (17.4, 18.9)	14.9 (13.8, 16.0)	16.7 (15.9, 17.5)	20.0 (18.9, 21.1)	22.4 (21.2, 23.5)
urban-rural difference	0.2 (-0.5, 1.0)	0.1 (-0.7, 1.1)	0.2 (-0.5, 1.0)	0.3 (-0.4, 1.0)	0.3 (-0.5, 1.2)	0.1 (-0.4, 0.6)	0.0 (-0.6, 0.6)	0.1 (-0.4, 0.5)	0.1 (-0.4, 0.7)	0.2 (-0.6, 0.9)
Turkmenistan										
rural	17.1 (15.5, 18.7)	13.5 (11.6, 15.5)	15.7 (14.1, 17.4)	19.1 (17.4, 20.8)	21.1 (19.3, 22.8)	18.9 (18.0, 19.7)	15.3 (13.9, 16.6)	17.5 (16.6, 18.4)	20.9 (19.7, 22.0)	22.8 (21.7, 23.9)
urban	17.5 (15.9, 19.1)	13.9 (11.9, 15.8)	16.1 (14.5, 17.7)	19.5 (17.8, 21.2)	21.4 (19.7, 23.1)	19.2 (18.3, 20.0)	15.5 (14.2, 16.9)	17.8 (16.9, 18.7)	21.1 (20.0, 22.2)	23.1 (22.0, 24.2)
urban-rural difference	0.4 (-0.4, 1.1)	0.4 (-0.5, 1.3)	0.4 (-0.4, 1.2)	0.4 (-0.4, 1.1)	0.4 (-0.4, 1.2)	0.3 (-0.2, 0.8)	0.3 (-0.4, 1.0)	0.3 (-0.2, 0.8)	0.3 (-0.3, 0.9)	0.3 (-0.5, 1.0)
Uzbekistan										
rural	18.0 (16.3, 19.6)	14.7 (11.6, 17.7)	16.8 (15.0, 18.4)	19.9 (18.4, 21.4)	20.9 (19.3, 22.4)	20.4 (19.3, 21.5)	17.1 (14.3, 19.8)	19.2 (18.0, 20.4)	22.3 (21.4, 23.2)	23.3 (22.5, 24.2)
urban	18.1 (16.4, 19.8)	14.9 (11.8, 18.0)	16.9 (15.2, 18.6)	20.0 (18.5, 21.5)	20.9 (19.4, 22.5)	20.4 (19.2, 21.6)	17.2 (14.5, 20.1)	19.2 (17.9, 20.5)	22.3 (21.3, 23.3)	23.2 (22.2, 24.2)
urban-rural difference	0.1 (-0.6, 0.9)	0.3 (-0.8, 1.3)	0.2 (-0.6, 1.0)	0.1 (-0.6, 0.8)	0.0 (-0.7, 0.7)	0.0 (-0.6, 0.6)	0.2 (-0.8, 1.1)	0.1 (-0.7, 0.7)	0.0 (-0.7, 0.5)	-0.1 (-0.8, 0.5)
Middle East and north Africa										
Algeria										
rural	17.3 (15.5, 19.0)	14.1 (11.1, 17.1)	16.0 (14.1, 17.8)	19.1 (17.5, 20.8)	20.4 (18.8, 22.1)	18.7 (17.4, 20.1)	15.6 (12.8, 18.3)	17.5 (16.0, 19.0)	20.6 (19.4, 21.9)	21.9 (20.8, 23.1)
urban	17.6 (15.8, 19.3)	14.2 (11.2, 17.2)	16.2 (14.4, 18.1)	19.6 (17.9, 21.2)	21.0 (19.3, 22.7)	19.3 (18.0, 20.6)	15.9 (13.2, 18.6)	18.0 (16.5, 19.4)	21.3 (20.0, 22.6)	22.7 (21.6, 23.8)
urban-rural difference	0.3 (-0.4, 1.0)	0.1 (-0.9, 1.0)	0.3 (-0.5, 1.0)	0.4 (-0.3, 1.1)	0.6 (-0.2, 1.3)	0.6 (-0.1, 1.2)	0.3 (-0.6, 1.2)	0.5 (-0.2, 1.2)	0.7 (0.1, 1.3)	0.8 (0.1, 1.5)
Bahrain										
rural	18.4 (16.9, 19.8)	14.8 (13.0, 16.6)	17.0 (15.5, 18.5)	20.4 (19.0, 21.8)	22.2 (20.4, 24.0)	20.3 (19.2, 21.4)	16.7 (15.1, 18.3)	18.9 (17.8, 20.0)	22.3 (21.2, 23.4)	24.1 (22.5, 25.6)
urban	18.7 (17.4, 20.1)	14.9 (13.2, 16.6)	17.3 (15.9, 18.7)	20.9 (19.5, 22.2)	22.8 (21.1, 24.5)	20.9 (20.0, 21.8)	17.1 (15.7, 18.5)	19.5 (18.5, 20.4)	23.0 (22.1, 24.0)	25.0 (23.6, 26.3)
urban-rural difference	0.3 (-0.4, 1.0)	0.1 (-0.9, 1.0)	0.3 (-0.5, 1.0)	0.4 (-0.2, 1.1)	0.6 (-0.2, 1.4)	0.6 (0.0, 1.3)	0.4 (-0.5, 1.3)	0.5 (-0.2, 1.3)	0.7 (0.1, 1.4)	0.9 (0.1, 1.6)
Egypt										
rural	18.9 (17.5, 20.4)	16.5 (14.8, 18.2)	17.3 (15.9, 18.8)	20.6 (19.2, 22.1)	22.6 (21.2, 24.1)	19.9 (19.0, 20.7)	17.4 (16.2, 18.7)	18.3 (17.5, 19.2)	21.6 (20.8, 22.4)	23.6 (22.8, 24.5)
urban	19.1 (17.7, 20.6)	16.6 (14.9, 18.3)	17.5 (16.1, 19.0)	20.9 (19.5, 22.3)	22.9 (21.5, 24.4)	20.3 (19.4, 21.2)	17.8 (16.5, 19.2)	18.7 (17.8, 19.6)	22.1 (21.2, 22.9)	24.1 (23.2, 25.0)
urban-rural difference	0.2 (-0.4, 0.8)	0.1 (-0.6, 0.9)	0.2 (-0.4, 0.8)	0.2 (-0.3, 0.9)	0.3 (-0.4, 0.9)	0.4 (-0.1, 0.9)	0.4 (-0.4, 1.1)	0.4 (-0.1, 0.9)	0.5 (0.0, 0.9)	0.5 (0.0, 1.0)
Iran										
rural	16.8 (16.2, 17.4)	14.3 (13.7, 14.9)	15.6 (15.0, 16.2)	18.4 (17.8, 19.1)	20.1 (19.5, 20.8)	18.8 (18.1, 19.4)	16.2 (15.6, 16.9)	17.5 (16.9, 18.2)	20.4 (19.7, 21.0)	22.0 (21.4, 22.7)
urban	17.4 (16.8, 18.0)	14.4 (13.8, 15.0)	16.0 (15.4, 16.6)	19.2 (18.6, 19.8)	21.2 (20.5, 21.8)	19.8 (19.1, 20.4)	16.7 (16.1, 17.4)	18.4 (17.7, 19.0)	21.6 (20.9, 22.2)	23.5 (22.9, 24.2)
urban-rural difference	0.6 (0.2, 0.9)	0.1 (-0.3, 0.5)	0.4 (0.1, 0.8)	0.8 (0.4, 1.1)	1.1 (0.7, 1.4)	1.0 (0.7, 1.3)	0.5 (0.2, 0.9)	0.9 (0.6, 1.2)	1.2 (0.9, 1.5)	1.5 (1.2, 1.8)
Iraq										
rural	18.2 (16.5, 20.0)	15.0 (12.1, 18.1)	16.9 (15.1, 18.7)	20.1 (18.4, 21.8)	21.6 (19.7, 23.4)	19.9 (18.5, 21.3)	16.7 (14.0, 19.5)	18.6 (17.1, 20.1)	21.8 (20.5, 23.2)	23.2 (21.8, 24.8)
urban	18.6 (16.8, 20.3)	15.1 (12.3, 18.2)	17.2 (15.4, 19.0)	20.6 (19.0, 22.2)	22.2 (20.4, 24.0)	20.5 (19.2, 21.9)	17.1 (14.3, 19.8)	19.2 (17.7, 20.6)	22.5 (21.3, 23.8)	24.1 (22.8, 25.6)
urban-rural difference	0.4 (-0.3, 1.1)	0.1 (-0.8, 1.1)	0.3 (-0.5, 1.0)	0.5 (-0.2, 1.2)	0.6 (-0.2, 1.4)	0.6 (0.0, 1.3)	0.4 (-0.5, 1.3)	0.6 (-0.1, 1.3)	0.7 (0.1, 1.4)	0.9 (0.1, 1.6)
Jordan										
rural	17.8 (16.4, 19.2)	14.0 (12.3, 15.6)	16.5 (15.1, 18.0)	19.8 (18.4, 21.3)	21.5 (20.0, 23.1)	19.3 (18.4, 20.1)	15.5 (14.3, 16.7)	18.0 (17.1, 18.9)	21.3 (20.4, 22.2)	23.0 (22.0, 24.0)
urban	17.8 (16.4, 19.3)	13.6 (12.0, 15.2)	16.5 (15.0, 17.9)	20.0 (18.6, 21.5)	22.0 (20.4, 23.6)	19.6 (18.8, 20.3)	15.3 (14.3, 16.4)	18.2 (17.4, 19.0)	21.8 (21.0, 22.6)	23.7 (22.8, 24.6)
urban-rural difference	0.0 (-0.6, 0.7)	-0.4 (-1.2, 0.4)	-0.1 (-0.7, 0.6)	0.2 (-0.4, 0.9)	0.5 (-0.2, 1.2)	0.3 (-0.2, 0.8)	-0.1 (-0.9, 0.5)	0.2 (-0.4, 0.7)	0.5 (-0.1, 1.0)	0.7 (0.1, 1.3)
Kuwait										
rural	19.7 (18.4, 21.1)	14.6 (13.1, 16.1)	18.4 (17.0, 19.8)	22.6 (21.3, 24.0)	22.8 (21.4, 24.2)	21.2 (20.1, 22.4)	16.1 (14.8, 17.5)	19.9 (18.8, 21.1)	24.2 (23.0, 25.3)	24.4 (23.2, 25.6)
urban	20.1 (18.9, 21.3)	14.6 (13.4, 15.8)	18.7 (17.5, 19.9)	23.2 (22.0, 24.4)	23.7 (22.4, 24.9)	21.9 (20.9, 22.9)	16.3 (15.4, 17.3)	20.5 (19.5, 21.4)	25.0 (24.0, 26.0)	25.4 (24.4, 26.4)
urban-rural difference	0.4 (-0.3, 1.1)	0.0 (-0.9, 0.9)	0.3 (-0.4, 1.0)	0.6 (-0.1, 1.3)	0.8 (0.1, 1.6)	0.6 (0.0, 1.3)	0.2 (-0.7, 1.1)	0.5 (-0.2, 1.2)	0.8 (0.2, 1.5)	1.1 (0.3, 1.8)
Lebanon										
rural	18.6 (17.4, 19.7)	14.7 (13.4, 15.9)	17.1 (15.9, 18.2)	20.6 (19.4, 21.8)	22.9 (21.5, 24.2)	20.5 (19.3, 21.8)	16.7 (15.4, 18.0)	19.1 (17.9, 20.4)	22.6 (21.4, 23.9)	24.8 (23.5, 26.3)
urban	19.0 (17.8, 20.1)	14.8 (13.5, 16.0)	17.4 (16.2, 18.6)	21.2 (20.0, 22.4)	23.6 (22.3, 25.0)	21.2 (20.1, 22.5)	17.0 (15.8, 18.3)	19.7 (18.5, 20.9)	23.4 (22.3, 24.7)	25.9 (24.5, 27.3)

Boys	Mean BMI in 1990 (kg/m^2)					Mean BMI in 2020 (kg/m^2)				
	Age-standardised	5 years	10 years	15 years	19 years	Age-standardised	5 years	10 years	15 years	19 years
urban-rural difference	0.4 (-0.1, 1.0)	0.1 (-0.6, 0.7)	0.3 (-0.2, 0.9)	0.6 (0.0, 1.1)	0.8 (0.1, 1.4)	0.7 (0.1, 1.2)	0.3 (-0.3, 1.0)	0.6 (0.0, 1.1)	0.8 (0.3, 1.4)	1.0 (0.3, 1.7)
Libya										
rural	18.0 (15.8, 20.2)	14.9 (11.7, 18.1)	16.7 (14.4, 18.9)	19.8 (17.7, 22.0)	21.5 (18.9, 24.0)	19.6 (17.4, 21.8)	16.5 (13.2, 19.7)	18.3 (16.1, 20.4)	21.5 (19.3, 23.5)	23.1 (20.6, 25.6)
urban	18.3 (16.1, 20.6)	15.0 (11.7, 18.2)	16.9 (14.7, 19.2)	20.3 (18.1, 22.4)	22.0 (19.5, 24.6)	20.2 (18.1, 22.4)	16.9 (13.6, 20.1)	18.8 (16.6, 21.0)	22.2 (20.1, 24.2)	23.9 (21.4, 26.4)
urban-rural difference	0.3 (-0.4, 1.0)	0.1 (-0.9, 1.0)	0.3 (-0.5, 1.0)	0.4 (-0.3, 1.1)	0.6 (-0.2, 1.4)	0.6 (-0.1, 1.3)	0.3 (-0.6, 1.3)	0.5 (-0.2, 1.2)	0.7 (0.0, 1.4)	0.8 (0.1, 1.6)
Morocco										
rural	16.5 (15.0, 18.1)	14.1 (12.4, 15.9)	15.4 (13.8, 17.0)	18.2 (16.7, 19.8)	18.9 (17.2, 20.6)	17.9 (17.3, 18.5)	15.5 (14.6, 16.3)	16.8 (16.2, 17.4)	19.6 (18.9, 20.3)	20.3 (19.3, 21.3)
urban	16.8 (15.3, 18.4)	14.1 (12.5, 15.8)	15.6 (14.0, 17.2)	18.6 (17.0, 20.2)	19.4 (17.7, 21.0)	18.4 (17.8, 19.0)	15.7 (14.9, 16.5)	17.2 (16.6, 17.8)	20.2 (19.5, 20.9)	21.0 (20.0, 22.0)
urban-rural difference	0.3 (-0.4, 1.0)	0.0 (-0.8, 0.9)	0.2 (-0.5, 0.9)	0.4 (-0.3, 1.1)	0.5 (-0.3, 1.2)	0.5 (0.0, 1.0)	0.3 (-0.4, 0.9)	0.4 (-0.1, 0.9)	0.6 (0.1, 1.1)	0.7 (0.1, 1.3)
Occupied Palestinian Territory										
rural	17.8 (16.3, 19.5)	14.7 (11.8, 17.7)	16.3 (14.7, 18.0)	19.7 (18.2, 21.2)	22.1 (20.5, 23.7)	19.4 (18.0, 20.8)	16.3 (13.5, 19.1)	17.9 (16.5, 19.3)	21.3 (20.0, 22.5)	23.7 (22.1, 25.1)
urban	18.2 (16.6, 19.8)	14.7 (11.9, 17.7)	16.6 (15.0, 18.2)	20.1 (18.7, 21.6)	22.7 (21.1, 24.3)	20.0 (18.7, 21.3)	16.6 (13.9, 19.3)	18.4 (17.1, 19.7)	22.0 (20.8, 23.2)	24.5 (23.1, 25.9)
urban-rural difference	0.3 (-0.4, 1.0)	0.0 (-0.9, 1.0)	0.3 (-0.5, 1.0)	0.5 (-0.2, 1.1)	0.6 (-0.1, 1.4)	0.6 (-0.1, 1.3)	0.3 (-0.6, 1.2)	0.5 (-0.2, 1.2)	0.7 (0.1, 1.4)	0.9 (0.2, 1.6)
Oman										
rural	18.2 (16.5, 19.8)	15.2 (12.3, 18.1)	16.9 (15.2, 18.5)	19.9 (18.5, 21.4)	21.6 (19.9, 23.3)	19.9 (18.7, 21.1)	17.0 (14.2, 19.7)	18.6 (17.4, 19.8)	21.6 (20.6, 22.7)	23.3 (21.9, 24.7)
urban	18.5 (16.9, 20.1)	15.3 (12.4, 18.1)	17.1 (15.5, 18.7)	20.3 (18.9, 21.8)	22.1 (20.5, 23.8)	20.5 (19.3, 21.6)	17.4 (14.7, 20.0)	19.1 (17.9, 20.3)	22.3 (21.4, 23.3)	24.2 (22.9, 25.5)
urban-rural difference	0.3 (-0.4, 1.0)	0.1 (-0.8, 1.1)	0.3 (-0.5, 1.0)	0.4 (-0.3, 1.1)	0.6 (-0.2, 1.3)	0.6 (0.0, 1.2)	0.4 (-0.5, 1.3)	0.5 (-0.2, 1.2)	0.7 (0.1, 1.3)	0.8 (0.1, 1.5)
Qatar										
rural	19.6 (17.9, 21.4)	16.5 (13.7, 19.4)	18.2 (16.5, 20.0)	21.5 (19.8, 23.1)	23.3 (21.4, 25.1)	21.4 (19.9, 22.9)	18.3 (15.5, 21.1)	20.0 (18.5, 21.5)	23.3 (21.8, 24.6)	25.0 (23.4, 26.7)
urban	20.0 (18.4, 21.7)	16.7 (13.9, 19.6)	18.6 (16.9, 20.2)	22.0 (20.4, 23.5)	23.9 (22.1, 25.6)	22.1 (20.6, 23.5)	18.8 (16.0, 21.5)	20.6 (19.2, 22.1)	24.0 (22.7, 25.3)	26.0 (24.4, 27.5)
urban-rural difference	0.4 (-0.3, 1.1)	0.2 (-0.8, 1.1)	0.3 (-0.4, 1.1)	0.5 (-0.2, 1.2)	0.7 (-0.1, 1.4)	0.7 (0.0, 1.3)	0.4 (-0.5, 1.3)	0.6 (-0.1, 1.3)	0.8 (0.2, 1.4)	0.9 (0.2, 1.6)
Saudi Arabia										
rural	18.2 (17.1, 19.2)	13.9 (12.5, 15.2)	16.8 (15.7, 17.9)	20.4 (19.4, 21.5)	22.2 (21.1, 23.3)	19.9 (18.7, 21.0)	15.6 (14.2, 17.0)	18.5 (17.3, 19.7)	22.1 (21.0, 23.2)	24.0 (22.7, 25.2)
urban	18.5 (17.5, 19.5)	13.9 (12.7, 15.0)	17.0 (16.0, 18.1)	20.9 (19.9, 21.9)	23.0 (21.9, 24.0)	20.5 (19.4, 21.6)	15.9 (14.7, 17.1)	19.0 (17.9, 20.1)	22.9 (21.8, 24.0)	24.9 (23.8, 26.1)
urban-rural difference	0.3 (-0.3, 1.0)	0.0 (-0.9, 0.9)	0.2 (-0.5, 1.0)	0.5 (-0.1, 1.1)	0.7 (0.0, 1.4)	0.6 (0.1, 1.2)	0.3 (-0.6, 1.1)	0.5 (-0.1, 1.1)	0.8 (0.2, 1.3)	1.0 (0.4, 1.6)
Syrian Arab Republic										
rural	18.0 (15.7, 20.3)	14.8 (11.6, 18.3)	16.6 (14.4, 19.0)	19.8 (17.7, 22.1)	21.5 (18.9, 24.2)	19.6 (17.5, 21.8)	16.5 (13.3, 19.9)	18.3 (16.1, 20.5)	21.5 (19.4, 23.6)	23.1 (20.6, 25.7)
urban	18.3 (16.1, 20.6)	14.9 (11.6, 18.4)	16.9 (14.7, 19.2)	20.3 (18.1, 22.5)	22.1 (19.5, 24.8)	20.2 (18.0, 22.4)	16.8 (13.6, 20.2)	18.8 (16.6, 21.0)	22.2 (20.0, 24.3)	23.9 (21.4, 26.5)
urban-rural difference	0.3 (-0.4, 1.1)	0.1 (-0.8, 1.1)	0.3 (-0.5, 1.0)	0.4 (-0.3, 1.1)	0.6 (-0.2, 1.4)	0.6 (-0.1, 1.2)	0.4 (-0.6, 1.3)	0.5 (-0.2, 1.2)	0.7 (0.0, 1.4)	0.8 (0.1, 1.6)
Tunisia										
rural	16.8 (15.7, 18.0)	14.9 (13.7, 16.1)	15.6 (14.4, 16.7)	18.2 (17.0, 19.3)	19.9 (18.7, 21.1)	18.7 (17.4, 20.0)	16.7 (15.4, 18.1)	17.4 (16.1, 18.8)	20.0 (18.7, 21.3)	21.7 (20.4, 23.1)
urban	17.2 (16.0, 18.3)	14.8 (13.7, 16.0)	15.8 (14.7, 17.0)	18.7 (17.5, 19.8)	20.6 (19.4, 21.8)	19.3 (18.0, 20.6)	16.9 (15.6, 18.3)	17.9 (16.6, 19.3)	20.8 (19.5, 22.1)	22.7 (21.3, 24.0)
urban-rural difference	0.3 (-0.2, 0.9)	0.0 (-0.6, 0.6)	0.2 (-0.3, 0.8)	0.5 (0.0, 1.0)	0.7 (0.2, 1.3)	0.6 (0.0, 1.1)	0.2 (-0.5, 0.9)	0.5 (-0.1, 1.0)	0.7 (0.2, 1.3)	1.0 (0.4, 1.5)
Turkey										
rural	17.5 (16.1, 18.8)	14.2 (12.7, 15.8)	16.1 (14.7, 17.6)	19.4 (18.0, 20.7)	21.0 (19.5, 22.4)	19.0 (18.3, 19.8)	15.8 (14.9, 16.7)	17.7 (16.9, 18.6)	20.9 (20.0, 21.8)	22.5 (21.5, 23.5)
urban	17.8 (16.4, 19.2)	14.5 (13.0, 16.1)	16.5 (15.1, 17.9)	19.7 (18.3, 21.1)	21.3 (19.9, 22.7)	19.7 (18.9, 20.4)	16.4 (15.5, 17.3)	18.3 (17.5, 19.2)	21.6 (20.7, 22.4)	23.2 (22.3, 24.1)
urban-rural difference	0.3 (-0.2, 0.9)	0.3 (-0.4, 1.0)	0.3 (-0.3, 0.9)	0.4 (-0.2, 0.9)	0.4 (-0.3, 1.0)	0.6 (0.2, 1.1)	0.6 (0.0, 1.2)	0.6 (0.2, 1.0)	0.6 (0.2, 1.1)	0.7 (0.1, 1.2)
United Arab Emirates										
rural	19.1 (17.5, 20.6)	16.2 (13.5, 19.0)	17.7 (16.1, 19.3)	20.8 (19.3, 22.3)	22.5 (20.7, 24.2)	20.9 (19.8, 22.0)	18.1 (15.5, 20.6)	19.6 (18.5, 20.7)	22.7 (21.7, 23.7)	24.3 (23.0, 25.6)
urban	19.6 (18.1, 21.1)	16.4 (13.7, 19.2)	18.2 (16.7, 19.7)	21.5 (20.1, 22.9)	23.3 (21.7, 25.1)	21.8 (20.8, 22.8)	18.6 (16.1, 21.1)	20.3 (19.3, 21.3)	23.7 (22.8, 24.5)	25.5 (24.3, 26.8)
urban-rural difference	0.5 (-0.2, 1.2)	0.2 (-0.7, 1.1)	0.4 (-0.3, 1.2)	0.7 (0.0, 1.4)	0.9 (0.1, 1.7)	0.8 (0.2, 1.5)	0.5 (-0.4, 1.4)	0.7 (0.1, 1.5)	1.0 (0.4, 1.6)	1.2 (0.5, 1.9)
Yemen										
rural	16.6 (15.1, 18.1)	14.7 (13.1, 16.2)	15.2 (13.8, 16.7)	18.0 (16.5, 19.5)	19.8 (18.2, 21.3)	18.2 (16.9, 19.5)	16.2 (14.9, 17.6)	16.8 (15.5, 18.1)	19.6 (18.3, 20.9)	21.3 (19.9, 22.7)
urban	17.0 (15.5, 18.4)	14.9 (13.4, 16.4)	15.6 (14.1, 17.0)	18.4 (17.0, 19.9)	20.3 (18.7, 21.8)	18.8 (17.5, 20.1)	16.7 (15.3, 18.1)	17.4 (16.1, 18.7)	20.3 (18.9, 21.6)	22.1 (20.7, 23.5)
urban-rural difference	0.4 (-0.2, 0.9)	0.2 (-0.5, 0.9)	0.3 (-0.2, 0.9)	0.4 (-0.1, 1.0)	0.5 (-0.1, 1.1)	0.6 (0.1, 1.1)	0.5 (-0.1, 1.1)	0.6 (0.1, 1.1)	0.7 (0.2, 1.2)	0.8 (0.2, 1.4)
**East and southeast Asia**										
East Asia										
China										
rural	17.5 (17.1, 17.9)	14.9 (14.5, 15.3)	16.4 (16.1, 16.8)	19.0 (18.6, 19.4)	20.2 (19.8, 20.6)	19.8 (19.0, 20.6)	17.3 (16.4, 18.1)	18.8 (18.0, 19.6)	21.3 (20.5, 22.1)	22.5 (21.7, 23.3)
urban	17.9 (17.5, 18.3)	15.3 (14.9, 15.7)	16.9 (16.5, 17.3)	19.5 (19.1, 19.9)	20.8 (20.3, 21.2)	20.3 (19.5, 21.1)	17.7 (16.9, 18.5)	19.3 (18.5, 20.0)	21.9 (21.1, 22.7)	23.1 (22.3, 23.9)
urban-rural difference	0.5 (0.2, 0.7)	0.4 (0.1, 0.7)	0.4 (0.2, 0.7)	0.5 (0.2, 0.8)	0.6 (0.3, 0.8)	0.5 (0.2, 0.8)	0.4 (0.1, 0.7)	0.5 (0.2, 0.8)	0.5 (0.2, 0.8)	0.6 (0.3, 0.9)

Boys	Mean BMI in 1990 (kg/m²)					Mean BMI in 2020 (kg/m²)				
	Age-standardised	5 years	10 years	15 years	19 years	Age-standardised	5 years	10 years	15 years	19 years
North Korea										
rural	17.4 (15.1, 19.8)	14.7 (11.2, 18.2)	16.4 (14.0, 18.8)	19.0 (16.7, 21.3)	20.4 (17.6, 23.2)	19.4 (17.0, 21.8)	16.6 (13.1, 20.3)	18.3 (16.0, 20.7)	20.9 (18.6, 23.2)	22.3 (19.5, 25.1)
urban	17.8 (15.5, 20.2)	15.0 (11.5, 18.7)	16.8 (14.4, 19.2)	19.4 (17.1, 21.7)	20.8 (17.9, 23.5)	19.8 (17.4, 22.2)	17.0 (13.5, 20.7)	18.7 (16.4, 21.1)	21.3 (19.0, 23.6)	22.7 (20.0, 25.5)
urban-rural difference	0.4 (-0.3, 1.1)	0.4 (-0.6, 1.3)	0.4 (-0.4, 1.1)	0.4 (-0.3, 1.1)	0.4 (-0.4, 1.1)	0.4 (-0.3, 1.1)	0.4 (-0.5, 1.3)	0.4 (-0.4, 1.1)	0.4 (-0.3, 1.0)	0.4 (-0.4, 1.2)
Taiwan										
rural	17.9 (16.8, 18.9)	14.5 (13.4, 15.7)	16.9 (15.9, 18.0)	19.5 (18.5, 20.6)	21.0 (19.7, 22.2)	19.5 (18.9, 20.2)	16.2 (15.4, 17.0)	18.6 (17.9, 19.3)	21.2 (20.5, 21.8)	22.6 (21.7, 23.5)
urban	18.2 (17.2, 19.2)	14.9 (13.8, 16.0)	17.3 (16.2, 18.3)	19.8 (18.8, 20.8)	21.2 (20.0, 22.4)	19.8 (19.1, 20.4)	16.5 (15.7, 17.3)	18.8 (18.2, 19.6)	21.4 (20.7, 22.0)	22.8 (21.9, 23.7)
urban-rural difference	0.3 (-0.3, 1.0)	0.4 (-0.4, 1.2)	0.3 (-0.3, 1.0)	0.3 (-0.3, 0.9)	0.2 (-0.5, 0.9)	0.2 (-0.3, 0.7)	0.3 (-0.4, 1.0)	0.3 (-0.3, 0.8)	0.2 (-0.3, 0.7)	0.1 (-0.4, 0.7)
Southeast Asia										
Brunei Darussalam										
rural	18.3 (16.9, 19.7)	14.6 (12.9, 16.3)	17.4 (16.0, 18.8)	20.3 (18.8, 21.7)	20.7 (19.0, 22.3)	20.8 (20.0, 21.6)	17.1 (15.9, 18.4)	19.9 (19.1, 20.8)	22.8 (22.0, 23.6)	23.2 (22.0, 24.4)
urban	18.4 (16.9, 19.7)	14.7 (12.9, 16.3)	17.5 (16.1, 18.9)	20.4 (18.9, 21.7)	20.8 (19.1, 22.4)	21.2 (20.5, 21.9)	17.5 (16.2, 18.7)	20.3 (19.6, 21.1)	23.1 (22.5, 23.8)	23.6 (22.4, 24.7)
urban-rural difference	0.1 (-0.6, 0.7)	0.1 (-0.7, 0.9)	0.1 (-0.6, 0.7)	0.1 (-0.6, 0.7)	0.1 (-0.6, 0.8)	0.4 (-0.2, 0.9)	0.4 (-0.4, 1.1)	0.4 (-0.2, 0.9)	0.4 (-0.2, 0.9)	0.3 (-0.3, 1.0)
Cambodia										
rural	16.7 (14.4, 19.0)	14.0 (10.7, 17.4)	15.6 (13.3, 17.9)	18.2 (16.0, 20.3)	19.6 (17.0, 22.2)	18.6 (16.3, 20.8)	16.0 (12.6, 19.3)	17.5 (15.2, 19.7)	20.1 (18.0, 22.2)	21.6 (19.0, 24.1)
urban	16.9 (14.6, 19.2)	14.3 (11.0, 17.7)	15.8 (13.6, 18.1)	18.4 (16.2, 20.6)	19.8 (17.2, 22.4)	19.1 (16.8, 21.3)	16.5 (13.2, 19.9)	18.1 (15.8, 20.2)	20.6 (18.5, 22.7)	22.0 (19.5, 24.5)
urban-rural difference	0.2 (-0.4, 0.9)	0.3 (-0.6, 1.2)	0.3 (-0.5, 1.0)	0.2 (-0.4, 0.9)	0.2 (-0.6, 0.9)	0.5 (-0.1, 1.2)	0.6 (-0.3, 1.5)	0.6 (-0.1, 1.3)	0.5 (-0.1, 1.2)	0.5 (-0.2, 1.2)
Indonesia										
rural	16.6 (15.8, 17.4)	14.3 (13.5, 15.2)	15.4 (14.6, 16.2)	17.9 (17.1, 18.8)	19.5 (18.7, 20.4)	18.0 (17.3, 18.6)	15.7 (15.1, 16.4)	16.8 (16.2, 17.4)	19.3 (18.7, 20.0)	20.9 (20.3, 21.6)
urban	16.8 (16.0, 17.6)	14.6 (13.7, 15.4)	15.6 (14.8, 16.4)	18.2 (17.3, 19.0)	19.7 (18.9, 20.6)	18.5 (17.9, 19.1)	16.3 (15.7, 17.0)	17.4 (16.7, 18.0)	19.9 (19.3, 20.5)	21.5 (20.8, 22.1)
urban-rural difference	0.2 (-0.2, 0.6)	0.2 (-0.2, 0.6)	0.2 (-0.1, 0.6)	0.2 (-0.2, 0.6)	0.2 (-0.2, 0.6)	0.6 (0.3, 0.9)	0.6 (0.2, 1.0)	0.6 (0.3, 0.9)	0.6 (0.2, 0.9)	0.6 (0.2, 0.9)
Lao PDR										
rural	16.6 (14.9, 18.3)	13.9 (10.9, 16.8)	15.5 (13.8, 17.3)	18.2 (16.6, 19.8)	19.3 (17.7, 20.9)	18.5 (17.0, 20.0)	15.8 (12.9, 18.7)	17.4 (15.8, 19.1)	20.1 (18.6, 21.5)	21.2 (19.8, 22.6)
urban	16.8 (15.2, 18.6)	14.1 (11.2, 17.1)	15.8 (14.0, 17.6)	18.4 (16.8, 20.1)	19.5 (17.9, 21.2)	19.0 (17.5, 20.6)	16.4 (13.4, 19.2)	18.0 (16.3, 19.6)	20.6 (19.1, 22.1)	21.7 (20.3, 23.1)
urban-rural difference	0.2 (-0.4, 0.9)	0.3 (-0.6, 1.2)	0.3 (-0.5, 1.0)	0.2 (-0.4, 0.9)	0.2 (-0.6, 0.9)	0.5 (-0.1, 1.2)	0.6 (-0.3, 1.5)	0.6 (-0.1, 1.2)	0.5 (-0.1, 1.1)	0.5 (-0.2, 1.2)
Malaysia										
rural	17.2 (16.2, 18.2)	13.5 (12.4, 14.6)	16.2 (15.2, 17.3)	19.1 (18.0, 20.1)	20.3 (19.2, 21.3)	19.5 (18.9, 20.0)	15.8 (15.1, 16.4)	18.5 (17.9, 19.1)	21.3 (20.7, 21.9)	22.5 (21.9, 23.2)
urban	17.4 (16.4, 18.5)	13.7 (12.6, 14.7)	16.4 (15.4, 17.5)	19.3 (18.3, 20.4)	20.5 (19.5, 21.6)	20.0 (19.4, 20.5)	16.2 (15.6, 16.8)	19.0 (18.4, 19.5)	21.8 (21.2, 22.4)	23.1 (22.4, 23.7)
urban-rural difference	0.2 (-0.2, 0.7)	0.2 (-0.3, 0.7)	0.2 (-0.2, 0.7)	0.2 (-0.2, 0.7)	0.3 (-0.2, 0.8)	0.5 (0.2, 0.8)	0.4 (0.0, 0.8)	0.5 (0.1, 0.8)	0.5 (0.2, 0.9)	0.6 (0.1, 0.9)
Maldives										
rural	16.7 (15.1, 18.1)	14.0 (11.1, 16.9)	15.8 (14.3, 17.3)	18.3 (16.9, 19.6)	18.7 (17.2, 20.2)	18.9 (17.8, 19.9)	16.2 (13.5, 18.9)	18.0 (16.9, 19.1)	20.5 (19.7, 21.3)	21.0 (20.0, 21.9)
urban	16.9 (15.3, 18.4)	14.3 (11.4, 17.2)	16.0 (14.4, 17.5)	18.4 (17.0, 19.8)	18.9 (17.4, 20.3)	19.4 (18.3, 20.4)	16.8 (14.0, 19.4)	18.5 (17.4, 19.6)	20.9 (20.1, 21.8)	21.4 (20.4, 22.4)
urban-rural difference	0.2 (-0.5, 0.8)	0.3 (-0.6, 1.2)	0.2 (-0.5, 0.9)	0.2 (-0.5, 0.8)	0.1 (-0.6, 0.8)	0.5 (-0.1, 1.1)	0.6 (-0.3, 1.4)	0.5 (-0.1, 1.1)	0.4 (-0.1, 1.0)	0.4 (-0.2, 1.0)
Myanmar										
rural	15.8 (14.2, 17.4)	13.2 (10.2, 16.1)	14.7 (13.0, 16.3)	17.3 (15.8, 18.8)	19.0 (17.4, 20.5)	17.6 (16.2, 19.1)	15.0 (12.2, 17.9)	16.5 (15.0, 18.0)	19.1 (17.8, 20.4)	20.8 (19.4, 22.1)
urban	16.1 (14.5, 17.7)	13.6 (10.6, 16.6)	15.0 (13.3, 16.6)	17.6 (16.1, 19.1)	19.2 (17.7, 20.8)	18.2 (16.8, 19.7)	15.7 (12.8, 18.6)	17.1 (15.6, 18.7)	19.7 (18.3, 21.1)	21.3 (20.0, 22.8)
urban-rural difference	0.3 (-0.3, 1.0)	0.4 (-0.5, 1.3)	0.3 (-0.3, 1.0)	0.3 (-0.3, 0.9)	0.3 (-0.4, 0.9)	0.6 (0.0, 1.2)	0.7 (-0.2, 1.6)	0.6 (0.0, 1.3)	0.6 (0.0, 1.2)	0.6 (-0.1, 1.2)
Philippines										
rural	16.5 (15.5, 17.5)	14.2 (13.2, 15.2)	15.2 (14.2, 16.2)	17.8 (16.8, 18.8)	20.0 (19.1, 21.1)	17.9 (17.3, 18.5)	15.6 (15.0, 16.2)	16.6 (16.0, 17.2)	19.2 (18.6, 19.8)	21.5 (20.9, 22.1)
urban	16.7 (15.7, 17.7)	14.5 (13.5, 15.5)	15.4 (14.4, 16.4)	17.9 (17.0, 19.0)	20.1 (19.1, 21.2)	18.4 (17.8, 19.0)	16.2 (15.6, 16.8)	17.1 (16.5, 17.7)	19.7 (19.1, 20.3)	21.9 (21.3, 22.5)
urban-rural difference	0.2 (-0.3, 0.6)	0.3 (-0.2, 0.8)	0.2 (-0.2, 0.7)	0.1 (-0.3, 0.6)	0.1 (-0.4, 0.5)	0.5 (0.2, 0.9)	0.6 (0.2, 1.0)	0.6 (0.2, 0.9)	0.5 (0.1, 0.8)	0.4 (0.0, 0.8)
Thailand										
rural	17.0 (16.4, 17.6)	14.3 (13.7, 14.9)	15.8 (15.2, 16.4)	18.5 (17.9, 19.1)	20.2 (19.5, 20.8)	19.4 (18.9, 20.0)	16.8 (16.2, 17.3)	18.3 (17.7, 18.8)	21.0 (20.4, 21.5)	22.6 (22.0, 23.3)
urban	17.5 (16.9, 18.1)	14.9 (14.3, 15.6)	16.4 (15.8, 17.0)	19.0 (18.4, 19.6)	20.6 (19.9, 21.3)	20.2 (19.7, 20.8)	17.7 (17.1, 18.3)	19.1 (18.5, 19.7)	21.7 (21.2, 22.2)	23.3 (22.7, 23.9)
urban-rural difference	0.5 (0.2, 0.9)	0.7 (0.2, 1.1)	0.6 (0.2, 1.0)	0.5 (0.1, 0.9)	0.4 (0.0, 0.9)	0.8 (0.4, 1.2)	0.9 (0.4, 1.4)	0.8 (0.4, 1.2)	0.7 (0.3, 1.1)	0.7 (0.2, 1.1)
Timor-Leste										
rural	15.5 (14.1, 17.0)	13.8 (12.4, 15.3)	14.2 (12.8, 15.8)	16.6 (15.2, 18.1)	18.9 (17.4, 20.5)	16.9 (16.2, 17.6)	15.2 (14.4, 16.0)	15.6 (14.9, 16.4)	18.0 (17.3, 18.7)	20.3 (19.5, 21.1)
urban	15.6 (14.2, 17.2)	14.0 (12.5, 15.8)	14.4 (12.9, 16.0)	16.7 (15.3, 18.2)	18.9 (17.4, 20.5)	17.3 (16.4, 18.1)	15.7 (14.6, 16.8)	16.0 (15.2, 16.9)	18.3 (17.5, 19.2)	20.5 (19.6, 21.5)
urban-rural difference	0.1 (-0.5, 0.8)	0.3 (-0.6, 1.1)	0.2 (-0.5, 0.9)	0.1 (-0.5, 0.7)	0.0 (-0.7, 0.7)	0.4 (-0.1, 0.9)	0.5 (-0.3, 1.3)	0.4 (-0.2, 1.0)	0.3 (-0.2, 0.8)	0.3 (-0.3, 0.8)
Viet Nam										
rural	15.8 (15.2, 16.3)	14.1 (13.5, 14.6)	14.5 (14.0, 15.1)	17.0 (16.4, 17.5)	18.8 (18.2, 19.4)	17.8 (17.2, 18.4)	16.0 (15.4, 16.7)	16.5 (15.9, 17.1)	18.9 (18.3, 19.5)	20.8 (20.2, 21.4)

Boys	Mean BMI in 1990 (kg/m^2)					Mean BMI in 2020 (kg/m^2)				
	Age-standardised	5 years	10 years	15 years	19 years	Age-standardised	5 years	10 years	15 years	19 years
urban	16.1 (15.5, 16.7)	14.5 (13.9, 15.1)	14.9 (14.3, 15.5)	17.2 (16.6, 17.8)	19.0 (18.4, 19.6)	18.6 (17.9, 19.2)	17.0 (16.3, 17.6)	17.4 (16.7, 18.0)	19.7 (19.1, 20.3)	21.5 (20.8, 22.1)
urban-rural difference	0.3 (-0.1, 0.7)	0.4 (0.0, 0.8)	0.3 (-0.1, 0.7)	0.2 (-0.2, 0.6)	0.2 (-0.3, 0.6)	0.8 (0.4, 1.2)	0.9 (0.5, 1.4)	0.8 (0.5, 1.3)	0.7 (0.4, 1.2)	0.7 (0.3, 1.1)
**High-income Asia Pacific**										
Japan										
rural	18.6 (18.3, 18.8)	15.4 (15.1, 15.6)	17.5 (17.3, 17.8)	20.4 (20.1, 20.6)	21.2 (20.9, 21.5)	18.7 (18.4, 19.0)	15.5 (15.2, 15.8)	17.7 (17.4, 18.0)	20.5 (20.2, 20.8)	21.4 (21.0, 21.7)
urban	18.6 (18.4, 18.9)	15.5 (15.2, 15.7)	17.6 (17.3, 17.8)	20.4 (20.1, 20.6)	21.2 (20.9, 21.5)	18.7 (18.4, 19.0)	15.6 (15.3, 15.9)	17.7 (17.4, 18.0)	20.5 (20.2, 20.8)	21.3 (21.0, 21.7)
urban-rural difference	0.0 (-0.1, 0.2)	0.1 (-0.1, 0.3)	0.1 (-0.1, 0.2)	0.0 (-0.2, 0.2)	0.0 (-0.2, 0.2)	0.0 (-0.1, 0.2)	0.1 (-0.1, 0.3)	0.0 (-0.1, 0.2)	0.0 (-0.2, 0.2)	0.0 (-0.2, 0.1)
Singapore										
rural	18.6 (16.7, 20.6)	15.4 (12.0, 18.8)	17.6 (15.5, 19.6)	20.3 (18.4, 22.2)	21.8 (19.7, 23.8)	19.6 (17.8, 21.4)	16.4 (13.2, 19.6)	18.5 (16.7, 20.5)	21.3 (19.6, 23.0)	22.7 (21.0, 24.5)
urban	18.9 (17.0, 20.9)	15.7 (12.3, 19.0)	17.9 (15.8, 19.9)	20.6 (18.7, 22.5)	22.1 (20.1, 24.0)	19.9 (18.2, 21.7)	16.6 (13.4, 19.8)	18.8 (17.0, 20.7)	21.6 (20.0, 23.3)	23.1 (21.5, 24.8)
urban-rural difference	0.3 (-0.4, 1.0)	0.3 (-0.6, 1.2)	0.3 (-0.4, 1.0)	0.3 (-0.3, 1.0)	0.3 (-0.4, 1.1)	0.3 (-0.3, 1.0)	0.3 (-0.6, 1.2)	0.3 (-0.4, 1.0)	0.3 (-0.3, 1.0)	0.4 (-0.4, 1.1)
South Korea										
rural	18.3 (17.2, 19.4)	14.7 (13.5, 15.8)	17.6 (16.5, 18.7)	20.0 (18.8, 21.1)	21.0 (19.9, 22.1)	19.7 (19.3, 20.0)	16.1 (15.7, 16.4)	19.0 (18.6, 19.3)	21.4 (21.0, 21.7)	22.4 (21.9, 22.8)
urban	18.7 (17.6, 19.7)	14.7 (13.5, 15.8)	17.8 (16.7, 18.9)	20.5 (19.4, 21.6)	21.8 (20.7, 22.9)	20.2 (19.8, 20.5)	16.2 (15.8, 16.5)	19.3 (19.0, 19.7)	22.0 (21.7, 22.4)	23.3 (22.9, 23.7)
urban-rural difference	0.4 (0.0, 0.7)	0.0 (-0.5, 0.4)	0.3 (-0.1, 0.6)	0.6 (0.1, 0.9)	0.8 (0.3, 1.2)	0.5 (0.3, 0.7)	0.1 (-0.2, 0.4)	0.4 (0.2, 0.6)	0.7 (0.4, 0.9)	0.9 (0.6, 1.2)
**High-income western**										
High-income English-speaking countries										
Australia										
rural	19.4 (18.5, 20.2)	15.6 (14.6, 16.5)	17.9 (17.0, 18.8)	21.4 (20.5, 22.3)	23.9 (22.9, 24.9)	20.4 (19.9, 21.0)	16.6 (16.0, 17.2)	18.9 (18.4, 19.5)	22.4 (21.9, 23.0)	25.0 (24.3, 25.6)
urban	19.2 (18.4, 20.1)	15.6 (14.7, 16.4)	17.8 (16.9, 18.7)	21.1 (20.3, 22.0)	23.6 (22.7, 24.6)	20.4 (19.9, 20.9)	16.8 (16.2, 17.4)	19.0 (18.4, 19.5)	22.3 (21.8, 22.9)	24.8 (24.2, 25.4)
urban-rural difference	-0.2 (-0.5, 0.3)	0.0 (-0.4, 0.5)	-0.1 (-0.5, 0.3)	-0.2 (-0.6, 0.2)	-0.3 (-0.8, 0.2)	0.0 (-0.3, 0.3)	0.2 (-0.2, 0.5)	0.1 (-0.2, 0.3)	-0.1 (-0.4, 0.2)	-0.2 (-0.5, 0.2)
Canada										
rural	18.7 (17.9, 19.4)	14.6 (13.6, 15.6)	17.3 (16.5, 18.1)	20.9 (20.1, 21.6)	22.6 (21.8, 23.3)	19.7 (19.0, 20.4)	15.6 (14.7, 16.5)	18.3 (17.6, 19.1)	21.9 (21.2, 22.6)	23.6 (22.7, 24.4)
urban	18.6 (17.8, 19.3)	14.4 (13.6, 15.2)	17.2 (16.4, 17.9)	20.8 (20.1, 21.5)	22.6 (21.9, 23.3)	19.8 (19.3, 20.4)	15.6 (15.1, 16.2)	18.4 (17.9, 19.0)	22.0 (21.5, 22.6)	23.8 (23.1, 24.5)
urban-rural difference	-0.1 (-0.7, 0.5)	-0.2 (-1.1, 0.7)	-0.1 (-0.8, 0.5)	-0.1 (-0.6, 0.5)	0.0 (-0.6, 0.6)	0.1 (-0.5, 0.7)	0.0 (-0.8, 0.9)	0.1 (-0.5, 0.8)	0.2 (-0.4, 0.8)	0.2 (-0.4, 0.9)
Ireland										
rural	18.7 (17.4, 19.9)	15.5 (14.2, 16.8)	17.3 (16.0, 18.6)	20.5 (19.2, 21.7)	22.4 (21.0, 23.9)	19.4 (19.0, 19.8)	16.3 (15.8, 16.7)	18.0 (17.6, 18.4)	21.2 (20.7, 21.6)	23.2 (22.3, 24.1)
urban	18.5 (17.3, 19.8)	15.3 (14.1, 16.6)	17.1 (15.9, 18.4)	20.4 (19.1, 21.6)	22.4 (21.0, 23.8)	19.5 (19.1, 19.9)	16.3 (15.8, 16.7)	18.1 (17.7, 18.5)	21.3 (20.9, 21.8)	23.4 (22.5, 24.2)
urban-rural difference	-0.1 (-0.6, 0.3)	-0.2 (-0.7, 0.3)	-0.2 (-0.6, 0.3)	-0.1 (-0.6, 0.4)	0.0 (-0.6, 0.5)	0.1 (-0.2, 0.4)	0.0 (-0.3, 0.3)	0.1 (-0.2, 0.4)	0.1 (-0.2, 0.5)	0.2 (-0.3, 0.7)
New Zealand										
rural	18.7 (18.0, 19.4)	15.1 (14.4, 15.9)	17.5 (16.7, 18.2)	20.5 (19.8, 21.3)	22.4 (21.7, 23.2)	20.0 (19.6, 20.4)	16.4 (16.0, 16.9)	18.8 (18.4, 19.2)	21.9 (21.4, 22.3)	23.7 (23.2, 24.3)
urban	19.1 (18.4, 19.7)	15.1 (14.4, 15.7)	17.7 (17.1, 18.4)	21.1 (20.5, 21.8)	23.2 (22.6, 23.9)	20.7 (20.3, 21.1)	16.7 (16.3, 17.1)	19.4 (19.0, 19.8)	22.8 (22.4, 23.2)	24.9 (24.4, 25.4)
urban-rural difference	0.4 (-0.1, 0.8)	0.0 (-0.6, 0.4)	0.3 (-0.2, 0.7)	0.6 (0.1, 1.0)	0.8 (0.3, 1.3)	0.7 (0.4, 1.0)	0.3 (-0.1, 0.6)	0.6 (0.3, 0.9)	0.9 (0.6, 1.2)	1.1 (0.8, 1.6)
United Kingdom										
rural	18.8 (18.3, 19.3)	15.5 (15.0, 15.9)	17.5 (17.0, 18.0)	20.6 (20.2, 21.1)	22.7 (22.2, 23.1)	19.5 (19.1, 20.0)	16.2 (15.7, 16.6)	18.2 (17.8, 18.7)	21.4 (20.9, 21.8)	23.4 (22.9, 23.8)
urban	18.8 (18.3, 19.3)	15.4 (14.9, 15.8)	17.5 (17.0, 18.0)	20.7 (20.2, 21.1)	22.7 (22.2, 23.2)	19.8 (19.4, 20.2)	16.4 (16.0, 16.8)	18.5 (18.1, 18.9)	21.6 (21.2, 22.1)	23.7 (23.2, 24.1)
urban-rural difference	0.0 (-0.3, 0.2)	-0.1 (-0.3, 0.2)	0.0 (-0.3, 0.2)	0.0 (-0.2, 0.2)	0.0 (-0.2, 0.3)	0.3 (0.1, 0.4)	0.2 (0.0, 0.4)	0.2 (0.0, 0.4)	0.3 (0.1, 0.5)	0.3 (0.1, 0.5)
United States of America										
rural	19.9 (19.4, 20.5)	15.5 (14.9, 16.0)	18.6 (18.1, 19.2)	22.2 (21.6, 22.7)	24.1 (23.5, 24.7)	21.0 (20.5, 21.6)	16.6 (16.0, 17.1)	19.8 (19.2, 20.3)	23.3 (22.7, 23.8)	25.2 (24.6, 25.8)
urban	19.8 (19.2, 20.4)	15.2 (14.7, 15.8)	18.5 (17.9, 19.1)	22.1 (21.5, 22.7)	24.1 (23.5, 24.7)	21.2 (20.6, 21.7)	16.6 (16.1, 17.1)	19.8 (19.3, 20.4)	23.5 (22.9, 24.0)	25.4 (24.8, 26.0)
urban-rural difference	-0.1 (-0.4, 0.2)	-0.2 (-0.5, 0.1)	-0.1 (-0.4, 0.1)	-0.1 (-0.4, 0.2)	0.0 (-0.4, 0.3)	0.1 (-0.1, 0.4)	0.0 (-0.2, 0.3)	0.1 (-0.1, 0.3)	0.2 (-0.1, 0.4)	0.2 (-0.1, 0.6)
Northwestern Europe										
Austria										
rural	18.7 (18.2, 19.1)	14.8 (14.2, 15.3)	17.4 (16.9, 17.9)	20.7 (20.3, 21.2)	22.2 (21.7, 22.8)	19.8 (19.4, 20.3)	15.9 (15.4, 16.4)	18.6 (18.1, 19.0)	21.9 (21.4, 22.4)	23.4 (22.8, 23.9)
urban	18.9 (18.6, 19.3)	14.7 (14.3, 15.2)	17.6 (17.2, 18.0)	21.1 (20.7, 21.5)	22.8 (22.3, 23.2)	20.1 (19.7, 20.5)	15.9 (15.4, 16.4)	18.7 (18.3, 19.2)	22.3 (21.8, 22.7)	23.9 (23.4, 24.4)
urban-rural difference	0.3 (-0.3, 0.8)	0.0 (-0.7, 0.5)	0.2 (-0.4, 0.7)	0.4 (-0.2, 0.9)	0.6 (-0.1, 1.2)	0.3 (-0.1, 0.6)	0.0 (-0.4, 0.4)	0.2 (-0.2, 0.5)	0.4 (0.0, 0.8)	0.6 (0.1, 1.0)
Belgium										
rural	18.2 (16.9, 19.4)	15.1 (13.7, 16.4)	17.1 (15.8, 18.3)	20.1 (18.9, 21.2)	20.5 (19.3, 21.8)	19.3 (18.4, 20.3)	16.2 (15.1, 17.4)	18.2 (17.2, 19.2)	21.2 (20.2, 22.2)	21.7 (20.6, 22.8)
urban	18.0 (16.8, 19.2)	14.8 (13.5, 16.0)	16.9 (15.6, 18.1)	20.0 (18.7, 21.2)	20.6 (19.2, 21.9)	19.1 (18.2, 19.9)	15.8 (14.9, 16.7)	17.9 (17.1, 18.8)	21.0 (20.2, 21.9)	21.6 (20.6, 22.6)
urban-rural difference	-0.1 (-0.8, 0.5)	-0.3 (-1.1, 0.5)	-0.2 (-0.8, 0.5)	-0.1 (-0.7, 0.5)	0.0 (-0.6, 0.7)	-0.3 (-0.8, 0.3)	-0.4 (-1.2, 0.4)	-0.3 (-0.9, 0.3)	-0.2 (-0.8, 0.4)	-0.1 (-0.7, 0.5)
Denmark										

Boys	Mean BMI in 1990 (kg/m^2)					Mean BMI in 2020 (kg/m^2)				
	Age-standardised	5 years	10 years	15 years	19 years	Age-standardised	5 years	10 years	15 years	19 years
rural	19.2 (18.4, 20.0)	15.7 (14.8, 16.6)	17.8 (16.9, 18.6)	21.0 (20.1, 21.8)	23.5 (22.7, 24.4)	19.4 (18.9, 19.9)	15.9 (15.4, 16.4)	17.9 (17.5, 18.4)	21.1 (20.7, 21.6)	23.7 (23.2, 24.2)
urban	19.1 (18.4, 19.8)	15.7 (14.9, 16.4)	17.7 (17.0, 18.4)	20.8 (20.1, 21.6)	23.4 (22.6, 24.1)	19.2 (18.7, 19.6)	15.7 (15.3, 16.2)	17.7 (17.3, 18.2)	20.9 (20.4, 21.3)	23.4 (23.0, 23.8)
urban-rural difference	-0.1 (-0.5, 0.4)	0.0 (-0.6, 0.5)	-0.1 (-0.5, 0.4)	-0.1 (-0.6, 0.3)	-0.2 (-0.6, 0.3)	-0.2 (-0.5, 0.0)	-0.2 (-0.6, 0.2)	-0.2 (-0.5, 0.1)	-0.3 (-0.6, 0.0)	-0.3 (-0.6, 0.0)
Finland										
rural	19.0 (17.6, 20.3)	14.9 (13.5, 16.2)	17.6 (16.3, 19.0)	21.0 (19.7, 22.3)	23.1 (21.4, 24.6)	20.2 (19.6, 20.8)	16.2 (15.5, 16.8)	18.9 (18.3, 19.5)	22.3 (21.6, 22.9)	24.3 (23.2, 25.4)
urban	18.9 (17.6, 20.2)	15.0 (13.6, 16.2)	17.6 (16.2, 18.8)	20.9 (19.5, 22.1)	22.8 (21.3, 24.4)	20.0 (19.4, 20.5)	16.0 (15.5, 16.6)	18.7 (18.1, 19.2)	21.9 (21.4, 22.5)	23.9 (22.9, 25.0)
urban-rural difference	-0.1 (-0.7, 0.5)	0.0 (-0.6, 0.7)	-0.1 (-0.6, 0.5)	-0.2 (-0.7, 0.4)	-0.2 (-0.8, 0.4)	-0.3 (-0.7, 0.1)	-0.1 (-0.6, 0.3)	-0.2 (-0.6, 0.2)	-0.3 (-0.7, 0.1)	-0.4 (-0.8, 0.1)
Germany										
rural	18.9 (18.2, 19.6)	14.8 (14.0, 15.6)	17.6 (16.8, 18.3)	20.9 (20.2, 21.6)	22.8 (22.2, 23.4)	19.6 (18.8, 20.4)	15.5 (14.7, 16.3)	18.3 (17.5, 19.1)	21.6 (20.8, 22.4)	23.6 (22.7, 24.4)
urban	18.8 (18.2, 19.4)	14.9 (14.2, 15.6)	17.6 (16.9, 18.2)	20.8 (20.1, 21.4)	22.6 (22.1, 23.1)	19.5 (18.8, 20.2)	15.5 (14.8, 16.3)	18.2 (17.5, 18.9)	21.4 (20.7, 22.2)	23.3 (22.5, 24.1)
urban-rural difference	-0.1 (-0.6, 0.4)	0.1 (-0.5, 0.7)	0.0 (-0.5, 0.5)	-0.1 (-0.6, 0.4)	-0.2 (-0.8, 0.4)	-0.1 (-0.5, 0.3)	0.0 (-0.5, 0.5)	-0.1 (-0.5, 0.4)	-0.2 (-0.6, 0.3)	-0.3 (-0.8, 0.3)
Greenland										
rural	18.2 (16.5, 19.9)	14.7 (11.8, 17.6)	16.9 (15.2, 18.7)	20.0 (18.4, 21.6)	21.9 (20.3, 23.5)	19.0 (17.4, 20.7)	15.5 (12.6, 18.4)	17.7 (16.0, 19.5)	20.8 (19.3, 22.4)	22.7 (21.1, 24.3)
urban	18.2 (16.5, 19.9)	14.7 (11.8, 17.6)	16.9 (15.2, 18.7)	20.0 (18.4, 21.7)	21.9 (20.2, 23.6)	18.9 (17.3, 20.5)	15.4 (12.6, 18.3)	17.7 (16.0, 19.4)	20.8 (19.2, 22.3)	22.6 (21.1, 24.3)
urban-rural difference	0.0 (-0.7, 0.7)	0.0 (-0.9, 0.9)	0.0 (-0.7, 0.7)	0.0 (-0.7, 0.7)	0.0 (-0.7, 0.7)	-0.1 (-0.7, 0.5)	-0.1 (-1.0, 0.8)	-0.1 (-0.7, 0.6)	-0.1 (-0.7, 0.5)	-0.1 (-0.8, 0.6)
Iceland										
rural	18.6 (16.4, 20.8)	15.1 (11.9, 18.2)	17.4 (15.2, 19.5)	20.5 (18.3, 22.6)	22.2 (19.6, 24.7)	19.4 (17.2, 21.6)	15.9 (12.7, 19.1)	18.2 (16.0, 20.4)	21.3 (19.2, 23.4)	23.0 (20.4, 25.5)
urban	18.6 (16.4, 20.8)	15.1 (11.9, 18.2)	17.4 (15.2, 19.6)	20.5 (18.3, 22.6)	22.2 (19.6, 24.7)	19.4 (17.1, 21.6)	15.8 (12.6, 19.0)	18.1 (15.9, 20.3)	21.2 (19.1, 23.4)	22.9 (20.3, 25.4)
urban-rural difference	0.0 (-0.6, 0.7)	0.0 (-0.9, 0.9)	0.0 (-0.7, 0.7)	0.0 (-0.6, 0.7)	0.0 (-0.7, 0.8)	-0.1 (-0.7, 0.6)	-0.1 (-0.9, 0.8)	-0.1 (-0.7, 0.6)	0.0 (-0.6, 0.6)	0.0 (-0.7, 0.7)
Luxembourg										
rural	18.4 (16.6, 20.1)	14.8 (11.9, 17.7)	17.2 (15.4, 19.0)	20.3 (18.6, 21.9)	21.6 (19.9, 23.2)	19.2 (17.5, 20.8)	15.6 (12.6, 18.5)	18.0 (16.2, 19.8)	21.1 (19.5, 22.7)	22.4 (20.8, 24.0)
urban	18.4 (16.7, 20.2)	14.8 (11.8, 17.7)	17.2 (15.4, 19.1)	20.3 (18.7, 22.0)	21.7 (20.0, 23.4)	19.1 (17.5, 20.9)	15.5 (12.6, 18.4)	17.9 (16.2, 19.8)	21.1 (19.4, 22.7)	22.4 (20.8, 23.9)
urban-rural difference	0.0 (-0.6, 0.7)	0.0 (-0.9, 0.9)	0.0 (-0.7, 0.7)	0.1 (-0.6, 0.7)	0.1 (-0.6, 0.8)	0.0 (-0.6, 0.6)	-0.1 (-0.9, 0.8)	0.0 (-0.7, 0.6)	0.0 (-0.6, 0.6)	0.0 (-0.7, 0.7)
Netherlands										
rural	18.1 (16.9, 19.3)	15.2 (13.8, 16.6)	16.9 (15.7, 18.1)	19.6 (18.4, 20.9)	21.4 (20.0, 22.9)	18.7 (17.8, 19.7)	15.9 (14.7, 17.0)	17.5 (16.6, 18.5)	20.3 (19.3, 21.3)	22.1 (20.8, 23.4)
urban	18.2 (17.0, 19.4)	15.4 (14.0, 16.8)	17.0 (15.8, 18.2)	19.7 (18.6, 20.9)	21.5 (20.2, 22.9)	18.8 (17.8, 19.8)	16.0 (14.8, 17.2)	17.6 (16.6, 18.6)	20.3 (19.4, 21.3)	22.1 (20.9, 23.3)
urban-rural difference	0.1 (-0.4, 0.6)	0.2 (-0.5, 0.8)	0.1 (-0.4, 0.7)	0.1 (-0.4, 0.6)	0.1 (-0.6, 0.7)	0.1 (-0.4, 0.5)	0.1 (-0.5, 0.7)	0.1 (-0.4, 0.5)	0.0 (-0.4, 0.5)	0.0 (-0.6, 0.6)
Norway										
rural	18.6 (17.4, 19.8)	15.9 (14.4, 17.4)	17.4 (16.2, 18.5)	20.1 (18.9, 21.3)	21.9 (20.7, 23.2)	19.1 (18.1, 20.1)	16.4 (15.2, 17.7)	17.9 (16.9, 18.9)	20.6 (19.6, 21.6)	22.4 (21.3, 23.5)
urban	18.5 (17.3, 19.7)	15.6 (14.1, 17.0)	17.2 (16.0, 18.4)	20.0 (18.8, 21.3)	22.0 (20.7, 23.3)	18.9 (18.0, 19.8)	16.0 (14.9, 17.1)	17.6 (16.7, 18.5)	20.5 (19.5, 21.4)	22.4 (21.3, 23.5)
urban-rural difference	-0.1 (-0.7, 0.4)	-0.3 (-1.0, 0.4)	-0.2 (-0.7, 0.4)	0.0 (-0.6, 0.5)	0.1 (-0.5, 0.7)	-0.2 (-0.7, 0.3)	-0.4 (-1.2, 0.3)	-0.3 (-0.8, 0.3)	-0.1 (-0.7, 0.4)	0.0 (-0.6, 0.6)
Sweden										
rural	19.0 (18.7, 19.3)	15.5 (14.9, 16.2)	17.8 (17.4, 18.2)	21.0 (20.7, 21.2)	22.1 (21.7, 22.4)	19.8 (19.1, 20.5)	16.3 (15.5, 17.1)	18.6 (17.9, 19.2)	21.8 (21.1, 22.5)	22.9 (22.1, 23.6)
urban	18.7 (18.5, 18.9)	15.3 (14.9, 15.7)	17.5 (17.3, 17.8)	20.7 (20.4, 20.9)	21.7 (21.3, 22.1)	19.4 (18.9, 20.0)	16.0 (15.4, 16.7)	18.2 (17.7, 18.8)	21.4 (20.7, 22.0)	22.4 (21.7, 23.1)
urban-rural difference	-0.3 (-0.6, 0.0)	-0.2 (-0.8, 0.4)	-0.3 (-0.7, 0.1)	-0.3 (-0.6, -0.1)	-0.4 (-0.6, -0.1)	-0.4 (-0.8, 0.1)	-0.3 (-0.9, 0.4)	-0.3 (-0.8, 0.1)	-0.4 (-0.8, 0.0)	-0.4 (-0.9, 0.0)
Switzerland										
rural	18.5 (17.3, 19.7)	15.0 (13.8, 16.2)	17.2 (16.1, 18.4)	20.2 (19.0, 21.4)	22.5 (21.3, 23.7)	19.2 (18.6, 19.8)	15.7 (15.0, 16.3)	17.9 (17.3, 18.5)	20.8 (20.2, 21.5)	23.2 (22.6, 23.8)
urban	18.6 (17.4, 19.8)	15.1 (13.9, 16.3)	17.3 (16.1, 18.6)	20.3 (19.1, 21.5)	22.6 (21.4, 23.8)	19.2 (18.6, 19.8)	15.7 (15.0, 16.3)	17.9 (17.3, 18.5)	20.8 (20.2, 21.5)	23.2 (22.6, 23.8)
urban-rural difference	0.1 (-0.3, 0.6)	0.1 (-0.4, 0.6)	0.1 (-0.3, 0.6)	0.1 (-0.4, 0.6)	0.1 (-0.4, 0.6)	0.0 (-0.3, 0.3)	0.0 (-0.4, 0.4)	0.0 (-0.3, 0.3)	0.0 (-0.3, 0.3)	0.0 (-0.3, 0.3)
Southwestern Europe										
Andorra										
rural	19.2 (17.0, 21.3)	15.4 (12.2, 18.6)	18.1 (15.9, 20.2)	21.0 (18.9, 23.1)	22.7 (20.1, 25.3)	19.7 (17.5, 21.9)	15.9 (12.7, 19.2)	18.6 (16.5, 20.8)	21.5 (19.4, 23.7)	23.2 (20.6, 25.8)
urban	19.2 (17.0, 21.3)	15.5 (12.3, 18.8)	18.1 (16.0, 20.3)	21.0 (18.9, 23.1)	22.6 (20.0, 25.2)	19.7 (17.6, 21.9)	16.1 (12.8, 19.4)	18.7 (16.5, 20.9)	21.5 (19.5, 23.7)	23.1 (20.6, 25.8)
urban-rural difference	0.0 (-0.6, 0.7)	0.1 (-0.8, 1.0)	0.1 (-0.6, 0.7)	0.0 (-0.7, 0.6)	-0.1 (-0.8, 0.6)	0.1 (-0.6, 0.7)	0.2 (-0.7, 1.0)	0.1 (-0.6, 0.8)	0.0 (-0.6, 0.7)	-0.1 (-0.7, 0.7)
Cyprus										
rural	19.8 (18.5, 21.1)	15.4 (13.9, 16.8)	18.7 (17.3, 20.0)	21.8 (20.5, 23.2)	23.7 (21.9, 25.5)	20.3 (19.6, 21.0)	15.9 (15.1, 16.8)	19.2 (18.5, 19.9)	22.4 (21.6, 23.2)	24.2 (22.7, 25.8)
urban	19.7 (18.4, 21.0)	15.5 (14.2, 16.9)	18.7 (17.4, 20.0)	21.7 (20.4, 23.0)	23.4 (21.6, 25.2)	20.3 (19.7, 20.9)	16.1 (15.4, 16.8)	19.2 (18.6, 19.8)	22.3 (21.6, 23.0)	24.0 (22.6, 25.5)
urban-rural difference	0.0 (-0.6, 0.5)	0.1 (-0.6, 0.9)	0.0 (-0.6, 0.6)	-0.1 (-0.7, 0.4)	-0.2 (-0.9, 0.4)	0.0 (-0.5, 0.4)	0.2 (-0.5, 0.8)	0.0 (-0.5, 0.5)	-0.1 (-0.6, 0.4)	-0.2 (-0.9, 0.4)
France										
rural	18.1 (16.9, 19.4)	14.9 (13.6, 16.1)	17.0 (15.8, 18.3)	19.9 (18.6, 21.2)	21.2 (19.9, 22.6)	18.4 (17.8, 19.1)	15.1 (14.4, 15.9)	17.3 (16.6, 18.0)	20.1 (19.5, 20.8)	21.5 (20.8, 22.3)
urban	18.3 (17.1, 19.6)	14.9 (13.7, 16.2)	17.2 (16.0, 18.5)	20.1 (18.9, 21.4)	21.6 (20.3, 22.9)	18.6 (18.0, 19.2)	15.2 (14.5, 15.9)	17.5 (16.8, 18.1)	20.4 (19.8, 21.1)	21.9 (21.2, 22.5)

Boys	Mean BMI in 1990 (kg/m^2)					Mean BMI in 2020 (kg/m^2)				
	Age-standardised	5 years	10 years	15 years	19 years	Age-standardised	5 years	10 years	15 years	19 years
urban-rural difference	0.2 (-0.3, 0.7)	0.1 (-0.5, 0.7)	0.2 (-0.3, 0.7)	0.3 (-0.2, 0.8)	0.4 (-0.2, 0.9)	0.2 (-0.2, 0.6)	0.1 (-0.4, 0.6)	0.2 (-0.2, 0.6)	0.3 (-0.1, 0.7)	0.4 (-0.2, 0.9)
Greece										
rural	19.5 (18.5, 20.5)	15.2 (14.2, 16.2)	18.5 (17.5, 19.5)	21.5 (20.5, 22.5)	23.0 (21.9, 24.1)	20.3 (19.7, 20.8)	16.0 (15.3, 16.6)	19.3 (18.7, 19.8)	22.3 (21.7, 22.9)	23.8 (23.1, 24.5)
urban	19.4 (18.5, 20.4)	15.2 (14.2, 16.2)	18.4 (17.5, 19.4)	21.4 (20.5, 22.5)	22.9 (21.9, 24.0)	20.1 (19.6, 20.7)	15.9 (15.3, 16.5)	19.2 (18.6, 19.7)	22.2 (21.6, 22.7)	23.6 (22.9, 24.3)
urban-rural difference	-0.1 (-0.4, 0.3)	0.0 (-0.4, 0.4)	0.0 (-0.4, 0.3)	-0.1 (-0.5, 0.3)	-0.1 (-0.6, 0.4)	-0.1 (-0.4, 0.2)	-0.1 (-0.4, 0.3)	-0.1 (-0.4, 0.2)	-0.2 (-0.5, 0.2)	-0.2 (-0.7, 0.2)
Israel										
rural	18.6 (18.0, 19.2)	14.1 (12.8, 15.5)	17.7 (17.1, 18.4)	20.8 (20.3, 21.2)	21.8 (21.3, 22.3)	19.8 (19.2, 20.4)	15.3 (14.2, 16.4)	18.9 (18.2, 19.5)	21.9 (21.3, 22.6)	23.0 (22.3, 23.6)
urban	18.6 (18.0, 19.1)	14.0 (12.8, 15.2)	17.6 (17.0, 18.2)	20.8 (20.3, 21.2)	21.9 (21.4, 22.4)	19.8 (19.3, 20.3)	15.2 (14.2, 16.1)	18.8 (18.3, 19.4)	22.0 (21.4, 22.6)	23.1 (22.5, 23.7)
urban-rural difference	-0.1 (-0.5, 0.4)	-0.2 (-0.9, 0.6)	-0.1 (-0.6, 0.4)	0.0 (-0.3, 0.3)	0.1 (-0.3, 0.4)	0.0 (-0.4, 0.4)	-0.1 (-0.8, 0.6)	0.0 (-0.5, 0.5)	0.1 (-0.3, 0.4)	0.1 (-0.2, 0.5)
Italy										
rural	19.8 (19.0, 20.6)	16.8 (15.8, 17.8)	18.8 (18.0, 19.6)	21.3 (20.5, 22.1)	23.2 (22.2, 24.2)	19.6 (19.0, 20.2)	16.6 (15.8, 17.4)	18.6 (18.0, 19.1)	21.1 (20.4, 21.7)	22.9 (22.1, 23.8)
urban	19.7 (18.9, 20.5)	17.1 (16.1, 18.1)	18.8 (18.0, 19.6)	21.0 (20.2, 21.8)	22.6 (21.6, 23.6)	19.5 (18.9, 20.0)	16.9 (16.2, 17.6)	18.6 (18.0, 19.1)	20.8 (20.2, 21.4)	22.4 (21.6, 23.1)
urban-rural difference	-0.1 (-0.5, 0.3)	0.3 (-0.2, 0.9)	0.0 (-0.4, 0.5)	-0.3 (-0.8, 0.2)	-0.6 (-1.2, 0.0)	-0.1 (-0.4, 0.2)	0.3 (-0.1, 0.7)	0.0 (-0.3, 0.3)	-0.3 (-0.7, 0.1)	-0.6 (-1.1, 0.0)
Malta										
rural	18.9 (17.5, 20.4)	15.5 (14.0, 17.1)	17.8 (16.4, 19.2)	20.7 (19.1, 22.3)	22.5 (20.5, 24.7)	19.3 (18.5, 20.2)	16.0 (14.9, 17.0)	18.2 (17.4, 19.0)	21.1 (20.0, 22.2)	23.0 (21.1, 24.9)
urban	19.2 (17.8, 20.6)	16.0 (14.6, 17.5)	18.1 (16.7, 19.5)	20.8 (19.2, 22.4)	22.5 (20.5, 24.6)	19.7 (18.9, 20.5)	16.6 (15.6, 17.5)	18.6 (17.9, 19.3)	21.3 (20.3, 22.4)	23.0 (21.3, 24.9)
urban-rural difference	0.3 (-0.4, 0.8)	0.5 (-0.3, 1.2)	0.3 (-0.3, 0.9)	0.1 (-0.5, 0.8)	0.0 (-0.7, 0.7)	0.3 (-0.2, 0.9)	0.6 (0.0, 1.2)	0.4 (-0.1, 0.9)	0.2 (-0.3, 0.8)	0.1 (-0.7, 0.8)
Portugal										
rural	18.9 (18.5, 19.2)	15.5 (15.0, 16.0)	17.8 (17.4, 18.2)	20.6 (20.2, 20.9)	22.3 (22.0, 22.6)	19.4 (18.8, 19.9)	16.0 (15.4, 16.5)	18.3 (17.8, 18.8)	21.1 (20.5, 21.6)	22.8 (22.2, 23.5)
urban	18.9 (18.5, 19.2)	15.6 (15.2, 16.1)	17.9 (17.5, 18.3)	20.5 (20.1, 20.8)	22.1 (21.9, 22.4)	19.4 (18.8, 19.9)	16.1 (15.6, 16.7)	18.4 (17.8, 18.9)	21.0 (20.4, 21.5)	22.6 (22.0, 23.3)
urban-rural difference	0.0 (-0.3, 0.3)	0.2 (-0.3, 0.6)	0.1 (-0.3, 0.4)	-0.1 (-0.3, 0.2)	-0.2 (-0.4, 0.0)	0.0 (-0.3, 0.3)	0.2 (-0.1, 0.5)	0.0 (-0.2, 0.3)	-0.1 (-0.4, 0.2)	-0.2 (-0.6, 0.2)
Spain										
rural	20.8 (19.8, 22.0)	16.9 (15.7, 18.1)	19.8 (18.7, 21.0)	22.6 (21.6, 23.8)	24.4 (23.3, 25.6)	20.1 (19.6, 20.6)	16.2 (15.7, 16.7)	19.1 (18.7, 19.6)	21.9 (21.4, 22.4)	23.7 (23.0, 24.4)
urban	20.7 (19.6, 21.8)	17.0 (15.9, 18.2)	19.8 (18.7, 20.9)	22.4 (21.4, 23.6)	24.1 (23.0, 25.2)	20.0 (19.6, 20.5)	16.3 (15.8, 16.8)	19.1 (18.7, 19.6)	21.8 (21.3, 22.3)	23.4 (22.7, 24.0)
urban-rural difference	-0.1 (-0.6, 0.3)	0.1 (-0.4, 0.6)	0.0 (-0.5, 0.4)	-0.2 (-0.7, 0.2)	-0.3 (-0.9, 0.2)	-0.1 (-0.3, 0.2)	0.2 (-0.2, 0.5)	0.0 (-0.3, 0.3)	-0.2 (-0.5, 0.2)	-0.3 (-0.8, 0.2)
**Latin America and Caribbean**										
Andean Latin America										
Bolivia										
rural	18.4 (16.7, 20.1)	15.6 (12.6, 18.6)	17.4 (15.7, 19.1)	19.9 (18.2, 21.5)	21.2 (19.5, 23.0)	20.5 (19.3, 21.6)	17.7 (15.0, 20.5)	19.5 (18.3, 20.7)	22.0 (21.0, 22.9)	23.3 (22.3, 24.3)
urban	18.8 (17.1, 20.6)	16.3 (13.3, 19.3)	17.9 (16.1, 19.6)	20.2 (18.6, 21.9)	21.5 (19.7, 23.2)	21.2 (20.1, 22.3)	18.6 (15.9, 21.3)	20.2 (19.1, 21.4)	22.6 (21.6, 23.6)	23.8 (22.8, 24.9)
urban-rural difference	0.5 (-0.2, 1.1)	0.6 (-0.3, 1.6)	0.5 (-0.2, 1.2)	0.4 (-0.3, 1.0)	0.3 (-0.5, 0.9)	0.7 (0.1, 1.3)	0.9 (0.0, 1.8)	0.8 (0.1, 1.4)	0.6 (0.1, 1.2)	0.5 (-0.1, 1.1)
Ecuador										
rural	17.9 (16.3, 19.5)	14.8 (13.2, 16.4)	16.9 (15.3, 18.5)	19.5 (17.9, 21.1)	21.3 (19.6, 22.8)	19.8 (19.1, 20.4)	16.7 (16.0, 17.4)	18.8 (18.1, 19.4)	21.4 (20.7, 22.0)	23.1 (22.4, 23.8)
urban	18.3 (16.8, 19.9)	15.2 (13.6, 16.8)	17.3 (15.7, 18.9)	19.9 (18.4, 21.5)	21.7 (20.1, 23.3)	20.4 (19.7, 21.0)	17.2 (16.5, 17.9)	19.3 (18.7, 20.0)	21.9 (21.3, 22.6)	23.7 (23.0, 24.4)
urban-rural difference	0.4 (-0.3, 1.1)	0.4 (-0.4, 1.1)	0.4 (-0.3, 1.1)	0.4 (-0.3, 1.1)	0.4 (-0.3, 1.1)	0.6 (0.2, 1.0)	0.6 (0.1, 1.0)	0.6 (0.1, 1.0)	0.6 (0.2, 1.0)	0.6 (0.1, 1.1)
Peru										
rural	17.5 (16.0, 19.0)	14.5 (13.0, 16.1)	16.2 (14.7, 17.7)	19.0 (17.5, 20.5)	21.3 (19.8, 22.8)	19.3 (18.9, 19.7)	16.4 (15.9, 16.8)	18.0 (17.6, 18.5)	20.9 (20.5, 21.3)	23.1 (22.7, 23.5)
urban	18.3 (16.7, 19.7)	15.6 (14.0, 17.1)	17.1 (15.5, 18.6)	19.7 (18.2, 21.2)	21.8 (20.3, 23.3)	20.4 (20.0, 20.8)	17.7 (17.2, 18.2)	19.2 (18.8, 19.7)	21.9 (21.5, 22.3)	24.0 (23.6, 24.4)
urban-rural difference	0.8 (0.2, 1.4)	1.0 (0.3, 1.7)	0.9 (0.2, 1.5)	0.7 (0.0, 1.3)	0.6 (-0.1, 1.2)	1.1 (0.8, 1.4)	1.3 (0.9, 1.8)	1.2 (0.9, 1.5)	1.0 (0.7, 1.3)	0.9 (0.6, 1.2)
Caribbean										
Antigua and Barbuda										
rural	18.9 (16.9, 20.8)	15.6 (12.2, 18.9)	17.7 (15.8, 19.7)	20.6 (18.8, 22.4)	22.2 (19.8, 24.5)	20.7 (18.9, 22.4)	17.4 (14.0, 20.6)	19.5 (17.8, 21.2)	22.4 (20.8, 23.9)	24.0 (21.8, 26.2)
urban	19.3 (17.3, 21.3)	16.1 (12.8, 19.5)	18.2 (16.2, 20.2)	21.0 (19.2, 22.9)	22.6 (20.3, 25.0)	21.2 (19.5, 22.9)	18.0 (14.8, 21.2)	20.1 (18.4, 21.8)	22.9 (21.3, 24.5)	24.5 (22.3, 26.7)
urban-rural difference	0.5 (-0.3, 1.2)	0.5 (-0.5, 1.5)	0.5 (-0.3, 1.3)	0.4 (-0.3, 1.2)	0.4 (-0.4, 1.2)	0.6 (-0.2, 1.3)	0.6 (-0.4, 1.6)	0.6 (-0.2, 1.3)	0.5 (-0.2, 1.2)	0.5 (-0.3, 1.3)
Bahamas										
rural	18.8 (17.0, 20.6)	15.6 (12.3, 18.9)	17.7 (15.9, 19.5)	20.5 (18.8, 22.2)	22.0 (19.7, 24.3)	20.7 (19.1, 22.2)	17.5 (14.3, 20.6)	19.6 (18.1, 21.1)	22.4 (21.0, 23.8)	23.9 (21.9, 25.8)
urban	19.4 (17.5, 21.1)	16.2 (12.9, 19.4)	18.3 (16.5, 20.0)	21.0 (19.4, 22.6)	22.5 (20.2, 24.7)	21.3 (19.8, 22.8)	18.2 (15.1, 21.3)	20.2 (18.8, 21.7)	23.0 (21.7, 24.3)	24.5 (22.5, 26.3)
urban-rural difference	0.5 (-0.2, 1.3)	0.6 (-0.4, 1.6)	0.6 (-0.2, 1.4)	0.5 (-0.3, 1.3)	0.5 (-0.4, 1.3)	0.6 (-0.1, 1.3)	0.7 (-0.3, 1.7)	0.7 (-0.1, 1.4)	0.6 (-0.1, 1.3)	0.6 (-0.2, 1.3)
Barbados										
rural	18.4 (16.6, 20.2)	15.1 (11.9, 18.4)	17.3 (15.5, 19.1)	20.2 (18.5, 21.8)	21.7 (19.5, 24.0)	20.2 (18.6, 21.8)	16.9 (13.8, 20.1)	19.1 (17.5, 20.7)	21.9 (20.5, 23.3)	23.5 (21.5, 25.6)
urban	18.9 (17.1, 20.8)	15.7 (12.4, 19.0)	17.8 (16.0, 19.6)	20.6 (18.9, 22.3)	22.2 (19.9, 24.3)	20.8 (19.2, 22.4)	17.6 (14.4, 20.7)	19.7 (18.1, 21.3)	22.5 (21.0, 23.9)	24.0 (22.0, 26.0)

Boys	Mean BMI in 1990 (kg/m^2)					Mean BMI in 2020 (kg/m^2)				
	Age-standardised	5 years	10 years	15 years	19 years	Age-standardised	5 years	10 years	15 years	19 years
urban-rural difference	0.5 (-0.3, 1.2)	0.6 (-0.4, 1.6)	0.5 (-0.3, 1.3)	0.5 (-0.3, 1.2)	0.4 (-0.4, 1.2)	0.6 (-0.2, 1.3)	0.6 (-0.4, 1.6)	0.6 (-0.2, 1.4)	0.6 (-0.2, 1.3)	0.5 (-0.3, 1.3)
Belize										
rural	18.6 (16.8, 20.5)	15.3 (12.1, 18.6)	17.5 (15.6, 19.3)	20.4 (18.6, 22.1)	21.9 (19.6, 24.3)	20.4 (18.8, 22.1)	17.1 (14.0, 20.3)	19.3 (17.7, 21.0)	22.2 (20.7, 23.7)	23.7 (21.6, 25.8)
urban	19.1 (17.2, 21.0)	15.9 (12.7, 19.1)	18.0 (16.1, 19.8)	20.8 (19.1, 22.6)	22.4 (20.1, 24.7)	21.0 (19.4, 22.7)	17.8 (14.7, 20.9)	19.9 (18.3, 21.5)	22.7 (21.2, 24.2)	24.3 (22.1, 26.4)
urban-rural difference	0.5 (-0.2, 1.2)	0.6 (-0.4, 1.6)	0.5 (-0.3, 1.3)	0.5 (-0.3, 1.2)	0.4 (-0.4, 1.3)	0.6 (-0.1, 1.3)	0.7 (-0.3, 1.7)	0.6 (-0.2, 1.4)	0.6 (-0.1, 1.3)	0.5 (-0.3, 1.3)
Bermuda										
rural	17.8 (15.5, 20.1)	14.5 (10.9, 18.1)	16.7 (14.4, 19.0)	19.6 (17.4, 21.8)	21.2 (18.5, 23.9)	19.5 (17.4, 21.9)	16.2 (12.7, 19.8)	18.4 (16.3, 20.7)	21.3 (19.2, 23.5)	22.9 (20.3, 25.5)
urban	18.3 (16.0, 20.6)	15.1 (11.6, 18.7)	17.2 (14.9, 19.6)	20.0 (17.8, 22.3)	21.6 (18.9, 24.4)	20.1 (17.9, 22.4)	16.9 (13.4, 20.5)	19.0 (16.9, 21.3)	21.8 (19.8, 24.1)	23.4 (20.8, 26.0)
urban-rural difference	0.5 (-0.3, 1.2)	0.6 (-0.4, 1.6)	0.5 (-0.3, 1.3)	0.5 (-0.3, 1.2)	0.4 (-0.4, 1.3)	0.6 (-0.1, 1.3)	0.7 (-0.4, 1.7)	0.6 (-0.2, 1.4)	0.6 (-0.1, 1.3)	0.5 (-0.2, 1.3)
Cuba										
rural	17.5 (16.1, 19.0)	14.1 (11.0, 17.2)	16.5 (15.0, 18.0)	19.3 (18.1, 20.6)	20.5 (19.2, 21.9)	18.9 (17.4, 20.4)	15.5 (12.3, 18.6)	17.9 (16.3, 19.4)	20.7 (19.3, 22.0)	21.9 (20.5, 23.3)
urban	18.1 (16.8, 19.4)	14.9 (11.7, 17.9)	17.1 (15.7, 18.5)	19.8 (18.7, 21.0)	21.0 (19.8, 22.1)	19.6 (18.1, 21.1)	16.3 (13.1, 19.4)	18.6 (17.1, 20.1)	21.3 (20.0, 22.6)	22.4 (21.1, 23.7)
urban-rural difference	0.6 (-0.1, 1.3)	0.7 (-0.3, 1.7)	0.6 (-0.1, 1.4)	0.5 (-0.2, 1.2)	0.4 (-0.3, 1.2)	0.7 (0.0, 1.3)	0.8 (-0.2, 1.8)	0.7 (0.0, 1.4)	0.6 (0.0, 1.2)	0.5 (-0.1, 1.2)
Dominica										
rural	17.5 (15.7, 19.3)	14.3 (11.0, 17.4)	16.5 (14.6, 18.3)	19.2 (17.6, 20.8)	20.6 (18.6, 22.5)	19.2 (17.6, 20.8)	16.0 (12.8, 19.2)	18.2 (16.5, 19.8)	20.9 (19.6, 22.4)	22.3 (20.5, 24.1)
urban	18.0 (16.2, 19.7)	14.8 (11.5, 18.1)	17.0 (15.2, 18.7)	19.7 (18.1, 21.3)	21.0 (19.0, 22.9)	19.8 (18.2, 21.3)	16.7 (13.4, 19.7)	18.8 (17.2, 20.3)	21.5 (20.1, 22.8)	22.8 (21.0, 24.5)
urban-rural difference	0.5 (-0.2, 1.2)	0.6 (-0.4, 1.6)	0.5 (-0.2, 1.3)	0.5 (-0.3, 1.2)	0.4 (-0.4, 1.2)	0.6 (-0.1, 1.3)	0.7 (-0.3, 1.7)	0.6 (-0.1, 1.4)	0.6 (-0.1, 1.2)	0.5 (-0.3, 1.3)
Dominican Republic										
rural	17.0 (15.3, 18.6)	13.7 (10.6, 16.9)	15.8 (14.1, 17.5)	18.7 (17.2, 20.1)	20.5 (19.1, 22.0)	19.0 (17.7, 20.3)	15.7 (12.6, 18.7)	17.8 (16.4, 19.2)	20.7 (19.6, 21.8)	22.5 (21.5, 23.6)
urban	17.4 (15.8, 19.0)	14.2 (11.1, 17.4)	16.3 (14.6, 17.9)	19.1 (17.6, 20.5)	20.9 (19.5, 22.4)	19.5 (18.2, 20.7)	16.3 (13.3, 19.3)	18.3 (17.0, 19.6)	21.2 (20.2, 22.2)	23.0 (22.0, 24.0)
urban-rural difference	0.4 (-0.3, 1.2)	0.5 (-0.5, 1.5)	0.5 (-0.3, 1.2)	0.4 (-0.3, 1.1)	0.4 (-0.4, 1.1)	0.5 (-0.1, 1.1)	0.6 (-0.4, 1.6)	0.5 (-0.1, 1.2)	0.5 (-0.1, 1.0)	0.4 (-0.2, 1.0)
Grenada										
rural	17.8 (15.4, 20.1)	14.5 (11.1, 18.1)	16.7 (14.3, 19.0)	19.6 (17.3, 21.8)	21.2 (18.5, 23.8)	19.5 (17.2, 21.8)	16.2 (12.7, 19.7)	18.4 (16.1, 20.7)	21.3 (19.1, 23.4)	22.9 (20.3, 25.5)
urban	18.3 (16.0, 20.6)	15.1 (11.6, 18.6)	17.2 (14.9, 19.5)	20.0 (17.8, 22.3)	21.6 (18.9, 24.3)	20.1 (17.9, 22.4)	16.9 (13.4, 20.4)	19.0 (16.8, 21.3)	21.9 (19.7, 24.0)	23.4 (20.8, 26.0)
urban-rural difference	0.5 (-0.3, 1.3)	0.6 (-0.5, 1.6)	0.5 (-0.3, 1.3)	0.5 (-0.3, 1.2)	0.4 (-0.4, 1.3)	0.6 (-0.1, 1.3)	0.7 (-0.3, 1.7)	0.6 (-0.1, 1.4)	0.6 (-0.2, 1.3)	0.5 (-0.3, 1.3)
Guyana										
rural	16.8 (15.1, 18.5)	13.6 (10.4, 16.8)	15.6 (13.9, 17.3)	18.5 (16.9, 20.1)	20.3 (18.6, 21.9)	18.4 (17.2, 19.7)	15.2 (12.3, 18.2)	17.2 (15.9, 18.6)	20.1 (19.0, 21.2)	21.9 (20.8, 23.0)
urban	17.4 (15.7, 19.1)	14.3 (11.1, 17.4)	16.2 (14.5, 18.0)	19.1 (17.5, 20.7)	20.8 (19.1, 22.4)	19.1 (17.8, 20.5)	16.0 (13.0, 19.0)	18.0 (16.6, 19.4)	20.8 (19.7, 22.0)	22.6 (21.4, 23.7)
urban-rural difference	0.6 (-0.1, 1.3)	0.7 (-0.3, 1.6)	0.6 (-0.1, 1.4)	0.6 (-0.1, 1.3)	0.5 (-0.2, 1.3)	0.7 (0.0, 1.4)	0.8 (-0.2, 1.8)	0.7 (0.0, 1.5)	0.7 (0.1, 1.3)	0.6 (0.0, 1.3)
Haiti										
rural	17.8 (15.5, 20.2)	14.5 (10.9, 18.2)	16.7 (14.4, 19.0)	19.6 (17.3, 21.9)	21.2 (18.5, 24.0)	19.6 (17.3, 21.8)	16.3 (12.7, 19.8)	18.4 (16.2, 20.7)	21.3 (19.2, 23.5)	22.9 (20.3, 25.5)
urban	18.3 (16.0, 20.7)	15.1 (11.5, 18.6)	17.2 (14.9, 19.6)	20.1 (17.8, 22.3)	21.6 (18.9, 24.4)	20.1 (18.0, 22.4)	16.9 (13.4, 20.4)	19.0 (16.8, 21.2)	21.9 (19.8, 24.0)	23.4 (20.8, 26.0)
urban-rural difference	0.5 (-0.3, 1.2)	0.6 (-0.4, 1.6)	0.5 (-0.3, 1.3)	0.5 (-0.3, 1.2)	0.4 (-0.4, 1.3)	0.6 (-0.1, 1.3)	0.7 (-0.3, 1.6)	0.6 (-0.2, 1.4)	0.6 (-0.1, 1.3)	0.5 (-0.3, 1.3)
Jamaica										
rural	16.5 (14.9, 18.1)	12.4 (9.8, 15.0)	15.3 (13.7, 16.8)	18.6 (17.1, 20.0)	20.2 (18.6, 21.7)	17.6 (16.5, 18.6)	13.5 (11.2, 15.9)	16.4 (15.2, 17.5)	19.6 (18.7, 20.6)	21.2 (20.2, 22.3)
urban	17.0 (15.4, 18.5)	13.0 (10.5, 15.5)	15.8 (14.2, 17.3)	19.0 (17.5, 20.4)	20.5 (19.0, 22.0)	18.2 (17.1, 19.2)	14.2 (12.0, 16.6)	17.0 (15.9, 18.1)	20.2 (19.3, 21.1)	21.8 (20.7, 22.8)
urban-rural difference	0.5 (-0.2, 1.1)	0.6 (-0.4, 1.5)	0.5 (-0.2, 1.2)	0.4 (-0.2, 1.0)	0.4 (-0.3, 1.0)	0.6 (0.0, 1.2)	0.7 (-0.2, 1.7)	0.6 (0.0, 1.3)	0.6 (0.0, 1.1)	0.5 (-0.1, 1.1)
Puerto Rico										
rural	17.8 (15.4, 20.1)	14.5 (10.9, 18.1)	16.7 (14.3, 19.0)	19.6 (17.3, 21.9)	21.2 (18.4, 23.9)	19.6 (17.3, 21.8)	16.3 (12.7, 19.7)	18.4 (16.2, 20.7)	21.3 (19.2, 23.5)	22.9 (20.3, 25.5)
urban	18.3 (15.9, 20.7)	15.1 (11.5, 18.7)	17.2 (14.9, 19.6)	20.1 (17.8, 22.3)	21.6 (18.9, 24.3)	20.2 (17.9, 22.4)	16.9 (13.4, 20.4)	19.1 (16.8, 21.4)	21.9 (19.7, 24.1)	23.4 (20.8, 26.0)
urban-rural difference	0.5 (-0.2, 1.3)	0.6 (-0.4, 1.6)	0.5 (-0.3, 1.3)	0.5 (-0.3, 1.2)	0.4 (-0.4, 1.2)	0.6 (-0.1, 1.3)	0.7 (-0.3, 1.7)	0.6 (-0.2, 1.4)	0.6 (-0.1, 1.3)	0.5 (-0.3, 1.3)
Saint Kitts and Nevis										
rural	18.5 (16.7, 20.2)	15.1 (11.9, 18.4)	17.3 (15.6, 19.1)	20.2 (18.6, 21.9)	21.9 (19.7, 24.1)	20.3 (18.7, 21.7)	16.9 (13.9, 20.0)	19.1 (17.6, 20.6)	22.0 (20.7, 23.3)	23.7 (21.7, 25.6)
urban	19.0 (17.1, 20.8)	15.7 (12.5, 19.0)	17.8 (16.0, 19.6)	20.7 (19.0, 22.3)	22.3 (20.1, 24.5)	20.8 (19.3, 22.3)	17.6 (14.5, 20.7)	19.7 (18.1, 21.2)	22.6 (21.2, 23.9)	24.2 (22.2, 26.2)
urban-rural difference	0.5 (-0.3, 1.2)	0.6 (-0.4, 1.6)	0.5 (-0.3, 1.3)	0.5 (-0.3, 1.2)	0.4 (-0.4, 1.2)	0.6 (-0.2, 1.3)	0.6 (-0.3, 1.7)	0.6 (-0.2, 1.4)	0.6 (-0.2, 1.2)	0.5 (-0.3, 1.3)
Saint Lucia										
rural	17.8 (15.4, 20.2)	14.5 (10.9, 18.2)	16.7 (14.3, 19.0)	19.6 (17.3, 21.8)	21.2 (18.5, 23.8)	19.5 (17.2, 21.8)	16.2 (12.6, 19.8)	18.4 (16.1, 20.7)	21.3 (19.1, 23.4)	22.9 (20.2, 25.5)
urban	18.3 (16.0, 20.6)	15.1 (11.5, 18.7)	17.2 (14.9, 19.5)	20.0 (17.8, 22.3)	21.6 (18.9, 24.3)	20.1 (17.8, 22.4)	16.9 (13.3, 20.6)	19.0 (16.7, 21.3)	21.9 (19.6, 24.0)	23.4 (20.8, 26.0)
urban-rural difference	0.5 (-0.3, 1.2)	0.6 (-0.4, 1.6)	0.5 (-0.3, 1.3)	0.5 (-0.3, 1.2)	0.4 (-0.4, 1.2)	0.6 (-0.1, 1.3)	0.7 (-0.3, 1.6)	0.6 (-0.1, 1.4)	0.6 (-0.1, 1.2)	0.5 (-0.3, 1.3)
Saint Vincent and the Grenadines										
rural	18.2 (16.4, 19.8)	14.9 (11.7, 17.9)	17.0 (15.2, 18.7)	19.9 (18.2, 21.5)	21.6 (19.6, 23.5)	19.9 (18.8, 21.0)	16.6 (13.6, 19.4)	18.7 (17.6, 19.9)	21.6 (20.7, 22.5)	23.3 (21.9, 24.7)

Boys	Mean BMI in 1990 (kg/m^2)					Mean BMI in 2020 (kg/m^2)				
	Age-standardised	5 years	10 years	15 years	19 years	Age-standardised	5 years	10 years	15 years	19 years
urban	18.6 (16.9, 20.4)	15.4 (12.3, 18.5)	17.5 (15.7, 19.3)	20.3 (18.7, 22.0)	22.0 (20.1, 24.0)	20.5 (19.4, 21.6)	17.2 (14.3, 20.1)	19.3 (18.2, 20.5)	22.2 (21.3, 23.0)	23.9 (22.5, 25.3)
urban-rural difference	0.5 (-0.3, 1.3)	0.6 (-0.4, 1.5)	0.5 (-0.3, 1.3)	0.5 (-0.3, 1.2)	0.4 (-0.4, 1.3)	0.6 (-0.1, 1.3)	0.7 (-0.3, 1.6)	0.6 (-0.2, 1.4)	0.6 (-0.1, 1.2)	0.5 (-0.3, 1.3)
Suriname										
rural	17.9 (15.6, 20.2)	14.6 (11.0, 18.1)	16.8 (14.5, 19.1)	19.6 (17.4, 21.9)	21.3 (18.6, 23.9)	19.6 (17.4, 21.9)	16.3 (12.8, 19.8)	18.5 (16.3, 20.8)	21.4 (19.2, 23.6)	23.0 (20.5, 25.6)
urban	18.4 (16.1, 20.7)	15.1 (11.6, 18.7)	17.3 (15.0, 19.6)	20.1 (17.9, 22.4)	21.7 (19.0, 24.4)	20.2 (18.0, 22.5)	17.0 (13.5, 20.5)	19.1 (16.9, 21.4)	21.9 (19.9, 24.1)	23.6 (21.0, 26.1)
urban-rural difference	0.5 (-0.2, 1.3)	0.6 (-0.4, 1.6)	0.5 (-0.3, 1.3)	0.5 (-0.3, 1.2)	0.4 (-0.4, 1.2)	0.6 (-0.1, 1.3)	0.7 (-0.3, 1.7)	0.6 (-0.2, 1.4)	0.6 (-0.1, 1.3)	0.5 (-0.3, 1.3)
Trinidad and Tobago										
rural	17.1 (15.8, 18.6)	13.7 (12.3, 15.1)	16.0 (14.6, 17.4)	19.0 (17.5, 20.5)	20.6 (18.8, 22.5)	19.0 (17.7, 20.4)	15.6 (14.1, 17.0)	17.9 (16.4, 19.2)	20.9 (19.5, 22.2)	22.5 (20.8, 24.3)
urban	17.7 (16.3, 19.2)	14.3 (12.9, 15.7)	16.5 (15.2, 18.0)	19.5 (18.1, 21.0)	21.2 (19.4, 23.1)	19.7 (18.4, 21.0)	16.3 (14.8, 17.7)	18.5 (17.1, 19.9)	21.5 (20.1, 22.9)	23.2 (21.5, 24.9)
urban-rural difference	0.6 (0.0, 1.1)	0.6 (0.0, 1.2)	0.6 (0.1, 1.1)	0.6 (0.0, 1.2)	0.5 (-0.2, 1.3)	0.7 (0.0, 1.3)	0.7 (0.0, 1.4)	0.7 (0.0, 1.3)	0.7 (0.0, 1.3)	0.6 (-0.1, 1.4)
Central Latin America										
Colombia										
rural	17.0 (15.4, 18.5)	14.3 (12.7, 15.9)	15.7 (14.2, 17.3)	18.5 (17.0, 20.0)	20.5 (18.9, 22.0)	19.0 (18.1, 19.8)	16.3 (15.4, 17.1)	17.7 (16.9, 18.5)	20.5 (19.6, 21.3)	22.5 (21.6, 23.3)
urban	17.4 (15.9, 19.0)	14.9 (13.4, 16.4)	16.2 (14.7, 17.7)	18.9 (17.4, 20.4)	20.8 (19.3, 22.3)	19.4 (18.6, 20.2)	16.8 (16.0, 17.7)	18.2 (17.4, 19.0)	20.8 (20.0, 21.7)	22.8 (21.9, 23.6)
urban-rural difference	0.5 (-0.1, 1.0)	0.6 (0.0, 1.1)	0.5 (0.0, 1.0)	0.4 (-0.1, 0.9)	0.3 (-0.2, 0.9)	0.4 (0.0, 0.8)	0.6 (0.1, 1.0)	0.5 (0.1, 0.9)	0.4 (0.0, 0.8)	0.3 (-0.1, 0.7)
Costa Rica										
rural	17.8 (16.1, 19.6)	14.8 (11.9, 17.8)	16.6 (14.9, 18.4)	19.4 (17.8, 21.1)	21.5 (19.6, 23.5)	19.4 (18.0, 20.8)	16.4 (13.6, 19.2)	18.2 (16.8, 19.6)	21.0 (19.8, 22.2)	23.1 (21.5, 24.6)
urban	18.3 (16.6, 20.1)	15.3 (12.3, 18.2)	17.1 (15.4, 18.8)	19.9 (18.3, 21.6)	22.0 (20.1, 23.9)	19.8 (18.5, 21.1)	16.8 (13.9, 19.5)	18.6 (17.3, 19.9)	21.4 (20.3, 22.6)	23.5 (22.1, 24.9)
urban-rural difference	0.5 (-0.3, 1.2)	0.4 (-0.5, 1.4)	0.5 (-0.3, 1.2)	0.5 (-0.2, 1.2)	0.5 (-0.3, 1.3)	0.4 (-0.3, 1.1)	0.4 (-0.6, 1.3)	0.4 (-0.3, 1.1)	0.4 (-0.2, 1.1)	0.4 (-0.3, 1.2)
El Salvador										
rural	17.8 (15.4, 20.3)	14.7 (11.1, 18.2)	16.7 (14.2, 19.0)	19.5 (17.2, 21.8)	21.3 (18.7, 24.1)	19.6 (17.3, 21.9)	16.4 (13.0, 19.9)	18.4 (16.2, 20.7)	21.3 (19.1, 23.4)	23.1 (20.5, 25.7)
urban	18.3 (15.9, 20.7)	15.2 (11.6, 18.7)	17.2 (14.7, 19.5)	20.0 (17.7, 22.3)	21.8 (19.2, 24.6)	20.1 (17.8, 22.3)	16.9 (13.5, 20.2)	18.9 (16.7, 21.1)	21.7 (19.6, 23.9)	23.6 (20.9, 26.2)
urban-rural difference	0.5 (-0.2, 1.2)	0.5 (-0.5, 1.4)	0.5 (-0.3, 1.3)	0.5 (-0.2, 1.2)	0.5 (-0.3, 1.3)	0.5 (-0.2, 1.1)	0.4 (-0.5, 1.4)	0.5 (-0.3, 1.2)	0.5 (-0.2, 1.1)	0.5 (-0.3, 1.3)
Guatemala										
rural	17.7 (16.2, 19.2)	14.4 (12.8, 16.0)	16.6 (15.1, 18.2)	19.4 (17.9, 20.9)	21.0 (19.4, 22.5)	18.8 (18.3, 19.4)	15.5 (14.8, 16.3)	17.8 (17.2, 18.4)	20.5 (19.9, 21.2)	22.1 (21.3, 22.9)
urban	18.2 (16.7, 19.7)	14.8 (13.2, 16.4)	17.1 (15.6, 18.6)	19.9 (18.4, 21.4)	21.5 (20.0, 23.1)	19.3 (18.7, 19.9)	15.9 (15.1, 16.7)	18.2 (17.5, 18.9)	21.0 (20.4, 21.7)	22.6 (21.8, 23.4)
urban-rural difference	0.5 (-0.2, 1.1)	0.4 (-0.5, 1.3)	0.5 (-0.3, 1.2)	0.5 (-0.1, 1.1)	0.6 (-0.1, 1.2)	0.4 (-0.1, 1.0)	0.4 (-0.5, 1.2)	0.4 (-0.2, 1.1)	0.5 (-0.1, 1.0)	0.5 (0.0, 1.1)
Honduras										
rural	17.8 (15.5, 20.2)	14.7 (11.3, 18.0)	16.7 (14.3, 19.0)	19.5 (17.3, 21.8)	21.3 (18.7, 24.1)	19.6 (17.3, 21.9)	16.4 (13.1, 19.8)	18.4 (16.1, 20.7)	21.3 (19.1, 23.5)	23.1 (20.5, 25.7)
urban	18.3 (16.0, 20.7)	15.1 (11.8, 18.5)	17.2 (14.8, 19.5)	20.0 (17.8, 22.3)	21.9 (19.2, 24.6)	20.1 (17.8, 22.3)	16.9 (13.6, 20.2)	18.9 (16.6, 21.2)	21.7 (19.6, 23.9)	23.6 (21.0, 26.2)
urban-rural difference	0.5 (-0.2, 1.3)	0.5 (-0.5, 1.5)	0.5 (-0.3, 1.3)	0.5 (-0.2, 1.2)	0.5 (-0.2, 1.3)	0.5 (-0.2, 1.1)	0.4 (-0.5, 1.4)	0.5 (-0.3, 1.2)	0.5 (-0.2, 1.1)	0.5 (-0.3, 1.2)
Mexico										
rural	18.3 (16.9, 19.6)	14.5 (13.2, 15.9)	17.1 (15.7, 18.4)	20.2 (18.9, 21.5)	22.1 (20.7, 23.4)	20.0 (19.4, 20.5)	16.2 (15.6, 16.8)	18.8 (18.2, 19.3)	21.9 (21.3, 22.4)	23.8 (23.1, 24.4)
urban	19.0 (17.6, 20.3)	15.1 (13.7, 16.4)	17.7 (16.4, 19.1)	20.9 (19.6, 22.3)	22.9 (21.6, 24.3)	20.6 (20.0, 21.1)	16.7 (16.1, 17.3)	19.3 (18.8, 19.9)	22.5 (22.0, 23.1)	24.5 (23.9, 25.2)
urban-rural difference	0.7 (0.2, 1.2)	0.5 (0.0, 1.0)	0.6 (0.2, 1.1)	0.8 (0.3, 1.3)	0.9 (0.4, 1.4)	0.6 (0.3, 1.0)	0.5 (0.1, 0.8)	0.6 (0.2, 0.9)	0.7 (0.3, 1.0)	0.8 (0.4, 1.2)
Nicaragua										
rural	17.8 (15.3, 20.2)	14.6 (11.1, 18.2)	16.6 (14.1, 19.0)	19.5 (17.1, 21.8)	21.3 (18.5, 24.0)	19.6 (17.3, 21.9)	16.4 (12.9, 19.9)	18.4 (16.1, 20.7)	21.2 (19.0, 23.5)	23.0 (20.4, 25.8)
urban	18.3 (15.8, 20.7)	15.1 (11.6, 18.7)	17.1 (14.7, 19.5)	20.0 (17.6, 22.3)	21.8 (19.0, 24.6)	20.0 (17.7, 22.3)	16.8 (13.4, 20.3)	18.9 (16.6, 21.1)	21.7 (19.5, 23.9)	23.5 (20.8, 26.2)
urban-rural difference	0.5 (-0.2, 1.2)	0.5 (-0.5, 1.4)	0.5 (-0.2, 1.3)	0.5 (-0.2, 1.2)	0.5 (-0.3, 1.3)	0.5 (-0.2, 1.1)	0.4 (-0.5, 1.4)	0.5 (-0.3, 1.2)	0.5 (-0.2, 1.1)	0.5 (-0.3, 1.2)
Panama										
rural	18.0 (16.5, 19.5)	15.1 (13.5, 16.6)	16.8 (15.3, 18.3)	19.6 (18.2, 21.1)	21.4 (19.8, 23.0)	19.8 (19.1, 20.4)	16.8 (16.1, 17.6)	18.6 (17.9, 19.3)	21.4 (20.8, 22.1)	23.2 (22.4, 24.0)
urban	18.4 (16.9, 19.9)	15.3 (13.8, 16.9)	17.1 (15.7, 18.6)	20.1 (18.6, 21.6)	21.9 (20.4, 23.5)	20.1 (19.4, 20.7)	17.0 (16.2, 17.8)	18.8 (18.1, 19.5)	21.8 (21.1, 22.4)	23.6 (22.8, 24.3)
urban-rural difference	0.4 (-0.2, 1.0)	0.3 (-0.4, 0.9)	0.3 (-0.2, 1.0)	0.4 (-0.1, 1.0)	0.5 (-0.1, 1.2)	0.3 (-0.3, 0.8)	0.2 (-0.5, 0.8)	0.2 (-0.3, 0.8)	0.3 (-0.2, 0.9)	0.4 (-0.2, 1.0)
Venezuela										
rural	18.1 (16.3, 19.9)	15.0 (12.3, 17.6)	17.0 (15.1, 18.7)	19.8 (18.0, 21.5)	21.8 (19.9, 23.7)	20.1 (18.7, 21.4)	16.9 (14.5, 19.3)	18.9 (17.5, 20.3)	21.7 (20.4, 23.1)	23.7 (22.2, 25.1)
urban	18.7 (16.9, 20.3)	15.5 (12.9, 18.1)	17.5 (15.7, 19.1)	20.3 (18.6, 22.0)	22.3 (20.5, 24.1)	20.5 (19.3, 21.8)	17.4 (15.1, 19.7)	19.3 (18.0, 20.6)	22.2 (21.0, 23.4)	24.2 (22.8, 25.5)
urban-rural difference	0.5 (-0.2, 1.2)	0.5 (-0.4, 1.4)	0.5 (-0.2, 1.3)	0.5 (-0.2, 1.2)	0.5 (-0.2, 1.3)	0.5 (-0.2, 1.1)	0.5 (-0.5, 1.3)	0.5 (-0.2, 1.2)	0.5 (-0.2, 1.1)	0.5 (-0.3, 1.2)
Southern Latin America										
Argentina										
rural	18.3 (16.8, 19.8)	14.3 (12.6, 15.9)	17.6 (16.0, 19.1)	20.1 (18.5, 21.6)	21.5 (19.8, 23.2)	20.1 (19.2, 21.0)	16.1 (15.0, 17.1)	19.4 (18.4, 20.3)	21.9 (21.0, 22.8)	23.3 (22.2, 24.4)
urban	18.7 (17.2, 20.2)	14.7 (13.2, 16.2)	18.0 (16.5, 19.4)	20.5 (19.0, 21.9)	21.8 (20.2, 23.4)	20.6 (20.0, 21.3)	16.6 (15.8, 17.4)	19.9 (19.2, 20.6)	22.4 (21.7, 23.0)	23.8 (22.9, 24.6)

Boys	Mean BMI in 1990 (kg/m^2)					Mean BMI in 2020 (kg/m^2)				
	Age-standardised	5 years	10 years	15 years	19 years	Age-standardised	5 years	10 years	15 years	19 years
urban-rural difference	0.4 (-0.3, 1.1)	0.4 (-0.4, 1.3)	0.4 (-0.3, 1.1)	0.4 (-0.3, 1.1)	0.3 (-0.4, 1.1)	0.5 (-0.1, 1.2)	0.6 (-0.3, 1.4)	0.5 (-0.2, 1.2)	0.5 (-0.1, 1.1)	0.5 (-0.2, 1.2)
Brazil										
rural	17.7 (17.1, 18.2)	14.9 (14.4, 15.5)	16.5 (16.0, 17.1)	19.1 (18.6, 19.7)	21.0 (20.5, 21.6)	19.3 (18.9, 19.6)	16.5 (16.1, 17.0)	18.1 (17.7, 18.5)	20.7 (20.3, 21.1)	22.6 (22.2, 23.0)
urban	18.2 (17.7, 18.7)	15.5 (15.0, 16.1)	17.1 (16.5, 17.6)	19.7 (19.2, 20.2)	21.5 (21.0, 22.1)	20.0 (19.7, 20.3)	17.3 (17.0, 17.7)	18.9 (18.5, 19.2)	21.5 (21.1, 21.8)	23.3 (23.0, 23.7)
urban-rural difference	0.6 (0.2, 0.9)	0.6 (0.2, 0.9)	0.6 (0.2, 0.9)	0.5 (0.2, 0.9)	0.5 (0.1, 0.9)	0.8 (0.5, 1.0)	0.8 (0.5, 1.1)	0.8 (0.5, 1.0)	0.7 (0.5, 1.0)	0.7 (0.5, 1.0)
Chile										
rural	19.2 (17.8, 20.6)	15.4 (13.9, 17.0)	18.2 (16.8, 19.7)	20.8 (19.4, 22.2)	23.3 (21.9, 24.7)	21.3 (20.2, 22.2)	17.5 (16.2, 18.8)	20.3 (19.2, 21.3)	22.9 (21.9, 23.9)	25.4 (24.3, 26.4)
urban	19.4 (18.0, 20.8)	15.9 (14.4, 17.5)	18.5 (17.1, 19.9)	20.9 (19.5, 22.3)	23.2 (21.8, 24.6)	21.5 (20.5, 22.4)	18.1 (16.8, 19.2)	20.6 (19.6, 21.5)	23.0 (22.1, 23.9)	25.3 (24.3, 26.3)
urban-rural difference	0.2 (-0.4, 0.8)	0.5 (-0.4, 1.3)	0.3 (-0.4, 0.9)	0.0 (-0.5, 0.7)	-0.1 (-0.8, 0.5)	0.2 (-0.3, 0.8)	0.6 (-0.2, 1.3)	0.3 (-0.2, 0.9)	0.1 (-0.4, 0.6)	-0.1 (-0.6, 0.5)
Paraguay										
rural	17.5 (15.9, 19.1)	14.1 (11.2, 17.1)	16.4 (14.9, 18.1)	19.1 (17.6, 20.5)	21.0 (19.3, 22.6)	19.2 (18.2, 20.3)	15.9 (13.2, 18.6)	18.2 (17.2, 19.3)	20.8 (20.0, 21.7)	22.8 (21.6, 23.9)
urban	18.0 (16.4, 19.6)	14.7 (11.8, 17.7)	17.0 (15.5, 18.6)	19.5 (18.1, 21.0)	21.4 (19.7, 23.1)	20.0 (18.9, 21.0)	16.7 (14.1, 19.3)	19.0 (17.9, 20.0)	21.5 (20.7, 22.3)	23.4 (22.2, 24.6)
urban-rural difference	0.5 (-0.1, 1.2)	0.6 (-0.3, 1.5)	0.6 (-0.2, 1.3)	0.5 (-0.2, 1.1)	0.4 (-0.3, 1.1)	0.7 (0.2, 1.3)	0.8 (0.0, 1.7)	0.7 (0.1, 1.4)	0.7 (0.2, 1.2)	0.6 (0.0, 1.2)
Uruguay										
rural	18.2 (16.7, 19.7)	15.0 (13.2, 16.7)	17.2 (15.7, 18.8)	19.9 (18.3, 21.4)	21.7 (19.9, 23.5)	20.2 (19.3, 21.1)	16.9 (15.6, 18.3)	19.2 (18.2, 20.2)	21.8 (20.9, 22.7)	23.6 (22.3, 24.9)
urban	18.7 (17.2, 20.1)	15.5 (13.9, 17.0)	17.7 (16.2, 19.1)	20.3 (18.8, 21.7)	22.0 (20.3, 23.7)	20.8 (20.1, 21.4)	17.5 (16.5, 18.6)	19.8 (19.0, 20.5)	22.4 (21.7, 23.0)	24.1 (23.0, 25.2)
urban-rural difference	0.4 (-0.3, 1.1)	0.5 (-0.4, 1.4)	0.4 (-0.3, 1.2)	0.4 (-0.3, 1.1)	0.3 (-0.4, 1.1)	0.6 (-0.1, 1.2)	0.6 (-0.3, 1.6)	0.6 (-0.1, 1.3)	0.5 (-0.1, 1.2)	0.5 (-0.2, 1.2)
**Oceania**										
Melanesia										
Fiji										
rural	17.2 (15.1, 19.3)	14.7 (11.1, 18.5)	15.7 (13.6, 17.8)	18.6 (16.7, 20.5)	21.6 (19.6, 23.6)	20.1 (18.8, 21.4)	17.6 (14.4, 20.8)	18.6 (17.2, 19.9)	21.5 (20.4, 22.5)	24.4 (23.3, 25.7)
urban	17.6 (15.5, 19.7)	15.2 (11.6, 19.0)	16.1 (14.0, 18.2)	18.9 (17.0, 20.9)	21.9 (19.9, 23.9)	20.5 (19.2, 21.8)	18.1 (14.9, 21.4)	19.0 (17.7, 20.3)	21.8 (20.8, 22.9)	24.8 (23.6, 25.9)
urban-rural difference	0.4 (-0.4, 1.1)	0.5 (-0.6, 1.5)	0.4 (-0.4, 1.2)	0.3 (-0.4, 1.1)	0.3 (-0.5, 1.0)	0.4 (-0.4, 1.2)	0.5 (-0.5, 1.6)	0.4 (-0.4, 1.3)	0.4 (-0.4, 1.1)	0.3 (-0.5, 1.2)
Papua New Guinea										
rural	18.8 (16.5, 21.1)	16.2 (12.4, 20.0)	17.5 (15.1, 19.8)	20.4 (18.3, 22.6)	22.4 (20.2, 24.6)	21.7 (19.8, 23.5)	19.0 (15.3, 22.5)	20.3 (18.4, 22.2)	23.2 (21.6, 24.9)	25.2 (23.4, 26.9)
urban	19.1 (16.8, 21.5)	16.5 (12.7, 20.4)	17.8 (15.4, 20.1)	20.7 (18.5, 22.9)	22.6 (20.3, 24.8)	22.0 (20.1, 23.9)	19.4 (15.8, 22.9)	20.6 (18.7, 22.6)	23.6 (21.8, 25.3)	25.5 (23.6, 27.3)
urban-rural difference	0.3 (-0.6, 1.1)	0.3 (-0.8, 1.4)	0.3 (-0.6, 1.2)	0.3 (-0.6, 1.1)	0.2 (-0.7, 1.2)	0.3 (-0.5, 1.2)	0.4 (-0.7, 1.5)	0.3 (-0.6, 1.2)	0.3 (-0.5, 1.1)	0.3 (-0.6, 1.2)
Solomon Islands										
rural	17.1 (15.0, 19.3)	14.8 (12.7, 17.1)	15.3 (13.2, 17.5)	18.5 (16.4, 20.7)	21.7 (19.6, 23.9)	20.0 (18.5, 21.4)	17.7 (16.2, 19.3)	18.2 (16.7, 19.7)	21.4 (19.9, 22.9)	24.6 (23.1, 26.0)
urban	17.3 (15.1, 19.4)	15.2 (13.0, 17.5)	15.5 (13.4, 17.7)	18.6 (16.4, 20.8)	21.6 (19.4, 23.8)	20.2 (18.7, 21.7)	18.2 (16.6, 19.8)	18.5 (17.0, 20.0)	21.5 (20.0, 23.1)	24.6 (23.0, 26.1)
urban-rural difference	0.2 (-0.6, 0.9)	0.4 (-0.5, 1.3)	0.2 (-0.6, 1.0)	0.1 (-0.7, 0.8)	-0.1 (-0.9, 0.7)	0.2 (-0.5, 0.9)	0.4 (-0.4, 1.3)	0.3 (-0.5, 1.0)	0.1 (-0.6, 0.8)	0.0 (-0.8, 0.7)
Vanuatu										
rural	18.2 (15.8, 20.5)	15.5 (11.6, 19.4)	16.8 (14.3, 19.1)	19.7 (17.5, 22.0)	21.8 (19.5, 24.1)	20.9 (18.9, 23.0)	18.3 (14.6, 22.0)	19.6 (17.5, 21.7)	22.5 (20.6, 24.4)	24.6 (22.6, 26.6)
urban	18.5 (16.1, 20.9)	15.9 (12.0, 19.8)	17.1 (14.6, 19.5)	20.0 (17.7, 22.3)	22.0 (19.7, 24.4)	21.3 (19.2, 23.4)	18.7 (15.0, 22.4)	19.9 (17.8, 22.1)	22.8 (20.9, 24.8)	24.9 (22.8, 27.0)
urban-rural difference	0.3 (-0.6, 1.2)	0.4 (-0.8, 1.5)	0.3 (-0.6, 1.2)	0.3 (-0.6, 1.1)	0.3 (-0.7, 1.2)	0.4 (-0.5, 1.2)	0.4 (-0.7, 1.5)	0.4 (-0.5, 1.3)	0.3 (-0.5, 1.2)	0.3 (-0.6, 1.2)
Polynesia and Micronesia										
American Samoa										
rural	19.9 (17.1, 22.7)	17.3 (12.8, 21.7)	18.4 (15.6, 21.2)	21.4 (18.7, 24.0)	24.3 (21.2, 27.3)	22.7 (20.3, 25.1)	20.1 (15.8, 24.2)	21.1 (18.7, 23.4)	24.2 (21.9, 26.4)	27.1 (24.4, 29.7)
urban	20.3 (17.5, 23.1)	17.6 (13.1, 22.0)	18.7 (15.9, 21.5)	21.8 (19.1, 24.4)	24.7 (21.6, 27.8)	23.1 (20.7, 25.4)	20.5 (16.2, 24.6)	21.5 (19.2, 23.9)	24.6 (22.4, 26.8)	27.5 (24.9, 30.3)
urban-rural difference	0.4 (-0.5, 1.3)	0.3 (-0.8, 1.5)	0.3 (-0.6, 1.3)	0.4 (-0.5, 1.3)	0.4 (-0.5, 1.4)	0.4 (-0.4, 1.3)	0.4 (-0.7, 1.6)	0.4 (-0.5, 1.3)	0.5 (-0.4, 1.3)	0.5 (-0.5, 1.4)
Cook Islands										
rural	21.5 (19.1, 23.8)	18.7 (14.5, 22.8)	19.8 (17.4, 22.1)	23.1 (20.9, 25.2)	26.5 (23.9, 29.0)	24.6 (23.1, 26.1)	21.8 (18.1, 25.6)	22.9 (21.4, 24.4)	26.2 (25.0, 27.4)	29.6 (27.9, 31.2)
urban	21.9 (19.5, 24.2)	19.0 (14.8, 23.1)	20.1 (17.8, 22.4)	23.5 (21.3, 25.6)	26.9 (24.5, 29.4)	25.1 (23.7, 26.5)	22.2 (18.5, 26.0)	23.3 (21.9, 24.7)	26.7 (25.5, 27.8)	30.1 (28.5, 31.7)
urban-rural difference	0.4 (-0.5, 1.3)	0.3 (-0.8, 1.5)	0.4 (-0.6, 1.3)	0.4 (-0.4, 1.3)	0.4 (-0.5, 1.4)	0.5 (-0.4, 1.3)	0.4 (-0.7, 1.6)	0.5 (-0.4, 1.4)	0.5 (-0.3, 1.3)	0.5 (-0.4, 1.5)
French Polynesia										
rural	20.2 (17.8, 22.4)	17.9 (13.9, 21.9)	18.7 (16.3, 20.9)	21.5 (19.3, 23.6)	24.3 (21.9, 26.7)	22.8 (21.5, 24.2)	20.6 (17.0, 24.2)	21.3 (20.0, 22.7)	24.1 (23.0, 25.2)	27.0 (25.4, 28.6)
urban	20.5 (18.2, 22.7)	18.3 (14.2, 22.3)	19.0 (16.7, 21.2)	21.8 (19.6, 24.0)	24.7 (22.3, 27.0)	23.3 (21.9, 24.6)	21.0 (17.4, 24.6)	21.8 (20.4, 23.0)	24.6 (23.5, 25.7)	27.5 (25.9, 29.0)
urban-rural difference	0.4 (-0.5, 1.3)	0.3 (-0.8, 1.5)	0.4 (-0.6, 1.3)	0.4 (-0.5, 1.3)	0.4 (-0.6, 1.4)	0.4 (-0.4, 1.3)	0.4 (-0.7, 1.6)	0.4 (-0.5, 1.4)	0.5 (-0.4, 1.3)	0.5 (-0.5, 1.4)
Kiribati										
rural	19.4 (17.2, 21.6)	16.8 (12.7, 21.0)	17.9 (15.6, 20.2)	20.9 (18.9, 23.0)	23.8 (21.6, 25.8)	22.1 (20.4, 23.7)	19.4 (15.7, 23.3)	20.5 (18.8, 22.2)	23.5 (22.0, 25.0)	26.4 (24.9, 27.9)
urban	19.8 (17.6, 22.0)	17.2 (13.1, 21.4)	18.3 (16.0, 20.6)	21.3 (19.2, 23.4)	24.2 (22.0, 26.3)	22.5 (20.8, 24.2)	19.9 (16.1, 23.9)	21.0 (19.1, 22.7)	24.0 (22.5, 25.5)	26.8 (25.3, 28.4)

Boys	Mean BMI in 1990 (kg/m²) Age-standardised	5 years	10 years	15 years	19 years	Mean BMI in 2020 (kg/m²) Age-standardised	5 years	10 years	15 years	19 years
urban-rural difference	0.4 (-0.5, 1.3)	0.3 (-0.8, 1.4)	0.4 (-0.6, 1.3)	0.4 (-0.5, 1.3)	0.4 (-0.5, 1.4)	0.4 (-0.4, 1.3)	0.4 (-0.7, 1.6)	0.4 (-0.5, 1.4)	0.5 (-0.4, 1.3)	0.5 (-0.4, 1.4)
Marshall Islands										
rural	17.8 (15.5, 20.0)	15.2 (11.0, 19.3)	16.3 (14.0, 18.5)	19.3 (17.2, 21.3)	21.8 (19.6, 23.9)	20.5 (18.8, 22.2)	17.9 (14.0, 21.8)	19.1 (17.3, 20.8)	22.0 (20.6, 23.5)	24.5 (23.0, 26.0)
urban	18.1 (15.9, 20.3)	15.5 (11.3, 19.6)	16.7 (14.4, 18.9)	19.6 (17.7, 21.6)	22.1 (20.1, 24.1)	20.9 (19.3, 22.6)	18.3 (14.3, 22.2)	19.5 (17.8, 21.2)	22.4 (21.0, 23.9)	24.9 (23.6, 26.2)
urban-rural difference	0.3 (-0.5, 1.2)	0.3 (-0.8, 1.5)	0.3 (-0.6, 1.2)	0.3 (-0.5, 1.2)	0.3 (-0.6, 1.3)	0.4 (-0.5, 1.3)	0.4 (-0.8, 1.5)	0.4 (-0.5, 1.3)	0.4 (-0.4, 1.2)	0.4 (-0.5, 1.3)
Micronesia (Federated States of)										
rural	18.4 (16.0, 20.6)	15.6 (11.3, 19.8)	17.1 (14.7, 19.4)	20.1 (17.9, 22.1)	21.5 (19.2, 23.7)	20.9 (19.2, 22.6)	18.1 (14.1, 22.1)	19.6 (17.9, 21.3)	22.6 (21.1, 24.0)	24.0 (22.5, 25.5)
urban	18.9 (16.5, 21.1)	16.0 (11.7, 20.3)	17.6 (15.2, 19.8)	20.6 (18.4, 22.7)	22.1 (19.8, 24.3)	21.4 (19.7, 23.2)	18.6 (14.6, 22.6)	20.1 (18.3, 21.9)	23.2 (21.6, 24.7)	24.7 (23.1, 26.2)
urban-rural difference	0.5 (-0.3, 1.3)	0.4 (-0.8, 1.5)	0.5 (-0.4, 1.3)	0.5 (-0.3, 1.3)	0.6 (-0.3, 1.5)	0.6 (-0.3, 1.4)	0.5 (-0.7, 1.6)	0.5 (-0.3, 1.4)	0.6 (-0.2, 1.4)	0.7 (-0.2, 1.5)
Nauru										
rural	21.3 (18.8, 23.6)	18.7 (14.5, 23.0)	19.6 (17.1, 21.9)	22.7 (20.4, 24.9)	26.1 (23.7, 28.4)	24.2 (22.3, 26.0)	21.6 (17.8, 25.7)	22.5 (20.6, 24.4)	25.6 (23.9, 27.3)	29.0 (27.2, 30.8)
urban	21.7 (19.4, 24.0)	19.0 (14.9, 23.4)	20.0 (17.7, 22.3)	23.1 (21.0, 25.3)	26.6 (24.3, 28.8)	24.7 (22.8, 26.5)	22.0 (18.2, 26.1)	23.0 (21.1, 24.8)	26.1 (24.5, 27.6)	29.6 (27.9, 31.2)
urban-rural difference	0.4 (-0.4, 1.3)	0.4 (-0.8, 1.5)	0.4 (-0.5, 1.3)	0.4 (-0.4, 1.3)	0.5 (-0.5, 1.4)	0.5 (-0.3, 1.4)	0.4 (-0.7, 1.6)	0.5 (-0.4, 1.4)	0.5 (-0.3, 1.4)	0.6 (-0.4, 1.5)
Niue										
rural	21.5 (19.1, 23.7)	18.9 (14.8, 23.1)	19.9 (17.5, 22.1)	23.0 (20.7, 25.1)	25.9 (23.3, 28.3)	24.5 (23.2, 25.8)	21.9 (18.3, 25.6)	22.9 (21.6, 24.2)	26.0 (24.9, 27.1)	28.9 (27.3, 30.4)
urban	21.9 (19.5, 24.1)	19.2 (15.1, 23.5)	20.3 (17.9, 22.5)	23.4 (21.1, 25.5)	26.3 (23.8, 28.7)	24.9 (23.5, 26.3)	22.3 (18.7, 26.1)	23.3 (22.0, 24.7)	26.4 (25.3, 27.6)	29.3 (27.8, 31.0)
urban-rural difference	0.4 (-0.5, 1.2)	0.3 (-0.8, 1.5)	0.3 (-0.6, 1.3)	0.4 (-0.5, 1.2)	0.4 (-0.6, 1.3)	0.4 (-0.4, 1.3)	0.4 (-0.8, 1.5)	0.4 (-0.5, 1.3)	0.4 (-0.4, 1.3)	0.5 (-0.5, 1.4)
Palau										
rural	20.1 (17.5, 22.8)	17.5 (13.3, 22.1)	18.6 (15.9, 21.3)	21.6 (19.1, 24.1)	24.6 (21.8, 27.5)	22.9 (20.8, 25.1)	20.3 (16.4, 24.5)	21.3 (19.2, 23.6)	24.4 (22.4, 26.4)	27.4 (25.2, 29.7)
urban	20.5 (17.8, 23.2)	17.9 (13.5, 22.3)	18.9 (16.3, 21.6)	22.0 (19.5, 24.5)	25.0 (22.2, 27.8)	23.4 (21.2, 25.5)	20.7 (16.7, 24.9)	21.8 (19.6, 23.9)	24.9 (22.8, 26.8)	27.9 (25.7, 30.2)
urban-rural difference	0.4 (-0.5, 1.3)	0.3 (-0.8, 1.5)	0.3 (-0.6, 1.3)	0.4 (-0.5, 1.2)	0.4 (-0.5, 1.3)	0.4 (-0.4, 1.3)	0.4 (-0.7, 1.5)	0.4 (-0.5, 1.3)	0.5 (-0.4, 1.3)	0.5 (-0.5, 1.4)
Samoa										
rural	19.2 (16.7, 21.7)	16.6 (12.4, 21.0)	17.7 (15.1, 20.1)	20.7 (18.3, 22.9)	23.6 (21.1, 25.9)	21.9 (20.1, 23.7)	19.3 (15.4, 23.2)	20.3 (18.5, 22.2)	23.4 (21.8, 24.9)	26.3 (24.7, 27.9)
urban	19.6 (17.1, 22.0)	17.0 (12.7, 21.3)	18.1 (15.5, 20.5)	21.1 (18.8, 23.4)	24.1 (21.6, 26.4)	22.3 (20.6, 24.2)	19.7 (16.0, 23.7)	20.8 (18.9, 22.7)	23.8 (22.2, 25.5)	26.8 (25.0, 28.5)
urban-rural difference	0.4 (-0.5, 1.3)	0.3 (-0.8, 1.5)	0.4 (-0.5, 1.3)	0.4 (-0.4, 1.3)	0.4 (-0.5, 1.4)	0.5 (-0.4, 1.3)	0.4 (-0.7, 1.6)	0.5 (-0.5, 1.4)	0.5 (-0.4, 1.3)	0.5 (-0.4, 1.5)
Tokelau										
rural	21.0 (18.5, 23.3)	18.4 (14.1, 22.5)	19.3 (16.8, 21.7)	22.4 (20.1, 24.6)	25.6 (23.1, 28.1)	23.8 (21.8, 25.7)	21.2 (17.3, 25.1)	22.1 (20.2, 24.1)	25.2 (23.5, 26.9)	28.4 (26.3, 30.5)
urban	21.3 (18.7, 23.7)	18.7 (14.4, 23.0)	19.6 (17.1, 22.1)	22.8 (20.3, 25.0)	26.0 (23.3, 28.5)	24.2 (22.2, 26.2)	21.5 (17.6, 25.5)	22.5 (20.6, 24.5)	25.6 (23.8, 27.5)	28.8 (26.7, 31.0)
urban-rural difference	0.3 (-0.6, 1.2)	0.3 (-0.8, 1.4)	0.3 (-0.6, 1.2)	0.3 (-0.5, 1.2)	0.3 (-0.6, 1.3)	0.4 (-0.5, 1.3)	0.4 (-0.8, 1.5)	0.4 (-0.5, 1.3)	0.4 (-0.5, 1.2)	0.4 (-0.5, 1.4)
Tonga										
rural	20.4 (18.2, 22.5)	17.7 (14.0, 21.6)	18.7 (16.4, 20.8)	21.9 (19.8, 23.8)	25.3 (23.1, 27.4)	23.3 (22.2, 24.4)	20.7 (17.5, 23.9)	21.6 (20.6, 22.7)	24.8 (24.0, 25.7)	28.2 (27.2, 29.3)
urban	20.8 (18.5, 22.9)	18.1 (14.3, 22.1)	19.1 (16.9, 21.3)	22.3 (20.2, 24.3)	25.7 (23.5, 27.9)	23.8 (22.6, 25.1)	21.1 (17.8, 24.6)	22.1 (20.9, 23.3)	25.3 (24.3, 26.3)	28.7 (27.5, 30.0)
urban-rural difference	0.4 (-0.4, 1.3)	0.4 (-0.7, 1.6)	0.4 (-0.5, 1.3)	0.4 (-0.4, 1.3)	0.4 (-0.5, 1.4)	0.5 (-0.4, 1.4)	0.5 (-0.6, 1.6)	0.5 (-0.4, 1.4)	0.5 (-0.4, 1.4)	0.5 (-0.4, 1.5)
Tuvalu										
rural	20.4 (17.9, 22.7)	17.7 (13.5, 21.9)	18.6 (16.2, 21.0)	21.8 (19.5, 24.0)	25.4 (23.0, 27.7)	23.2 (21.6, 24.7)	20.6 (16.9, 24.4)	21.5 (19.9, 23.0)	24.6 (23.3, 26.0)	28.2 (26.8, 29.7)
urban	20.7 (18.3, 23.1)	18.1 (13.8, 22.3)	19.0 (16.6, 21.4)	22.2 (19.9, 24.3)	25.8 (23.4, 28.1)	23.6 (22.1, 25.1)	21.0 (17.3, 24.8)	21.9 (20.3, 23.5)	25.1 (23.8, 26.4)	28.7 (27.3, 30.1)
urban-rural difference	0.4 (-0.5, 1.3)	0.3 (-0.8, 1.4)	0.3 (-0.6, 1.3)	0.4 (-0.5, 1.2)	0.4 (-0.6, 1.4)	0.4 (-0.4, 1.3)	0.4 (-0.7, 1.5)	0.4 (-0.5, 1.3)	0.5 (-0.4, 1.3)	0.5 (-0.4, 1.4)
**South Asia**										
Afghanistan										
rural	16.5 (14.6, 18.3)	14.0 (10.9, 17.2)	15.1 (13.2, 17.0)	17.9 (16.2, 19.7)	20.2 (18.4, 21.9)	18.7 (17.4, 20.0)	16.2 (13.3, 19.1)	17.4 (15.9, 18.8)	20.2 (18.9, 21.4)	22.4 (21.3, 23.5)
urban	17.1 (15.2, 18.9)	14.5 (11.3, 17.7)	15.7 (13.8, 17.6)	18.5 (16.8, 20.3)	20.8 (19.0, 22.5)	19.3 (17.9, 20.6)	16.7 (13.8, 19.7)	17.9 (16.4, 19.4)	20.7 (19.5, 22.0)	23.0 (21.9, 24.0)
urban-rural difference	0.6 (-0.1, 1.3)	0.6 (-0.3, 1.5)	0.6 (-0.2, 1.3)	0.6 (-0.1, 1.3)	0.6 (-0.2, 1.4)	0.5 (-0.1, 1.2)	0.5 (-0.4, 1.4)	0.5 (-0.2, 1.2)	0.6 (-0.1, 1.2)	0.6 (-0.1, 1.2)
Bangladesh										
rural	14.8 (13.3, 16.4)	11.8 (9.3, 14.2)	13.4 (11.8, 14.9)	16.5 (15.0, 18.0)	18.8 (17.2, 20.4)	16.8 (16.1, 17.5)	13.8 (11.8, 15.7)	15.4 (14.7, 16.0)	18.5 (17.9, 19.1)	20.8 (20.1, 21.5)
urban	15.5 (13.9, 17.1)	12.7 (10.2, 15.1)	14.2 (12.6, 15.7)	17.2 (15.6, 18.7)	19.4 (17.7, 21.0)	17.5 (16.8, 18.2)	14.6 (12.6, 16.6)	16.1 (15.4, 16.8)	19.1 (18.5, 19.8)	21.4 (20.6, 22.1)
urban-rural difference	0.7 (0.1, 1.4)	0.9 (0.0, 1.7)	0.8 (0.1, 1.5)	0.7 (0.0, 1.3)	0.6 (-0.1, 1.3)	0.7 (0.2, 1.2)	0.9 (0.1, 1.6)	0.7 (0.2, 1.3)	0.6 (0.2, 1.1)	0.5 (0.0, 1.1)
Bhutan										
rural	15.9 (14.1, 17.6)	13.3 (10.1, 16.3)	14.6 (12.8, 16.4)	17.4 (15.8, 19.0)	19.1 (17.5, 20.7)	18.1 (17.0, 19.2)	15.5 (12.6, 18.3)	16.8 (15.7, 18.0)	19.7 (18.8, 20.5)	21.3 (20.5, 22.2)
urban	16.5 (14.7, 18.2)	13.9 (10.8, 16.9)	15.2 (13.4, 17.0)	18.0 (16.3, 19.6)	19.6 (18.0, 21.2)	18.6 (17.5, 19.7)	16.1 (13.1, 18.9)	17.4 (16.2, 18.6)	20.2 (19.2, 21.1)	21.8 (20.8, 22.7)
urban-rural difference	0.6 (-0.1, 1.3)	0.6 (-0.3, 1.6)	0.6 (-0.2, 1.3)	0.6 (-0.1, 1.3)	0.6 (-0.2, 1.3)	0.5 (-0.1, 1.1)	0.6 (-0.4, 1.5)	0.5 (-0.2, 1.2)	0.5 (-0.1, 1.1)	0.5 (-0.2, 1.2)
India										

Boys	Mean BMI in 1990 (kg/m^2)					Mean BMI in 2020 (kg/m^2)				
	Age-standardised	5 years	10 years	15 years	19 years	Age-standardised	5 years	10 years	15 years	19 years
rural	15.2 (14.6, 15.7)	13.1 (12.5, 13.7)	14.0 (13.4, 14.5)	16.4 (15.9, 17.0)	18.4 (17.8, 18.9)	17.1 (16.6, 17.5)	15.0 (14.5, 15.5)	15.9 (15.4, 16.4)	18.4 (17.9, 18.8)	20.3 (19.8, 20.7)
urban	15.9 (15.2, 16.6)	13.5 (12.8, 14.3)	14.6 (13.9, 15.3)	17.3 (16.6, 18.0)	19.4 (18.7, 20.1)	17.7 (17.2, 18.1)	15.3 (14.8, 15.8)	16.4 (15.9, 16.8)	19.0 (18.6, 19.5)	21.1 (20.6, 21.6)
urban-rural difference	0.7 (0.4, 1.2)	0.4 (0.0, 0.9)	0.6 (0.3, 1.1)	0.8 (0.5, 1.3)	1.0 (0.6, 1.5)	0.6 (0.3, 0.8)	0.3 (0.0, 0.6)	0.5 (0.2, 0.8)	0.7 (0.4, 0.9)	0.9 (0.6, 1.1)
Nepal										
rural	15.6 (14.0, 17.2)	13.1 (10.0, 16.1)	14.3 (12.7, 15.9)	17.1 (15.6, 18.5)	18.7 (17.2, 20.2)	18.1 (17.1, 19.1)	15.6 (12.7, 18.4)	16.8 (15.8, 17.8)	19.6 (19.0, 20.3)	21.2 (20.6, 21.9)
urban	16.6 (15.0, 18.2)	13.9 (10.9, 17.0)	15.3 (13.6, 16.9)	18.2 (16.7, 19.6)	19.9 (18.3, 21.3)	19.2 (18.2, 20.1)	16.5 (13.7, 19.4)	17.9 (16.9, 18.9)	20.8 (20.2, 21.4)	22.5 (21.8, 23.1)
urban-rural difference	1.0 (0.3, 1.6)	0.9 (0.0, 1.8)	1.0 (0.3, 1.7)	1.1 (0.4, 1.6)	1.1 (0.5, 1.7)	1.1 (0.6, 1.6)	1.0 (0.2, 1.8)	1.1 (0.5, 1.6)	1.2 (0.8, 1.6)	1.2 (0.8, 1.7)
Pakistan										
rural	15.9 (15.2, 16.5)	14.1 (13.3, 14.8)	14.5 (13.8, 15.2)	17.1 (16.5, 17.8)	19.2 (18.5, 20.0)	17.9 (17.2, 18.5)	16.1 (15.3, 16.8)	16.5 (15.8, 17.1)	19.1 (18.5, 19.8)	21.2 (20.5, 22.0)
urban	16.3 (15.6, 16.9)	14.3 (13.5, 15.0)	14.8 (14.1, 15.5)	17.6 (16.9, 18.3)	19.8 (19.1, 20.6)	18.2 (17.5, 18.9)	16.2 (15.5, 17.0)	16.8 (16.1, 17.4)	19.6 (18.9, 20.2)	21.8 (21.1, 22.5)
urban-rural difference	0.4 (-0.1, 0.9)	0.2 (-0.3, 0.7)	0.3 (-0.1, 0.8)	0.5 (0.0, 0.9)	0.6 (0.1, 1.1)	0.4 (0.0, 0.7)	0.2 (-0.3, 0.6)	0.3 (-0.1, 0.7)	0.4 (0.0, 0.8)	0.5 (0.1, 1.0)
Sri Lanka										
rural	15.8 (14.2, 17.4)	13.3 (10.2, 16.4)	14.5 (12.9, 16.1)	17.2 (15.8, 18.7)	19.2 (17.6, 20.8)	18.1 (16.9, 19.2)	15.6 (12.7, 18.4)	16.8 (15.6, 17.9)	19.5 (18.6, 20.5)	21.5 (20.3, 22.6)
urban	16.4 (14.7, 18.1)	13.9 (10.7, 17.0)	15.1 (13.4, 16.8)	17.9 (16.3, 19.4)	19.8 (18.2, 21.5)	18.6 (17.4, 19.8)	16.1 (13.2, 18.9)	17.3 (16.0, 18.6)	20.1 (19.1, 21.2)	22.1 (20.8, 23.4)
urban-rural difference	0.6 (-0.1, 1.3)	0.6 (-0.4, 1.5)	0.6 (-0.1, 1.3)	0.6 (-0.1, 1.3)	0.7 (-0.1, 1.4)	0.6 (-0.1, 1.2)	0.5 (-0.4, 1.5)	0.6 (-0.2, 1.3)	0.6 (-0.1, 1.2)	0.6 (-0.1, 1.3)
**Sub-Saharan Africa**										
Central Africa										
Angola										
rural	16.2 (14.2, 18.3)	14.0 (10.6, 17.4)	15.0 (13.0, 17.0)	17.7 (15.8, 19.6)	19.1 (17.1, 21.2)	17.9 (16.2, 19.6)	15.7 (12.4, 18.9)	16.7 (14.9, 18.4)	19.3 (17.8, 20.9)	20.8 (19.1, 22.5)
urban	16.6 (14.6, 18.6)	14.4 (11.1, 17.9)	15.4 (13.4, 17.4)	18.0 (16.1, 19.9)	19.4 (17.4, 21.4)	18.1 (16.4, 19.8)	15.9 (12.7, 19.1)	16.9 (15.2, 18.6)	19.5 (18.0, 21.0)	20.9 (19.3, 22.5)
urban-rural difference	0.4 (-0.4, 1.2)	0.4 (-0.6, 1.5)	0.4 (-0.5, 1.2)	0.4 (-0.4, 1.2)	0.3 (-0.5, 1.2)	0.2 (-0.6, 0.9)	0.2 (-0.8, 1.3)	0.2 (-0.6, 1.0)	0.2 (-0.5, 0.8)	0.1 (-0.6, 0.9)
Central African Republic										
rural	16.3 (13.8, 18.8)	14.1 (10.4, 17.7)	15.1 (12.6, 17.6)	17.7 (15.4, 20.2)	19.2 (16.4, 22.1)	18.0 (15.6, 20.4)	15.8 (12.0, 19.4)	16.8 (14.3, 19.2)	19.4 (17.1, 21.8)	20.9 (18.1, 23.7)
urban	16.7 (14.3, 19.3)	14.6 (10.8, 18.2)	15.5 (13.1, 18.0)	18.1 (15.8, 20.6)	19.6 (16.8, 22.5)	18.2 (15.8, 20.7)	16.1 (12.4, 19.7)	17.0 (14.6, 19.5)	19.6 (17.4, 22.0)	21.1 (18.3, 24.0)
urban-rural difference	0.4 (-0.4, 1.2)	0.4 (-0.6, 1.5)	0.4 (-0.4, 1.3)	0.4 (-0.4, 1.2)	0.4 (-0.5, 1.3)	0.3 (-0.5, 1.0)	0.3 (-0.7, 1.4)	0.3 (-0.5, 1.1)	0.3 (-0.5, 1.0)	0.2 (-0.6, 1.0)
Congo										
rural	16.3 (15.0, 17.7)	14.5 (13.0, 16.1)	15.0 (13.6, 16.4)	17.6 (16.2, 18.9)	19.5 (18.0, 21.0)	18.0 (16.3, 19.7)	16.2 (14.3, 18.1)	16.7 (14.9, 18.4)	19.3 (17.5, 21.0)	21.2 (19.3, 22.9)
urban	16.7 (15.6, 17.9)	14.9 (13.7, 16.1)	15.4 (14.2, 16.6)	18.0 (16.8, 19.2)	19.9 (18.6, 21.1)	18.3 (16.6, 19.9)	16.4 (14.7, 18.2)	16.9 (15.3, 18.6)	19.5 (17.8, 21.2)	21.4 (19.7, 23.1)
urban-rural difference	0.4 (-0.4, 1.2)	0.4 (-0.6, 1.5)	0.4 (-0.4, 1.3)	0.4 (-0.4, 1.2)	0.4 (-0.5, 1.3)	0.2 (-0.5, 1.0)	0.2 (-0.8, 1.3)	0.2 (-0.6, 1.0)	0.2 (-0.5, 1.0)	0.2 (-0.6, 1.1)
DR Congo										
rural	15.8 (14.0, 17.6)	13.2 (10.1, 16.2)	14.6 (12.8, 16.4)	17.4 (15.7, 19.1)	18.7 (17.0, 20.5)	17.6 (16.1, 19.1)	14.9 (12.1, 17.9)	16.4 (14.8, 17.9)	19.1 (17.7, 20.5)	20.5 (19.1, 21.8)
urban	16.2 (14.5, 18.0)	13.6 (10.6, 16.6)	15.1 (13.3, 16.8)	17.8 (16.2, 19.5)	19.2 (17.4, 20.8)	17.8 (16.4, 19.3)	15.2 (12.4, 18.0)	16.6 (15.2, 18.1)	19.4 (18.1, 20.8)	20.7 (19.4, 22.1)
urban-rural difference	0.4 (-0.4, 1.2)	0.4 (-0.7, 1.5)	0.4 (-0.4, 1.3)	0.4 (-0.3, 1.2)	0.4 (-0.4, 1.3)	0.3 (-0.5, 1.0)	0.2 (-0.8, 1.3)	0.3 (-0.5, 1.0)	0.3 (-0.4, 1.0)	0.3 (-0.5, 1.0)
Equatorial Guinea										
rural	16.3 (13.9, 18.9)	14.1 (10.5, 17.8)	15.1 (12.7, 17.6)	17.7 (15.4, 20.1)	19.2 (16.5, 22.1)	18.0 (15.6, 20.4)	15.8 (12.2, 19.5)	16.8 (14.4, 19.2)	19.4 (17.1, 21.7)	20.9 (18.2, 23.7)
urban	16.7 (14.3, 19.2)	14.5 (10.9, 18.1)	15.5 (13.1, 18.0)	18.1 (15.7, 20.5)	19.7 (16.9, 22.5)	18.3 (15.9, 20.6)	16.1 (12.5, 19.6)	17.0 (14.7, 19.4)	19.7 (17.4, 21.9)	21.2 (18.5, 23.9)
urban-rural difference	0.4 (-0.4, 1.2)	0.4 (-0.6, 1.5)	0.4 (-0.4, 1.3)	0.4 (-0.4, 1.2)	0.4 (-0.5, 1.3)	0.3 (-0.5, 1.0)	0.3 (-0.8, 1.3)	0.3 (-0.6, 1.1)	0.3 (-0.5, 1.0)	0.2 (-0.6, 1.1)
Gabon										
rural	16.6 (14.5, 18.6)	14.3 (10.8, 17.7)	15.5 (13.3, 17.5)	18.1 (16.1, 20.0)	19.1 (16.9, 21.2)	18.3 (16.4, 20.2)	16.0 (12.7, 19.5)	17.2 (15.3, 19.2)	19.8 (18.1, 21.6)	20.8 (18.9, 22.7)
urban	17.1 (15.0, 19.0)	14.8 (11.3, 18.2)	15.9 (13.9, 18.0)	18.5 (16.6, 20.4)	19.5 (17.4, 21.5)	18.6 (16.7, 20.5)	16.4 (13.0, 19.7)	17.5 (15.6, 19.4)	20.1 (18.4, 21.8)	21.0 (19.2, 22.9)
urban-rural difference	0.5 (-0.3, 1.3)	0.5 (-0.6, 1.6)	0.5 (-0.4, 1.3)	0.4 (-0.3, 1.3)	0.4 (-0.5, 1.3)	0.3 (-0.5, 1.1)	0.3 (-0.7, 1.4)	0.3 (-0.5, 1.1)	0.3 (-0.5, 1.0)	0.3 (-0.6, 1.1)
East Africa										
Burundi										
rural	16.5 (14.1, 18.9)	14.5 (11.1, 17.9)	15.2 (12.8, 17.6)	17.8 (15.4, 20.0)	19.6 (16.8, 22.4)	18.2 (16.0, 20.5)	16.3 (12.8, 19.7)	17.0 (14.7, 19.2)	19.5 (17.4, 21.7)	21.4 (18.9, 24.0)
urban	16.9 (14.6, 19.3)	15.1 (11.6, 18.5)	15.7 (13.3, 18.1)	18.2 (15.9, 20.5)	20.0 (17.3, 22.7)	18.5 (16.3, 20.8)	16.7 (13.2, 20.0)	17.3 (15.1, 19.6)	19.8 (17.6, 21.9)	21.6 (19.1, 24.2)
urban-rural difference	0.4 (-0.3, 1.2)	0.6 (-0.4, 1.5)	0.5 (-0.3, 1.3)	0.4 (-0.3, 1.1)	0.3 (-0.5, 1.2)	0.3 (-0.3, 0.9)	0.4 (-0.5, 1.3)	0.3 (-0.4, 1.0)	0.2 (-0.4, 0.9)	0.2 (-0.5, 0.9)
Comoros										
rural	16.5 (14.2, 18.8)	14.5 (11.0, 17.9)	15.2 (12.9, 17.6)	17.8 (15.5, 20.0)	19.6 (17.0, 22.3)	18.2 (16.0, 20.5)	16.3 (12.9, 19.6)	17.0 (14.8, 19.3)	19.5 (17.4, 21.7)	21.4 (18.8, 24.0)
urban	16.9 (14.6, 19.3)	15.1 (11.6, 18.5)	15.7 (13.4, 18.1)	18.2 (15.9, 20.5)	20.0 (17.3, 22.7)	18.5 (16.3, 20.8)	16.7 (13.3, 20.1)	17.3 (15.1, 19.6)	19.8 (17.7, 22.0)	21.6 (19.0, 24.2)
urban-rural difference	0.5 (-0.3, 1.2)	0.6 (-0.4, 1.5)	0.5 (-0.3, 1.3)	0.4 (-0.3, 1.1)	0.3 (-0.5, 1.1)	0.3 (-0.4, 1.0)	0.4 (-0.6, 1.3)	0.3 (-0.4, 1.0)	0.2 (-0.4, 0.9)	0.2 (-0.5, 0.9)
Djibouti										

Boys	Mean BMI in 1990 (kg/m^2)					Mean BMI in 2020 (kg/m^2)				
	Age-standardised	5 years	10 years	15 years	19 years	Age-standardised	5 years	10 years	15 years	19 years
rural	16.5 (14.1, 18.9)	14.5 (10.9, 18.2)	15.2 (12.9, 17.7)	17.8 (15.5, 20.1)	19.6 (16.9, 22.4)	18.2 (16.0, 20.4)	16.2 (12.7, 19.7)	17.0 (14.8, 19.2)	19.5 (17.4, 21.7)	21.4 (18.8, 23.9)
urban	16.9 (14.6, 19.3)	15.0 (11.5, 18.7)	15.7 (13.4, 18.1)	18.2 (15.9, 20.4)	20.0 (17.3, 22.7)	18.5 (16.3, 20.8)	16.6 (13.2, 20.1)	17.3 (15.1, 19.5)	19.8 (17.6, 21.9)	21.6 (19.0, 24.1)
urban-rural difference	0.5 (-0.2, 1.2)	0.6 (-0.4, 1.6)	0.5 (-0.3, 1.3)	0.4 (-0.3, 1.1)	0.3 (-0.5, 1.2)	0.3 (-0.3, 1.0)	0.4 (-0.5, 1.4)	0.3 (-0.4, 1.0)	0.3 (-0.4, 0.9)	0.2 (-0.5, 0.9)
Eritrea										
rural	15.8 (14.1, 17.7)	13.8 (10.8, 17.0)	14.6 (12.8, 16.5)	17.1 (15.4, 18.7)	19.0 (17.3, 20.8)	17.6 (16.0, 19.2)	15.6 (12.6, 18.7)	16.3 (14.7, 18.0)	18.9 (17.4, 20.4)	20.8 (19.2, 22.4)
urban	16.3 (14.5, 18.1)	14.4 (11.3, 17.6)	15.1 (13.2, 16.9)	17.5 (15.8, 19.2)	19.4 (17.6, 21.2)	17.9 (16.2, 19.6)	16.0 (13.0, 19.2)	16.7 (15.0, 18.4)	19.1 (17.6, 20.7)	21.0 (19.3, 22.6)
urban-rural difference	0.5 (-0.3, 1.2)	0.6 (-0.4, 1.6)	0.5 (-0.3, 1.3)	0.4 (-0.3, 1.1)	0.4 (-0.4, 1.2)	0.3 (-0.4, 1.0)	0.4 (-0.5, 1.4)	0.3 (-0.4, 1.1)	0.3 (-0.4, 0.9)	0.2 (-0.5, 0.9)
Ethiopia										
rural	15.0 (13.3, 16.8)	13.0 (9.9, 16.1)	13.8 (12.0, 15.6)	16.3 (14.7, 17.9)	18.1 (16.5, 19.7)	16.6 (15.6, 17.7)	14.6 (11.9, 17.4)	15.4 (14.3, 16.5)	17.9 (17.1, 18.8)	19.7 (18.9, 20.6)
urban	15.5 (13.8, 17.3)	13.6 (10.5, 16.7)	14.3 (12.5, 16.1)	16.7 (15.2, 18.4)	18.4 (16.9, 20.1)	17.0 (15.9, 18.1)	15.1 (12.4, 17.9)	15.8 (14.7, 17.0)	18.3 (17.4, 19.1)	20.0 (19.1, 20.8)
urban-rural difference	0.5 (-0.1, 1.2)	0.6 (-0.3, 1.6)	0.5 (-0.2, 1.3)	0.5 (-0.2, 1.1)	0.4 (-0.3, 1.1)	0.4 (-0.1, 0.9)	0.5 (-0.3, 1.3)	0.4 (-0.2, 1.0)	0.3 (-0.1, 0.7)	0.2 (-0.2, 0.7)
Kenya										
rural	16.6 (14.9, 18.4)	14.9 (12.1, 17.7)	15.4 (13.6, 17.2)	17.8 (16.1, 19.5)	19.4 (17.7, 21.2)	18.4 (17.1, 19.7)	16.7 (14.2, 19.3)	17.2 (15.8, 18.6)	19.6 (18.3, 20.9)	21.2 (20.2, 22.3)
urban	17.0 (15.3, 18.7)	15.5 (12.7, 18.3)	15.9 (14.1, 17.6)	18.2 (16.5, 19.9)	19.7 (18.0, 21.5)	18.7 (17.4, 19.9)	17.1 (14.6, 19.6)	17.5 (16.2, 18.9)	19.8 (18.6, 21.0)	21.4 (20.3, 22.4)
urban-rural difference	0.5 (-0.3, 1.2)	0.6 (-0.4, 1.6)	0.5 (-0.3, 1.3)	0.4 (-0.3, 1.1)	0.3 (-0.5, 1.1)	0.3 (-0.3, 0.9)	0.4 (-0.5, 1.4)	0.3 (-0.3, 1.0)	0.2 (-0.3, 0.8)	0.1 (-0.5, 0.8)
Madagascar										
rural	16.5 (14.1, 18.9)	14.5 (11.1, 18.0)	15.2 (12.9, 17.6)	17.8 (15.5, 20.1)	19.6 (17.0, 22.4)	18.2 (16.0, 20.4)	16.2 (12.9, 19.6)	17.0 (14.7, 19.2)	19.5 (17.4, 21.6)	21.4 (18.8, 24.0)
urban	16.9 (14.5, 19.3)	15.0 (11.6, 18.5)	15.7 (13.3, 18.1)	18.2 (15.9, 20.5)	20.0 (17.3, 22.8)	18.5 (16.2, 20.7)	16.6 (13.3, 19.9)	17.3 (15.1, 19.4)	19.8 (17.6, 21.9)	21.6 (19.0, 24.1)
urban-rural difference	0.4 (-0.3, 1.2)	0.6 (-0.4, 1.5)	0.5 (-0.3, 1.2)	0.4 (-0.3, 1.1)	0.3 (-0.5, 1.1)	0.3 (-0.4, 0.9)	0.4 (-0.6, 1.3)	0.3 (-0.4, 1.0)	0.2 (-0.4, 0.9)	0.2 (-0.5, 0.9)
Malawi										
rural	16.1 (14.3, 17.9)	14.1 (11.0, 17.3)	14.8 (12.9, 16.7)	17.3 (15.6, 19.0)	19.5 (17.8, 21.3)	17.8 (16.5, 19.0)	15.8 (12.9, 18.7)	16.5 (15.1, 17.8)	19.0 (17.9, 20.1)	21.2 (20.2, 22.1)
urban	16.5 (14.7, 18.3)	14.7 (11.6, 17.8)	15.3 (13.4, 17.1)	17.7 (16.0, 19.4)	19.8 (18.1, 21.5)	18.0 (16.8, 19.3)	16.2 (13.3, 19.1)	16.7 (15.4, 18.2)	19.2 (18.1, 20.3)	21.3 (20.4, 22.3)
urban-rural difference	0.4 (-0.3, 1.1)	0.5 (-0.4, 1.5)	0.5 (-0.3, 1.2)	0.4 (-0.3, 1.1)	0.3 (-0.4, 1.1)	0.3 (-0.4, 0.8)	0.4 (-0.5, 1.3)	0.3 (-0.4, 0.9)	0.2 (-0.3, 0.7)	0.1 (-0.5, 0.7)
Mauritius										
rural	17.6 (15.9, 19.3)	15.8 (12.8, 18.8)	16.4 (14.7, 18.1)	18.8 (17.2, 20.4)	20.4 (18.4, 22.3)	19.6 (18.7, 20.6)	17.8 (15.1, 20.5)	18.5 (17.5, 19.4)	20.8 (20.1, 21.6)	22.4 (21.0, 23.7)
urban	18.0 (16.3, 19.8)	16.3 (13.3, 19.3)	16.9 (15.2, 18.6)	19.2 (17.6, 20.8)	20.7 (18.7, 22.7)	19.9 (18.8, 20.9)	18.1 (15.5, 20.8)	18.7 (17.7, 19.8)	21.1 (20.2, 21.9)	22.5 (21.1, 24.0)
urban-rural difference	0.4 (-0.3, 1.2)	0.5 (-0.4, 1.5)	0.4 (-0.3, 1.2)	0.4 (-0.3, 1.1)	0.3 (-0.5, 1.1)	0.3 (-0.4, 0.9)	0.3 (-0.6, 1.2)	0.3 (-0.4, 1.0)	0.2 (-0.4, 0.8)	0.2 (-0.6, 0.9)
Mozambique										
rural	16.8 (15.3, 18.5)	15.1 (12.9, 17.3)	15.5 (13.9, 17.1)	18.1 (16.5, 19.7)	20.2 (18.5, 22.0)	18.3 (17.3, 19.3)	16.6 (14.7, 18.4)	17.0 (15.9, 18.0)	19.5 (18.6, 20.5)	21.7 (20.6, 22.8)
urban	17.4 (15.8, 18.9)	15.7 (13.5, 17.9)	16.0 (14.5, 17.6)	18.5 (17.0, 20.1)	20.6 (19.0, 22.3)	18.7 (17.7, 19.7)	17.0 (15.3, 18.8)	17.4 (16.4, 18.4)	19.9 (18.9, 20.8)	21.9 (20.8, 23.0)
urban-rural difference	0.5 (-0.1, 1.2)	0.6 (-0.3, 1.5)	0.5 (-0.1, 1.3)	0.5 (-0.2, 1.1)	0.4 (-0.3, 1.1)	0.4 (-0.2, 0.9)	0.5 (-0.4, 1.3)	0.4 (-0.2, 1.0)	0.3 (-0.2, 0.8)	0.3 (-0.3, 0.9)
Rwanda										
rural	16.2 (14.5, 17.9)	14.2 (11.2, 17.3)	14.8 (13.1, 16.5)	17.5 (15.9, 19.1)	19.9 (18.2, 21.4)	17.9 (16.7, 19.1)	15.9 (13.2, 18.9)	16.5 (15.3, 17.8)	19.2 (18.2, 20.2)	21.6 (20.6, 22.6)
urban	16.6 (14.9, 18.3)	14.9 (11.9, 17.9)	15.3 (13.6, 17.0)	17.8 (16.2, 19.4)	20.1 (18.4, 21.7)	18.2 (17.0, 19.4)	16.4 (13.7, 19.4)	16.8 (15.6, 18.1)	19.4 (18.4, 20.3)	21.6 (20.6, 22.6)
urban-rural difference	0.4 (-0.2, 1.1)	0.7 (-0.3, 1.6)	0.5 (-0.2, 1.2)	0.3 (-0.3, 0.9)	0.2 (-0.5, 0.8)	0.3 (-0.2, 0.8)	0.5 (-0.4, 1.4)	0.3 (-0.3, 0.9)	0.2 (-0.3, 0.6)	0.0 (-0.5, 0.5)
Seychelles										
rural	17.4 (16.2, 18.6)	14.2 (13.0, 15.4)	16.4 (15.2, 17.6)	19.2 (18.0, 20.4)	19.9 (18.2, 21.5)	19.5 (19.1, 20.0)	16.4 (16.0, 16.8)	18.6 (18.2, 19.0)	21.4 (21.0, 21.8)	22.0 (20.8, 23.1)
urban	17.7 (16.5, 18.9)	14.7 (13.5, 15.9)	16.8 (15.6, 18.0)	19.4 (18.2, 20.6)	19.9 (18.3, 21.6)	19.6 (19.2, 20.0)	16.7 (16.2, 17.1)	18.7 (18.3, 19.1)	21.3 (20.9, 21.8)	21.9 (20.6, 23.0)
urban-rural difference	0.3 (-0.1, 0.7)	0.5 (0.1, 0.9)	0.3 (0.0, 0.7)	0.2 (-0.2, 0.6)	0.1 (-0.3, 0.5)	0.1 (-0.2, 0.3)	0.3 (0.0, 0.6)	0.1 (-0.1, 0.4)	0.0 (-0.3, 0.3)	-0.1 (-0.5, 0.2)
Somalia										
rural	16.5 (14.1, 18.8)	14.5 (10.9, 17.9)	15.2 (12.8, 17.6)	17.8 (15.5, 20.1)	19.6 (16.9, 22.3)	18.2 (16.0, 20.4)	16.2 (12.8, 19.5)	16.9 (14.8, 19.1)	19.5 (17.4, 21.6)	21.4 (18.9, 24.0)
urban	16.9 (14.5, 19.3)	15.0 (11.5, 18.5)	15.7 (13.3, 18.1)	18.2 (15.9, 20.4)	20.0 (17.3, 22.6)	18.5 (16.3, 20.7)	16.6 (13.2, 19.9)	17.3 (15.1, 19.5)	19.7 (17.6, 21.9)	21.5 (19.1, 24.1)
urban-rural difference	0.4 (-0.3, 1.1)	0.6 (-0.4, 1.5)	0.5 (-0.3, 1.2)	0.4 (-0.3, 1.1)	0.3 (-0.5, 1.1)	0.3 (-0.4, 1.0)	0.4 (-0.5, 1.4)	0.3 (-0.4, 1.0)	0.2 (-0.4, 0.9)	0.2 (-0.6, 0.9)
South Sudan										
rural	16.9 (14.6, 19.1)	14.9 (11.4, 18.3)	15.6 (13.3, 17.8)	18.1 (16.0, 20.3)	20.2 (17.7, 22.6)	18.7 (16.5, 20.8)	16.7 (13.4, 20.0)	17.4 (15.2, 19.5)	20.0 (17.9, 21.9)	22.0 (19.8, 24.3)
urban	17.3 (15.1, 19.6)	15.5 (12.0, 18.9)	16.1 (13.8, 18.3)	18.5 (16.4, 20.7)	20.6 (18.1, 23.0)	19.0 (16.9, 21.0)	17.1 (13.9, 20.4)	17.7 (15.6, 19.8)	20.2 (18.2, 22.2)	22.2 (20.0, 24.5)
urban-rural difference	0.5 (-0.3, 1.2)	0.6 (-0.4, 1.5)	0.5 (-0.3, 1.3)	0.4 (-0.3, 1.1)	0.4 (-0.4, 1.1)	0.3 (-0.4, 1.0)	0.4 (-0.5, 1.4)	0.3 (-0.4, 1.1)	0.3 (-0.4, 0.9)	0.2 (-0.5, 0.9)
Sudan										
rural	16.1 (14.2, 18.0)	14.1 (11.0, 17.1)	14.9 (12.9, 16.9)	17.4 (15.7, 19.2)	19.1 (17.3, 20.9)	17.8 (16.4, 19.2)	15.8 (12.9, 18.6)	16.6 (15.1, 18.1)	19.1 (17.8, 20.4)	20.8 (19.6, 21.9)
urban	16.6 (14.7, 18.4)	14.7 (11.4, 17.7)	15.4 (13.4, 17.3)	17.9 (16.1, 19.7)	19.5 (17.7, 21.3)	18.2 (16.8, 19.6)	16.2 (13.4, 19.1)	17.0 (15.5, 18.5)	19.5 (18.1, 20.8)	21.0 (19.8, 22.3)
urban-rural difference	0.5 (-0.2, 1.2)	0.6 (-0.4, 1.6)	0.5 (-0.2, 1.3)	0.5 (-0.2, 1.2)	0.4 (-0.3, 1.2)	0.4 (-0.3, 1.0)	0.4 (-0.5, 1.4)	0.4 (-0.3, 1.1)	0.3 (-0.3, 0.9)	0.3 (-0.4, 1.0)

Boys	Mean BMI in 1990 (kg/m²)					Mean BMI in 2020 (kg/m²)				
	Age-standardised	5 years	10 years	15 years	19 years	Age-standardised	5 years	10 years	15 years	19 years
Tanzania										
rural	16.6 (14.7, 18.5)	15.1 (12.8, 17.3)	15.2 (13.3, 17.1)	17.6 (15.8, 19.5)	20.1 (17.9, 22.2)	18.3 (16.7, 19.9)	16.8 (14.8, 18.8)	16.9 (15.3, 18.5)	19.4 (17.8, 20.9)	21.8 (20.0, 23.6)
urban	17.0 (15.1, 19.0)	15.7 (13.3, 18.1)	15.7 (13.7, 17.6)	18.0 (16.2, 19.9)	20.4 (18.2, 22.6)	18.6 (16.9, 20.3)	17.2 (15.0, 19.5)	17.2 (15.5, 18.9)	19.6 (18.0, 21.2)	22.0 (20.1, 23.9)
urban-rural difference	0.5 (-0.3, 1.2)	0.6 (-0.4, 1.5)	0.5 (-0.3, 1.3)	0.4 (-0.3, 1.1)	0.3 (-0.5, 1.1)	0.3 (-0.3, 0.9)	0.4 (-0.5, 1.4)	0.3 (-0.3, 1.0)	0.2 (-0.4, 0.9)	0.2 (-0.5, 0.9)
Uganda										
rural	16.3 (14.8, 17.9)	14.4 (12.8, 16.1)	14.9 (13.3, 16.5)	17.5 (16.0, 19.1)	19.8 (18.3, 21.4)	18.0 (17.1, 18.8)	16.2 (15.2, 17.1)	16.6 (15.7, 17.5)	19.2 (18.4, 20.1)	21.5 (20.7, 22.4)
urban	16.9 (15.3, 18.5)	15.2 (13.5, 17.0)	15.5 (13.9, 17.1)	18.1 (16.5, 19.6)	20.2 (18.7, 21.8)	18.5 (17.6, 19.4)	16.8 (15.6, 18.0)	17.1 (16.2, 18.1)	19.7 (18.8, 20.5)	21.8 (21.0, 22.7)
urban-rural difference	0.6 (0.0, 1.2)	0.8 (-0.1, 1.7)	0.6 (0.0, 1.3)	0.5 (-0.1, 1.1)	0.4 (-0.2, 1.0)	0.5 (0.0, 1.0)	0.7 (-0.1, 1.5)	0.5 (0.0, 1.1)	0.4 (0.0, 0.8)	0.3 (-0.1, 0.7)
Zambia										
rural	16.6 (14.7, 18.4)	14.5 (11.5, 17.7)	15.3 (13.4, 17.3)	17.9 (16.1, 19.7)	19.6 (17.9, 21.3)	18.4 (17.0, 19.7)	16.3 (13.4, 19.1)	17.1 (15.6, 18.6)	19.7 (18.4, 20.9)	21.4 (20.4, 22.4)
urban	17.0 (15.1, 18.9)	15.1 (12.0, 18.2)	15.8 (13.9, 17.7)	18.3 (16.5, 20.1)	19.9 (18.1, 21.6)	18.6 (17.3, 19.9)	16.7 (13.9, 19.5)	17.4 (16.0, 18.9)	19.9 (18.7, 21.1)	21.5 (20.5, 22.5)
urban-rural difference	0.4 (-0.3, 1.1)	0.6 (-0.4, 1.5)	0.5 (-0.3, 1.2)	0.4 (-0.3, 1.0)	0.3 (-0.4, 1.1)	0.3 (-0.4, 0.9)	0.4 (-0.5, 1.3)	0.3 (-0.4, 1.0)	0.2 (-0.4, 0.8)	0.1 (-0.5, 0.8)
Southern Africa										
Botswana										
rural	16.0 (14.2, 17.8)	13.8 (10.4, 17.1)	14.8 (12.9, 16.6)	17.3 (15.6, 19.0)	19.1 (17.3, 21.0)	17.6 (16.1, 19.0)	15.4 (12.3, 18.5)	16.4 (14.8, 17.9)	18.9 (17.6, 20.3)	20.7 (19.3, 22.1)
urban	16.5 (14.7, 18.4)	14.3 (11.0, 17.5)	15.3 (13.4, 17.2)	17.9 (16.2, 19.6)	19.8 (17.9, 21.6)	17.7 (16.3, 19.1)	15.5 (12.4, 18.5)	16.5 (15.0, 18.0)	19.1 (17.8, 20.4)	20.9 (19.5, 22.3)
urban-rural difference	0.6 (-0.2, 1.4)	0.5 (-0.5, 1.6)	0.6 (-0.2, 1.4)	0.6 (-0.2, 1.4)	0.6 (-0.2, 1.5)	0.1 (-0.6, 0.8)	0.1 (-0.9, 1.0)	0.1 (-0.6, 0.9)	0.2 (-0.5, 0.8)	0.2 (-0.6, 1.0)
Lesotho										
rural	16.0 (14.2, 17.8)	13.7 (10.6, 17.0)	14.8 (13.0, 16.6)	17.4 (15.8, 19.0)	19.1 (17.5, 20.7)	17.7 (16.4, 19.0)	15.5 (12.5, 18.4)	16.5 (15.1, 17.8)	19.1 (18.0, 20.1)	20.8 (19.6, 21.9)
urban	16.4 (14.7, 18.3)	14.2 (11.0, 17.5)	15.2 (13.4, 17.1)	17.8 (16.2, 19.4)	19.5 (17.9, 21.2)	17.7 (16.3, 19.0)	15.5 (12.5, 18.4)	16.5 (15.1, 17.8)	19.1 (18.0, 20.2)	20.8 (19.6, 21.9)
urban-rural difference	0.5 (-0.2, 1.2)	0.5 (-0.5, 1.5)	0.5 (-0.3, 1.3)	0.5 (-0.2, 1.2)	0.5 (-0.3, 1.2)	0.0 (-0.6, 0.6)	0.0 (-0.9, 1.0)	0.0 (-0.7, 0.7)	0.0 (-0.5, 0.5)	0.0 (-0.6, 0.5)
Namibia										
rural	16.5 (14.5, 18.5)	14.4 (11.0, 17.8)	15.3 (13.3, 17.4)	17.8 (15.8, 19.7)	19.5 (17.4, 21.5)	18.2 (16.2, 20.1)	16.0 (12.7, 19.3)	17.0 (15.0, 18.9)	19.5 (17.7, 21.3)	21.1 (19.2, 23.0)
urban	17.1 (15.1, 19.1)	14.9 (11.5, 18.3)	15.9 (13.9, 18.0)	18.4 (16.5, 20.3)	20.1 (18.0, 22.1)	18.3 (16.5, 20.2)	16.2 (12.8, 19.4)	17.2 (15.3, 19.0)	19.6 (17.9, 21.3)	21.3 (19.5, 23.1)
urban-rural difference	0.6 (-0.2, 1.4)	0.6 (-0.5, 1.6)	0.6 (-0.2, 1.4)	0.6 (-0.1, 1.4)	0.6 (-0.2, 1.5)	0.2 (-0.5, 0.9)	0.1 (-0.8, 1.1)	0.1 (-0.6, 0.9)	0.2 (-0.5, 0.8)	0.2 (-0.6, 0.9)
South Africa										
rural	16.1 (14.9, 17.4)	14.1 (12.8, 15.3)	15.0 (13.8, 16.3)	17.4 (16.2, 18.7)	18.9 (17.7, 20.2)	18.3 (17.6, 18.9)	16.2 (15.6, 16.9)	17.2 (16.5, 17.8)	19.6 (18.9, 20.2)	21.1 (20.4, 21.8)
urban	16.9 (15.6, 18.2)	14.3 (13.1, 15.7)	15.6 (14.3, 16.9)	18.4 (17.1, 19.7)	20.2 (18.8, 21.5)	18.4 (17.8, 19.0)	15.9 (15.2, 16.5)	17.2 (16.5, 17.8)	19.9 (19.3, 20.5)	21.7 (21.0, 22.4)
urban-rural difference	0.7 (0.2, 1.3)	0.3 (-0.3, 0.9)	0.6 (0.1, 1.2)	0.9 (0.4, 1.5)	1.2 (0.7, 1.8)	0.1 (-0.2, 0.5)	-0.3 (-0.7, 0.1)	0.0 (-0.4, 0.3)	0.3 (0.0, 0.7)	0.6 (0.2, 1.0)
Eswatini										
rural	16.8 (15.0, 18.5)	14.6 (11.4, 17.8)	15.5 (13.7, 17.2)	18.1 (16.5, 19.7)	20.2 (18.6, 21.8)	18.5 (17.2, 19.9)	16.4 (13.3, 19.5)	17.2 (15.8, 18.6)	19.8 (18.7, 20.9)	21.9 (20.8, 23.1)
urban	17.4 (15.7, 19.2)	15.2 (12.0, 18.4)	16.1 (14.3, 17.9)	18.8 (17.2, 20.4)	20.9 (19.3, 22.5)	18.7 (17.3, 20.1)	16.5 (13.5, 19.6)	17.4 (15.9, 18.9)	20.1 (18.9, 21.2)	22.2 (21.0, 23.4)
urban-rural difference	0.6 (-0.1, 1.4)	0.6 (-0.4, 1.6)	0.6 (-0.1, 1.4)	0.7 (0.0, 1.4)	0.7 (0.0, 1.5)	0.2 (-0.4, 0.9)	0.1 (-0.8, 1.1)	0.2 (-0.5, 0.9)	0.3 (-0.3, 0.8)	0.3 (-0.3, 0.9)
Zimbabwe										
rural	16.8 (15.3, 18.3)	14.6 (11.5, 17.6)	15.5 (14.0, 17.1)	18.1 (16.8, 19.4)	20.2 (19.0, 21.5)	17.7 (16.4, 18.9)	15.5 (12.4, 18.5)	16.4 (15.1, 17.7)	19.0 (18.0, 20.0)	21.1 (20.1, 22.1)
urban	17.4 (15.9, 19.0)	15.3 (12.1, 18.4)	16.2 (14.7, 17.8)	18.7 (17.4, 20.1)	20.8 (19.5, 22.1)	17.9 (16.7, 19.2)	15.8 (12.7, 18.8)	16.7 (15.4, 18.0)	19.2 (18.2, 20.3)	21.3 (20.3, 22.3)
urban-rural difference	0.6 (-0.1, 1.4)	0.7 (-0.3, 1.7)	0.7 (-0.1, 1.5)	0.6 (-0.1, 1.4)	0.6 (-0.2, 1.4)	0.3 (-0.3, 0.8)	0.3 (-0.6, 1.2)	0.3 (-0.4, 0.9)	0.2 (-0.2, 0.7)	0.2 (-0.3, 0.7)
West Africa										
Benin										
rural	16.7 (14.9, 18.6)	14.7 (11.6, 17.8)	15.5 (13.5, 17.4)	17.9 (16.1, 19.7)	20.3 (18.5, 22.2)	18.5 (17.1, 19.9)	16.5 (13.5, 19.4)	17.3 (15.7, 18.8)	19.8 (18.5, 21.1)	22.1 (20.9, 23.4)
urban	17.2 (15.4, 19.2)	15.3 (12.1, 18.4)	16.0 (14.0, 18.0)	18.4 (16.6, 20.2)	20.7 (18.8, 22.6)	18.8 (17.3, 20.2)	16.8 (13.8, 19.7)	17.5 (16.0, 19.1)	19.9 (18.6, 21.3)	22.2 (20.9, 23.5)
urban-rural difference	0.5 (-0.3, 1.3)	0.6 (-0.4, 1.7)	0.5 (-0.3, 1.4)	0.5 (-0.3, 1.3)	0.4 (-0.4, 1.3)	0.2 (-0.5, 0.9)	0.3 (-0.6, 1.3)	0.2 (-0.5, 1.0)	0.2 (-0.5, 0.8)	0.1 (-0.6, 0.8)
Burkina Faso										
rural	16.2 (14.4, 18.0)	14.5 (12.7, 16.4)	14.8 (13.0, 16.6)	17.2 (15.5, 19.1)	20.1 (18.2, 22.0)	18.0 (16.3, 19.6)	16.3 (14.5, 17.9)	16.6 (14.9, 18.2)	19.0 (17.3, 20.7)	21.9 (20.1, 23.7)
urban	16.7 (14.9, 18.6)	15.2 (13.2, 17.2)	15.4 (13.5, 17.2)	17.7 (15.9, 19.6)	20.5 (18.5, 22.5)	18.2 (16.4, 19.9)	16.6 (14.7, 18.5)	16.8 (15.1, 18.6)	19.2 (17.4, 20.9)	22.0 (20.1, 23.9)
urban-rural difference	0.5 (-0.2, 1.3)	0.7 (-0.3, 1.7)	0.6 (-0.2, 1.4)	0.5 (-0.3, 1.3)	0.4 (-0.4, 1.2)	0.2 (-0.5, 0.9)	0.4 (-0.6, 1.3)	0.3 (-0.5, 1.0)	0.2 (-0.5, 0.8)	0.1 (-0.7, 0.8)
Cabo Verde										
rural	16.3 (14.4, 18.2)	14.2 (11.0, 17.3)	15.1 (13.1, 17.0)	17.5 (15.8, 19.3)	19.6 (17.8, 21.5)	18.0 (16.8, 19.3)	16.0 (13.1, 18.7)	16.8 (15.4, 18.3)	19.3 (18.1, 20.4)	21.4 (20.4, 22.4)
urban	16.9 (15.0, 18.8)	14.9 (11.7, 17.9)	15.7 (13.7, 17.6)	18.1 (16.3, 19.8)	20.2 (18.4, 22.0)	18.4 (17.1, 19.6)	16.3 (13.5, 19.1)	17.2 (15.7, 18.6)	19.6 (18.4, 20.8)	21.6 (20.7, 22.6)
urban-rural difference	0.6 (-0.2, 1.4)	0.6 (-0.4, 1.7)	0.6 (-0.2, 1.5)	0.6 (-0.2, 1.4)	0.5 (-0.3, 1.4)	0.3 (-0.3, 1.0)	0.4 (-0.6, 1.3)	0.3 (-0.4, 1.1)	0.3 (-0.3, 0.9)	0.3 (-0.4, 0.9)
Cameroon										

Boys	Mean BMI in 1990 (kg/m^2)					Mean BMI in 2020 (kg/m^2)				
	Age-standardised	5 years	10 years	15 years	19 years	Age-standardised	5 years	10 years	15 years	19 years
rural	17.0 (15.2, 18.7)	14.9 (11.8, 18.0)	15.6 (13.7, 17.4)	18.1 (16.5, 19.7)	20.9 (19.2, 22.5)	18.7 (17.1, 20.3)	16.7 (13.6, 19.8)	17.4 (15.7, 19.0)	19.9 (18.4, 21.4)	22.7 (21.1, 24.1)
urban	17.5 (15.8, 19.2)	15.6 (12.4, 18.7)	16.2 (14.4, 18.0)	18.6 (17.0, 20.2)	21.3 (19.7, 23.0)	18.9 (17.3, 20.6)	17.0 (14.0, 20.1)	17.6 (15.9, 19.3)	20.1 (18.6, 21.5)	22.7 (21.3, 24.2)
urban-rural difference	0.6 (-0.2, 1.3)	0.7 (-0.3, 1.7)	0.6 (-0.2, 1.4)	0.5 (-0.2, 1.2)	0.4 (-0.3, 1.2)	0.2 (-0.5, 0.9)	0.3 (-0.6, 1.3)	0.2 (-0.5, 1.0)	0.2 (-0.5, 0.8)	0.1 (-0.6, 0.8)
Chad										
rural	16.3 (13.9, 18.7)	14.2 (10.7, 17.7)	15.1 (12.7, 17.5)	17.6 (15.3, 19.8)	19.8 (17.1, 22.4)	18.1 (15.8, 20.3)	16.0 (12.5, 19.4)	16.9 (14.6, 19.1)	19.3 (17.2, 21.4)	21.5 (19.0, 24.0)
urban	16.8 (14.4, 19.2)	14.8 (11.3, 18.3)	15.6 (13.2, 18.0)	18.0 (15.7, 20.2)	20.2 (17.4, 22.8)	18.3 (16.0, 20.5)	16.3 (12.8, 19.7)	17.1 (14.8, 19.3)	19.5 (17.3, 21.6)	21.6 (19.1, 24.1)
urban-rural difference	0.5 (-0.3, 1.3)	0.6 (-0.4, 1.7)	0.5 (-0.3, 1.3)	0.4 (-0.3, 1.2)	0.4 (-0.5, 1.2)	0.2 (-0.5, 0.9)	0.3 (-0.7, 1.3)	0.2 (-0.5, 1.0)	0.1 (-0.5, 0.8)	0.1 (-0.7, 0.8)
Côte d'Ivoire										
rural	16.7 (14.8, 18.7)	14.6 (11.4, 18.0)	15.5 (13.4, 17.5)	17.9 (16.1, 19.8)	20.4 (18.5, 22.3)	18.5 (16.7, 20.3)	16.4 (13.2, 19.6)	17.2 (15.4, 19.1)	19.7 (18.0, 21.4)	22.2 (20.5, 23.9)
urban	17.2 (15.3, 19.2)	15.3 (12.0, 18.6)	16.0 (14.0, 18.0)	18.3 (16.5, 20.2)	20.7 (18.8, 22.6)	18.7 (16.9, 20.4)	16.7 (13.5, 19.9)	17.4 (15.6, 19.2)	19.8 (18.1, 21.4)	22.2 (20.4, 23.9)
urban-rural difference	0.5 (-0.2, 1.3)	0.6 (-0.4, 1.7)	0.5 (-0.3, 1.3)	0.4 (-0.3, 1.1)	0.3 (-0.5, 1.1)	0.2 (-0.5, 0.8)	0.3 (-0.6, 1.3)	0.2 (-0.5, 0.9)	0.1 (-0.5, 0.7)	0.0 (-0.7, 0.7)
Gambia										
rural	16.4 (14.0, 18.8)	14.3 (10.7, 17.8)	15.1 (12.7, 17.5)	17.6 (15.3, 19.9)	19.8 (17.1, 22.5)	18.1 (15.9, 20.4)	16.0 (12.6, 19.4)	16.9 (14.6, 19.2)	19.3 (17.2, 21.5)	21.5 (19.0, 24.2)
urban	16.9 (14.4, 19.3)	14.9 (11.3, 18.4)	15.6 (13.2, 18.1)	18.0 (15.7, 20.3)	20.2 (17.5, 23.0)	18.3 (16.1, 20.5)	16.3 (12.8, 19.7)	17.1 (14.9, 19.4)	19.5 (17.3, 21.6)	21.6 (19.1, 24.1)
urban-rural difference	0.5 (-0.3, 1.3)	0.6 (-0.4, 1.7)	0.5 (-0.3, 1.4)	0.4 (-0.3, 1.2)	0.4 (-0.4, 1.2)	0.2 (-0.5, 0.9)	0.3 (-0.7, 1.3)	0.2 (-0.5, 1.0)	0.1 (-0.6, 0.8)	0.1 (-0.7, 0.8)
Ghana										
rural	16.4 (14.7, 18.0)	14.3 (12.6, 16.0)	15.2 (13.6, 16.8)	17.7 (16.0, 19.2)	19.4 (17.7, 20.9)	18.4 (17.7, 19.1)	16.3 (15.5, 17.2)	17.3 (16.6, 18.0)	19.7 (19.0, 20.4)	21.4 (20.7, 22.1)
urban	16.9 (15.2, 18.5)	15.0 (13.3, 16.6)	15.8 (14.1, 17.4)	18.0 (16.4, 19.7)	19.6 (18.0, 21.2)	18.5 (17.9, 19.2)	16.7 (15.9, 17.5)	17.5 (16.7, 18.2)	19.7 (19.0, 20.4)	21.3 (20.6, 22.0)
urban-rural difference	0.5 (-0.2, 1.3)	0.7 (-0.1, 1.6)	0.5 (-0.2, 1.3)	0.4 (-0.3, 1.2)	0.3 (-0.5, 1.1)	0.1 (-0.3, 0.5)	0.3 (-0.2, 0.9)	0.2 (-0.2, 0.6)	0.0 (-0.4, 0.4)	-0.1 (-0.5, 0.3)
Guinea										
rural	16.1 (14.2, 18.1)	14.3 (10.8, 17.8)	15.1 (12.9, 17.5)	17.6 (15.4, 19.8)	19.8 (17.3, 22.4)	17.9 (16.1, 19.6)	16.0 (12.6, 19.5)	16.9 (14.7, 19.1)	19.4 (17.3, 21.5)	21.6 (19.0, 24.1)
urban	16.7 (14.7, 18.6)	14.9 (11.4, 18.5)	15.7 (13.4, 18.0)	18.1 (15.9, 20.3)	20.2 (17.6, 22.8)	18.1 (16.3, 19.9)	16.3 (13.0, 19.7)	17.1 (15.0, 19.3)	19.5 (17.4, 21.6)	21.7 (19.1, 24.2)
urban-rural difference	0.5 (-0.3, 1.3)	0.6 (-0.4, 1.7)	0.5 (-0.3, 1.4)	0.5 (-0.3, 1.2)	0.4 (-0.5, 1.2)	0.2 (-0.5, 0.9)	0.3 (-0.6, 1.3)	0.2 (-0.5, 1.0)	0.1 (-0.5, 0.8)	0.1 (-0.7, 0.8)
Guinea Bissau										
rural	16.4 (14.1, 18.8)	14.1 (10.8, 17.3)	14.8 (12.8, 16.8)	17.3 (15.5, 19.2)	20.1 (18.2, 22.2)	18.1 (16.0, 20.4)	15.8 (12.7, 18.9)	16.5 (14.7, 18.3)	19.0 (17.4, 20.6)	21.8 (20.1, 23.7)
urban	16.9 (14.6, 19.2)	14.7 (11.5, 18.0)	15.3 (13.3, 17.3)	17.8 (15.9, 19.7)	20.5 (18.5, 22.5)	18.3 (16.1, 20.5)	16.1 (13.1, 19.2)	16.7 (14.9, 18.5)	19.2 (17.5, 20.8)	21.9 (20.1, 23.7)
urban-rural difference	0.5 (-0.3, 1.3)	0.6 (-0.4, 1.7)	0.5 (-0.3, 1.4)	0.4 (-0.3, 1.2)	0.4 (-0.5, 1.2)	0.2 (-0.5, 0.9)	0.3 (-0.6, 1.3)	0.2 (-0.5, 1.0)	0.1 (-0.5, 0.8)	0.1 (-0.7, 0.8)
Liberia										
rural	16.2 (14.4, 18.0)	14.1 (11.0, 17.2)	14.9 (13.1, 16.7)	17.4 (15.7, 19.1)	19.9 (18.1, 21.6)	17.9 (16.6, 19.2)	15.8 (13.0, 18.8)	16.6 (15.3, 18.0)	19.1 (18.0, 20.2)	21.6 (20.4, 22.7)
urban	16.6 (14.9, 18.5)	14.7 (11.6, 17.8)	15.4 (13.6, 17.2)	17.7 (16.1, 19.4)	20.1 (18.4, 21.8)	18.0 (16.7, 19.3)	16.1 (13.2, 19.0)	16.8 (15.4, 18.2)	19.1 (18.0, 20.3)	21.5 (20.3, 22.7)
urban-rural difference	0.4 (-0.3, 1.2)	0.6 (-0.4, 1.6)	0.5 (-0.3, 1.3)	0.4 (-0.3, 1.1)	0.3 (-0.5, 1.1)	0.1 (-0.5, 0.7)	0.3 (-0.7, 1.2)	0.1 (-0.5, 0.8)	0.0 (-0.5, 0.6)	-0.1 (-0.7, 0.5)
Mali										
rural	16.5 (14.8, 18.2)	14.4 (11.2, 17.5)	15.4 (13.6, 17.1)	17.8 (16.3, 19.3)	19.5 (17.9, 21.1)	18.4 (16.9, 20.0)	16.2 (13.2, 19.2)	17.3 (15.7, 18.9)	19.7 (18.3, 21.2)	21.4 (20.0, 22.9)
urban	16.9 (15.1, 18.6)	14.9 (11.7, 18.0)	15.8 (14.0, 17.6)	18.1 (16.5, 19.7)	19.7 (18.0, 21.5)	18.4 (16.9, 19.9)	16.4 (13.3, 19.4)	17.3 (15.8, 18.9)	19.6 (18.3, 21.1)	21.2 (19.8, 22.7)
urban-rural difference	0.4 (-0.4, 1.2)	0.5 (-0.5, 1.6)	0.4 (-0.4, 1.3)	0.3 (-0.5, 1.1)	0.2 (-0.6, 1.0)	0.0 (-0.7, 0.7)	0.2 (-0.8, 1.1)	0.1 (-0.7, 0.8)	-0.1 (-0.7, 0.6)	-0.2 (-0.9, 0.5)
Mauritania										
rural	16.6 (14.5, 18.6)	14.6 (11.3, 17.8)	15.3 (13.2, 17.3)	17.7 (15.8, 19.6)	20.4 (18.2, 22.5)	18.3 (16.6, 20.2)	16.3 (13.2, 19.4)	17.0 (15.2, 19.0)	19.5 (17.8, 21.2)	22.1 (20.2, 24.2)
urban	17.1 (15.0, 19.0)	15.2 (11.9, 18.4)	15.8 (13.7, 17.8)	18.2 (16.3, 20.0)	20.8 (18.7, 22.9)	18.5 (16.8, 20.4)	16.6 (13.6, 19.8)	17.3 (15.4, 19.1)	19.6 (17.9, 21.3)	22.2 (20.3, 24.2)
urban-rural difference	0.5 (-0.3, 1.3)	0.6 (-0.4, 1.7)	0.5 (-0.3, 1.4)	0.5 (-0.3, 1.3)	0.4 (-0.4, 1.3)	0.2 (-0.5, 0.9)	0.3 (-0.6, 1.3)	0.2 (-0.5, 1.0)	0.1 (-0.5, 0.8)	0.1 (-0.7, 0.8)
Niger										
rural	15.7 (13.9, 17.6)	13.6 (10.3, 16.8)	14.6 (12.7, 16.5)	17.1 (15.3, 18.8)	18.6 (16.8, 20.4)	17.5 (15.8, 19.1)	15.3 (12.2, 18.4)	16.3 (14.7, 18.0)	18.8 (17.3, 20.3)	20.3 (18.7, 22.0)
urban	16.3 (14.4, 18.2)	14.2 (10.8, 17.5)	15.2 (13.2, 17.1)	17.5 (15.8, 19.3)	19.0 (17.1, 20.9)	17.7 (16.0, 19.4)	15.6 (12.4, 18.7)	16.6 (14.8, 18.3)	19.0 (17.4, 20.5)	20.4 (18.8, 22.1)
urban-rural difference	0.5 (-0.3, 1.3)	0.6 (-0.4, 1.7)	0.5 (-0.3, 1.4)	0.5 (-0.3, 1.3)	0.4 (-0.5, 1.3)	0.2 (-0.5, 0.9)	0.3 (-0.7, 1.3)	0.2 (-0.5, 1.0)	0.2 (-0.5, 0.8)	0.1 (-0.6, 0.8)
Nigeria										
rural	15.8 (14.2, 17.3)	13.8 (12.2, 15.4)	14.6 (13.0, 16.2)	16.9 (15.3, 18.5)	19.0 (17.4, 20.7)	17.5 (16.5, 18.4)	15.5 (14.5, 16.5)	16.3 (15.4, 17.2)	18.6 (17.7, 19.6)	20.8 (19.8, 21.7)
urban	16.2 (14.6, 17.8)	14.4 (12.8, 16.2)	15.1 (13.4, 16.7)	17.2 (15.6, 18.9)	19.3 (17.6, 21.0)	17.6 (16.6, 18.5)	15.8 (14.7, 16.9)	16.4 (15.4, 17.5)	18.6 (17.6, 19.6)	20.6 (19.6, 21.7)
urban-rural difference	0.4 (-0.3, 1.2)	0.6 (-0.2, 1.6)	0.5 (-0.3, 1.3)	0.3 (-0.4, 1.1)	0.2 (-0.6, 1.0)	0.1 (-0.5, 0.7)	0.3 (-0.5, 1.1)	0.2 (-0.5, 0.8)	0.0 (-0.6, 0.6)	-0.1 (-0.8, 0.5)
Sao Tome and Principe										
rural	17.0 (15.2, 18.8)	14.9 (11.7, 18.0)	15.7 (13.9, 17.5)	18.3 (16.6, 19.9)	20.4 (18.8, 22.1)	18.6 (17.4, 19.7)	16.4 (13.6, 19.3)	17.3 (16.0, 18.6)	19.8 (18.8, 20.8)	22.0 (21.1, 22.9)
urban	17.5 (15.8, 19.3)	15.5 (12.4, 18.7)	16.3 (14.5, 18.1)	18.7 (17.1, 20.3)	20.9 (19.2, 22.5)	18.8 (17.6, 20.0)	16.8 (14.0, 19.7)	17.5 (16.3, 18.8)	20.0 (19.0, 21.0)	22.1 (21.2, 23.1)
urban-rural difference	0.5 (-0.2, 1.3)	0.7 (-0.3, 1.7)	0.6 (-0.2, 1.4)	0.5 (-0.2, 1.2)	0.4 (-0.4, 1.2)	0.2 (-0.4, 0.8)	0.4 (-0.6, 1.3)	0.3 (-0.4, 0.9)	0.2 (-0.4, 0.7)	0.1 (-0.5, 0.7)

Boys	Mean BMI in 1990 (kg/m^2)					Mean BMI in 2020 (kg/m^2)				
	Age-standardised	5 years	10 years	15 years	19 years	Age-standardised	5 years	10 years	15 years	19 years
Senegal										
rural	15.2 (13.5, 17.0)	13.1 (10.1, 16.2)	14.0 (12.2, 15.8)	16.4 (14.8, 18.1)	18.6 (16.9, 20.4)	16.9 (15.4, 18.3)	14.8 (11.9, 17.7)	15.6 (14.2, 17.1)	18.1 (16.8, 19.3)	20.3 (19.0, 21.6)
urban	15.7 (13.9, 17.5)	13.7 (10.7, 16.8)	14.5 (12.7, 16.4)	16.8 (15.2, 18.5)	19.0 (17.3, 20.7)	17.0 (15.6, 18.5)	15.1 (12.2, 17.9)	15.8 (14.3, 17.3)	18.2 (16.9, 19.5)	20.3 (19.0, 21.7)
urban-rural difference	0.5 (-0.2, 1.2)	0.6 (-0.4, 1.6)	0.5 (-0.2, 1.3)	0.4 (-0.2, 1.2)	0.4 (-0.4, 1.1)	0.2 (-0.4, 0.8)	0.3 (-0.6, 1.2)	0.2 (-0.5, 0.9)	0.1 (-0.4, 0.7)	0.1 (-0.5, 0.6)
Sierra Leone										
rural	16.5 (14.7, 18.3)	14.3 (11.2, 17.4)	15.4 (13.6, 17.2)	17.8 (16.1, 19.4)	19.4 (17.8, 21.1)	18.3 (17.3, 19.2)	16.1 (13.3, 18.8)	17.1 (16.1, 18.2)	19.5 (18.9, 20.2)	21.2 (20.5, 21.9)
urban	17.0 (15.3, 18.8)	15.0 (11.9, 18.1)	16.0 (14.2, 17.8)	18.3 (16.7, 19.9)	19.9 (18.2, 21.5)	18.5 (17.6, 19.5)	16.5 (13.7, 19.2)	17.4 (16.4, 18.5)	19.8 (19.1, 20.4)	21.4 (20.6, 22.1)
urban-rural difference	0.6 (-0.2, 1.3)	0.7 (-0.3, 1.7)	0.6 (-0.2, 1.4)	0.5 (-0.2, 1.3)	0.4 (-0.3, 1.3)	0.3 (-0.3, 0.8)	0.4 (-0.5, 1.3)	0.3 (-0.3, 0.9)	0.2 (-0.2, 0.7)	0.2 (-0.3, 0.7)
Togo										
rural	16.7 (14.9, 18.5)	14.6 (11.4, 17.7)	15.5 (13.7, 17.4)	18.0 (16.3, 19.6)	19.9 (18.1, 21.7)	18.5 (17.0, 19.9)	16.3 (13.5, 19.3)	17.3 (15.8, 18.8)	19.7 (18.4, 21.1)	21.6 (20.2, 23.1)
urban	17.2 (15.5, 19.0)	15.2 (12.0, 18.4)	16.1 (14.3, 17.9)	18.4 (16.8, 20.1)	20.2 (18.5, 22.0)	18.7 (17.2, 20.2)	16.7 (13.8, 19.7)	17.5 (16.0, 19.1)	19.9 (18.5, 21.2)	21.7 (20.2, 23.2)
urban-rural difference	0.5 (-0.3, 1.3)	0.6 (-0.4, 1.7)	0.5 (-0.3, 1.4)	0.4 (-0.3, 1.2)	0.4 (-0.5, 1.2)	0.2 (-0.5, 0.9)	0.3 (-0.7, 1.3)	0.2 (-0.5, 1.0)	0.1 (-0.5, 0.8)	0.1 (-0.7, 0.8)

Supplementary Figure 1. Number of height data sources, by region, year and sex.

(A) For each sex, the size of each circle shows the number of data sources for each region and year, and the colours indicate the relative count of national, subnational and community data sources. (B) For each sex, the size of each circle shows the number of data sources for each region and year, and the colours indicate whether the data sources covered rural areas, urban areas or both.

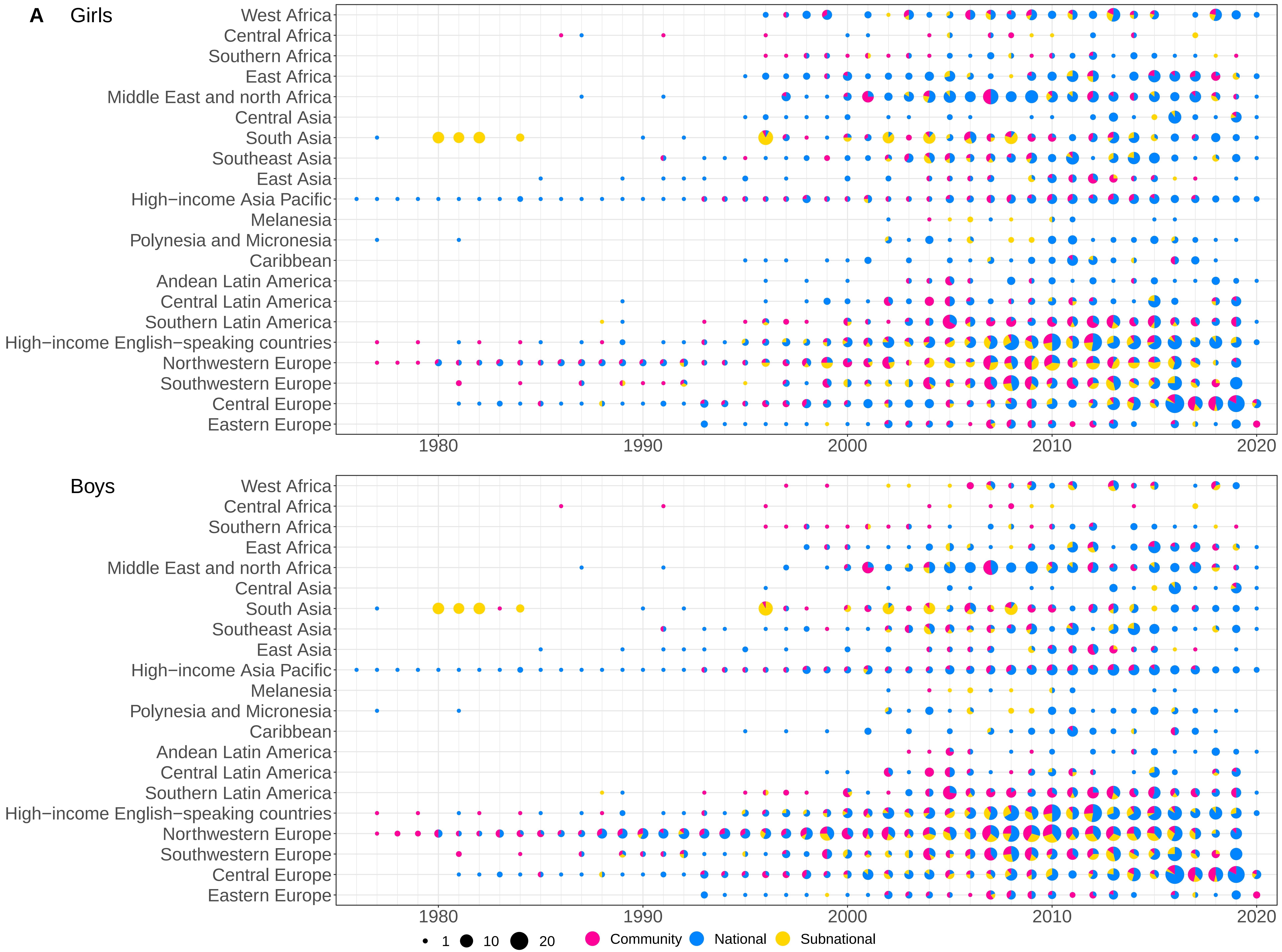
A
Girls
Boys
West Africa
Central Africa
Southern Africa
East Africa
Middle East and north Africa
Central Asia
South Asia
Southeast Asia
East Asia
High−income Asia Pacific
Melanesia
Polynesia and Micronesia
Caribbean
Andean Latin America
Central Latin America
Southern Latin America
High−income English−speaking countries
Northwestern Europe
Southwestern Europe
Central Europe
Eastern Europe
1980
1990
2000
2010
2020
1
10
20
Community
National
Subnational

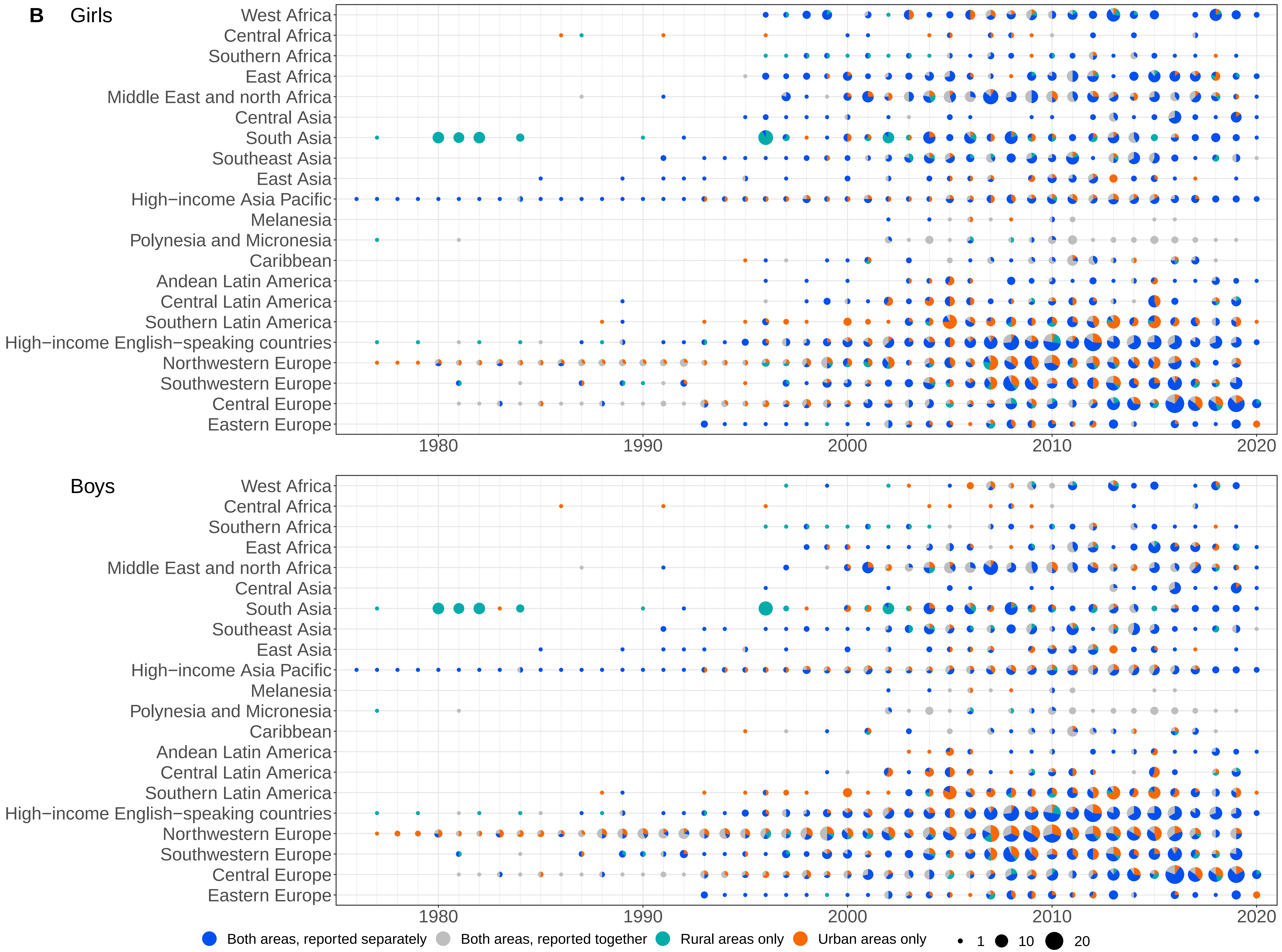

B
Girls
Boys
West Africa
Central Africa
Southern Africa
East Africa
Middle East and north Africa
Central Asia
South Asia
Southeast Asia
East Asia
High−income Asia Pacific
Melanesia
Polynesia and Micronesia
Caribbean
Andean Latin America
Central Latin America
Southern Latin America
High−income English−speaking countries
Northwestern Europe
Southwestern Europe
Central Europe
Eastern Europe
1980
1990
2000
2010
2020
Both areas, reported separately
Both areas, reported together
Rural areas only
Urban areas only
1
10
20

Supplementary Figure 2. Number of body-mass index (BMI) data sources, by region, year and sex.

(A) For each sex, the size of each circle shows the number of data sources for each region and year, and the colours indicate the relative count of national, subnational and community data sources. (B) For each sex, the size of each circle shows the number of data sources for each region and year, and the colours indicate whether the data sources covered rural areas, urban areas or both.

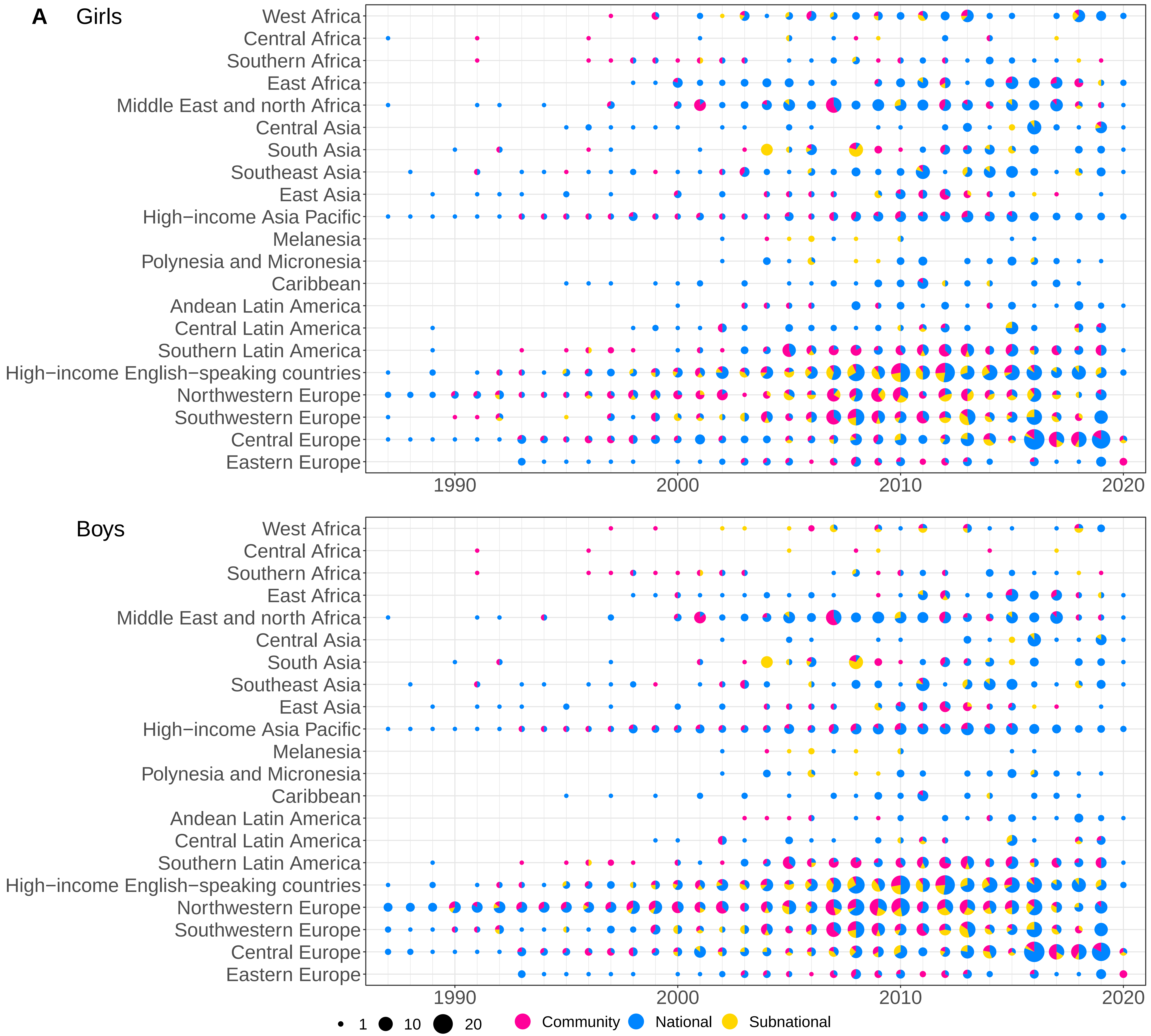
A
Girls
Boys
West Africa
Central Africa
Southern Africa
East Africa
Middle East and north Africa
Central Asia
South Asia
Southeast Asia
East Asia
High−income Asia Pacific
Melanesia
Polynesia and Micronesia
Caribbean
Andean Latin America
Central Latin America
Southern Latin America
High−income English−speaking countries
Northwestern Europe
Southwestern Europe
Central Europe
Eastern Europe
1990
2000
2010
2020
1
10
20
Community
National
Subnational

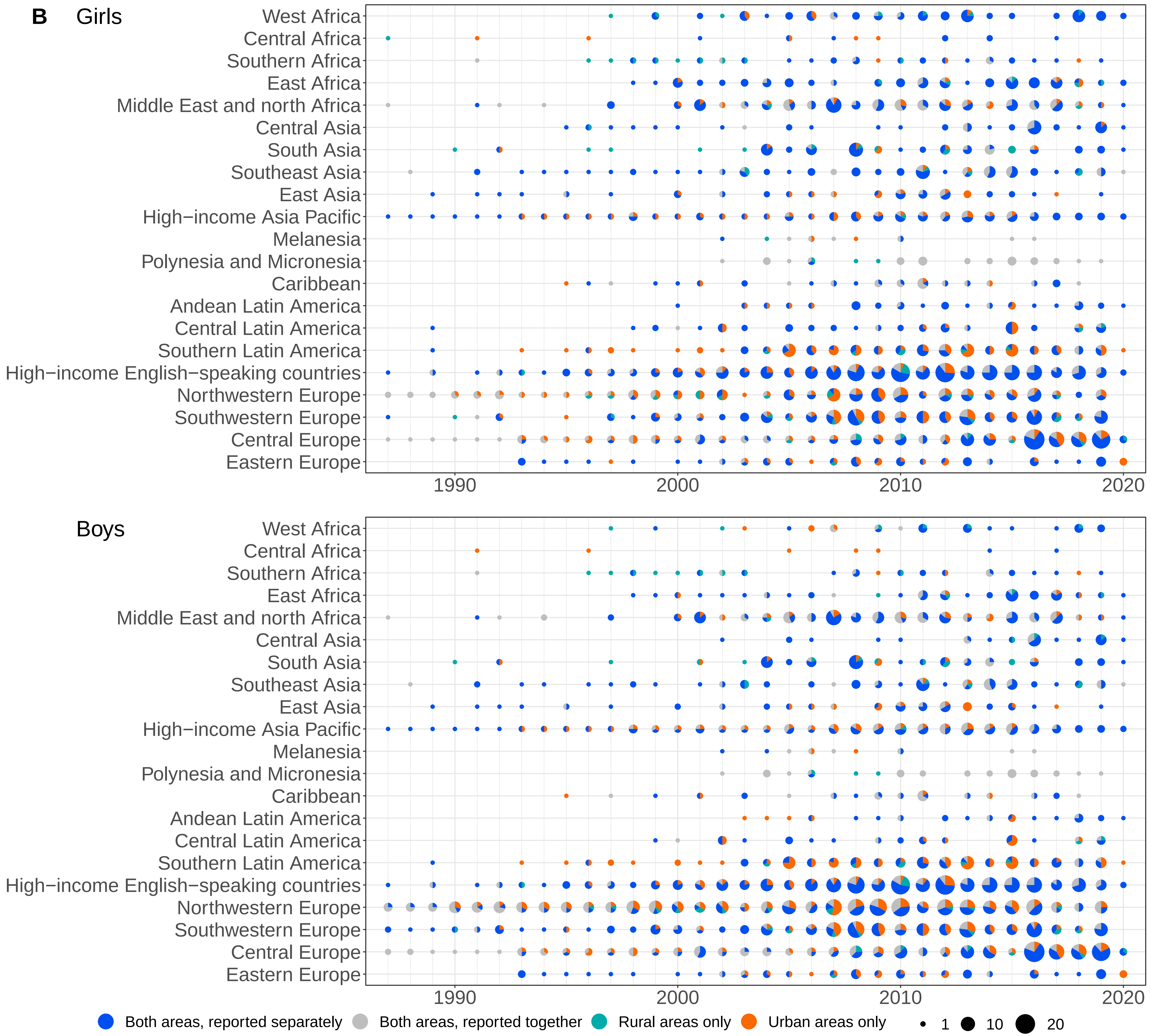
B
Girls
Boys
West Africa
Central Africa
Southern Africa
East Africa
Middle East and north Africa
Central Asia
South Asia
Southeast Asia
East Asia
High−income Asia Pacific
Melanesia
Polynesia and Micronesia
Caribbean
Andean Latin America
Central Latin America
Southern Latin America
High−income English−speaking countries
Northwestern Europe
Southwestern Europe
Central Europe
Eastern Europe
1990
2000
2010
2020
Both areas, reported separately
Both areas, reported together
Rural areas only
Urban areas only
1
10
20

Supplementary Figure 3. Comparison of urban-rural difference in height and body-mass index (BMI) in 2020, by sex.

We did not estimate the difference between rural and urban BMI or height for areas classified as entirely urban (Bermuda, Kuwait, Nauru and Singapore) or entirely rural (Tokelau).

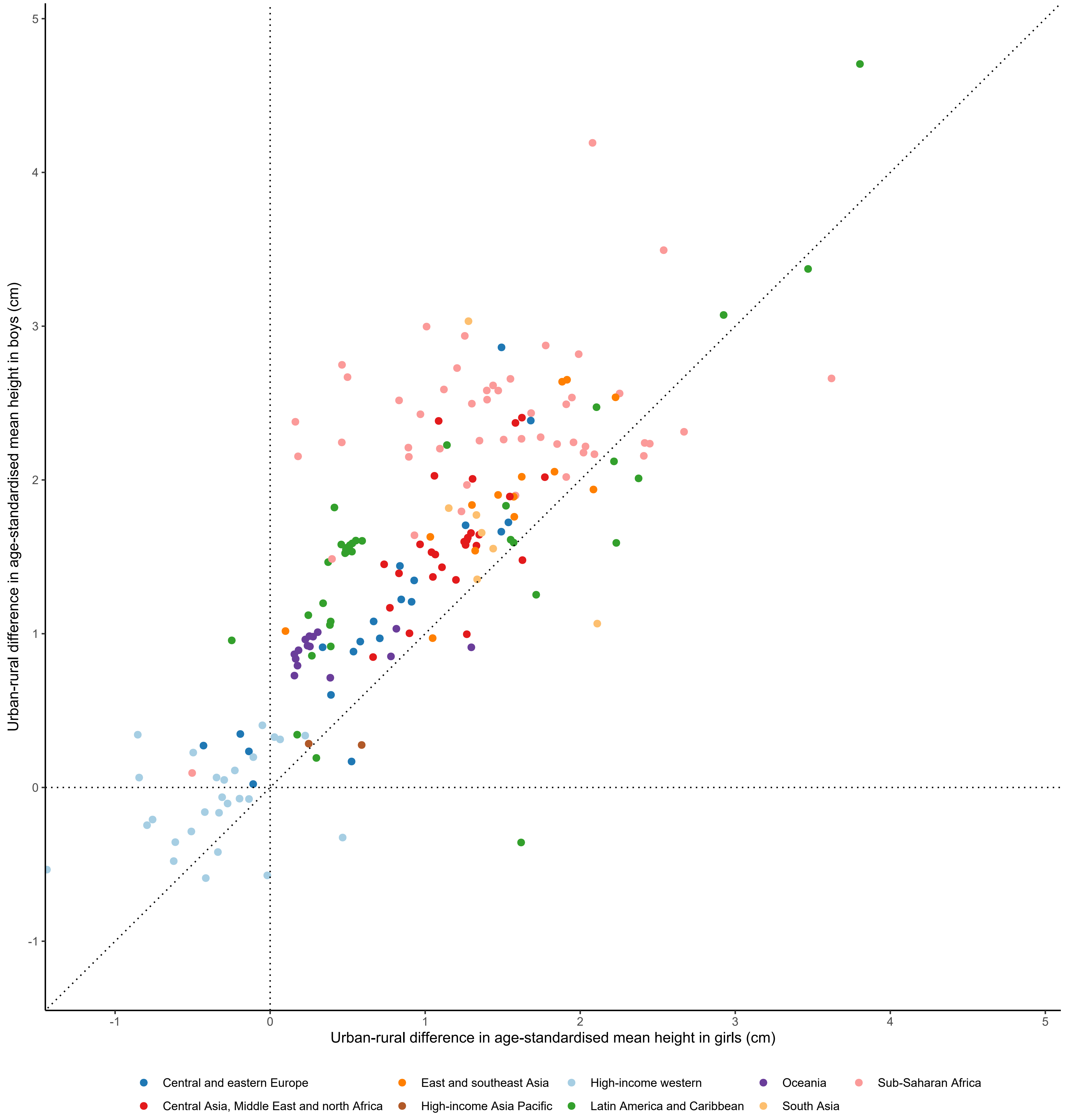
Urban-rural difference in age-standardised mean height in boys (cm)
Urban-rural difference in age-standardised mean height in girls (cm)
Central and eastern Europe
Central Asia, Middle East and north Africa
East and southeast Asia
High-income Asia Pacific
High-income western
Latin America and Caribbean
Oceania
South Asia
Sub-Saharan Africa

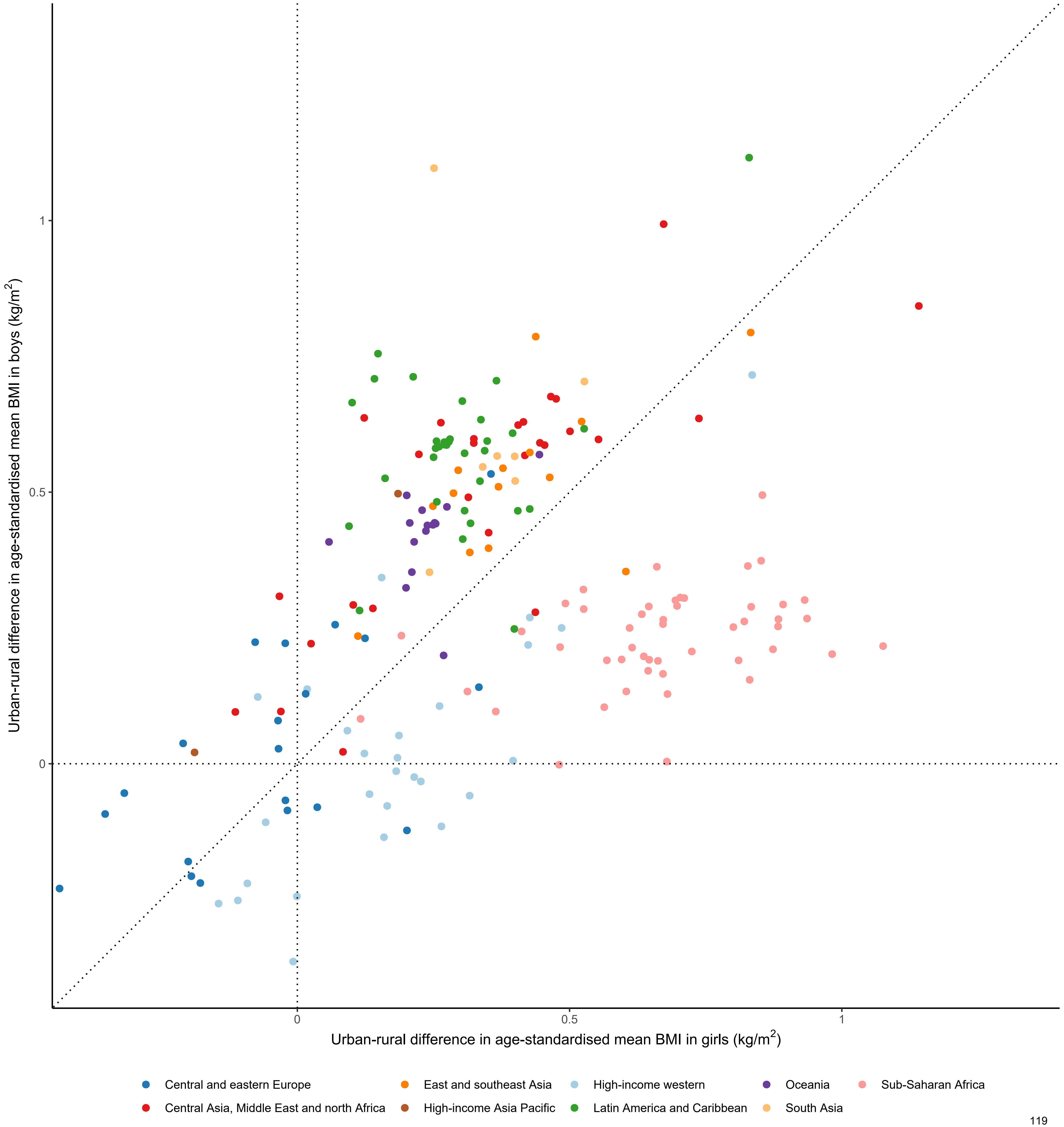
Urban-rural difference in age-standardised mean BMI in boys (kg/m²)
Urban-rural difference in age-standardised mean BMI in girls (kg/m²)
Central and eastern Europe
Central Asia, Middle East and north Africa
East and southeast Asia
High-income Asia Pacific
High-income western
Latin America and Caribbean
Oceania
South Asia
Sub-Saharan Africa

Supplementary Figure 4. Change in the urban-rural height and body-mass index (BMI) difference from 1990 to 2020, by age.

(A) Change in urban-rural difference in mean height in relation to change in mean rural height at ages 5, 10, 15 and 19 years. (B) Change in urban-rural difference in mean BMI in relation to change in mean BMI in at ages 5, 10, 15 and 19 years. (C) Change in urban-rural difference in mean height and urban-rural difference in mean BMI at ages 5, 10, 15 and 19 years.

Each solid arrow in lighter shading shows one country, beginning in 1990 and ending in 2020. The dashed arrows in darker shade show the regional averages, calculated as the unweighted arithmetic mean of the values for all countries in each region along the horizontal and vertical axes. For urban-rural difference, a positive number shows higher urban mean height and BMI and a negative number shows higher rural mean height and BMI. The dark vertical dashed lines indicate the median of the WHO reference population. For BMI, the lighter vertical dashed lines are placed one standard deviation below and above the median of the WHO reference population.

We did not estimate the difference between rural and urban height and BMI for areas classified as entirely urban (Bermuda, Kuwait, Nauru and Singapore) or entirely rural (Tokelau).

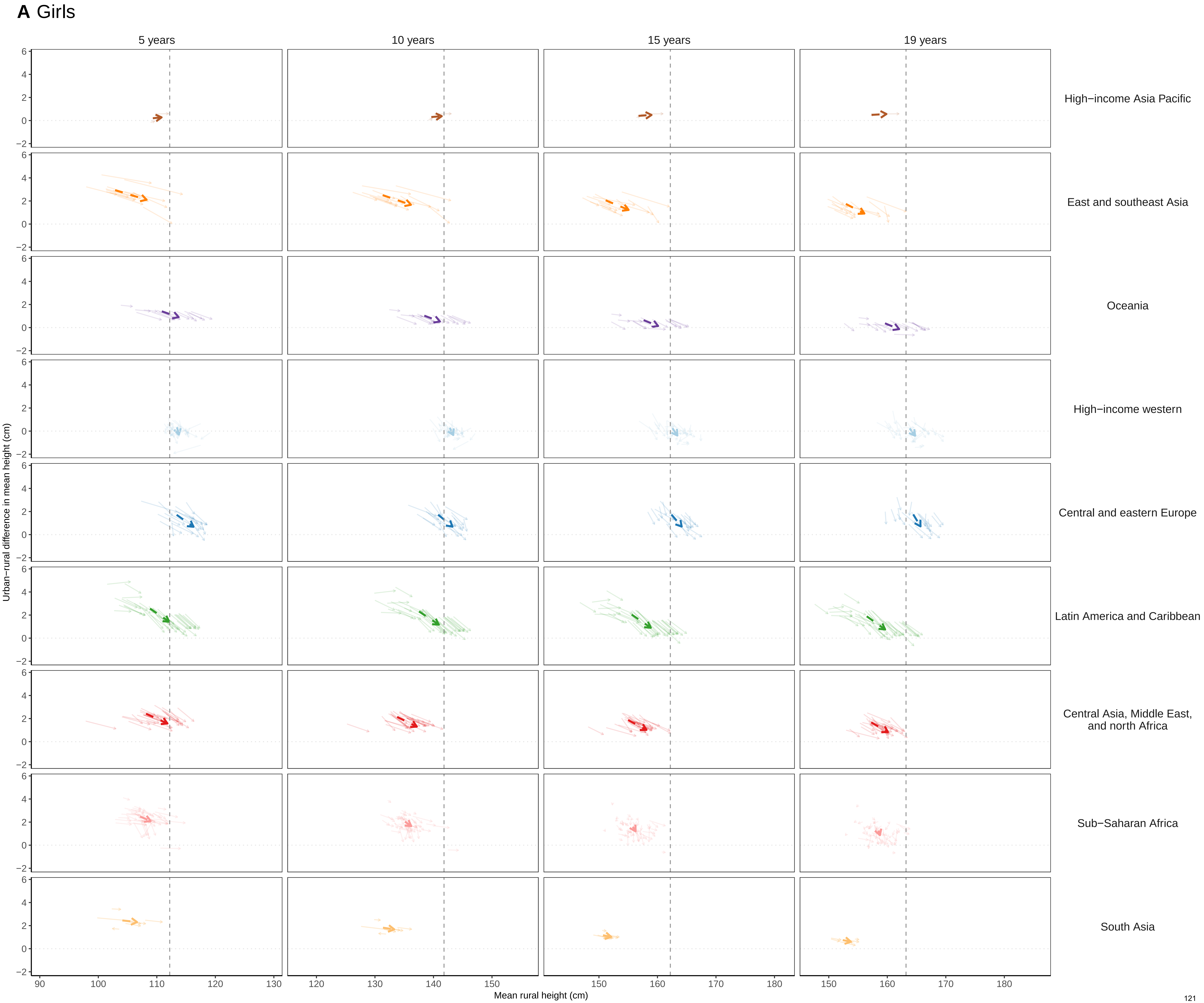

A Girls
5 years
10 years
15 years
19 years
High−income Asia Pacific
East and southeast Asia
Oceania
High−income western
Central and eastern Europe
Latin America and Caribbean
Central Asia, Middle East, and north Africa
Sub−Saharan Africa
South Asia
Urban−rural difference in mean height (cm)
Mean rural height (cm)

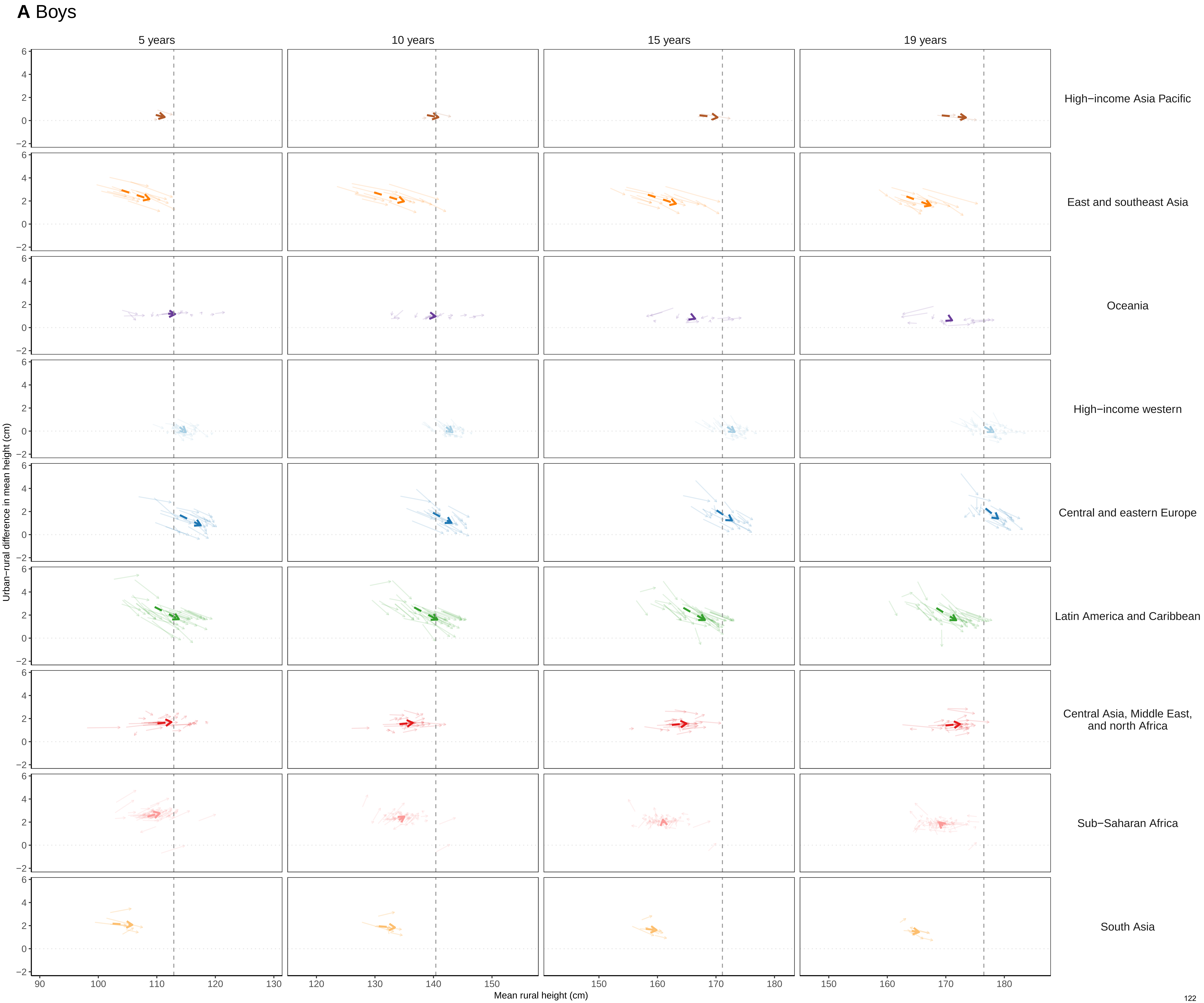

A Boys
5 years
10 years
15 years
19 years
High−income Asia Pacific
East and southeast Asia
Oceania
High−income western
Central and eastern Europe
Latin America and Caribbean
Central Asia, Middle East, and north Africa
Sub−Saharan Africa
South Asia
Urban−rural difference in mean height (cm)
Mean rural height (cm)
6
4
2
0
−2
90
100
110
120
130
140
150
160
170
180

B Girls

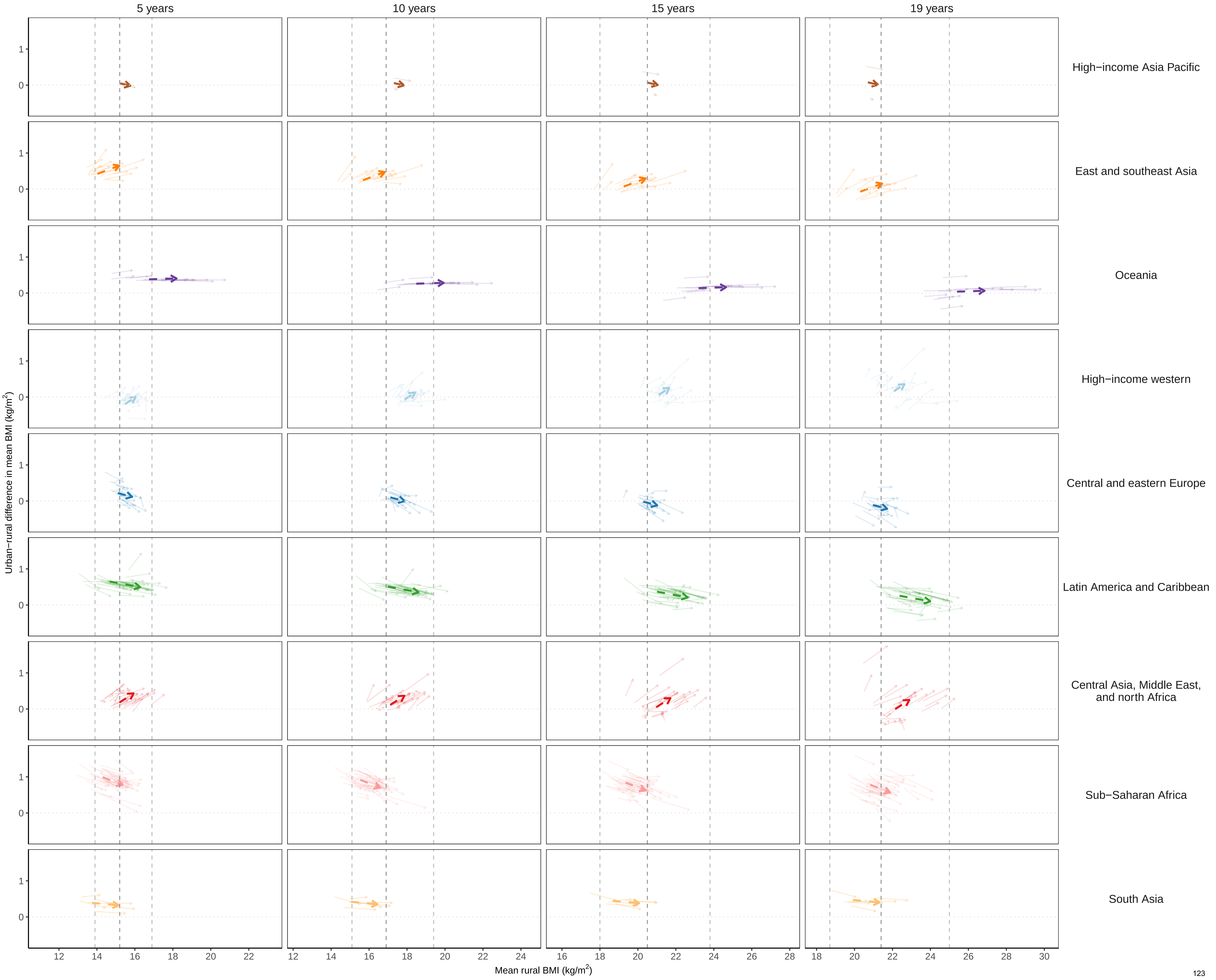

5 years
10 years
15 years
19 years
High−income Asia Pacific
East and southeast Asia
Oceania
High−income western
Central and eastern Europe
Latin America and Caribbean
Central Asia, Middle East, and north Africa
Sub−Saharan Africa
South Asia
Urban−rural difference in mean BMI (kg/m2)
Mean rural BMI (kg/m2)

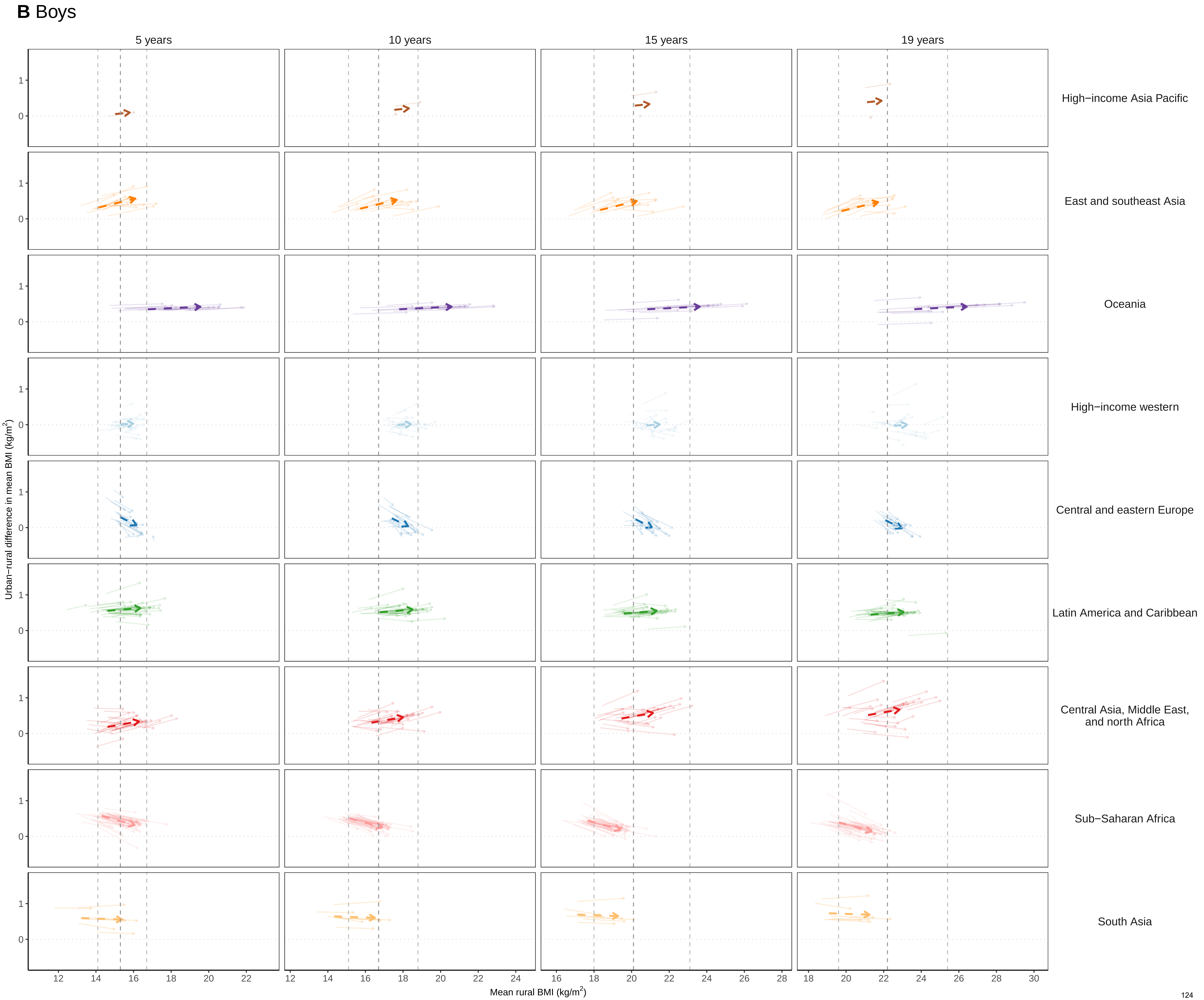
B Boys
5 years
10 years
15 years
19 years
High−income Asia Pacific
East and southeast Asia
Oceania
High−income western
Central and eastern Europe
Latin America and Caribbean
Central Asia, Middle East, and north Africa
Sub−Saharan Africa
South Asia
Urban−rural difference in mean BMI (kg/m2)
Mean rural BMI (kg/m2)
0
1
12
14
16
18
20
22
24
26
28
30

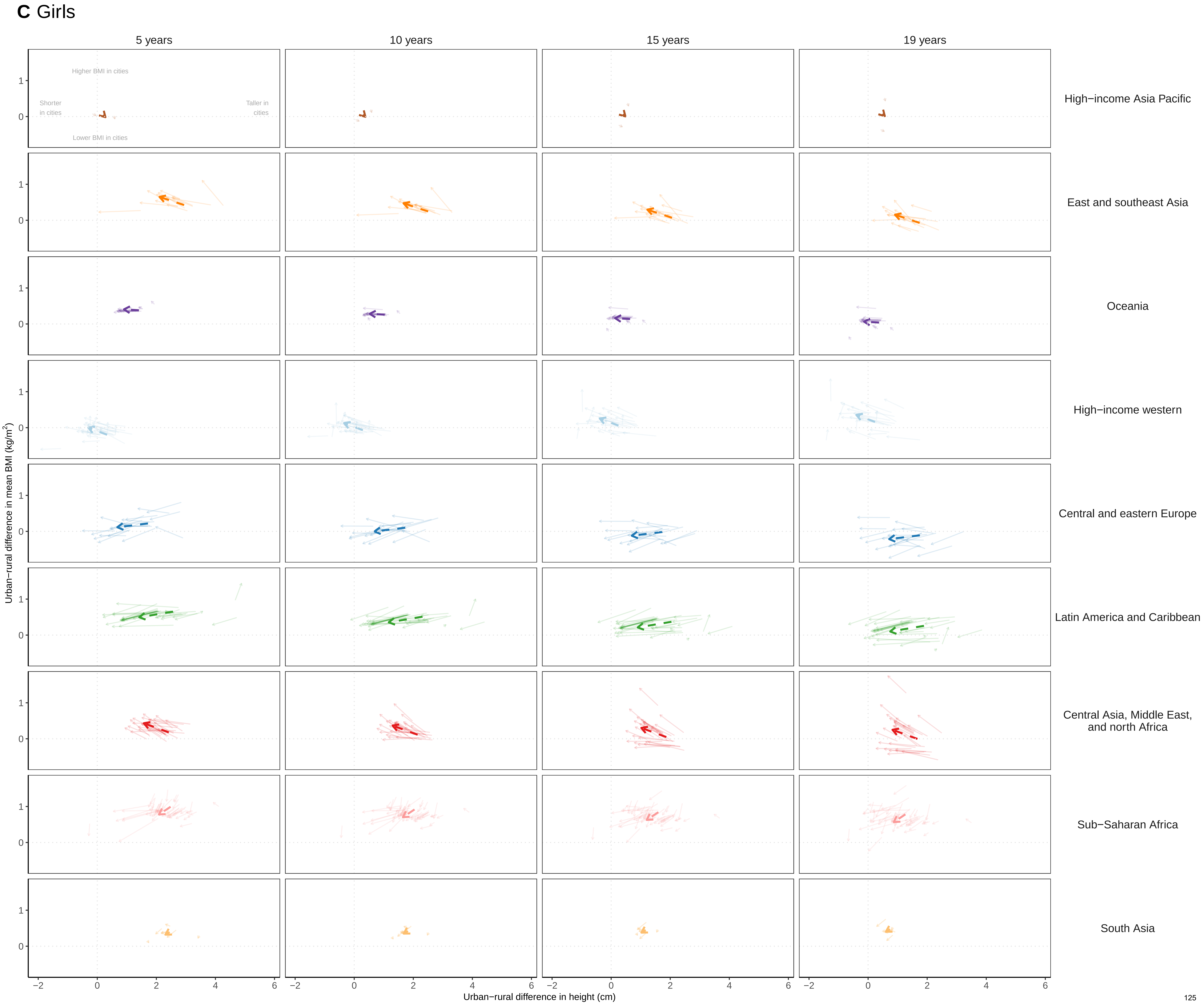
C Girls
5 years
10 years
15 years
19 years
Higher BMI in cities
Shorter in cities
Taller in cities
Lower BMI in cities
High−income Asia Pacific
East and southeast Asia
Oceania
High−income western
Central and eastern Europe
Latin America and Caribbean
Central Asia, Middle East, and north Africa
Sub−Saharan Africa
South Asia
Urban−rural difference in mean BMI (kg/m2)
Urban−rural difference in height (cm)
−2
0
2
4
6
1
0

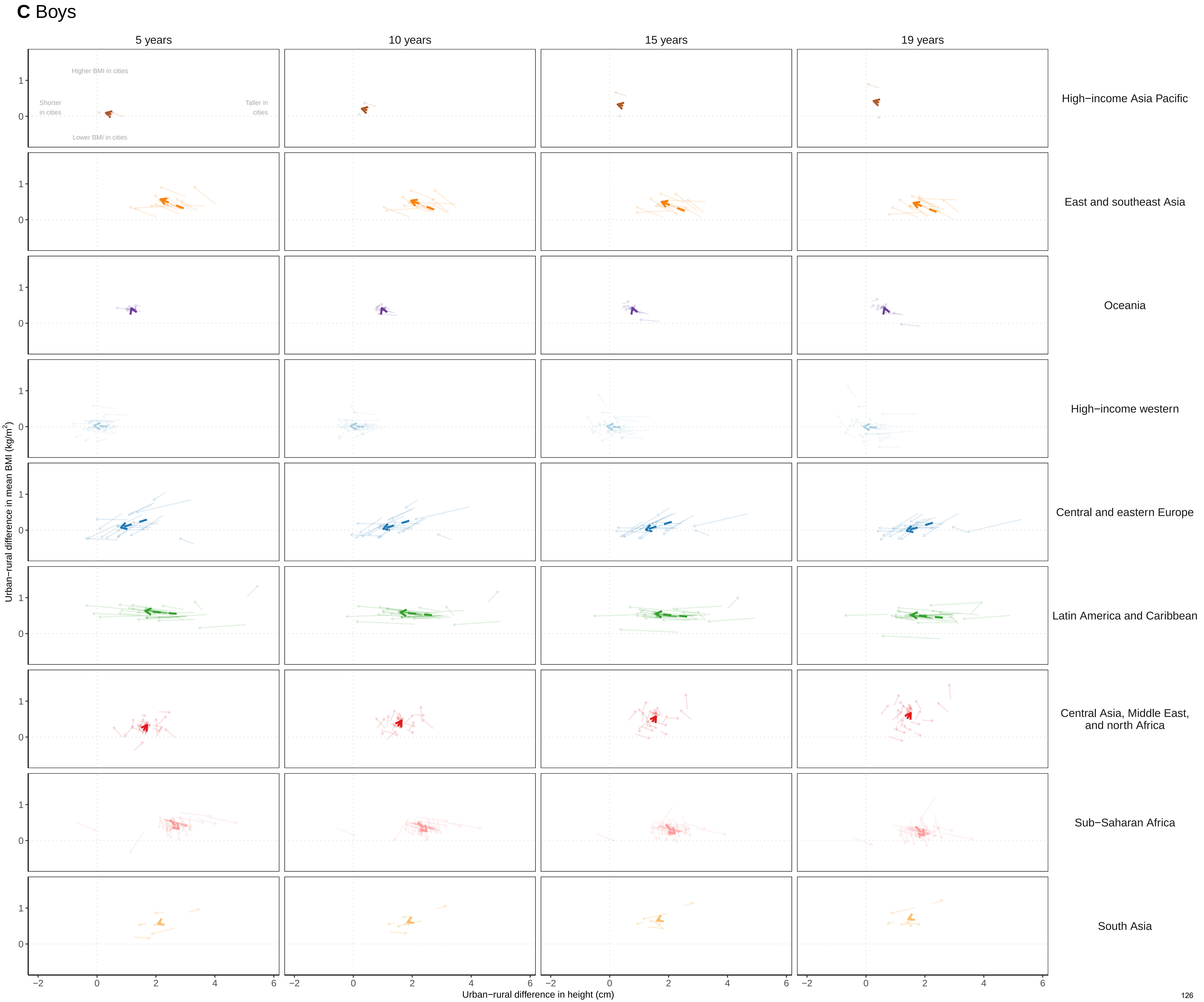

C Boys
5 years
10 years
15 years
19 years
Higher BMI in cities
Shorter in cities
Taller in cities
Lower BMI in cities
High−income Asia Pacific
East and southeast Asia
Oceania
High−income western
Central and eastern Europe
Latin America and Caribbean
Central Asia, Middle East, and north Africa
Sub−Saharan Africa
South Asia
Urban−rural difference in mean BMI (kg/m2)
Urban−rural difference in height (cm)
−2
0
2
4
6
1

Supplementary Figure 5. Urban and rural height in 2020 and change from 1990 to 2020, by age.

The maps show mean height in 2020 and change from 1990 to 2020, by urban and rural place of residence and country for girls and boys at ages 5, 10, 15 and 19 years. The density plot below each map shows the distribution of estimates across countries. The top right graph in each panel shows the mean height in 2020 for all countries. The height of each column is the posterior mean estimate shown together with its 95% credible interval. Countries are ordered by region and super-region. The bottom right graph in each panel shows the change from 1990 to 2020 in mean height in relation to the uncertainty of the change measured by posterior standard deviation. Each point shows one country. Shaded areas approximately show the posterior probability (PP) of an estimated change being a true increase or decrease. The PP of a decrease is one minus that of an increase. If an increase in mean height is statistically indistinguishable from a decrease, the PP of an increase and a decrease is 0.50. PPs closer to 0.50 indicate more uncertainty, those towards 1 indicate more certainty of change.

We did not estimate mean rural height in areas classified as entirely urban (Bermuda, Kuwait, Nauru and Singapore), mean urban height in areas classified as entirely rural (Tokelau), or their change over time in these areas, as indicated by grey colour.

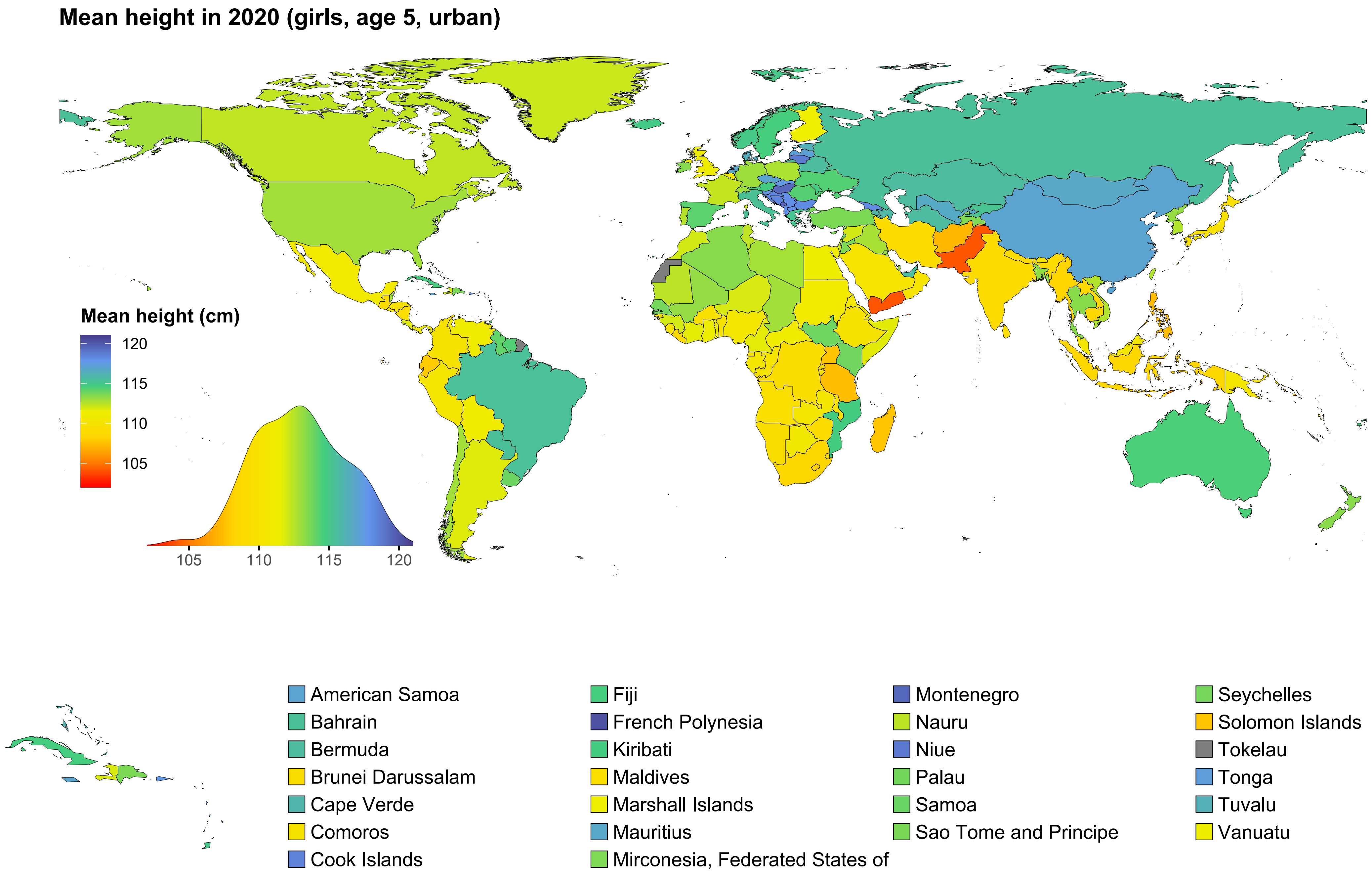
Mean height in 2020 (girls, age 5, urban)
Mean height (cm)
120
115
110
105
105 110 115 120
American Samoa
Bahrain
Bermuda
Brunei Darussalam
Cape Verde
Comoros
Cook Islands
Fiji
French Polynesia
Kiribati
Maldives
Marshall Islands
Mauritius
Mirconesia, Federated States of
Montenegro
Nauru
Niue
Palau
Samoa
Sao Tome and Principe
Seychelles
Solomon Islands
Tokelau
Tonga
Tuvalu
Vanuatu

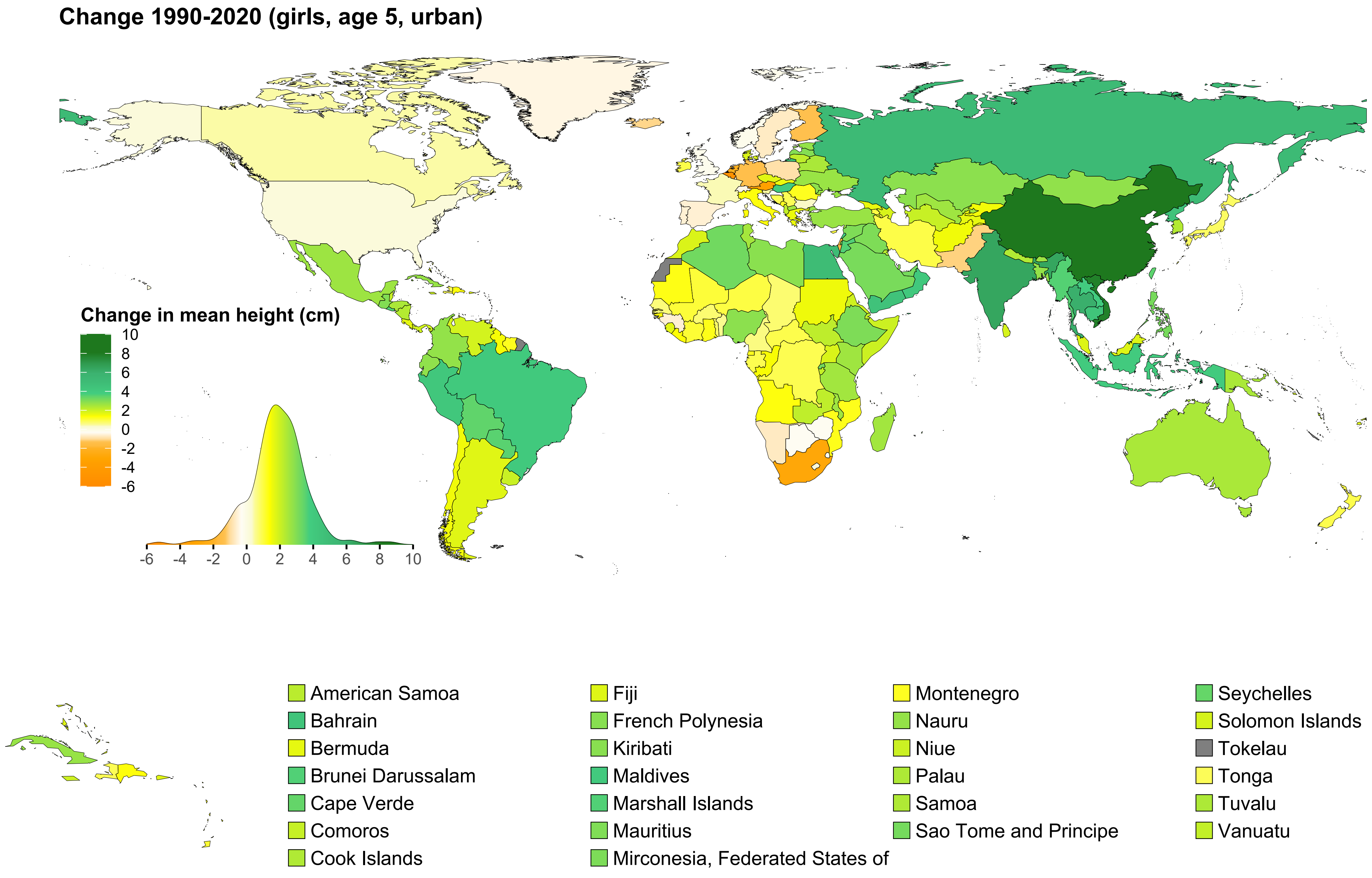
Change 1990-2020 (girls, age 5, urban)
Change in mean height (cm)
10
8
6
4
2
0
-2
-4
-6
-6 -4 -2 0 2 4 6 8 10
American Samoa
Bahrain
Bermuda
Brunei Darussalam
Cape Verde
Comoros
Cook Islands
Fiji
French Polynesia
Kiribati
Maldives
Marshall Islands
Mauritius
Mirconesia, Federated States of
Montenegro
Nauru
Niue
Palau
Samoa
Sao Tome and Principe
Seychelles
Solomon Islands
Tokelau
Tonga
Tuvalu
Vanuatu

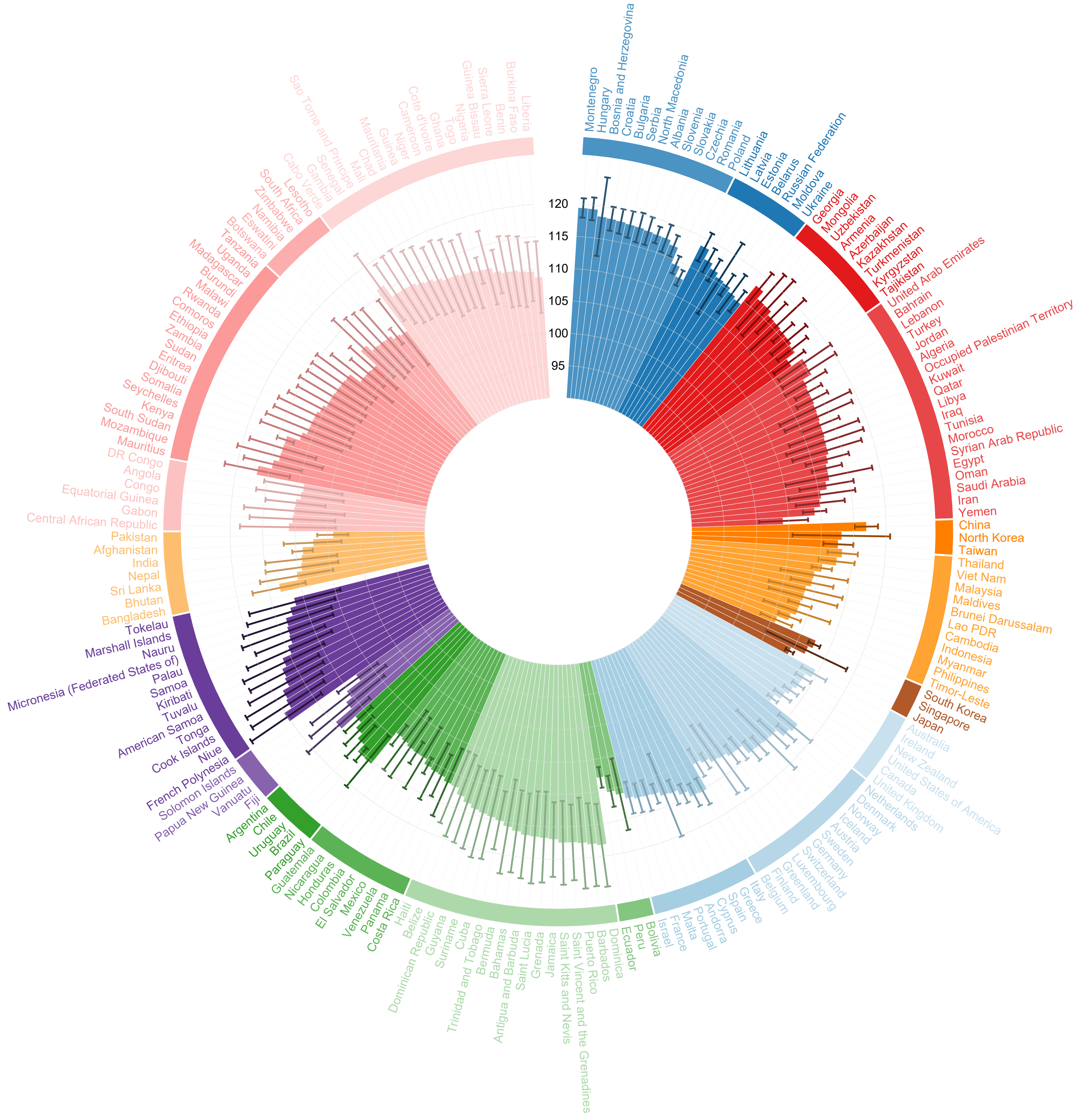
120
115
110
105
100
95

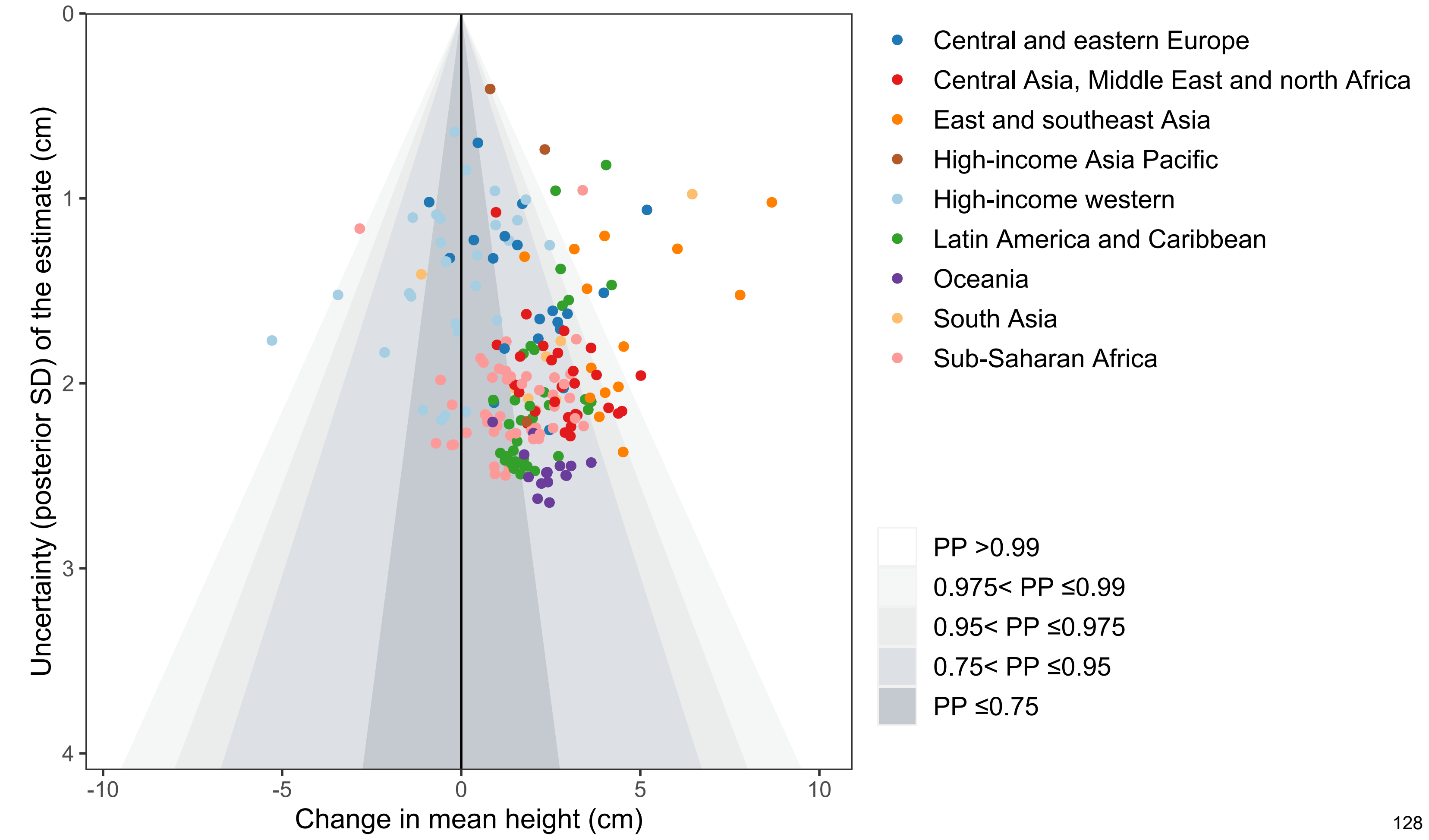
Uncertainty (posterior SD) of the estimate (cm)
Change in mean height (cm)
0
1
2
3
4
-10 -5 0 5 10
Central and eastern Europe
Central Asia, Middle East and north Africa
East and southeast Asia
High-income Asia Pacific
High-income western
Latin America and Caribbean
Oceania
South Asia
Sub-Saharan Africa
PP >0.99
0.975< PP ≤0.99
0.95< PP ≤0.975
0.75< PP ≤0.95
PP ≤0.75

Mean height in 2020 (girls, age 5, rural)

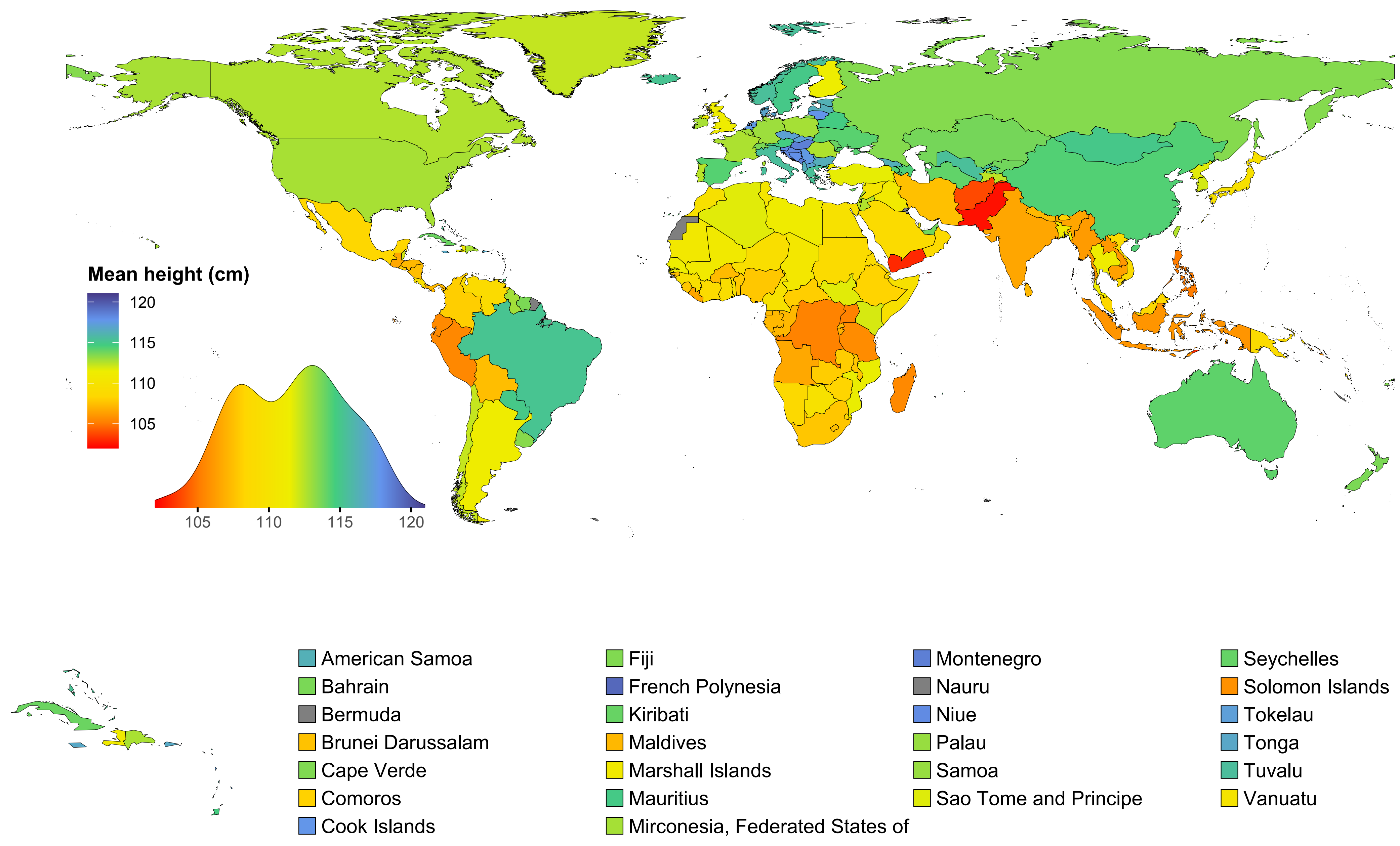

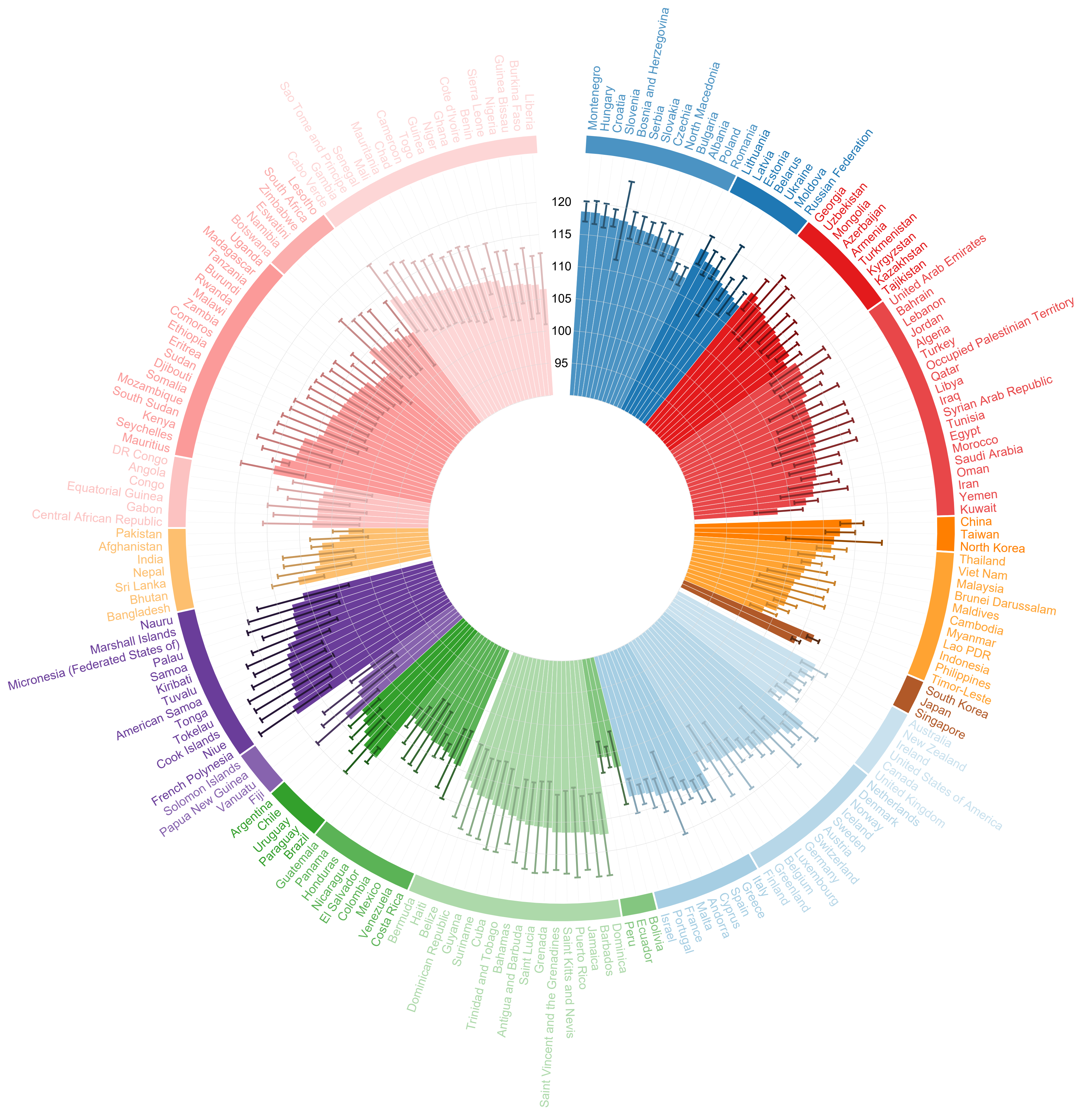

Change 1990-2020 (girls, age 5, rural)

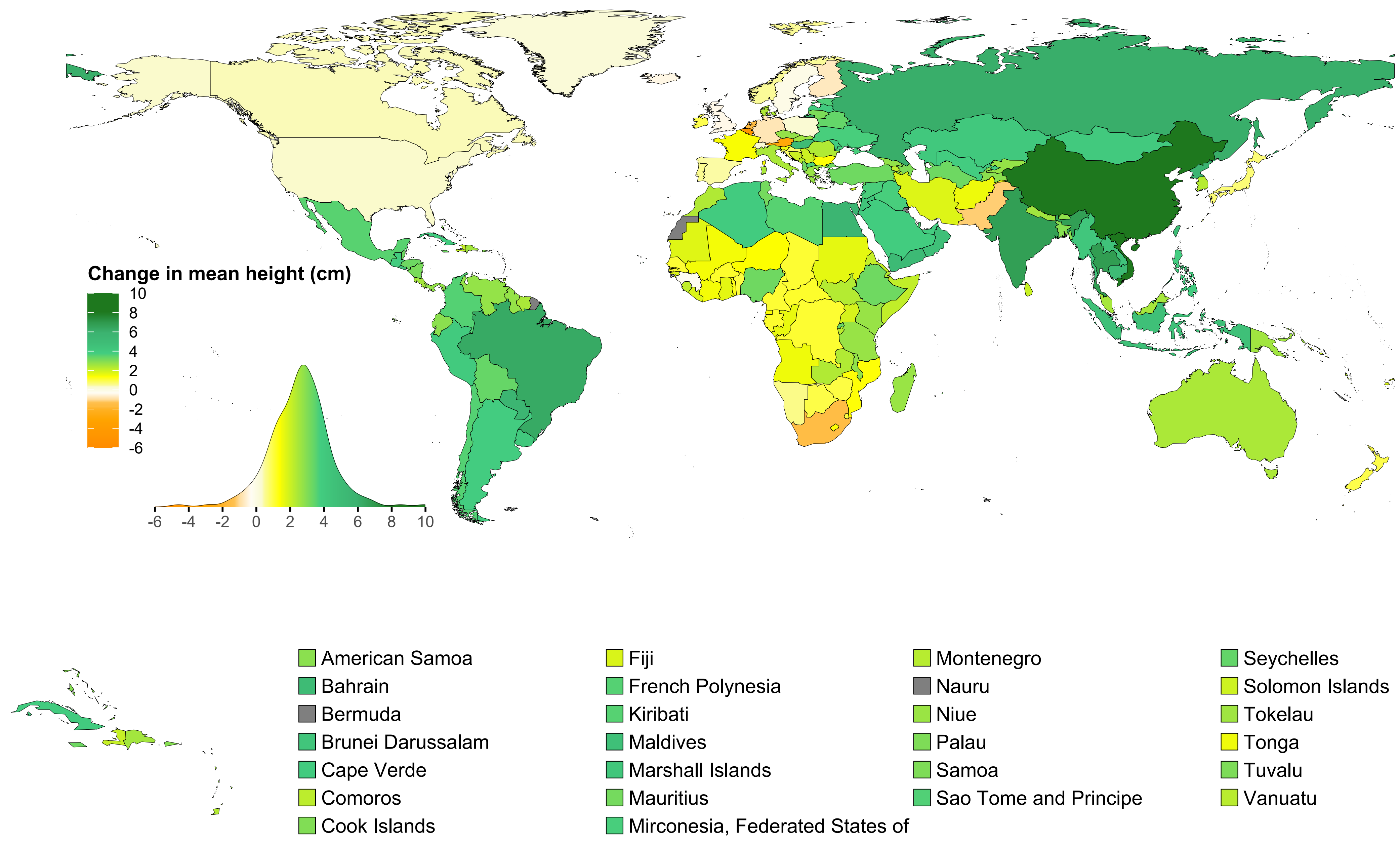

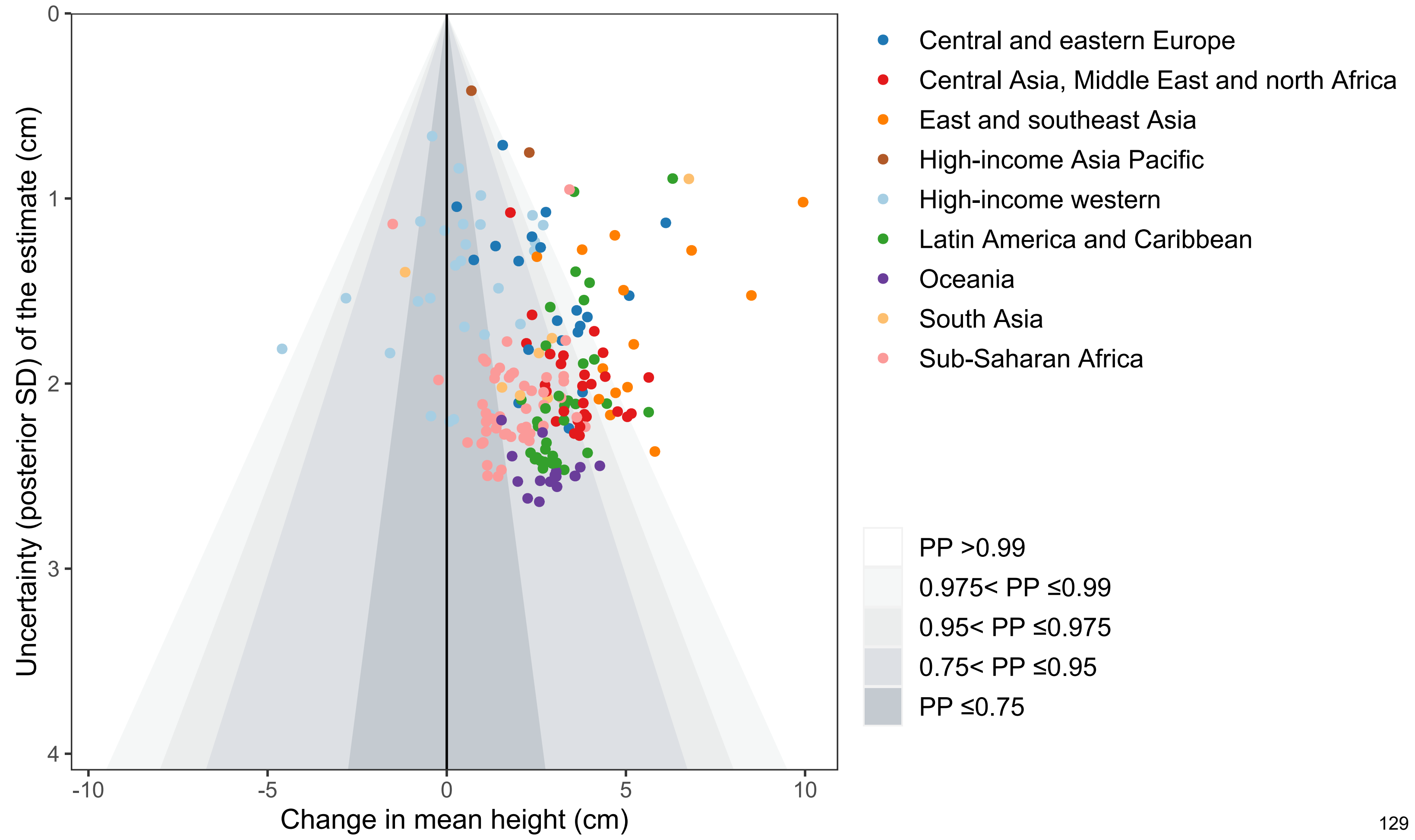

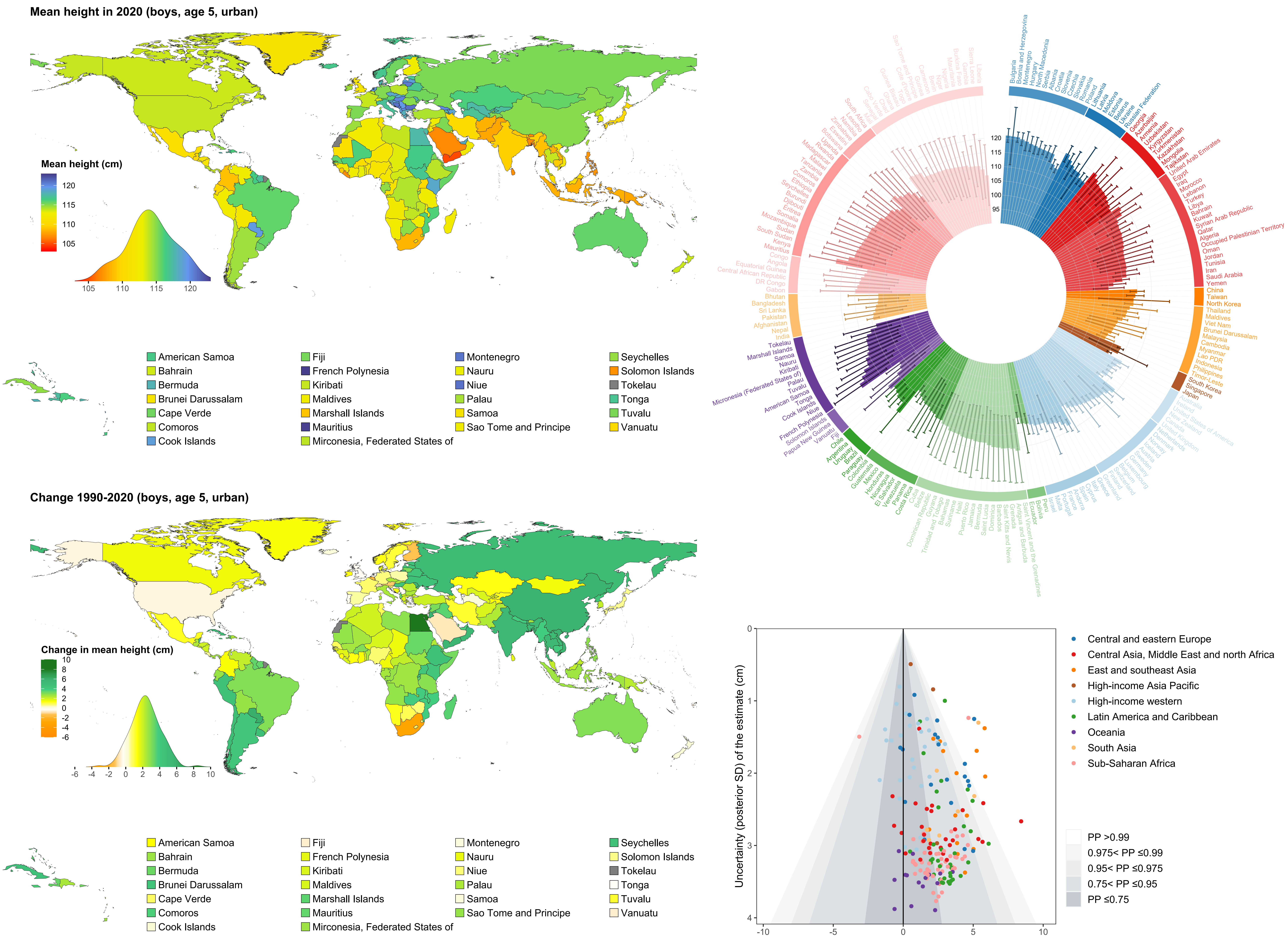
Mean height in 2020 (boys, age 5, urban)
Mean height (cm)
120
115
110
105
105 110 115 120
American Samoa
Bahrain
Bermuda
Brunei Darussalam
Cape Verde
Comoros
Cook Islands
Fiji
French Polynesia
Kiribati
Maldives
Marshall Islands
Mauritius
Mirconesia, Federated States of
Montenegro
Nauru
Niue
Palau
Samoa
Sao Tome and Principe
Seychelles
Solomon Islands
Tokelau
Tonga
Tuvalu
Vanuatu
Change 1990-2020 (boys, age 5, urban)
Change in mean height (cm)
10
8
6
4
2
0
-2
-4
-6
-6 -4 -2 0 2 4 6 8 10
American Samoa
Bahrain
Bermuda
Brunei Darussalam
Cape Verde
Comoros
Cook Islands
Fiji
French Polynesia
Kiribati
Maldives
Marshall Islands
Mauritius
Mirconesia, Federated States of
Montenegro
Nauru
Niue
Palau
Samoa
Sao Tome and Principe
Seychelles
Solomon Islands
Tokelau
Tonga
Tuvalu
Vanuatu
120
115
110
105
100
95
Uncertainty (posterior SD) of the estimate (cm)
Change in mean height (cm)
-10 -5 0 5 10
0 1 2 3 4
Central and eastern Europe
Central Asia, Middle East and north Africa
East and southeast Asia
High-income Asia Pacific
High-income western
Latin America and Caribbean
Oceania
South Asia
Sub-Saharan Africa
PP >0.99
0.975< PP ≤0.99
0.95< PP ≤0.975
0.75< PP ≤0.95
PP ≤0.75

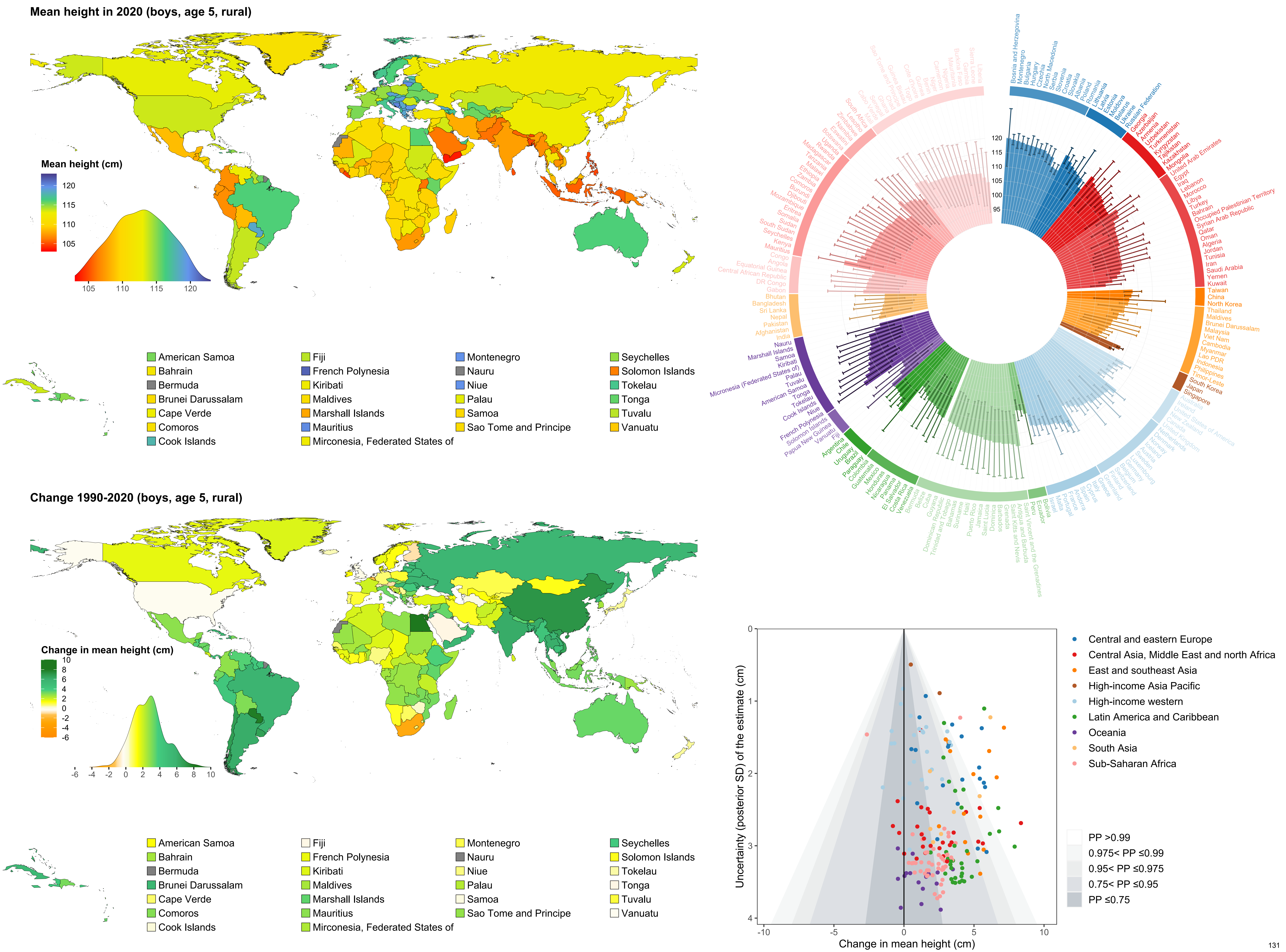
Mean height in 2020 (boys, age 5, rural)
Mean height (cm)
120
115
110
105
105 110 115 120
American Samoa
Bahrain
Bermuda
Brunei Darussalam
Cape Verde
Comoros
Cook Islands
Fiji
French Polynesia
Kiribati
Maldives
Marshall Islands
Mauritius
Mirconesia, Federated States of
Montenegro
Nauru
Niue
Palau
Samoa
Sao Tome and Principe
Seychelles
Solomon Islands
Tokelau
Tonga
Tuvalu
Vanuatu
Change 1990-2020 (boys, age 5, rural)
Change in mean height (cm)
10 8 6 4 2 0 -2 -4 -6
-6 -4 -2 0 2 4 6 8 10
American Samoa
Bahrain
Bermuda
Brunei Darussalam
Cape Verde
Comoros
Cook Islands
Fiji
French Polynesia
Kiribati
Maldives
Marshall Islands
Mauritius
Mirconesia, Federated States of
Montenegro
Nauru
Niue
Palau
Samoa
Sao Tome and Principe
Seychelles
Solomon Islands
Tokelau
Tonga
Tuvalu
Vanuatu
120 115 110 105 100 95
Uncertainty (posterior SD) of the estimate (cm)
Change in mean height (cm)
0 1 2 3 4
-10 -5 0 5 10
Central and eastern Europe
Central Asia, Middle East and north Africa
East and southeast Asia
High-income Asia Pacific
High-income western
Latin America and Caribbean
Oceania
South Asia
Sub-Saharan Africa
PP >0.99
0.975< PP ≤0.99
0.95< PP ≤0.975
0.75< PP ≤0.95
PP ≤0.75

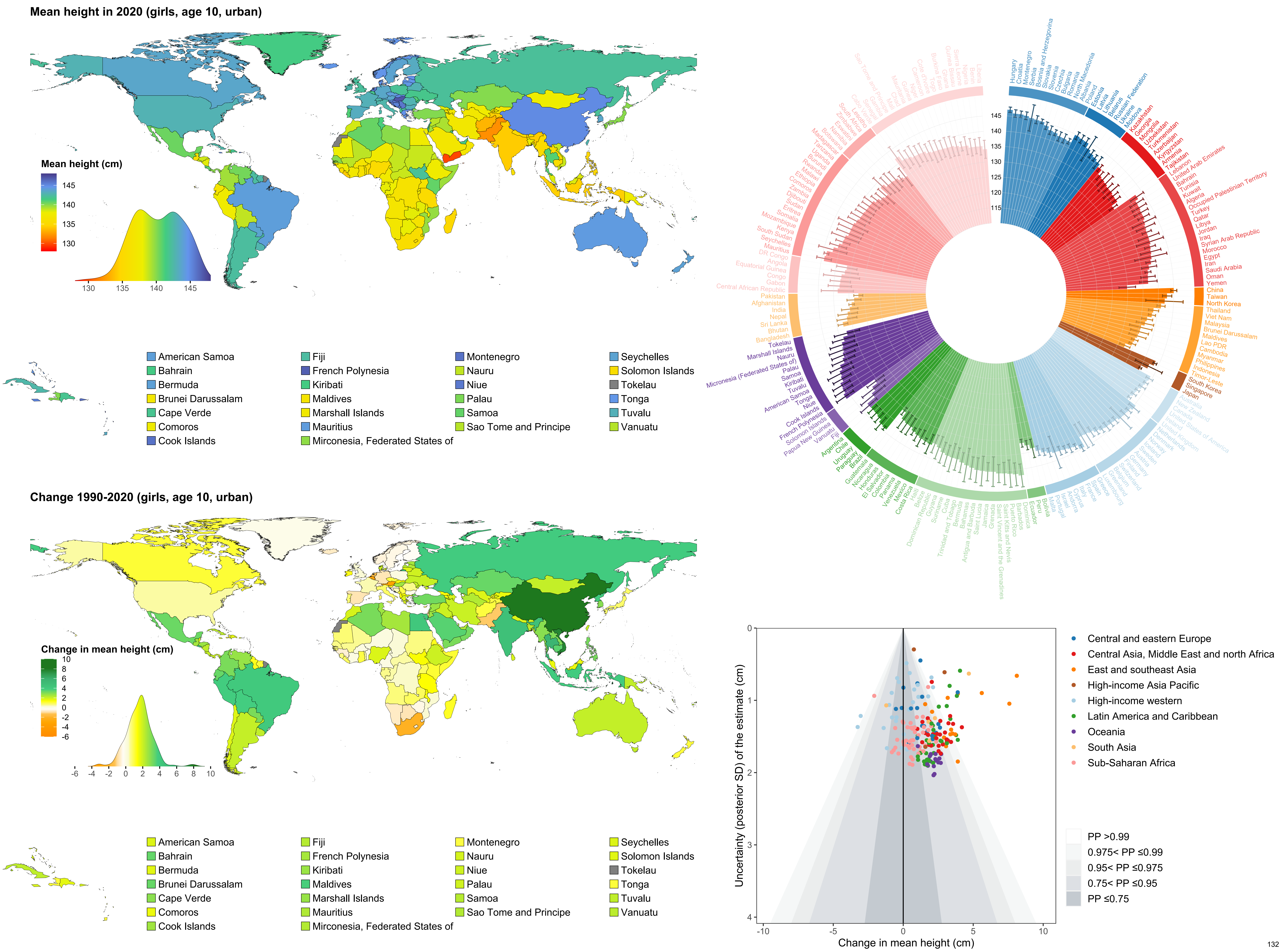
Mean height in 2020 (girls, age 10, urban)
Mean height (cm)
145
140
135
130
130 135 140 145
American Samoa
Bahrain
Bermuda
Brunei Darussalam
Cape Verde
Comoros
Cook Islands
Fiji
French Polynesia
Kiribati
Maldives
Marshall Islands
Mauritius
Mirconesia, Federated States of
Montenegro
Nauru
Niue
Palau
Samoa
Sao Tome and Principe
Seychelles
Solomon Islands
Tokelau
Tonga
Tuvalu
Vanuatu
Change 1990-2020 (girls, age 10, urban)
Change in mean height (cm)
10
8
6
4
2
0
-2
-4
-6
-6 -4 -2 0 2 4 6 8 10
American Samoa
Bahrain
Bermuda
Brunei Darussalam
Cape Verde
Comoros
Cook Islands
Fiji
French Polynesia
Kiribati
Maldives
Marshall Islands
Mauritius
Mirconesia, Federated States of
Montenegro
Nauru
Niue
Palau
Samoa
Sao Tome and Principe
Seychelles
Solomon Islands
Tokelau
Tonga
Tuvalu
Vanuatu
145
140
135
130
125
120
115
Uncertainty (posterior SD) of the estimate (cm)
Change in mean height (cm)
-10 -5 0 5 10
0 1 2 3 4
Central and eastern Europe
Central Asia, Middle East and north Africa
East and southeast Asia
High-income Asia Pacific
High-income western
Latin America and Caribbean
Oceania
South Asia
Sub-Saharan Africa
PP >0.99
0.975< PP ≤0.99
0.95< PP ≤0.975
0.75< PP ≤0.95
PP ≤0.75

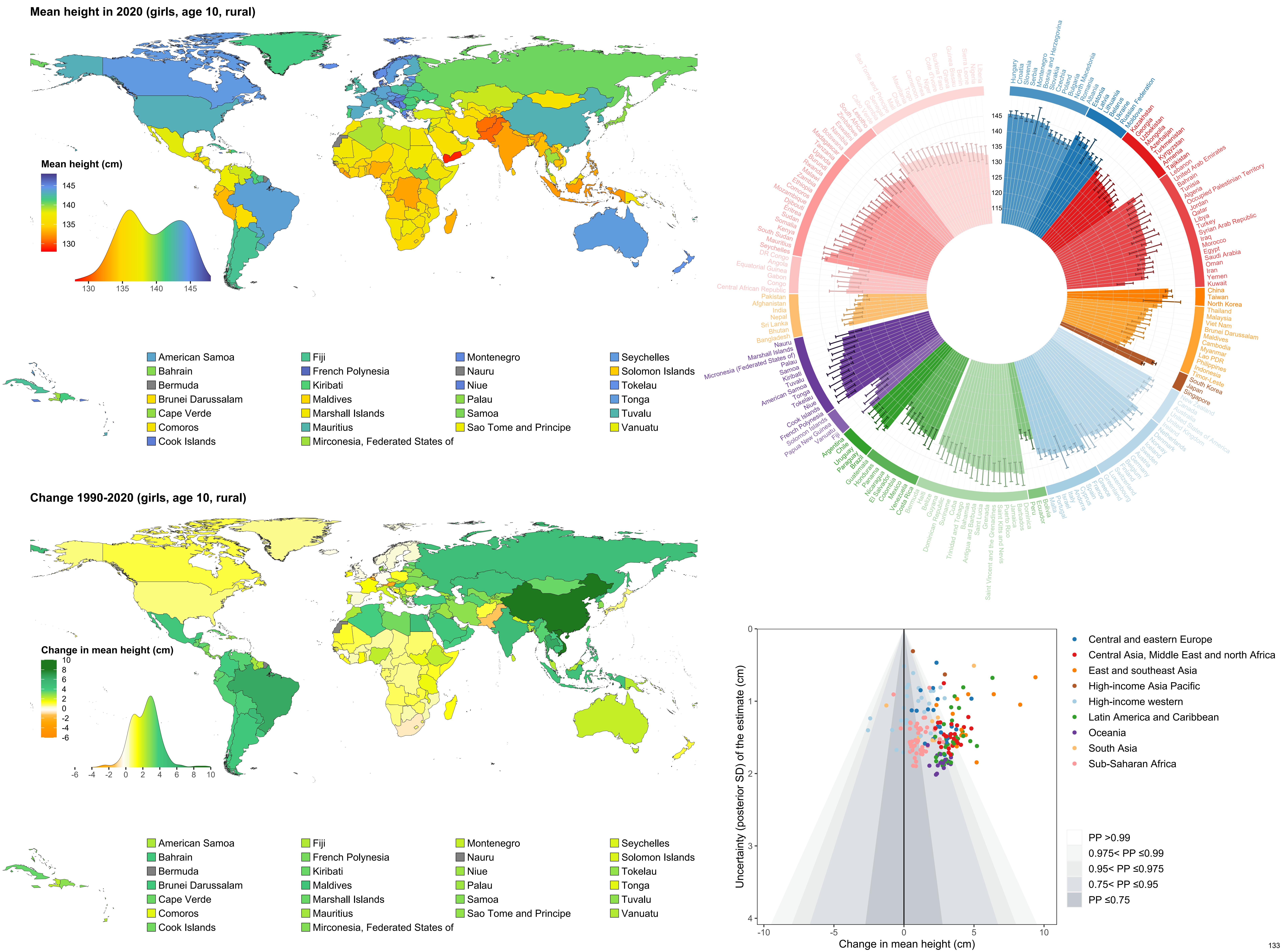
Mean height in 2020 (girls, age 10, rural)
Mean height (cm)
145
140
135
130
130 135 140 145
American Samoa
Bahrain
Bermuda
Brunei Darussalam
Cape Verde
Comoros
Cook Islands
Fiji
French Polynesia
Kiribati
Maldives
Marshall Islands
Mauritius
Mirconesia, Federated States of
Montenegro
Nauru
Niue
Palau
Samoa
Sao Tome and Principe
Seychelles
Solomon Islands
Tokelau
Tonga
Tuvalu
Vanuatu
Change 1990-2020 (girls, age 10, rural)
Change in mean height (cm)
10
8
6
4
2
0
-2
-4
-6
-6 -4 -2 0 2 4 6 8 10
American Samoa
Bahrain
Bermuda
Brunei Darussalam
Cape Verde
Comoros
Cook Islands
Fiji
French Polynesia
Kiribati
Maldives
Marshall Islands
Mauritius
Mirconesia, Federated States of
Montenegro
Nauru
Niue
Palau
Samoa
Sao Tome and Principe
Seychelles
Solomon Islands
Tokelau
Tonga
Tuvalu
Vanuatu
145
140
135
130
125
120
115
Uncertainty (posterior SD) of the estimate (cm)
Change in mean height (cm)
0
1
2
3
4
-10 -5 0 5 10
Central and eastern Europe
Central Asia, Middle East and north Africa
East and southeast Asia
High-income Asia Pacific
High-income western
Latin America and Caribbean
Oceania
South Asia
Sub-Saharan Africa
PP >0.99
0.975< PP ≤0.99
0.95< PP ≤0.975
0.75< PP ≤0.95
PP ≤0.75

Mean height in 2020 (boys, age 10, urban)

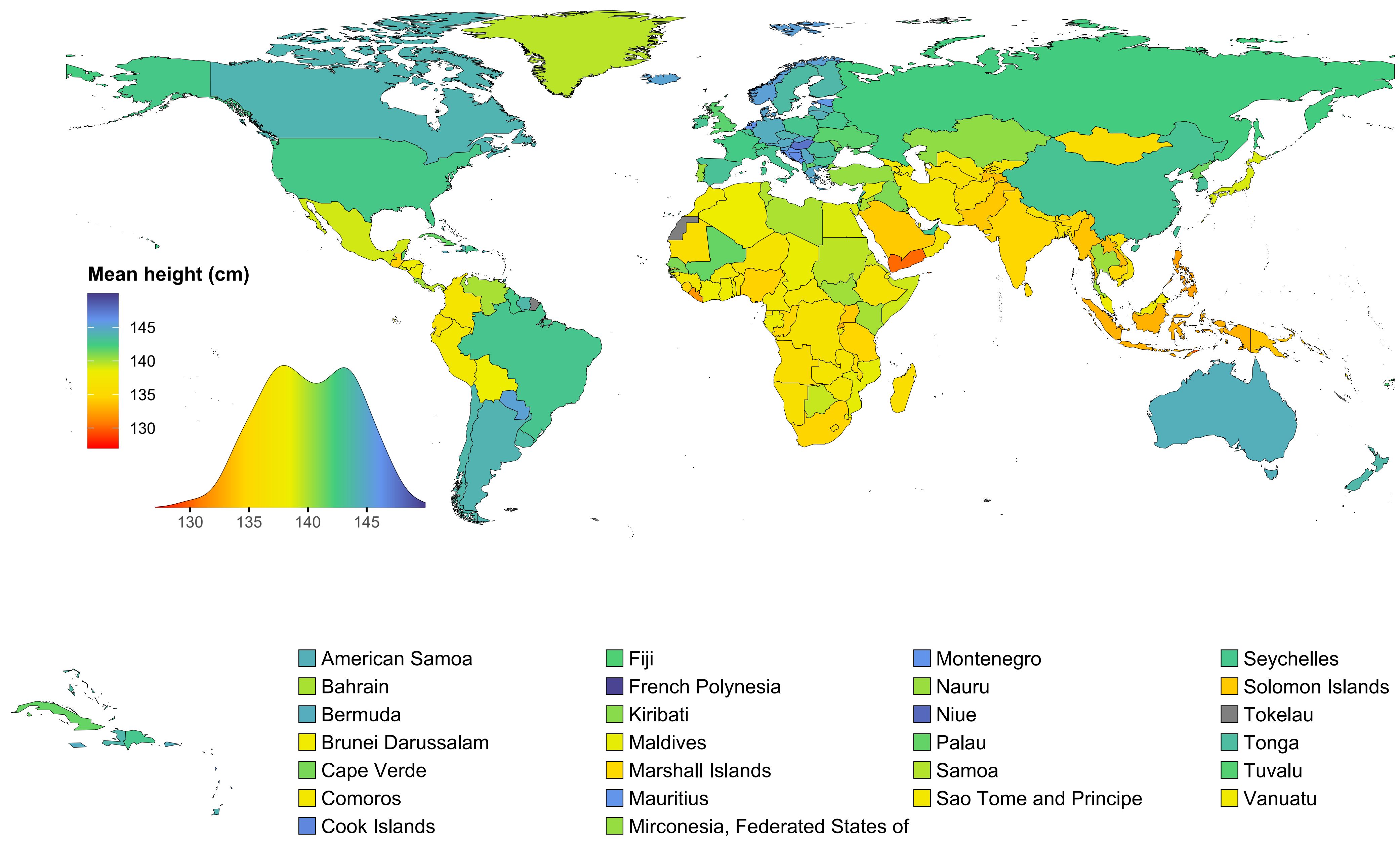

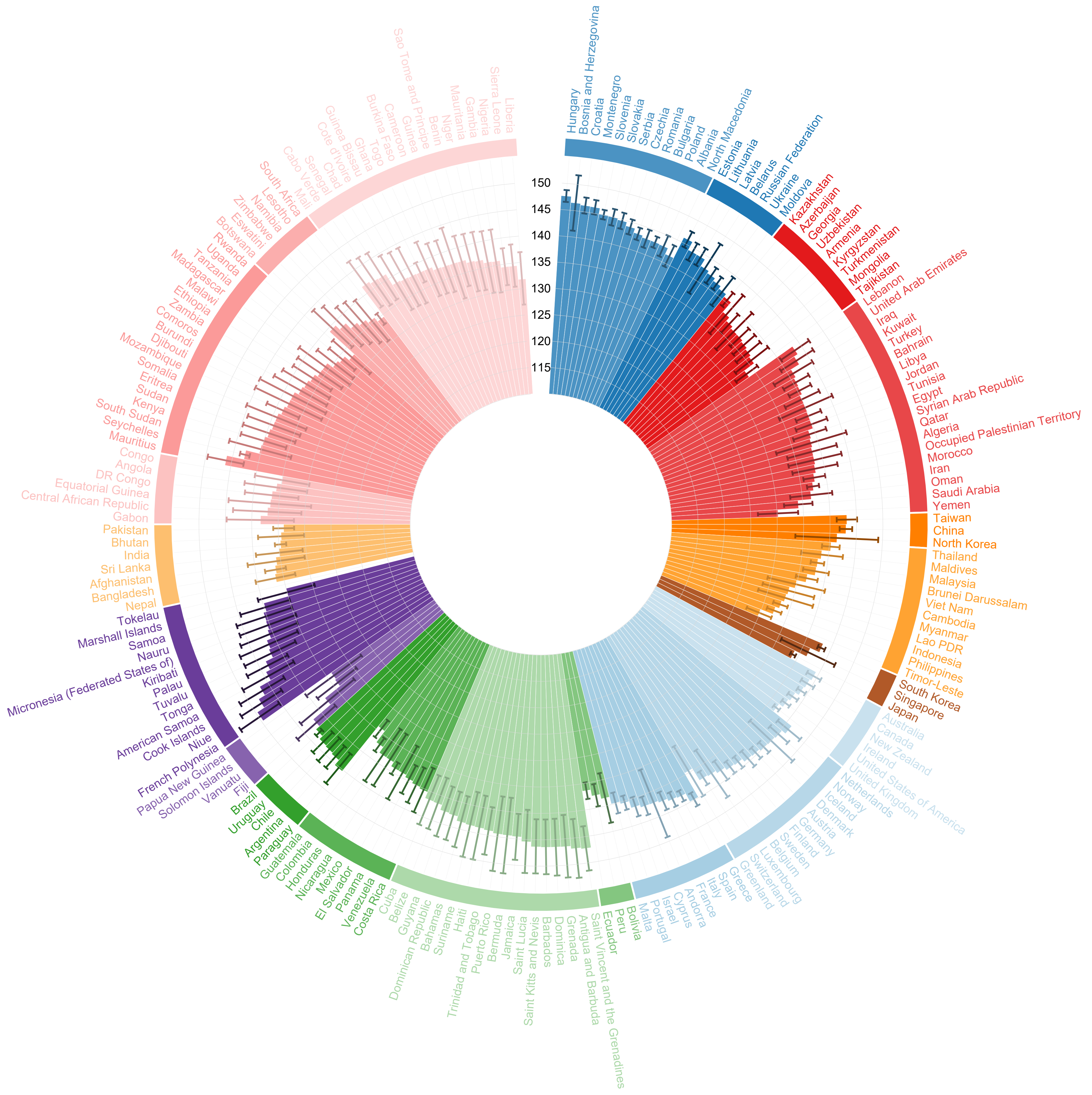

Change 1990-2020 (boys, age 10, urban)

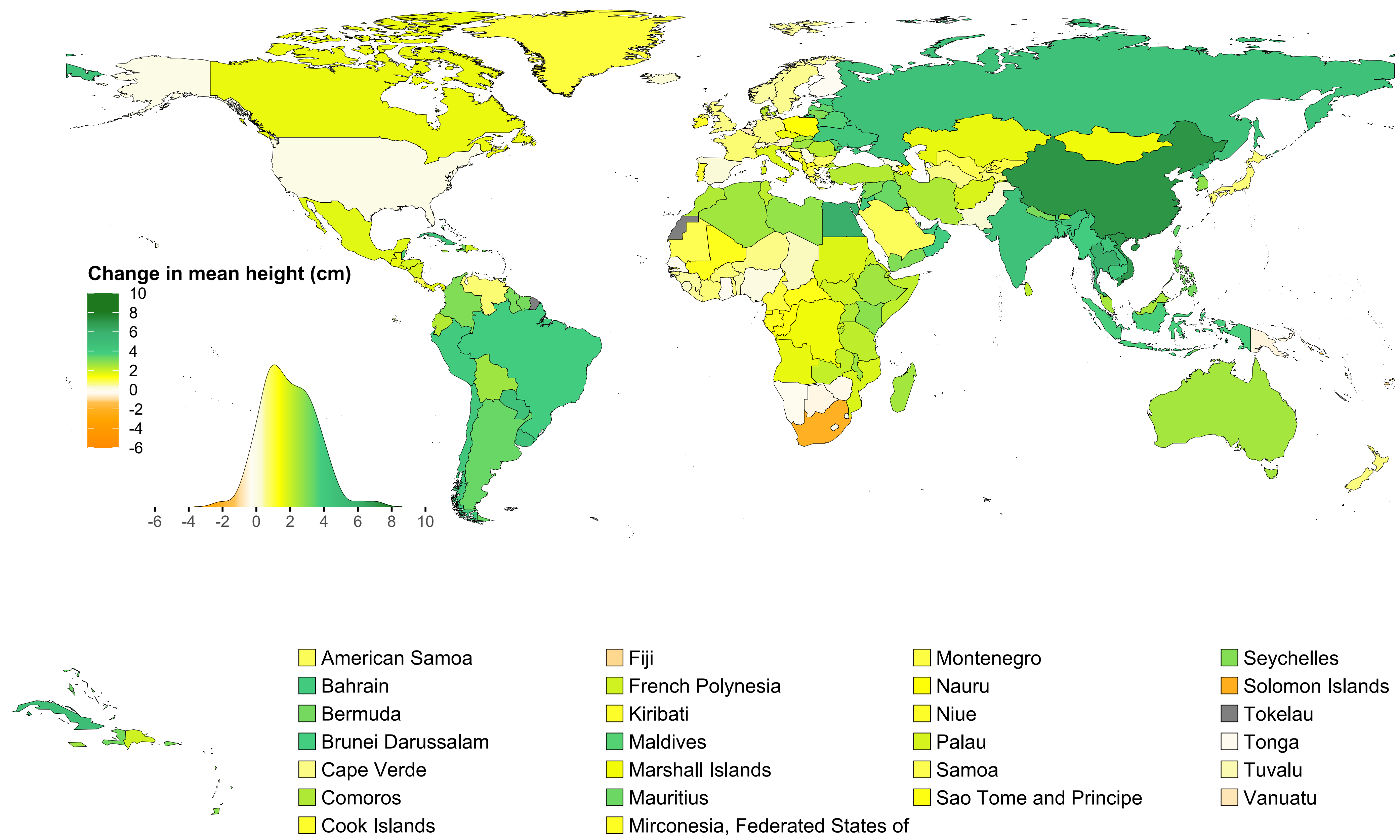

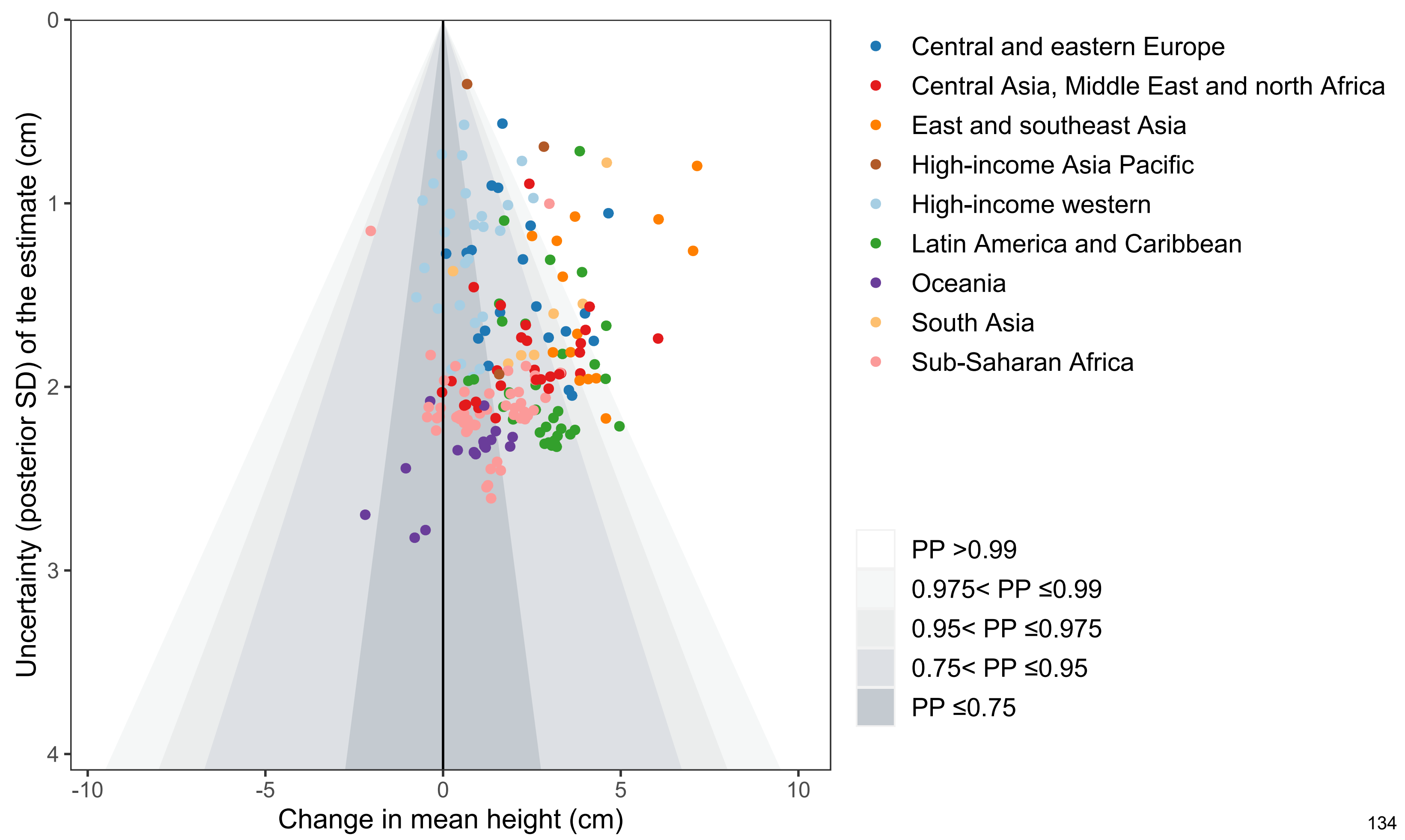

Mean height in 2020 (boys, age 10, rural)

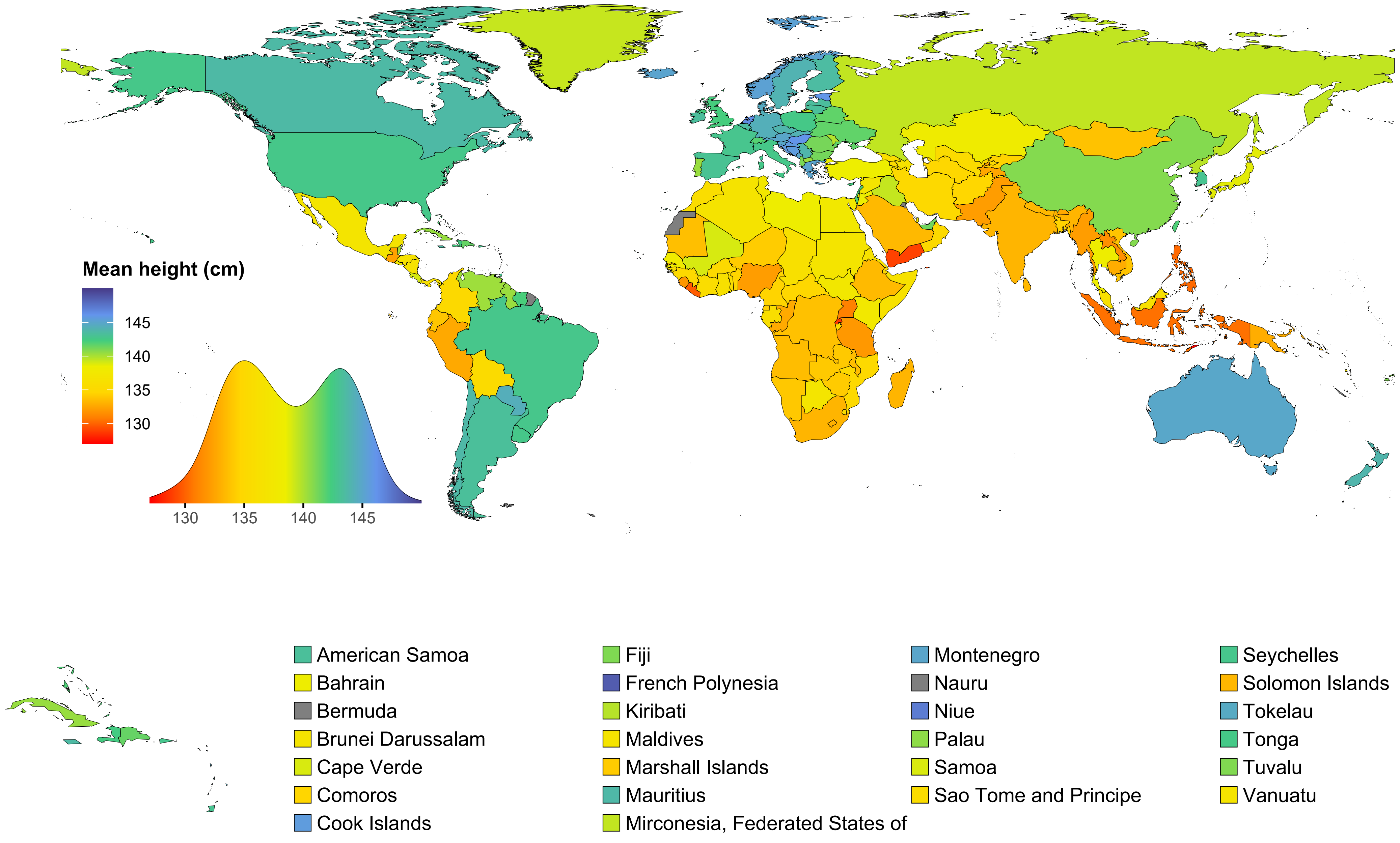

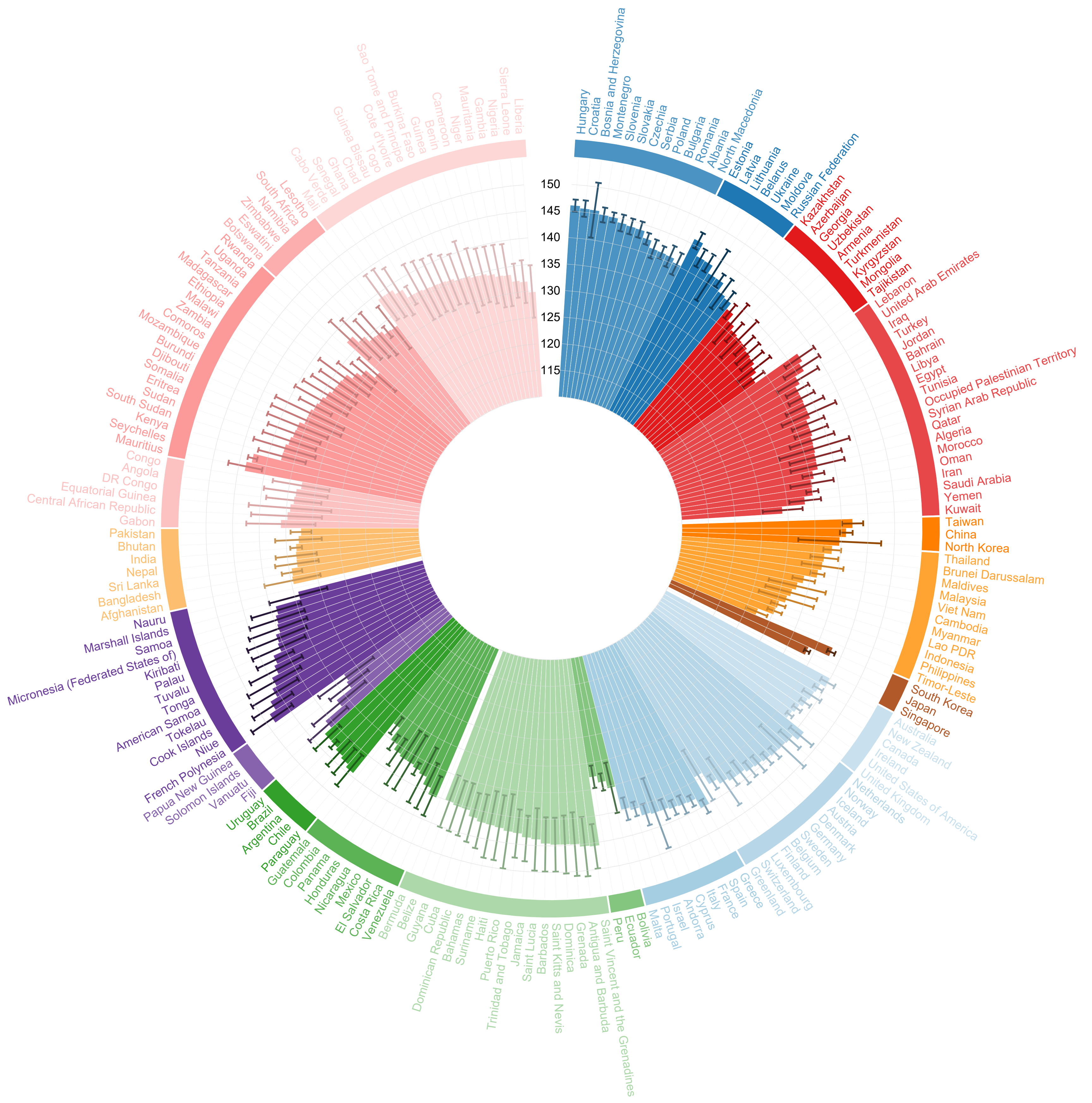

Change 1990-2020 (boys, age 10, rural)

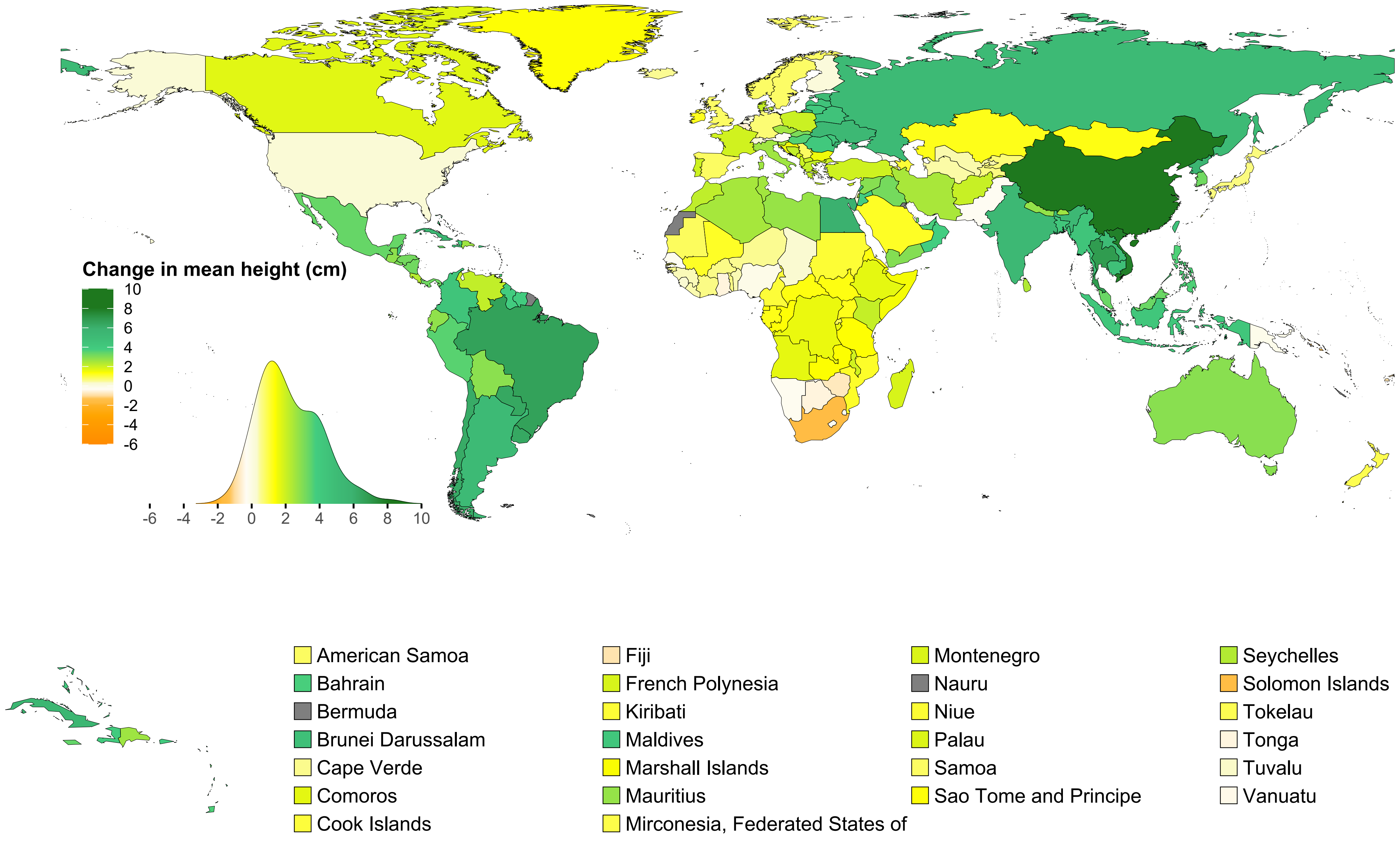

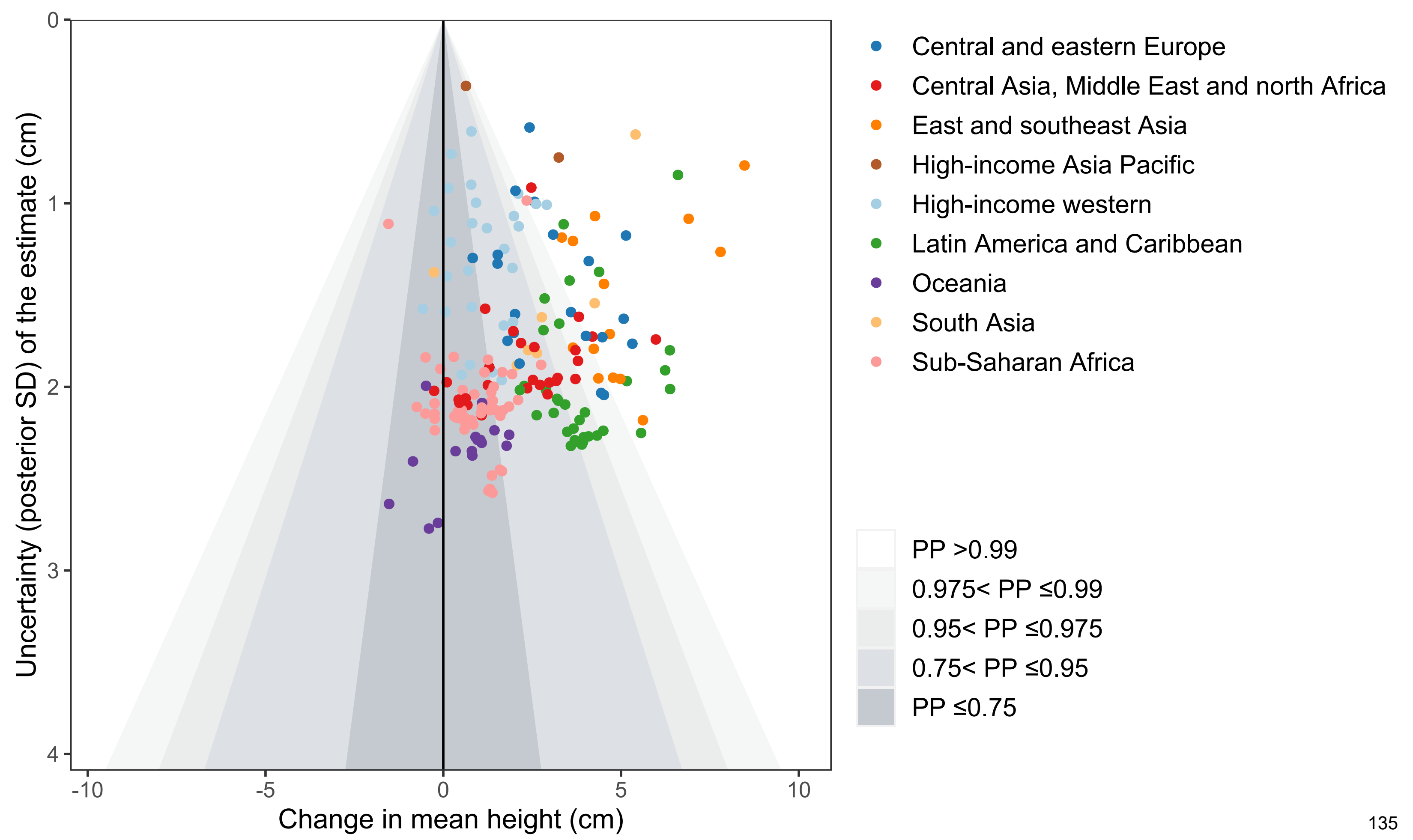

Mean height in 2020 (girls, age 15, urban)

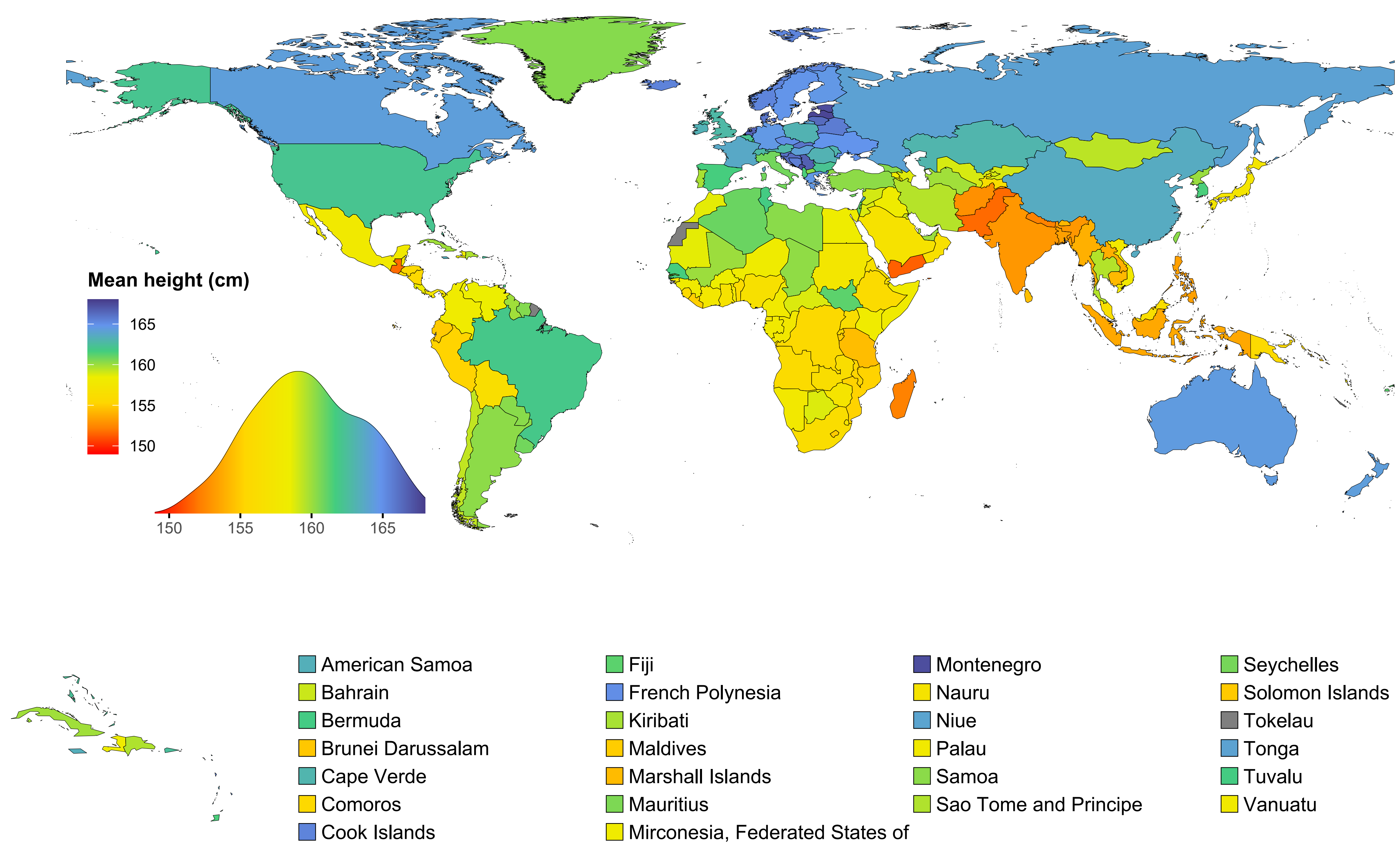

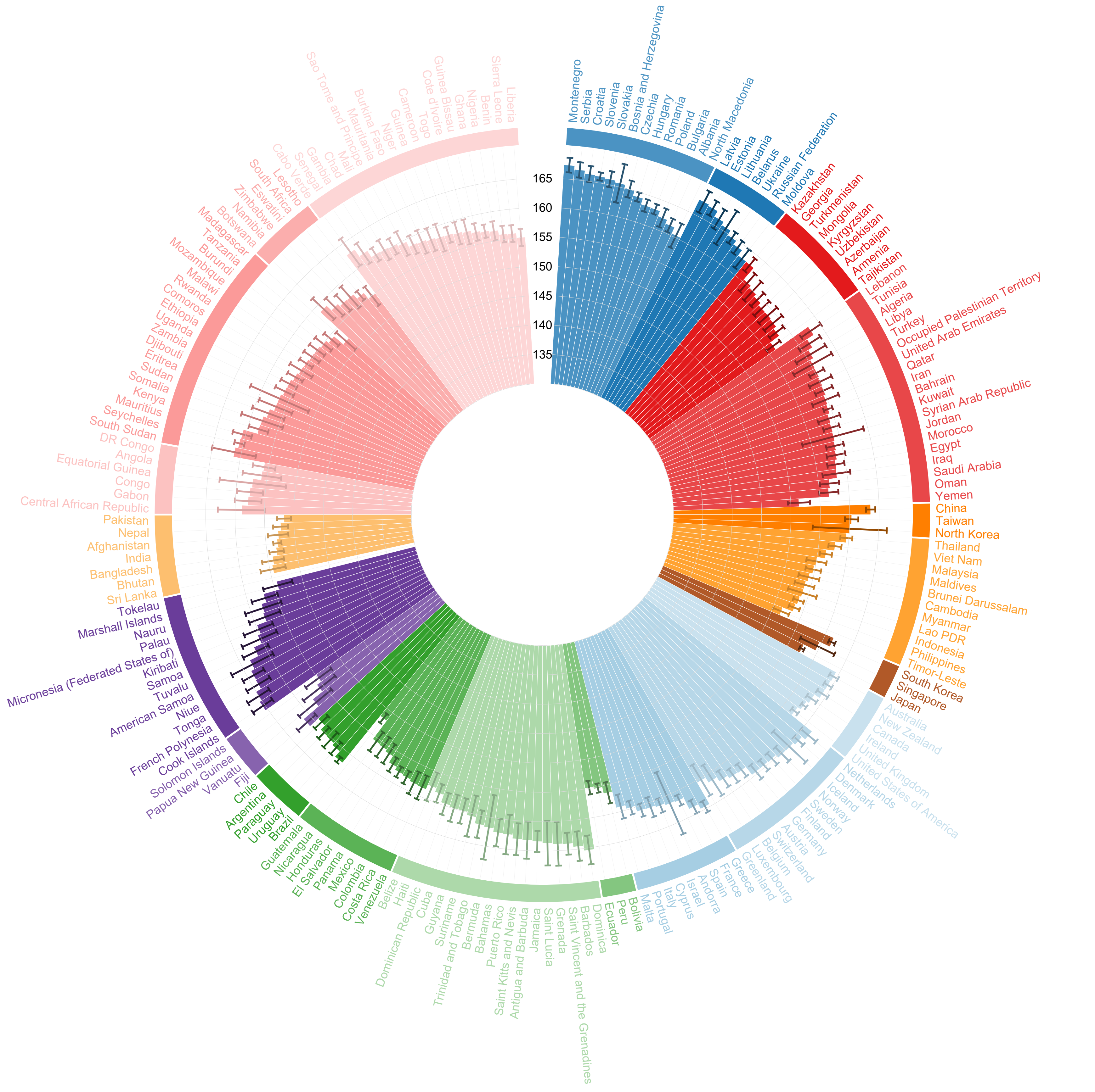

Change 1990-2020 (girls, age 15, urban)

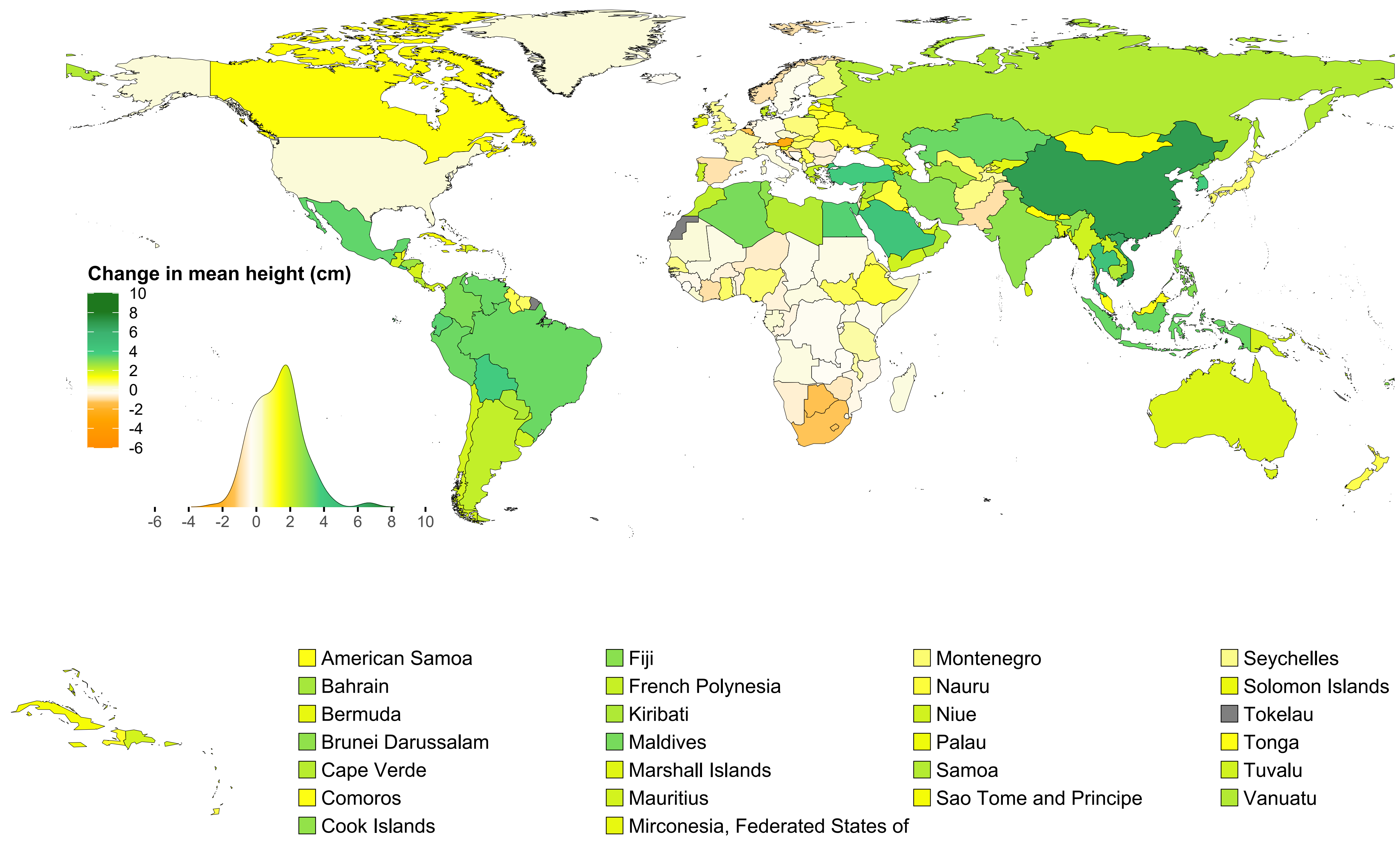

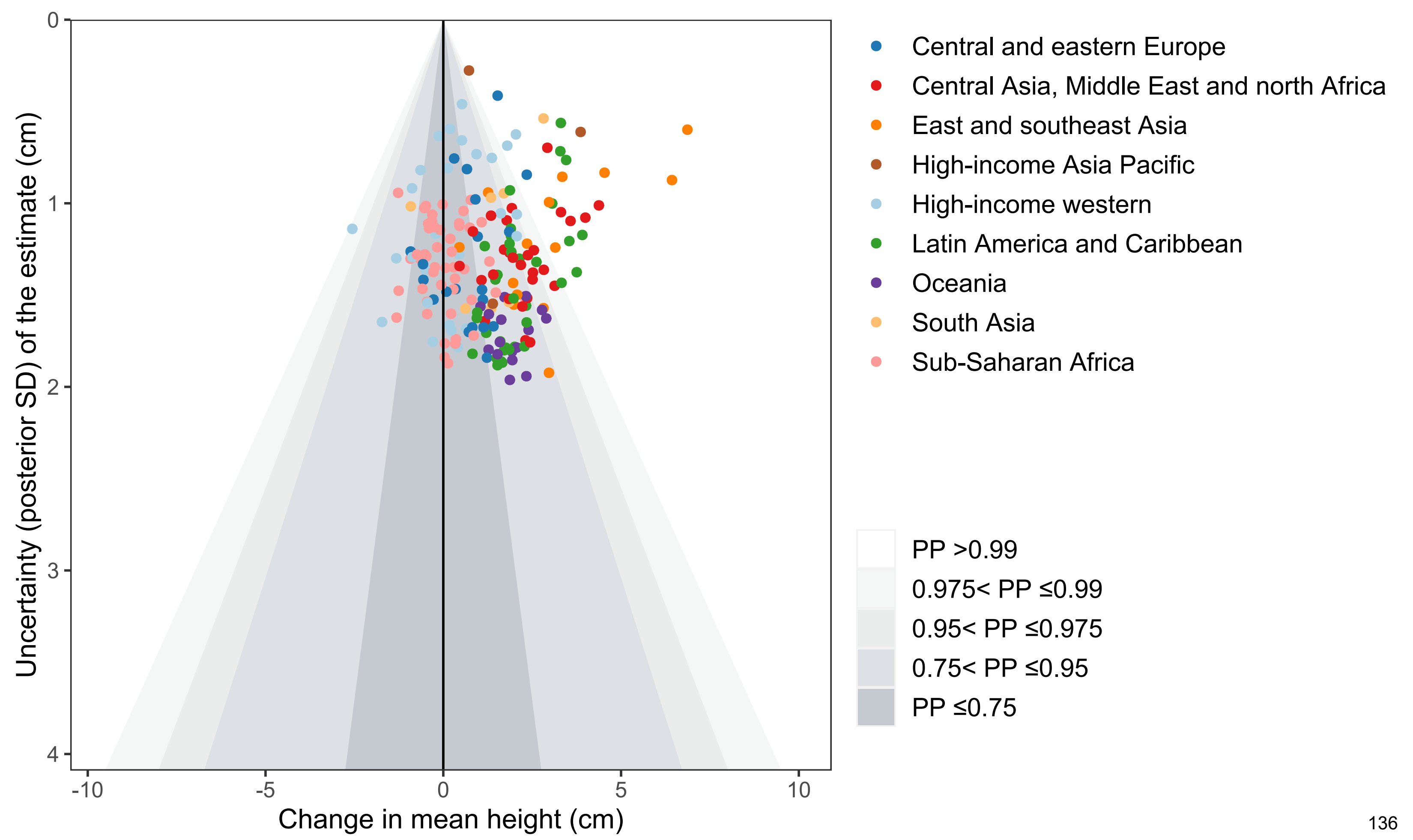

Mean height in 2020 (girls, age 15, rural)

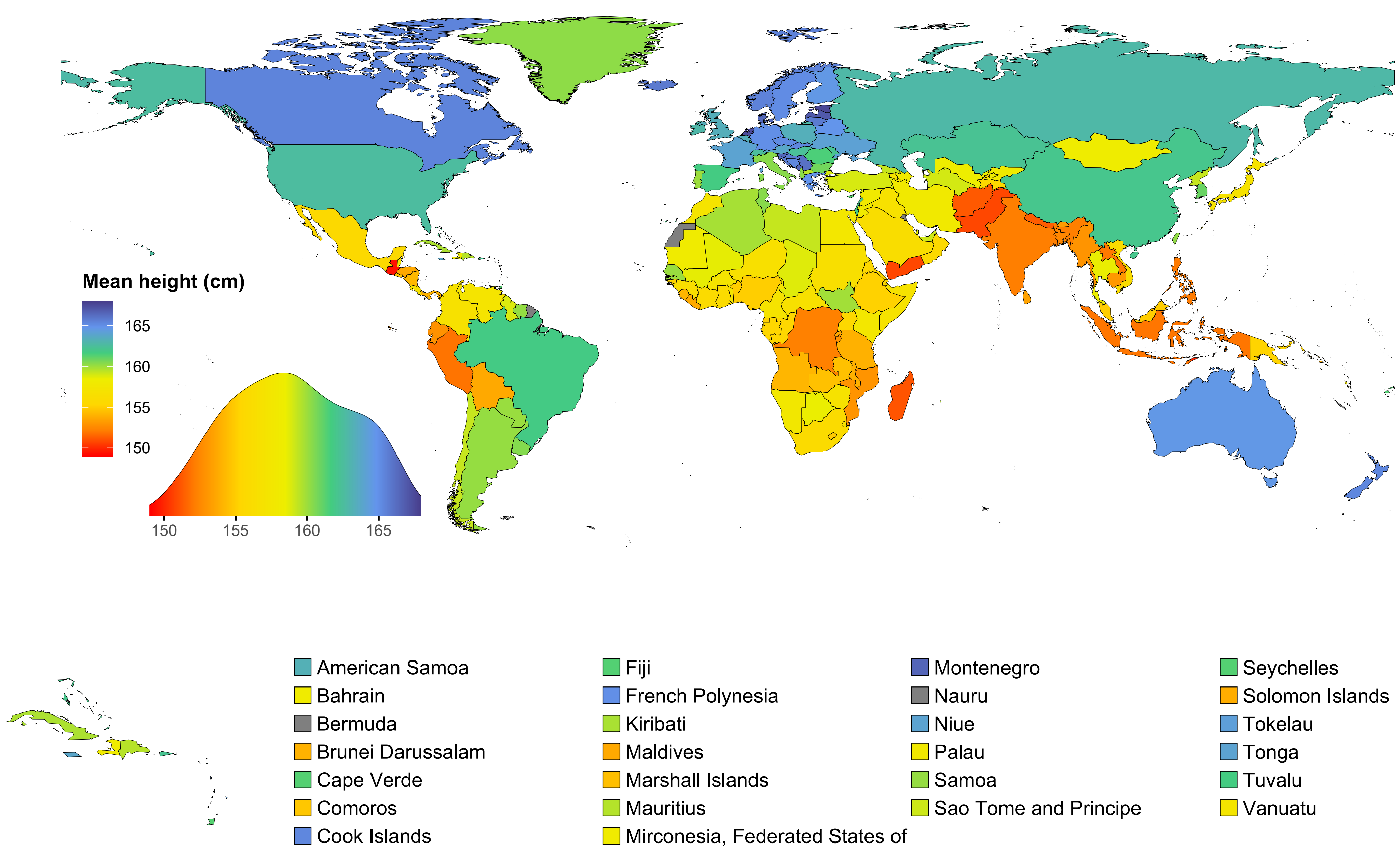

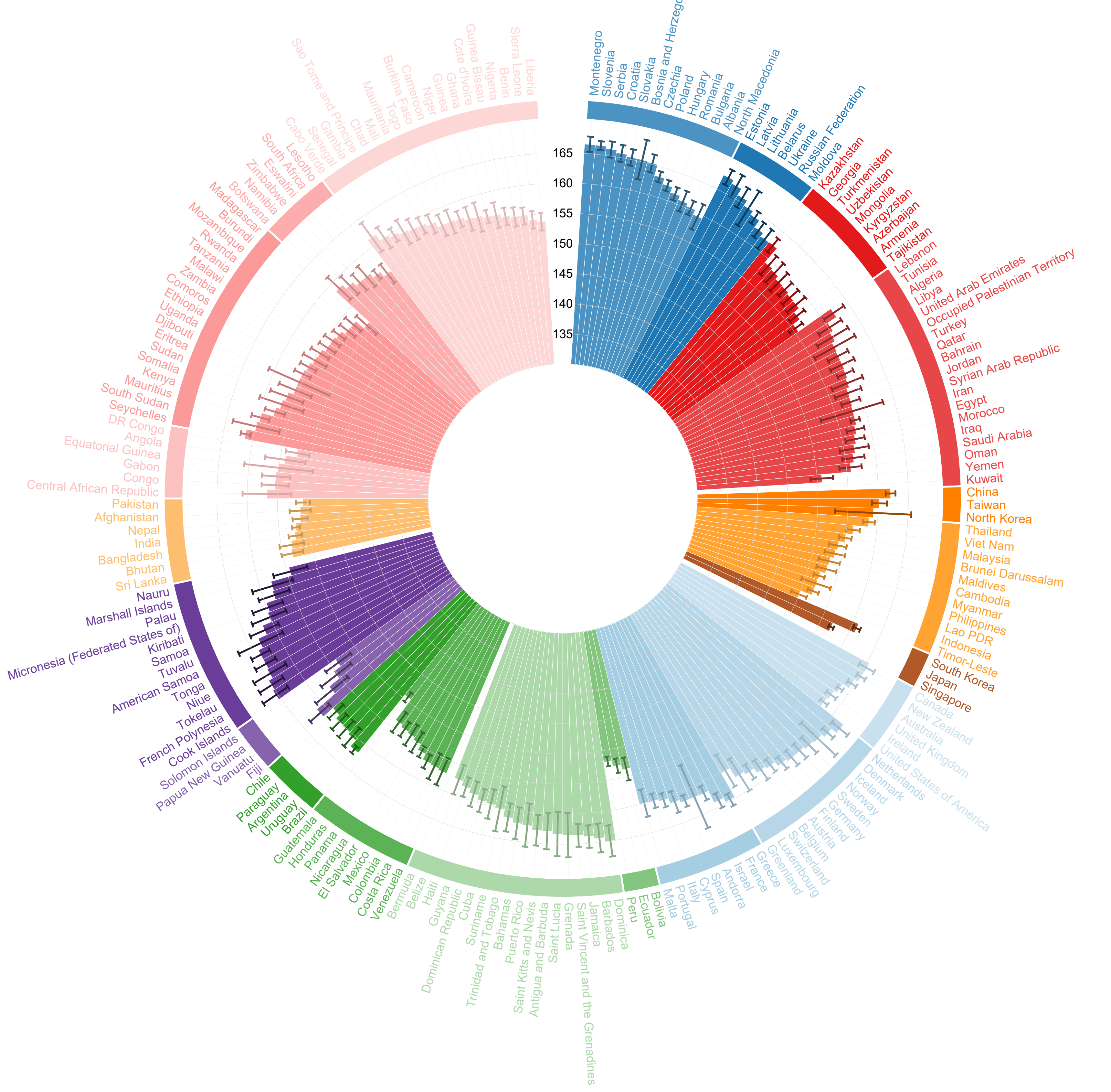

Change 1990-2020 (girls, age 15, rural)

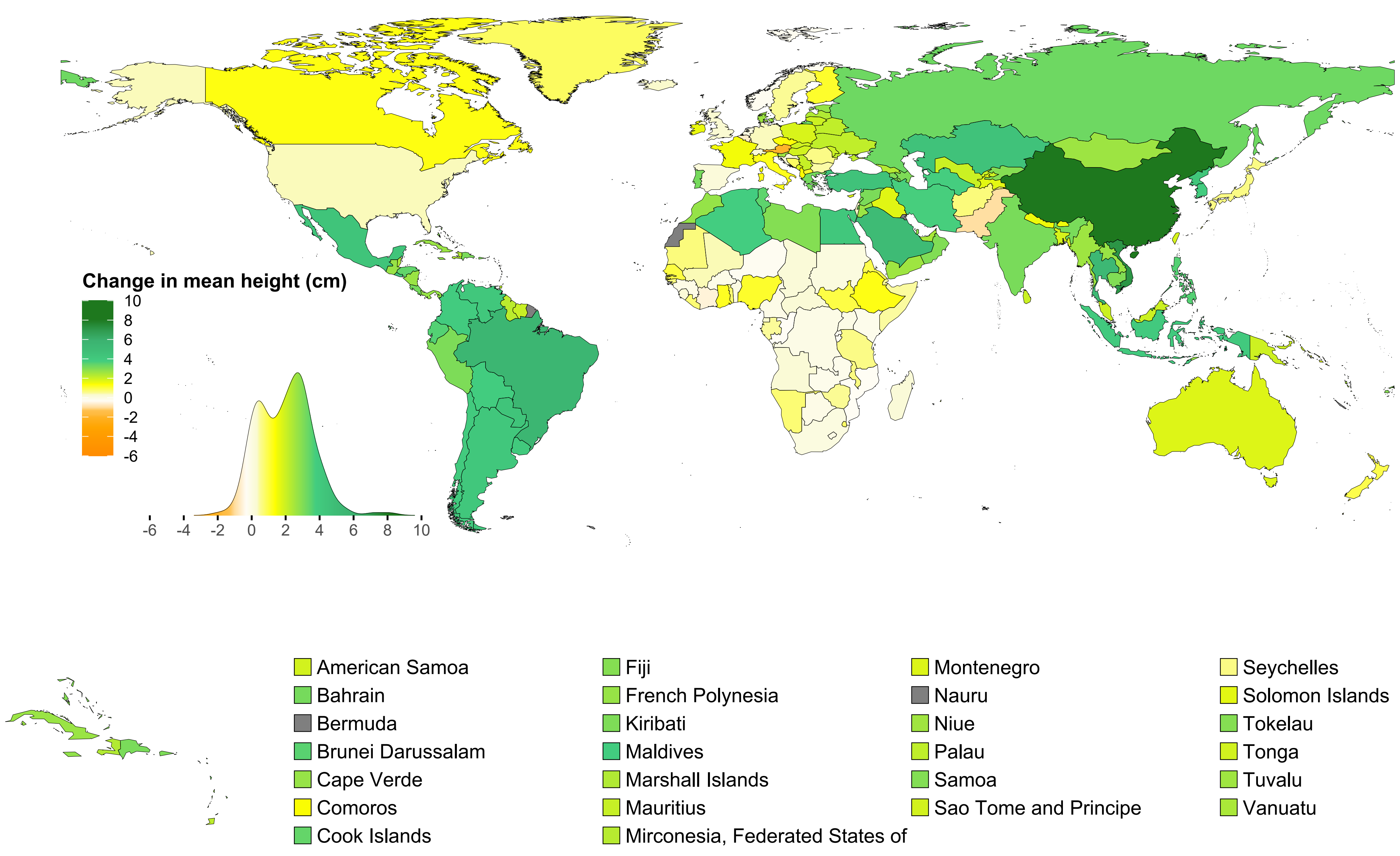

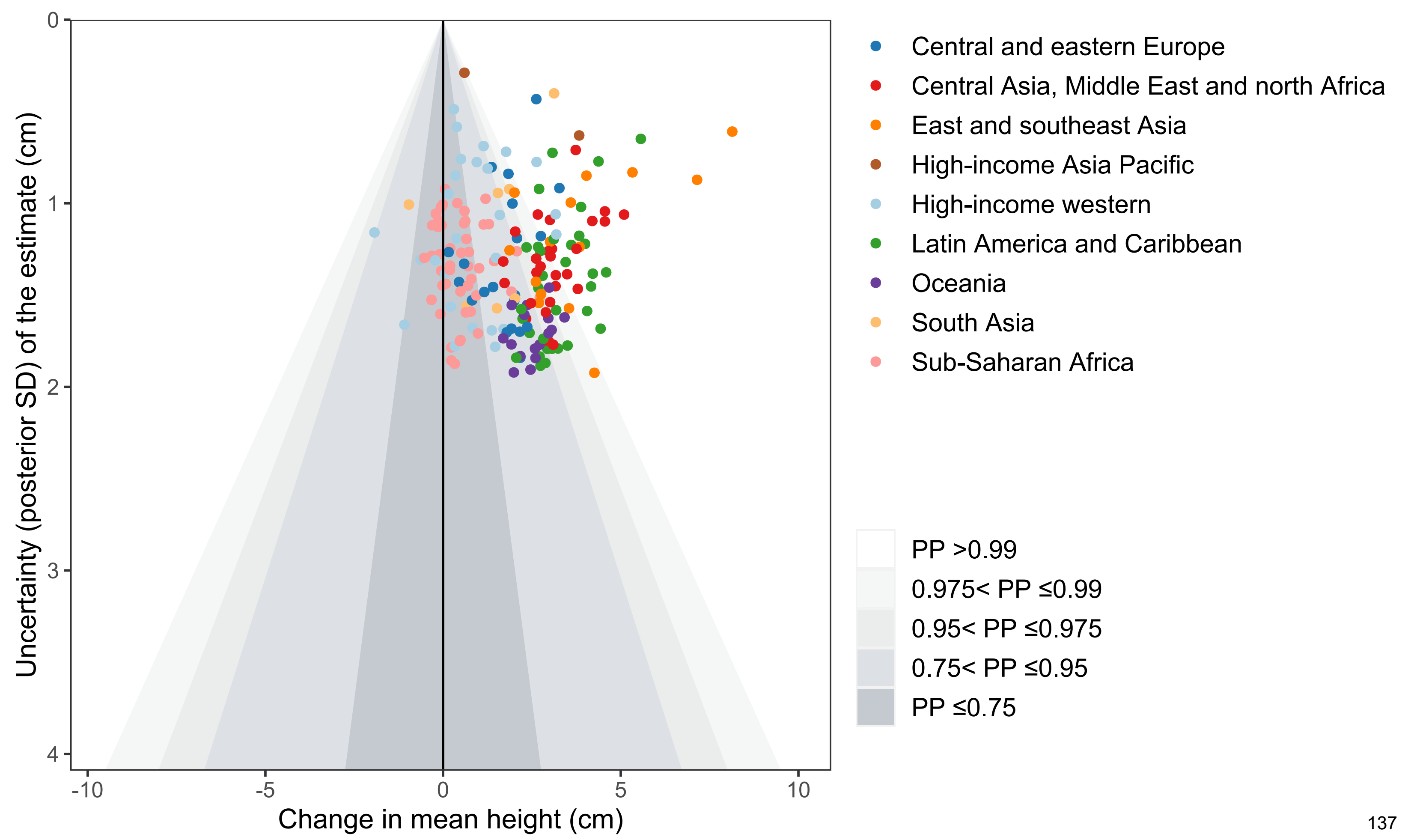

Mean height in 2020 (boys, age 15, urban)

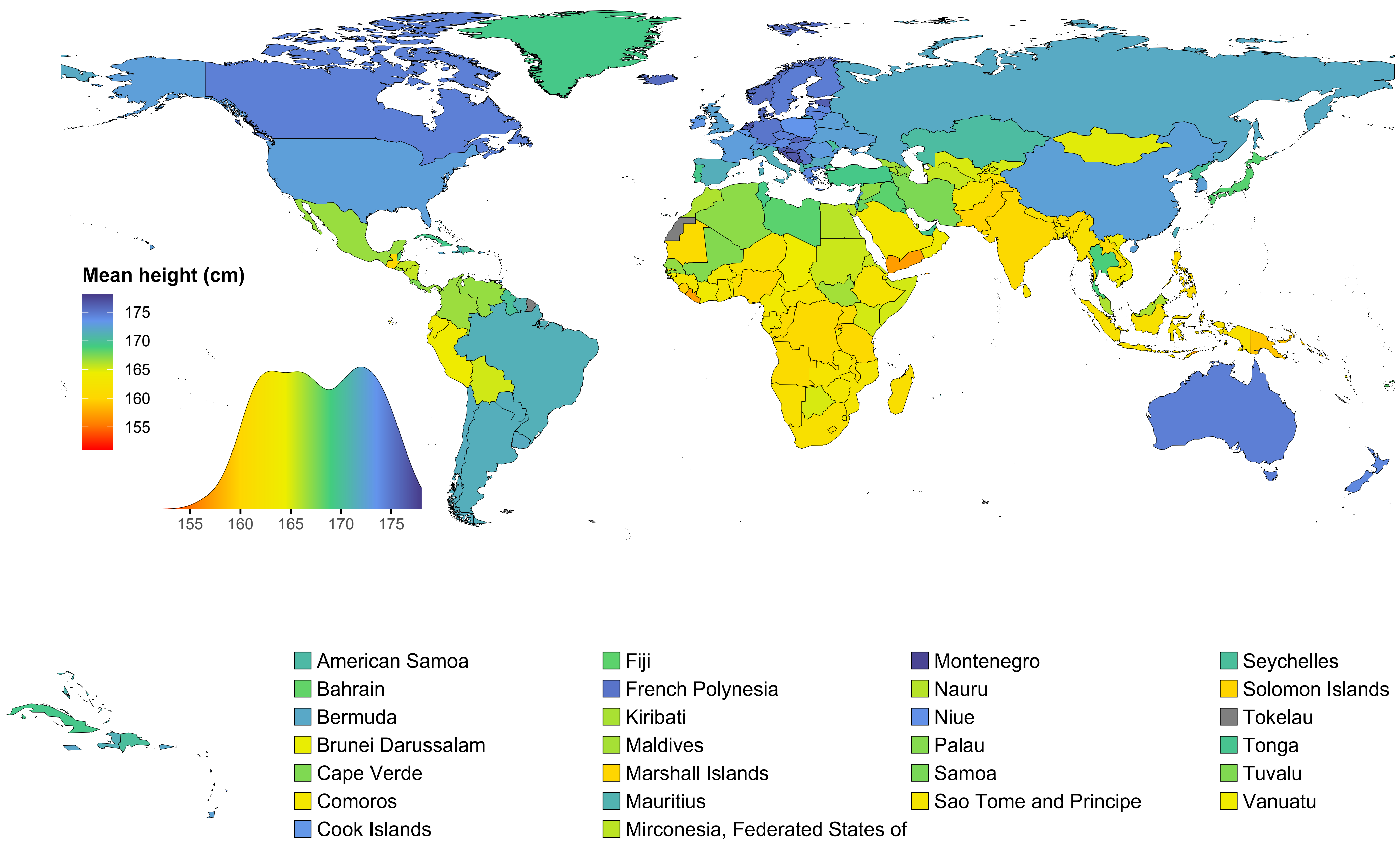

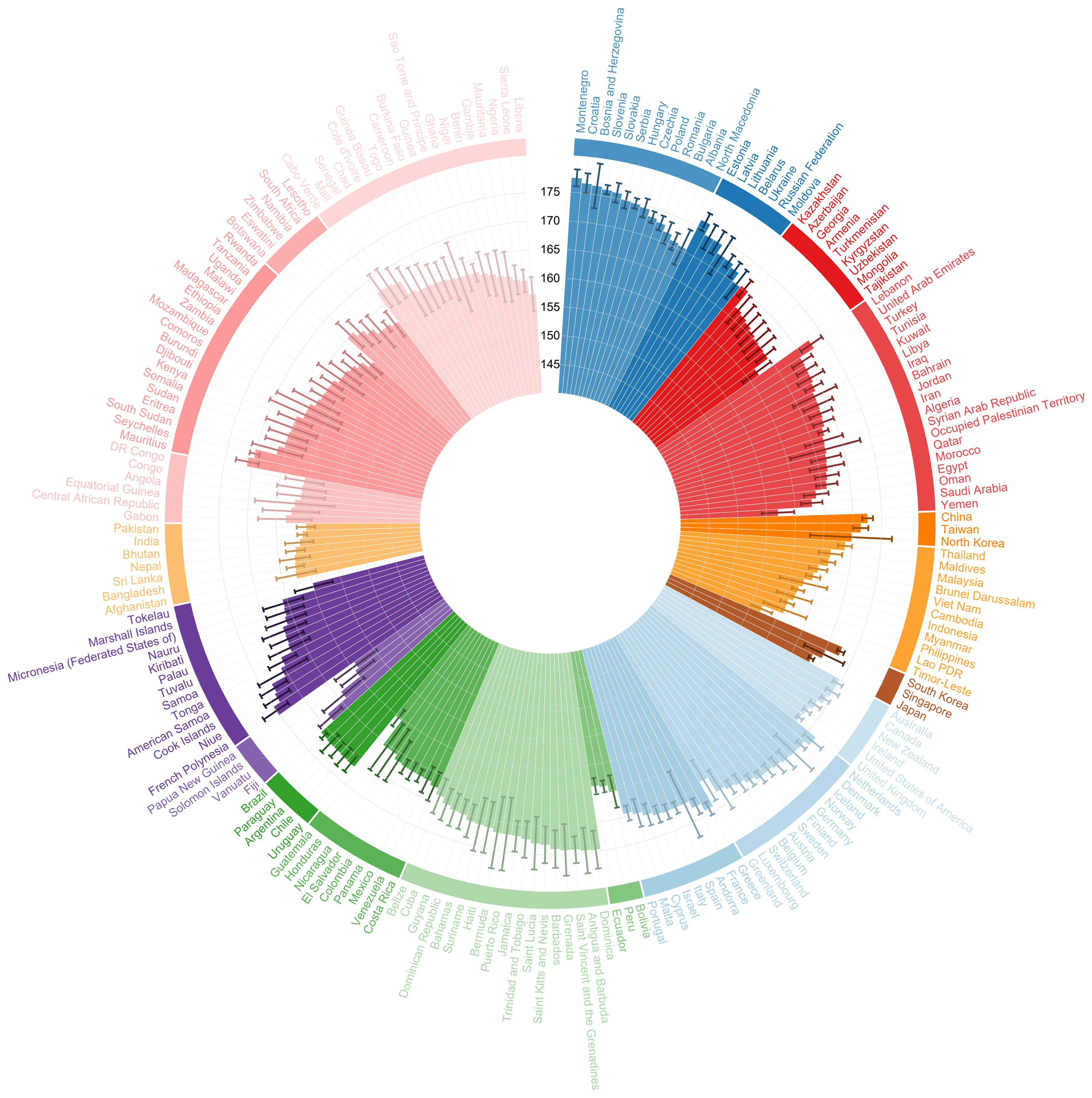

Change 1990-2020 (boys, age 15, urban)

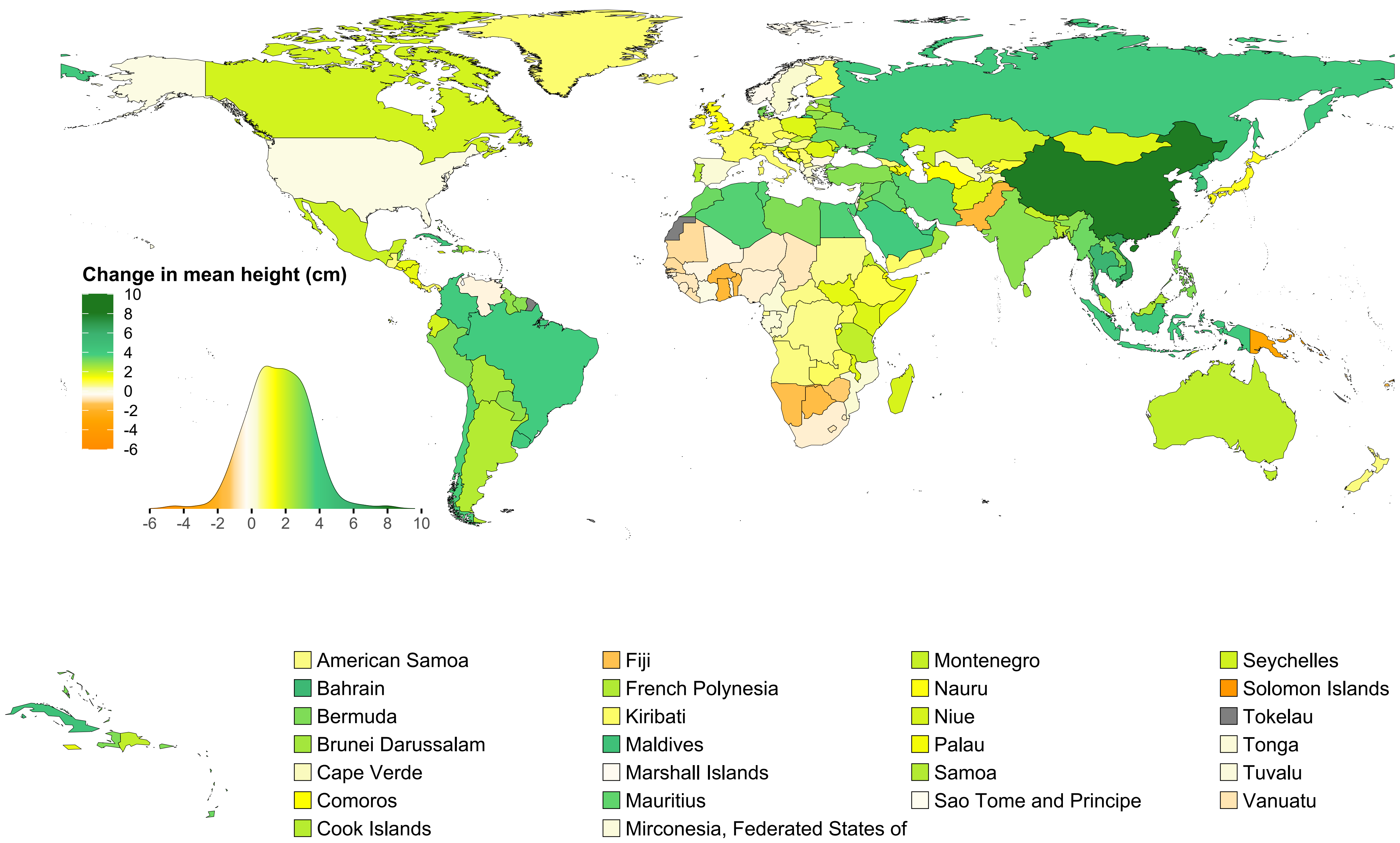

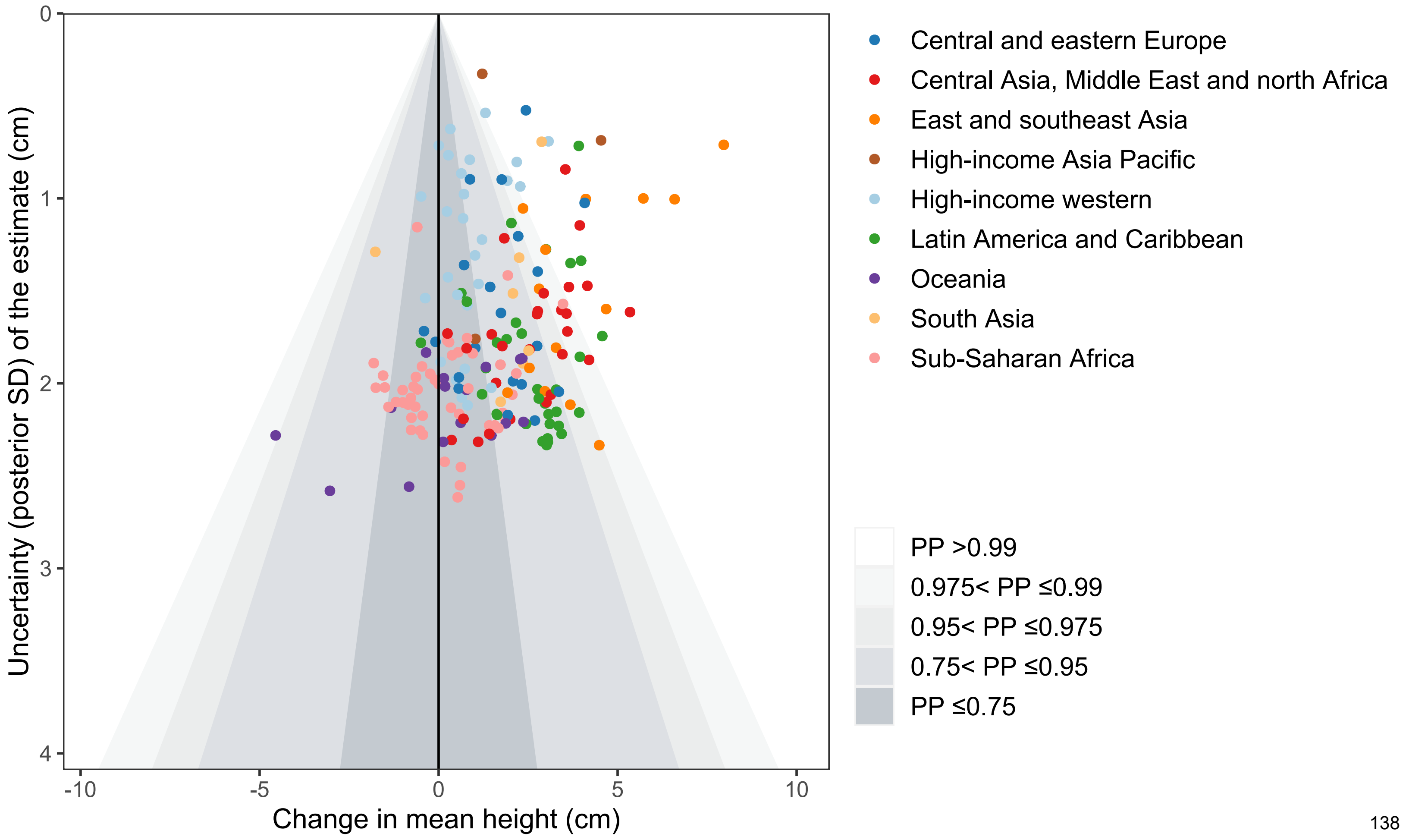

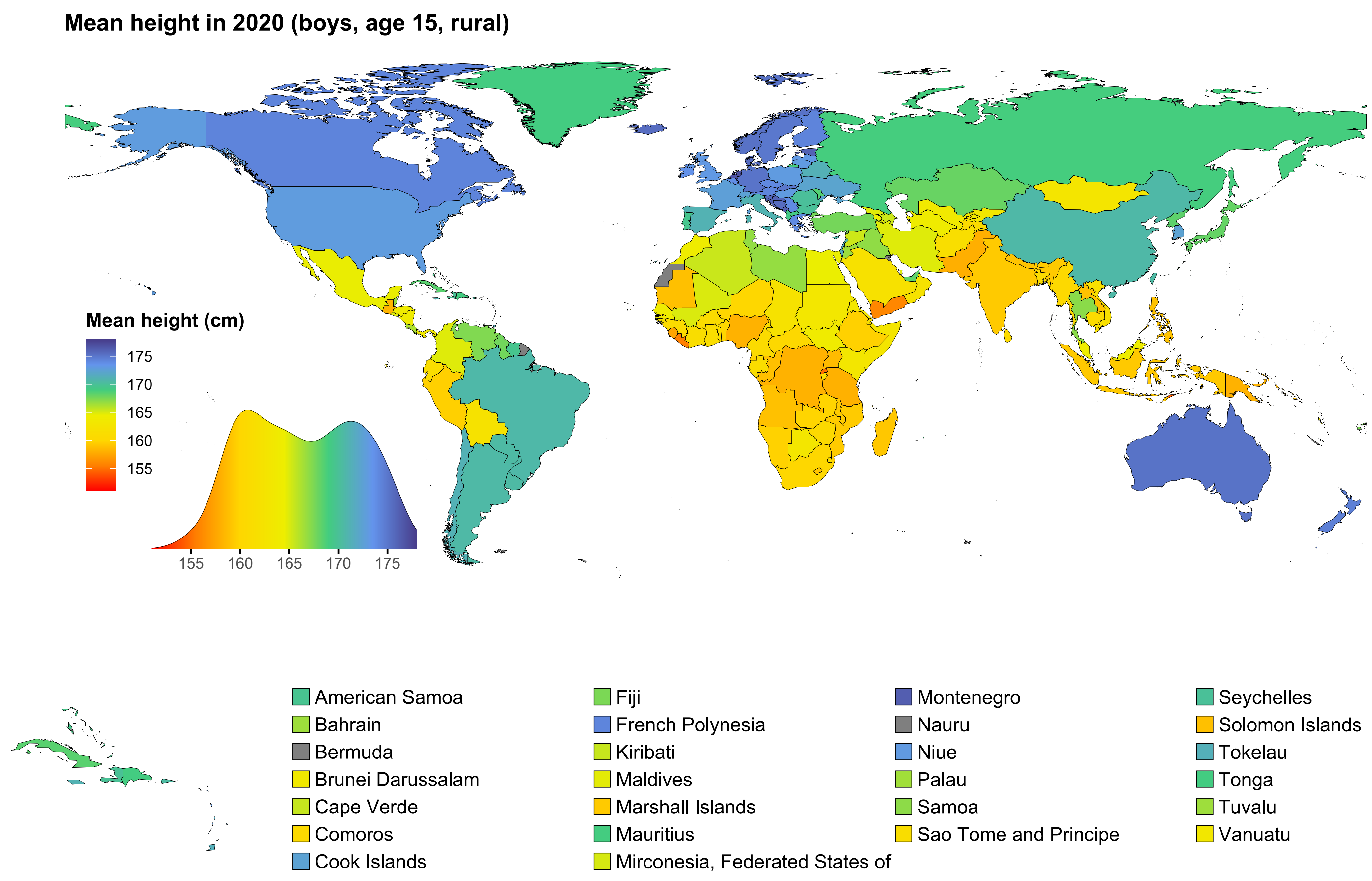
Mean height in 2020 (boys, age 15, rural)
Mean height (cm)
175
170
165
160
155
155 160 165 170 175
American Samoa
Bahrain
Bermuda
Brunei Darussalam
Cape Verde
Comoros
Cook Islands
Fiji
French Polynesia
Kiribati
Maldives
Marshall Islands
Mauritius
Mirconesia, Federated States of
Montenegro
Nauru
Niue
Palau
Samoa
Sao Tome and Principe
Seychelles
Solomon Islands
Tokelau
Tonga
Tuvalu
Vanuatu

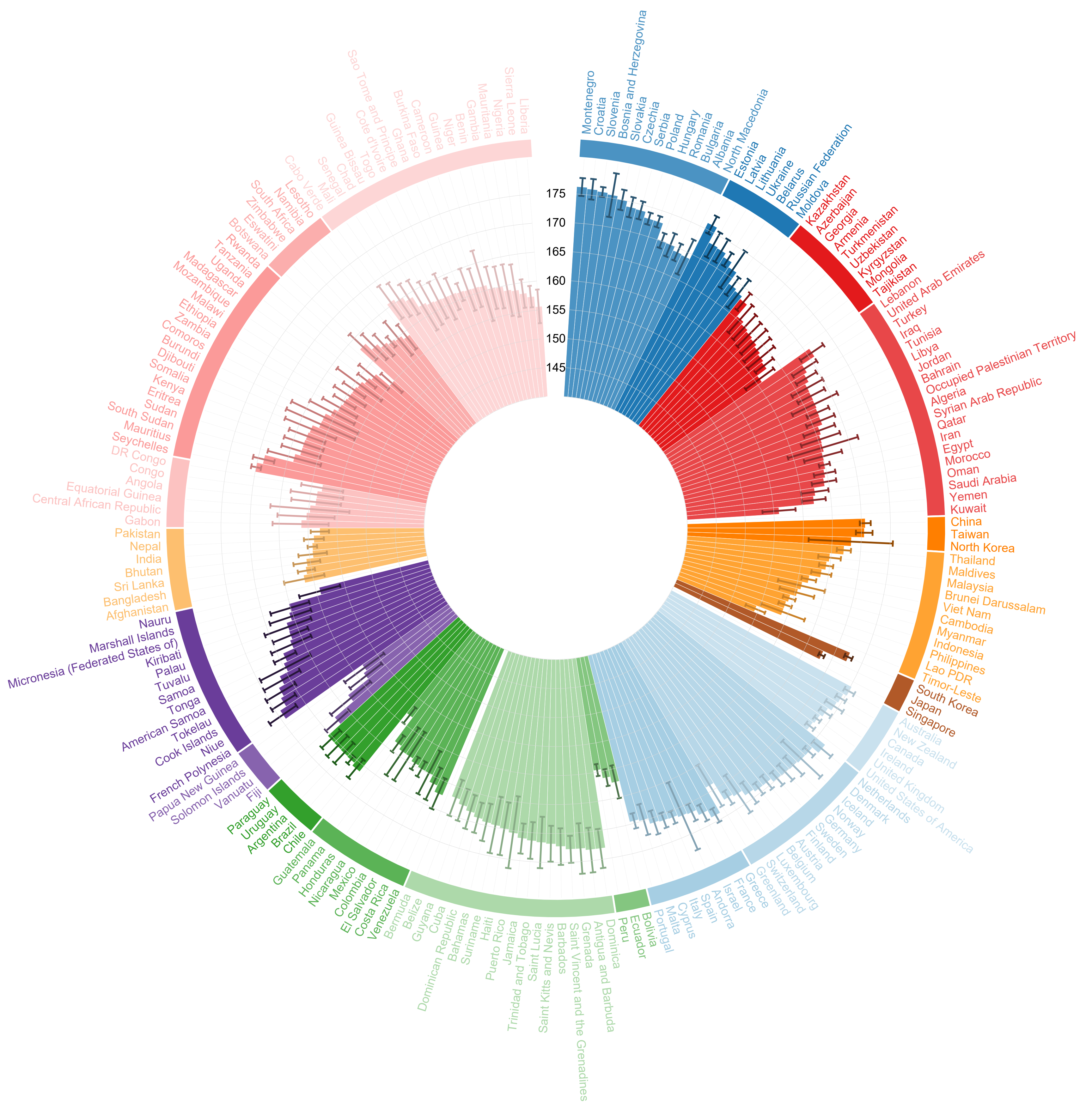
175
170
165
160
155
150
145

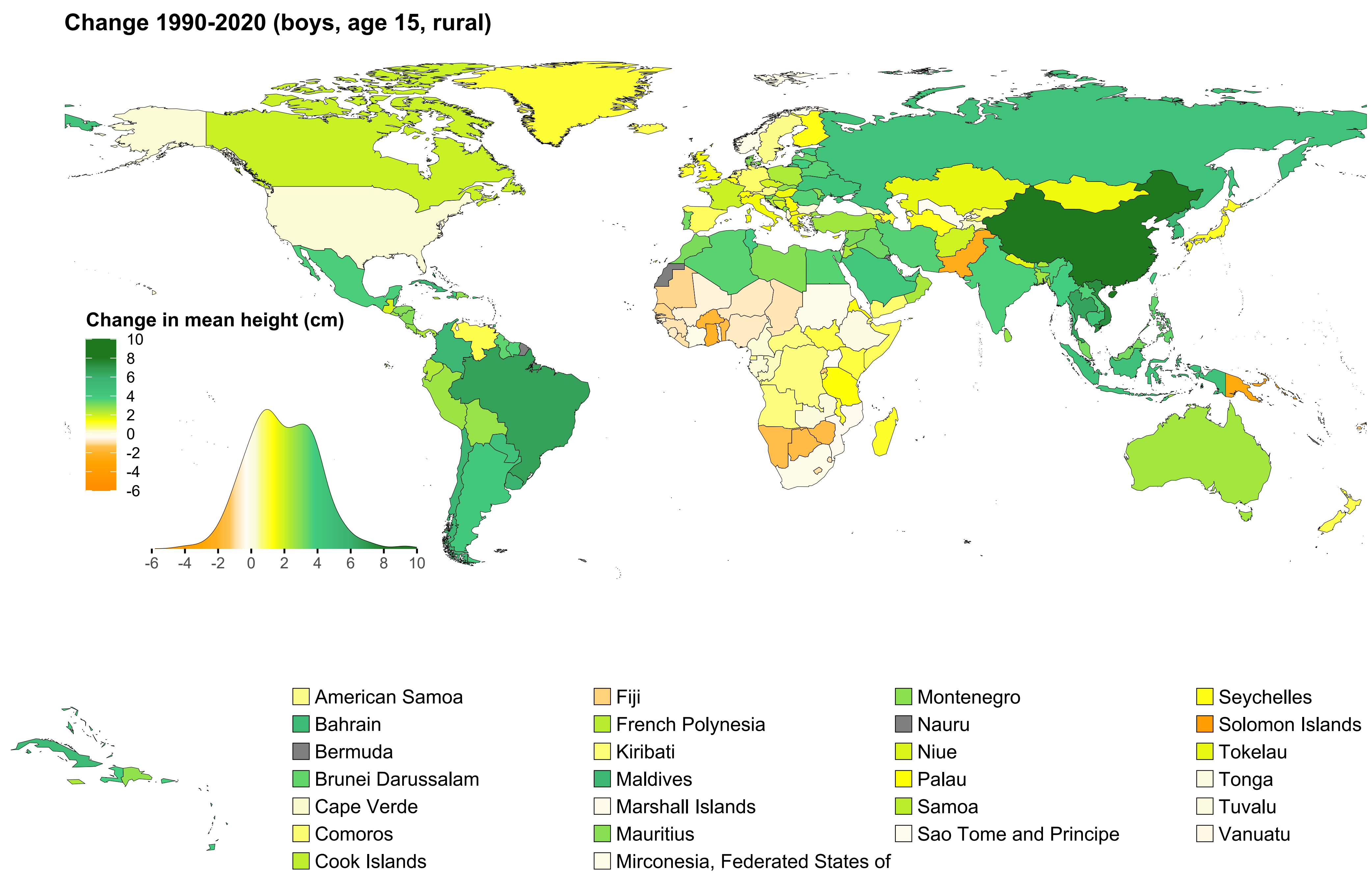
Change 1990-2020 (boys, age 15, rural)
Change in mean height (cm)
10
8
6
4
2
0
-2
-4
-6
-6 -4 -2 0 2 4 6 8 10
American Samoa
Bahrain
Bermuda
Brunei Darussalam
Cape Verde
Comoros
Cook Islands
Fiji
French Polynesia
Kiribati
Maldives
Marshall Islands
Mauritius
Mirconesia, Federated States of
Montenegro
Nauru
Niue
Palau
Samoa
Sao Tome and Principe
Seychelles
Solomon Islands
Tokelau
Tonga
Tuvalu
Vanuatu

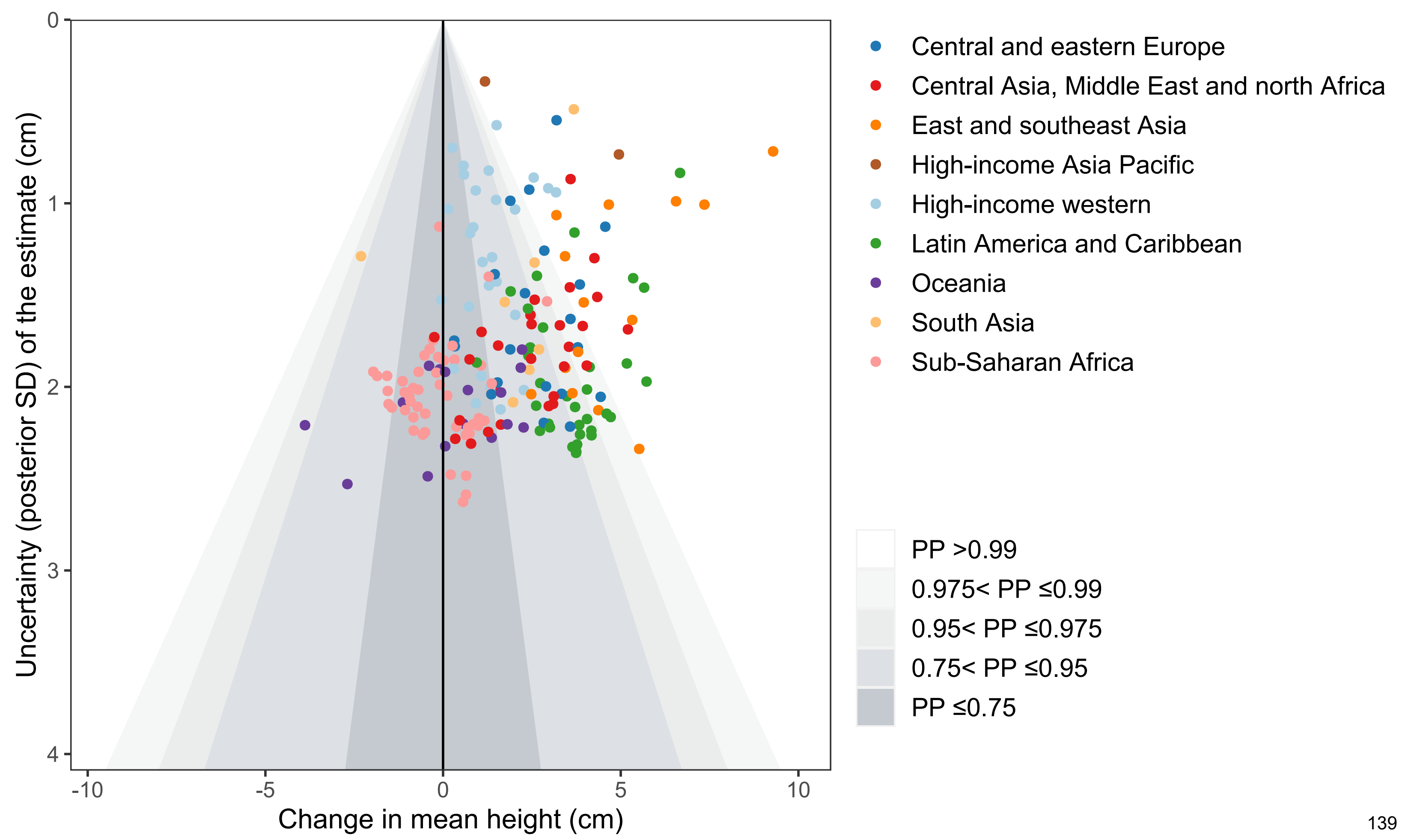
Uncertainty (posterior SD) of the estimate (cm)
0
1
2
3
4
-10 -5 0 5 10
Change in mean height (cm)
Central and eastern Europe
Central Asia, Middle East and north Africa
East and southeast Asia
High-income Asia Pacific
High-income western
Latin America and Caribbean
Oceania
South Asia
Sub-Saharan Africa
PP >0.99
0.975< PP ≤0.99
0.95< PP ≤0.975
0.75< PP ≤0.95
PP ≤0.75

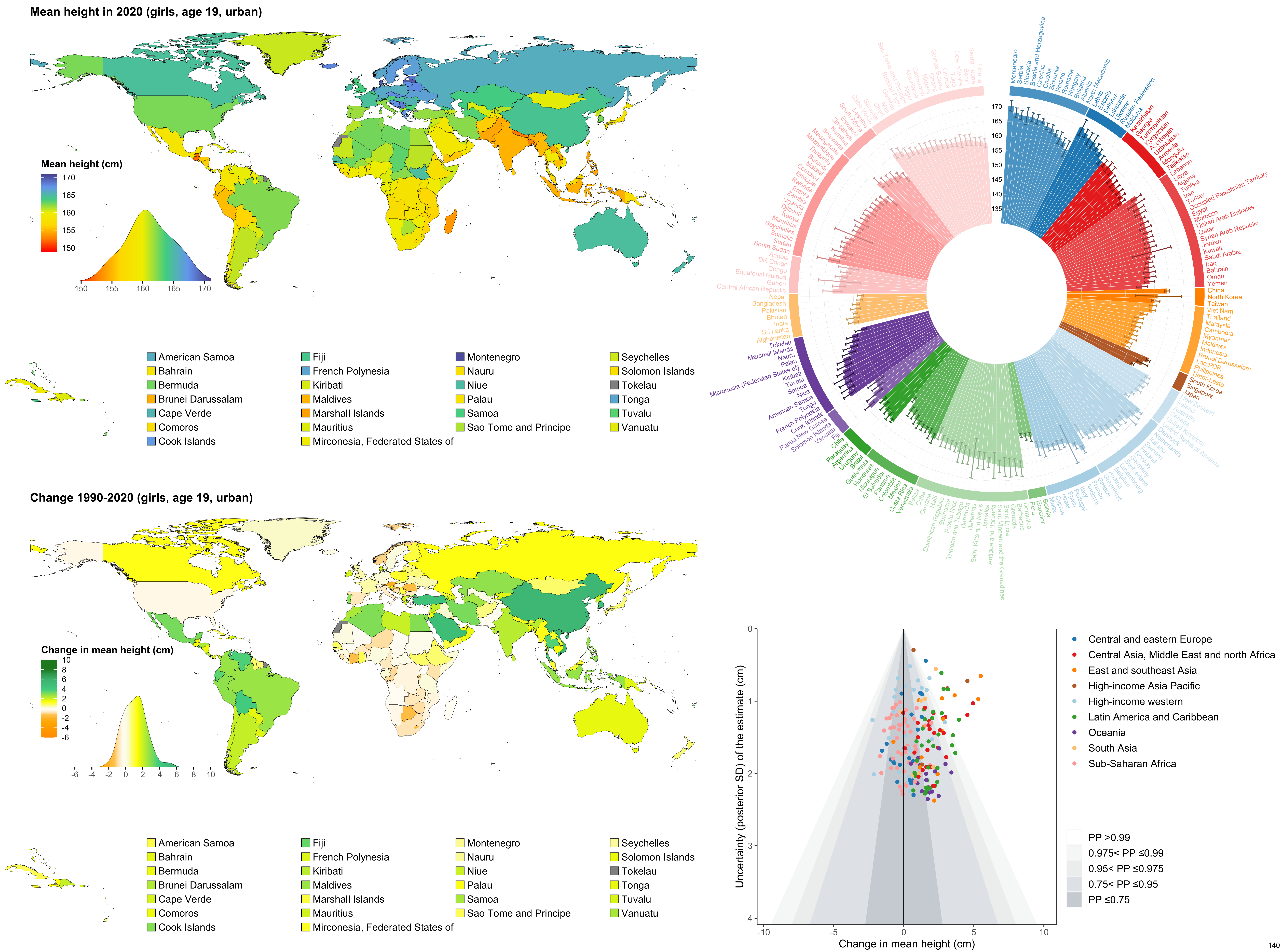
Mean height in 2020 (girls, age 19, urban)
Mean height (cm)
170
165
160
155
150
American Samoa
Bahrain
Bermuda
Brunei Darussalam
Cape Verde
Comoros
Cook Islands
Fiji
French Polynesia
Kiribati
Maldives
Marshall Islands
Mauritius
Mirconesia, Federated States of
Montenegro
Nauru
Niue
Palau
Samoa
Sao Tome and Principe
Seychelles
Solomon Islands
Tokelau
Tonga
Tuvalu
Vanuatu
Change 1990-2020 (girls, age 19, urban)
Change in mean height (cm)
10
8
6
4
2
0
-2
-4
-6
Uncertainty (posterior SD) of the estimate (cm)
Change in mean height (cm)
Central and eastern Europe
Central Asia, Middle East and north Africa
East and southeast Asia
High-income Asia Pacific
High-income western
Latin America and Caribbean
Oceania
South Asia
Sub-Saharan Africa
PP >0.99
0.975< PP ≤0.99
0.95< PP ≤0.975
0.75< PP ≤0.95
PP ≤0.75

Mean height in 2020 (girls, age 19, rural)

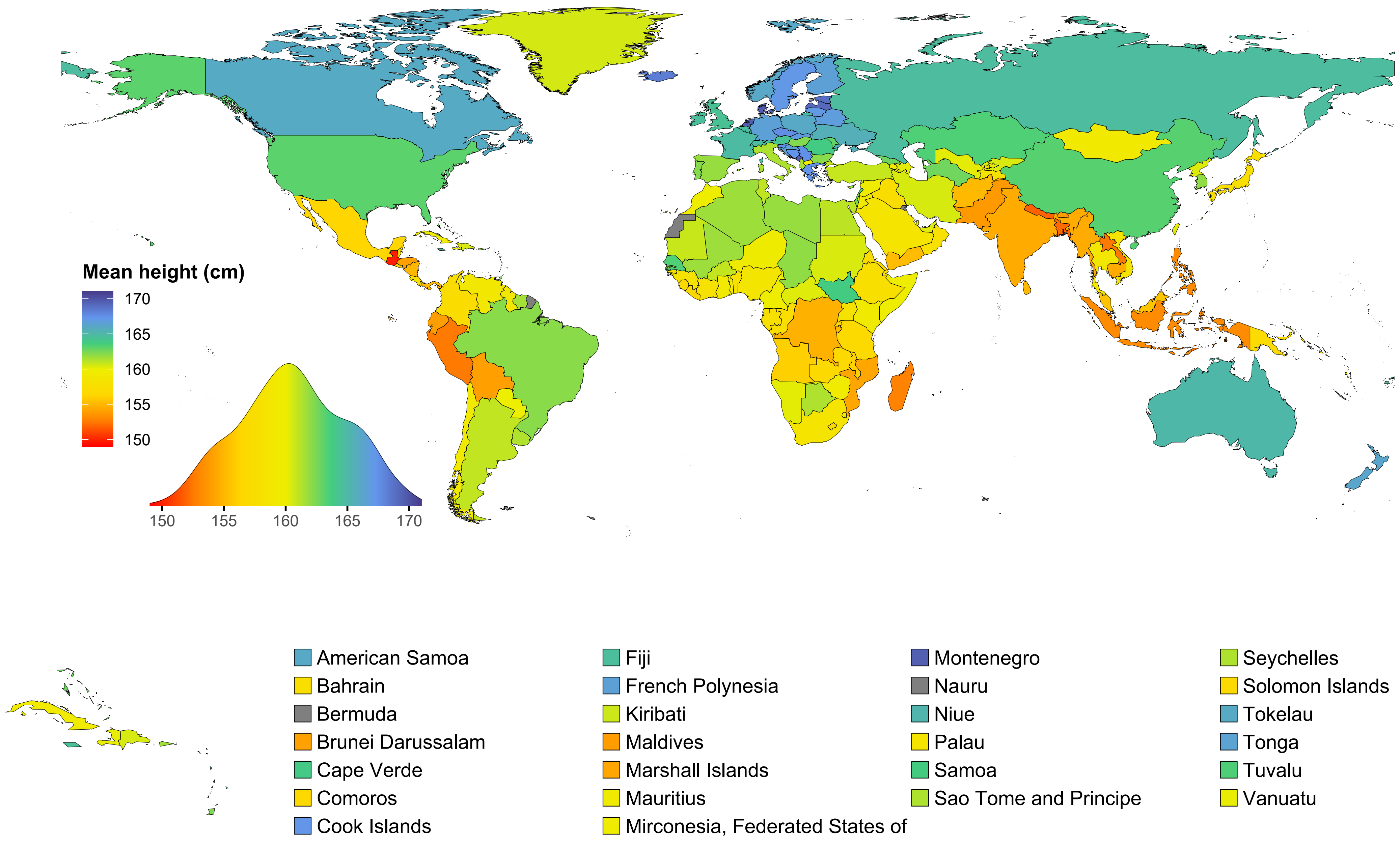

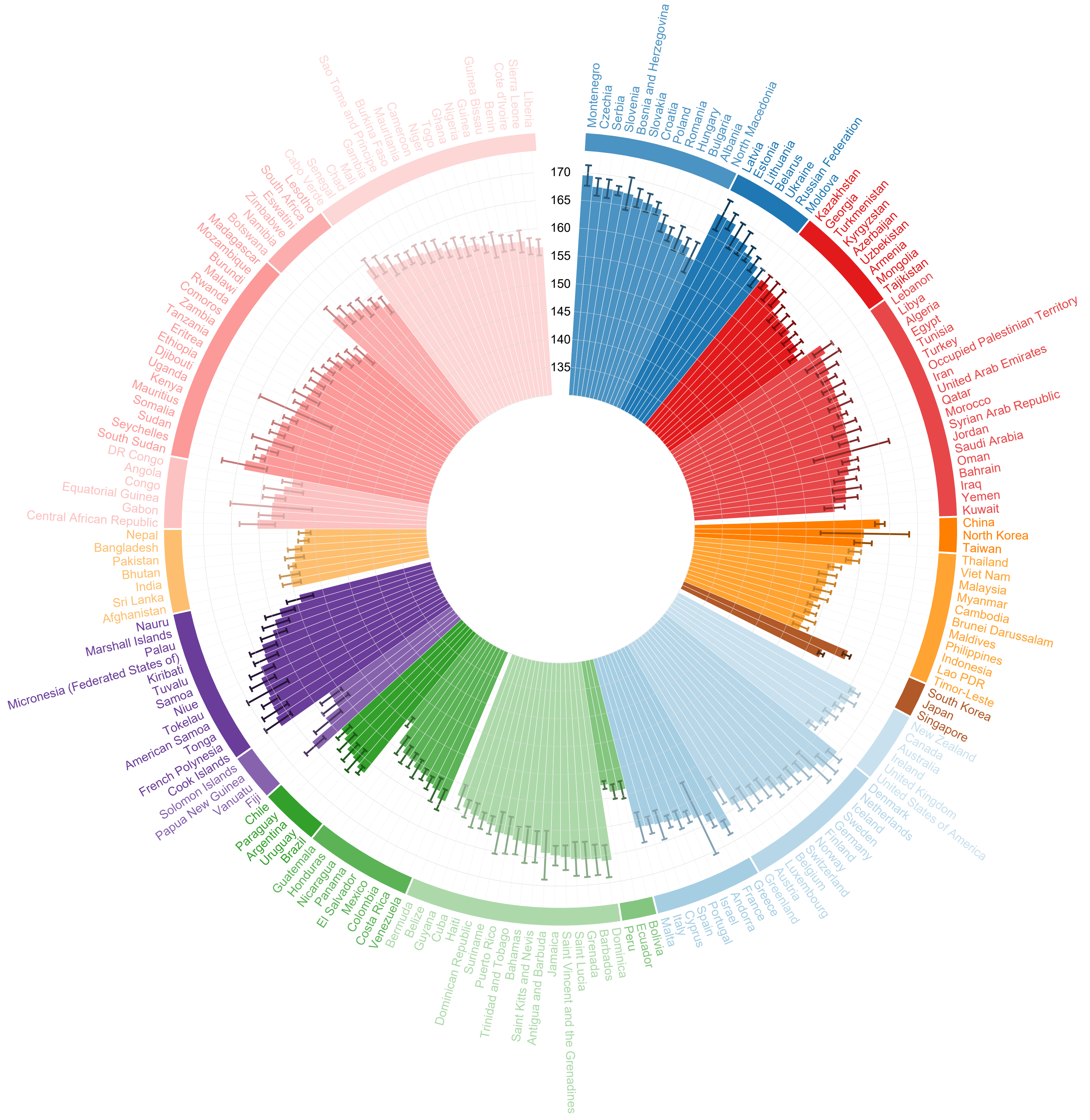

Change 1990-2020 (girls, age 19, rural)

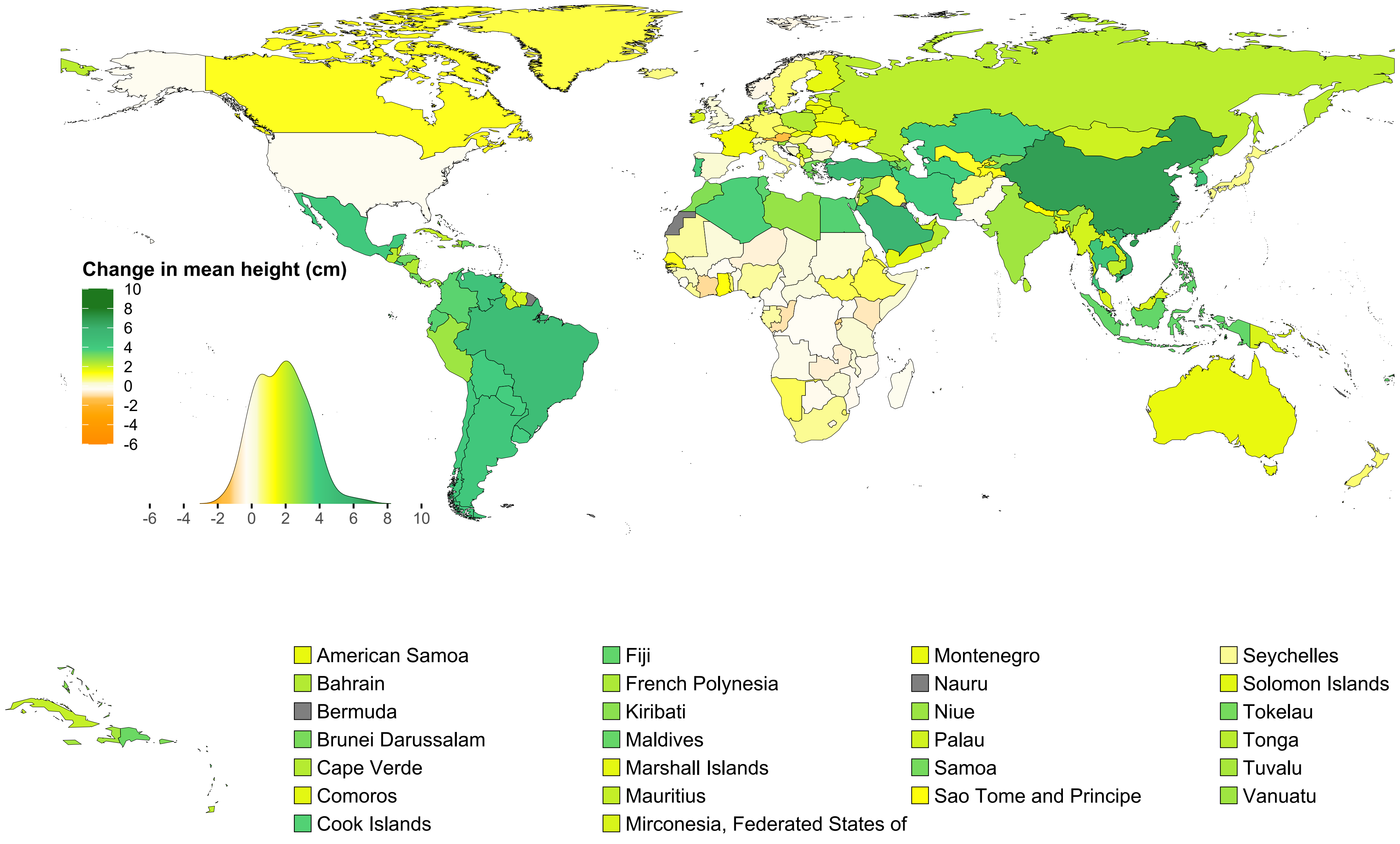

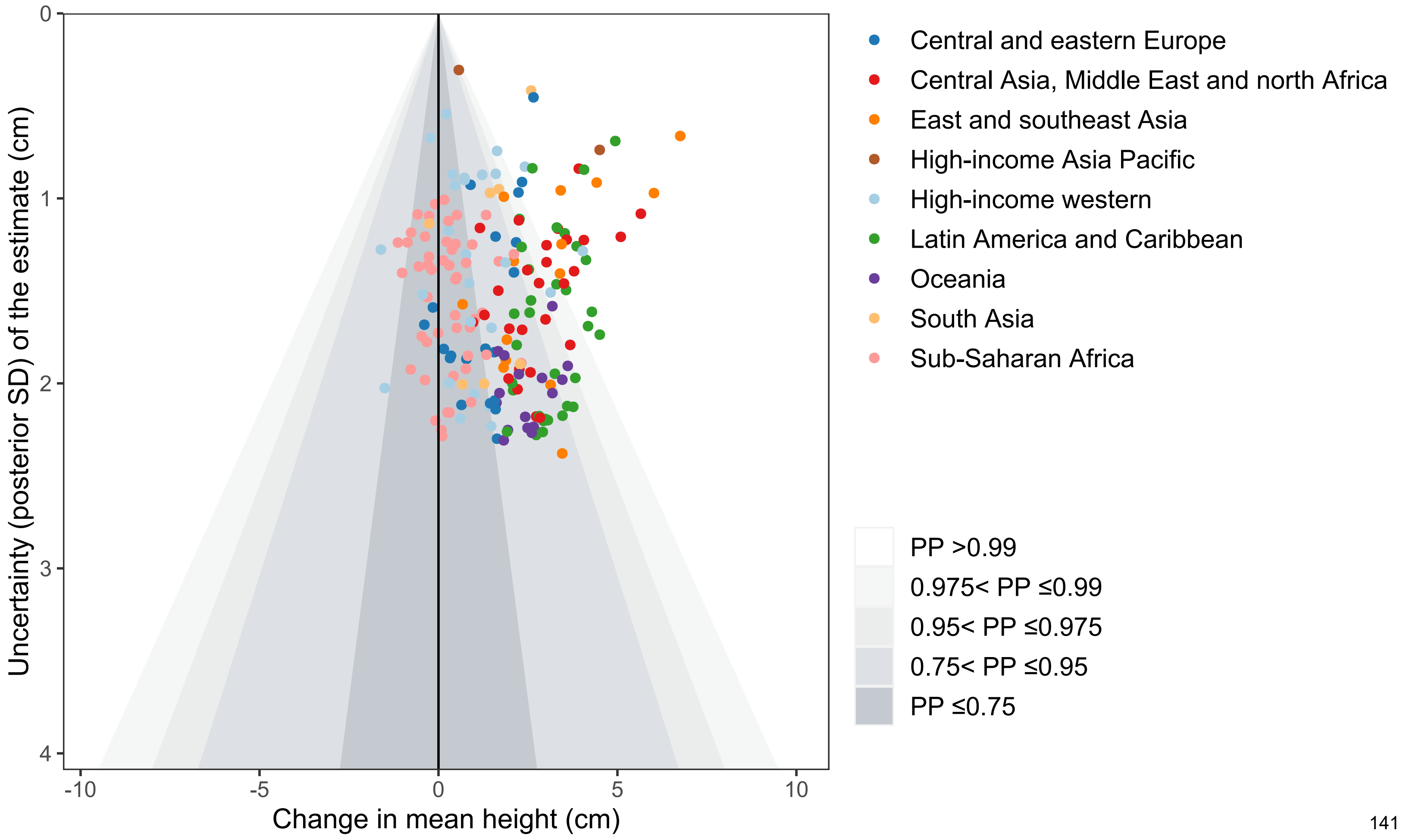

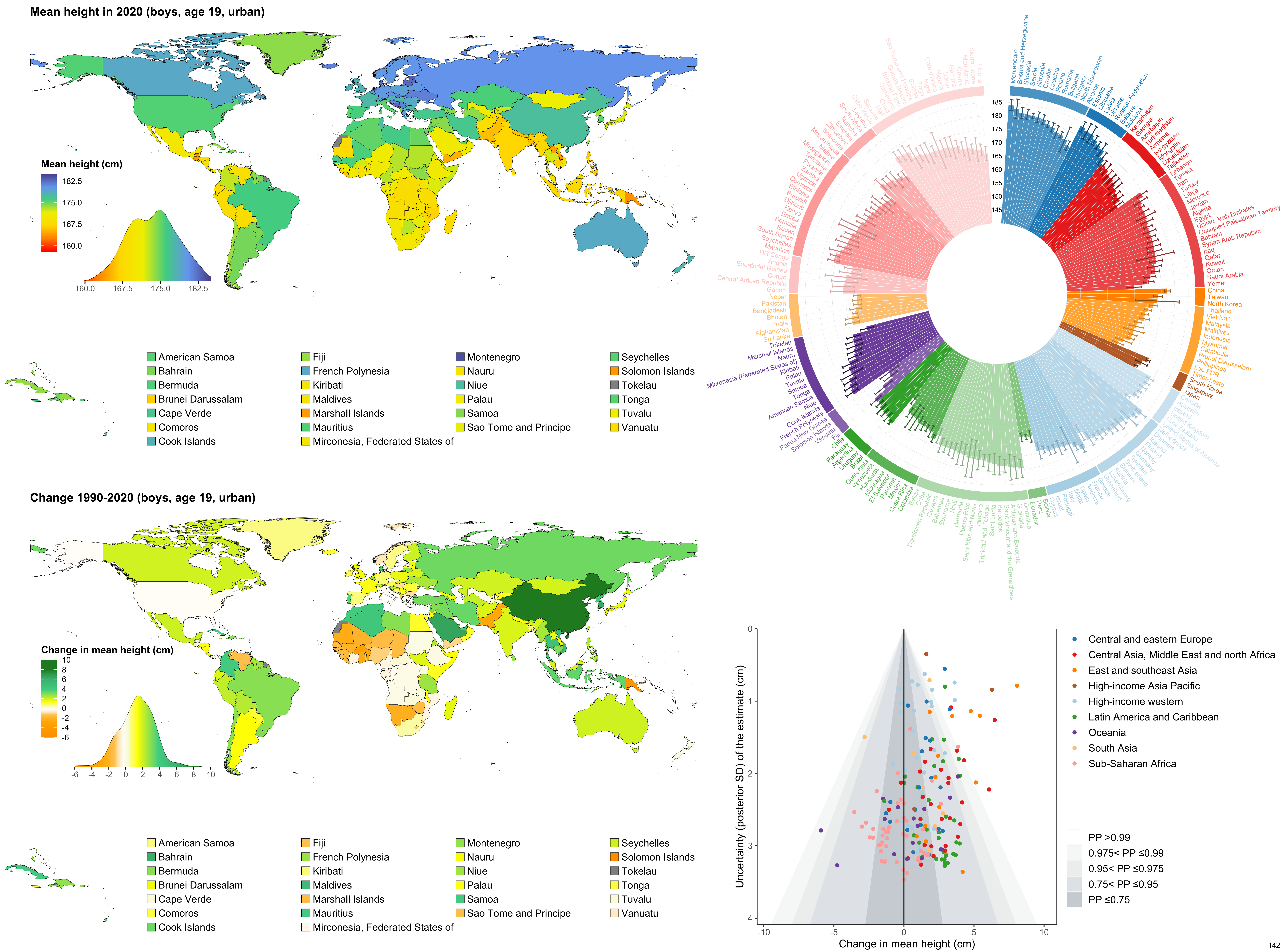
Mean height in 2020 (boys, age 19, urban)
Mean height (cm)
182.5
175.0
167.5
160.0
160.0 167.5 175.0 182.5
American Samoa
Bahrain
Bermuda
Brunei Darussalam
Cape Verde
Comoros
Cook Islands
Fiji
French Polynesia
Kiribati
Maldives
Marshall Islands
Mauritius
Mirconesia, Federated States of
Montenegro
Nauru
Niue
Palau
Samoa
Sao Tome and Principe
Seychelles
Solomon Islands
Tokelau
Tonga
Tuvalu
Vanuatu
Change 1990-2020 (boys, age 19, urban)
Change in mean height (cm)
10
8
6
4
2
0
-2
-4
-6
-6 -4 -2 0 2 4 6 8 10
American Samoa
Bahrain
Bermuda
Brunei Darussalam
Cape Verde
Comoros
Cook Islands
Fiji
French Polynesia
Kiribati
Maldives
Marshall Islands
Mauritius
Mirconesia, Federated States of
Montenegro
Nauru
Niue
Palau
Samoa
Sao Tome and Principe
Seychelles
Solomon Islands
Tokelau
Tonga
Tuvalu
Vanuatu
185
180
175
170
165
160
155
150
145
Montenegro
Bosnia and Herzegovina
Slovakia
Serbia
Slovenia
Croatia
Czechia
Poland
Romania
Bulgaria
Hungary
North Macedonia
Albania
Estonia
Lithuania
Latvia
Ukraine
Belarus
Moldova
Russian Federation
Kazakhstan
Georgia
Azerbaijan
Turkmenistan
Armenia
Kyrgyzstan
Mongolia
Uzbekistan
Tajikistan
Lebanon
Tunisia
Iran
Turkey
Libya
Morocco
Jordan
Algeria
Egypt
United Arab Emirates
Occupied Palestinian Territory
Bahrain
Syrian Arab Republic
Iraq
Qatar
Kuwait
Oman
Saudi Arabia
Yemen
China
Taiwan
North Korea
Thailand
Viet Nam
Malaysia
Maldives
Indonesia
Myanmar
Cambodia
Brunei Darussalam
Philippines
Lao PDR
Timor-Leste
South Korea
Singapore
Japan
Nepal
Pakistan
Bangladesh
Bhutan
India
Afghanistan
Sri Lanka
Tokelau
Marshall Islands
Nauru
Micronesia (Federated States of)
Kiribati
Palau
Tuvalu
Samoa
Tonga
American Samoa
Niue
Cook Islands
French Polynesia
Papua New Guinea
Solomon Islands
Vanuatu
Fiji
Chile
Paraguay
Argentina
Uruguay
Brazil
Guatemala
Venezuela
Honduras
Nicaragua
El Salvador
Panama
Mexico
Costa Rica
Colombia
Ecuador
Peru
Bolivia
Central African Republic
Gabon
Equatorial Guinea
Angola
DR Congo
Congo
Mauritius
Seychelles
South Sudan
Sudan
Somalia
Eritrea
Kenya
Djibouti
Egypt
Burundi
Ethiopia
Comoros
Uganda
Zambia
Rwanda
Tanzania
Madagascar
Malawi
Mozambique
Zimbabwe
Botswana
Eswatini
Namibia
South Africa
Lesotho
Liberia
Sierra Leone
Mauritania
Central and eastern Europe
Central Asia, Middle East and north Africa
East and southeast Asia
High-income Asia Pacific
High-income western
Latin America and Caribbean
Oceania
South Asia
Sub-Saharan Africa
Uncertainty (posterior SD) of the estimate (cm)
0
1
2
3
4
-10 -5 0 5 10
Change in mean height (cm)
PP >0.99
0.975< PP ≤0.99
0.95< PP ≤0.975
0.75< PP ≤0.95
PP ≤0.75

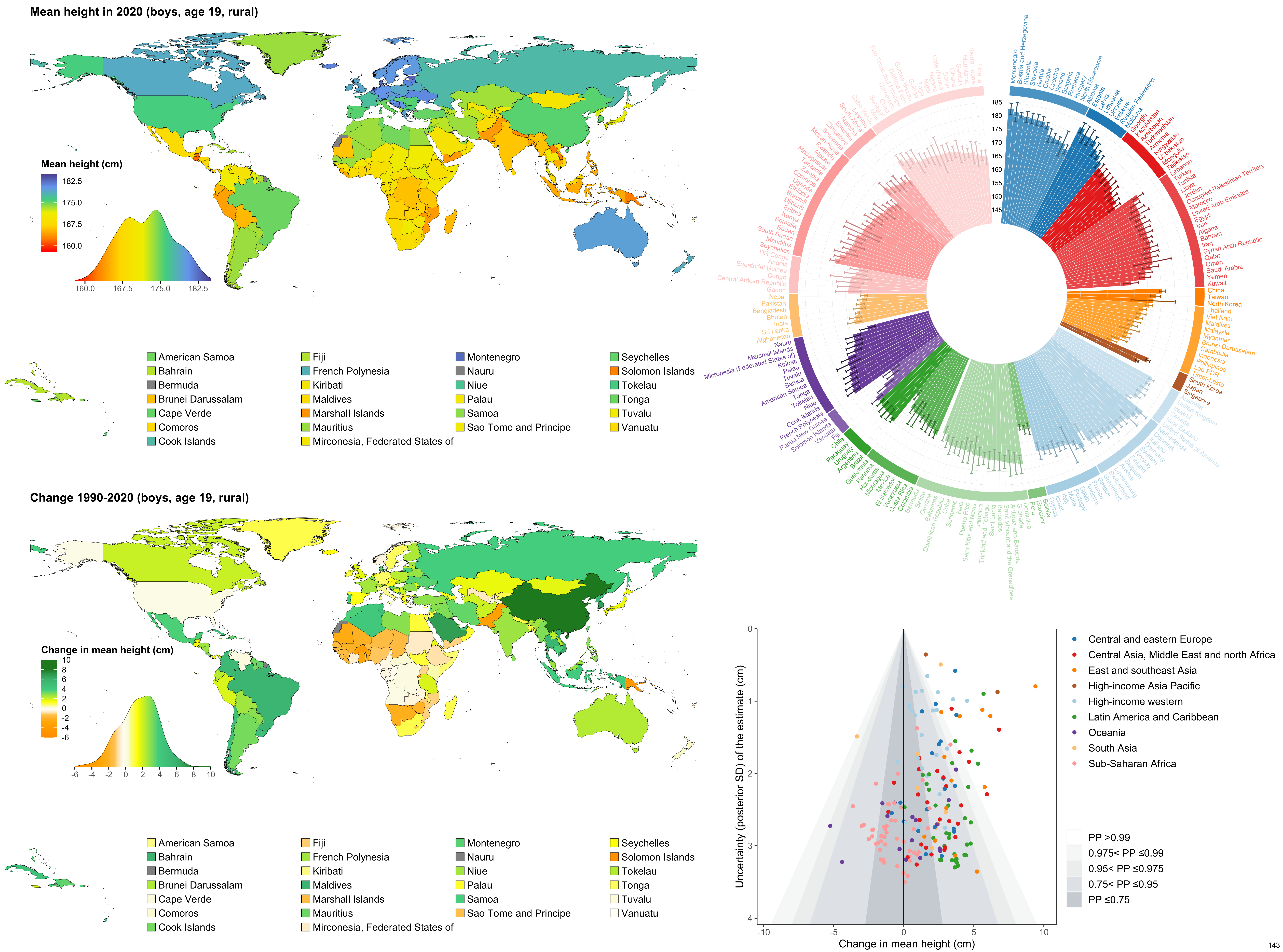

Mean height in 2020 (boys, age 19, rural)
Mean height (cm)
182.5
175.0
167.5
160.0
American Samoa
Bahrain
Bermuda
Brunei Darussalam
Cape Verde
Comoros
Cook Islands
Fiji
French Polynesia
Kiribati
Maldives
Marshall Islands
Mauritius
Mirconesia, Federated States of
Montenegro
Nauru
Niue
Palau
Samoa
Sao Tome and Principe
Seychelles
Solomon Islands
Tokelau
Tonga
Tuvalu
Vanuatu
Change 1990-2020 (boys, age 19, rural)
Change in mean height (cm)
10
8
6
4
2
0
-2
-4
-6
American Samoa
Bahrain
Bermuda
Brunei Darussalam
Cape Verde
Comoros
Cook Islands
Fiji
French Polynesia
Kiribati
Maldives
Marshall Islands
Mauritius
Mirconesia, Federated States of
Montenegro
Nauru
Niue
Palau
Samoa
Sao Tome and Principe
Seychelles
Solomon Islands
Tokelau
Tonga
Tuvalu
Vanuatu
185
180
175
170
165
160
155
150
145
Uncertainty (posterior SD) of the estimate (cm)
Change in mean height (cm)
Central and eastern Europe
Central Asia, Middle East and north Africa
East and southeast Asia
High-income Asia Pacific
High-income western
Latin America and Caribbean
Oceania
South Asia
Sub-Saharan Africa
PP >0.99
0.975< PP ≤0.99
0.95< PP ≤0.975
0.75< PP ≤0.95
PP ≤0.75

Supplementary Figure 6. Urban and rural body-mass index (BMI) in 2020 and change from 1990 to 2020, by age.

The maps show mean BMI in 2020 and change from 1990 to 2020, by urban and rural place of residence and country for girls and boys at ages 5, 10, 15 and 19 years. See Supplementary Figure 5 caption for descriptions of other sections of the figure.

We did not estimate mean rural BMI in areas classified as entirely urban (Bermuda, Kuwait, Nauru and Singapore), mean urban BMI in areas classified as entirely rural (Tokelau), or their change over time in these areas, as indicated by grey colour.

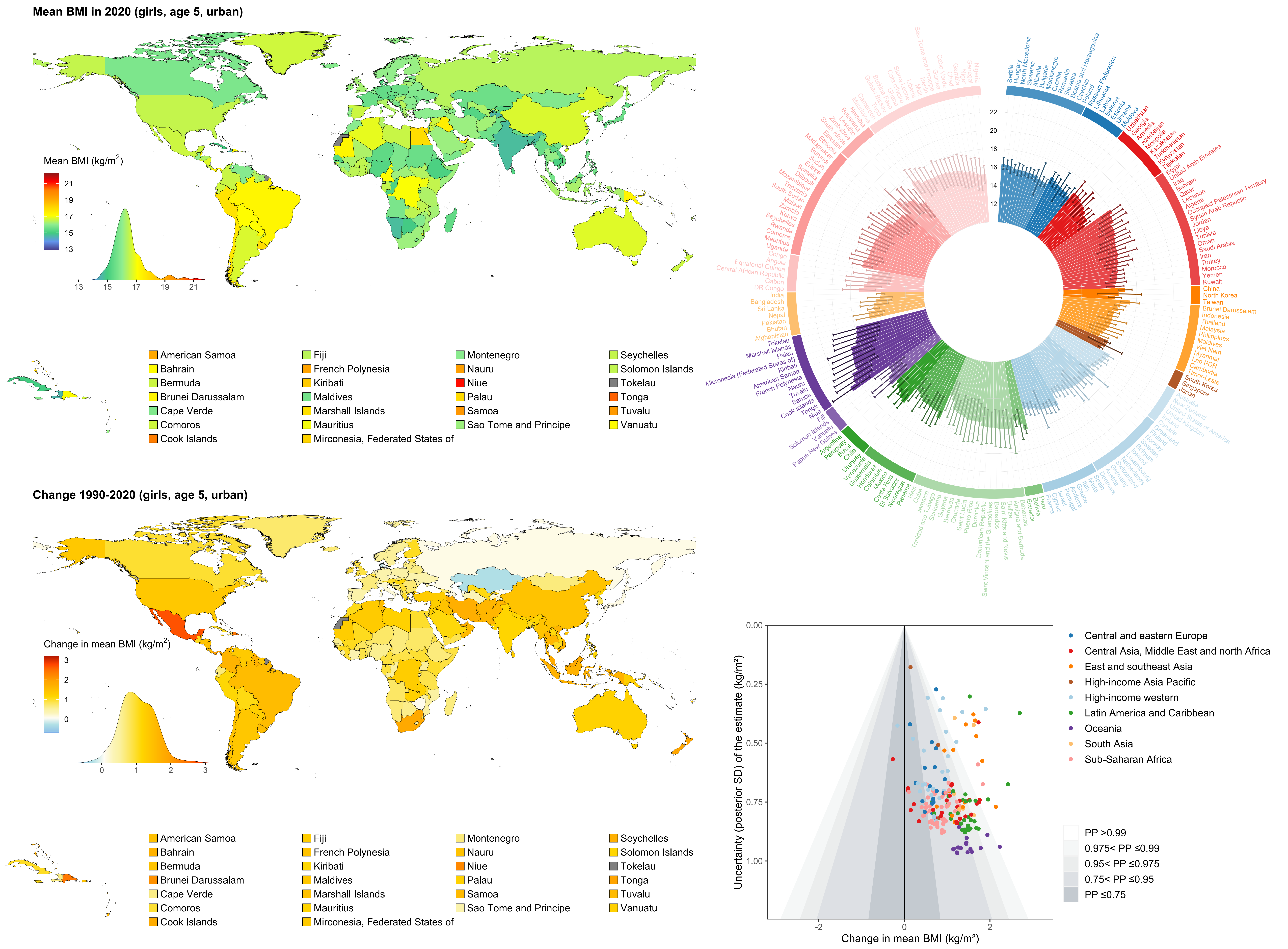
Mean BMI in 2020 (girls, age 5, urban)
Mean BMI (kg/m2)
21
19
17
15
13
American Samoa
Bahrain
Bermuda
Brunei Darussalam
Cape Verde
Comoros
Cook Islands
Fiji
French Polynesia
Kiribati
Maldives
Marshall Islands
Mauritius
Mirconesia, Federated States of
Montenegro
Nauru
Niue
Palau
Samoa
Sao Tome and Principe
Seychelles
Solomon Islands
Tokelau
Tonga
Tuvalu
Vanuatu
Change 1990-2020 (girls, age 5, urban)
Change in mean BMI (kg/m2)
3
2
1
0
Central and eastern Europe
Central Asia, Middle East and north Africa
East and southeast Asia
High-income Asia Pacific
High-income western
Latin America and Caribbean
Oceania
South Asia
Sub-Saharan Africa
PP >0.99
0.975< PP ≤0.99
0.95< PP ≤0.975
0.75< PP ≤0.95
PP ≤0.75
Uncertainty (posterior SD) of the estimate (kg/m²)
Change in mean BMI (kg/m²)

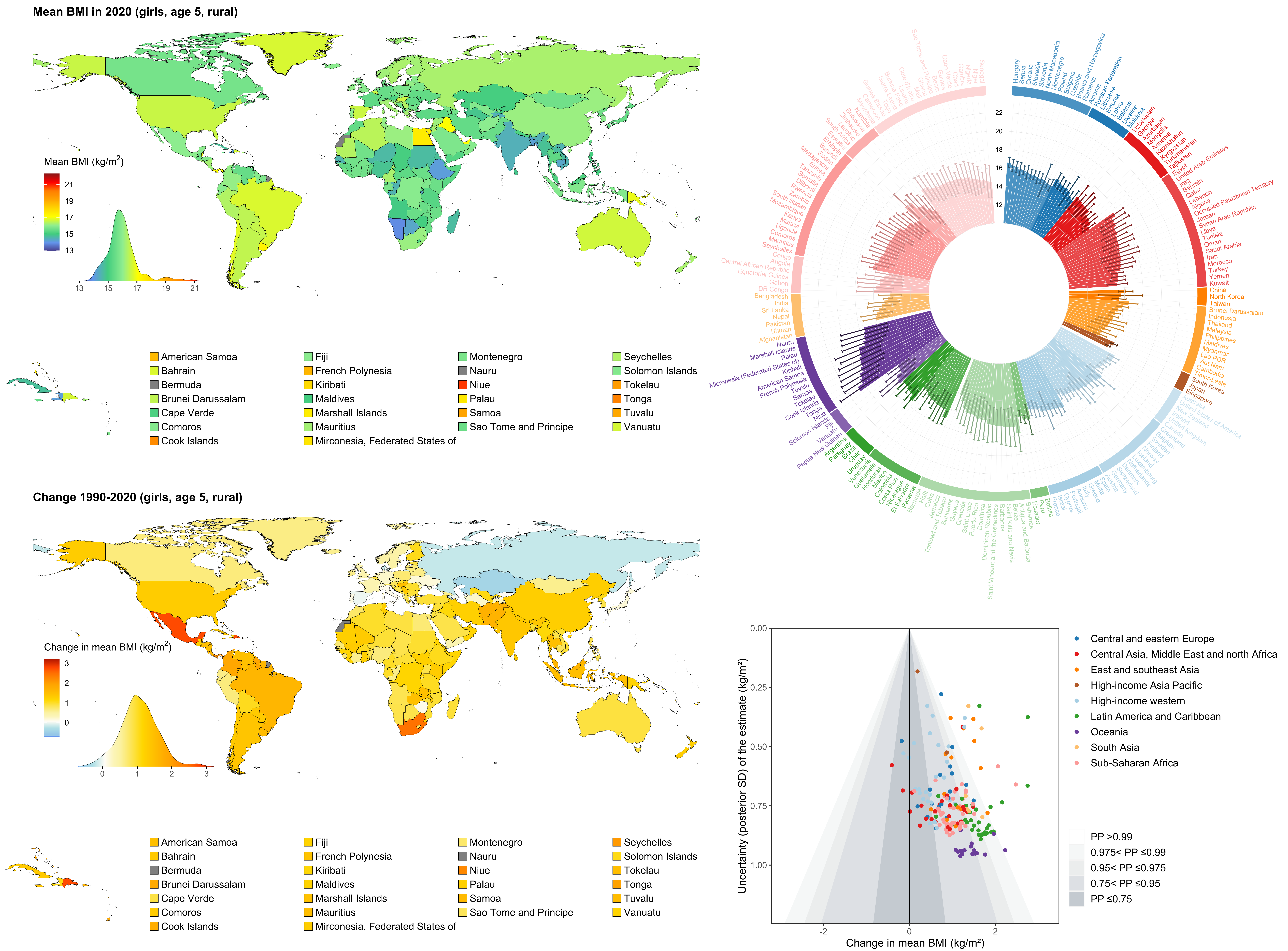

Mean BMI in 2020 (girls, age 5, rural)
Mean BMI (kg/m²)
21
19
17
15
13
American Samoa
Bahrain
Bermuda
Brunei Darussalam
Cape Verde
Comoros
Cook Islands
Fiji
French Polynesia
Kiribati
Maldives
Marshall Islands
Mauritius
Mirconesia, Federated States of
Montenegro
Nauru
Niue
Palau
Samoa
Sao Tome and Principe
Seychelles
Solomon Islands
Tokelau
Tonga
Tuvalu
Vanuatu
Change 1990-2020 (girls, age 5, rural)
Change in mean BMI (kg/m²)
3
2
1
0
Uncertainty (posterior SD) of the estimate (kg/m²)
0.00
0.25
0.50
0.75
1.00
Change in mean BMI (kg/m²)
-2
0
2
Central and eastern Europe
Central Asia, Middle East and north Africa
East and southeast Asia
High-income Asia Pacific
High-income western
Latin America and Caribbean
Oceania
South Asia
Sub-Saharan Africa
PP >0.99
0.975< PP ≤0.99
0.95< PP ≤0.975
0.75< PP ≤0.95
PP ≤0.75

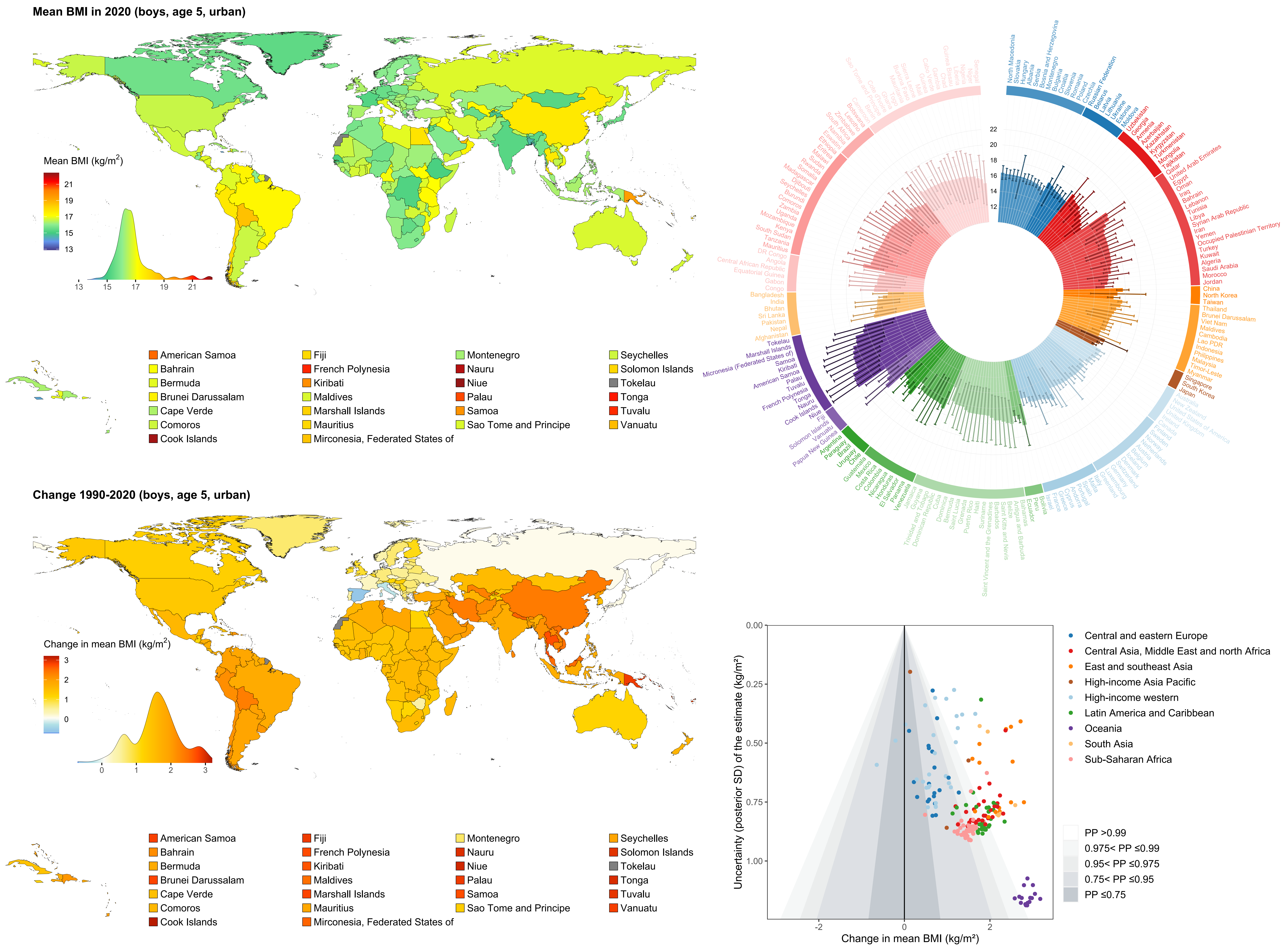

Mean BMI in 2020 (boys, age 5, urban)
Mean BMI (kg/m2)
21
19
17
15
13
American Samoa
Bahrain
Bermuda
Brunei Darussalam
Cape Verde
Comoros
Cook Islands
Fiji
French Polynesia
Kiribati
Maldives
Marshall Islands
Mauritius
Mirconesia, Federated States of
Montenegro
Nauru
Niue
Palau
Samoa
Sao Tome and Principe
Seychelles
Solomon Islands
Tokelau
Tonga
Tuvalu
Vanuatu
Change 1990-2020 (boys, age 5, urban)
Change in mean BMI (kg/m2)
3
2
1
0
Central and eastern Europe
Central Asia, Middle East and north Africa
East and southeast Asia
High-income Asia Pacific
High-income western
Latin America and Caribbean
Oceania
South Asia
Sub-Saharan Africa
PP >0.99
0.975< PP ≤0.99
0.95< PP ≤0.975
0.75< PP ≤0.95
PP ≤0.75
Uncertainty (posterior SD) of the estimate (kg/m²)
Change in mean BMI (kg/m²)

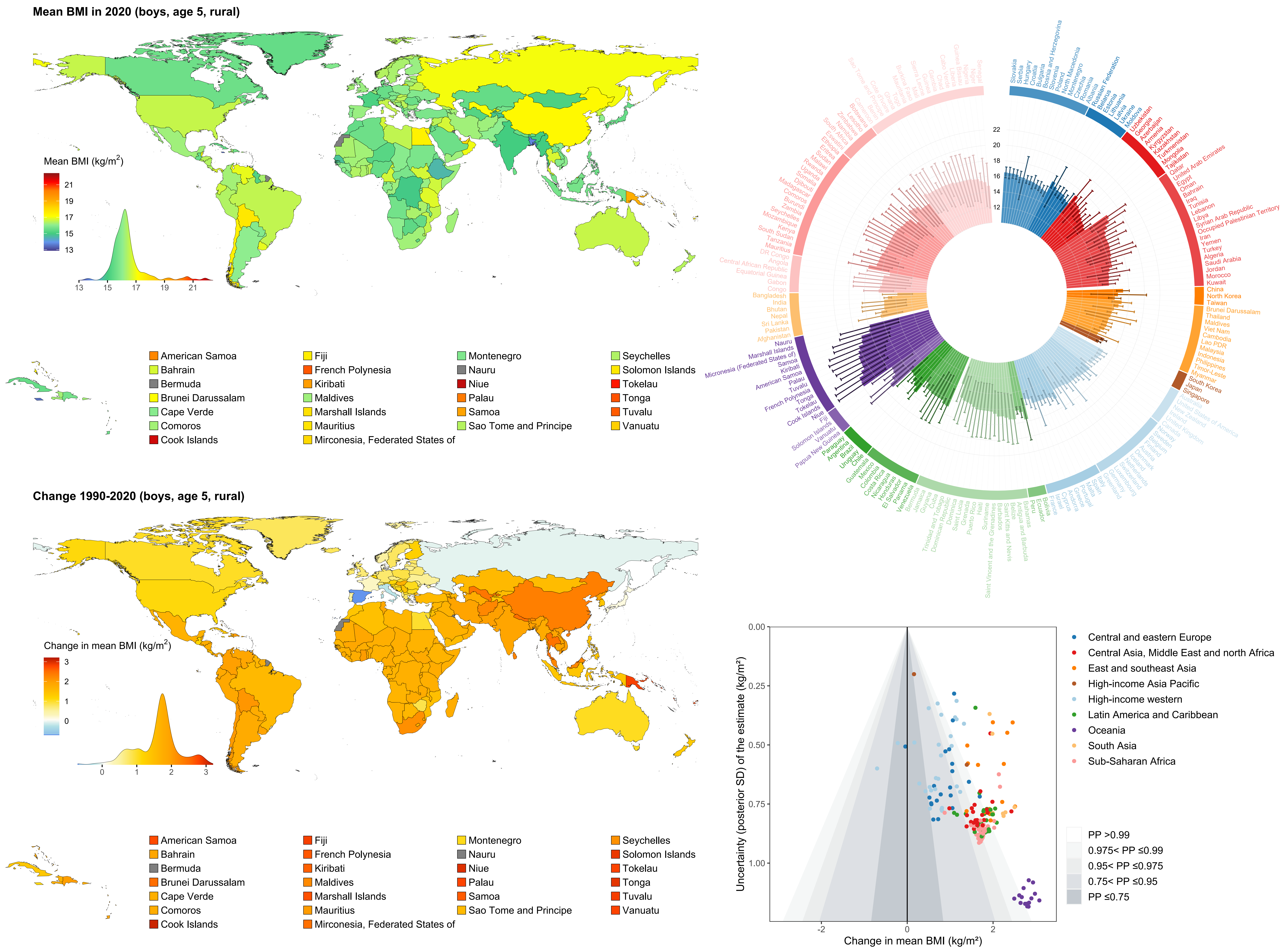

Mean BMI in 2020 (boys, age 5, rural)
Mean BMI (kg/m^2)
21
19
17
15
13
American Samoa
Bahrain
Bermuda
Brunei Darussalam
Cape Verde
Comoros
Cook Islands
Fiji
French Polynesia
Kiribati
Maldives
Marshall Islands
Mauritius
Mirconesia, Federated States of
Montenegro
Nauru
Niue
Palau
Samoa
Sao Tome and Principe
Seychelles
Solomon Islands
Tokelau
Tonga
Tuvalu
Vanuatu
Change 1990-2020 (boys, age 5, rural)
Change in mean BMI (kg/m^2)
3
2
1
0
American Samoa
Bahrain
Bermuda
Brunei Darussalam
Cape Verde
Comoros
Cook Islands
Fiji
French Polynesia
Kiribati
Maldives
Marshall Islands
Mauritius
Mirconesia, Federated States of
Montenegro
Nauru
Niue
Palau
Samoa
Sao Tome and Principe
Seychelles
Solomon Islands
Tokelau
Tonga
Tuvalu
Vanuatu
22
20
18
16
14
12
Uncertainty (posterior SD) of the estimate (kg/m²)
0.00
0.25
0.50
0.75
1.00
-2
0
2
Change in mean BMI (kg/m²)
Central and eastern Europe
Central Asia, Middle East and north Africa
East and southeast Asia
High-income Asia Pacific
High-income western
Latin America and Caribbean
Oceania
South Asia
Sub-Saharan Africa
PP >0.99
0.975< PP ≤0.99
0.95< PP ≤0.975
0.75< PP ≤0.95
PP ≤0.75

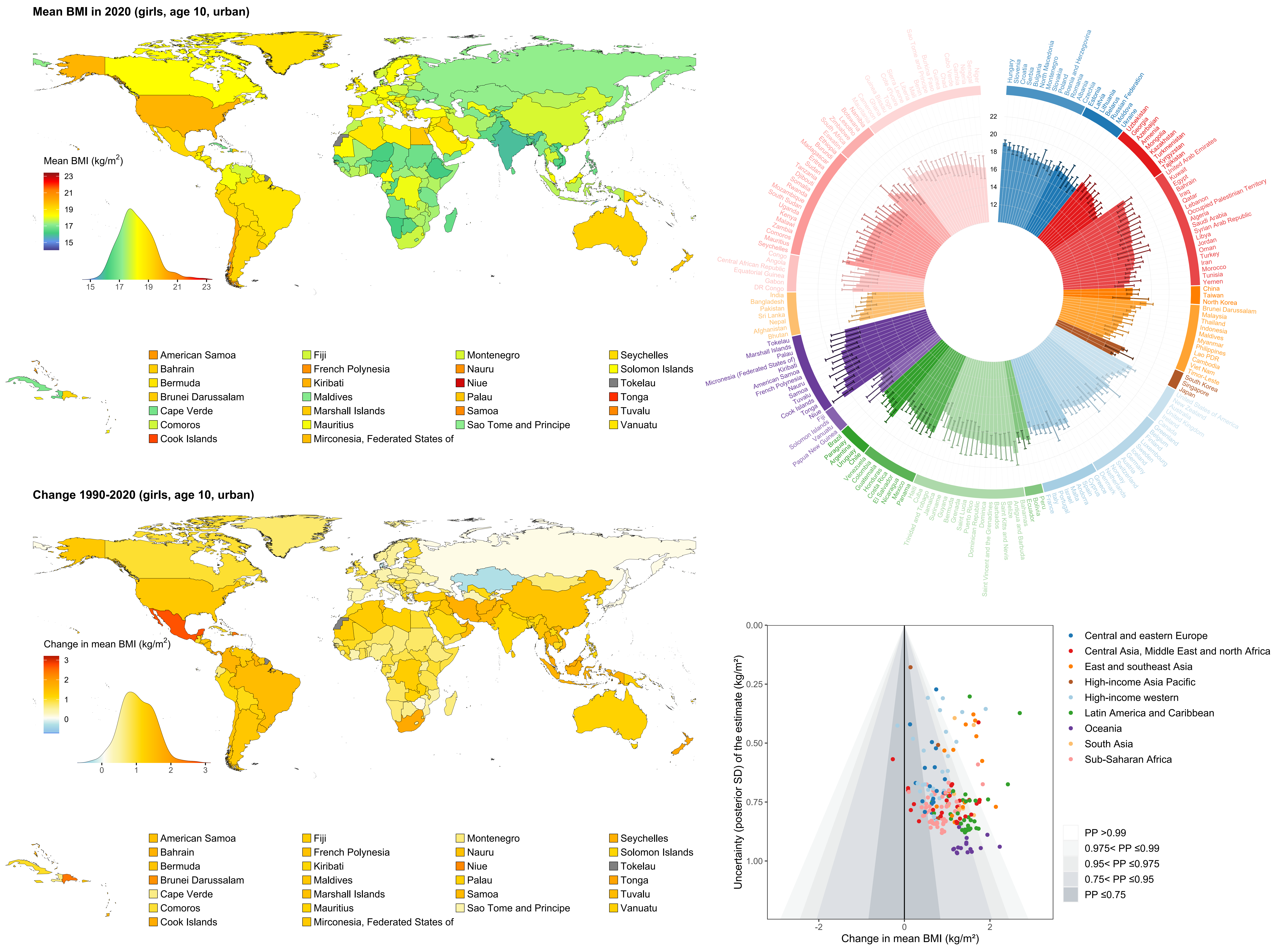
Mean BMI in 2020 (girls, age 10, urban)
Mean BMI (kg/m²)
23
21
19
17
15
American Samoa
Bahrain
Bermuda
Brunei Darussalam
Cape Verde
Comoros
Cook Islands
Fiji
French Polynesia
Kiribati
Maldives
Marshall Islands
Mauritius
Mirconesia, Federated States of
Montenegro
Nauru
Niue
Palau
Samoa
Sao Tome and Principe
Seychelles
Solomon Islands
Tokelau
Tonga
Tuvalu
Vanuatu
Change 1990-2020 (girls, age 10, urban)
Change in mean BMI (kg/m²)
3
2
1
0
American Samoa
Bahrain
Bermuda
Brunei Darussalam
Cape Verde
Comoros
Cook Islands
Fiji
French Polynesia
Kiribati
Maldives
Marshall Islands
Mauritius
Mirconesia, Federated States of
Montenegro
Nauru
Niue
Palau
Samoa
Sao Tome and Principe
Seychelles
Solomon Islands
Tokelau
Tonga
Tuvalu
Vanuatu
22
20
18
16
14
12
Uncertainty (posterior SD) of the estimate (kg/m²)
Change in mean BMI (kg/m²)
0.00
0.25
0.50
0.75
1.00
-2
0
2
Central and eastern Europe
Central Asia, Middle East and north Africa
East and southeast Asia
High-income Asia Pacific
High-income western
Latin America and Caribbean
Oceania
South Asia
Sub-Saharan Africa
PP >0.99
0.975< PP ≤0.99
0.95< PP ≤0.975
0.75< PP ≤0.95
PP ≤0.75

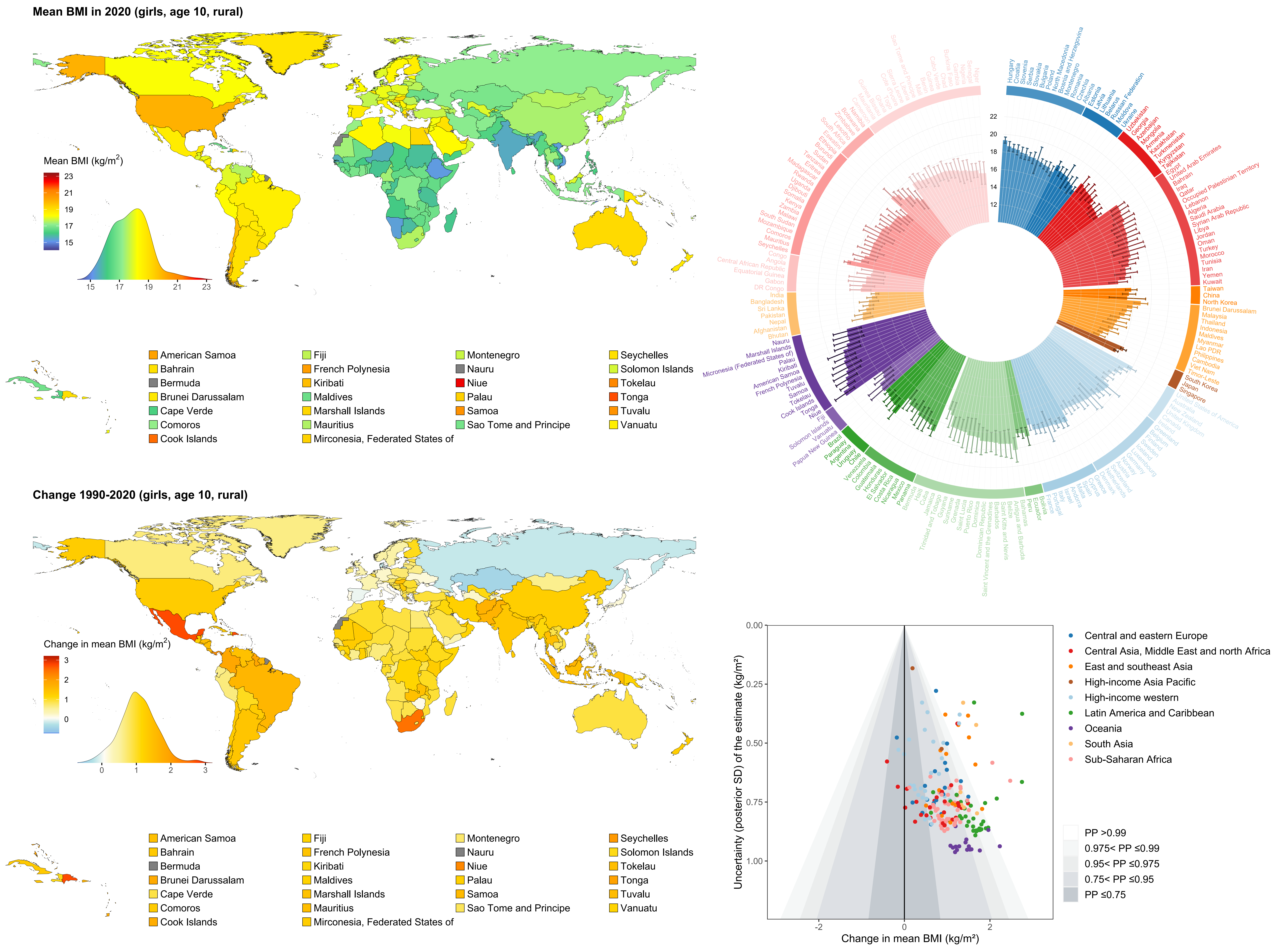
Mean BMI in 2020 (girls, age 10, rural)
Mean BMI (kg/m²)
23
21
19
17
15
15 17 19 21 23
American Samoa
Bahrain
Bermuda
Brunei Darussalam
Cape Verde
Comoros
Cook Islands
Fiji
French Polynesia
Kiribati
Maldives
Marshall Islands
Mauritius
Mirconesia, Federated States of
Montenegro
Nauru
Niue
Palau
Samoa
Sao Tome and Principe
Seychelles
Solomon Islands
Tokelau
Tonga
Tuvalu
Vanuatu
Change 1990-2020 (girls, age 10, rural)
Change in mean BMI (kg/m²)
3
2
1
0
0 1 2 3
American Samoa
Bahrain
Bermuda
Brunei Darussalam
Cape Verde
Comoros
Cook Islands
Fiji
French Polynesia
Kiribati
Maldives
Marshall Islands
Mauritius
Mirconesia, Federated States of
Montenegro
Nauru
Niue
Palau
Samoa
Sao Tome and Principe
Seychelles
Solomon Islands
Tokelau
Tonga
Tuvalu
Vanuatu
22
20
18
16
14
12
Uncertainty (posterior SD) of the estimate (kg/m²)
0.00
0.25
0.50
0.75
1.00
-2
0
2
Change in mean BMI (kg/m²)
Central and eastern Europe
Central Asia, Middle East and north Africa
East and southeast Asia
High-income Asia Pacific
High-income western
Latin America and Caribbean
Oceania
South Asia
Sub-Saharan Africa
PP >0.99
0.975< PP ≤0.99
0.95< PP ≤0.975
0.75< PP ≤0.95
PP ≤0.75

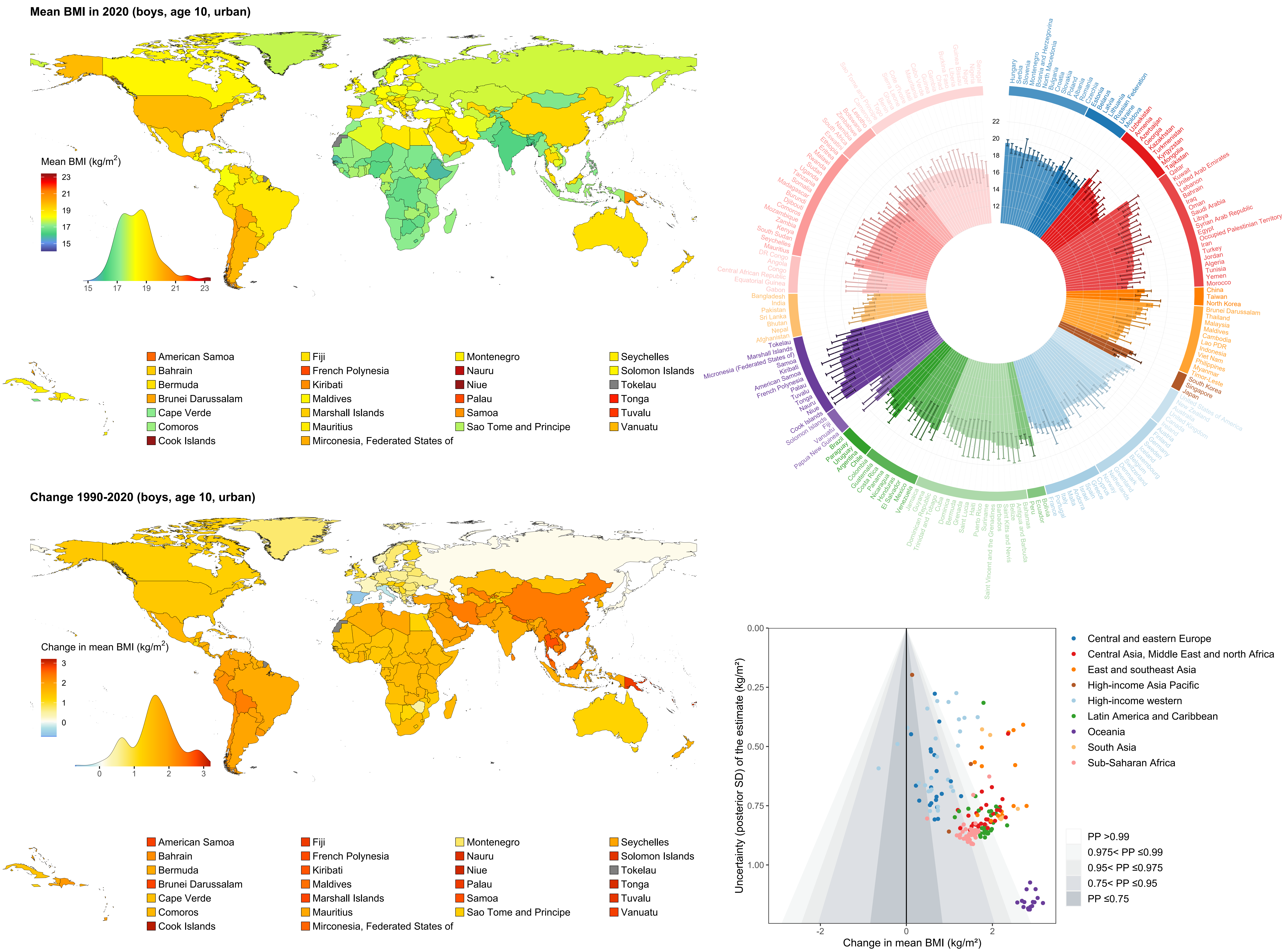
Mean BMI in 2020 (boys, age 10, urban)
Mean BMI (kg/m2)
23
21
19
17
15
15 17 19 21 23
American Samoa
Bahrain
Bermuda
Brunei Darussalam
Cape Verde
Comoros
Cook Islands
Fiji
French Polynesia
Kiribati
Maldives
Marshall Islands
Mauritius
Mirconesia, Federated States of
Montenegro
Nauru
Niue
Palau
Samoa
Sao Tome and Principe
Seychelles
Solomon Islands
Tokelau
Tonga
Tuvalu
Vanuatu
Change 1990-2020 (boys, age 10, urban)
Change in mean BMI (kg/m2)
3
2
1
0
0 1 2 3
American Samoa
Bahrain
Bermuda
Brunei Darussalam
Cape Verde
Comoros
Cook Islands
Fiji
French Polynesia
Kiribati
Maldives
Marshall Islands
Mauritius
Mirconesia, Federated States of
Montenegro
Nauru
Niue
Palau
Samoa
Sao Tome and Principe
Seychelles
Solomon Islands
Tokelau
Tonga
Tuvalu
Vanuatu
22
20
18
16
14
12
Uncertainty (posterior SD) of the estimate (kg/m²)
0.00
0.25
0.50
0.75
1.00
-2
0
2
Change in mean BMI (kg/m²)
Central and eastern Europe
Central Asia, Middle East and north Africa
East and southeast Asia
High-income Asia Pacific
High-income western
Latin America and Caribbean
Oceania
South Asia
Sub-Saharan Africa
PP >0.99
0.975< PP ≤0.99
0.95< PP ≤0.975
0.75< PP ≤0.95
PP ≤0.75

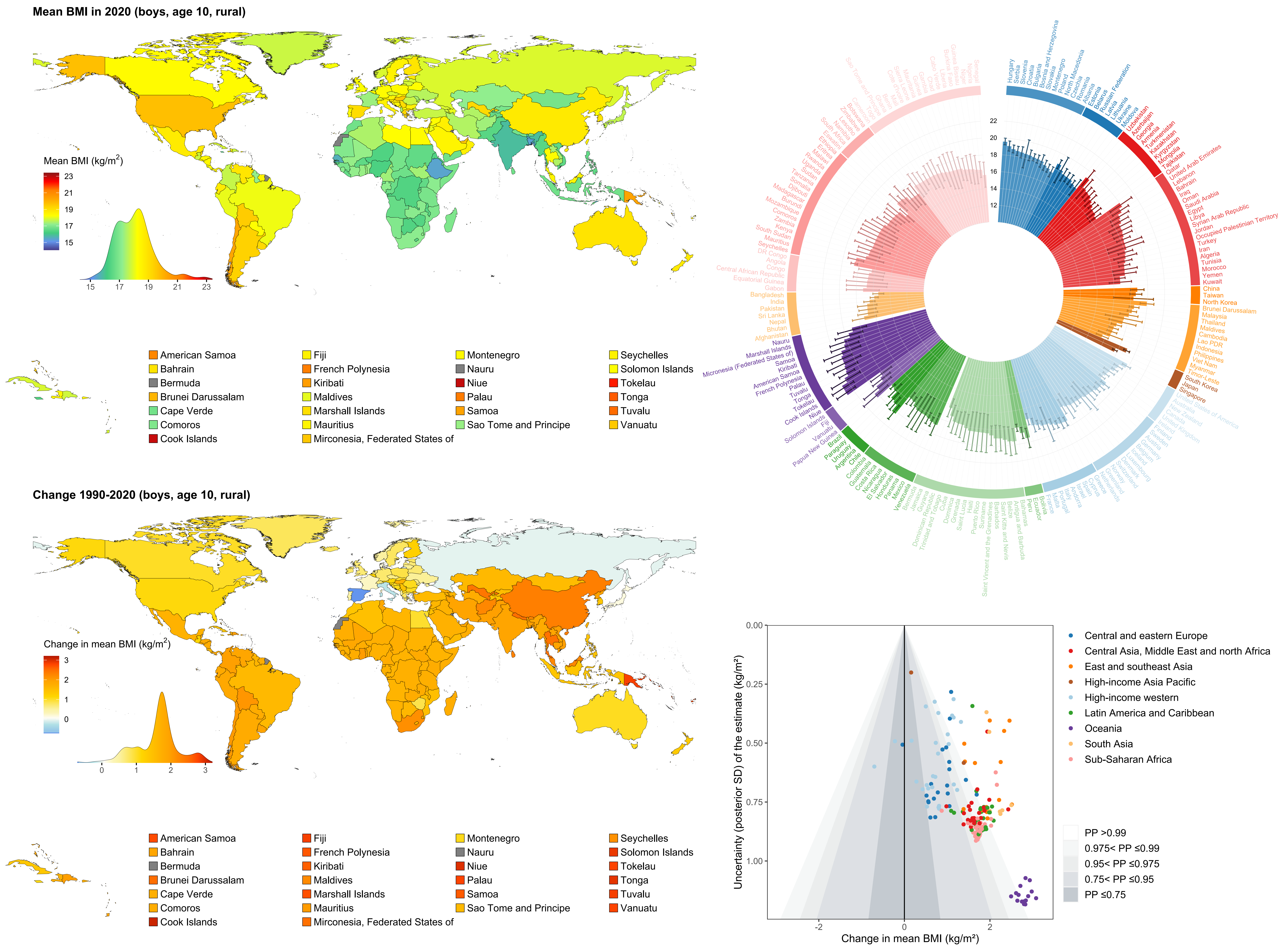

Mean BMI in 2020 (boys, age 10, rural)
Mean BMI (kg/m²)
23
21
19
17
15
15 17 19 21 23
American Samoa
Bahrain
Bermuda
Brunei Darussalam
Cape Verde
Comoros
Cook Islands
Fiji
French Polynesia
Kiribati
Maldives
Marshall Islands
Mauritius
Mirconesia, Federated States of
Montenegro
Nauru
Niue
Palau
Samoa
Sao Tome and Principe
Seychelles
Solomon Islands
Tokelau
Tonga
Tuvalu
Vanuatu
Change 1990-2020 (boys, age 10, rural)
Change in mean BMI (kg/m²)
3
2
1
0
0 1 2 3
American Samoa
Bahrain
Bermuda
Brunei Darussalam
Cape Verde
Comoros
Cook Islands
Fiji
French Polynesia
Kiribati
Maldives
Marshall Islands
Mauritius
Mirconesia, Federated States of
Montenegro
Nauru
Niue
Palau
Samoa
Sao Tome and Principe
Seychelles
Solomon Islands
Tokelau
Tonga
Tuvalu
Vanuatu
22
20
18
16
14
12
Uncertainty (posterior SD) of the estimate (kg/m²)
0.00
0.25
0.50
0.75
1.00
-2
0
2
Change in mean BMI (kg/m²)
Central and eastern Europe
Central Asia, Middle East and north Africa
East and southeast Asia
High-income Asia Pacific
High-income western
Latin America and Caribbean
Oceania
South Asia
Sub-Saharan Africa
PP >0.99
0.975< PP ≤0.99
0.95< PP ≤0.975
0.75< PP ≤0.95
PP ≤0.75

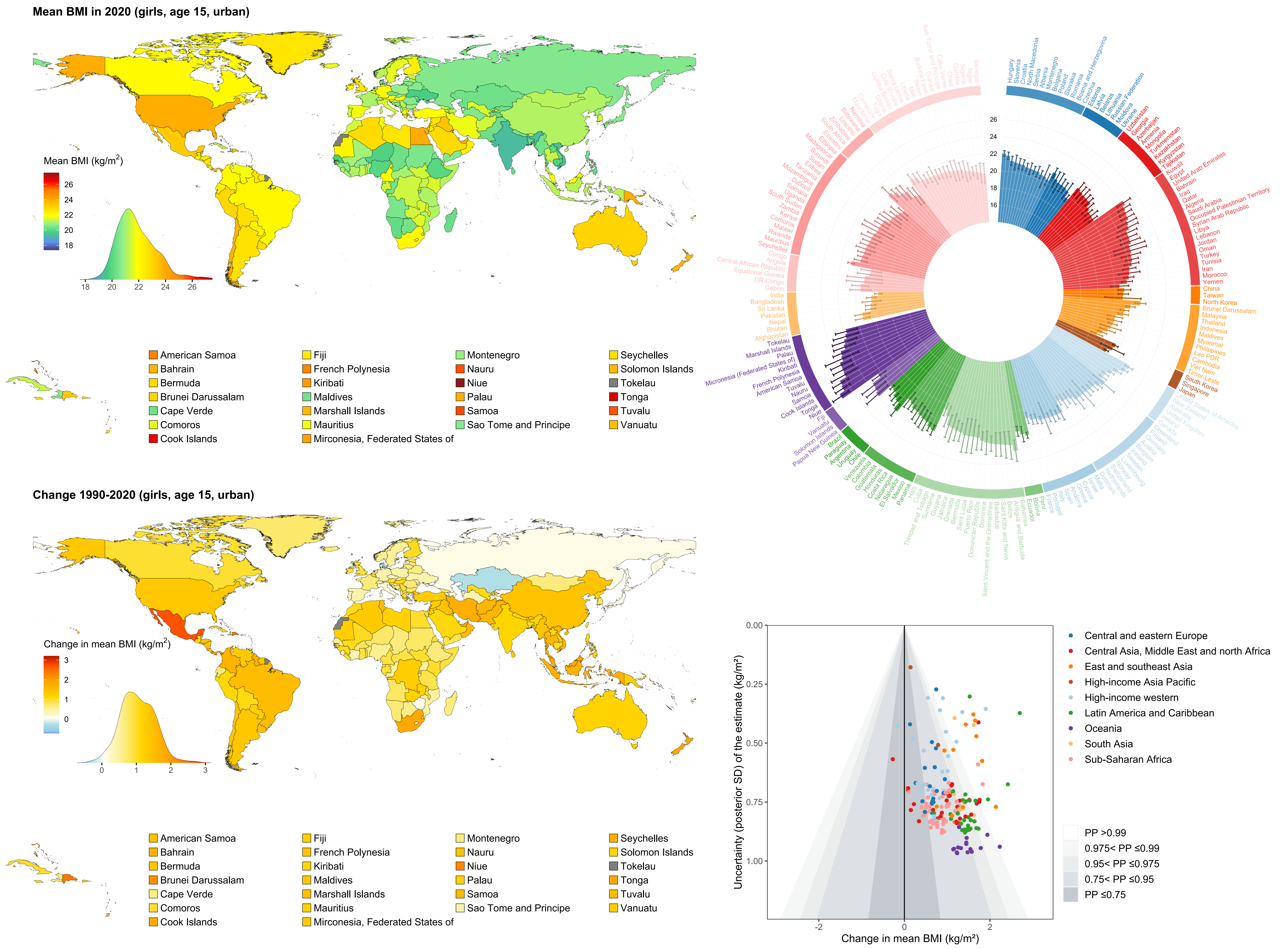

Mean BMI in 2020 (girls, age 15, urban)
Mean BMI (kg/m²)
26
24
22
20
18
18 20 22 24 26
American Samoa
Bahrain
Bermuda
Brunei Darussalam
Cape Verde
Comoros
Cook Islands
Fiji
French Polynesia
Kiribati
Maldives
Marshall Islands
Mauritius
Mirconesia, Federated States of
Montenegro
Nauru
Niue
Palau
Samoa
Sao Tome and Principe
Seychelles
Solomon Islands
Tokelau
Tonga
Tuvalu
Vanuatu
Change 1990-2020 (girls, age 15, urban)
Change in mean BMI (kg/m²)
3
2
1
0
0 1 2 3
American Samoa
Bahrain
Bermuda
Brunei Darussalam
Cape Verde
Comoros
Cook Islands
Fiji
French Polynesia
Kiribati
Maldives
Marshall Islands
Mauritius
Mirconesia, Federated States of
Montenegro
Nauru
Niue
Palau
Samoa
Sao Tome and Principe
Seychelles
Solomon Islands
Tokelau
Tonga
Tuvalu
Vanuatu
26
24
22
20
18
16
Uncertainty (posterior SD) of the estimate (kg/m²)
0.00
0.25
0.50
0.75
1.00
-2
0
2
Change in mean BMI (kg/m²)
Central and eastern Europe
Central Asia, Middle East and north Africa
East and southeast Asia
High-income Asia Pacific
High-income western
Latin America and Caribbean
Oceania
South Asia
Sub-Saharan Africa
PP >0.99
0.975< PP ≤0.99
0.95< PP ≤0.975
0.75< PP ≤0.95
PP ≤0.75

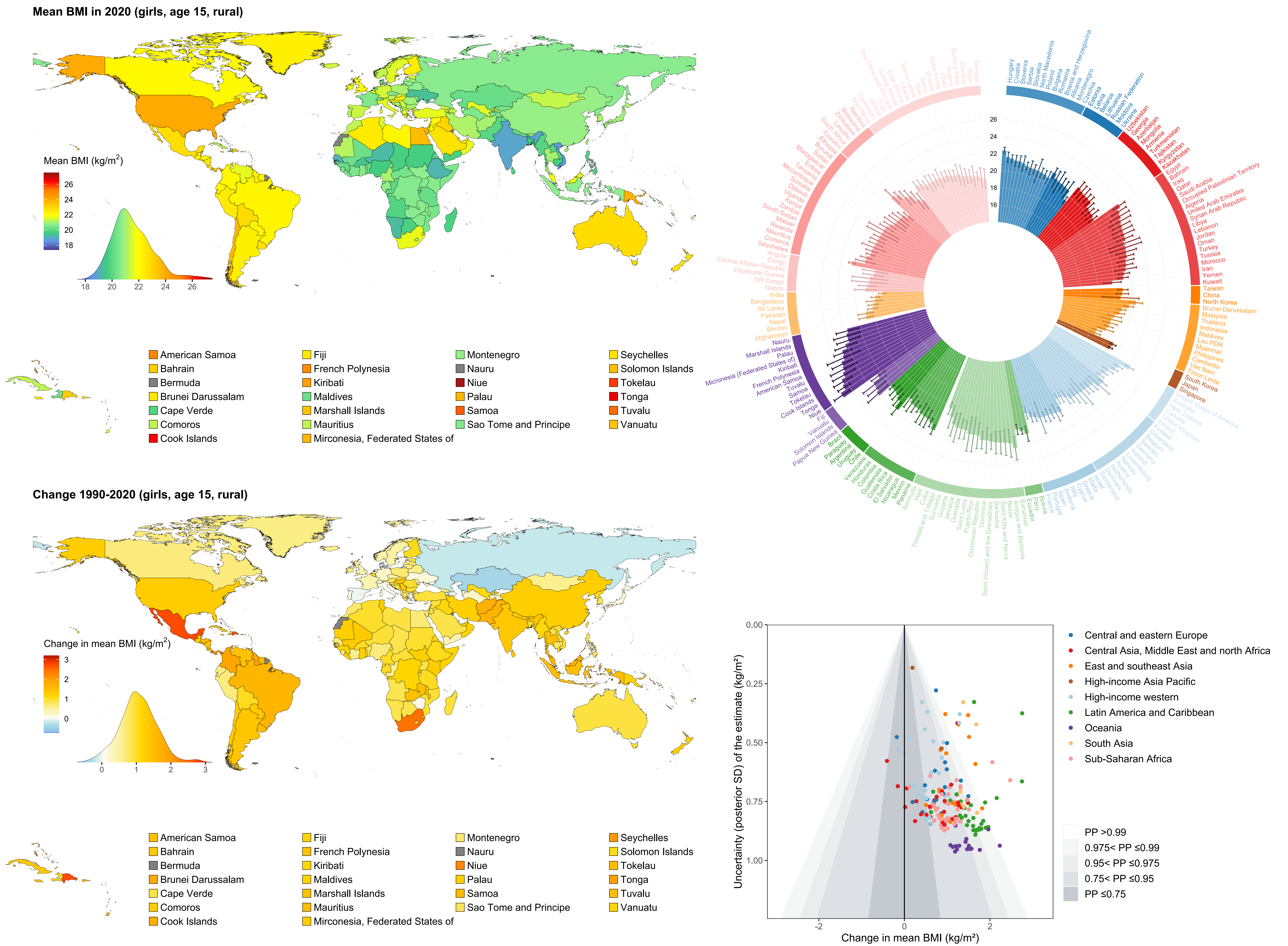
Mean BMI in 2020 (girls, age 15, rural)
Mean BMI (kg/m^2)
American Samoa
Bahrain
Bermuda
Brunei Darussalam
Cape Verde
Comoros
Cook Islands
Fiji
French Polynesia
Kiribati
Maldives
Marshall Islands
Mauritius
Mirconesia, Federated States of
Montenegro
Nauru
Niue
Palau
Samoa
Sao Tome and Principe
Seychelles
Solomon Islands
Tokelau
Tonga
Tuvalu
Vanuatu
Change 1990-2020 (girls, age 15, rural)
Change in mean BMI (kg/m^2)
Uncertainty (posterior SD) of the estimate (kg/m²)
Change in mean BMI (kg/m²)
Central and eastern Europe
Central Asia, Middle East and north Africa
East and southeast Asia
High-income Asia Pacific
High-income western
Latin America and Caribbean
Oceania
South Asia
Sub-Saharan Africa
PP >0.99
0.975< PP ≤0.99
0.95< PP ≤0.975
0.75< PP ≤0.95
PP ≤0.75

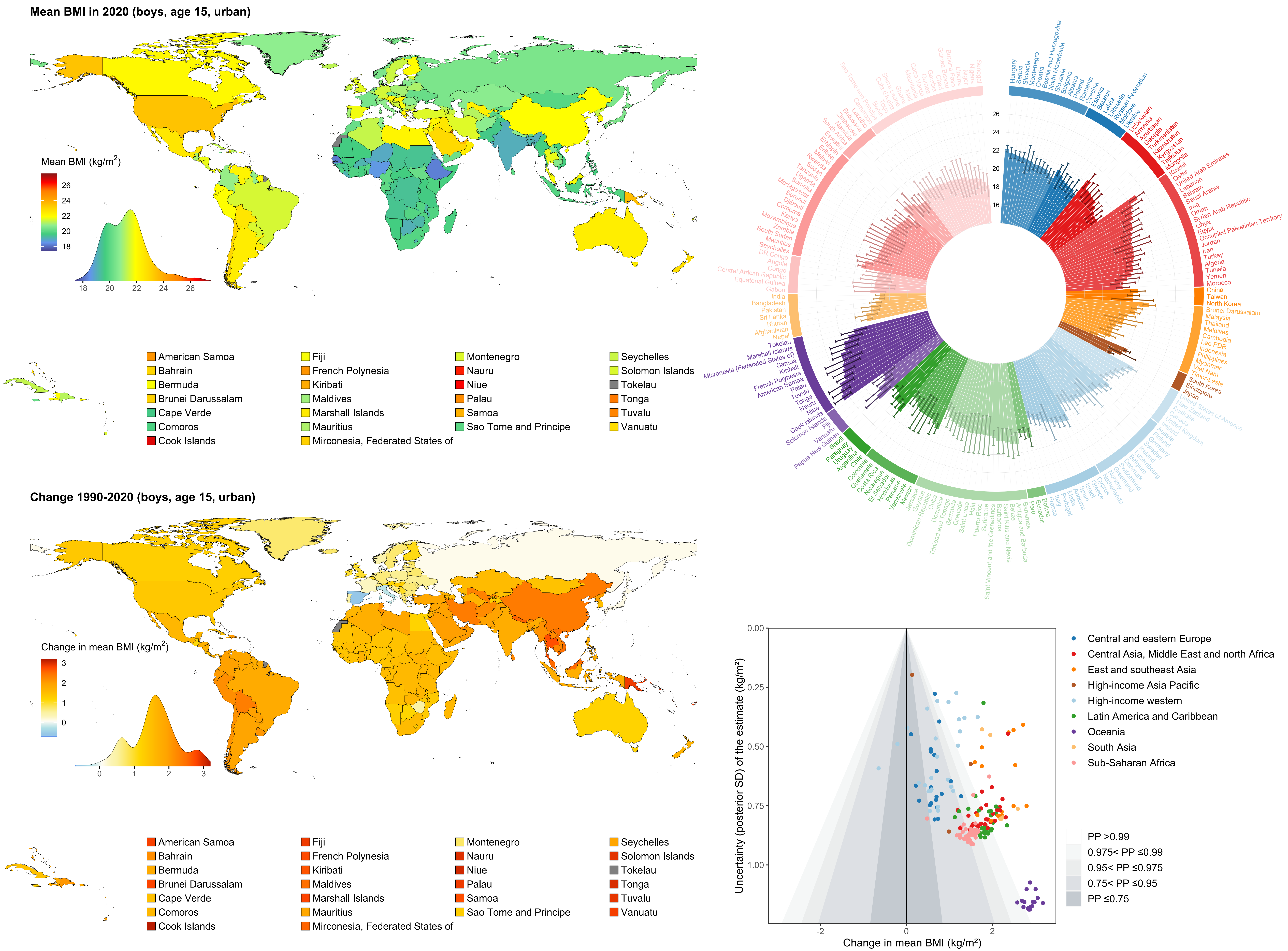

Mean BMI in 2020 (boys, age 15, urban)
Mean BMI (kg/m²)
18
20
22
24
26
American Samoa
Bahrain
Bermuda
Brunei Darussalam
Cape Verde
Comoros
Cook Islands
Fiji
French Polynesia
Kiribati
Maldives
Marshall Islands
Mauritius
Mirconesia, Federated States of
Montenegro
Nauru
Niue
Palau
Samoa
Sao Tome and Principe
Seychelles
Solomon Islands
Tokelau
Tonga
Tuvalu
Vanuatu
Change 1990-2020 (boys, age 15, urban)
Change in mean BMI (kg/m²)
0
1
2
3
American Samoa
Bahrain
Bermuda
Brunei Darussalam
Cape Verde
Comoros
Cook Islands
Fiji
French Polynesia
Kiribati
Maldives
Marshall Islands
Mauritius
Mirconesia, Federated States of
Montenegro
Nauru
Niue
Palau
Samoa
Sao Tome and Principe
Seychelles
Solomon Islands
Tokelau
Tonga
Tuvalu
Vanuatu
16
18
20
22
24
26
Uncertainty (posterior SD) of the estimate (kg/m²)
0.00
0.25
0.50
0.75
1.00
-2
0
2
Change in mean BMI (kg/m²)
Central and eastern Europe
Central Asia, Middle East and north Africa
East and southeast Asia
High-income Asia Pacific
High-income western
Latin America and Caribbean
Oceania
South Asia
Sub-Saharan Africa
PP >0.99
0.975< PP ≤0.99
0.95< PP ≤0.975
0.75< PP ≤0.95
PP ≤0.75

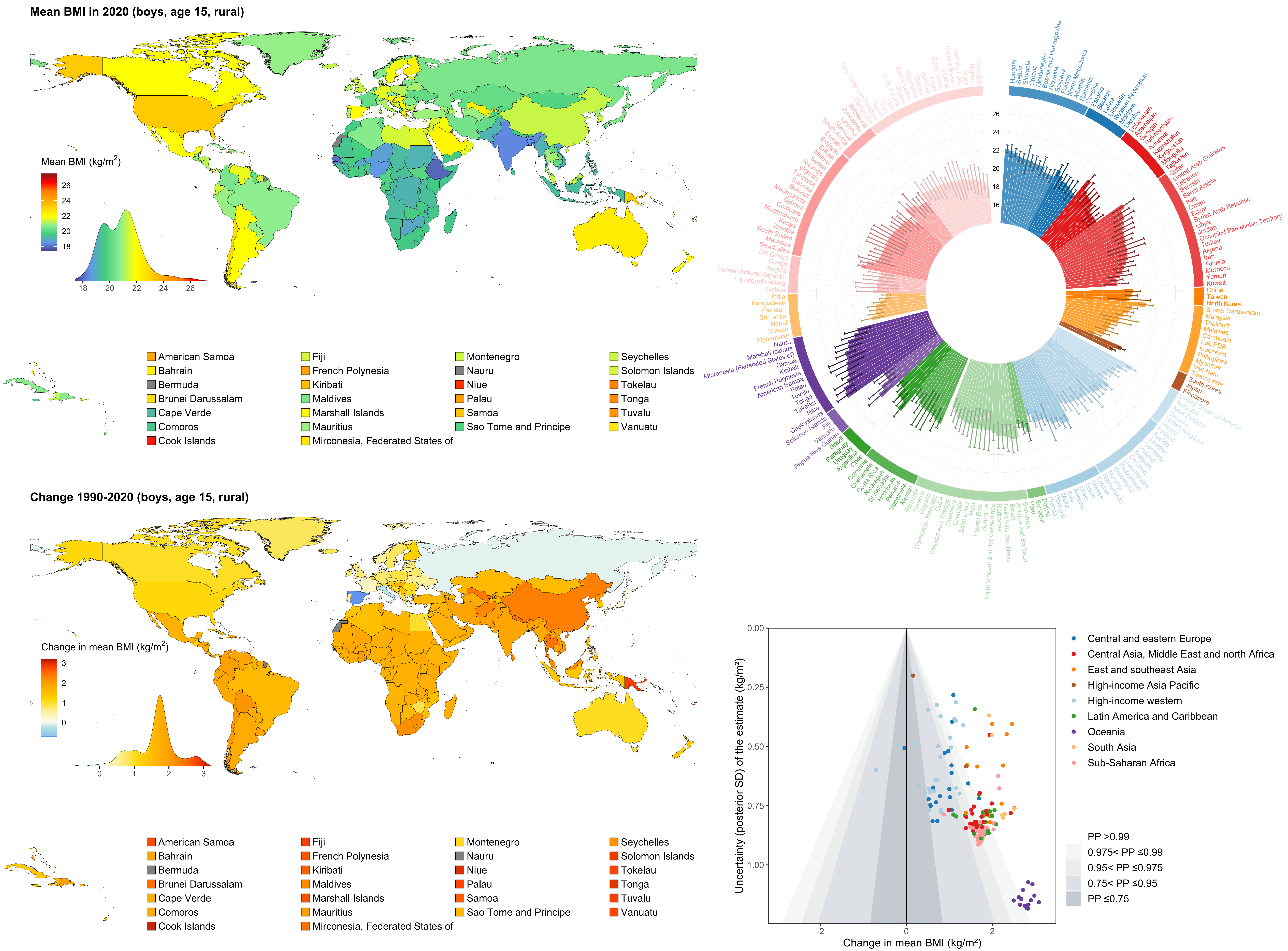

Mean BMI in 2020 (boys, age 15, rural)
Mean BMI (kg/m²)
26
24
22
20
18
18 20 22 24 26
American Samoa
Bahrain
Bermuda
Brunei Darussalam
Cape Verde
Comoros
Cook Islands
Fiji
French Polynesia
Kiribati
Maldives
Marshall Islands
Mauritius
Mirconesia, Federated States of
Montenegro
Nauru
Niue
Palau
Samoa
Sao Tome and Principe
Seychelles
Solomon Islands
Tokelau
Tonga
Tuvalu
Vanuatu
Change 1990-2020 (boys, age 15, rural)
Change in mean BMI (kg/m²)
3
2
1
0
0 1 2 3
American Samoa
Bahrain
Bermuda
Brunei Darussalam
Cape Verde
Comoros
Cook Islands
Fiji
French Polynesia
Kiribati
Maldives
Marshall Islands
Mauritius
Mirconesia, Federated States of
Montenegro
Nauru
Niue
Palau
Samoa
Sao Tome and Principe
Seychelles
Solomon Islands
Tokelau
Tonga
Tuvalu
Vanuatu
26
24
22
20
18
16
Uncertainty (posterior SD) of the estimate (kg/m²)
0.00
0.25
0.50
0.75
1.00
-2
0
2
Change in mean BMI (kg/m²)
Central and eastern Europe
Central Asia, Middle East and north Africa
East and southeast Asia
High-income Asia Pacific
High-income western
Latin America and Caribbean
Oceania
South Asia
Sub-Saharan Africa
PP >0.99
0.975< PP ≤0.99
0.95< PP ≤0.975
0.75< PP ≤0.95
PP ≤0.75

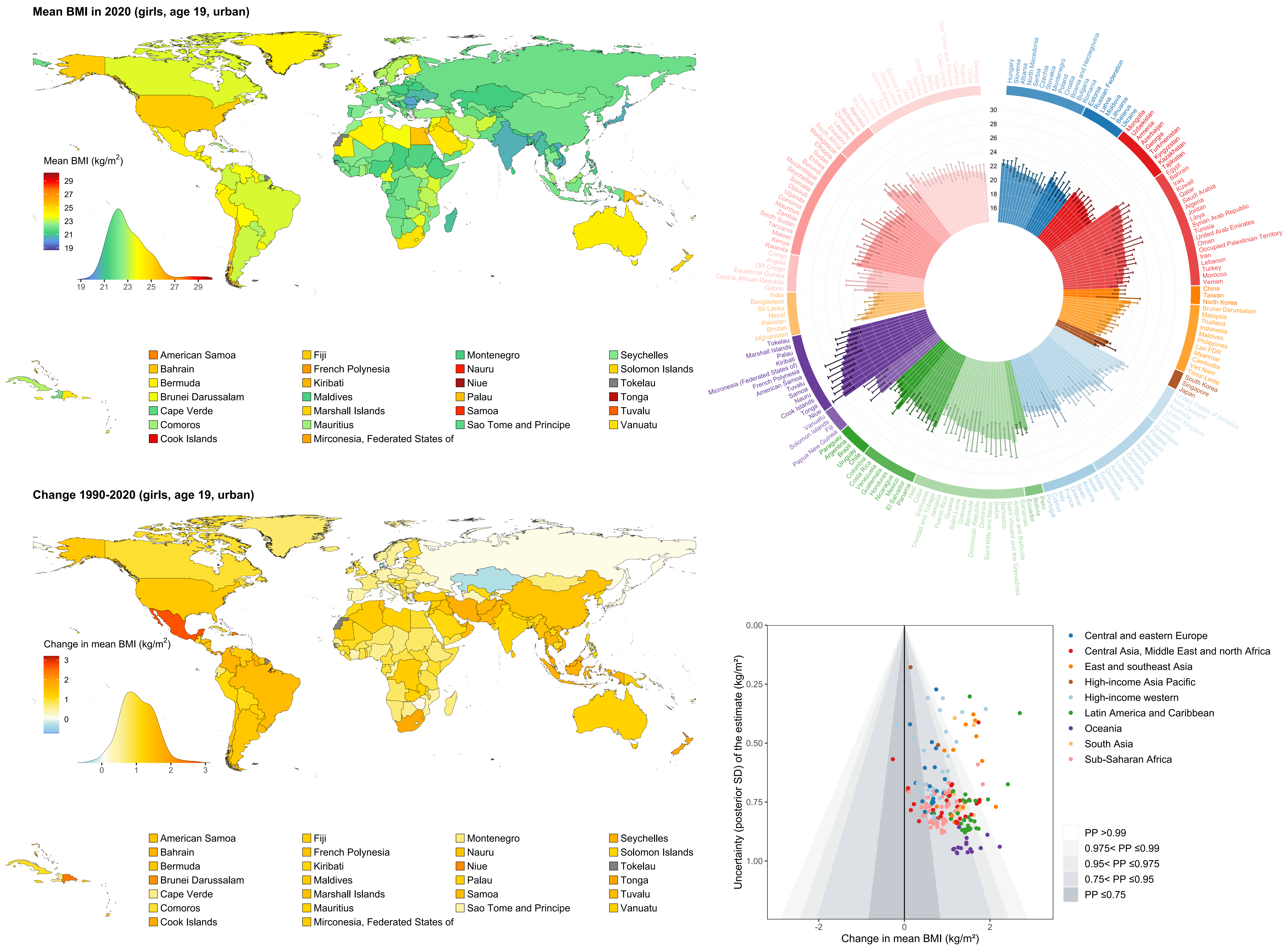
Mean BMI in 2020 (girls, age 19, urban)
Mean BMI (kg/m2)
29
27
25
23
21
19
American Samoa
Bahrain
Bermuda
Brunei Darussalam
Cape Verde
Comoros
Cook Islands
Fiji
French Polynesia
Kiribati
Maldives
Marshall Islands
Mauritius
Mirconesia, Federated States of
Montenegro
Nauru
Niue
Palau
Samoa
Sao Tome and Principe
Seychelles
Solomon Islands
Tokelau
Tonga
Tuvalu
Vanuatu
Change 1990-2020 (girls, age 19, urban)
Change in mean BMI (kg/m2)
3
2
1
0
Uncertainty (posterior SD) of the estimate (kg/m²)
Change in mean BMI (kg/m²)
Central and eastern Europe
Central Asia, Middle East and north Africa
East and southeast Asia
High-income Asia Pacific
High-income western
Latin America and Caribbean
Oceania
South Asia
Sub-Saharan Africa
PP >0.99
0.975< PP ≤0.99
0.95< PP ≤0.975
0.75< PP ≤0.95
PP ≤0.75

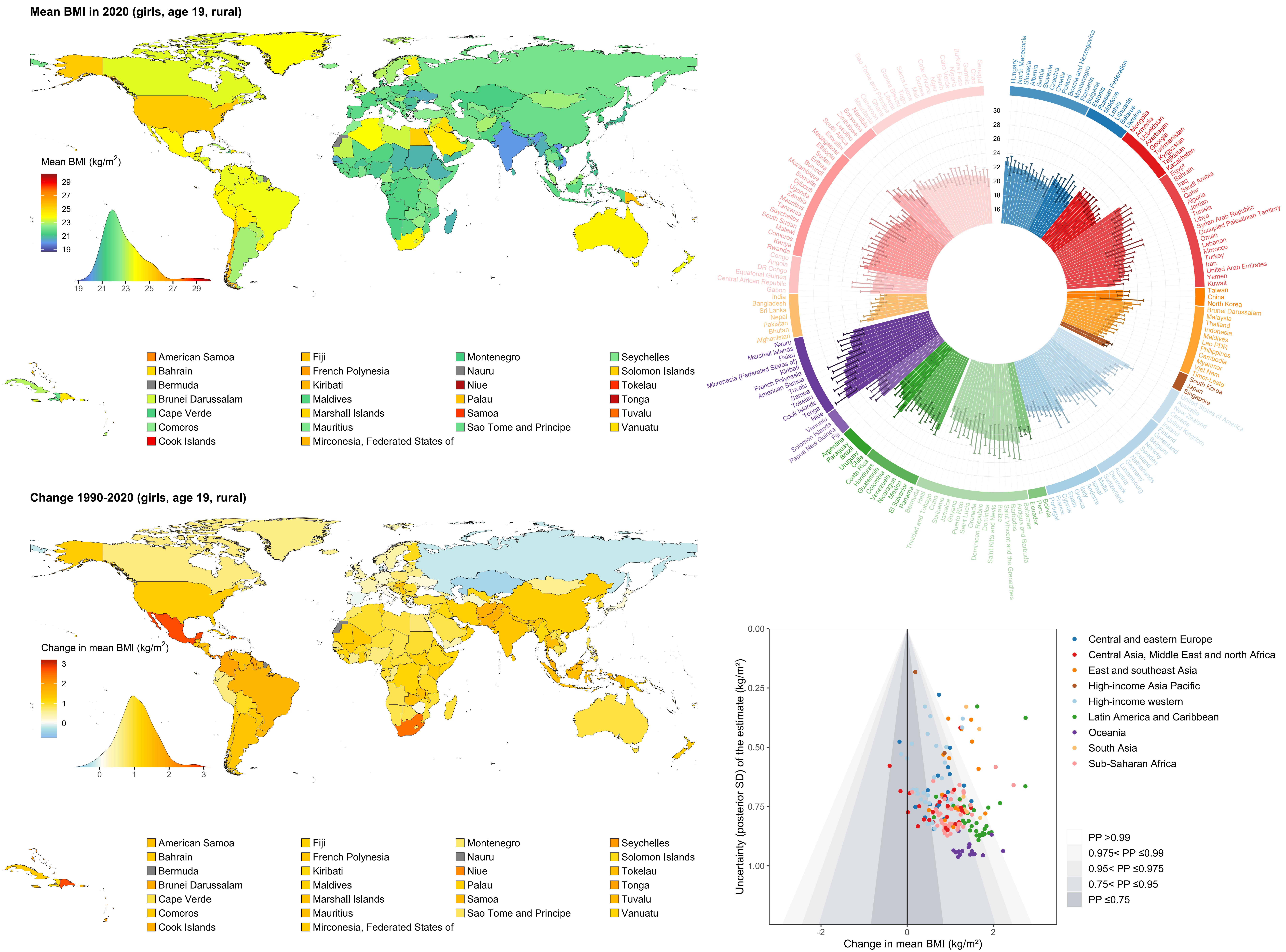
Mean BMI in 2020 (girls, age 19, rural)
Mean BMI (kg/m²)
29
27
25
23
21
19
American Samoa
Bahrain
Bermuda
Brunei Darussalam
Cape Verde
Comoros
Cook Islands
Fiji
French Polynesia
Kiribati
Maldives
Marshall Islands
Mauritius
Mirconesia, Federated States of
Montenegro
Nauru
Niue
Palau
Samoa
Sao Tome and Principe
Seychelles
Solomon Islands
Tokelau
Tonga
Tuvalu
Vanuatu
Change 1990-2020 (girls, age 19, rural)
Change in mean BMI (kg/m²)
3
2
1
0
American Samoa
Bahrain
Bermuda
Brunei Darussalam
Cape Verde
Comoros
Cook Islands
Fiji
French Polynesia
Kiribati
Maldives
Marshall Islands
Mauritius
Mirconesia, Federated States of
Montenegro
Nauru
Niue
Palau
Samoa
Sao Tome and Principe
Seychelles
Solomon Islands
Tokelau
Tonga
Tuvalu
Vanuatu
Uncertainty (posterior SD) of the estimate (kg/m²)
Change in mean BMI (kg/m²)
Central and eastern Europe
Central Asia, Middle East and north Africa
East and southeast Asia
High-income Asia Pacific
High-income western
Latin America and Caribbean
Oceania
South Asia
Sub-Saharan Africa
PP >0.99
0.975< PP ≤0.99
0.95< PP ≤0.975
0.75< PP ≤0.95
PP ≤0.75

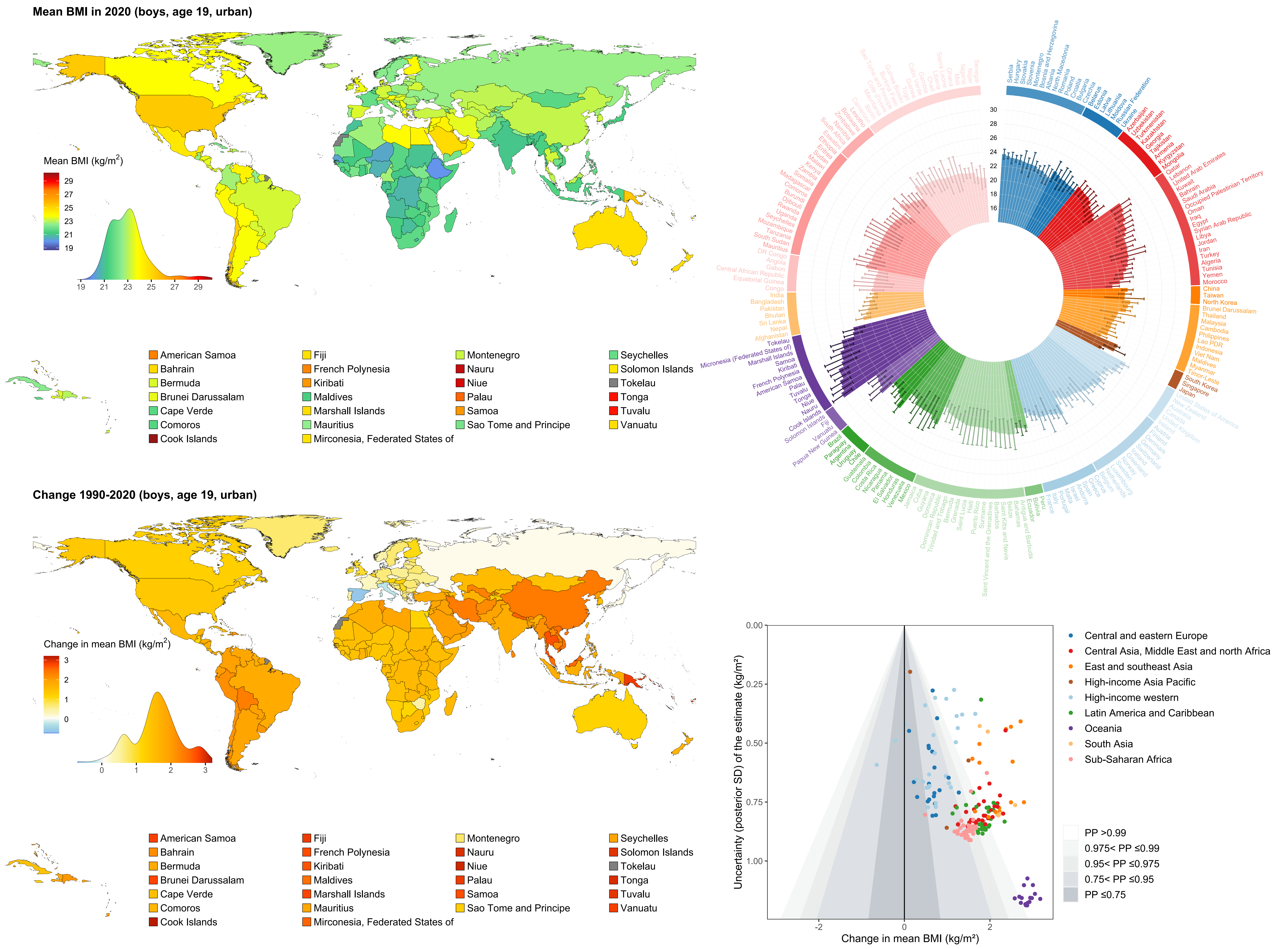

Mean BMI in 2020 (boys, age 19, urban)
Mean BMI (kg/m²)
American Samoa
Bahrain
Bermuda
Brunei Darussalam
Cape Verde
Comoros
Cook Islands
Fiji
French Polynesia
Kiribati
Maldives
Marshall Islands
Mauritius
Mirconesia, Federated States of
Montenegro
Nauru
Niue
Palau
Samoa
Sao Tome and Principe
Seychelles
Solomon Islands
Tokelau
Tonga
Tuvalu
Vanuatu
Change 1990-2020 (boys, age 19, urban)
Change in mean BMI (kg/m²)
Uncertainty (posterior SD) of the estimate (kg/m²)
Change in mean BMI (kg/m²)
Central and eastern Europe
Central Asia, Middle East and north Africa
East and southeast Asia
High-income Asia Pacific
High-income western
Latin America and Caribbean
Oceania
South Asia
Sub-Saharan Africa
PP >0.99
0.975< PP ≤0.99
0.95< PP ≤0.975
0.75< PP ≤0.95
PP ≤0.75

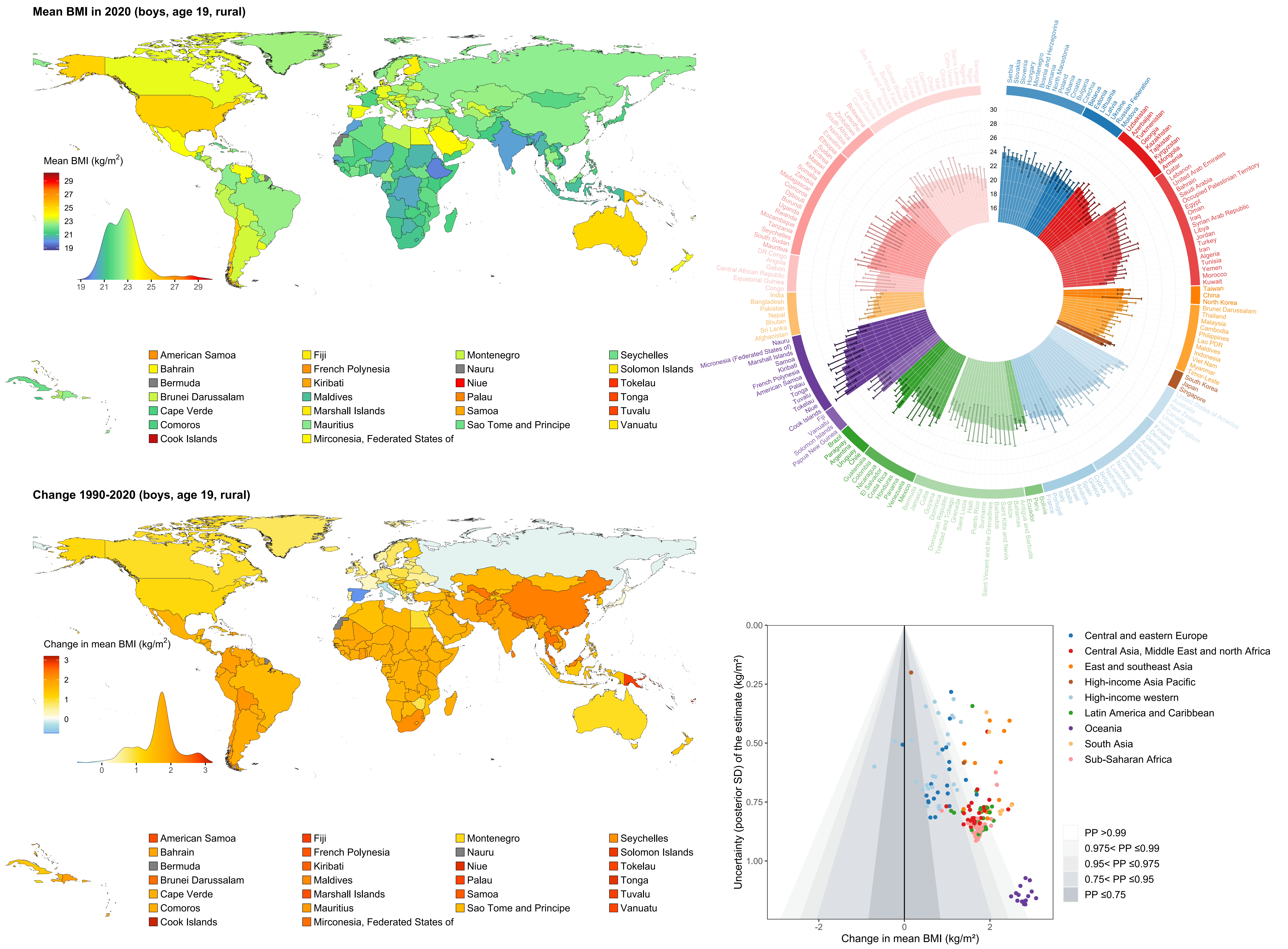
Mean BMI in 2020 (boys, age 19, rural)
Mean BMI (kg/m²)
29
27
25
23
21
19
American Samoa
Bahrain
Bermuda
Brunei Darussalam
Cape Verde
Comoros
Cook Islands
Fiji
French Polynesia
Kiribati
Maldives
Marshall Islands
Mauritius
Mirconesia, Federated States of
Montenegro
Nauru
Niue
Palau
Samoa
Sao Tome and Principe
Seychelles
Solomon Islands
Tokelau
Tonga
Tuvalu
Vanuatu
Change 1990-2020 (boys, age 19, rural)
Change in mean BMI (kg/m²)
3
2
1
0
Uncertainty (posterior SD) of the estimate (kg/m²)
Change in mean BMI (kg/m²)
Central and eastern Europe
Central Asia, Middle East and north Africa
East and southeast Asia
High-income Asia Pacific
High-income western
Latin America and Caribbean
Oceania
South Asia
Sub-Saharan Africa
PP >0.99
0.975< PP ≤0.99
0.95< PP ≤0.975
0.75< PP ≤0.95
PP ≤0.75

Supplementary Figure 7. Urban-rural height difference in 2020 and change from 1990 to 2020, by age.

The top map in each panel shows the urban-rural difference in mean height in 2020 for girls and boys at ages 5, 10, 15 and 19 years. A positive number shows higher urban mean height and a negative number shows higher rural mean height. The bottom map in each panel shows the change from 1990 to 2020. The density plot below each map shows the distribution of estimates across countries. The top right graph in each panel shows the urban-rural difference in age-standardised mean height in relation to the uncertainty of the difference measured by posterior standard deviation. The bottom right graph in each panel shows the change from 1990 to 2020 in urban-rural difference in mean height in relation to the uncertainty of the change measured by posterior standard deviation. Each point shows one country. Shaded areas approximately show the posterior probability (PP) of a true difference (top right panel) and a true increase or decrease in difference (bottom right panel).

We did not estimate the difference between rural and urban height for areas classified as entirely urban (Bermuda, Kuwait, Nauru and Singapore) or entirely rural (Tokelau), as indicated by grey colour.

Urban-rural difference in 2020 (girls, age 5)

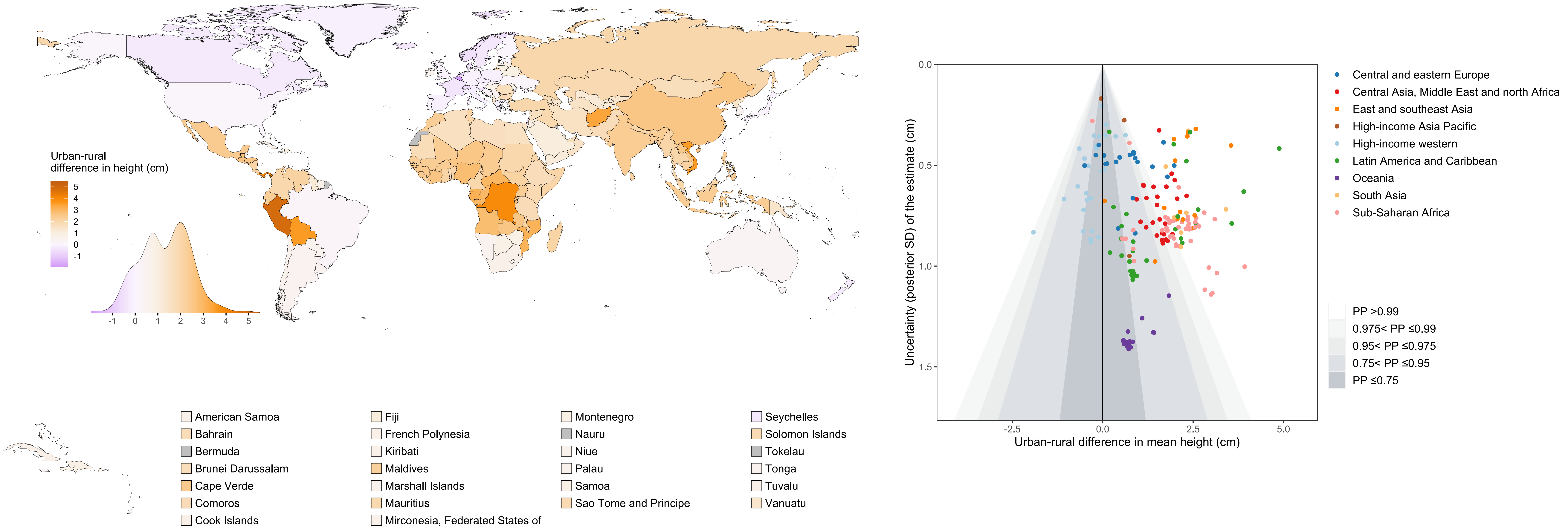

Change 1990-2020 (girls, age 5)

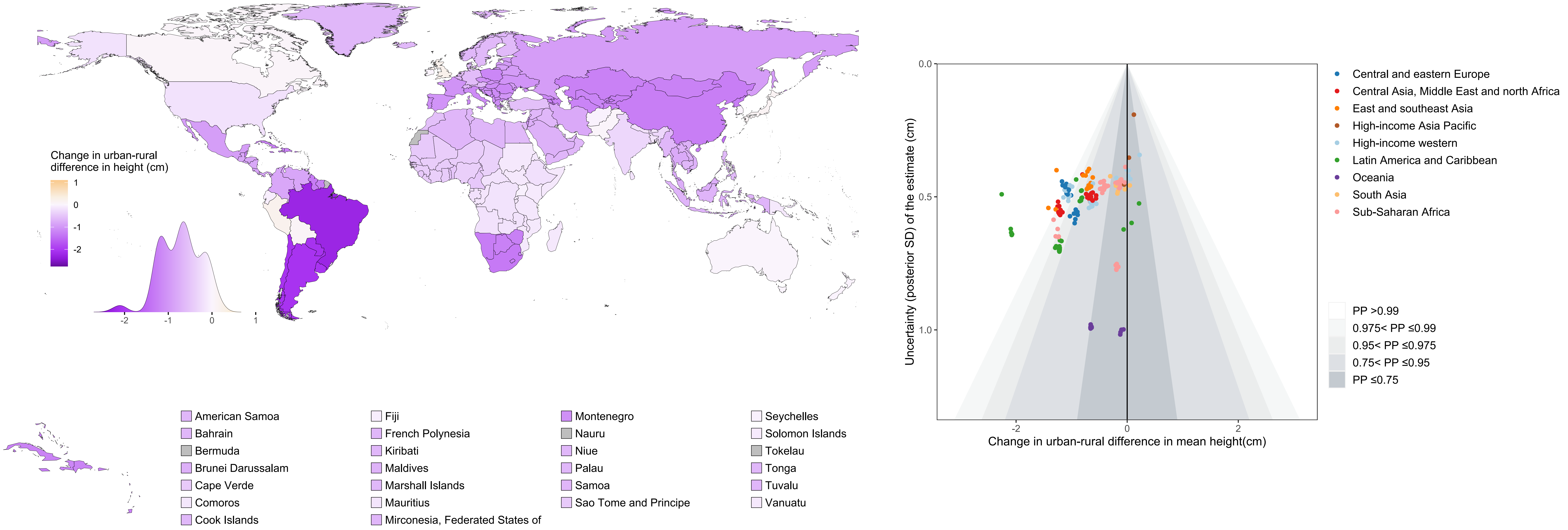

Urban-rural difference in 2020 (boys, age 5)

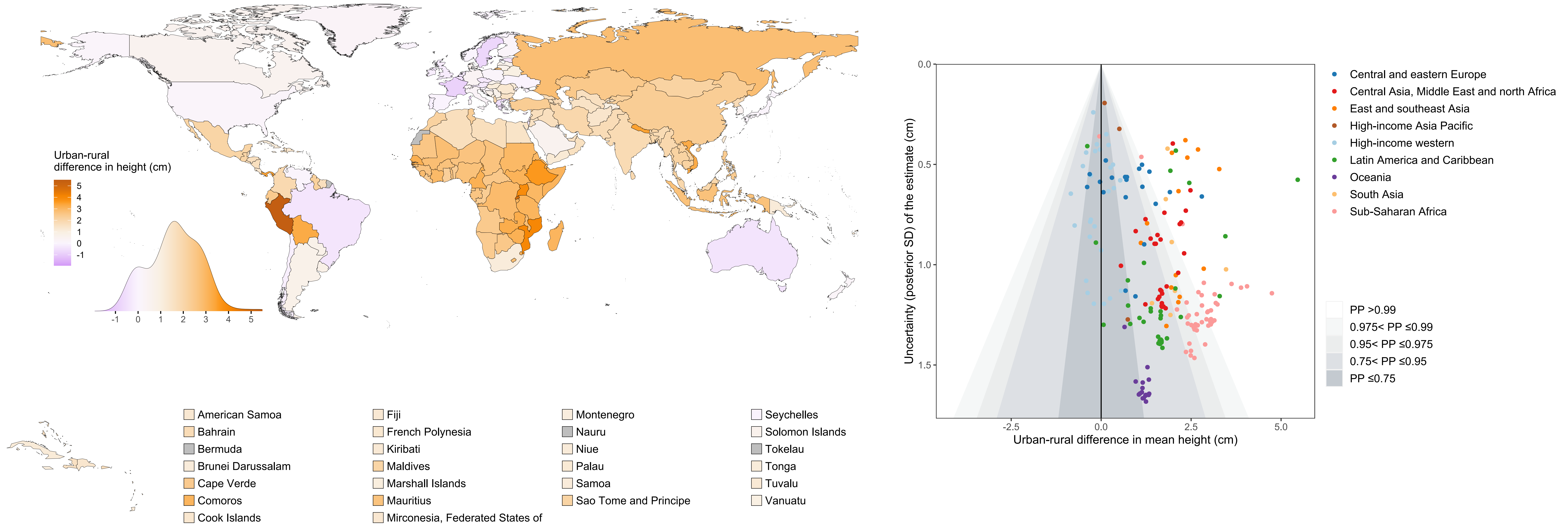

Change 1990-2020 (boys, age 5)

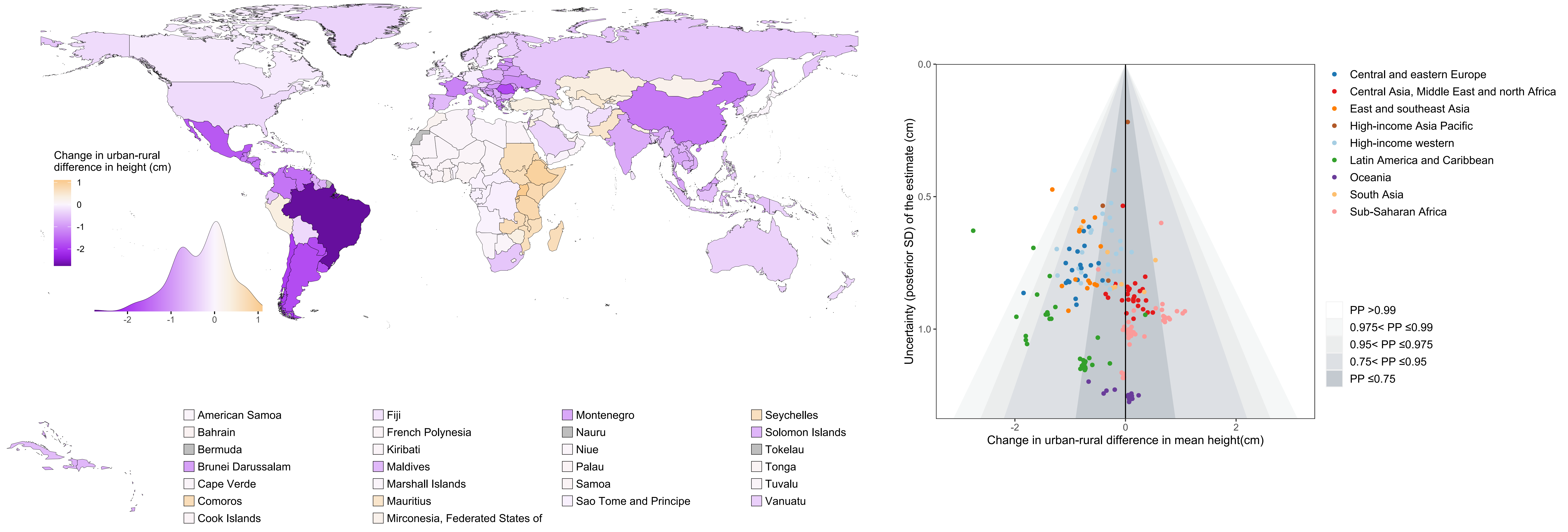

Urban-rural difference in 2020 (girls, age 10)

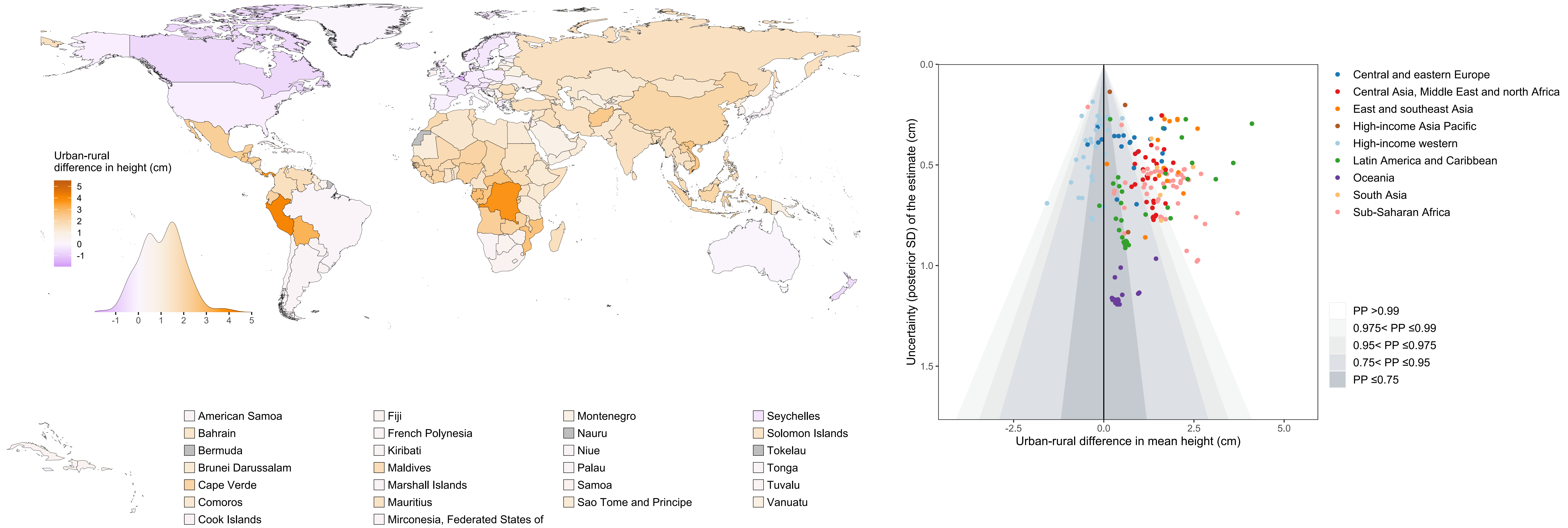

Change 1990-2020 (girls, age 10)

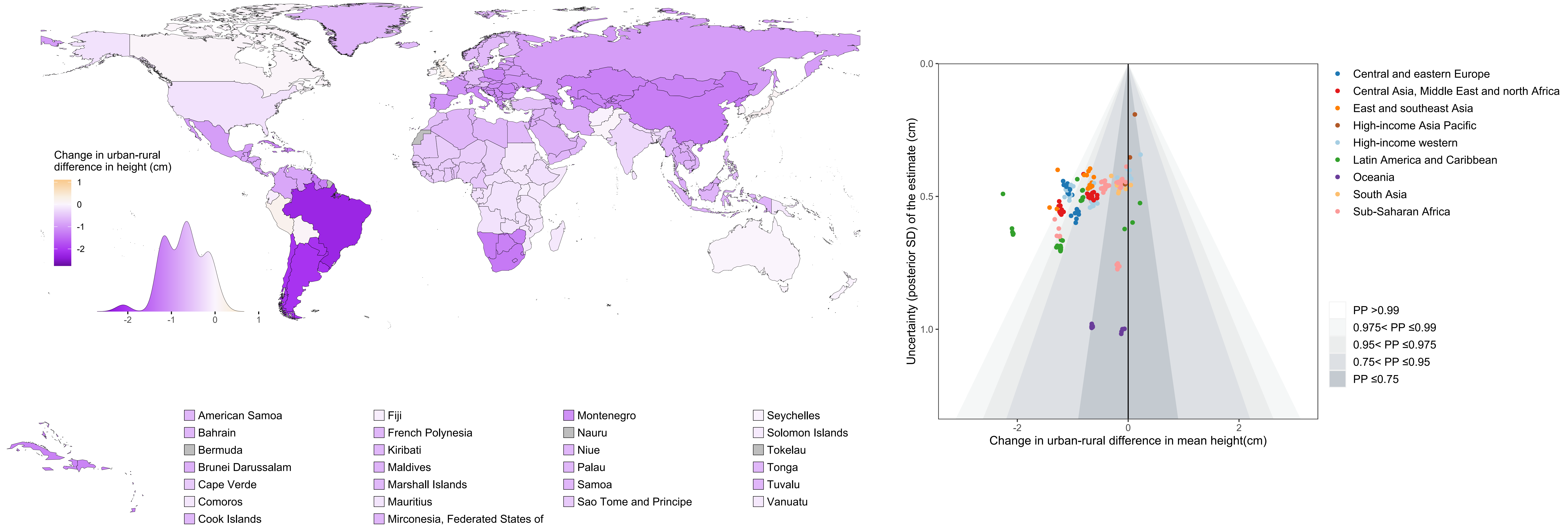

Urban-rural difference in 2020 (boys, age 10)

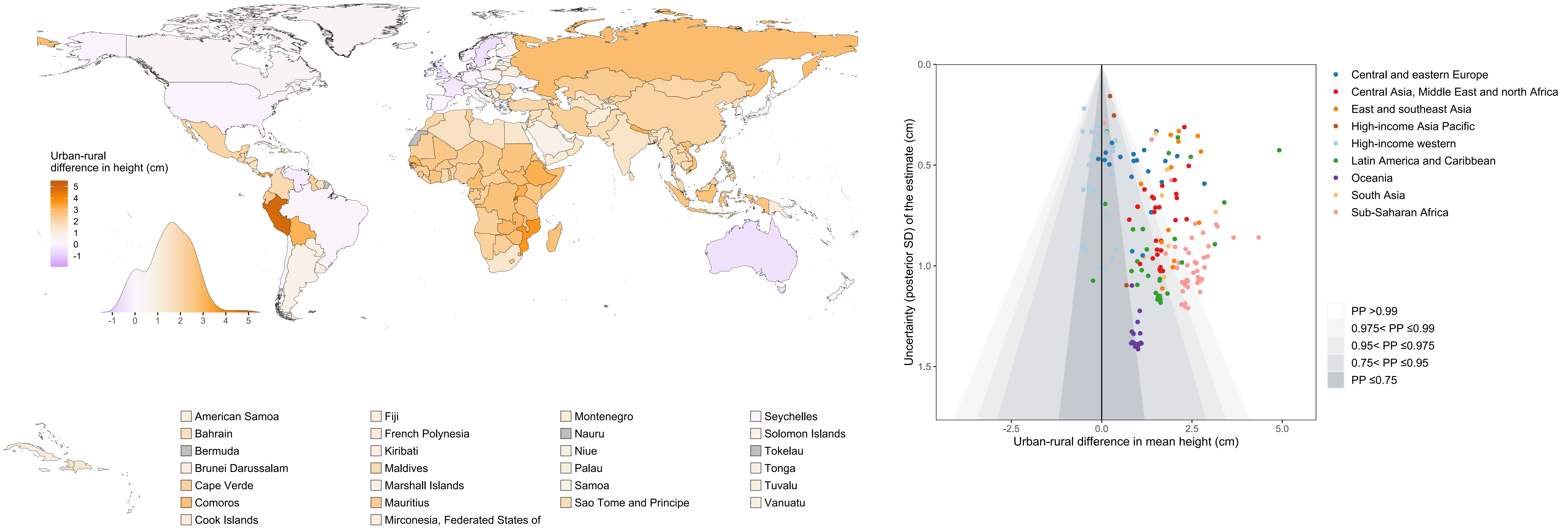

Change 1990-2020 (boys, age 10)

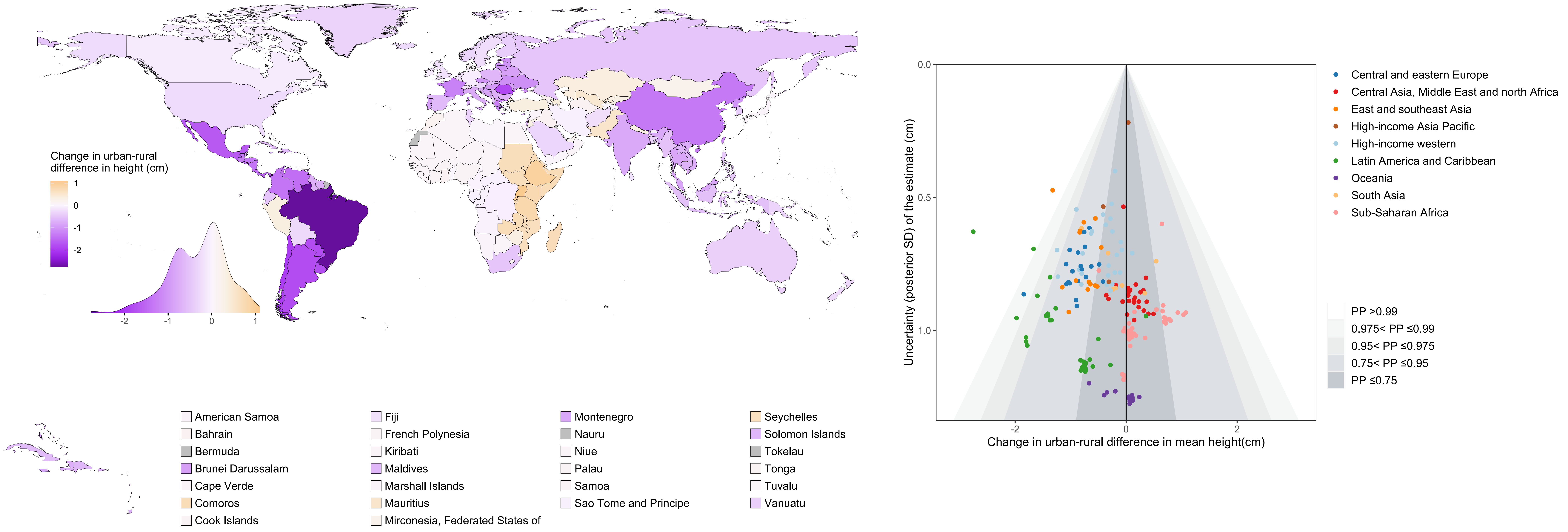

Urban-rural difference in 2020 (girls, age 15)

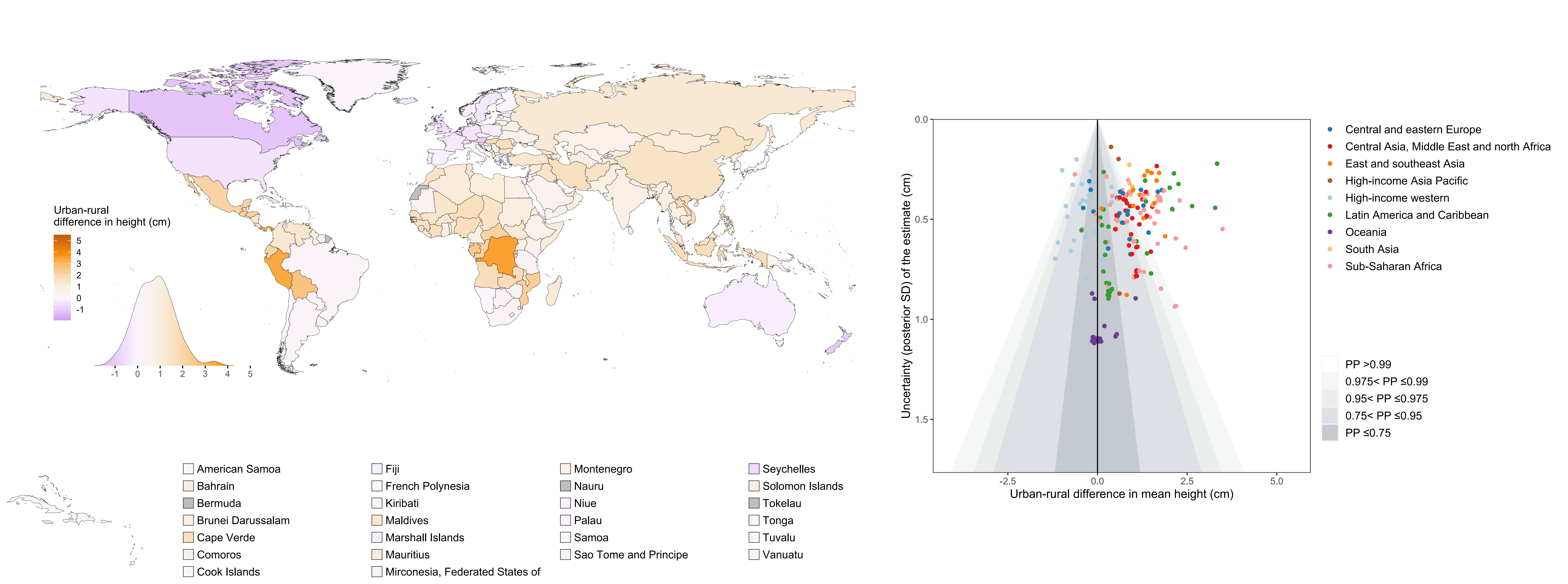

Change 1990-2020 (girls, age 15)

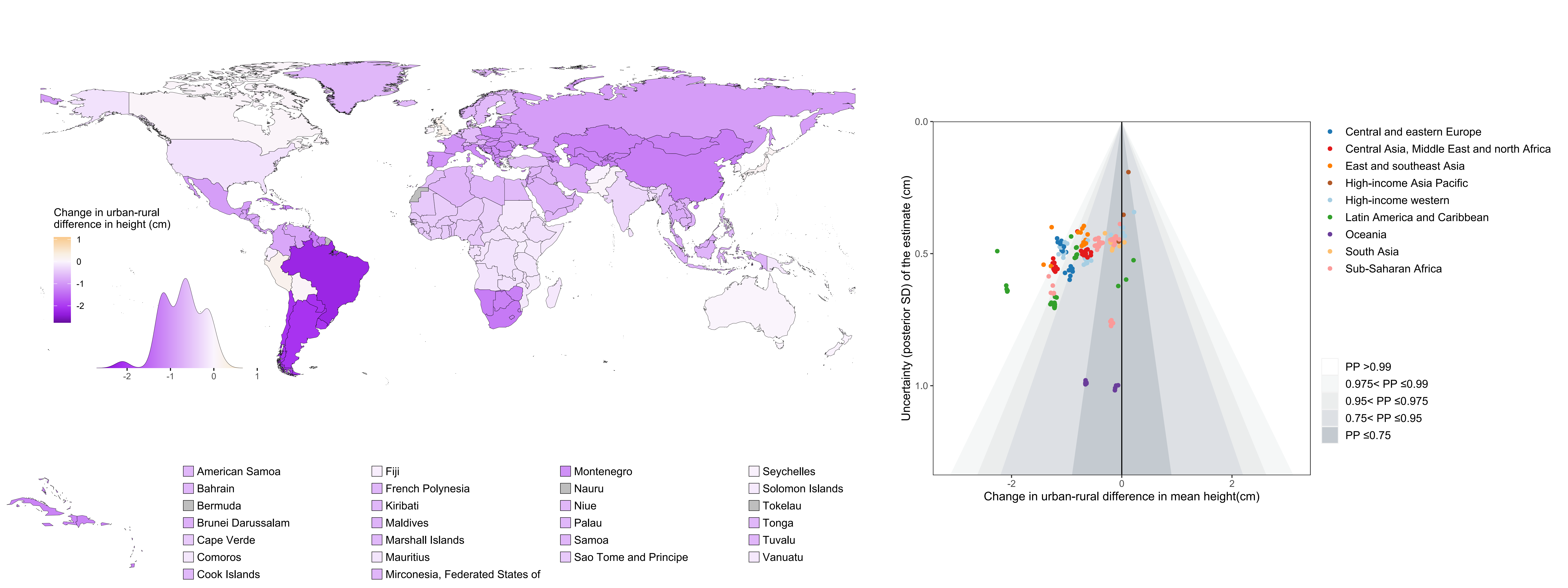

Urban-rural difference in 2020 (boys, age 15)

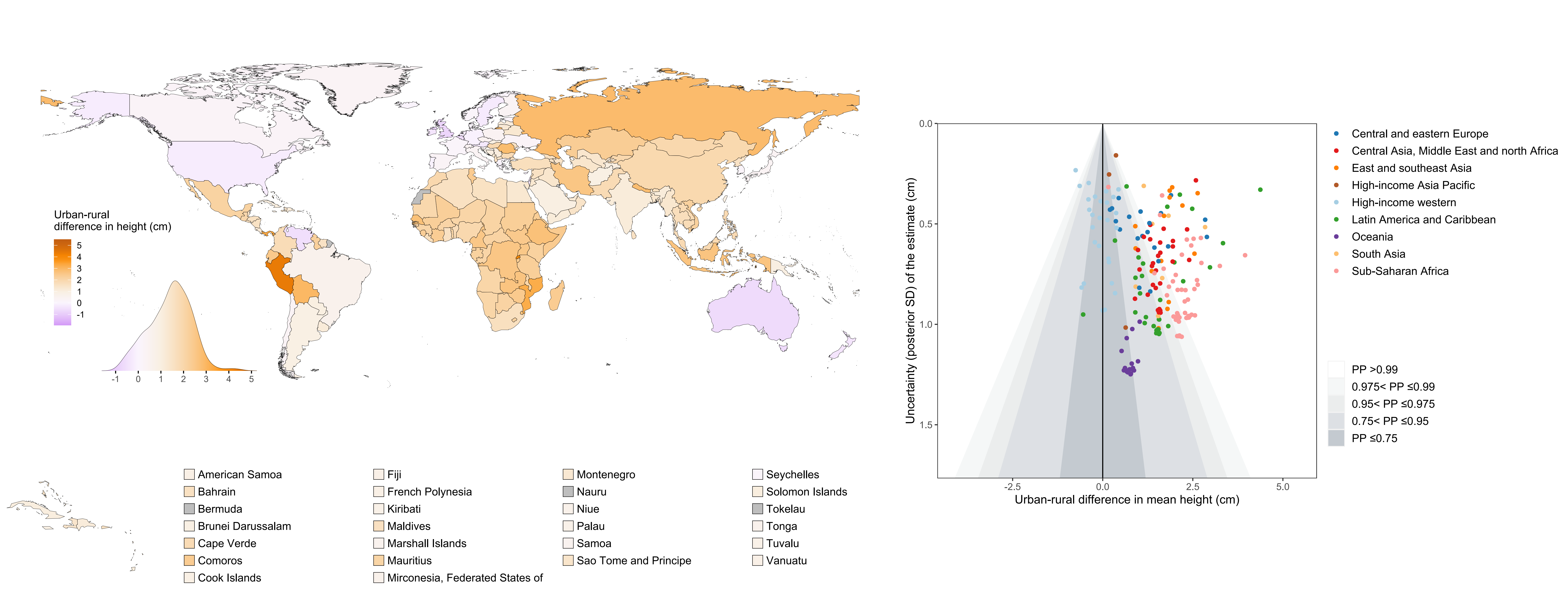

Change 1990-2020 (boys, age 15)

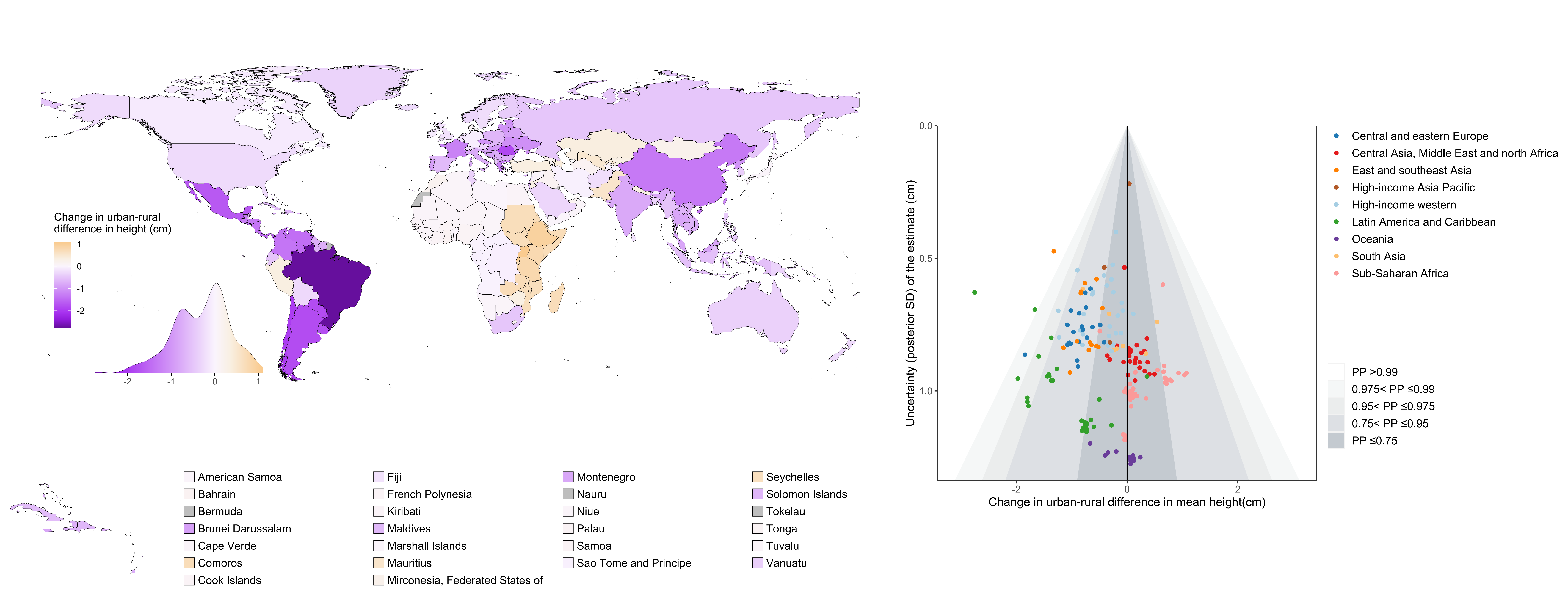

Urban-rural difference in 2020 (girls, age 19)

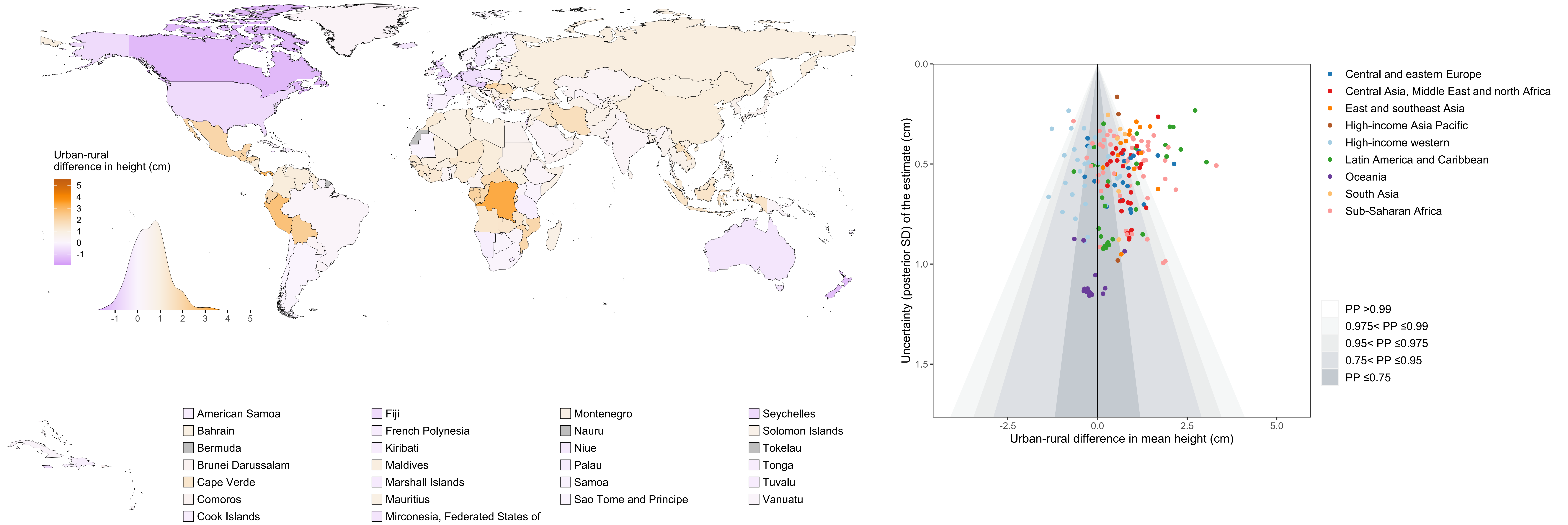

Change 1990-2020 (girls, age 19)

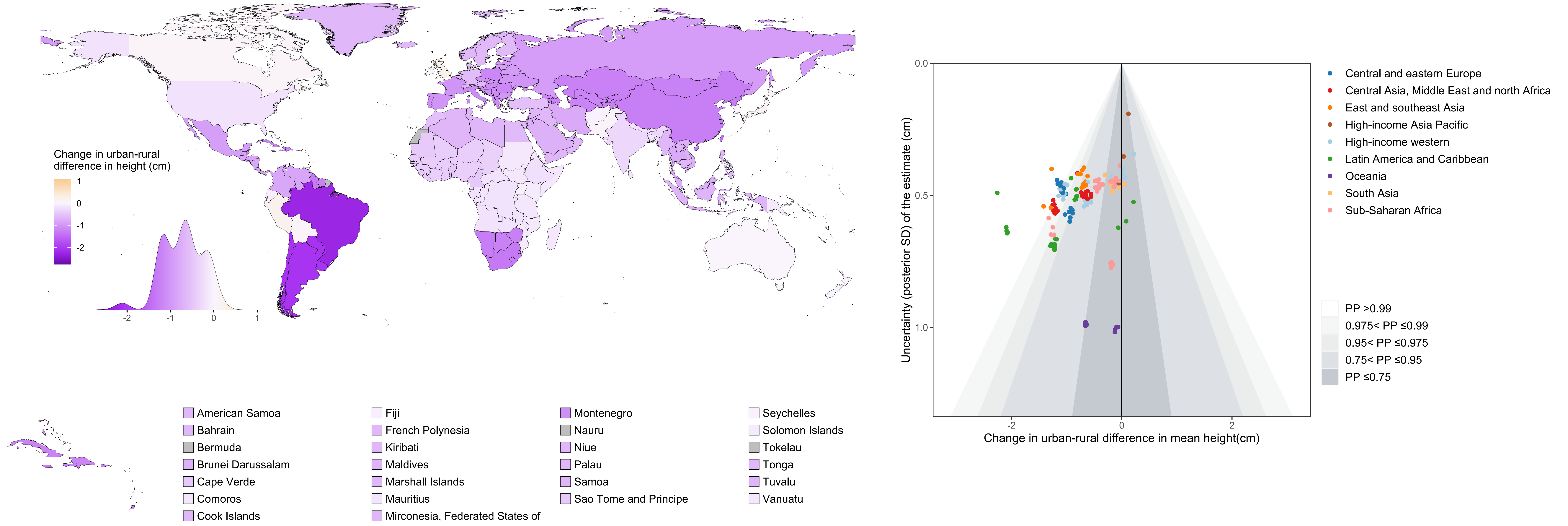

Urban-rural difference in 2020 (boys, age 19)

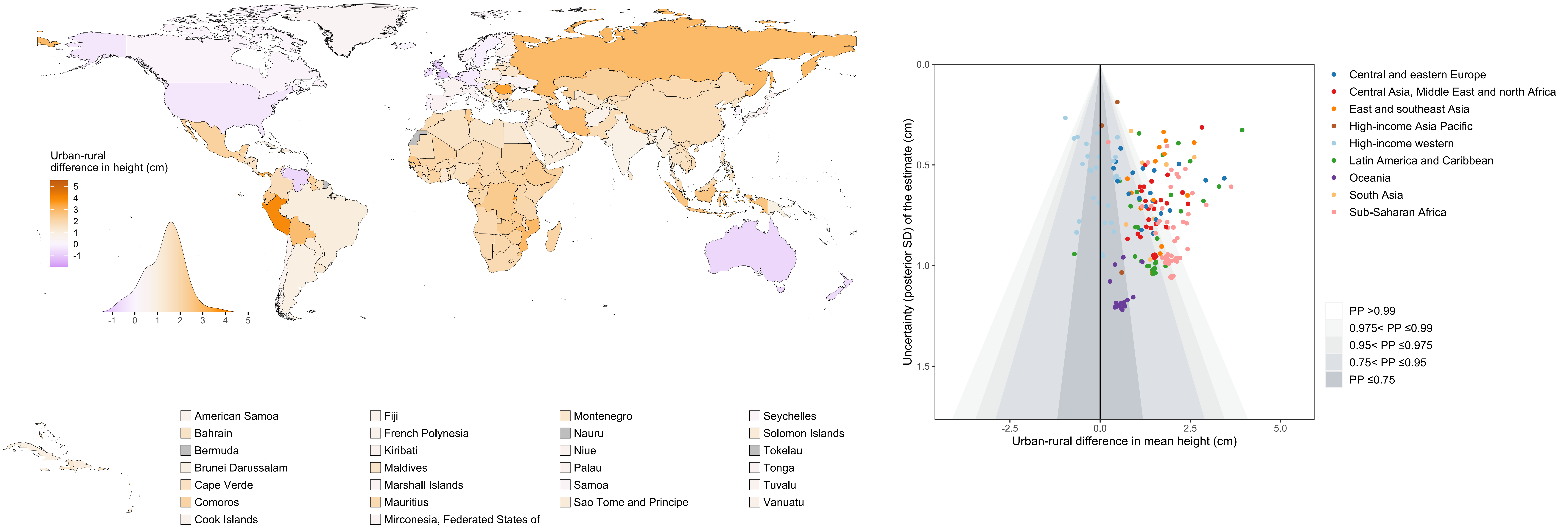

Change 1990-2020 (boys, age 19)

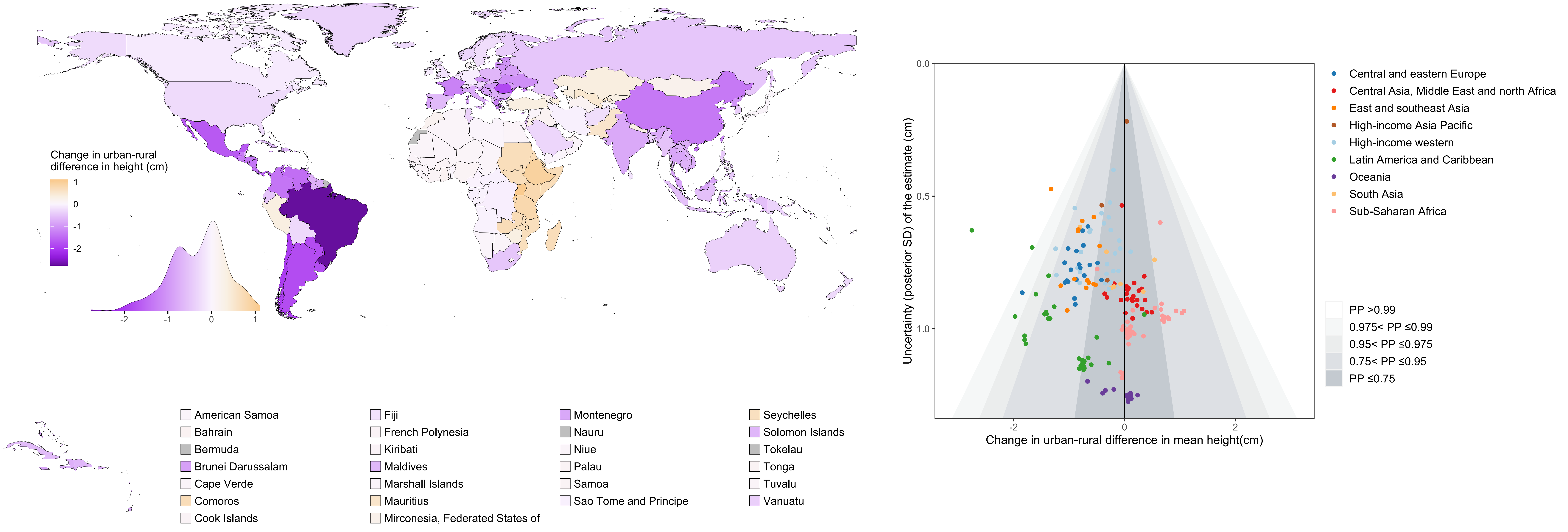

Supplementary Figure 8. Urban-rural body-mass index (BMI) difference in 2020 and change from 1990 to 2020, by age.

The maps show the urban-rural difference in mean BMI in 2020, and its change from 1990 to 2020 for girls and boys results at ages 5, 10, 15 and 19 years. A positive number shows higher urban mean BMI and a negative number show higher rural mean BMI. See Supplementary Figure 7 caption for descriptions of other sections of the figure.

We did not estimate the difference between rural and urban BMI for areas classified as entirely urban (Bermuda, Kuwait, Nauru and Singapore) or entirely rural (Tokelau), as indicated by grey colour.

Urban-rural difference in 2020 (girls, age 5)

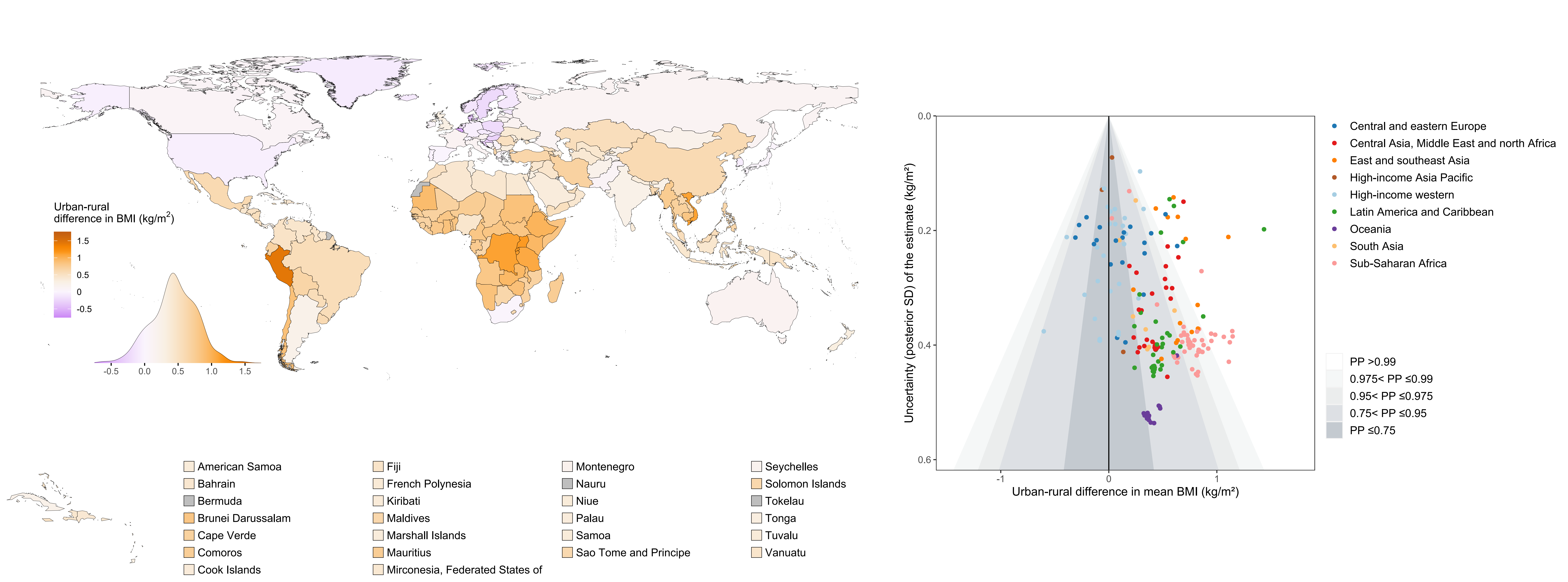

Change 1990-2020 (girls, age 5)

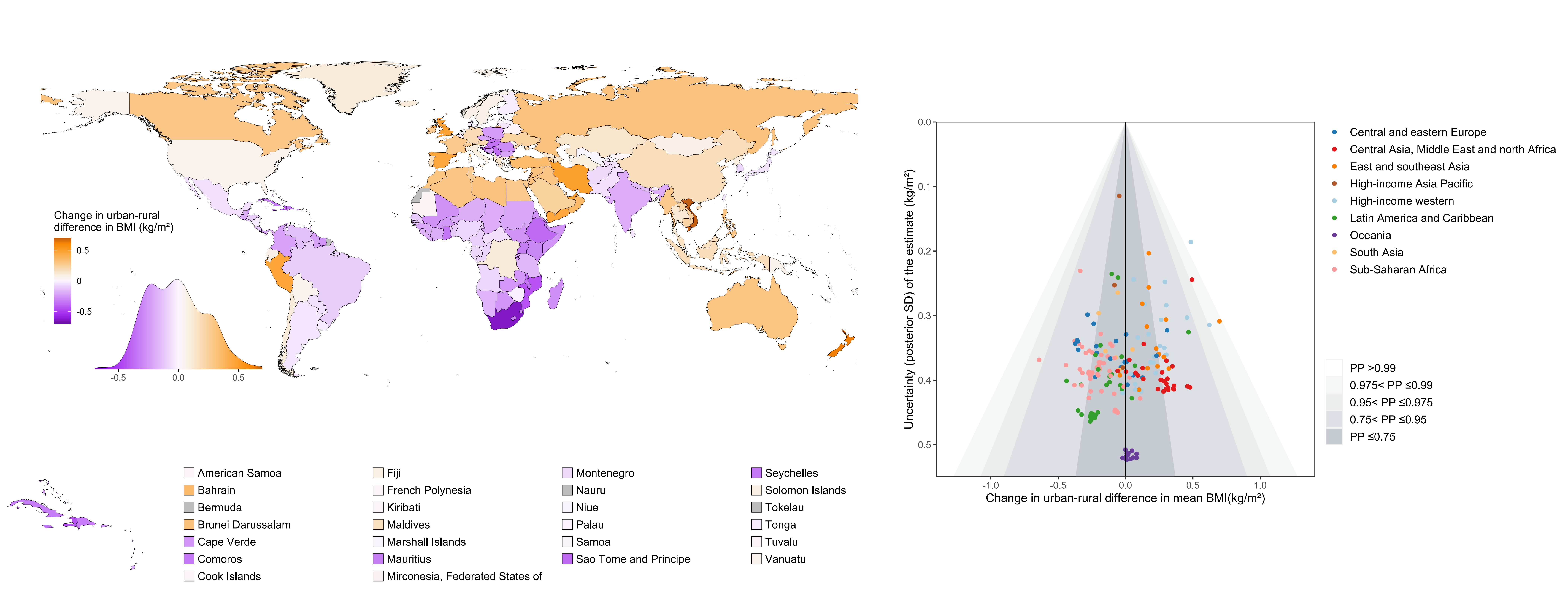

Urban-rural difference in 2020 (boys, age 5)

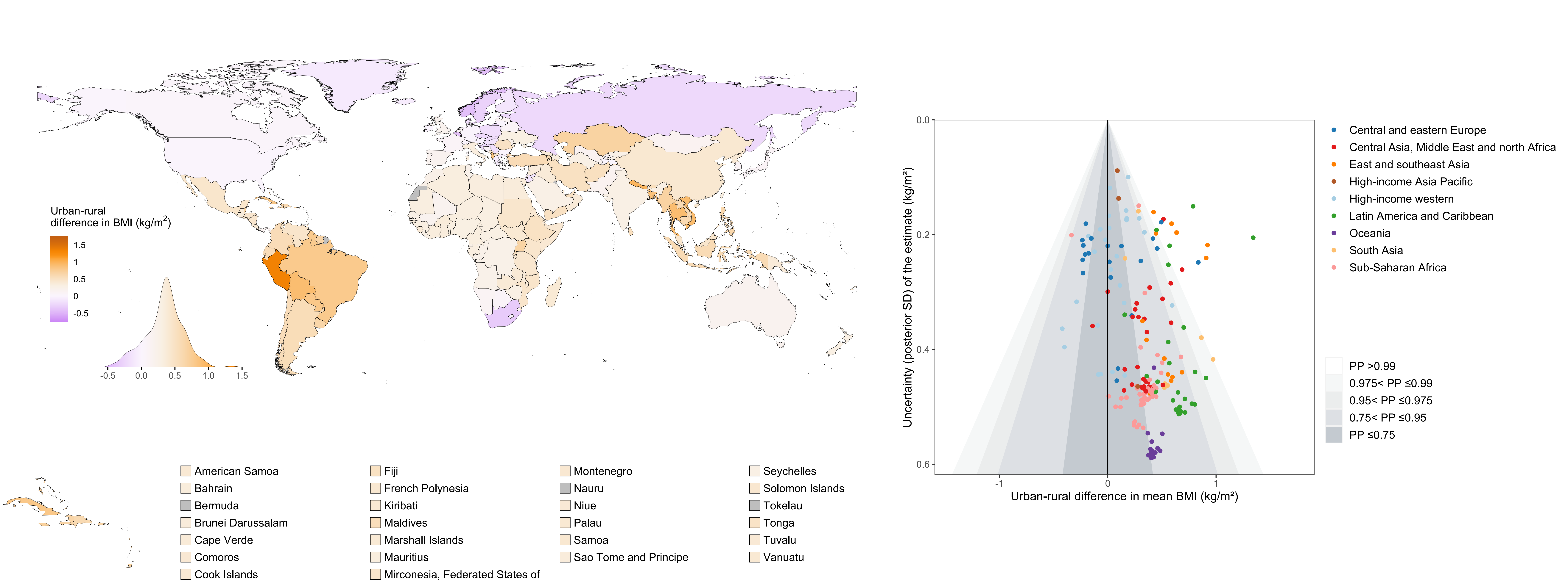

Change 1990-2020 (boys, age 5)

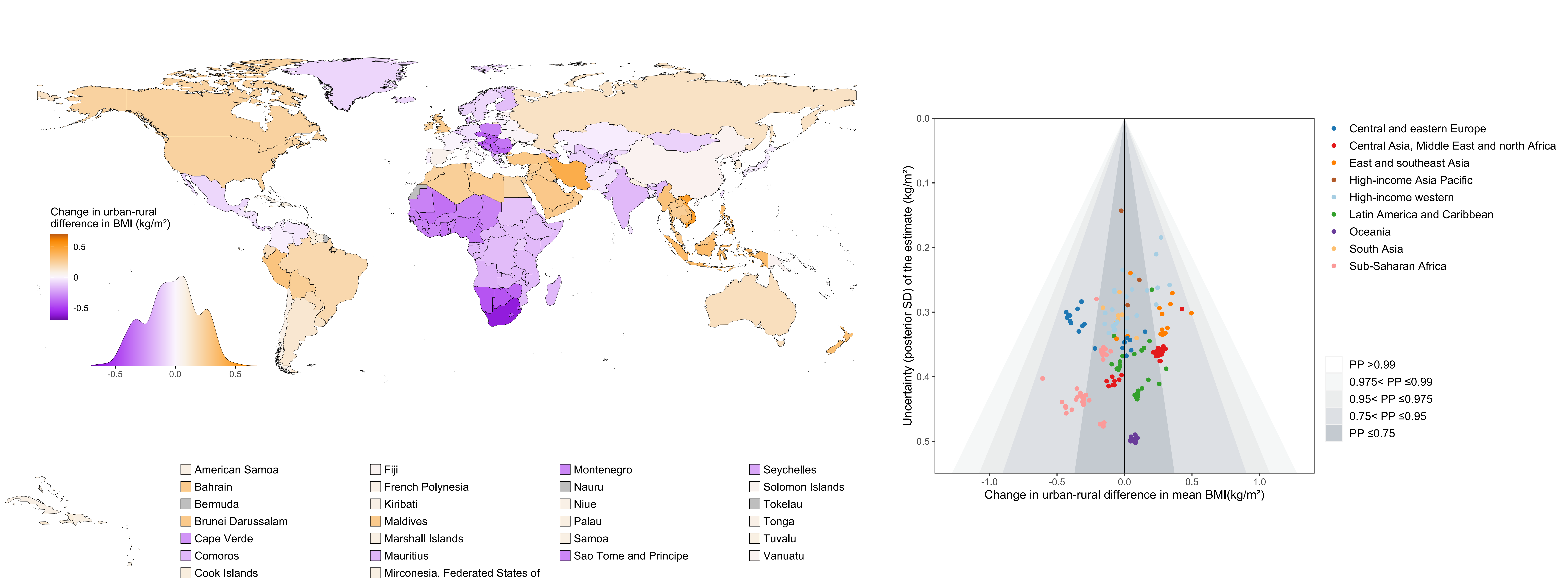

Urban-rural difference in 2020 (girls, age 10)

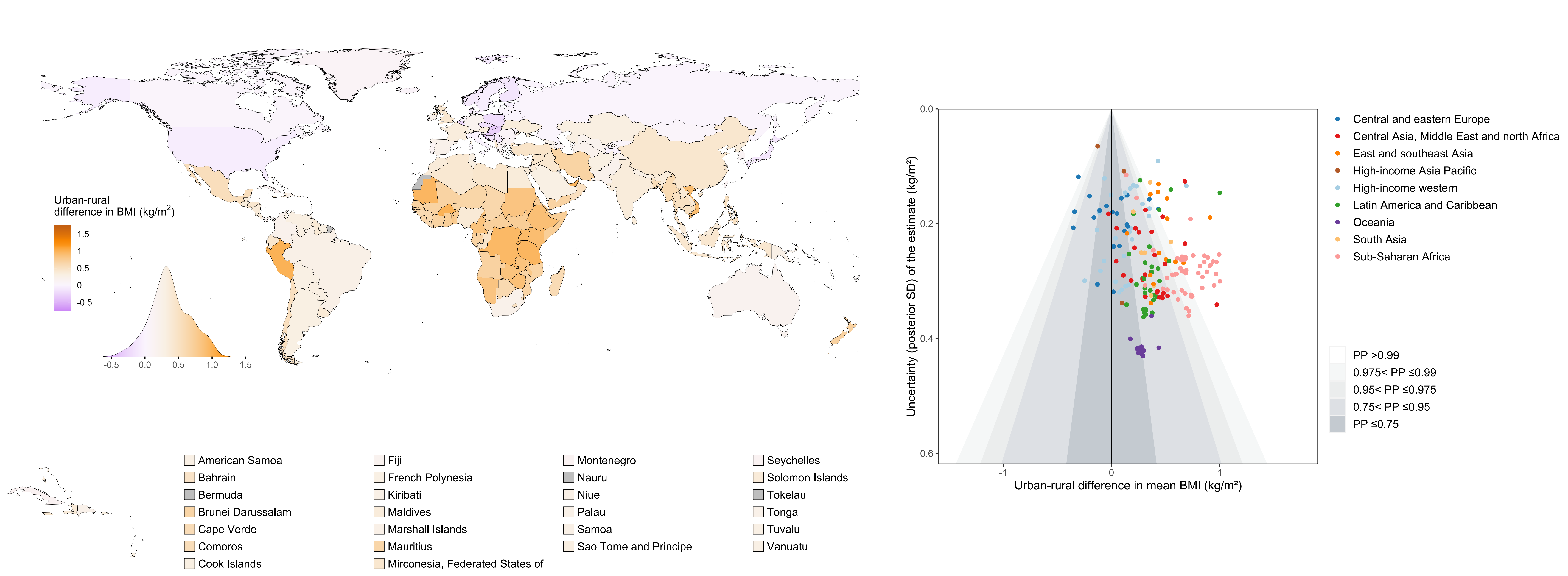

Change 1990-2020 (girls, age 10)

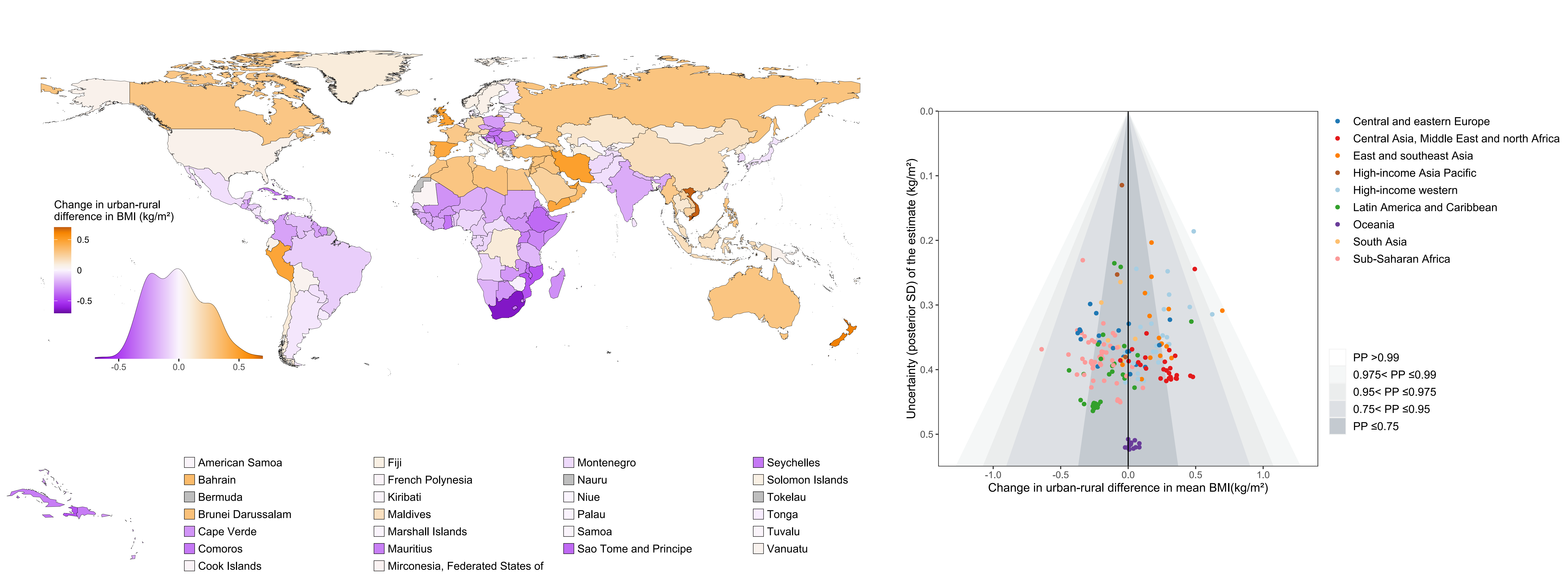

Urban-rural difference in 2020 (boys, age 10)

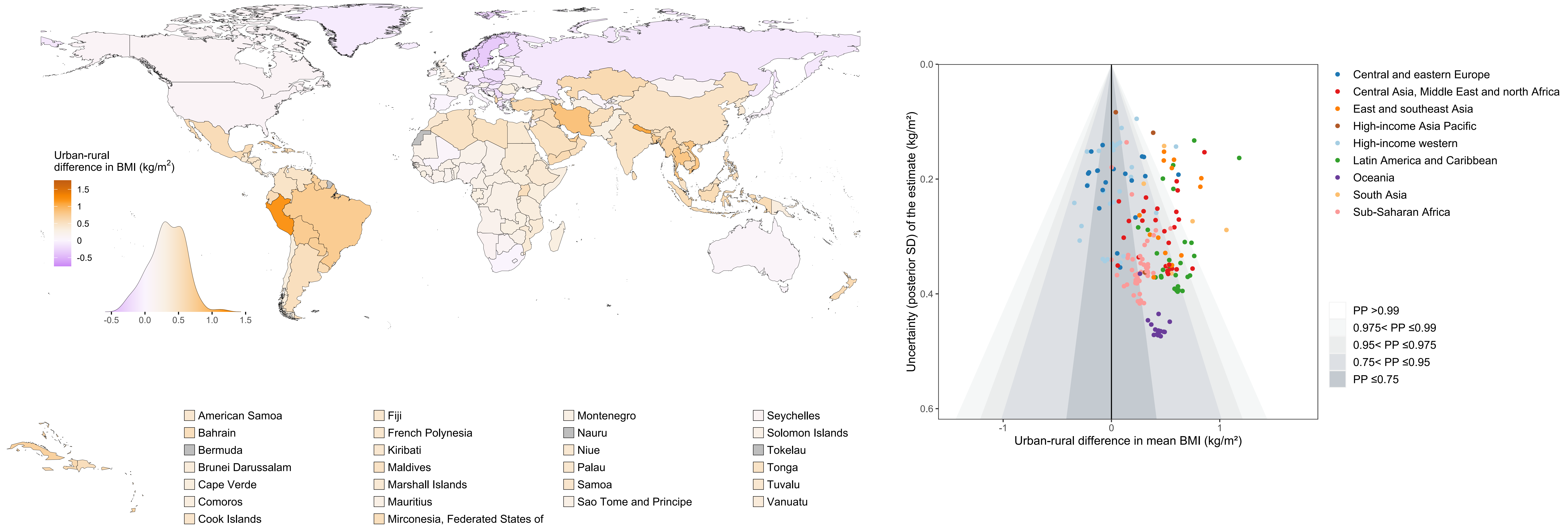

Change 1990-2020 (boys, age 10)

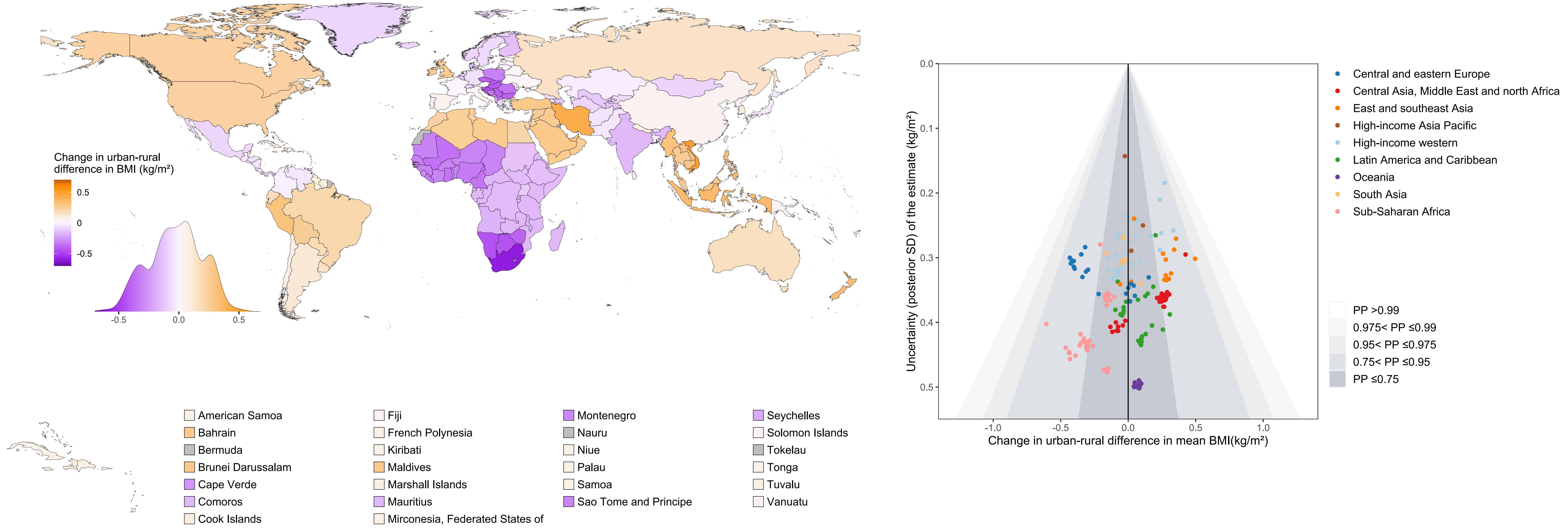

Urban-rural difference in 2020 (girls, age 15)

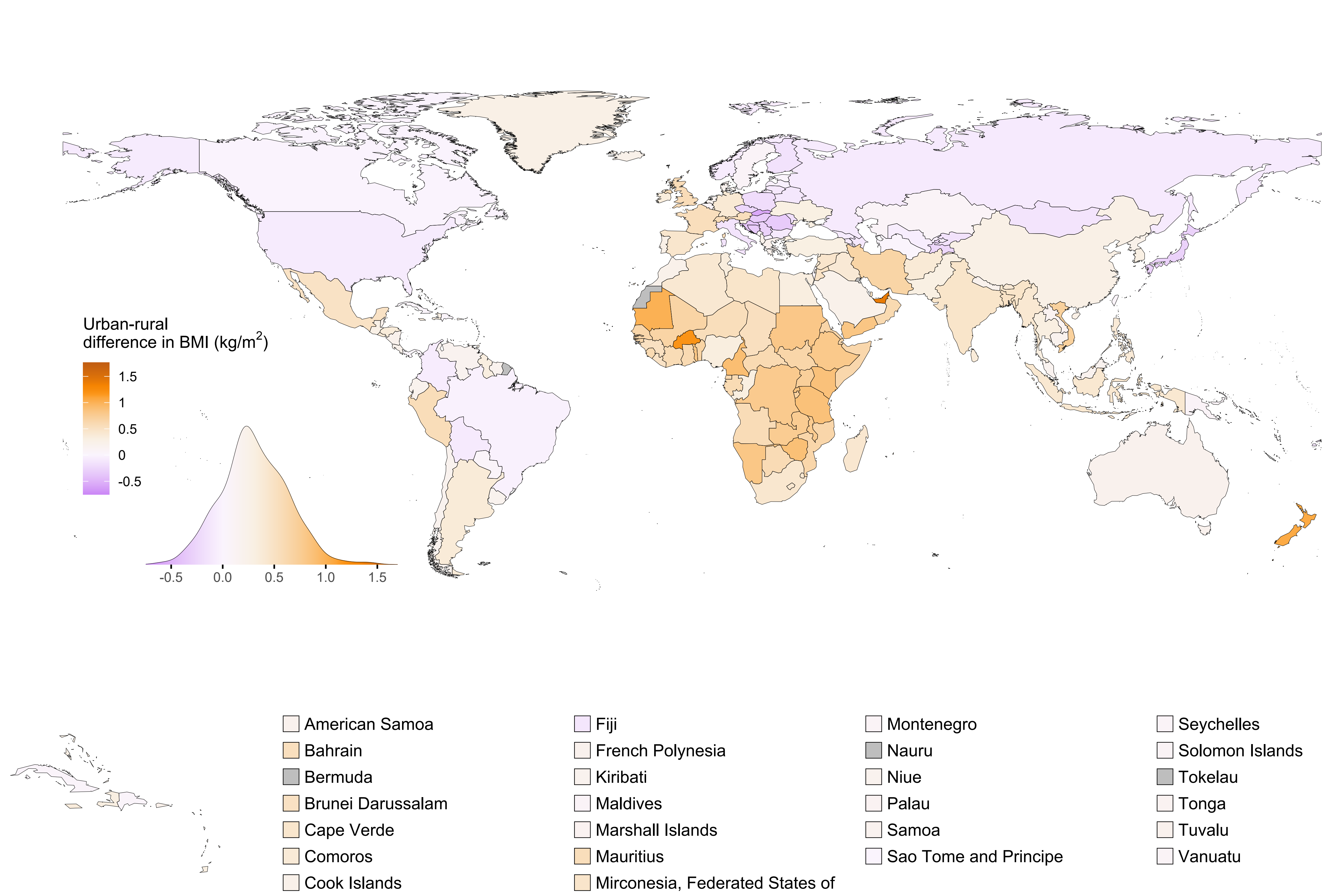

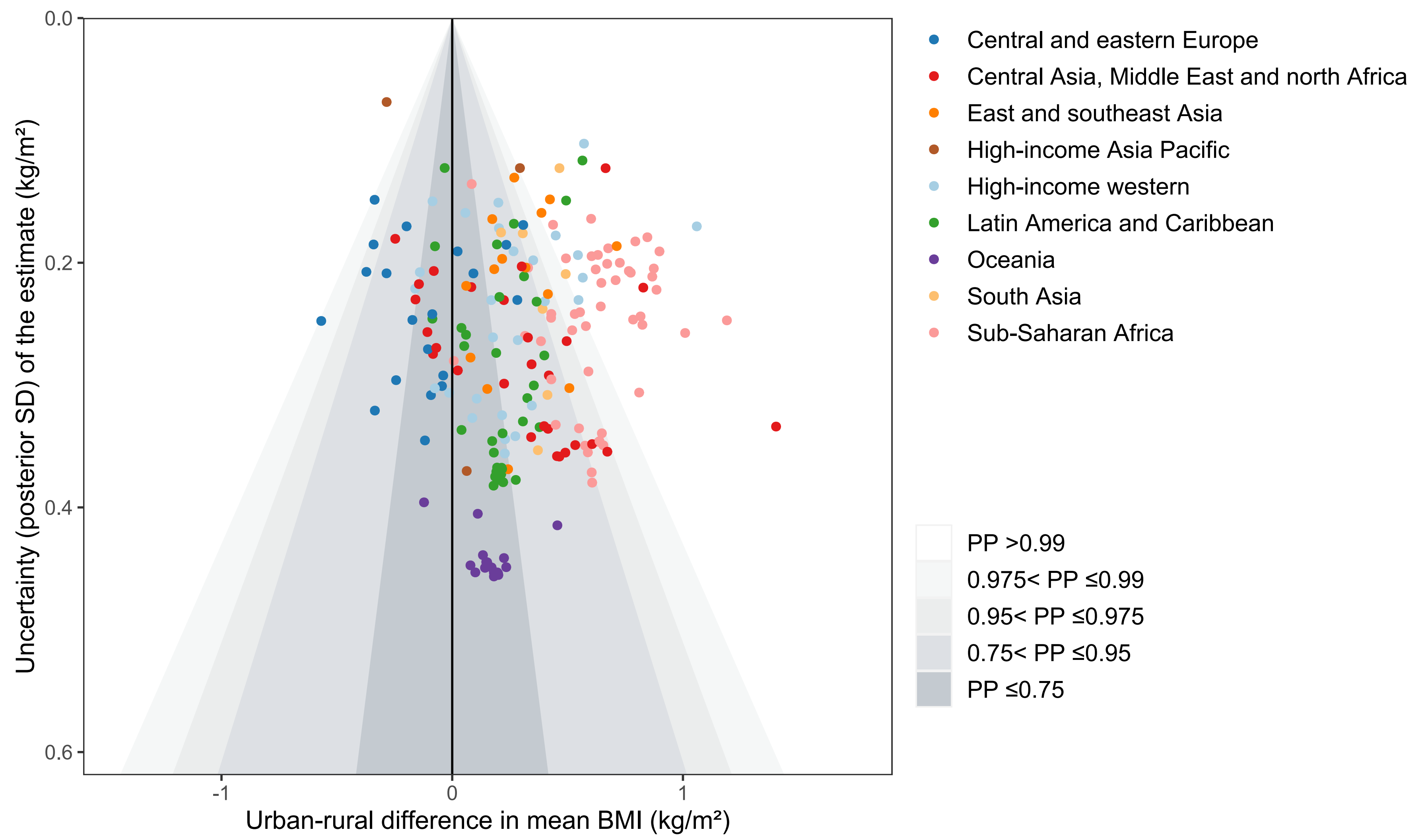

Change 1990-2020 (girls, age 15)

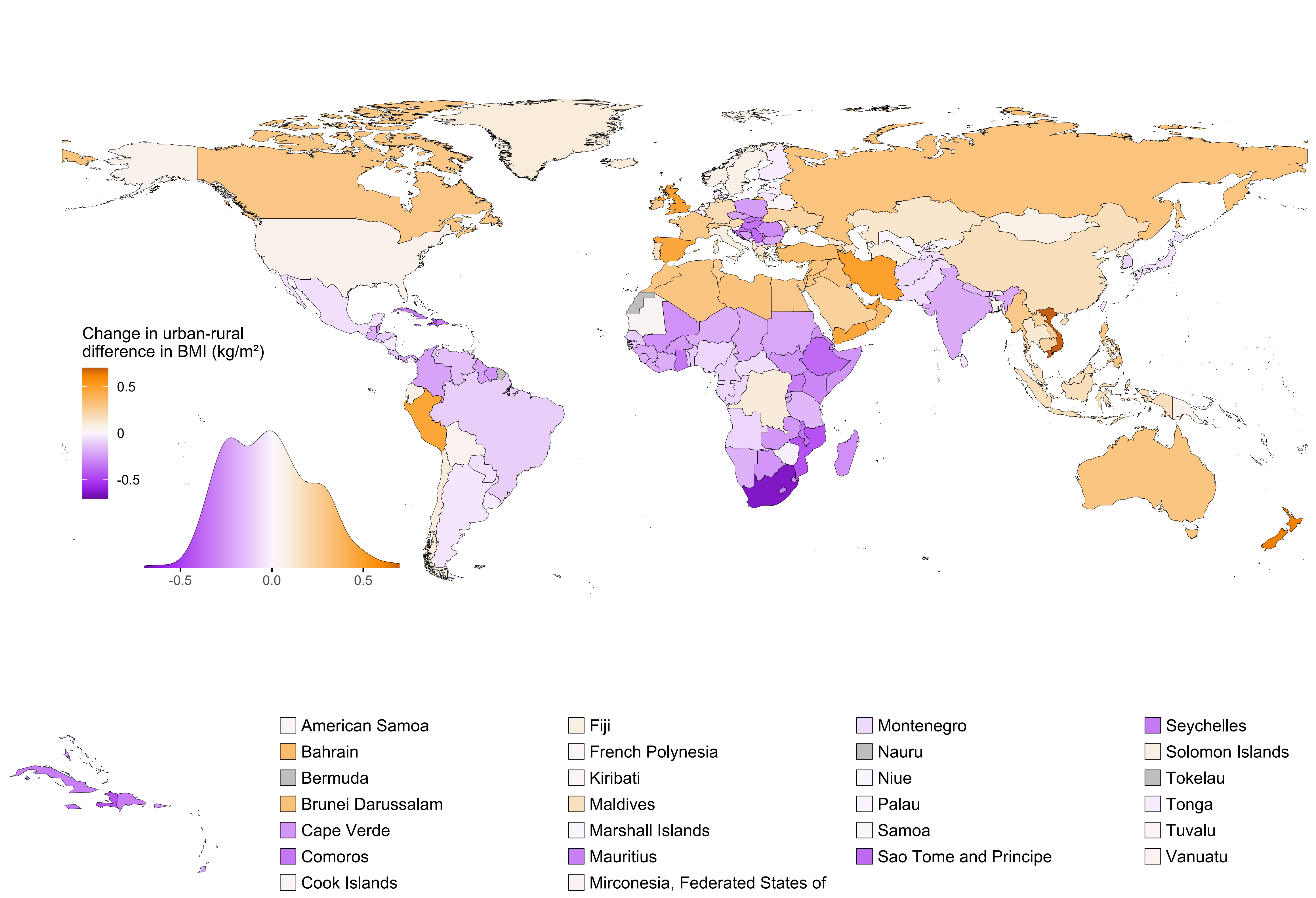

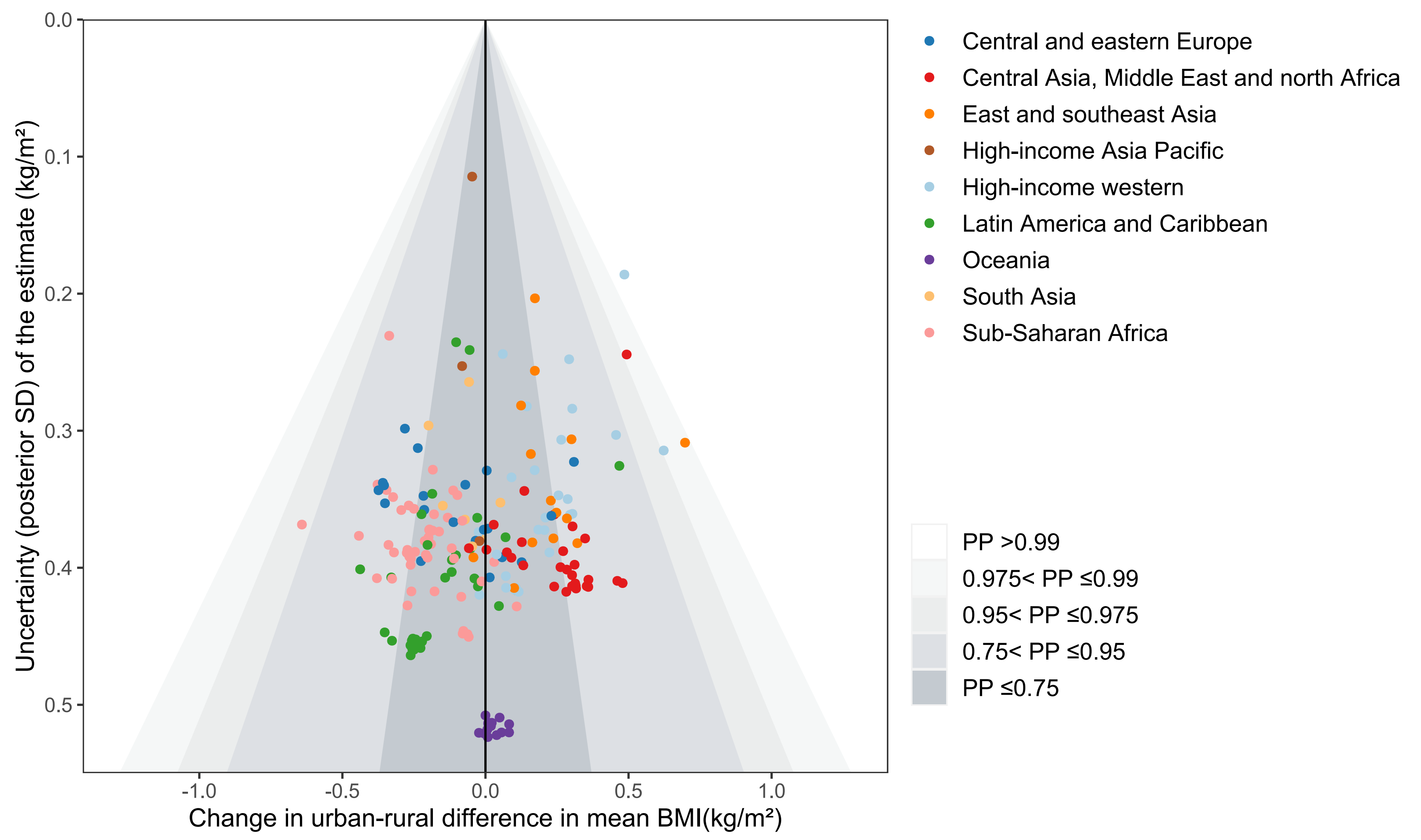

Urban-rural difference in 2020 (boys, age 15)

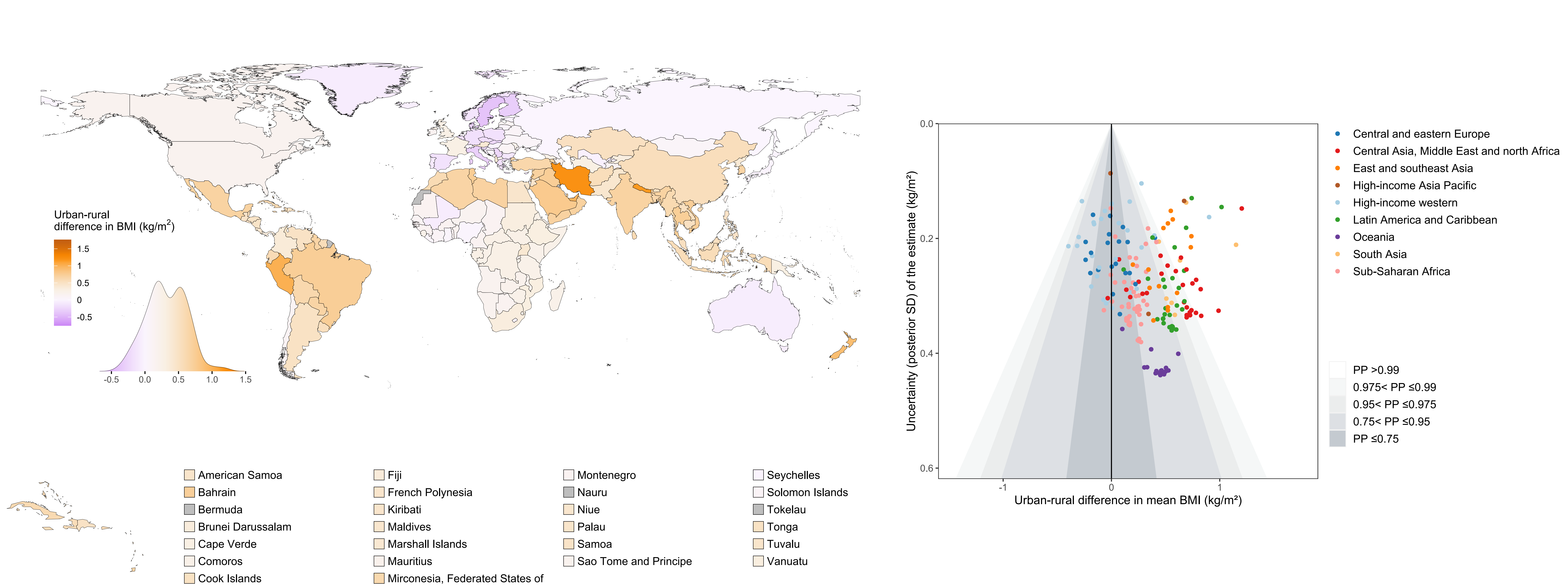

Change 1990-2020 (boys, age 15)

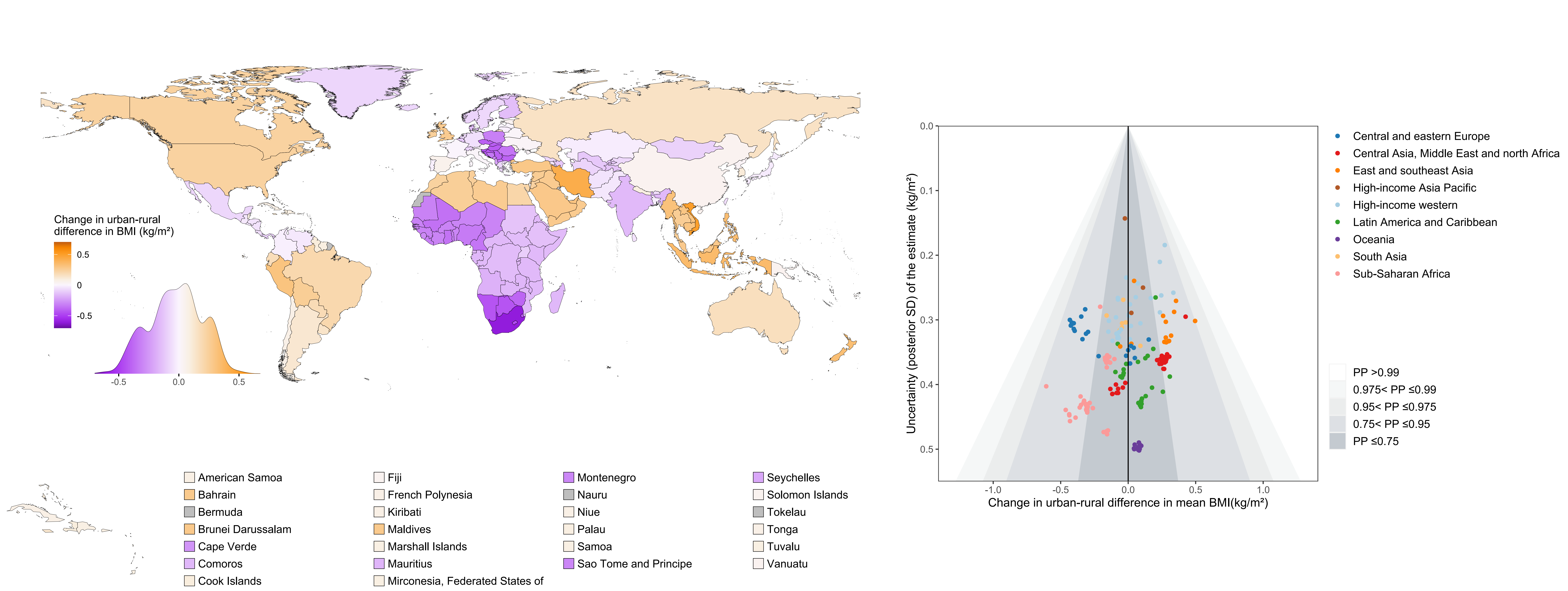

Urban-rural difference in 2020 (girls, age 19)

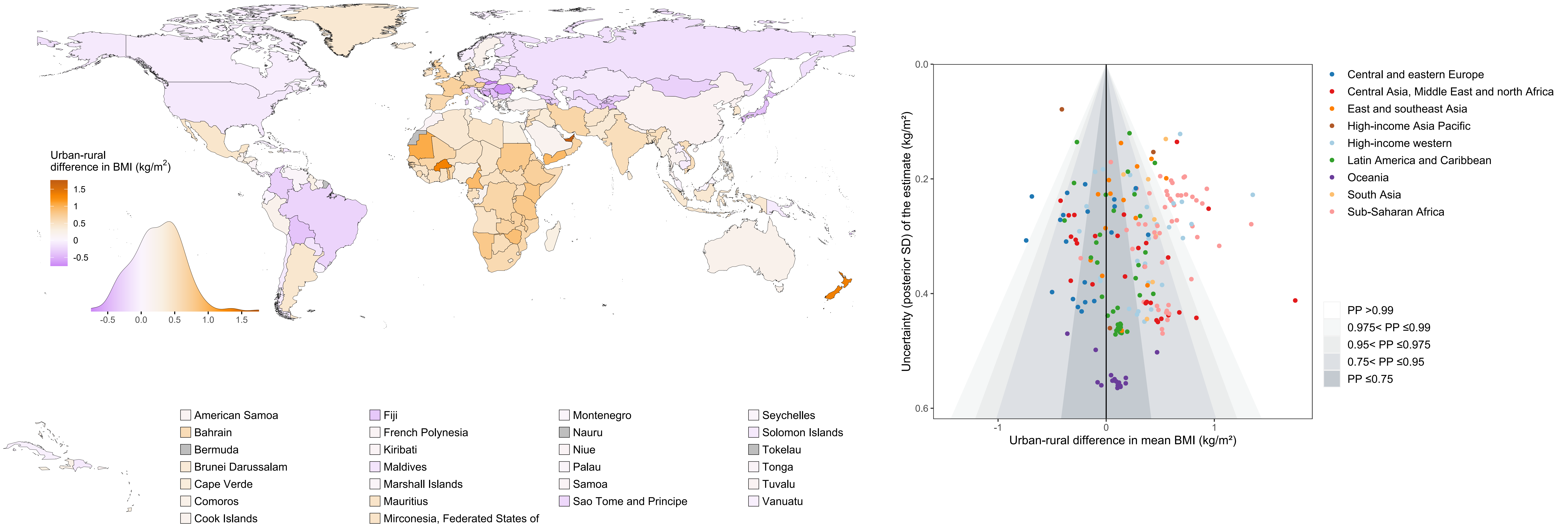

Change 1990-2020 (girls, age 19)

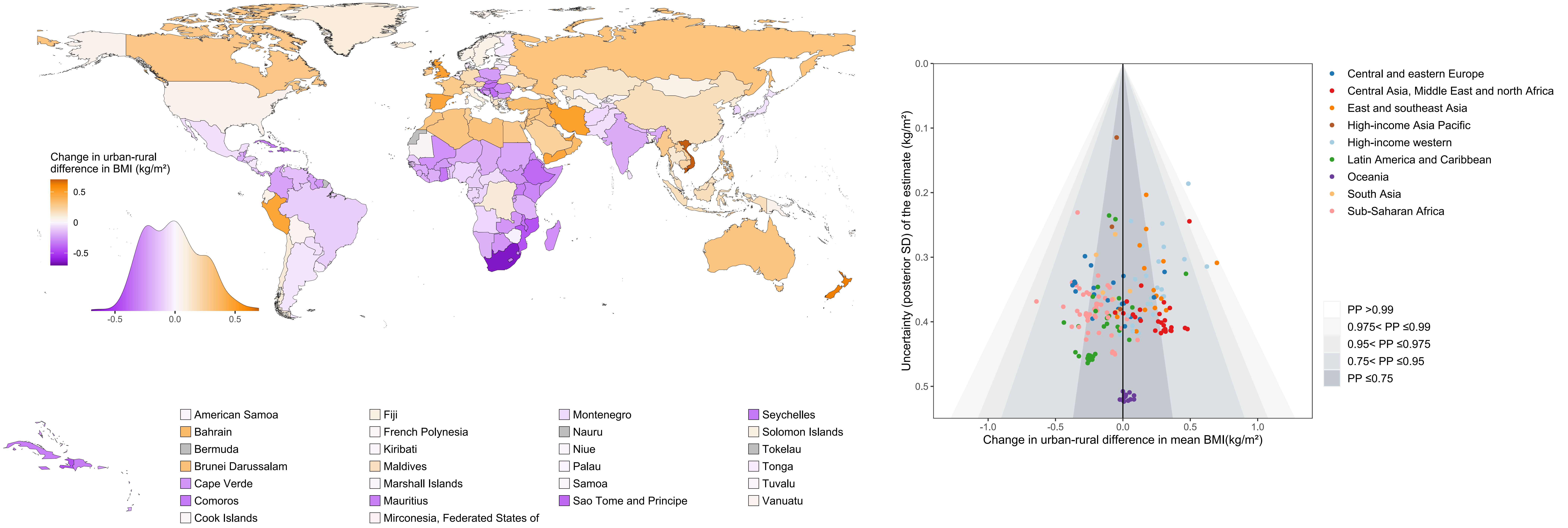

Urban-rural difference in 2020 (boys, age 19)

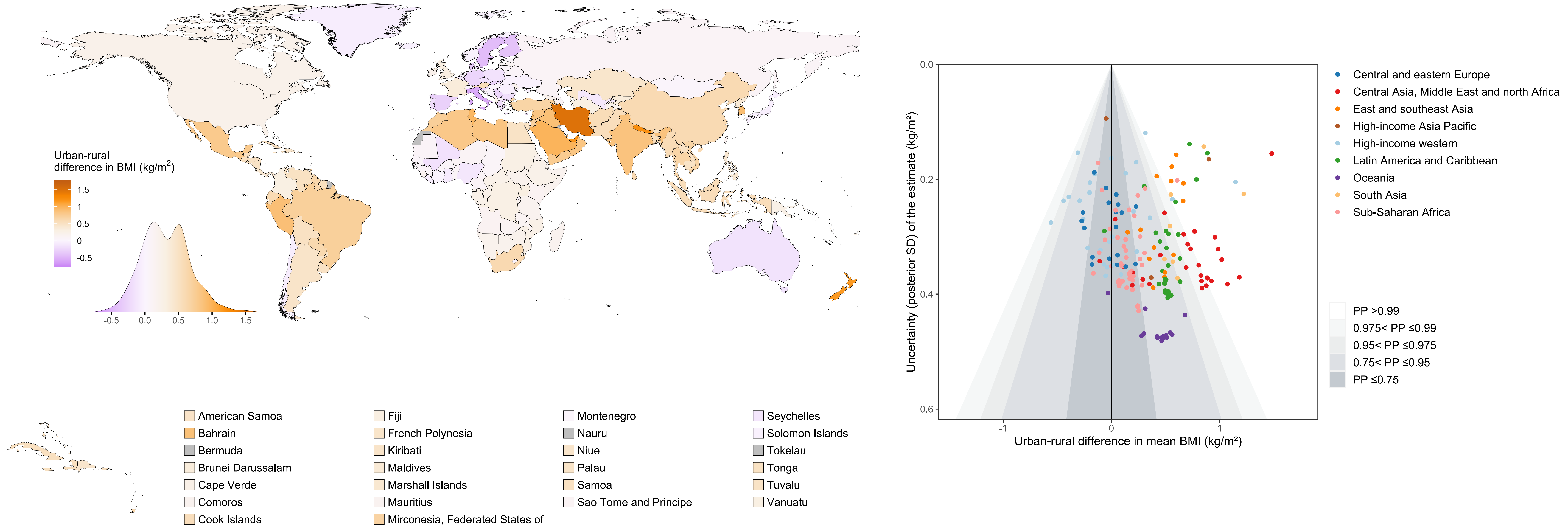

Change 1990-2020 (boys, age 19)

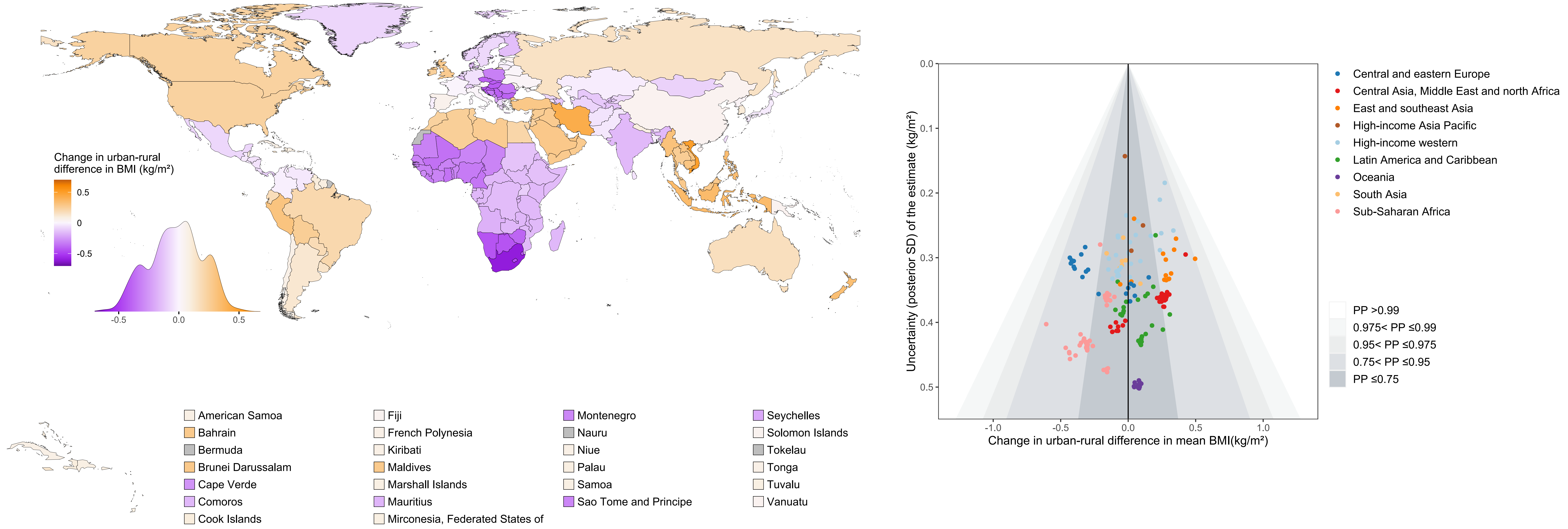

References

1 NCD Risk Factor Collaboration (NCD-RisC). Repositioning of the global epicentre of non-optimal cholesterol. Nature 582, 73-77 (2020). https://doi.org:10.1038/s41586-020-2338-1

2 NCD Risk Factor Collaboration (NCD-RisC). Trends in adult body-mass index in 200 countries from 1975 to 2014: a pooled analysis of 1698 population-based measurement studies with 19·2 million participants. Lancet 387, 1377-1396 (2016). https://doi.org:10.1016/s0140-6736(16)30054-x

3 NCD Risk Factor Collaboration (NCD-RisC). Rising rural body-mass index is the main driver of the global obesity epidemic in adults. Nature 569, 260-264 (2019). https://doi.org:10.1038/s41586-019-1171-x

4 NCD Risk Factor Collaboration (NCD-RisC). Height and body-mass index trajectories of school-aged children and adolescents from 1985 to 2019 in 200 countries and territories: a pooled analysis of 2181 population-based studies with 65 million participants. Lancet 396, 1511-1524 (2020). https://doi.org:10.1016/s0140-6736(20)31859-6

5 NCD Risk Factor Collaboration (NCD-RisC). Worldwide trends in hypertension prevalence and progress in treatment and control from 1990 to 2019: a pooled analysis of 1201 population-representative studies with 104 million participants. Lancet 398, 957-980 (2021). https://doi.org:10.1016/s0140-6736(21)01330-1
